# Synthesis-Supported GC–MS/Retention-Index Library of Hydroxythymol Diesters for Analysis of Botanical Matrices

**DOI:** 10.3390/plants15213430

**Published:** 2026-11-04

**Authors:** Milan Ž. Dimitrijević, Niko S. Radulović

**Affiliations:** Department of Chemistry, Faculty of Sciences and Mathematics, University of Niš, Višegradska 33, 18000 Niš, Serbia; milan.dimitrijevic@pmf.edu.rs

**Keywords:** GC–MS, retention indices, hydroxythymol diesters, regioisomers, essential oil, *Doronicum columnae*, structure–retention relationships

## Abstract

Reliable identification of volatile constituents in complex natural matrices by gas chromatography–mass spectrometry (GC–MS) remains challenging when closely related regioisomeric and acyl-homologous compounds display similar mass spectra and inconsistent retention-index (RI) data. This study aimed to develop a synthetic GC–MS/RI reference library of diacylated hydroxythymol derivatives and apply it to the characterization of thymol-derived constituents in the root essential oil of *Doronicum columnae* (Asteraceae). A total of 49 hydroxythymol diesters bearing isobutyryl, 2-methylbutyryl, and isovaleryl residues were included in the library, covering all accessible regioisomeric combinations within the selected set of six positions of additional oxygenation/acylation on the thymol scaffold (C-2, C-5, C-6, C-7, C-8, and C-9); 44 of these compounds have not previously been described. RI values were determined under unified conditions on non-polar DB-5MS and polar HP-Innowax columns, and the compounds were characterized by GC–MS and, when applicable, nuclear magnetic resonance (NMR) spectroscopy. The dataset revealed clear structure–retention relationships, with positional isomerism exerting a stronger influence on retention than acyl-residue variation. Correlation analysis and a semi-empirical model ester subtraction approach revealed regular structure–retention trends within defined regioisomeric series. Application of the synthetic reference library, supported by dual-column RI data, mass spectral comparison, and co-injection, enabled the identification of 16 diacylated hydroxythymol derivatives in the more polar fraction of *D. columnae* root essential oil, 12 of which are reported here for the first time as natural constituents. The developed approach provides an analytical framework for distinguishing thymol-derived esters in complex botanical matrices.

## 1. Introduction

Gas chromatography–mass spectrometry (GC–MS) is one of the most widely used techniques for the analysis and identification of volatile organic compounds in complex mixtures. Owing to its high separation power and the availability of extensive mass spectral libraries, GC–MS is routinely applied to the characterization of essential oils and other natural product matrices. Nevertheless, reliable identification remains challenging, especially for minor constituents and structurally related isomers, which often display highly similar mass spectra and closely related retention characteristics [1,2].

A major limitation of GC–MS-based identification arises from the dependence on spectral libraries and retention index (RI) data, which are often incomplete, inconsistent, or generated under different experimental conditions. This problem becomes particularly pronounced in the case of positional and structurally related isomers, where subtle structural differences may not be adequately reflected in electron ionization mass spectra. Consequently, ambiguous assignments and misidentifications are not uncommon in the analysis of complex natural mixtures [1,2,3].

Beyond their relevance in natural product chemistry and their reported biological activities, thymol-derived compounds are also of broader interest owing to the well-established role of thymol in food chemistry [4,5]. Thymol is widely recognized as a flavoring substance [4] and has attracted considerable attention because of its antimicrobial and antioxidant properties [5], which have led to applications in food preservation, active packaging systems, edible coatings, and shelf-life extension studies [5,6]. Structural modification of thymol through oxygenation and esterification can substantially alter volatility, lipophilicity, stability, and sensory characteristics. Consequently, hydroxythymol diesters may be regarded as structurally diverse analogues of a food- and flavor-relevant monoterpenoid scaffold. Reliable identification of such compounds is therefore important not only in phytochemical investigations but also in the characterization of botanicals, flavoring materials, herbal preparations, essential-oil-containing food supplements, and other plant-derived products, where closely related thymol derivatives may occur as minor constituents.

Thymol-derived compounds represent a prominent class of oxygenated monoterpenoids widely distributed within the Asteraceae family, particularly in underground plant organs and their essential oils [3,7,8,9,10,11,12,13,14]. Numerous studies have documented structurally diverse thymol derivatives, including simple esters, ether–esters, and diacylated derivatives, in genera such as *Arnica*, *Telekia*, *Carpesium*, *Pulicaria*, *Helenium*, and related taxa [3,7,8,9,10,11,12,13,14]. These compounds frequently constitute major or characteristic constituents of root essential oils and contribute substantially to their chemical complexity [3,7,8,9,10,11,12,13,14].

Hydroxythymol diesters contribute to the structural diversity of Asteraceae volatiles through variation in both the position of additional oxygenation and the identities of the two acyl residues [7,8,9,10]. Reliable differentiation of these constituents is relevant to comparative phytochemical studies of medicinal and aromatic plants. Their low abundance, closely similar EI mass spectra, limited chromatographic separation and incomplete reference data can prevent identification by routine GC–MS library searches. Structurally supported reference data are therefore needed before their distribution or possible value in botanical fingerprints can be assessed.

In addition to positional variation on the thymol core, the nature of the acyl substituent represents an important source of structural diversity. In plants, low-molecular-weight esters commonly incorporate short branched-chain acyl groups such as isobutyryl, 2-methylbutyryl, and isovaleryl moieties, which arise biosynthetically from branched-chain amino acid catabolism via the corresponding acyl-CoA intermediates. Transfer of these acyl residues onto suitable alcohol acceptors by alcohol acyltransferases generates homologous series of structurally related esters, often differing only in the identity of the acyl group. Such variation is directly relevant to thymol-derived esters and contributes further to the analytical complexity of these mixtures [15,16,17].

Several positional isomers of hydroxythymol diesters have already been reported in the literature. Earlier studies described complex mixtures of thymol esters in *Gaillardia* and *Helenium* species, where several regioisomeric diesters were identified, including 7-isobutyryloxythymyl isobutyrate and 7-(2-methylbutyryloxy)thymyl isobutyrate, often occurring as inseparable mixtures [11]. In addition, related 9-substituted thymol diesters, such as 9-isobutyryloxythymyl isobutyrate, were also reported in these early phytochemical investigations, including studies on *Helenium* species [11,12] as well as related Asteraceae taxa such as *Schkuhria* [13]. More specifically, root essential oils of *Arnica montana* were shown to contain a series of bifunctional thymol derivatives, including 7-isobutyryloxythymyl isobutyrate, 9-isobutyryloxythymyl isobutyrate, 9-(2-methylbutyryloxy)thymyl isobutyrate, together with corresponding methyl ethers and isovaleryloxy analogues [7]. A homologous series of thymol esters differing in the acyl moiety (isobutyryloxy vs. 2-methylbutyryloxy) was further observed in *Telekia speciosa* [8]. Similarly, essential oils of *Carpesium divaricatum* and *C. cernuum* were found to contain structurally related 7-, 9-, and 10-substituted thymol diesters, including epoxidized and dehydrogenated derivatives [9,10]. Additional oxygenated thymol derivatives, including thymyl isobutyrate and methoxylated thymyl esters, have been reported from *Pulicaria* species and related taxa [14]. Notably, certain compounds corresponding to 9-isobutyryloxythymyl isobutyrate have been described using an alternative numbering system as 10-isobutyryloxythymyl isobutyrate, reflecting inconsistencies in thymol substitution nomenclature across different studies.

Despite their frequent occurrence, the identification of thymol-derived esters remains difficult. These compounds are commonly present as complex mixtures of closely related positional isomers and acyl homologues, often differing only in the location of oxygenation and/or the nature of the acyl residue. As a result, chromatographic separation may be limited, while their mass spectra remain highly similar. In many published studies, such compounds were assigned based on limited spectral data, approximate RI agreement, or comparison with incompletely characterized literature precedents [1,2,7,8,9,10].

Additional difficulties arise from discrepancies in published analytical data for ostensibly identical compounds. For example, certain thymol diesters reported in the literature, including 7-isobutyryloxythymyl isobutyrate, show mass spectral patterns that do not fully agree across reports, even though the proposed structures are the same or closely related [9,10]. Such inconsistencies may stem from differences in instrumental conditions, stationary phases, data interpretation, or tentative structural assignments, and they underline the need for systematic studies based on synthetic reference compounds and reproducible chromatographic datasets acquired on both non-polar and polar stationary phases [1,2].

Our recent investigation of the essential oil of *Doronicum columnae* roots showed that the oil is dominated by thymyl isobutyrate and thymyl 2-methylbutyrate, whereas the minor constituents belong mainly to oxygenated methoxy-*p*-cymene derivatives, including 6-methoxythymyl esters, 6-acyloxythymyl methyl ethers, and 9-acyloxythymyl methyl ethers [3]. Importantly, chromatographic separation of the oil furnished a more polar fraction enriched in additional trace constituents, whose mass spectra suggested further thymol-like structures, but whose identities could not be assigned with high confidence by GC–MS alone [3]. This observation indicated that the chemical complexity of the *D. columnae* system extends beyond the methoxy-*p*-cymene derivatives previously characterized and includes structurally related, more highly functionalized aromatic esters [3].

Although the present study does not investigate a food sample directly, the analytical challenge addressed here is relevant to food, flavor, and botanical quality-control analysis. The *D. columnae* essential-oil fraction was used as a chemically complex proof-of-concept botanical matrix to demonstrate how a synthesis-supported GC–MS/RI library can assist in distinguishing closely related thymol-derived esters that may also be encountered in flavoring materials, herbal preparations, essential-oil-containing food supplements, and other complex botanical matrices.

In view of the foregoing, a comprehensive reference dataset covering all relevant positional isomers of diacylated thymol derivatives is still lacking. Although compounds substituted at positions such as C-7 and C-9 have been reported [7,9,10,11], no systematic study has addressed the full set of positional isomers relevant to naturally occurring hydroxythymol diesters under unified chromatographic conditions. Such a dataset is essential for establishing reliable structure–retention relationships, evaluating the diagnostic value of EI mass spectra, and enabling confident identification of related constituents in complex essential oil matrices [1,2].

In the present study, we address these challenges through the systematic synthesis and chromatographic characterization of positional isomers of diacylated hydroxythymol derivatives. A library of compounds was prepared encompassing acyloxylation at different positions of the thymol scaffold (C-2, C-5, C-6, C-7, C-8, and C-9), combined with naturally relevant acyl groups, namely isobutyryl, 2-methylbutyryl, and isovaleryl residues. The resulting analytical set, examined on both a non-polar DB-5MS column and a polar HP-Innowax column, provided retention indices and mass spectral data for a structurally coherent series of regioisomeric diesters. This approach enabled the high-confidence identification of compounds present in the more polar chromatographic fraction of *D. columnae* root essential oil and, more broadly, provides a semi-empirical framework for the GC–MS identification of related thymol-derived esters in complex natural mixtures, including essential oils, herbal preparations, flavoring materials, essential-oil-containing food supplements, and other botanical matrices of potential relevance to flavor, fragrance, and food-supplement analysis.

## 2. Results and Discussion

**Scheme 1:**
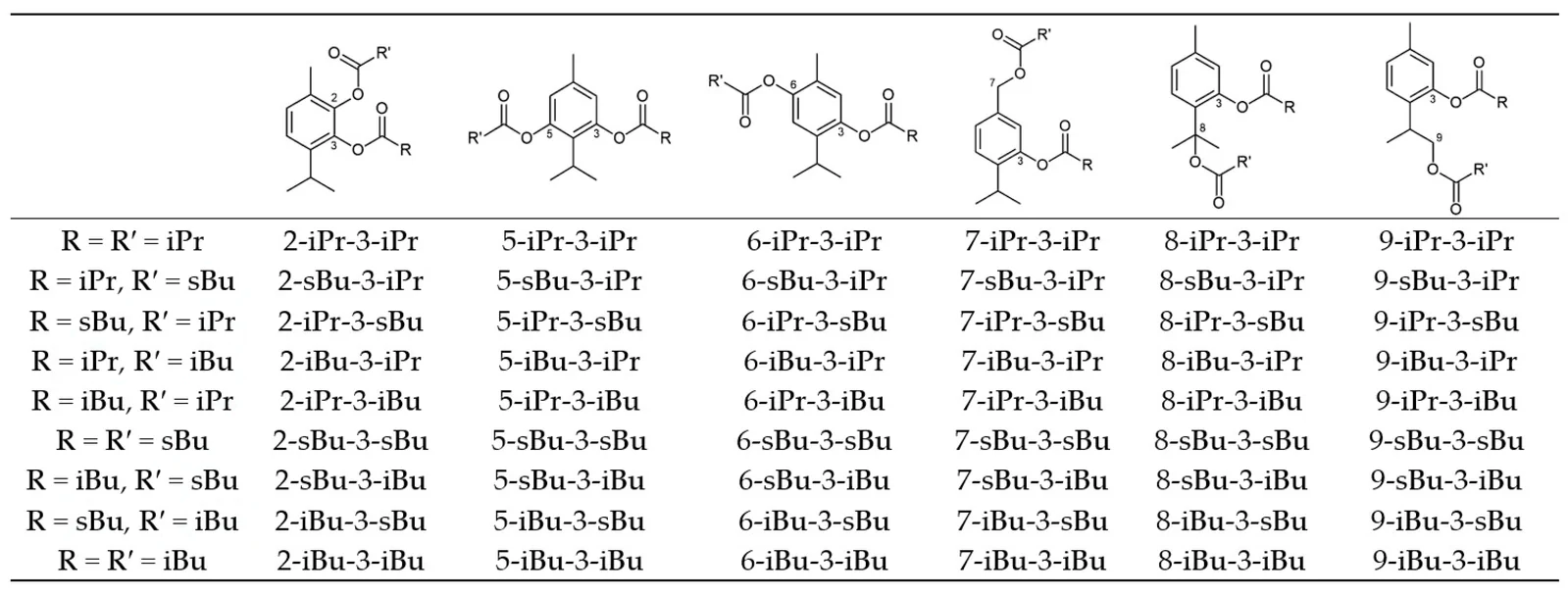
Regioisomeric hydroxythymol diesters investigated in this study and the coding system used throughout the manuscript. In compound codes, iPr, sBu, and iBu denote the alkyl part of the acyl residue RCO–: iPrCO = isobutyryl, sBuCO = 2-methylbutyryl, and iBuCO = isovaleryl. Numbers denote positions on the *p*-cymene skeleton; the final 3-R indicates esterification of the phenolic oxygen at C-3.

To systematically investigate the chromatographic behavior of hydroxythymol diesters and to facilitate the identification of related natural products detected in the root essential oil of *Doronicum columnae*, a comprehensive library of regioisomeric thymol derivatives was prepared. The designed library encompassed combinations of three naturally occurring branched-chain acyl residues, namely isobutyryl, 2-methylbutyryl, and isovaleryl groups, with six different positions of additional oxygenation/acylation on the thymol skeleton (C-2, C-5, C-6, C-7, C-8, and C-9, Scheme 1). This approach generated a series of closely related regioisomeric and acyl-homologous compounds differing only in the position of oxygenation and/or the structure of the acyl substituent.

The numbering and coding system used throughout this work is shown in Scheme 1. The thymol derivatives are numbered according to the *p*-cymene skeleton used in our previous studies. In this system, the original phenolic hydroxyl group of thymol is attached to C-3; therefore, the suffix 3-R in the compound codes denotes esterification of this phenolic hydroxyl group. The prefixes 2-R, 5-R, and 6-R denote additional acyloxy substituents located on the aromatic ring, whereas 7-R, 8-R, and 9-R denote acyloxy substituents located on the methyl group, the tertiary benzylic carbon of the isopropyl group, and the terminal carbon of the isopropyl side chain, respectively. Thus, a code such as 7-iPr-3-sBu denotes a 7-acyloxythymyl ester bearing an iPrCO residue at C-7 and an sBuCO residue on the phenolic oxygen at C-3. In compound codes, iPr, sBu, and iBu refer to the alkyl part of the acyl residue RCO–, corresponding to iPrCO = isobutyryl, sBuCO = 2-methylbutyryl, and iBuCO = isovaleryl. This numbering should be distinguished from alternative systems used in the literature, where some 9-substituted thymol derivatives have been described as 10-substituted analogues.

The 2-acyloxythymyl esters were prepared from thymol through ortho-formylation followed by Dakin oxidation, which afforded 2-hydroxythymol as a key intermediate for subsequent esterification reactions (Scheme S1, Figures S1–S31, Tables S1–S7). Esterification with isobutyric, 2-methylbutyric, and isovaleric acids under Steglich conditions consistently yielded symmetrical 2-acyloxythymyl esters and mixtures of the corresponding regioisomeric monoesters, namely 2-acyloxythymols and 2-hydroxythymyl esters, in an approximately 55:45 ratio, respectively, as determined by integration of diagnostic signals in the ^1^H NMR spectra. Attempts to separate monoesters by silica gel chromatography were unsuccessful, as essentially identical ratios were observed throughout all collected fractions. Such behavior may indicate rapid acyl migration between the adjacent oxygen atoms, resulting in a persistent equilibrium mixture. Subsequent esterification of these monoester mixtures afforded the corresponding unsymmetrical diesters, for which chromatographic fractionation produced fractions enriched in one or the other regioisomer. Although complete separation could not be achieved because of their very similar polarities, the relative abundance of the two diesters varied systematically across the chromatographic fractions. The less strongly retained regioisomer predominated in the early fractions, whereas the more strongly retained analogue became enriched in the later fractions, with the maximum enrichment reaching approximately 3:2.

The synthesis of the 5-acyloxythymyl esters commenced from orcinol, which was converted into 2-acetyl-5-methylresorcinol through sequential acetylation and Fries rearrangement (Scheme S2, Figures S32–S42, Table S8). Subsequent reaction with methylmagnesium iodide afforded 5-hydroxydehydrothymol, which was hydrogenated to give 5-hydroxythymol as the key intermediate. Esterification of 5-hydroxythymol with isobutyric, 2-methylbutyric, and isovaleric acids under Steglich conditions provided the corresponding monoesters, symmetrical diesters, and mixed diesters.

The 6-acyloxythymyl esters were prepared from thymohydroquinone through Steglich esterification with isobutyric, 2-methylbutyric, and isovaleric acids (Scheme S3, Figures S43–S71, Tables S9–S12). In contrast to the 2-substituted series, esterification afforded regioisomeric monoesters that could be readily separated by silica gel chromatography, allowing the individual isolation of both 6-acyloxythymols and 6-hydroxythymyl esters. No evidence of acyl migration or equilibration between the two monoester forms was observed. The isolated monoesters were subsequently subjected to further esterification with the corresponding branched-chain carboxylic acids, providing access to a complete series of symmetrical and mixed diesters. The availability of both regioisomeric monoesters as pure compounds enabled the controlled preparation and reliable characterization of the corresponding 6-acyloxythymyl esters, which were included in the retention-index database.

The synthesis of the 7-acyloxythymyl esters was based on the selective esterification of 7-hydroxythymol (Scheme S4, Figures S72–S125, Tables S13–S21). Under the applied Steglich conditions, acylation occurred preferentially at the aliphatic hydroxyl group located at C-7, affording the corresponding 7-acyloxythymols together with the respective symmetrical diesters. In contrast to the 2- and 6-substituted series, no regioisomeric monoester pair was observed, reflecting the substantially higher reactivity of the benzylic hydroxyl group relative to the phenolic hydroxyl function. The isolated 7-acyloxythymols served as key intermediates for the preparation of mixed diesters, which were obtained by subsequent esterification of the remaining phenolic hydroxyl group (C-3) with the appropriate branched-chain carboxylic acids. This strategy provided access to both symmetrical and unsymmetrical members of the 7-substituted thymol diester series.

The synthesis of the 8-acyloxythymyl esters commenced from 8-hydroxythymol, which was obtained by Grignard addition of methylmagnesium iodide to 2-hydroxy-4-methylacetophenone (Scheme S5, Figures S126–S136, Table S22). In contrast to the 2-, 6-, and 7-substituted series, the target 8-substituted diesters were prepared by deprotonation of 8-hydroxythymol with sodium hydride, followed by direct acylation of the resulting dianion with the corresponding acid chlorides. The 8-substituted series proved to be the most challenging synthetically. Although 8-hydroxythymol was obtained in good yield, subsequent acylation did not allow isolation of the corresponding monoesters. Instead, the reactions predominantly afforded diacylated products, which proved difficult to purify and were susceptible to partial decomposition during isolation. Consequently, only 8-isobutyryloxythymyl isobutyrate was isolated in pure form and fully characterized by spectroscopic methods, whereas the remaining 8-substituted diesters were generated as reference GC–MS components and used primarily for the acquisition of EI-MS and retention-index data. Their assignments were supported by reaction logic, chromatographic behavior, and diagnostic EI mass spectral fragmentation, with the corresponding mass spectra provided in the Supplementary Information.

For the mixed diesters, structural assignment relied largely on characteristic EI mass spectral fragmentation. The observed mass spectra closely resembled those of the corresponding 8,9-dehydrothymyl esters and were found to be diagnostic for the acyl group attached to the phenolic oxygen atom at C-3. Thus, the mass spectrum of 8-isobutyryloxythymyl 2-methylbutyrate closely matched that of 8,9-dehydrothymyl 2-methylbutyrate, supporting assignment of the 2-methylbutyryl residue to the phenolic oxygen. This fragmentation behavior supported the proposed acyl-group assignments, although the mixed C-8 derivatives were not independently confirmed by NMR spectroscopy. Two mixed derivatives, namely 8-isovaleroxythymyl 2-methylbutyrate and 8-(2-methylbutyryloxy)thymyl isovalerate, could not be obtained under the applied reaction conditions; therefore, no chromatographic or mass spectral data are available for these compounds.

The reference library contains entries supported by different levels of structural evidence. Within the C-8 series, 8-iPr-3-iPr was isolated and characterized by NMR spectroscopy, whereas the other six entries were assigned as components of synthetic mixtures from their synthetic origin and chromatographic and EI-MS behavior. Similar distinctions apply to other derivatives prepared only for GC–MS analysis and to the composite spectrum of the co-eluting C-2 pair in Figure S24. No C-8 derivative was assigned among the natural constituents of the investigated fraction; the reference structures used for the natural-constituent assignments are supported by the NMR data in Tables S9–S11, S13–S21, and S23–S26.

The synthesis of the 9-acyloxythymyl esters followed a strategy analogous to that employed for the 7-substituted series. The key intermediate, 9-hydroxythymol, was obtained through a multistep sequence involving Pechmann condensation of *m*-cresol with methyl acetoacetate to afford 4,7-dimethylcoumarin, followed by alkaline ring opening and decarboxylation to give 8,9-dehydrothymol (Scheme S6, Figures S137–S165, Tables S23–S26). Subsequent hydroboration–oxidation of the double bond furnished 9-hydroxythymol, which was subjected to Steglich esterification with isobutyric, 2-methylbutyric, and isovaleric acids. Under these conditions, acylation occurred preferentially at the primary hydroxyl group located at C-9, affording the corresponding 9-acyloxythymols together with the respective symmetrical diesters. The isolated monoesters served as key intermediates for the preparation of mixed diesters through subsequent esterification of the remaining phenolic hydroxyl group at C-3. In contrast to the 8-substituted series, the monoesters proved sufficiently stable to allow isolation and full spectroscopic characterization. This stepwise approach provided access to both symmetrical and unsymmetrical members of the 9-substituted thymol diester series.

Using this strategy, a total of 49 diacylated hydroxythymol derivatives were included in the synthetic GC–MS/RI reference library (Scheme 1). Of these, 44 represent previously undescribed compounds. Their retention indices were determined on both a non-polar DB-5MS (RIa) column and a polar HP-Innowax (RIp) column (Table 1 and Table 2). To the best of our knowledge, this represents one of the most extensive unified chromatographic datasets covering such a broad range of regioisomeric thymol esters analyzed under identical chromatographic conditions.

The generated library included derivatives bearing acyloxy substituents at positions C-2, C-5, C-6, C-7, C-8, and C-9 of the thymol framework, allowing direct comparison of the influence of substituent position on chromatographic retention. Because all compounds were analyzed on both stationary phases, the obtained retention indices provide a valuable reference set for the identification of structurally related natural products in complex mixtures.

Inspection of the retention indices revealed that positional isomerism exerted a greater influence on chromatographic retention than variation of the acyl residue. While replacement of an isobutyryl group by the corresponding C_5_ homologues (2-methylbutyryl or isovaleryl) generally produced moderate increases in retention indices, relocation of the second acyloxy substituent to a different position on the aromatic ring or side chain often resulted in substantially larger shifts in both RIa and RIp values. For clarity, the abbreviations used in compound codes refer to the alkyl group of the corresponding acyl residue RCO– rather than to the complete acyl group.

Comparison of the retention indices revealed that the position of the second acyloxy substituent exerted a pronounced influence on chromatographic behavior. The bis(isobutyryl) derivatives followed the elution order 8 < 2 < 9 < 5 < 6 < 7 on both stationary phases. A broadly similar pattern was observed for other acyl combinations, although several reversals and near-coincidences occurred. These exceptions show that substituent position and acyl-residue identity jointly influence retention. The persistence of this trend on stationary phases of markedly different polarity indicates that retention is governed primarily by intrinsic structural features of the molecules rather than by hydrophobicity alone.

Thus, chromatographic behavior appears to depend largely on the spatial arrangement of the two ester functionalities and the overall molecular geometry. The lowest retention indices were consistently observed for the 8-substituted derivatives, followed closely by the 2-substituted analogues. In both series, the two ester groups are located in close spatial proximity, which may promote more compact molecular conformations and partial steric shielding of the ester functionalities, thereby reducing interactions with the stationary phase. The 9-substituted derivatives displayed intermediate retention behavior, likely reflecting the greater conformational flexibility of the acyloxymethyl side chain. In contrast, the 5-, 6-, and especially 7-substituted derivatives exhibited the highest retention indices. In these compounds, the ester groups are more spatially separated and exposed, increasing the effective molecular surface available for intermolecular interactions. The particularly high retention of the 7-substituted derivatives may additionally be associated with the presence of a benzylic ester side chain, which combines conformational flexibility with a highly accessible ester functionality.

A further notable feature concerns the relative retention of the two C_5_ acyl isomers. In the 5-, 6-, 7-, and 9-substituted series, derivatives containing isovaleryl residues generally showed higher RI values than the corresponding 2-methylbutyryl analogues. In contrast, the opposite trend was observed for the more compact 2- and 8-substituted derivatives, where 2-methylbutyryl-containing compounds consistently eluted after the corresponding isovaleryl analogues. This reversal suggests that the influence of acyl-chain branching depends strongly on the spatial arrangement of the two ester groups and becomes particularly pronounced in sterically constrained regioisomeric series. The α-branched 2-methylbutyryl residue appears to exert a greater influence on molecular shape when the ester functionalities are located in close spatial proximity, as in the 2- and 8-substituted derivatives. Consequently, subtle differences in branching position may affect the effective molecular surface and intermolecular interactions with the stationary phase to a greater extent than in the more extended 5-, 6-, 7-, and 9-substituted analogues.

To further evaluate the structure–retention relationships within this series, the retention data were analyzed using a semi-empirical model ester subtraction approach. In this analysis, the RI value of an appropriate monoester model compound (Table S27) was subtracted from the experimentally determined RI value of the corresponding hydroxythymol diester, giving a residual retention-index increment associated with the second acyloxy substituent:

ΔRIindividual = RIdiester − RImodel(1)

where RIdiester is the experimentally determined retention index of the diester and RImodel is the retention index of the corresponding model monoester. The individual residual increments calculated in this way are provided in Table S28, whereas the mean residual increments are summarized in Table 3.

Although retention indices are not strictly additive, the calculated mean residual increments showed a considerable degree of regularity within several regioisomeric series. Therefore, the expected retention index of a related hydroxythymol diester can be approximated using the following expression:

(2) $$\mathrm{RIest}\;=\;\mathrm{RImodel}\;+\;\bar{\Delta \mathrm{RI}}$$

where RIest is the estimated retention index of the diester and $\bar{\Delta \mathrm{RI}}$ is the mean residual retention-index increment corresponding to the second acyloxy substituent. When two complementary model esters are available, two complementary RI estimates can be obtained for the same diester. Agreement between these estimates provides an additional indication of the reliability of the proposed structural assignment.

The reliability of the increment-based approach was further assessed by calculating the deviation of each individual residual increment from the corresponding mean value:

(3) $$\delta \Delta \mathrm{RI}\;=\;\Delta \mathrm{RIindividual}\;-\;\bar{\Delta \mathrm{RI}}$$

Positive δΔRI values indicate residual increments higher than the corresponding mean value, whereas negative values indicate lower-than-average increments (Table S29). Relatively small deviations were observed for the 5-, 6-, 7-, and 9-substituted series, indicating that the residual increments are largely transferable within these groups. This suggests that, for these derivatives, the contribution of a given acyloxy substituent to the overall retention behavior is reasonably consistent and can be used to estimate the RI values of related analogues.

In contrast, larger deviations were observed for the 2- and 8-substituted derivatives. This lower degree of additivity is consistent with their more compact and sterically constrained molecular arrangements discussed above. In these regioisomeric series, the close spatial proximity of the two ester functionalities may enhance conformational and steric effects, making the retention contribution of each acyloxy group less independent from the rest of the molecule. Therefore, the model ester subtraction approach appears most reliable for the more extended 5-, 6-, 7-, and 9-substituted derivatives, whereas RI estimates for the 2- and 8-substituted series should be interpreted more cautiously.

Overall, this analysis demonstrates that the synthesized library provides not only experimental RI and mass spectral data, but also a semi-empirical framework for rationalizing the retention behavior of related hydroxythymol diesters. The model ester subtraction approach is not intended to replace synthetic reference compounds, dual-column RI data, EI-MS comparison, or co-injection. Rather, it provides a plausibility filter for evaluating tentative assignments when complete reference coverage is unavailable. Agreement between experimentally observed RI values and the estimated RI range derived from model ester increments can therefore serve as an additional, but not standalone, criterion for the GC–MS identification of hydroxythymol diesters.

Correlation analysis of the retention indices determined on DB-5MS and HP-Innowax columns revealed a strong overall relationship between the two datasets. For the complete set of synthesized diesters, the RI values on the polar column correlated well with those obtained on the non-polar column, indicating that the general retention behavior is largely preserved across stationary phases (Figure S166). However, the correlation was not perfect, demonstrating that HP-Innowax does not merely reproduce the DB-5MS elution pattern with a constant offset, but provides additional structural discrimination.

When individual regioisomeric series were considered separately, very high RIa–RIp correlations were observed, showing that changes in the acyl residues affect retention in a largely parallel manner on both columns. The strongest regularity was observed for the 2-, 5-, and 6-substituted series, whereas slightly larger deviations were found for the 7-, 8-, and 9-substituted derivatives. These deviations likely reflect differences in molecular geometry, conformational flexibility, and accessibility of the ester functionalities to the polar stationary phase.

Pairwise correlations between individual regioisomeric series revealed that the 5-, 6-, 7-, and 9-substituted derivatives form the most regular subset. On the DB-5MS column, these series showed very high mutual correlations, with R^2^ values generally above 0.99 (Figure S167). This indicates that homologous variation of the acyl residues produces nearly parallel retention trends within these more extended regioisomeric frameworks. A similar relationship was also observed on the HP-Innowax column, although slightly larger deviations were apparent, particularly in correlations involving the 7-substituted series (Figure S168). These results suggest that, for the 5-, 6-, 7-, and 9-substituted derivatives, the effect of changing the acyl group is largely transferable between different positions of oxygenation/acylation on the thymol scaffold.

The 2-substituted derivatives showed weaker, although still meaningful, correlations with the 5-, 6-, 7-, and 9-substituted series. This effect was moderate on the DB-5MS column, but became more pronounced on the HP-Innowax column, where correlations involving the 2-substituted series showed larger deviations from linearity. This behavior is consistent with the compact ortho arrangement of the two ester functionalities in the 2-substituted derivatives. In such structures, steric and conformational effects may have a greater influence on retention, making the contribution of the acyl residues less directly transferable to the more extended regioisomeric series.

The 8-substituted derivatives also showed high correlations with several other series on the DB-5MS column, particularly with the 6- and 9-substituted derivatives. However, these correlations should be interpreted with some caution because two mixed 8-substituted diesters were not obtained, reducing the number of data points available for comparison. On the HP-Innowax column, the correlations involving the 8-substituted derivatives were generally lower than on DB-5MS, suggesting that the polar stationary phase is more sensitive to the distinctive structural features of this series. This is in agreement with the proposed compact geometry of the 8-substituted derivatives and the presence of an ester group attached to a tertiary carbon atom.

Interestingly, the 2- and 8-substituted series also correlated with one another on the DB-5MS column, supporting the view that both groups share certain retention features associated with compact molecular geometry. Nevertheless, the corresponding correlation on HP-Innowax was weaker, indicating that their interactions with the polar stationary phase are not governed by identical structural factors. Thus, although both series deviate from the more regular 5-, 6-, 7-, and 9-substituted subset, they should not be treated as completely equivalent.

Overall, the correlation analysis confirms that retention behavior within the hydroxythymol diester library is highly structured and partly predictable. The strongest predictive relationships were observed for the 5-, 6-, 7-, and 9-substituted derivatives, whereas the 2- and 8-substituted series displayed controlled but systematic deviations. These findings support the use of subgroup-specific structure–retention relationships rather than a single global correlation for all regioisomeric types. In combination with dual-column RI data and residual increment analysis, this correlation approach provides an additional tool for evaluating tentative assignments of hydroxythymol diesters in complex natural matrices.

Several constituents identified here have precedents in other Asteraceae oils. Related 7- and 9-substituted thymol diesters have been reported in *Arnica*, *Telekia* and *Carpesium*, including 7-isobutyryloxythymyl isobutyrate, 9-isobutyryloxythymyl isobutyrate and related 2-methylbutyryloxy derivatives [7,8,9,10]. These reports differ in chromatographic conditions [11] and in the evidence available for individual assignments, limiting direct comparison of constituent lists. The present reference data facilitate comparison of positional and acyl variants across such matrices. Whether the observed patterns have diagnostic value will require comparable analyses of independently collected samples and related taxa.

The tentative assignments initially suggested by literature comparison were therefore treated only as preliminary. Comparison of the literature data with the present results revealed noticeable differences in retention indices. For example, 7-isobutyryloxythymyl isobutyrate, 9-isobutyryloxythymyl isobutyrate, 9-(2-methylbutyryloxy)thymyl isobutyrate, and 9-isovaleroxythymyl isobutyrate were previously reported with RI values of 1924, 1891, 1970, and 1974, respectively, on a non-polar CP-Sil 5 column [7], whereas the corresponding RI values determined in the present study on DB-5MS were 1961, 1921, 2007, and 2011. Similar, although smaller, differences were also observed on polar stationary phases, where the literature RI values on FFAP were 2556, 2483, 2561, and 2576 [7], compared with 2568, 2497, 2578, and 2583 on HP-Innowax in the present study. Comparable, but expected, discrepancies were also evident when our DB-5MS data were compared with RI values reported on Rtx-1MS and DB-1 columns for *Telekia* and *Carpesium* oils [8,9,10].

These differences are most likely due, at least in part, to the use of different stationary phases, temperature programs, and RI calibration conditions. Nevertheless, the magnitude of the observed deviations emphasizes that literature RI values alone are insufficient for high-confidence identification of closely related hydroxythymol diesters. This is particularly important for positional isomers and acyl homologues, which frequently display closely related EI mass spectra and may occur as minor constituents in complex essential oil fractions.

An additional complication was observed for 7-isobutyryloxythymyl isobutyrate. In the present study, the EI mass spectrum of the synthesized standard showed *m*/*z* 166 as the dominant diagnostic fragment, whereas *m*/*z* 149 was considerably less intense. In contrast, the spectrum reported for the compound assigned as 7-isobutyryloxythymyl isobutyrate in the *Carpesium divaricatum* study displayed *m*/*z* 149 as the base peak, with *m*/*z* 166 of lower relative intensity [9]. Although differences in instrumental conditions and data processing cannot be excluded, this discrepancy may also suggest co-elution or assignment of a closely related isomer in the earlier report. Therefore, the identity of this compound in the present *D. columnae* fraction was established only after synthesis of the synthetic reference compounds, comparison of RI values on both DB-5MS and HP-Innowax columns, EI-MS matching, and co-injection experiments.

Overall, comparison with literature data highlights the analytical value of synthetic reference compounds for hydroxythymol diester identification. The available literature provided important evidence for the natural occurrence of these compounds, but the observed RI and mass-spectral discrepancies demonstrate that reliable identification of hydroxythymol diesters in complex matrices requires combined use of synthesized references, dual-column RI data, EI mass spectra, and co-injection.

**Scheme 2:**
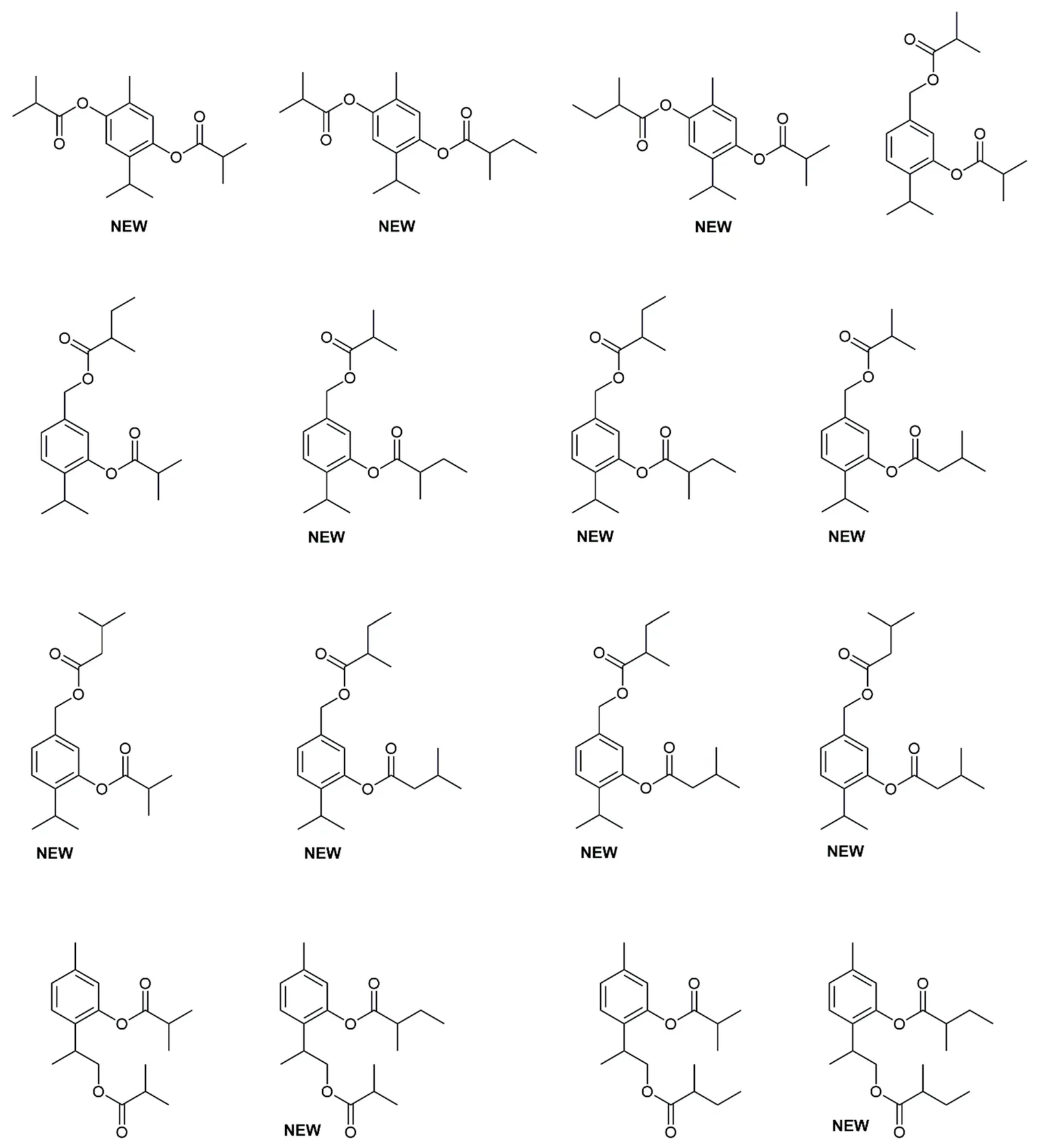
Structures of hydroxythymol diesters identified in the more polar fraction of the root essential oil of *D. columnae*. Compounds marked with **NEW** are reported here for the first time as natural occurrences.

Application of the synthetic reference library enabled the identification of 16 hydroxythymol diesters in the more polar fraction of *D. columnae* root essential oil, including 12 compounds reported here for the first time as natural constituents. The evidence supporting the individual assignments is summarized in Table 4, while the corresponding chemical structures are presented in Scheme 2. The identification criteria comprised comparison with the synthetic references by EI-MS, retention-index measurements, and co-injection. Representative co-injection chromatograms are presented in Figures S169–S171. In addition, the synthetic work provided access to a total of 49 diacylated thymol derivatives, 44 of which have not previously been described in the literature. These results demonstrate that the chemical diversity of *D. columnae* root essential oil extends beyond the previously reported methoxy-*p*-cymene derivatives and includes a structurally rich series of regioisomeric hydroxythymol diesters.

The assignments of 6-iPr-3-sBu and 6-sBu-3-iPr required particular attention because their synthetic references had closely similar retention indices on DB-5MS (2044 and 2045, respectively) and the same rounded index on HP-Innowax (2604). In the unspiked botanical fraction, two shoulders preceded the larger peak assigned to 7-sBu-3-iPr. Separate additions of the NMR-characterized synthetic references enhanced the earlier feature for 6-iPr-3-sBu and the later feature for 6-sBu-3-iPr (Figure S171). These responses support the individual assignments within a partially overlapping chromatographic region; baseline separation was not achieved. The very similar EI mass spectra and the identical rounded HP-Innowax index were treated as supporting information and not as independent means of distinguishing the two regioisomers.

The C-7 series spans all nine combinations of isobutyryl, 2-methylbutyryl and isovaleryl residues at the benzylic and phenolic oxygen atoms. Its greater diversity therefore refers to the number of identified structures, not their relative abundance. Together with the accompanying C-6 and C-9 derivatives and the thymyl esters and oxygenated methoxy-*p*-cymene derivatives reported previously [3], these findings expand the structural description of the investigated *D. columnae* root oil and provide reference information for future comparisons of related botanical materials.

Overall, the present study establishes a comprehensive GC–MS/RI reference dataset for hydroxythymol diesters and demonstrates its practical value in the analysis of complex natural matrices. The combination of synthesis, dual-column RI determination, EI-MS comparison, co-injection, and structure–retention analysis allowed reliable differentiation of closely related regioisomeric and acyl-homologous compounds that could not be confidently identified from literature data alone. The generated dataset therefore provides a useful analytical framework for future identification of thymol-derived esters in essential oils, herbal preparations, food-related botanical matrices, and other complex natural products.

Previous descriptions of the constitutional structures and previous reports of their occurrence in nature were assessed separately using structure-based searches in SciFinder (performed on 23 October 2026) and examination of the relevant phytochemical literature. In Table 4, “new natural occurrence” denotes a compound for which no earlier report from a natural source was located in these searches; it does not imply individual isolation from the botanical fraction in the present study.

## 3. Materials and Methods

### 3.1. General Experimental Procedures

All reagents and solvents were obtained from commercial sources (Sigma-Aldrich, St. Louis, MO, USA; Merck, Darmstadt, Germany; Fisher Scientific, Waltham, MA, USA) and used as received unless otherwise stated. Organic solvents were distilled prior to use when necessary. Column and dry-flash chromatography were performed on silica gel 60 (40–63 µm, Acros Organics, Geel, Belgium), while thin-layer chromatography (TLC) was carried out on pre-coated silica gel plates (Kieselgel 60 F254, 0.2 mm, Merck, Darmstadt, Germany). TLC spots were visualized under UV light (254 nm) and by spraying with 50% (*v*/*v*) aqueous H_2_SO_4_ followed by heating. Elemental analyses (C, H, O) were performed using a Carlo Erba Elemental Analyzer 1106 (Carlo Erba, Milan, Italy). High-resolution mass spectra (HRMS) were recorded on a JEOL JMS-700 mass spectrometer (JEOL, Tokyo, Japan) under electron ionization (EI) conditions. Infrared spectra (ATR-FTIR) were obtained using a Thermo Nicolet 6700 spectrometer (Thermo Nicolet, Waltham, MA, USA), while UV–Vis spectra were measured in acetonitrile on a Shimadzu UV-1800 spectrophotometer (Shimadzu, Tokyo, Japan).

#### 3.1.1. GC–MS Analysis and Retention Indices

GC–MS analyses were performed in triplicate using a Hewlett-Packard 6890N gas chromatograph coupled to a 5975B mass selective detector (Agilent Technologies, Palo Alto, CA, USA). Separation was achieved on a DB-5MS fused silica capillary column (30 m × 0.25 mm i.d., film thickness 0.25 µm; 5% polydiphenylsiloxane, 95% polydimethylsiloxane). The injector and transfer line temperatures were set at 250 °C and 300 °C, respectively. The oven temperature was programmed from 70 °C to 290 °C at a rate of 5 °C/min, followed by a 10 min isothermal hold. Helium was used as the carrier gas at a constant flow of 1.0 mL/min. Samples (1 mg/mL in diethyl ether) were injected (1.0 μL) in pulsed split mode (initial flow 1.5 mL/min for 0.5 min, then 1.0 mL/min; split ratio 40:1). Mass spectra were recorded under electron ionization (EI, 70 eV) in the *m*/*z* range 35–650.

For analyses on a polar stationary phase, an HP-Innowax capillary column (polyethylene glycol stationary phase, 30 m × 0.25 mm i.d., film thickness 0.25 µm; Agilent Technologies) was used. The injector and transfer line temperatures were set at 200 °C and 230 °C, respectively. The oven temperature was programmed from 60 °C (3 min) to 220 °C at 4 °C/min, followed by a 10 min hold. Helium was used as the carrier gas (1.0 mL/min), and injections were performed in splitless mode.

Linear retention indices (RI) were calculated relative to a homologous series of *n*-alkanes (C_8_–C_30_), analyzed under the same chromatographic conditions as the investigated compounds, according to the van den Dool and Kratz equation for temperature-programmed GC runs. RI values are reported as integer mean values from three independent injections. Compound identification was based on comparison of experimental RI values with literature data, matching of EI mass spectra with those of synthetic reference compounds, and comparison with spectra from commercial libraries (Wiley 11, NIST 17, MassFinder 2.3) and a laboratory-built MS library. Where available, co-injection with the corresponding synthetic reference was used to support the assignment. Co-injection results were interpreted in relation to the separation observed in the unspiked sample. For adequately separated components, an increase in the corresponding peak without a separate additional peak, together with spectral agreement with the reference, was considered supportive. For partially overlapping components, the response was evaluated by comparing features already discernible in the unspiked fraction with those enhanced by separate additions of the individual references. In these cases, the assignment relied on the correspondence between the pre-existing retention feature and the standard-addition response, considered together with the available RI and EI-MS evidence. Spectra acquired from overlapping regions were interpreted with allowance for contributions from neighboring constituents.

#### 3.1.2. NMR Spectroscopy

^1^H and ^13^C NMR spectra were recorded on a Bruker Avance III 400 MHz spectrometer (Bruker Corporation, Fällanden, Switzerland) operating at 400 and 100.6 MHz, respectively, using a 5 mm dual ^1^H/^13^C probe at 20 °C. Two-dimensional experiments (^1^H–^1^H COSY, NOESY, gHSQC, and gHMBC) as well as DEPT-90/135 experiments were performed using standard Bruker pulse sequences. Samples were dissolved in CDCl_3_ with tetramethylsilane (TMS) as an internal reference. Chemical shifts (δ) are reported in ppm relative to TMS (δ_H_ = 0.00 ppm) or the (residual) solvent signal (CHCl_3_ for ^1^H and CDCl_3_ for ^13^C) and coupling constants (*J*) are given in Hz. The following abbreviations were used to describe signal multiplicities: s, singlet; d, doublet; t, triplet; q, quartet; sept, septet; m, multiplet; br s, broad singlet; dd, doublet of doublets; ddd, doublet of doublet of doublets; dddd, doublet of doublet of doublet of doublets; dt, doublet of triplets; dq, doublet of quartets; ddt, doublet of doublet of triplets; dtd, doublet of triplet of doublets; dtt, doublet of triplet of triplets; ddq, doublet of doublets of quartets; dqd, doublet of quartet of doublets; dqdq, doublet of quartet of doublet of quartets; dqt, doublet of quartet of triplets; dtq, doublet of triplet of quartets; ddqd, doublet of doublet of quartet of doublets; ddtd, doublet of doublet of triplet of doublets; dddq, doublet of doublet of doublet of quartets; dddqt, doublet of doublet of doublet of quartet of triplets; dddtq, doublet of doublet of doublet of triplet of quartets; dqdddd, doublet of quartet of doublet of doublet of doublet of doublets; qd, quartet of doublets; qdd, quartet of doublet of doublets; qddq, quartet of doublet of doublets of quartets; tdq, triplet of doublet of quartets; tqt, triplet of quartet of triplets; ttq, triplet of triplet of quartets; tsept, triplet of septets; septd, septet of doublets; septdd, septet of doublet of doublets; septddd, septet of doublet of doublet of doublets; septt, septet of triplets. ^1^H NMR full spin analyses were performed by iterative manual adjustment of δ and *J* values to achieve optimal agreement with the experimental spectra, followed by refinement using MestReNova 11.0.3 software (Tools/Spin Simulation). This procedure enabled systematic alignment of calculated and experimental NMR parameters, with normalized root-mean-square deviation (NRMSD) values below 0.05% [19]. Chemical shifts in the main experimental descriptions are generally reported to two decimal places for ^1^H and one decimal place for ^13^C. Additional decimal places are retained where rounding would obscure distinct resonances. The Supplementary Materials preserves the ^1^H chemical-shift parameters obtained by iterative full spin analysis to four decimal places to support reproduction of the calculated spectra. The number of reported digits specifies numerical precision and should not be interpreted as an estimate of absolute chemical-shift accuracy. Additional precision in the tabulated ^13^C data is retained where needed to distinguish closely spaced resonances.

### 3.2. Essential Oil Fraction

The root essential oil of *Doronicum columnae* Ten. used in the present study was obtained during our previous investigation, as described in detail elsewhere [3]. Briefly, roots of *D. columnae* were collected from a wild population in the Šar Mountains, Republic of Serbia, during the flowering stage in May 2021. Voucher specimens were deposited in the Herbarium of the Faculty of Sciences and Mathematics, University of Niš, Serbia, under the acquisition number HMN-15655. The underground parts were separated, washed to remove soil, dried, and used for essential-oil isolation.

The essential oil was obtained from the dried roots by hydrodistillation using a Clevenger-type apparatus, giving a yellowish oil in 0.56% yield (*w*/*w*, based on dry plant material). During the previous study, the essential oil was subjected to dry-column vacuum chromatography on silica gel. The fraction relevant to the present work represented a combined more polar fraction obtained by pooling fractions 12–16, which were eluted with 10–20% (*v*/*v*) diethyl ether in *n*-hexane [10.0 mg (fr. 12), 8.3 mg (fr. 13), 7.0 mg (fr. 14), 4.9 mg (fr. 15), and 15.6 mg (fr. 16); 45.8 mg in total]. This combined fraction was enriched in minor thymol-derived constituents. In the present work, the available portion of this fraction was re-examined by GC–MS under the chromatographic conditions described in Section 3.1.1 in order to characterize additional hydroxythymol diesters that could not be assigned with high confidence in the original study.

The botanical application was restricted to a previously obtained, enriched fraction of root essential oil from one collection of *D. columnae*. The results concern the occurrence of the assigned constituents in the analyzed material; they do not establish their prevalence across populations, seasonal variability or relative abundance in the unfractionated essential oil.

### 3.3. Synthesis of 2-Acyloxythymyl Esters

#### 3.3.1. Formylation of Thymol

The formylation of thymol was performed according to a modified Reimer–Tiemann procedure [20]. A three-necked round-bottom flask equipped with a reflux condenser, thermometer, and septum was charged with a hot aqueous solution of sodium hydroxide (30 g in 30 mL of water). A solution of thymol (15 g, 0.1 mol) in water (15 mL) was then added, and the reaction mixture was stirred magnetically. The temperature of the mixture was maintained at 60–65 °C to prevent precipitation of sodium thymolate. Chloroform (23.8 g, 16 mL, 0.2 mol) was added dropwise through the septum, while maintaining the reaction temperature in the range of 65–70 °C. After complete addition, the reaction mixture was heated on a water bath for 1 h to ensure completion of the reaction. The mixture was then cooled to room temperature and acidified to pH 1 by addition of 1 M hydrochloric acid. The aqueous phase was extracted with diethyl ether (3 × 50 mL). The combined organic extracts were washed with saturated aqueous NaCl solution (2 × 50 mL) and dried with anhydrous MgSO_4_. After filtration, the solvent was removed under reduced pressure using a rotary evaporator to afford a crude residue (12 g). Purification was achieved by dry-flash chromatography on silica gel using diethyl ether/hexane (1:20, *v*/*v*) as the eluent. This procedure afforded 3 g (17%) of 2-formylthymol as reddish liquid. The purity of this product was confirmed by NMR, TLC and GC–MS analysis.

*2-Formylthymol (syn. 2-hydroxy-3-isopropyl-6-methylbenzaldehyde)*: reddish liquid; retention index (RI) = 1434 (DB-5MS column); MS (EI), *m*/*z* (%) 178 (35), 164 (11), 163 (100), 149 (9), 135 (7), 115 (16), 105 (6), 91 (22), 79 (6), 77 (11); analyzed C 74.10, H 7.94, calculated for C_11_H_14_O_2_, C 74.13, H 7.92, O 17.95%; ^1^H NMR (CDCl_3_) δ 1.22 (d, ^3^*J*_8,9/10_ = 6.9, 6 H, CH_3_-9 and CH_3_-10), 2.57 (dd, ^4^*J*_6,7_ = −0.8, ^5^*J*_5,7_ = 0.3, 3 H, CH_3_-7), 3.32 (septdd, ^3^*J*_8,9/10_ = 6.9, ^4^*J*_5,8_ = −0.5, ^5^*J*_6,8_ = 0.3, 1 H, CH-8), 6.68 (dqd, ^3^*J*_5,6_ = 7.7, ^4^*J*_6,7_ = −0.8, ^5^*J*_6,8_ = 0.3, 1 H, CH-6), 7.32 (ddq, ^3^*J*_5,6_ = 7.7, ^4^*J*_5,8_ = −0.5, ^5^*J*_5,7_ = 0.3, 1 H, CH-5), 10.30 (s, 1 H, CHO), 12.29 (brs, 1 H, OH); ^13^C NMR (CDCl_3_) δ 18.1 (C-7), 22.4 (C-9 and C-10), 26.1 (C-8), 118.1 (C-2), 121.5 (C-6), 134.1 (C-5), 135.5 (C-4), 139.4 (C-1), 161.0 (C-3), 195.8 (CHO).

#### 3.3.2. Dakin Oxidation of Formylated Thymol (Synthesis of 2-Hydroxythymol)

The synthesis of 2-hydroxythymol was achieved via Dakin oxidation of the corresponding formylated thymol [21]. A solution of formylated thymol (1.78 g, 0.01 mol) in 1 M aqueous NaOH (10 mL) was prepared in a 100 mL round-bottom flask at room temperature. Hydrogen peroxide (15 mL, 3% (*w*/*w*) aqueous solution) was added, and the reaction mixture was stirred at room temperature for 24 h. After completion of the reaction, the mixture was acidified to pH 1 by addition of 1 M hydrochloric acid and extracted with diethyl ether (3 × 30 mL). The combined organic layers were washed with saturated aqueous NaCl (2 × 30 mL) and dried with anhydrous MgSO_4_. The drying agent was removed by filtration, and the solvent was evaporated under reduced pressure to afford a crude residue (1.6 g). Purification was carried out by column chromatography on silica gel using diethyl ether/*n*-hexane (1:5, *v*/*v*) as the eluent, yielding 2-hydroxythymol (1.2 g, 72%). The purity of the product was confirmed by NMR, TLC and GC–MS analyses.

*2-Hydroxythymol (syn. 3-isopropyl-6-methylbenzene-1,2-diol)*: brown solid; RI = 1445 (DB-5MS column); FTIR (neat; cm^−1^) 3432, 2965, 2874, 1629, 1582, 1508, 1467, 1432, 1386, 1367, 1336, 1301, 1274, 1236, 1204, 1176, 1154, 1109, 1066, 1044, 961, 926, 864, 801, 755, 716, 676; MS (EI), *m*/*z* (%) 166 (34), 152 (10), 151 (100), 133 (14), 105 (23), 103 (6), 91 (7), 79 (8), 77 (13), 65 (5); analyzed C 72.28, H 8.46, calculated for C_10_H_14_O_2_, C 72.26, H 8.49, O 19.25%; ^1^H NMR (CDCl_3_) δ 1.24 (d, ^3^*J*_8,9/10_ = 6.9, 6 H, CH_3_-9 and CH_3_-10), 2.23 (dd, ^4^*J*_6,7_ = −0.7, ^5^*J*_5,7_ = 0.3, 3 H, CH_3_-7), 3.15 (septdd, ^3^*J*_8,9/10_ = 6.9, ^4^*J*_5,8_ = −0.5, ^5^*J*_6,8_ = 0.3, 1 H, CH-8), 5.02 (brs, 1 H, C2-OH), 5.25 (brs, 1 H, C3-OH), 6.67 (dqd, ^3^*J*_5,6_ = 8.0, ^4^*J*_6,7_ = −0.7, ^5^*J*_6,8_ = 0.3, 1 H, CH-6), 6.70 (ddq, ^3^*J*_5,6_ = 8.0, ^4^*J*_5,8_ = −0.5, ^5^*J*_5,7_ = 0.3, 1 H, CH-5); ^13^C NMR (CDCl_3_) δ 15.5 (C-7), 22.8 (C-9 and C-10), 27.3 (C-8), 117.5 (C-5), 121.3 (C-1), 122.0 (C-6), 132.6 (C-4), 141.1 (C-3), 141.6 (C-2).

#### 3.3.3. Esterification of 2-Hydroxythymol with Corresponding Carboxylic Acids

Esters of 2-hydroxythymol with isobutyric, 2-methylbutyric, and isovaleric acids were synthesized according to a standard Steglich esterification protocol using *N*,*N*′-dicyclohexylcarbodiimide (DCC) and 4-(dimethylamino)pyridine (DMAP). A solution of 2-hydroxythymol (200 mg, 1.2 mmol), the corresponding carboxylic acid (1.5 mmol), DMAP (5 mg, 0.04 mmol), and DCC (309 mg, 1.5 mmol) in dry CH_2_Cl_2_ (50 mL) was stirred at room temperature overnight. The precipitated *N*,*N*′-dicyclohexylurea was removed by filtration, and the filtrate was concentrated under reduced pressure. The crude product was purified by column chromatography on silica gel using *n*-hexane/Et_2_O (19:1, *v*/*v*, followed by 4:1, *v*/*v*) as the eluent. 2-Isobutyryloxythymyl isobutyrate was obtained in 15% yield (54 mg), while an inseparable mixture of 2-hydroxythymyl isobutyrate and 2-isobutyryloxythymol was obtained in 69% yield (195 mg). 2-(2-Methylbutyryloxy)thymyl 2-methylbutyrate was obtained in 11% yield (42 mg), while an inseparable mixture of 2-hydroxythymyl 2-methylbutyrate and 2-(2-methylbutyryloxy)thymol was obtained in 66% yield (198 mg). 2-Isovaleroxythymyl isovalerate was obtained in 13% yield (53 mg), while an inseparable mixture of 2-hydroxythymyl isovalerate and 2-isovaleroxythymol was obtained in 68% yield (204 mg). The purity of all prepared esters was confirmed by NMR, TLC and GC–MS analyses.

*2-Isobutyryloxythymyl isobutyrate (syn. 3-isopropyl-6-methyl-1,2-phenylene diisobutyrate, **2-iPr-3-iPr**)*: colorless liquid; RI = 1902 (DB-5MS column); UV (CH_3_CN) λ_max_(log ε) 261 (2.51), 210 (4.06), 193 (4.60) nm; FTIR (neat; cm^−1^) 2972, 2935, 2876, 1762, 1497, 1460, 1428, 1387, 1366, 1339, 1269, 1234, 1176, 1108, 1091, 1050, 964, 945, 903, 861, 847, 813, 747, 697; MS (EI), *m*/*z* (%) 306 (6), 236 (10), 167 (10), 166 (87), 151 (13), 77 (5), 71 (98), 70 (6), 43 (100), 41 (12); HRMS, *m*/*z* 306.1828 [M]^+^, calculated for C_18_H_26_O_4_^•+^, 306.1826; analyzed C 70.58, H 8.52, calculated for C_18_H_26_O_4_, C 70.56, H 8.55, O 20.89%; ^1^H NMR (CDCl_3_) δ 1.18 (d, ^3^*J*_8,9/10_ = 6.9, 6 H, CH_3_-9 and CH_3_-10), 1.31 (d, ^3^*J*_2″,3″_ = 7.0, 6 H, CH_3_-3″), 1.32 (d, ^3^*J*_2′,3′_ = 7.0, 6 H, CH_3_-3′), 2.12 (dd, ^4^*J*_6,7_ = −0.7, ^5^*J*_5,7_ = 0.3, 3 H, CH_3_-7), 2.80 (sept, ^3^*J*_2″,3″_ = 7.0, 1 H, CH-2″), 2.82 (sept, ^3^*J*_2′,3′_ = 7.0, 1 H, CH-2′), 2.92 (septdd, ^3^*J*_8,9/10_ = 6.9, ^4^*J*_5,8_ = −0.5, ^5^*J*_6,8_ = 0.3, 1 H, CH-8), 7.07 (dqd, ^3^*J*_5,6_ = 8.1, ^4^*J*_6,7_ = −0.7, ^5^*J*_6,8_ = 0.3, 1 H, CH-6), 7.08 (ddq, ^3^*J*_5,6_ = 8.1, ^4^*J*_5,8_ = −0.5, ^5^*J*_5,7_ = 0.3, 1 H, CH-5); ^13^C NMR (CDCl_3_) δ 16.1 (C-7), 19.16 (C-3′), 19.22 (C-3″), 23.1 (C-9 and C-10), 27.4 (C-8), 34.13^a^ (C-2″), 34.14^a^ (C-2′), 123.4 (C-5), 128.2 (C-6), 129.2 (C-1), 139.6 (C-4), 140.3 (C-3), 141.3 (C-2), 174.3 (C-1″), 174.6 (C-1′). ^a^ The signals of C-2′ and C-2″ may be interchanged.

*2-Hydroxythymyl isobutyrate (syn. 2-hydroxy-6-isopropyl-3-methylphenyl isobutyrate) and 2-isobutyryloxythymol (syn. 2-hydroxy-3-isopropyl-6-methylphenyl isobutyrate)*: yellowish liquid; RI = 1642 (DB-5MS column); MS (EI), *m*/*z* (%) 236 (9), 166 (33), 151 (38), 91 (6), 77 (9), 71 (49), 44 (13), 43 (100), 42 (8), 41 (22); analyzed C 71.14, H 8.55, calculated for C_14_H_20_O_3_, C 71.16, H 8.53, O 20.31%; ^1^H NMR (CDCl_3_) (2-hydroxythymyl isobutyrate) δ 1.18 (d, ^3^*J*_8,9/10_ = 6.9, 6 H, CH_3_-9 and CH_3_-10), 1.3717 (d, ^3^*J*_2′,3′_ = 7, 6 H, CH_3_-3′), 2.22 (dd, ^4^*J*_6,7_ = −0.7, ^5^*J*_5,7_ = 0.3, 3 H, CH_3_-7), 2.9122 (sept, ^3^*J*_2′,3′_ = 7.0, 1 H, CH-2′), 2.93 (septdd, ^3^*J*_8,9/10_ = 6.9, ^4^*J*_5,8_ = −0.5, ^5^*J*_6,8_ = 0.3, 1 H, CH-8), 4.90 (brs, 1 H, OH), 6.7829 (ddq, ^3^*J*_5,6_ = 8.0, ^4^*J*_5,8_ = −0.5, ^5^*J*_5,7_ = 0.3, 1 H, CH-5), 6.98 (dqd, ^3^*J*_5,6_ = 8.0, ^4^*J*_6,7_ = −0.7, ^5^*J*_6,8_ = 0.3, 1 H, CH-6); ^13^C NMR (CDCl_3_) δ 15.7 (C-7), 19.25 (C-3′), 23.1 (C-9 and C-10), 27.5 (C-8), 34.40 (C-2′), 117.7 (C-5), 123.8 (C-1), 128.10 (C-6), 136.6 (C-3), 138.9 (C-4), 145.4 (C-2), 175.7 (C-1′); ^1^H NMR (CDCl_3_) (2-isobutyryloxythymol) δ 1.23 (d, ^3^*J*_8,9/10_ = 6.9, 6 H, CH_3_-9 and CH_3_-10), 1.3706 (d, ^3^*J*_2′,3′_ = 7.0, 6 H, CH_3_-3′), 2.12 (dd, ^4^*J*_6,7_ = −0.7, ^5^*J*_5,7_ = 0.3, 3 H, CH_3_-7), 2.9084 (sept, ^3^*J*_2′,3′_ = 7.0, 1 H, CH-2′), 3.23 (septdd, ^3^*J*_8,9/10_ = 6.9, ^4^*J*_5,8_ = −0.5, ^5^*J*_6,8_ = 0.3, 1 H, CH-8), 5.03 (brs, 1 H, OH), 6.7517 (dqd, ^3^*J*_5,6_ = 8.0, ^4^*J*_6,7_ = −0.7, ^5^*J*_6,8_ = 0.3, 1 H, CH-6), 6.9765 (ddq, ^3^*J*_5,6_ = 8.0, ^4^*J*_5,8_ = −0.5, ^5^*J*_5,7_ = 0.3, 1 H, CH-5); ^13^C NMR (CDCl_3_) δ 16.3 (C-7), 19.31 (C-3′), 22.7 (C-9 and C-10), 27.2 (C-8), 34.39 (C-2′), 122.3 (C-6), 123.4 (C-5), 128.13 (C-1), 134.6 (C-4), 137.4 (C-2), 144.6 (C-3), 175.2 (C-1′).

*2-(2-Methylbutyryloxy)thymyl 2-methylbutyrate (syn. 3-isopropyl-6-methyl-1,2-phenylene bis(2-methylbutyrate, **2-sBu-3-sBu**)*: colorless liquid; RI = 2089 (DB-5MS column); MS (EI), *m*/*z* (%) 334 (6), 250 (14), 167 (7), 166 (61), 151 (9), 85 (71), 84 (4), 58 (4), 57 (100), 41 (10).

*2-Hydroxythymyl 2-methylbutyrate (syn. 2-hydroxy-6-isopropyl-3-methylphenyl 2-methylbutyrate) and 2-(2-methylbutyryloxy)thymol (syn. 2-hydroxy-3-isopropyl-6-methylphenyl 2-methylbutyrate)*: yellowish solid; RI = 1735 (DB-5MS column); FTIR (neat; cm^−1^) 3433, 2965, 2933, 2873, 1728, 1579, 1497, 1460, 1382, 1362, 1347, 1301, 1266, 1227, 1176, 1146, 1113, 1094, 1035, 1010, 969, 928, 902, 868, 845, 800, 749, 718, 680; MS (EI), *m*/*z* (%) 250 (5), 166 (19), 151 (23), 91 (4), 85 (26), 77 (6), 58 (5), 57 (100), 55 (5), 41 (15); analyzed C 71.99, H 8.82, calculated for C_15_H_22_O_3_, C 71.97, H 8.86, O 19.17%; ^1^H NMR (CDCl_3_) (2-hydroxythymyl 2-methylbutyrate) δ 1.0436 (ddd, ^3^*J*_3′A,4′_ = 7.5, ^3^*J*_3′B,4′_ = 7.5, ^4^*J*_2′,4′_ = 0.3, 3 H, CH_3_-4′), 1.18 (d, ^3^*J*_8,9/10_ = 6.9, 6 H, CH_3_-9 and CH_3_-10), 1.3428 (d, ^3^*J*_2′,5′_ = 7.0, 3 H, CH_3_-5′), 1.6398 (dqd, ^2^*J*_3′A,3′B_ = −13.7, ^3^*J*_3′B,4′_ = 7.5, ^3^*J*_2′,3′B_ = 6.5, 1 H, CH-3′B), 1.8919 (ddq, ^2^*J*_3′A,3′B_ = −13.7, ^3^*J*_2′,3′A_ = 7.5, ^3^*J*_3′A,4′_ = 7.5, 1 H, CH-3′A), 2.15 (dd, ^4^*J*_6,7_ = −0.6, ^5^*J*_5,7_ = 0.3, 3 H, CH_3_-7), 2.7144 (dqdq, ^3^*J*_2′,3′A_ = 7.5, ^3^*J*_2′,5′_ = 7.0, ^3^*J*_2′,3′B_ = 6.5, ^4^*J*_2′,4′_ = 0.3, 1 H, CH-2′), 2.94 (septdd, ^3^*J*_8,9/10_ = 6.9, ^4^*J*_5,8_ = −0.5, ^5^*J*_6,8_ = 0.3, 1 H, CH-8), 5.05 (brs, 1 H, OH), 6.77 (ddq, ^3^*J*_5,6_ = 8.0, ^4^*J*_5,8_ = −0.5, ^5^*J*_5,7_ = 0.3, 1 H, CH-5), 6.95 (dqd, ^3^*J*_5,6_ = 8.0, ^4^*J*_6,7_ = −0.6, ^5^*J*_6,8_ = 0.3, 1 H, CH-6); ^13^C NMR (CDCl_3_) δ 11.8 (C-4′), 15.6 (C-7), 16.96 (C-5′), 23.11/23.13 (C-9 and 10), 26.89 (C-3′), 27.4 (C-8), 41.48 (C-2′), 117.6 (C-5), 123.9 (C-1), 128.05 (C-6), 136.6 (C-3), 138.9 (C-4), 145.4 (C-2), 175.4 (C-1′); ^1^H NMR (CDCl_3_) (2-(2-methylbutyryloxy)thymol) δ 1.0398 (ddd, ^3^*J*_3′A,4′_ = 7.5, ^3^*J*_3′B,4′_ = 7.5, ^4^*J*_2′,4′_ = 0.3, 3 H, CH_3_-4′), 1.22 (d, ^3^*J*_8,9/10_ = 6.9, 6 H, CH_3_-9 and CH_3_-10), 1.3428 (d, ^3^*J*_2′,5′_ = 7.0, 3 H, CH_3_-5′), 1.6398 (dqd, ^2^*J*_3′A,3′B_ = −13.7, ^3^*J*_3′B,4′_ = 7.5, ^3^*J*_2′,3′B_ = 6.5, 1 H, CH-3′B), 1.8919 (ddq, ^2^*J*_3′A,3′B_ = −13.7, ^3^*J*_2′,3′A_ = 7.5, ^3^*J*_3′A,4′_ = 7.5, 1 H, CH-3′A), 2.12 (dd, ^4^*J*_6,7_ = −0.6, ^5^*J*_5,7_ = 0.3, 3 H, CH_3_-7), 2.7144 (dqdq, ^3^*J*_2′,3′A_ = 7.5, ^3^*J*_2′,5′_ = 7.0, ^3^*J*_2′,3′B_ = 6.5, ^4^*J*_2′,4′_ = 0.3, 1 H, CH-2′), 3.21 (septdd, ^3^*J*_8,9/10_ = 6.9, ^4^*J*_5,8_ = −0.5, ^5^*J*_6,8_ = 0.3, 1 H, CH-8), 5.09 (brs, 1 H, OH), 6.75 (dqd, ^3^*J*_5,6_ = 8.0, ^4^*J*_6,7_ = −0.6, ^5^*J*_6,8_ = 0.3, 1 H, CH-6), 6.97 (ddq, ^3^*J*_5,6_ = 8.0, ^4^*J*_5,8_ = −0.5, ^5^*J*_5,7_ = 0.3, 1 H, CH-5); ^13^C NMR (CDCl_3_) δ 11.9 (C-4′), 16.3 (C-7), 17.02 (C-5′), 22.72/22.73 (C-9 and C-10), 26.89 (C-3′), 27.2 (C-8), 41.44 (C-2′), 122.3 (C-6), 123.3 (C-5), 128.13 (C-1), 134.6 (C-4), 137.5 (C-2), 144.6 (C-3), 174.8 (C-1′).

*2-Isovaleroxythymyl isovalerate (syn. 3-isopropyl-6-methyl-1,2-phenylene diisovalerate, **2-iBu-3-iBu**)*: colorless liquid; RI = 2055 (DB-5MS column); MS (EI), *m*/*z* (%) 250 (7), 167 (12), 166 (100), 151 (11), 85 (71), 69 (7), 57 (84), 55 (6), 43 (17), 41 (34).

*2-Hydroxythymyl isovalerate (syn. 2-hydroxy-6-isopropyl-3-methylphenyl isovalerate) and 2-isovaleroxythymol (syn. 2-hydroxy-3-isopropyl-6-methylphenyl isovalerate)*: white solid; RI = 1740 (DB-5MS column); FTIR (neat; cm^−1^) 3371, 2958, 2871, 1726, 1620, 1506, 1462, 1429, 1389, 1362, 1337, 1293, 1275, 1258, 1212, 1191, 1171, 1148, 1095, 1069, 1061, 972, 941, 911, 851, 805, 775, 745, 719; MS (EI), *m*/*z* (%) 250 (10), 167 (10), 166 (88), 151 (73), 91 (9), 85 (59), 77 (13), 57 (100), 43 (18), 41 (35); analyzed C 71.96, H 8.88, calculated for C_15_H_22_O_3_, C 71.97, H 8.86, O 19.17%; ^1^H NMR (CDCl_3_) (2-hydroxythymyl isovalerate) 1.0758 (d, ^3^*J*_3′,4′_ = 6.7, 6 H, CH_3_-4′), 1.18 (d, ^3^*J*_8,9/10_ = 6.9, 6 H, CH_3_-9 and CH_3_-10), 2.21 (dd, ^4^*J*_6,7_ = −0.7, ^5^*J*_5,7_ = 0.3, 3 H, CH_3_-7), 2.2777 (tsept, ^3^*J*_2′,3′_ = 7.2, ^3^*J*_3′,4′_ = 6.7, 1 H, CH-3′), 2.5240 (d, ^3^*J*_2′,3′_ = 7.2, 2 H, CH-2′), 2.93 (septdd, ^3^*J*_8,9/10_ = 6.9, ^4^*J*_5,8_ = −0.5, ^5^*J*_6,8_ = 0.3, 1 H, CH-8), 4.99 (brs, 1 H, OH), 6.78 (ddq, ^3^*J*_5,6_ = 8.0, ^4^*J*_5,8_ = −0.5, ^5^*J*_5,7_ = 0.3, 1 H, CH-5), 6.9740 (dqd, ^3^*J*_5,6_ = 8.0, ^4^*J*_6,7_ = −0.7, ^5^*J*_6,8_ = 0.3, 1 H, CH-6); ^13^C NMR (CDCl_3_) δ 15.7 (C-7), 22.60 (C-4′), 23.2 (C-9 and C-10), 25.94 (C-3′), 27.7 (C-8), 43.25 (C-2′), 117.7 (C-5), 123.8 (C-1), 128.18 (C-6), 136.6 (C-3), 139.0 (C-4), 145.4 (C-2), 171.7 (C-1′); ^1^H NMR (CDCl_3_) (2-isovaleroxythymol) δ 1.0758 (d, ^3^*J*_3′,4′_ = 6.7, 6 H, CH_3_-4′), 1.22 (d, ^3^*J*_8,9/10_ = 6.9, 6 H, CH_3_-9 and CH_3_-10), 2.13 (dd, ^4^*J*_6,7_ = −0.7, ^5^*J*_5,7_ = 0.3, 3 H, CH_3_-7), 2.2729 (tsept, ^3^*J*_2′,3′_ = 7.2, ^3^*J*_3′,4′_ = 6.7, 1 H, CH-3′), 2.5163 (d, ^3^*J*_2′,3′_ = 7.2, 2 H, CH-2′), 3.23 (septdd, ^3^*J*_8,9/10_ = 6.9, ^4^*J*_5,8_ = −0.5, ^5^*J*_6,8_ = 0.3, 1 H, CH-8), 5.06 (brs, 1 H, OH), 6.75 (dqd, ^3^*J*_5,6_ = 8.0, ^4^*J*_6,7_ = −0.7, ^5^*J*_6,8_ = 0.3, 1 H, CH-6), 6.9764 (ddq, ^3^*J*_5,6_ = 8.0, ^4^*J*_5,8_ = −0.5, ^5^*J*_5,7_ = 0.3, 1 H, CH-5); ^13^C NMR (CDCl_3_) δ 16.4 (C-7), 22.62 (C-4′), 22.7 (C-9 and C-10), 25.96 (C-3′), 27.2 (C-8), 43.17 (C-2′), 122.3 (C-6), 123.4 (C-5), 128.15 (C-1), 134.5 (C-4), 137.5 (C-2), 144.6 (C-3), 171.2 (C-1′).

Mixed diesters of 2-hydroxythymol monoesters with isobutyric, 2-methylbutyric, and isovaleric acids were synthesized following a standard Steglich esterification protocol as described above.

*2-Isobutyryloxythymyl 2-methylbutyrate and 2-(2-methylbutyryloxy)thymyl isobutyrate:* These compounds were prepared from a mixture of 2-hydroxythymyl 2-methylbutyrate and 2-(2-methylbutyryloxy)thymol (125 mg, 0.5 mmol), isobutyric acid (44 mg, 0.5 mmol), DCC (103 mg, 0.5 mmol), and DMAP (5 mg, 0.04 mmol) in 20 mL of dry CH_2_Cl_2_. Column chromatographic purification on silica gel using *n*-hexane/diethyl ether (19:1, *v*/*v*) afforded 128 mg (80%) of a mixture of 2-isobutyryloxythymyl 2-methylbutyrate and 2-(2-methylbutyryloxy)thymyl isobutyrate.

*2-Isobutyryloxythymyl 2-methylbutyrate (syn. 2-isobutyryloxy-6-isopropyl-3-methylphenyl 2-methylbutyrate, **2-iPr-3-sBu**):* colorless liquid; RI = 2004 (DB-5MS column); FTIR (neat; cm^−1^) 2969, 2937, 2877, 1761, 1497, 1459, 1428, 1386, 1364, 1339, 1267, 1222, 1175, 1107, 1090, 966, 942, 891, 857, 813, 746; MS (EI), *m*/*z* (%) 236 (9), 167 (9), 166 (78), 151 (12), 91 (6), 85 (49), 71 (47), 57 (100), 43 (51), 41 (19); HRMS, *m*/*z* 320.1980 [M]^+^, calculated for C_19_H_28_O_4_^•+^, 320.1982; analyzed C 71.21, H 8.83, calculated for C_19_H_28_O_4_, C 71.22, H 8.81, O 19.97%; ^1^H NMR (CDCl_3_) δ 1.0153 (ddd, ^3^*J*_3″A,4″_ = 7.5, ^3^*J*_3″B,4″_ = 7.5, ^4^*J*_2″,4″_ = 0.3, 3 H, CH_3_-4″), 1.1811 (d, ^3^*J*_8,9/10_ = 6.9, 6 H, CH_3_-9 and CH_3_-10), 1.2940 (d, ^3^*J*_2″,5″_ = 7.0, 3 H, CH_3_-5″), 1.3218/1.3245 (d, ^3^*J*_2′,3′_ = 7.0, 6 H, CH_3_-3′), 1.5830 (dqd, ^2^*J*_3″A,3″B_ = −13.7, ^3^*J*_3″B,4″_ = 7.5, ^3^*J*_2″,3″B_ = 6.9, 1 H, CH-3″B), 1.8636 (dqd, ^2^*J*_3″A,3″B_ = −13.7, ^3^*J*_3″A,4″_ = 7.5, ^3^*J*_2″,3″A_ = 6.9, 1 H, CH-3″A), 2.13 (dd, ^4^*J*_6,7_ = −0.7, ^5^*J*_5,7_ = 0.3, 3 H, CH_3_-7), 2.6164 (qddq, ^3^*J*_2″,5″_ = 7.0, ^3^*J*_2″,3″A_ = 6.9, ^3^*J*_2″,3″B_ = 6.9, ^4^*J*_2″,4″_ = 0.3, 1 H, CH-2″), 2.8156 (sept, ^3^*J*_2′,3′_ = 7.0, 1 H, CH-2′), 2.9227 (septdd, ^3^*J*_8,9/10_ = 6.9, ^4^*J*_5,8_ = −0.5, ^5^*J*_6,8_ = 0.3, 1 H, CH-8), 7.0720 (dqd, ^3^*J*_5,6_ = 8.1, ^4^*J*_6,7_ = −0.7, ^5^*J*_6,8_ = 0.3, 1 H, CH-6), 7.0841 (ddq, ^3^*J*_5,6_ = 8.1, ^4^*J*_5,8_ = −0.5, ^5^*J*_5,7_ = 0.3, 1 H, CH-5); ^13^C NMR (CDCl_3_) δ 11.85 (C-4″), 16.3 (C-7), 16.7 (C-5″), 19.14/19.17 (C-3′), 23.08/23.11 (C-9 and C-10), 26.7 (C-3″), 27.31 (C-8), 34.09 (C-2′), 41.05 (C-2″), 123.34 (C-5), 128.24 (C-6), 129.2 (C-1), 139.6 (C-4), 140.28 (C-3), 141.34 (C-2), 173.9 (C-1″), 174.7 (C-1′).

*2-(2-Methylbutyryloxy)thymyl isobutyrate (syn. 2-isobutyryloxy-3-isopropyl-6-methylphenyl 2-methylbutyrate, **2-sBu-3-iPr**):* colorless liquid; RI = 1997 (DB-5MS column); FTIR (neat; cm^−1^) 2969, 2937, 2877, 1761, 1497, 1459, 1428, 1386, 1364, 1339, 1267, 1222, 1175, 1107, 1090, 966, 942, 891, 857, 813, 746; MS (EI), *m*/*z* (%) 236 (5), 167 (5), 166 (45), 151 (7), 85 (42), 71 (43), 57 (100), 55 (4), 43 (57), 41 (23); HRMS, *m*/*z* 320.1980 [M]^+^, calculated for C_19_H_28_O_4_^•+^, 320.1982; analyzed C 71.21, H 8.83, calculated for C_19_H_28_O_4_, C 71.22, H 8.81, O 19.97%; ^1^H NMR (CDCl_3_) δ 1.0220 (ddd, ^3^*J*_3′A,4′_ = 7.5, ^3^*J*_3′B,4′_ = 7.5, ^4^*J*_2′,4′_ = 0.3, 3 H, CH_3_-4′), 1.1811 (d, ^3^*J*_8,9/10_ = 6.9, 6 H, CH_3_-9 and CH_3_-10), 1.3029 (d, ^3^*J*_2′,5′_ = 7.0, 3 H, CH_3_-5′), 1.3123/1.3153 (d, ^3^*J*_2″,3″_ = 7.0, 6 H, CH_3_-3″), 1.5893 (dqd, ^2^*J*_3′A,3′B_ = −13.7, ^3^*J*_3′B,4′_ = 7.5, ^3^*J*_2′,3′B_ = 7.0, 1 H, CH-3′B), 1.8750 (dqd, ^2^*J*_3′A,3′B_ = −13.7, ^3^*J*_3′A,4′_ = 7.5, ^3^*J*_2′,3′A_ = 6.6, 1 H, CH-3′A), 2.12 (dd, ^4^*J*_6,7_ = −0.7, ^5^*J*_5,7_ = 0.3, 3 H, CH_3_-7), 2.6294 (dqdq, ^3^*J*_2′,3′B_ = 7.0, ^3^*J*_2′,5′_ = 7.0, ^3^*J*_2′,3′A_ = 6.6, ^4^*J*_2′,4′_ = 0.3, 1 H, CH-2′), 2.8007 (sept, ^3^*J*_2″,3″_ = 7.0, 1 H, CH-2″), 2.9285 (septdd, ^3^*J*_8,9/10_ = 6.9, ^4^*J*_5,8_ = −0.5, ^5^*J*_6,8_ = 0.3, 1 H, CH-8), 7.0720 (dqd, ^3^*J*_5,6_ = 8.1, ^4^*J*_6,7_ = −0.7, ^5^*J*_6,8_ = 0.3, 1 H, CH-6), 7.0870 (ddq, ^3^*J*_5,6_ = 8.1, ^4^*J*_5,8_ = −0.5, ^5^*J*_5,7_ = 0.3, 1 H, CH-5); ^13^C NMR (CDCl_3_) δ 11.83 (C-4′), 16.1 (C-7), 16.6 (C-5′), 19.21/19.25 (C-3″), 23.14/23.18 (C-9 and C-10), 26.6 (C-3′), 27.27 (C-8), 34.09 (C-2″), 41.07 (C-2′), 123.34 (C-5), 128.24 (C-6), 129.1 (C-1), 139.7 (C-4), 140.28 (C-3), 141.27 (C-2), 174.4 (C-1′), 174.2 (C-1″).

*2-Isobutyryloxythymyl isovalerate and 2-isovaleroxythymyl isobutyrate:* These compounds were prepared from a mixture of 2-hydroxythymyl isobutyrate and 2-isobutyryloxythymol (118 mg, 0.5 mmol), isovaleric acid (51 mg, 0.5 mmol), DCC (103 mg, 0.5 mmol), and DMAP (5 mg, 0.04 mmol) in 20 mL of dry CH_2_Cl_2_. Column chromatographic purification on silica gel using n-hexane/diethyl ether (19:1, *v*/*v*) afforded 135 mg (84%) of a mixture of 2-isobutyryloxythymyl isovalerate and 2-isovaleroxythymyl isobutyrate.

*2-Isobutyryloxythymyl isovalerate (syn. 2-isobutyryloxy-6-isopropyl-3-methylphenyl isovalerate, **2-iPr-3-iBu**):* colorless liquid; RI = 1980 (DB-5MS column); FTIR (neat; cm^−1^) 2964, 2875, 1763, 1497, 1461, 1428, 1387, 1365, 1337, 1284, 1268, 1237, 1176, 1148, 1093, 1050, 974, 942, 903, 855, 814, 782, 748; MS (EI), *m*/*z* (%) 236 (10), 167 (11), 166 (100), 151 (13), 85 (38), 77 (6), 71 (74), 57 (52), 43 (85), 41 (28); HRMS, *m*/*z* 320.1985 [M]^+^, calculated for C_19_H_28_O_4_^•+^, 320.1982; analyzed C 71.24, H 8.82, calculated for C_19_H_28_O_4_, C 71.22, H 8.81, O 19.97%; ^1^H NMR (CDCl_3_) δ 1.0480 (d, ^3^*J*_3″,4″_ = 6.7, 6 H, CH_3_-4″), 1.1815 (d, ^3^*J*_8,9/10_ = 6.9, 6 H, CH_3_-9 and CH_3_-10), 1.3281 (d, ^3^*J*_2′,3′_ = 7.0, 6 H, CH_3_-3′), 2.14 (dd, ^4^*J*_6,7_ = −0.6, ^5^*J*_5,7_ = 0.3, 3 H, CH_3_-7), 2.2246 (tsept, ^3^*J*_2″,3″_ = 7.2, ^3^*J*_3″,4″_ = 6.7, 1 H, CH-3″), 2.4205 (d, ^3^*J*_2″,3″_ = 7.2, 2 H, CH_2_-2″), 2.8198 (sept, ^3^*J*_2′,3′_ = 7.0, 1 H, CH-2′), 2.9242 (septdd, ^3^*J*_8,9/10_ = 6.9, ^4^*J*_5,8_ = −0.5, ^5^*J*_6,8_ = 0.3, 1 H, CH-8), 7.0756 (dqd, ^3^*J*_5,6_ = 8.2, ^4^*J*_6,7_ = −0.6, ^5^*J*_6,8_ = 0.3, 1 H, CH-6), 7.0880 (ddq, ^3^*J*_5,6_ = 8.2, ^4^*J*_5,8_ = −0.5, ^5^*J*_5,7_ = 0.3, 1 H, CH-5); ^13^C NMR (CDCl_3_) δ 16.3 (C-7), 19.2 (C-3′), 22.64 (C-4″), 23.1 (C-9 and C-10), 25.70 (C-3″), 27.38 (C-8), 34.18 (C-2′), 42.9 (C-2″), 123.44 (C-5), 128.2 (C-6), 129.22 (C-1), 139.6 (C-4), 140.3 (C-3), 141.3 (C-2), 170.2 (C-1″), 174.7 (C-1′).

*2-Isovaleroxythymyl isobutyrate (syn. 2-isobutyryloxy-3-isopropyl-6-methylphenyl isovalerate, **2-iBu-3-iPr**):* colorless liquid; RI = 1975 (DB-5MS column); FTIR (neat; cm^−1^) 2969, 2937, 2877, 1761, 1497, 1459, 1428, 1386, 1364, 1339, 1267, 1222, 1175, 1107, 1090, 966, 942, 891, 857, 813, 746; MS (EI), *m*/*z* (%) 236 (9), 167 (10), 166 (93), 151 (11), 85 (41), 71 (81), 57 (58), 44 (6), 43 (100), 41 (36); HRMS, *m*/*z* 320.1985 [M]^+^, calculated for C_19_H_28_O_4_^•+^, 320.1982; analyzed C 71.21, H 8.83, calculated for C_19_H_28_O_4_, C 71.22, H 8.81, O 19.97%; ^1^H NMR (CDCl_3_) δ 1.0531 (d, ^3^*J*_3′,4′_ = 6.7, 6 H, CH_3_-4′), 1.1841 (d, ^3^*J*_8,9/10_ = 6.9, 6 H, CH_3_-9 and CH_3_-10), 1.3192 (d, ^3^*J*_2″,3″_ = 7.0, 6 H, CH_3_-3″), 2.12 (dd, ^4^*J*_6,7_ = −0.6, ^5^*J*_5,7_ = 0.3, 3 H, CH_3_-7), 2.2305 (tsept, ^3^*J*_2′,3′_ = 7.2, ^3^*J*_3′,4′_ = 6.7, 1 H, CH-3′), 2.4374 (d, ^3^*J*_2′,3′_ = 7.2, 2 H, CH_2_-2′), 2.8066 (sept, ^3^*J*_2″,3″_ = 7.0, 1 H, CH-2″), 2.9310 (septdd, ^3^*J*_8,9/10_ = 6.9, ^4^*J*_5,8_ = −0.5, ^5^*J*_6,8_ = 0.3, 1 H, CH-8), 7.0756 (dqd, ^3^*J*_5,6_ = 8.2, ^4^*J*_6,7_ = −0.6, ^5^*J*_6,8_ = 0.3, 1 H, CH-6), 7.0880 (ddq, ^3^*J*_5,6_ = 8.2, ^4^*J*_5,8_ = −0.5, ^5^*J*_5,7_ = 0.3, 1 H, CH-5); ^13^C NMR (CDCl_3_) δ 16.1 (C-7), 19.3 (C-3″), 22.64 (C-4′), 23.2 (C-9 and C-10), 25.70 (C-3′), 27.42 (C-8), 34.16 (C-2″), 43.0 (C-2′), 123.42 (C-5), 128.3 (C-6), 129.17 (C-1), 139.7 (C-4), 140.2 (C-3), 141.2 (C-2), 170.7 (C-1′), 174.3 (C-1″).

*2-(2-Methylbutyryloxy)thymyl isovalerate and 2-isovaleroxythymyl 2-methylbutyrate:* These compounds were prepared from a mixture of 2-hydroxythymyl isovalerate and 2-isovaleroxythymol (125 mg, 0.5 mmol), 2-methylbutyric acid (51 mg, 0.5 mmol), DCC (103 mg, 0.5 mmol), and DMAP (5 mg, 0.04 mmol) in 20 mL of dry CH_2_Cl_2_. Column chromatographic purification on silica gel using n-hexane/diethyl ether (19:1, *v*/*v*) afforded 130 mg (78%) of a mixture of 2-isobutyryloxythymyl isovalerate and 2-isovaleroxythymyl isobutyrate.

*2-(2-Methylbutyryloxy)thymyl isovalerate (syn. 6-isopropyl-3-methyl-2-(2-methylbutyryloxy)phenyl isovalerate, **2-sBu-3-iBu**) and 2-isovaleroxythymyl 2-methylbutyrate (6-isopropyl-3-methyl-2-isovaleroxyphenyl 2-methylbutyrate, **2-iBu-3-sBu**):* colorless liquid; RI = 2070 (DB-5MS column); FTIR (neat; cm^−1^) 2964, 2875, 1762, 1497, 1459, 1427, 1363, 1337, 1283, 1265, 1221, 1147, 1096, 1040, 1008, 971, 939, 890, 860, 814, 747; MS (EI), *m*/*z* (%) 250 (7), 167 (7), 166 (59), 151 (8), 85 (67), 58 (4), 57 (100), 55 (4), 43 (5), 41 (18); HRMS, *m*/*z* 334.2135 [M]^+^, calculated for C_20_H_30_O_4_^•+^, 334.2139; analyzed C 71.80, H 9.07, calculated for C_20_H_30_O_4_, C 71.82, H 9.04, O 19.13%; ^1^H NMR (CDCl_3_) (*2-(2-methylbutyryloxy)thymyl isovalerate*) δ 1.0291 (ddd, ^3^*J*_3′A,4′_ = 7.4, ^3^*J*_3′B,4′_ = 7.4, ^4^*J*_2′,4′_ = 0.3, 3 H, CH_3_-4′), 1.0462 (d, ^3^*J*_3″,4″_ = 6.7, 6 H, CH_3_-4″), 1.1814 (d, ^3^*J*_8,9/10_ = 6.9, 6 H, CH_3_-9 and CH_3_-10), 1.3114 (d, ^3^*J*_2′,5′_ = 7.0, 3 H, CH_3_-5′), 1.5904 (dqd, ^2^*J*_3′A,3′B_ = −13.7, ^3^*J*_3′B,4′_ = 7.4, ^3^*J*_2′,3′B_ = 6.6, 1 H, CH-3′B), 1.8709 (dqd, ^2^*J*_3′A,3′B_ = −13.7, ^3^*J*_3′A,4′_ = 7.4, ^3^*J*_2′,3′A_ = 7.2, 1 H, CH-3′A), 2.14 (dd, ^4^*J*_6,7_ = −0.7, ^5^*J*_5,7_ = 0.3, 3 H, CH_3_-7), 2.2256 (tsept, ^3^*J*_2″,3″_ = 7.1, ^3^*J*_3″,4″_ = 6.7, 1 H, CH-3″), 2.4210 (d, ^3^*J*_2″,3″_ = 7.1, 2 H, CH_2_-2″), 2.6316 (dqdq, ^3^*J*_2′,3′A_ = 7.2, ^3^*J*_2′,5′_ = 7.0, ^3^*J*_2′,3′B_ = 6.6, ^4^*J*_2′,4′_ = 0.3, 1 H, CH-2′), 2.9303 (septdd, ^3^*J*_8,9/10_ = 6.9, ^4^*J*_5,8_ = −0.5, ^5^*J*_6,8_ = 0.3, 1 H, CH-8), 7.0770 (dqd, ^3^*J*_5,6_ = 8.1, ^4^*J*_6,7_ = −0.7, ^5^*J*_6,8_ = 0.3, 1 H, CH-6), 7.0891 (ddq, ^3^*J*_5,6_ = 8.1, ^4^*J*_5,8_ = −0.5, ^5^*J*_5,7_ = 0.3, 1 H, CH-5); ^13^C NMR (CDCl_3_) δ 11.92 (C-4′), 16.4 (C-7), 16.87 (C-5′), 22.66 (C-4″), 23.15/23.18 (C-9 and C-10), 25.66 (C-3″), 26.69 (C-3′), 27.3 (C-8), 41.19 (C-2′), 42.9 (C-2″), 123.43 (C-5), 128.2 (C-6), 129.22 (C-1), 139.67 (C-4), 140.3 (C-3), 141.28 (C-2), 170.3 (C-1″), 174.3 (C-1′); ^1^H NMR (CDCl_3_) (*2-isovaleroxythymyl 2-methylbutyrate*) δ 1.0219 (ddd, ^3^*J*_3″A,4″_ = 7.4, ^3^*J*_3″B,4″_ = 7.4, ^4^*J*_2″,4″_ = 0.3, 3 H, CH_3_-4″), 1.0515 (d, ^3^*J*_3′,4′_ = 6.7, 6 H, CH_3_-4′), 1.1832 (d, ^3^*J*_8,9/10_ = 6.9, 6 H, CH_3_-9 and CH_3_-10), 1.3021 (d, ^3^*J*_2″,5″_ = 7.0, 3 H, CH_3_-5″), 1.5845 (dqd, ^2^*J*_3″A,3″B_ = −13.7, ^3^*J*_3″B,4″_ = 7.4, ^3^*J*_2″,3″B_ = 6.6, 1 H, CH-3″B), 1.8604 (dqd, ^2^*J*_3″A,3″B_ = −13.7, ^3^*J*_3″A,4″_ = 7.4, ^3^*J*_2″,3″A_ = 7.2, 1 H, CH-3″A), 2.13 (dd, ^4^*J*_6,7_ = −0.7, ^5^*J*_5,7_ = 0.3, 3 H, CH_3_-7), 2.2309 (tsept, ^3^*J*_2′,3′_ = 7.1, ^3^*J*_3′,4′_ = 6.7, 1 H, CH-3′), 2.4377 (d, ^3^*J*_2′,3′_ = 7.1, 2 H, CH_2_-2′), 2.6205 (dqdq, ^3^*J*_2″,3″A_ = 7.2, ^3^*J*_2″,5″_ = 7.0, ^3^*J*_2″,3″B_ = 6.6, ^4^*J*_2″,4″_ = 0.3, 1 H, CH-2″), 2.9265 (septdd, ^3^*J*_8,9/10_ = 6.9, ^4^*J*_5,8_ = −0.5, ^5^*J*_6,8_ = 0.3, 1 H, CH-8), 7.0770 (dqd, ^3^*J*_5,6_ = 8.1, ^4^*J*_6,7_ = −0.7, ^5^*J*_6,8_ = 0.3, 1 H, CH-6), 7.0891 (ddq, ^3^*J*_5,6_ = 8.1, ^4^*J*_5,8_ = −0.5, ^5^*J*_5,7_ = 0.3, 1 H, CH-5); ^13^C NMR (CDCl_3_) δ 11.94 (C-4″), 16.3 (C-7), 16.94 (C-5″), 22.66 (C-4′), 23.24/23.25 (C-9 and C-10), 25.66 (C-3′), 26.74 (C-3″), 27.4 (C-8), 41.17 (C-2″), 43.0 (C-2′), 123.43 (C-5), 128.3 (C-6), 129.22 (C-1), 139.67 (C-4), 140.2 (C-3), 141.28 (C-2), 170.7 (C-1′), 173.9 (C-1″).

### 3.4. Synthesis of 5-Acyloxythymyl Esters

#### 3.4.1. Acetylation of Orcinol

A mixture of orcinol (2.0 g, 16 mmol), acetic anhydride (10 g, 98 mmol), and *p*-toluenesulfonic acid (0.2 g, 1.2 mmol) was refluxed for 30 min [3]. After completion of the reaction, the mixture was cooled to room temperature, and the excess acetic anhydride was decomposed by addition of 10% (*w*/*w*) aqueous NaOH solution. The reaction mixture was extracted with diethyl ether (3 × 30 mL). The combined organic layers were washed with saturated aqueous NaCl solution and dried with anhydrous MgSO_4_. The drying agent was removed by filtration, and the solvent was evaporated under reduced pressure to afford orcinol diacetate as a colorless liquid (3.03 g, 90%).

*Orcinol diacetate*: colorless liquid; RI = 1545 (DB-5MS column); MS (EI), *m*/*z* (%) 208 (5), 166 (15), 125 (7), 124 (100), 123 (11), 95 (6), 77 (4), 67 (4), 65 (3), 43 (35).

#### 3.4.2. Fries Rearrangement of Orcinol Diacetate

A mixture of orcinol diacetate (1 g, 5 mmol) and AlCl_3_ (2.2 g, 17 mmol) was stirred at 150 °C for 1.5 h [22]. After cooling to room temperature, the reaction mixture was poured onto ice containing 10% (*w*/*w*) aqueous HCl and stirred for 30 min. The mixture was extracted with ethyl acetate, and the combined organic extracts were dried with anhydrous MgSO_4_. After filtration, the solvent was removed under reduced pressure to afford crude 2,6-diacetyl-5-methylresorcinol. The crude product was then stirred in 85% (*w*/*w*) H_2_SO_4_ at room temperature for 2 h. The reaction mixture was poured onto ice, stirred for 30 min, and extracted with ethyl acetate. The combined organic layers were dried over anhydrous MgSO_4_, filtered, and concentrated under reduced pressure. Purification by dry-flash chromatography on silica gel using diethyl ether/hexane (1:5, *v*/*v*) as the eluent afforded 2-acetyl-5-methylresorcinol as a yellow solid (480 mg, 60%).

*2-Acetyl-5-resorcinol (syn. 1-(2,6-dihydroxy-4-methylphenyl)ethan-1-one):* yellow solid; RI = 1640 (DB-5MS column); MS (EI), *m*/*z* (%) 166 (35), 152 (8), 151 (100), 77 (6), 65 (7), 55 (9), 53 (10), 51 (8), 43 (18), 41 (7).

#### 3.4.3. Synthesis of 5-Hydroxydehydrothymol

5-Hydroxythymol was synthesized via a Grignard reaction of 2-acetyl-5-methylresorcinol with methylmagnesium iodide, ref. [23] followed by spontaneous dehydration of the initially formed tertiary alcohol. Magnesium turnings (600 mg, 25 mmol) and a crystal of iodine were suspended in anhydrous diethyl ether (20 mL), and a solution of methyl iodide (3.6 g, 25 mmol) in anhydrous diethyl ether (20 mL) was added dropwise, maintaining gentle reflux. After complete addition, the reaction mixture was refluxed for an additional 30 min. The mixture was then cooled to 0 °C, and a solution of 2-acetyl-5-methylresorcinol (332 mg, 2 mmol) in diethyl ether (10 mL) was added dropwise over 15 min. The reaction mixture was subsequently refluxed for 30 min. After cooling to 0 °C, saturated aqueous NH_4_Cl solution was added dropwise to quench the reaction. The mixture was extracted with diethyl ether (3 × 20 mL), and the combined organic layers were dried with anhydrous MgSO_4_. After filtration, the solvent was removed under reduced pressure to afford the 300 mg of 5-hydroxydehydrothymol as pale brown liquid.

*5-Hydroxydehydrothymol (syn. 5-methyl-2-(prop-1-en-2-yl)benzene-1,3-diol):* pale brown liquid; RI = 1430 (DB-5MS column); MS (EI), *m*/*z* (%) 164 (100), 149 (57), 121 (60), 93 (27), 91 (44), 77 (36), 67 (18), 65 (16), 53 (22), 51 (22).

#### 3.4.4. Synthesis of 5-Hydroxythymol

A solution of 5-hydroxydehydrothymol (200 mg, 1.3 mmol) and 5% Pd/C (100 mg) in anhydrous ethyl acetate (50 mL) was stirred under an atmosphere of hydrogen (1 atm) at room temperature for 24 h. After completion, the mixture was filtered to remove the catalyst, and the solvent was removed under reduced pressure. Purification by dry-flash chromatography on silica gel using diethyl ether/hexane (1:5, *v*/*v*) as the eluent to afford 5-hydroxythymol (190 mg, 94%).

*5-Hydroxythymol (syn. 2-isopropyl-5-methylbenzene-1,3-diol):* pale brown solid; RI = 1539 (DB-5MS column); FTIR (neat; cm^−1^) 3484, 3395, 2965, 2877, 1622, 1586, 1527, 1420, 1379, 1348, 1326, 1272, 1235, 1204, 1162, 1134, 1111, 1065, 1027, 988, 974, 930, 816, 752, 701; MS (EI), *m*/*z* (%) 166 (29), 152 (10), 151 (100), 105 (7), 91 (6), 79 (10), 77 (13), 53 (7), 43 (6), 41 (6); analyzed C 72.27, H 8.50, calculated for C_10_H_14_O_2_, C 72.26, H 8.49, O 19.25%; ^1^H NMR (CDCl_3_) δ 1.35 (d, ^3^*J*_8,9/10_ = 7.1, 6 H, CH_3_-9 and CH_3_-10), 2.18 (t, ^4^*J*_2/6,7_ = −0.7, 3 H, CH_3_-7), 3.41 (septt, ^3^*J*_8,9/10_ = 7.1, ^5^*J*_2/6,8_ = 0.3, 1 H, CH-8), 4.90 (brs, 2 H, OH), 6.16 (qd, ^4^*J*_2/6,7_ = −0.7, ^5^*J*_2/6,8_ = 0.3, 2 H, CH-2 and CH-6); ^13^C NMR (CDCl_3_) δ 20.9 (C-7), 21.0 (C-9 and C-10), 24.5 (C-8), 109.5 (C-2 and C-6), 117.6 (C-4), 136.9 (C-1), 154.8 (C-3 and C-5).

#### 3.4.5. Esterification of 5-Hydroxythymol with Corresponding Carboxylic Acids

Mono- and diesters of 5-hydroxythymol with isobutyric, 2-methylbutyric, and isovaleric acids were synthesized following a standard Steglich esterification protocol as described above in Section 3.3.3.

*5-Hydroxythymyl isobutyrate and 5-isobutyryloxythymyl isobutyrate:* These were prepared only for GC–MS analysis from 5-hydroxythymol (5 mg, 0.03 mmol), isobutyric acid (4 mg, 0.05 mmol), DCC (10 mg, 0.05 mmol), and DMAP (1.3 mg, 0.01 mmol) in 1 mL of dry CH_2_Cl_2_.

*5-Hydroxythymyl isobutyrate (3-hydroxy-2-isopropyl-5-methylphenyl isobutyrate):* RI = 1747 (DB-5MS column); MS (EI), *m*/*z* (%) 236 (6), 166 (32), 152 (4), 151 (46), 91 (4), 71 (36), 53 (6), 44 (5), 43 (100), 41 (15).

*5-Isobutyryloxythymyl isobutyrate (syn. 2-isopropyl-5-methyl-1,3-phenylene diisobutyrate, **5-iPr-3-iPr**):* RI = 1933 (DB-5MS column); MS (EI), *m*/*z* (%) 306 (5), 236 (17), 167 (11), 166 (99), 165 (9), 151 (37), 71 (52), 44 (5), 43 (100), 41 (16).

*5-Hydroxythymyl 2-methylbutyrate and 5-(2-methylbutyryloxy)thymyl 2-methylbutyrate:* These were prepared only for GC–MS analysis from 5-hydroxythymol (5 mg, 0.03 mmol), 2-methylbutyric acid (5 mg, 0.05 mmol), DCC (10 mg, 0.05 mmol), and DMAP (1.3 mg, 0.01 mmol) in 1 mL of dry CH_2_Cl_2_.

*5-Hydroxythymyl 2-methylbutyrate (3-hydroxy-2-isopropyl-5-methylphenyl 2-methylbutyrate):* RI = 1846 (DB-5MS column); MS (EI), *m*/*z* (%) 250 (4), 166 (21), 151 (29), 85 (21), 58 (4), 57 (100), 55 (6), 53 (6), 43 (9), 41 (19).

*5-(2-Methylbutyryloxy)thymyl 2-methylbutyrate (syn. 2-isopropyl-5-methyl-1,3-phenylene bis(2-methylbutyrate), **5-sBu-3-sBu**):* RI = 2121 (DB-5MS column); MS (EI), *m*/*z* (%) 334 (4), 250 (18), 167 (11), 166 (100), 165 (8), 151 (25), 85 (31), 58 (4), 57 (92), 41 (14).

*5-Hydroxythymyl isovalerate and 5-isovaleroxythymyl isovalerate:* These were prepared from 5-hydroxythymol (50 mg, 0.3 mmol), isovaleric acid (41 mg, 0.4 mmol), DCC (83 mg, 0.4 mmol), and DMAP (1.3 mg, 0.01 mmol) in 10 mL of CH_2_Cl_2_. Column chromatographic purification with *n*-hexane/diethyl ether (19:1, and then 4:1, *v*/*v*) yielded 5-isovaleroxythymyl isovalerate 25 mg (25%) and 5-hydroxythymyl isovalerate 47 mg (63%).

*5-Hydroxythymyl isovalerate (syn. 3-hydroxy-2-isopropyl-5-methylphenyl isovalerate):* colorless liquid; RI = 1848 (DB-5MS column); MS (EI), *m*/*z* (%) 250 (8), 166 (45), 165 (6), 152 (10), 151 (100), 85 (10), 77 (6), 57 (27), 43 (7), 41 (12); analyzed C 71.98, H 8.85, calculated for C_15_H_22_O_3_, C 71.97, H 8.86, O 19.17%; ^1^H NMR (CDCl_3_) δ 1.06 (d, ^3^*J*_3′,4′_ = 6.6, 6 H, CH_3_-4′), 1.28 (d, ^3^*J*_8,9/10_ = 7.1, 6 H, CH_3_-9 and CH_3_-10), 2.23 (dd, ^4^*J*_2,7_ = −0.7, ^4^*J*_6,7_ = −0.7, 3 H, CH_3_-7), 2.26 (tsept, ^3^*J*_2′,3′_ = 7.2, ^3^*J*_3′,4′_ = 6.6, 1 H, CH-3′), 2.45 (d, ^3^*J*_2′,3′_ = 7.2, 2 H, CH-2′), 3.15 (septdd, ^3^*J*_8,9/10_ = 7.1, ^5^*J*_2,8_ = 0.3, ^5^*J*_6,8_ = 0.3, 1 H, CH-8), 4.86 (brs, 1 H, OH), 6.38 (dqd, ^4^*J*_2,6_ = 1.7, ^4^*J*_2,7_ = −0.7, ^5^*J*_2,8_ = 0.3, 1 H, CH-2), 6.41 (dqd, ^4^*J*_2,6_ = 1.7, ^4^*J*_6,7_ = −0.7, ^5^*J*_6,8_ = 0.3, 1 H, CH-6); ^13^C NMR (CDCl_3_) δ 20.86 (C-9 and C-10), 20.94 (C-7), 22.6 (C-4′), 25.5 (C-8), 25.9 (C-3′), 43.5 (C-2′), 114.7 (C-6), 115.9 (C-2), 123.5 (C-4), 136.9 (C-1), 149.5 (C-3), 154.9 (C-5), 172.0 (C-1′).

*5-Isovaleroxythymyl isovalerate (syn. 2-isopropyl-5-methyl-1,3-phenylene diisovalerate, **5-iBu-3-iBu**):* colorless liquid; RI = 2126 (DB-5MS column); UV (CH_3_CN) λ_max_(log ε) 264 (2.89), 196 (4.42) nm; FTIR (neat; cm^−1^) 2962, 2874, 1759, 1626, 1575, 1467, 1416, 1364, 1289, 1242, 1177, 1149, 1120, 1084, 1024, 984, 910, 883, 843, 774, 720; MS (EI), *m*/*z* (%) 334 (3), 250 (14), 167 (12), 166 (100), 165 (7), 152 (3), 151 (34), 85 (14), 57 (25), 41 (7); HRMS, *m*/*z* 334.2142 [M]^+^, calculated for C_20_H_30_O_4_^•+^, 334.2139; analyzed C 71.81, H 9.05, calculated for C_20_H_30_O_4_, C 71.82, H 9.04, O 19.13%; ^1^H NMR (CDCl_3_) 1.06 (d, ^3^*J*_3′/3″,4′/4″_ = 6.7, 12 H, CH_3_-4′ and CH_3_-4″), 1.21 (d, ^3^*J*_8,9/10_ = 7.1, 6 H, CH_3_-9 and CH_3_-10), 2.26 (tsept, ^3^*J*_2′/2″,3′/3″_ = 7.2, ^3^*J*_3′/3″,4′/4″_ = 6.7, 2 H, CH-3′), 2.29 (t, ^4^*J*_2/6,7_ = −0.7, 3 H, CH_3_-7), 2.45 (d, ^3^*J*_2′/2″,3′/3″_ = 7.2, 4 H, CH-2′), 3.04 (septt, ^3^*J*_8,9/10_ = 7.1, ^5^*J*_2/6,8_ = 0.3, 1 H, CH-8), 6.70 (qd, ^4^*J*_2/6,7_ = −0.7, ^5^*J*_2/6,8_ = 0.3, 2 H, CH-2 and CH-6); ^13^C NMR (CDCl_3_) δ 21.0 (C-7), 21.2 (C-9 and C-10), 22.6 (C-4′ and C-4″), 25.80 (C-3′ and C-3″), 25.82 (C-8), 43.5 (C-2′ and C-2″), 121.4 (C-2 and C-6), 128.9 (C-4), 136.8 (C-1), 149.4 (C-3 and C-5), 171.6 (C-1′ and C-1″).

Mixed diesters of 5-hydroxythymol with isobutyric, 2-methylbutyric, and isovaleric acids were synthesized following a standard Steglich esterification protocol as described above in Section 3.3.3. These diesters were synthesized only for GC–MS analysis.

*5-Isobutyryloxythymyl 2-methylbutyrate (syn. 3-isobutyryloxy-2-isopropyl-5-methylphenyl 2-methylbutyrate, **5-iPr-3-sBu**):* It was prepared only for GC–MS analysis from 5-hydroxythymol (5 mg, 0.03 mmol), isobutyric acid (2.6 mg, 0.03 mmol), 2-methylbutyric acid (3 mg, 0.03 mmol), DCC (10 mg, 0.05 mmol), and DMAP (1.3 mg, 0.01 mmol) in 1 mL of dry CH_2_Cl_2_.

*5-Isobutyryloxythymyl isovalerate (syn. 3-isobutyryloxy-2-isopropyl-5-methylphenyl isovalerate, **5-iPr-3-iBu**):* It was prepared only for GC–MS analysis from 5-hydroxythymyl isovalerate (7.5 mg, 0.03 mmol), isobutyric acid (2.6 mg, 0.03 mmol), DCC (10 mg, 0.05 mmol), and DMAP (1.3 mg, 0.01 mmol) in 1 mL of dry CH_2_Cl_2_; RI = 2028 (DB-5MS column); MS (EI), *m*/*z* (%) 236 (13), 167 (11), 166 (100), 165 (8), 151 (35), 85 (14), 71 (18), 57 (24), 43 (36), 41 (13).

*5-(2-Methylbutyryloxy)thymyl isovalerate (syn. 3-(2-methylbutyryloxy)-2-isopropyl-5-methylphenyl isovalerate, **5-sBu-3-iBu**):* It was prepared only for GC–MS analysis from 5-hydroxythymyl isovalerate (7.5 mg, 0.03 mmol), 2-methylbutyric acid (3 mg, 0.03 mmol), DCC (10 mg, 0.05 mmol), and DMAP (1.3 mg, 0.01 mmol) in 1 mL of dry CH_2_Cl_2_; RI = 2123 (DB-5MS column); MS (EI), *m*/*z* (%) 250 (10), 167 (13), 166 (87), 165 (10), 151 (25), 85 (34), 57 (100), 55 (9), 43 (15), 41 (33).

### 3.5. Synthesis of 6-Acyloxythymyl Esters

#### Esterification of 6-Hydroxythymol (Thymohydroquinone) with Corresponding Carboxylic Acids

Thymohydroquinone was prepared from thymol according to a procedure analogous to that previously reported for the synthesis of related methoxy-*p*-cymene derivatives [3]. Mono- and diesters of thymohydroquinone with isobutyric, 2-methylbutyric, and isovaleric acids were synthesized following a standard Steglich esterification protocol as described above in Section 3.3.3.

*6-Hydroxythymyl isobutyrate, 6-isobutyryloxythymol and 6-isobutyryloxythymyl isobutyrate:* These were prepared from thymohydroquinone (200 mg, 1.2 mmol), isobutyric acid (132 mg, 1.5 mmol), DCC (309 mg, 1.5 mmol), and DMAP (5 mg, 0.04 mmol) in 50 mL of CH_2_Cl_2_. Column chromatographic purification with *n*-hexane/diethyl ether (19:1, and then 4:1, *v*/*v*) yielded 6-isobutyryloxythymyl isobutyrate 50 mg (13%), 6-hydroxythymyl isobutyrate 46 mg (16%), and 6-isobutyryloxythymol 73 mg (26%).

*6-Hydroxythymyl isobutyrate (4-hydroxy-2-isopropyl-5-methylphenyl isobutyrate):* The obtained RI, MS, UV, FTIR and NMR spectral data were in full agreement with the literature values reported by Dimitrijevic et al. [3].

*6-Isobutyryloxythymol (syn. 4-hydroxy-5-isopropyl-2-methylphenyl isobutyrate):* The obtained RI, MS, UV, FTIR and NMR spectral data were in full agreement with the literature values reported by Dimitrijevic et al. [3].

*6-Isobutyryloxythymyl isobutyrate (syn. 2-isopropyl-5-methyl-1,4-phenylene diisobutyrate, **6-iPr-3-iPr**):* white solid; RI = 1952 (DB-5MS column); UV (CH_3_CN) λ_max_(log ε) 273 (3.01), 268 (3.00), 193 (4.63) nm; FTIR (neat; cm^−1^) 2967, 2934, 2876, 2357, 1750, 1502, 1459, 1400, 1380, 1365, 1350, 1303, 1278, 1240, 1156, 1121, 1101, 1047, 1002, 928, 893, 842, 816, 754, 668, 618; MS (EI), *m*/*z* (%) 306 (2), 236 (8), 167 (11), 166 (100), 151 (12), 137 (2), 77 (2), 71 (12), 43 (24), 41 (5); analyzed C 70.53, H 8.57, O 20.90%, HRMS, *m*/*z* 306.1823 [M]^+^, calculated for C_18_H_26_O_4_^•+^, 306.1826; calculated for C_18_H_26_O_4_, C 70.56, H 8.55, O 20.89%; ^1^H NMR (CDCl_3_) δ 1.18 (d, ^3^*J*_8,9/10_ = 6.9, 6 H, CH_3_-9 and CH_3_-10), 1.33 (d, ^3^*J*_2″,3″_ = 7.0, 6 H, CH_3_-3″), 1.34 (d, ^3^*J*_2′,3′_ = 7.0, 6 H, CH_3_-3′), 2.11 (dd, ^4^*J*_2,7_ = −0.7, ^5^*J*_5,7_ = 0.3, 3 H, CH_3_-7), 2.8253 (sept, ^3^*J*_2″,3″_ = 7.0, 1 H, CH-2″), 2.8301 (sept, ^3^*J*_2′,3′_ = 7.0, 1 H, CH-2′), 2.95 (septdd, ^3^*J*_8,9/10_ = 6.9, ^4^*J*_5,8_ = −0.6, ^5^*J*_2,8_ = 0.3, 1 H, CH-8), 6.84 (qdd, ^4^*J*_2,7_ = −0.7, ^5^*J*_2,5_ = 0.3, ^5^*J*_2,8_ = 0.3, 1 H, CH-2), 6.90 (ddq, ^4^*J*_5,8_ = −0.6, ^5^*J*_2,5_ = 0.3, ^5^*J*_5,7_ = 0.3, 1 H, CH-5); ^13^C NMR (CDCl_3_) δ 16.0 (C-7), 19.1 (C-3″), 19.2 (C-3′), 22.9 (C-9 and C-10), 27.4 (C-8), 34.3 (C-2′), 34.4 (C-2″), 119.9 (C-5), 124.5 (C-2), 128.5 (C-1), 139.0 (C-4), 145.5 (C-3), 147.2 (C-6), 175.3 (C-1′), 175.8 (C-1″).

*6-Hydroxythymyl 2-methylbutyrate, 6-(2-methylbutyryloxy)thymol and 6-(2-methylbutyryoxy)thymyl 2-methylbutyrate:* These were prepared from thymohydroquinone (200 mg, 1.2 mmol), 2-methylbutyric acid (153 mg, 1.5 mmol), DCC (309 mg, 1.5 mmol), and DMAP (5 mg, 0.04 mmol) in 50 mL of CH_2_Cl_2_. Column chromatographic purification with *n*-hexane/diethyl ether (19:1, and then 4:1, *v*/*v*) yielded 6-(2-methylbutyryoxy)thymyl 2-methylbutyrate 52 mg (13%), 6-hydroxythymyl 2-methylbutyrate 45 mg (15%), and 6-(2-methylbutyryloxy)thymol 70 mg (23%).

*6-Hydroxythymyl 2-methylbutyrate (4-hydroxy-2-isopropyl-5-methylphenyl 2-methylbutyrate):* The obtained RI, MS, UV, FTIR and NMR spectral data were in full agreement with the literature values reported by Dimitrijevic et al. [3].

*6-(2-Methylbutyryloxy)thymol (syn. 4-Hydroxy-5-isopropyl-2-methylphenyl 2-methylbutyrate):* The obtained RI, MS, UV, FTIR and NMR spectral data were in full agreement with the literature values reported by Dimitrijevic et al. [3].

*6-(2-Methylbutyryoxy)thymyl 2-methylbutyrate (syn. 2-isopropyl-5-methyl-1,4-phenylene bis(2-methylbutyrate), **6-sBu-3-sBu**):* yellowish liquid; RI = 2142 (DB-5MS column); UV (CH_3_CN) λ_max_(log ε) 273 (3.06), 267 (3.06), 193 (4.74) nm; FTIR (neat; cm^−1^) 2965, 2934, 2877, 1752, 1500, 1459, 1401, 1381, 1362, 1342, 1260, 1219, 1152, 1108, 1087, 1059, 1008, 965, 910, 800, 743; MS (EI), *m*/*z* (%) 334 (2), 250 (9), 167 (11), 166 (100), 151 (10), 137 (3), 85 (10), 57 (30), 55 (2), 41 (8); HRMS, *m*/*z* 334.2136 [M]^+^, calculated for C_20_H_30_O_4_^•+^, 334.2139; analyzed C 71.80, H 9.05, calculated for C_20_H_30_O_4_, C 71.82, H 9.04, O 19.13%; ^1^H NMR (CDCl_3_) δ 1.03 (ddd, ^3^*J*_3″A,4″_ = 7.5, ^3^*J*_3″B,4″_ = 7.5, ^4^*J*_2″,4″_ = 0.3, 3 H, CH_3_-4″), 1.04 (ddd, ^3^*J*_3′A,4′_ = 7.5, ^3^*J*_3′B,4′_ = 7.5, ^4^*J*_2′,4′_ = 0.3, 3 H, CH_3_-4′), 1.18 (d, ^3^*J*_8,9/10_ = 6.9, 6 H, CH_3_-9 and CH_3-_10), 1.31 (d, ^3^*J*_2″,5″_ = 7.0, 3 H, CH_3_-5″), 1.32 (d, ^3^*J*_2′,5′_ = 7.0, 3 H, CH_3_-5′), 1.6196 (dqd, ^2^*J*_3″A,3″B_ = −13.7, ^3^*J*_3″B,4″_ = 7.5, ^3^*J*_2″,3″B_ = 6.5, 1 H, CH-3″B), 1.6230 (dqd, ^2^*J*_3′A,3′B_ = −13.7, ^3^*J*_3′B,4′_ = 7.5, ^3^*J*_2′,3′B_ = 6.5, 1 H, CH-3′B), 1.86 (ddq, ^2^*J*_3″A,3″B_ = −13.7, ^3^*J*_2″,3″A_ = 7.5, ^3^*J*_3″A,4″_ = 7.5, 1 H, CH-3″A), 1.87 (ddq, ^2^*J*_3′A,3′B_ = −13.7, ^3^*J*_2′,3′A_ = 7.5, ^3^*J*_3′A,4′_ = 7.5, 1 H, CH-3′A), 2.12 (dd, ^4^*J*_2,7_ = −0.7, ^5^*J*_5,7_ = 0.3, 3 H, CH_3_-7), 2.6394 (dqdq, ^3^*J*_2″,3″A_ = 7.5, ^3^*J*_2″,5″_ = 7.0, ^3^*J*_2″,3″B_ = 6.5, ^4^*J*_2″,4″_ = 0.3, 1 H, CH-2″), 2.6429 (dqdq, ^3^*J*_2′,3′A_ = 7.5, ^3^*J*_2′,5′_ = 7.0, ^3^*J*_2′,3′B_ = 6.5, ^4^*J*_2′,4′_ = 0.3, 1 H, CH-2′), 2.97 (septd, ^3^*J*_8,9/10_ = 6.9, ^4^*J*_5,8_ = −0.6, 1 H, CH-8), 6.83 (qd, ^4^*J*_2,7_ = −0.7, ^5^*J*_2,5_ = 0.3 1 H, CH-2), 6.89 (ddq, ^4^*J*_5,8_ = −0.6, ^5^*J*_2,5_ = 0.3, ^5^*J*_5,7_ = 0.3, 1 H, CH-5); ^13^C NMR (CDCl_3_) δ 11.85^a^ (C-4″), 11.88^a^ (C-4′), 16.1 (C-7), 16.83^b^ (C-5″), 16.86^b^ (C-5′), 23.0 (C-9 and C-10), 26.85^c^ (C-3″), 26.86^c^ (C-3′), 27.3 (C-8), 41.3 (C-2′), 41.4 (C-2″), 119.9 (C-5), 124.5 (C-2), 128.5 (C-1), 139.0 (C-4), 145.5 (C-3), 147.2 (C-6), 174.9 (C-1′), 175.4 (C-1″). ^a^ The signals of C-4′ and C-4″ may be interchanged; ^b^ the signals of C-5′ and C-5″ may be interchanged; ^c^ the signals of C-3′ and C-3″ may be interchanged.

*6-Isovaleroxythymyl isovalerate (syn. 2-isopropyl-5-methyl-1,4-phenylene diisovalerate, **6-iBu-3-iBu**):* It was prepared only for GC–MS analysis from thymohydroquinone (5 mg, 0.03 mmol), isovaleric acid (7 mg, 0.07 mmol), DCC (14.4 mg, 0.07 mmol), and DMAP (1.3 mg, 0.01 mmol) in 1 mL of dry CH_2_Cl_2_; RI = 2145 (DB-5MS column); MS (EI), *m*/*z* (%) 250 (6), 167 (10), 166 (100), 165 (2), 151 (12), 137 (2), 85 (10), 57 (15), 43 (3), 41 (6).

Mixed diesters of thymohydroquinone with isobutyric, 2-methylbutyric, and isovaleric acids were synthesized following a standard Steglich esterification protocol as described above in Section 3.3.3.

*6-Isobutyryloxythymyl 2-methylbutyrate (syn. 4-isobutyryloxy-2-isopropyl-5-methylphenyl 2-methylbutyrate, **6-iPr-3sBu**):* It was prepared from 6-isobutyryloxythymol (23.6 mg, 0.1 mmol), 2-methylbutyric acid (10.2 mg, 0.1 mmol), DCC (20.6 mg, 0.1 mmol), and DMAP (1.3 mg, 0.01 mmol) in 10 mL of CH_2_Cl_2_. Column chromatographic purification with *n*-hexane/diethyl ether (19:1, *v*/*v*) yielded 6-isobutyryloxythymyl 2-methylbutyrate 28 mg (88%); colorless liquid; RI = 2044 (DB-5MS column); UV (CH_3_CN) λ_max_(log ε) 273 (2.96), 268 (2.95), 193 (4.66) nm; FTIR (neat; cm^−1^) 2966, 2934, 2876, 1752, 1500, 1459, 1401, 1382, 1364, 1341, 1221, 1153, 1111, 1087, 1052, 1028, 965, 920, 905, 890, 820, 742; MS (EI), *m*/*z* (%) 320 (2), 236 (7), 167 (11), 166 (100), 151 (11), 137 (3), 85 (5), 71 (6), 57 (20), 43 (11); HRMS, *m*/*z* 320.1984 [M]^+^, calculated for C_19_H_28_O_4_^•+^, 320.1982; analyzed C 71.24, H 8.80, calculated for C_19_H_28_O_4_, C 71.22, H 8.81, O 19.97%; ^1^H NMR (CDCl_3_) δ 1.03 (ddd, ^3^*J*_3″A,4″_ = 7.5, ^3^*J*_3″B,4″_ = 7.5, ^4^*J*_2″,4″_ = 0.3, 3 H, CH_3_-4″), 1.18 (d, ^3^*J*_8,9/10_ = 6.9, 6 H, CH_3_-9 and CH_3-_10), 1.31 (d, ^3^*J*_2″,5″_ = 7.0, 3 H, CH_3_-5″), 1.34 (d, ^3^*J*_2′,3′_ = 7.0, 6 H, CH_3_-3′), 1.62 (dqd, ^2^*J*_3″A,3″B_ = −13.7, ^3^*J*_3″B,4″_ = 7.5, ^3^*J*_2″,3″B_ = 6.5, 1 H, CH-3″B), 1.86 (ddq, ^2^*J*_3″A,3″B_ = −13.7, ^3^*J*_2″,3″A_ = 7.5, ^3^*J*_3″A,4″_ = 7.5, 1 H, CH-3″A), 2.11 (dd, ^4^*J*_2,7_ = −0.7, ^5^*J*_5,7_ = 0.3, 3 H, CH_3_-7), 2.64 (dqdq, ^3^*J*_2″,3″A_ = 7.5, ^3^*J*_2″,5″_ = 7.0, ^3^*J*_2″,3″B_ = 6.5, ^4^*J*_2″,4″_ = 0.3, 1 H, CH-2″), 2.83 (sept, ^3^*J*_2′,3′_ = 7.0, 1 H, CH-2′), 2.97 (septd, ^3^*J*_8,9/10_ = 6.9, ^4^*J*_5,8_ = −0.6, 1 H, CH-8), 6.83 (qd, ^4^*J*_2,7_ = −0.7, ^5^*J*_2,5_ = 0.3, 1 H, CH-2), 6.90 (ddq, ^4^*J*_5,8_ = −0.6, ^5^*J*_2,5_ = 0.3, ^5^*J*_5,7_ = 0.3, 1 H, CH-5); ^13^C NMR (CDCl_3_) δ 11.9 (C-4″), 16.0 (C-7), 16.8 (C-5″), 19.2 (C-3′), 23.0 (C-9 and C-10), 26.9 (C-3″), 27.3 (C-8), 34.3 (C-2′), 41.4 (C-2″), 119.9 (C-5), 124.5 (C-2), 128.5 (C-1), 139.0 (C-4), 145.5 (C-3), 147.2 (C-6), 175.3 (C-1′), 175.4 (C-1″).

*6-(2-Methylbutyryloxy)thymyl isobutyrate (syn. 3-isobutyryloxy-5-isopropyl-2-methylphenyl 2-methylbutyrate, **6-sBu-3-iPr**):* It was prepared from 6-(2-methylbutyryloxy)thymol (25 mg, 0.1 mmol), isobutyric acid (8.8 mg, 0.1 mmol), DCC (20.6 mg, 0.1 mmol), and DMAP (1.3 mg, 0.01 mmol) in 10 mL of CH_2_Cl_2_. Column chromatographic purification with *n*-hexane/diethyl ether (19:1, *v*/*v*) yielded 6-(2-methylbutyryloxy)thymyl isobutyrate 30 mg (94%); colorless liquid; RI = 2045 (DB-5MS column); UV (CH_3_CN) λ_max_(log ε) 273 (3.16), 268 (3.16), 193 (4.88) nm; FTIR (neat; cm^−1^) 2965, 2931, 2876, 2355, 2342, 1752, 1500, 1459, 1401, 1381, 1364, 1341, 1223, 1153, 1111, 1087, 1051, 1028, 965, 919, 906, 816, 744, 669; MS (EI), *m*/*z* (%) 320 (2), 236 (6), 167 (11), 166 (100), 151 (10), 137 (2), 85 (4), 71 (7), 57 (17), 43 (13); HRMS, *m*/*z* 320.1986 [M]^+^, calculated for C_19_H_28_O_4_^•+^, 320.1982; analyzed C 71.24, H 8.80%, calculated for C_19_H_28_O_4_, C 71.22, H 8.81, O 19.97%; ^1^H NMR (CDCl_3_) δ 1.04 (ddd, ^3^*J*_3′A,4′_ = 7.5, ^3^*J*_3′B,4′_ = 7.5, ^4^*J*_2′,4′_ = 0.3, 3 H, CH_3_-4′), 1.18 (d, ^3^*J*_8,9/10_ = 6.9, 6 H, CH_3_-9 and CH_3-_10), 1.32 (d, ^3^*J*_2′,5′_ = 7.0, 3 H, CH_3_-5′), 1.33 (d, ^3^*J*_2″,3″_ = 7.0, 6 H, CH_3_-3″), 1.62 (dqd, ^2^*J*_3′A,3′B_ = −13.7, ^3^*J*_3′B,4′_ = 7.5, ^3^*J*_2′,3′B_ = 6.5, 1 H, CH-3′B), 1.87 (ddq, ^2^*J*_3′A,3′B_ = −13.7, ^3^*J*_2′,3′A_ = 7.5, ^3^*J*_3′A,4′_ = 7.5, 1 H, CH-3′A), 2.12 (dd, ^4^*J*_2,7_ = −0.7, ^5^*J*_5,7_ = 0.3, 3 H, CH_3_-7), 2.64 (dqdq, ^3^*J*_2′,3′A_ = 7.5, ^3^*J*_2′,5′_ = 7.0, ^3^*J*_2′,3′B_ = 6.5, ^4^*J*_2′,4′_ = 0.3, 1 H, CH-2′), 2.83 (sept, ^3^*J*_2″,3″_ = 7.0, 1 H, CH-2″), 2.95 (septd, ^3^*J*_8,9/10_ = 6.9, ^4^*J*_5,8_ = −0.6, 1 H, CH-8), 6.84 (qd, ^4^*J*_2,7_ = −0.7, ^5^*J*_2,5_ = 0.3, 1 H, CH-2), 6.89 (ddq, ^4^*J*_5,8_ = −0.6, ^5^*J*_2,5_ = 0.3, ^5^*J*_5,7_ = 0.3, 1 H, CH-5); ^13^C NMR (CDCl_3_) δ 11.9 (C-4′), 16.1 (C-7), 16.9 (C-5′), 19.1 (C-3″), 22.90/22.91 (C-9 and C-10), 26.9 (C-3′), 27.4 (C-8), 34.4 (C-2″), 41.4 (C-2′), 119.9 (C-5), 124.5 (C-2), 128.5 (C-1), 138.9 (C-4), 145.5 (C-3), 147.2 (C-6), 174.9 (C-1′), 175.8 (C-1″).

*6-Isobutyryloxythymyl isovalerate (syn. 4-isobutyryloxy-2-isopropyl-5-methylphenyl isovalerate, **6-iPr-3-iBu**):* It was prepared only for GC–MS analysis from 6-isobutyryloxythymol (7.1 mg, 0.03 mmol), isovaleric acid (3.1 mg, 0.03 mmol), DCC (6.2 mg, 0.03 mmol), and DMAP (1.3 mg, 0.01 mmol) in 1 mL of dry CH_2_Cl_2_; RI = 2047 (DB-5MS column); MS (EI), *m*/*z* (%) 236 (7), 167 (9), 166 (100), 151 (10), 137 (2), 85 (6), 71 (6), 57 (11), 43 (16), 41 (8).

*6-Isovaleroxythymyl isobutyrate (syn. 4-isobutyryloxy-5-isopropyl-2-methylphenyl isovalerate, **6-iBu-3-iPr**):* It was prepared only for GC–MS analysis from 6-hydroxythymyl isobutyrate (7.1 mg, 0.03 mmol), isovaleric acid (3.1 mg, 0.03 mmol), DCC (6.2 mg, 0.03 mmol), and DMAP (1.3 mg, 0.01 mmol) in 1 mL of dry CH_2_Cl_2_; RI = 2050 (DB-5MS column); MS (EI), *m*/*z* (%) 236 (6), 167 (12), 166 (100), 151 (12), 85 (8), 71 (14), 57 (18), 43 (39), 42 (5), 41 (22).

*6-(2-Methylbutyryloxy)thymyl isovalerate (syn. 4-(2-methylbutyryloxy)-2-isopropyl-5-methylphenyl isovalerate, **6-sBu-3-iBu**):* It was prepared only for GC–MS analysis from 6-(2-methylyloxy)thymol (7.5 mg, 0.03 mmol), isovaleric acid (3.1 mg, 0.03 mmol), DCC (6.2 mg, 0.03 mmol), and DMAP (1.3 mg, 0.01 mmol) in 1 mL of dry CH_2_Cl_2_; RI = 2143 (DB-5MS column); MS (EI), *m*/*z* (%) 250 (5), 167 (10), 166 (100), 151 (9), 85 (10), 69 (3), 57 (40), 55 (3), 43 (4), 41 (15).

*6-Isovaleroxythymyl 2-methylbutyrate (syn. 4-(2-methylbutyryloxy)-5-isopropyl-2-methylphenyl isovalerate, **6-iBu-3-sBu**):* It was prepared only for GC–MS analysis from 6-hydroxythymyl 2-methylbutyrate (7.5 mg, 0.03 mmol), isovaleric acid (3.1 mg, 0.03 mmol), DCC (6.2 mg, 0.03 mmol), and DMAP (1.3 mg, 0.01 mmol) in 1 mL of dry CH_2_Cl_2_; RI = 2144 (DB-5MS column); MS (EI), *m*/*z* (%) 250 (7), 167 (11), 166 (100), 151 (9), 150 (2), 137 (2), 85 (9), 57 (24), 43 (2), 41 (7).

### 3.6. Synthesis of 7-Acyloxythymyl Esters

#### Esterification of 7-Hydroxythymol with Corresponding Carboxylic Acids

7-Hydroxythymol was prepared according to the previously reported procedure [24]. Mono- and diesters of 7-hydroxythymol with isobutyric, 2-methylbutyric, and isovaleric acids were synthesized following a standard Steglich esterification protocol as described above in Section 3.3.3.

*7-Isobutyryloxythymol and 7-isobutyryloxythymyl isobutyrate:* These were prepared from 7-hydroxythymol (100 mg, 0.6 mmol), isobutyric acid (80 mg, 0.9 mmol), DCC (186 mg, 0.9 mmol), and DMAP (5 mg, 0.04 mmol) in 30 mL of CH_2_Cl_2_. Column chromatographic purification with *n*-hexane/diethyl ether (19:1, and then 4:1, *v*/*v*) yielded 7-isobutyryloxythymyl isobutyrate 68 mg (37%) and 7-isobutyryloxythymol 60 mg (42%).

*7-Isobutyryloxythymol (syn. 4-hydroxy-4-isopropylbenzyl isobutyrate):* RI = 1803 (DB-5MS column); FTIR (neat; cm^−1^) 3363, 2968, 2932, 2857, 1756, 1737, 1705, 1644, 1519, 1469, 1420, 1388, 1364, 1342, 1228, 1186, 1150, 1094, 1059, 969, 911, 893, 826, 788, 760; MS (EI), *m*/*z* (%) 166 (44), 151 (56), 149 (8), 121 (9), 103 (7), 91 (14), 77 (15), 71 (39), 43 (100), 41 (35); analyzed C 71.15, H 8.55%, calculated for C_14_H_20_O_3_, C 71.16, H 8.53, O 20.31%; ^1^H NMR (CDCl_3_) δ 1.19 (d, ^3^*J*_2′,3′_ = 7.0, 6 H, CH_3_-3′), 1.25 (d, ^3^*J*_8,9/10_ = 6.9, 6 H, CH_3_-9 and CH_3_-10), 2.60 (sept, ^3^*J*_2′,3′_ = 7.0, 1 H, CH-2′), 3.21 (septddd, ^3^*J*_8,9/10_ = 6.9, ^4^*J*_5,8_ = −0.5, ^5^*J*_6,8_ = 0.4, ^5^*J*_2,8_ = 0.3, 1 H, CH-8), 5.04 (ddd, ^4^*J*_2,7_ = −0.6, ^4^*J*_6,7_ = −0.6, ^5^*J*_5,7_ = 0.3, 2 H, CH_2_-7), 5.16 (brs, 1 H, OH), 6.76 (dtd, ^4^*J*_2,6_ = 1.8, ^4^*J*_2,7_ = −0.6, ^5^*J*_2,8_ = 0.3, 1 H, CH-2), 6.89 (ddtd, ^3^*J*_5,6_ = 7.8, ^4^*J*_2,6_ = 1.8, ^4^*J*_6,7_ = −0.6, ^5^*J*_6,8_ = 0.4, 1 H, CH-6), 7.18 (ddt, ^3^*J*_5,6_ = 7.8, ^4^*J*_5,8_ = −0.5, ^5^*J*_5,7_ = 0.3, 1 H, CH-5); ^13^C NMR (CDCl_3_) δ 19.1 (C-3′), 22.7 (C-9 and C-10), 27.0 (C-8), 34.2 (C-2′), 66.0 (C-7), 115.0 (C-2), 120.6 (C-6), 126.7 (C-5), 134.7 (C-4), 134.9 (C-1), 153.1 (C-3), 177.5 (C-1′).

*7-Isobutyryloxythymyl isobutyrate (syn. 3-isobutyryloxy-4-isopropylbenzyl isobutyrate, **7-iPr-3-iPr**):* white solid; RI = 1961 (DB-5MS column); UV (CH_3_CN) λ_max_(log ε) 272 (2.59), 264 (2.62), 194 (4.65) nm; FTIR (neat; cm^−1^) 2969, 2937, 2875, 2363, 2331, 2181, 2040, 1755, 1734, 1575, 1507, 1469, 1422, 1387, 1365, 1341, 1283, 1128, 1185, 1148, 1115, 1093, 1058, 966, 911, 882, 824, 766; MS (EI), *m*/*z* (%) 236 (45), 221 (14), 218 (22), 166 (100), 149 (25), 133 (14), 71 (60), 43 (88), 41 (16); HRMS, *m*/*z* 306.1829 [M]^+^, calculated for C_18_H_26_O_4_^•+^, 306.1826; analyzed C 70.53, H 8.57%, calculated for C_18_H_26_O_4_, C 70.56, H 8.55, O 20.89%; ^1^H NMR (CDCl_3_) δ 1.18 (d, ^3^*J*_2′,3′_ = 7.0, 6 H, CH_3_-3′), 1.20 (d, ^3^*J*_8,9/10_ = 6.9, 6 H, CH_3_-9 and CH_3_-10), 1.34 (d, ^3^*J*_2″,3″_ = 7.0, 6 H, CH_3_-3″), 2.59 (sept, ^3^*J*_2′,3′_ = 7.0, 1 H, CH-2′), 2.84 (sept, ^3^*J*_2″,3″_ = 7.0, 1 H, CH-2″), 3.01 (septddd, ^3^*J*_8,9/10_ = 6.9, ^4^*J*_5,8_ = −0.6, ^5^*J*_6,8_ = 0.4, ^5^*J*_2,8_ = 0.3, 1 H, CH-8), 5.07 (ddd, ^4^*J*_6,7_ = −0.6, ^4^*J*_2,7_ = −0.5, ^5^*J*_5,7_ = 0.3, 2 H, CH_2_-7), 6.97 (dtd, ^4^*J*_2,6_ = 1.8, ^4^*J*_2,7_ = −0.5, ^5^*J*_2,8_ = 0.3, 1 H, CH-2), 7.19 (ddtd, ^3^*J*_5,6_ = 8.0, ^4^*J*_2,6_ = 1.8, ^4^*J*_6,7_ = −0.6, ^5^*J*_6,8_ = 0.4, 1 H, CH-6), 7.30 (ddt, ^3^*J*_5,6_ = 8.0, ^4^*J*_5,8_ = −0.6, ^5^*J*_5,7_ = 0.3, 1 H, CH-5); ^13^C NMR (CDCl_3_) δ 19.10^a^ (C-3′), 19.12^a^ (C-3″), 23.0 (C-9 and C-10), 27.3 (C-8), 34.1 (C-2′), 34.4 (C-2″), 65.5 (C-7), 122.0 (C-2), 125.9 (C-6), 126.9 (C-5), 135.0 (C-1), 140.2 (C-4), 148.3 (C-3), 175.7 (C-1″), 177.0 (C-1′). ^a^ The signals of C-3′ and C-3″ may be interchanged.

*7-(2-Methylbutyryloxy)thymol and 7-(2-methylbutyryoxy)thymyl 2-methylbutyrate:* These were prepared from 7-hydroxythymol (100 mg, 0.6 mmol), 2-methylbutyric acid (92 mg, 0.9 mmol), DCC (186 mg, 0.9 mmol), and DMAP (5 mg, 0.04 mmol) in 30 mL of CH_2_Cl_2_. Column chromatographic purification with *n*-hexane/diethyl ether (19:1, and then 4:1, *v*/*v*) yielded 7-(2-methylbutyryoxy)thymyl 2-methylbutyrate 70 mg (35%) and 7-(2-methylbutyryloxy)thymol 62 mg (42%).

*7-(2-Methylbutyryloxy)thymol (syn. 3-hydroxy-4-isopropylbenzyl 2-methylbutyrate):* yellowish liquid; RI = 1889 (DB-5MS column); MS (EI), *m*/*z* (%) 166 (44), 151 (56), 149 (8), 121 (9), 103 (7), 91 (14), 77 (15), 71 (39), 43 (100), 41 (35); analyzed C 71.95, H 8.87%, calculated for C_15_H_22_O_3_, C 71.97, H 8.86, O 19.17%; ^1^H NMR (CDCl_3_) δ 0.90 (ddd, ^3^*J*_3′A,4′_ = 7.5, ^3^*J*_3′B,4′_ = 7.5, ^4^*J*_2′,4′_ = 0.3, 3 H, CH_3_-4′), 1.16 (d, ^3^*J*_2′,5′_ = 7.0, 3 H, CH_3_-5′), 1.25 (d, ^3^*J*_8,9/10_ = 6.9, 6 H, CH_3_-9 and CH_3_-10), 1.49 (dqd, ^2^*J*_3′A,3′B_ = −13.7, ^3^*J*_3′B,4′_ = 7.5, ^3^*J*_2′,3′B_ = 6.5, 1 H, CH-3′B), 1.70 (ddq, ^2^*J*_3′A,3′B_ = −13.7, ^3^*J*_2′,3′A_ = 7.5, ^3^*J*_3′A,4′_ = 7.5, 1 H, CH-3′A), 2.42 (dqdq, ^3^*J*_2′,3′A_ = 7.5, ^3^*J*_2′,5′_ = 7.0, ^3^*J*_2′,3′B_ = 6.5, ^4^*J*_2′,4′_ = 0.3, 1 H, CH-2′), 3.21 (septddd, ^3^*J*_8,9/10_ = 6.9, ^4^*J*_5,8_ = −0.5, ^5^*J*_6,8_ = 0.4, ^5^*J*_2,8_ = 0.3, 1 H, CH-8), 5.04 (ddd, ^4^*J*_2,7_ = −0.6, ^4^*J*_6,7_ = −0.6, ^5^*J*_5,7_ = 0.3, 2 H, CH_2_-7), 5.22 (brs, 1 H, OH), 6.77 (dtd, ^4^*J*_2,6_ = 1.8, ^4^*J*_2,7_ = −0.6, ^5^*J*_2,8_ = 0.3, 1 H, CH-2), 6.89 (ddtd, ^3^*J*_5,6_ = 7.8, ^4^*J*_2,6_ = 1.8, ^4^*J*_6,7_ = −0.6, ^5^*J*_6,8_ = 0.4, 1 H, CH-6), 7.18 (ddt, ^3^*J*_5,6_ = 7.8, ^4^*J*_5,8_ = −0.5, ^5^*J*_5,7_ = 0.3, 1 H, CH-5); ^13^C NMR (CDCl_3_) δ 11.8 (C-4′), 16.7 (C-5′), 22.7 (C-9 and C-10), 26.9 (C-3′), 27.0 (C-8), 41.2 (C-2′), 65.9 (C-7), 115.1 (C-2), 120.7 (C-6), 126.7 (C-5), 134.7 (C-4), 134.9 (C-1), 153.1 (C-3), 177.1 (C-1′).

*7-(2-Methylbutyryoxy)thymyl 2-methylbutyrate (syn. 4-isopropyl-3-(2-methylbutyriloxy)benzyl 2-methylbutyrate, **7-sBu-3-sBu**):* colorless liquid; RI = 2135 (DB-5MS column); UV (CH_3_CN) λ_max_(log ε) 271 (2.61), 264 (2.64), 194 (4.65) nm; FTIR (neat; cm^−1^) 2965, 2935, 2877, 2361, 2343, 1754, 1733, 1575, 1507, 1460, 1422, 1382, 1363, 1283, 1216, 1175, 1148, 1120, 1087, 1058, 1009, 967, 902, 822, 734, 669; MS (EI), *m*/*z* (%) 250 (34), 232 (13), 167 (8), 166 (74), 149 (18), 148 (9), 85 (37), 57 (100), 41 (16); HRMS, *m*/*z* 334.2142 [M]^+^, calculated for C_20_H_30_O_4_^•+^, 334.2139; analyzed C 71.80, H 9.05, O 19.15%, calculated for C_20_H_30_O_4_, C 71.82, H 9.04, O 19.13%; ^1^H NMR (CDCl_3_) δ 0.90 (ddd, ^3^*J*_3′A,4′_ = 7.5, ^3^*J*_3′B,4′_ = 7.5, ^4^*J*_2′,4′_ = 0.3, 3 H, CH_3_-4′), 1.04 (ddd, ^3^*J*_3″A,4″_ = 7.5, ^3^*J*_3″B,4″_ = 7.5, ^4^*J*_2″,4″_ = 0.3, 3 H, CH_3_-4″), 1.16 (d, ^3^*J*_2′,5′_ = 7.0, 3 H, CH_3_-5′), 1.20 (d, ^3^*J*_8,9/10_ = 6.9, 6 H, CH_3_-9 and CH_3_-10), 1.32 (d, ^3^*J*_2″,5″_ = 7.0, 3 H, CH_3_-5″), 1.48 (dqd, ^2^*J*_3′A,3′B_ = −13.7, ^3^*J*_3′B,4′_ = 7.5, ^3^*J*_2′,3′B_ = 6.5, 1 H, CH-3′B), 1.63 (dqd, ^2^*J*_3″A,3″B_ = −13.7, ^3^*J*_3″B,4″_ = 7.5, ^3^*J*_2″,3″B_ = 6.5, 1 H, CH-3″B), 1.70 (ddq, ^2^*J*_3′A,3′B_ = −13.7, ^3^*J*_2′,3′A_ = 7.5, ^3^*J*_3′A,4′_ = 7.5, 1 H, CH-3′A), 1.87 (ddq, ^2^*J*_3″A,3″B_ = −13.7, ^3^*J*_2″,3″A_ = 7.5, ^3^*J*_3″A,4″_ = 7.5, 1 H, CH-3″A), 2.42 (dqdq, ^3^*J*_2′,3′A_ = 7.5, ^3^*J*_2′,5′_ = 7.0, ^3^*J*_2′,3′B_ = 6.5, ^4^*J*_2′,4′_ = 0.3, 1 H, CH-2′), 2.66 (dqdq, ^3^*J*_2″,3″A_ = 7.5, ^3^*J*_2″,5″_ = 7.0, ^3^*J*_2″,3″B_ = 6.5, ^4^*J*_2″,4″_ = 0.3, 1 H, CH-2″), 3.02 (septddd, ^3^*J*_8,9/10_ = 6.9, ^4^*J*_5,8_ = −0.6, ^5^*J*_6,8_ = 0.4, ^5^*J*_2,8_ = 0.3, 1 H, CH-8), 5.08 (ddd, ^4^*J*_2,7_ = −0.6, ^4^*J*_6,7_ = −0.6, ^5^*J*_5,7_ = 0.3, 2 H, CH_2_-7), 6.96 (dtd, ^4^*J*_2,6_ = 1.8, ^4^*J*_2,7_ = −0.6, ^5^*J*_2,8_ = 0.3, 1 H, CH-2), 7.19 (ddtd, ^3^*J*_5,6_ = 8.0, ^4^*J*_2,6_ = 1.8, ^4^*J*_6,7_ = −0.6, ^5^*J*_6,8_ = 0.4, 1 H, CH-6), 7.30 (ddt, ^3^*J*_5,6_ = 8.0, ^4^*J*_5,8_ = −0.6, ^5^*J*_5,7_ = 0.3, 1 H, CH-5); ^13^C NMR (CDCl_3_) δ 11.75 (C-4′), 11.84 (C-4″), 16.7 (C-5′), 16.8 (C-5″), 23.0 (C-9 and C-10), 26.85 (C-3″), 26.90 (C-3′), 27.2 (C-8), 41.2 (C-2′), 41.5 (C-2″), 65.4 (C-7), 122.0 (C-2), 125.9 (C-6), 126.9 (C-5), 135.0 (C-1), 140.2 (C-4), 148.3 (C-3), 175.3 (C-1″), 176.7 (C-1′).

*7-Isovaleroxythymol and 7-isovaleroxythymyl isovalerate:* These were prepared from 7-hydroxythymol (100 mg, 0.6 mmol), isovaleric acid (92 mg, 0.9 mmol), DCC (186 mg, 0.9 mmol), and DMAP (5 mg, 0.04 mmol) in 30 mL of CH_2_Cl_2_. Column chromatographic purification with *n*-hexane/diethyl ether (19:1, and then 4:1, *v*/*v*) yielded 7-isovaleroxythymyl isovalerate 75 mg (47%) and 7-isovaleroxythymol 70 mg (37%).

*7-Isovaleroxythymol (syn. 3-hydroxy-4-isopropylbenzyl isovalerate):* yellowish liquid; RI = 1916 (DB-5MS column); MS (EI), *m*/*z* (%) 250 (17), 166 (100), 149 (31), 133 (21), 123 (32), 85 (21), 60 (19), 57 (41), 43 (37), 41 (38).

*7-isovaleroxythymyl isovalerate (syn. 4-isopropyl-3-isovaleroxybenzyl isovalerate, **7-iBu-3-iBu**):* colorless liquid; RI = 2150 (DB-5MS column); FTIR (neat; cm^−1^) 2961, 2873, 1737, 1657, 1506, 1466, 1422, 1370, 1292, 1238, 1182, 1151, 1091, 1059, 974, 914, 890, 822; MS (EI), *m*/*z* (%) 250 (34), 235 (9), 167 (10), 166 (100), 149 (11), 133 (7), 123 (7), 85 (27), 57 (39), 41 (12); HRMS, *m*/*z* 334.2140 [M]^+^, calculated for C_20_H_30_O_4_^•+^, 334.2139; analyzed C 71.84, H 9.03%, calculated for C_20_H_30_O_4_, C 71.82, H 9.04, O 19.13%; ^1^H NMR (CDCl_3_) δ 0.95 (d, ^3^*J*_3′,4′_ = 6.7, 6 H, CH_3_-4′), 1.07 (d, ^3^*J*_3″,4″_ = 6.7, 6 H, CH_3_-4″), 1.20 (d, ^3^*J*_8,9/10_ = 6.9, 6 H, CH_3_-9 and CH_3_-10), 2.12 (tsept, ^3^*J*_2′,3′_ = 7.2, ^3^*J*_3′,4′_ = 6.7, 1 H, CH-3′), 2.23 (d, ^3^*J*_2′,3′_ = 7.2, 2 H, CH_2_-2′), 2.27 (tsept, ^3^*J*_2″,3″_ = 7.2, ^3^*J*_3″,4″_ = 6.7, 1 H, CH-3″), 2.47 (d, ^3^*J*_2″,3″_ = 7.2, 2 H, CH_2_-2″), 3.01 (septddd, ^3^*J*_8,9/10_ = 6.9, ^4^*J*_5,8_ = −0.6, ^5^*J*_6,8_ = 0.4, ^5^*J*_2,8_ = 0.3, 1 H, CH-8), 5.07 (ddd, ^4^*J*_2,7_ = −0.6, ^4^*J*_6,7_ = −0.6, ^5^*J*_5,7_ = 0.3, 2 H, CH_2_-7), 6.98 (dtd, ^4^*J*_2,6_ = 1.8, ^4^*J*_2,7_ = −0.6, ^5^*J*_2,8_ = 0.3, 1 H, CH-2), 7.19 (ddtd, ^3^*J*_5,6_ = 8.0, ^4^*J*_2,6_ = 1.8, ^4^*J*_6,7_ = −0.6, ^5^*J*_6,8_ = 0.4, 1 H, CH-6), 7.30 (ddt, ^3^*J*_5,6_ = 8.0, ^4^*J*_5,8_ = −0.6, ^5^*J*_5,7_ = 0.3, 1 H, CH-5); ^13^C NMR (CDCl_3_) δ 22.5 (C-4′), 22.6 (C-4″), 23.1 (C-9 and C-10), 25.8 (C-3′), 25.9 (C-3″), 27.4 (C-8), 43.4 (C-2″), 43.5 (C-2′), 65.4 (C-7), 122.2 (C-2), 126.1 (C-6), 127.0 (C-5), 134.9 (C-1), 140.3 (C-4), 148.2 (C-3), 171.7 (C-1″), 173.0 (C-1′).

Mixed diesters of 7-hydroxythymol with isobutyric, 2-methylbutyric, and isovaleric acids were synthesized following a standard Steglich esterification protocol as described above in Section 3.3.3.

*7-Isobutyryloxythymyl 2-methylbutyrate (syn. 5-(isobutyryloxymethyl)-2-isopropylphenyl 2-methylbutyrate, **7-iPr-3-sBu**):* It was prepared from 7-isobutyryloxythymol (20 mg, 0.08 mmol), 2-methylbutyric acid (9 mg, 0.09 mmol), DCC (18.5 mg, 0.09 mmol), and DMAP (1.3 mg, 0.01 mmol) in 10 mL of CH_2_Cl_2_. Column chromatographic purification with *n*-hexane/diethyl ether (19:1, *v*/*v*) yielded 7-isobutyryloxythymyl 2-methylbutyrate 25 mg (92%); white solid; RI = 2051 (DB-5MS column); UV (CH_3_CN) λ_max_(log ε) 271 (2.69), 264 (2.70), 194 (4.74) nm; FTIR (neat; cm^−1^) 3301, 2959, 2915, 2849, 2159, 2016, 1729, 1507, 1469, 1420, 1390, 1364, 1287, 1254, 1215, 1193, 1177, 1150, 1105, 1061, 1047, 991, 943, 903, 822, 720, 658; MS (EI), *m*/*z* (%) 236 (43), 232 (15), 166 (67), 149 (18), 148 (10), 133 (11), 85 (35), 57 (100), 41 (17); HRMS, *m*/*z* 320.1978 [M]^+^, calculated for C_19_H_28_O_4_^•+^, 320.1982; analyzed C 71.24, H 8.80%, calculated for C_19_H_28_O_4_, C 71.22, H 8.81, O 19.97%; ^1^H NMR (CDCl_3_) δ 1.04 (ddd, ^3^*J*_3″A,4″_ = 7.5, ^3^*J*_3″B,4″_ = 7.5, ^4^*J*_2″,4″_ = 0.3, 3 H, CH_3_-4″), 1.18 (d, ^3^*J*_2′,3′_ = 7.0, 6 H, CH_3_-3′), 1.20 (d, ^3^*J*_8,9/10_ = 6.9, 6 H, CH_3_-9 and CH_3_-10), 1.33 (d, ^3^*J*_2″,5″_ = 7.0, 3 H, CH_3_-5″), 1.63 (dqd, ^2^*J*_3″A,3″B_ = −13.7, ^3^*J*_3″B,4″_ = 7.5, ^3^*J*_2″,3″B_ = 6.5, 1 H, CH-3″B), 1.87 (ddq, ^2^*J*_3″A,3″B_ = −13.7, ^3^*J*_2″,3″A_ = 7.5, ^3^*J*_3″A,4″_ = 7.5, 1 H, CH-3″A), 2.59 (sept, ^3^*J*_2′,3′_ = 7.0, 1 H, CH-2′), 2.66 (dqdq, ^3^*J*_2″,3″A_ = 7.5, ^3^*J*_2″,5″_ = 7.0, ^3^*J*_2″,3″B_ = 6.5, ^4^*J*_2″,4″_ = 0.3, 1 H, CH-2″), 3.02 (septddd, ^3^*J*_8,9/10_ = 6.9, ^4^*J*_5,8_ = −0.5, ^5^*J*_6,8_ = 0.4, ^5^*J*_2,8_ = 0.3, 1 H, CH-8), 5.07 (ddd, ^4^*J*_2,7_ = −0.6, ^4^*J*_6,7_ = −0.6, ^5^*J*_5,7_ = 0.3, 2 H, CH_2_-7), 6.96 (dtd, ^4^*J*_2,6_ = 1.8, ^4^*J*_2,7_ = −0.6, ^5^*J*_2,8_ = 0.3, 1 H, CH-2), 7.19 (ddtd, ^3^*J*_5,6_ = 8.0, ^4^*J*_2,6_ = 1.8, ^4^*J*_6,7_ = −0.6, ^5^*J*_6,8_ = 0.4, 1 H, CH-6), 7.30 (ddt, ^3^*J*_5,6_ = 8.0, ^4^*J*_5,8_ = −0.6, ^5^*J*_5,7_ = 0.3, 1 H, CH-5); ^13^C NMR (CDCl_3_) δ 11.9 (C-4″), 16.8 (C-5″), 19.1 (C-3′), 23.0 (C-9 and C-10), 26.9 (C-3″), 27.3 (C-8), 34.1 (C-2′), 41.5 (C-2″), 65.5 (C-7), 122.0 (C-2), 125.9 (C-6), 126.9 (C-5), 135.0 (C-1), 140.2 (C-4), 148.3 (C-3), 175.3 (C-1″), 177.1 (C-1′).

*7-(2-Methylbutyryloxy)thymyl isobutyrate (syn. 3-isobutyryloxy-4-isopropylbenzyl 2-methylbutyrate, **7-sBu-3-iPr**):* It was prepared from 7-(2-methylbutyryloxy)thymol (20 mg, 0.08 mmol), isobutyric acid (8 mg, 0.09 mmol), DCC (18.5 mg, 0.09 mmol), and DMAP (1.3 mg, 0.01 mmol) in 10 mL of CH_2_Cl_2_. Column chromatographic purification with *n*-hexane/diethyl ether (19:1, *v*/*v*) yielded 7-(2-methylbutyryloxy)thymyl isobutyrate 22 mg (86%); white solid; RI = 2045 (DB-5MS column); UV (CH_3_CN) λ_max_(log ε) 271 (2.68), 264 (2.68), 194 (4.73) nm; FTIR (neat; cm^−1^) 2965, 2930, 2875, 2039, 1757, 1733, 1577, 1507, 1460, 1422, 1385, 1347, 1283, 1228, 1179, 1124, 1092, 1058, 1047, 964, 911, 884, 822, 767; MS (EI), *m*/*z* (%) 250 (30), 218 (19), 166 (100), 149 (24), 133 (12), 71 (42), 57 (21), 41 (14); HRMS, *m*/*z* 320.1985 [M]^+^, calculated for C_19_H_28_O_4_^•+^, 320.1982; analyzed C 71.24, H 8.80%, calculated for C_19_H_28_O_4_, C 71.22, H 8.81, O 19.97%; ^1^H NMR (CDCl_3_) δ 0.90 (ddd, ^3^*J*_3′A,4′_ = 7.5, ^3^*J*_3′B,4′_ = 7.5, ^4^*J*_2′,4′_ = 0.3, 3 H, CH_3_-4′), 1.16 (d, ^3^*J*_2′,5′_ = 7.0, 3 H, CH_3_-5′), 1.20 (d, ^3^*J*_8,9/10_ = 6.9, 6 H, CH_3_-9 and CH_3_-10), 1.35 (d, ^3^*J*_2″,3″_ = 7.0, 6 H, CH_3_-3″), 1.48 (dqd, ^2^*J*_3′A,3′B_ = −13.7, ^3^*J*_3′B,4′_ = 7.5, ^3^*J*_2′,3′B_ = 6.5, 1 H, CH-3′B), 1.70 (ddq, ^2^*J*_3′A,3′B_ = −13.7, ^3^*J*_2′,3′A_ = 7.5, ^3^*J*_3′A,4′_ = 7.5, 1 H, CH-3′A), 2.42 (dqdq, ^3^*J*_2′,3′A_ = 7.5, ^3^*J*_2′,5′_ = 7.0, ^3^*J*_2′,3′B_ = 6.5, ^4^*J*_2′,4′_ = 0.3, 1 H, CH-2′), 2.85 (sept, ^3^*J*_2″,3″_ = 7.0, 1 H, CH-2″), 3.00 (septddd, ^3^*J*_8,9/10_ = 6.9, ^4^*J*_5,8_ = −0.6, ^5^*J*_6,8_ = 0.4, ^5^*J*_2,8_ = 0.3, 1 H, CH-8), 5.07 (ddd, ^4^*J*_2,7_ = −0.6, ^4^*J*_6,7_ = −0.6, ^5^*J*_5,7_ = 0.3, 2 H, CH_2_-7), 6.97 (dtd, ^4^*J*_2,6_ = 1.8, ^4^*J*_2,7_ = −0.6, ^5^*J*_2,8_ = 0.3, 1 H, CH-2), 7.19 (ddtd, ^3^*J*_5,6_ = 8.0, ^4^*J*_2,6_ = 1.8, ^4^*J*_6,7_ = −0.6, ^5^*J*_6,8_ = 0.4, 1 H, CH-6), 7.30 (ddt, ^3^*J*_5,6_ = 8.0, ^4^*J*_5,8_ = −0.6, ^5^*J*_5,7_ = 0.3, 1 H, CH-5); ^13^C NMR (CDCl_3_) δ 11.8 (C-4′), 16.7 (C-5′), 19.1 (C-3″), 23.0 (C-9 and C-10), 26.9 (C-3′), 27.3 (C-8), 34.4 (C-2″), 41.2 (C-2′), 65.4 (C-7), 122.1 (C-2), 125.9 (C-6), 126.9 (C-5), 135.0 (C-1), 140.2 (C-4), 148.3 (C-3), 175.7 (C-1″), 176.7 (C-1′).

*7-Isobutyryloxythymyl isovalerate (syn. 5-(isobutyryloxymethyl)-2-isopropylphenyl isovalerate, **7-iPr-3-iBu**):* It was prepared from 7-isobutyryloxythymol (20 mg, 0.08 mmol), isovaleric acid (9 mg, 0.09 mmol), DCC (18.5 mg, 0.09 mmol), and DMAP (1.3 mg, 0.01 mmol) in 10 mL of CH_2_Cl_2_. Column chromatographic purification with *n*-hexane/diethyl ether (19:1, *v*/*v*) yielded 7-isobutyryloxythymyl isovalerate 26 mg (96%); colorless liquid; RI = 2057 (DB-5MS column); FTIR (neat; cm^−1^) 2962, 2916, 2850, 1757, 1732, 1469, 1422, 1387, 1293, 1229, 1181, 1152, 1118, 1095, 1059, 1047, 991, 943, 912, 823, 720; MS (EI), *m*/*z* (%) 236 (53), 221 (15), 166 (100), 149 (21), 85 (40), 57 (53), 44 (42), 43 (64), 42 (18), 41 (63); HRMS, *m*/*z* 320.1980 [M]^+^, calculated for C_19_H_28_O_4_^•+^, 320.1982; analyzed C 71.25, H 8.83%, calculated for C_19_H_28_O_4_, C 71.22, H 8.81, O 19.97%; ^1^H NMR (CDCl_3_) δ 1.07 (d, ^3^*J*_3″,4″_ = 6.7, 6 H, CH_3_-4″), 1.19 (d, ^3^*J*_2′,3′_ = 7.0, 6 H, CH_3_-3′), 1.20 (d, ^3^*J*_8,9/10_ = 6.9, 6 H, CH_3_-9 and CH_3_-10), 2.27 (tsept, ^3^*J*_2″,3″_ = 7.2, ^3^*J*_3″,4″_ = 6.7, 1 H, CH-3″), 2.47 (d, ^3^*J*_2″,3″_ = 7.2, 2 H, CH_2_-2″), 2.60 (sept, ^3^*J*_2′,3′_ = 7.0, 1 H, CH-2′), 3.01 (septddd, ^3^*J*_8,9/10_ = 6.9, ^4^*J*_5,8_ = −0.6, ^5^*J*_6,8_ = 0.4, ^5^*J*_2,8_ = 0.3, 1 H, CH-8), 5.07 (ddd, ^4^*J*_2,7_ = −0.6, ^4^*J*_6,7_ = −0.6, ^5^*J*_5,7_ = 0.3, 2 H, CH_2_-7), 6.97 (dtd, ^4^*J*_2,6_ = 1.8, ^4^*J*_2,7_ = −0.6, ^5^*J*_2,8_ = 0.3, 1 H, CH-2), 7.19 (ddtd, ^3^*J*_5,6_ = 8.0, ^4^*J*_2,6_ = 1.8, ^4^*J*_6,7_ = −0.6, ^5^*J*_6,8_ = 0.4, 1 H, CH-6), 7.30 (ddt, ^3^*J*_5,6_ = 8.0, ^4^*J*_5,8_ = −0.6, ^5^*J*_5,7_ = 0.3, 1 H, CH-5); ^13^C NMR (CDCl_3_) δ 19.1 (C-3′), 22.6 (C-4″), 23.1 (C-9 and C-10), 25.9 (C-3″), 27.4 (C-8), 34.1 (C-2′), 43.5 (C-2″), 65.5 (C-7), 122.0 (C-2), 125.9 (C-6), 127.0 (C-5), 135.0 (C-1), 140.2 (C-4), 148.2 (C-3), 171.8 (C-1″), 177.1 (C-1′).

*7-Isovaleroxythymyl isobutyrate (syn. 3-isobutyryloxy-4-isopropylbenzyl isovalerate, **7-iBu-3-iPr**):* It was prepared from 7-isovaleroxythymol (20 mg, 0.08 mmol), isobutyric acid (8 mg, 0.09 mmol), DCC (18.5 mg, 0.09 mmol), and DMAP (1.3 mg, 0.01 mmol) in 10 mL of CH_2_Cl_2_. Column chromatographic purification with *n*-hexane/diethyl ether (19:1, *v*/*v*) yielded 7-isobutyryloxythymyl isovalerate 24 mg (94%); colorless liquid; RI = 2061 (DB-5MS column); FTIR (neat; cm^−1^) 2964, 2874, 1757, 1736, 1576, 1506, 1469, 1422, 1387, 1365, 1342, 1283, 1238, 1187, 1148, 1098, 1059, 967, 914, 888, 825, 734; MS (EI), *m*/*z* (%) 236 (51), 166 (99), 149 (23), 133 (13), 85 (61), 71 (18), 57 (100), 43 (90), 42 (13), 41 (72); HRMS, *m*/*z* 320.1985 [M]^+^, calculated for C_19_H_28_O_4_^•+^, 320.1982; analyzed C 71.22, H 8.84%, calculated for C_19_H_28_O_4_, C 71.22, H 8.81, O 19.97%; ^1^H NMR (CDCl_3_) δ 0.95 (d, ^3^*J*_3′,4′_ = 6.7, 6 H, CH_3_-4′), 1.20 (d, ^3^*J*_8,9/10_ = 6.9, 6 H, CH_3_-9 and CH_3_-10), 1.35 (d, ^3^*J*_2″,3″_ = 7.0, 6 H, CH_3_-3″), 2.12 (tsept, ^3^*J*_2′,3′_ = 7.2, ^3^*J*_3′,4′_ = 6.7, 1 H, CH-3′), 2.23 (d, ^3^*J*_2′,3′_ = 7.2, 2 H, CH_2_-2′), 2.85 (sept, ^3^*J*_2″,3″_ = 7.0, 1 H, CH-2″), 3.00 (septddd, ^3^*J*_8,9/10_ = 6.9, ^4^*J*_5,8_ = −0.6, ^5^*J*_6,8_ = 0.4, ^5^*J*_2,8_ = 0.3, 1 H, CH-8), 5.07 (ddd, ^4^*J*_6,7_ = −0.6, ^4^*J*_2,7_ = −0.5, ^5^*J*_5,7_ = 0.3, 2 H, CH_2_-7), 6.97 (dtd, ^4^*J*_2,6_ = 1.8, ^4^*J*_2,7_ = −0.5, ^5^*J*_2,8_ = 0.3, 1 H, CH-2), 7.19 (ddtd, ^3^*J*_5,6_ = 8.0, ^4^*J*_2,6_ = 1.8, ^4^*J*_6,7_ = −0.6, ^5^*J*_6,8_ = 0.4, 1 H, CH-6), 7.30 (ddt, ^3^*J*_5,6_ = 8.0, ^4^*J*_5,8_ = −0.6, ^5^*J*_5,7_ = 0.3, 1 H, CH-5); ^13^C NMR (CDCl_3_) δ 19.1 (C-3″), 22.6 (C-4′), 23.0 (C-9 and C-10), 25.8 (C-3′), 27.3 (C-8), 34.4 (C-2″), 43.5 (C-2′), 65.5 (C-7), 122.2 (C-2), 126.1 (C-6), 126.9 (C-5), 134.9 (C-1), 140.3 (C-4), 148.3 (C-3), 173.1 (C-1′), 175.7 (C-1″).

*7-(2-Methylbutyryloxy)thymyl isovalerate (syn. 4-isopropyl-3-(2-methylbutyryloxy)benzyl isovalerate, **7-sBu-3-iBu**):* It was prepared from 7-(2-methylbutyryloxy)thymol (20 mg, 0.08 mmol), isovaleric acid (9 mg, 0.09 mmol), DCC (18.5 mg, 0.09 mmol), and DMAP (1.3 mg, 0.01 mmol) in 10 mL of CH_2_Cl_2_. Column chromatographic purification with *n*-hexane/diethyl ether (19:1, *v*/*v*) yielded 7-(2-ethylbutyryloxy)thymyl isobutyrate 24 mg (90%); colorless liquid; RI = 2142 (DB-5MS column); FTIR (neat; cm^−1^) 2963, 2874, 1755, 1736, 1577, 1507, 1462, 1422, 1364, 1293, 1246, 1216, 1167, 1151, 1118, 1089, 1059, 1007, 904, 822, 734; MS (EI), *m*/*z* (%) 250 (42), 167 (10), 166 (100), 149 (17), 148 (8), 133 (9), 91 (8), 85 (44), 57 (91), 41 (30); HRMS, *m*/*z* 334.2138 [M]^+^, calculated for C_20_H_30_O_4_^•+^, 334.2139; analyzed C 71.83, H 9.02%, calculated for C_20_H_30_O_4_, C 71.82, H 9.04, O 19.13%; ^1^H NMR (CDCl_3_) δ 0.90 (ddd, ^3^*J*_3′A,4′_ = 7.5, ^3^*J*_3′B,4′_ = 7.5, ^4^*J*_2′,4′_ = 0.3, 3 H, CH_3_-4′), 1.07 (d, ^3^*J*_3″,4″_ = 6.7, 6 H, CH_3_-4″), 1.16 (d, ^3^*J*_2′,5′_ = 7.0, 3 H, CH_3_-5′), 1.20 (d, ^3^*J*_8,9/10_ = 6.9, 6 H, CH_3_-9 and CH_3_-10), 1.49 (dqd, ^2^*J*_3′A,3′B_ = −13.7, ^3^*J*_3′B,4′_ = 7.5, ^3^*J*_2′,3′B_ = 6.5, 1 H, CH-3′B), 1.70 (ddq, ^2^*J*_3′A,3′B_ = −13.7, ^3^*J*_2′,3′A_ = 7.5, ^3^*J*_3′A,4′_ = 7.5, 1 H, CH-3′A), 2.27 (tsept, ^3^*J*_2″,3″_ = 7.2, ^3^*J*_3″,4″_ = 6.7, 1 H, CH-3″), 2.42 (dqdq, ^3^*J*_2′,3′A_ = 7.5, ^3^*J*_2′,5′_ = 7.0, ^3^*J*_2′,3′B_ = 6.5, ^4^*J*_2′,4′_ = 0.3, 1 H, CH-2′), 2.47 (d, ^3^*J*_2″,3″_ = 7.2, 2 H, CH_2_-2″), 3.01 (septddd, ^3^*J*_8,9/10_ = 6.9, ^4^*J*_5,8_ = −0.6, ^5^*J*_2,8_ = 0.4, ^5^*J*_6,8_ = 0.4, 1 H, CH-8), 5.08 (ddd, ^4^*J*_2,7_ = −0.5, ^4^*J*_6,7_ = −0.5, ^5^*J*_5,7_ = 0.3, 2 H, CH_2_-7), 6.97 (dtd, ^4^*J*_2,6_ = 1.8, ^4^*J*_2,7_ = −0.5, ^5^*J*_2,8_ = 0.4, 1 H, CH-2), 7.19 (ddtd, ^3^*J*_5,6_ = 8.0, ^4^*J*_2,6_ = 1.8, ^4^*J*_6,7_ = −0.5, ^5^*J*_6,8_ = 0.4, 1 H, CH-6), 7.30 (ddt, ^3^*J*_5,6_ = 8.0, ^4^*J*_5,8_ = −0.6, ^5^*J*_5,7_ = 0.3, 1 H, CH-5); ^13^C NMR (CDCl_3_) δ 11.8 (C-4′), 16.7 (C-5′), 22.6 (C-4″), 23.1 (C-9 and C-10), 25.9 (C-3″), 26.9 (C-3′), 27.4 (C-8), 41.2 (C-2′), 43.5 (C-2″), 65.4 (C-7), 122.1 (C-2), 126.0 (C-6), 127.0 (C-5), 135.0 (C-1), 140.2 (C-4), 148.2 (C-3), 171.8 (C-1″), 176.7 (C-1′).

*7-Isovaleroxythymyl 2-methylbutyrate (syn. 4-isopropyl-3-(2-methylbutyryloxy)benzyl isovalerate, **7-iBu-3-sBu**):* It was prepared from 7-isovaleroxythymol (20 mg, 0.08 mmol), 2-methylbutyric acid (9 mg, 0.09 mmol), DCC (18.5 mg, 0.09 mmol), and DMAP (1.3 mg, 0.01 mmol) in 10 mL of CH_2_Cl_2_. Column chromatographic purification with *n*-hexane/diethyl ether (19:1, *v*/*v*) yielded 7-isovaleroxythymyl 2-methylbutyrate 23 mg (86%); colorless liquid; RI = 2147 (DB-5MS column); FTIR (neat; cm^−1^) 2964, 2875, 1757, 1734, 1576, 1507, 1463, 1422, 1384, 1364, 1284, 1236, 1178, 1147, 1089, 1059, 1013, 971, 915, 888, 824, 734; MS (EI), *m*/*z* (%) 250 (15), 232 (6), 166 (35), 149 (8), 133 (5), 85 (32), 84 (6), 57 (100), 43 (7), 41 (20); HRMS, *m*/*z* 334.2141 [M]^+^, calculated for C_20_H_30_O_4_^•+^, 334.2139; analyzed C 71.86, H 9.01%, calculated for C_20_H_30_O_4_, C 71.82, H 9.04, O 19.13%; ^1^H NMR (CDCl_3_) δ 0.95 (d, ^3^*J*_3′,4′_ = 6.7, 6 H, CH_3_-4′), 1.04 (ddd, ^3^*J*_3″A,4″_ = 7.5, ^3^*J*_3″B,4″_ = 7.5, ^4^*J*_2″,4″_ = 0.3, 3 H, CH_3_-4″), 1.20 (d, ^3^*J*_8,9/10_ = 6.9, 6 H, CH_3_-9 and CH_3_-10), 1.33 (d, ^3^*J*_2″,5″_ = 7.0, 3 H, CH_3_-5″), 1.63 (dqd, ^2^*J*_3″A,3″B_ = −13.7, ^3^*J*_3″B,4″_ = 7.5, ^3^*J*_2″,3″B_ = 6.5, 1 H, CH-3″B), 1.87 (ddq, ^2^*J*_3″A,3″B_ = −13.7, ^3^*J*_2″,3″A_ = 7.5, ^3^*J*_3″A,4″_ = 7.5, 1 H, CH-3″A), 2.12 (tsept, ^3^*J*_2′,3′_ = 7.2, ^3^*J*_3′,4′_ = 6.7, 1 H, CH-3′), 2.23 (d, ^3^*J*_2′,3′_ = 7.2, 2 H, CH_2_-2′), 2.66 (dqdq, ^3^*J*_2″,3″A_ = 7.5, ^3^*J*_2″,5″_ = 7.0, ^3^*J*_2″,3″B_ = 6.5, ^4^*J*_2″,4″_ = 0.3, 1 H, CH-2″), 3.02 (septddd, ^3^*J*_8,9/10_ = 6.9, ^4^*J*_5,8_ = −0.6, ^5^*J*_6,8_ = 0.4, ^5^*J*_2,8_ = 0.3, 1 H, CH-8), 5.07 (ddd, ^4^*J*_6,7_ = −0.6, ^4^*J*_2,7_ = −0.5, ^5^*J*_5,7_ = 0.3, 2 H, CH_2_-7), 6.96 (dtd, ^4^*J*_2,6_ = 1.8, ^4^*J*_2,7_ = −0.5, ^5^*J*_2,8_ = 0.3, 1 H, CH-2), 7.19 (ddtd, ^3^*J*_5,6_ = 8.0, ^4^*J*_2,6_ = 1.8, ^4^*J*_6,7_ = −0.6, ^5^*J*_6,8_ = 0.4, 1 H, CH-6), 7.30 (ddt, ^3^*J*_5,6_ = 8.0, ^4^*J*_5,8_ = −0.6, ^5^*J*_5,7_ = 0.3, 1 H, CH-5); ^13^C NMR (CDCl_3_) δ 11.9 (C-4″), 16.8 (C-5″), 22.6 (C-4′), 23.0 (C-9 and C-10), 25.9 (C-3′), 26.9 (C-3″), 27.3 (C-8), 41.5 (C-2″), 43.5 (C-2′), 65.5 (C-7), 122.2 (C-2), 126.1 (C-6), 126.9 (C-5), 134.9 (C-1), 140.3 (C-4), 148.3 (C-3), 173.1 (C-1′), 175.3 (C-1″).

### 3.7. Synthesis of 8-Acyloxythymyl Esters

#### 3.7.1. Synthesis of 8-Hydroxythymol

8-Hydroxythymol was synthesized via a Grignard reaction of 2-hydroxy-4-methylacetophenone with methylmagnesium iodide [23]. Magnesium turnings (1.2 g, 50 mmol) and a crystal of iodine were suspended in anhydrous diethyl ether (40 mL), and a solution of methyl iodide (7.2 g, 50 mmol) in anhydrous diethyl ether (40 mL) was added dropwise, maintaining gentle reflux. After complete addition, the reaction mixture was refluxed for an additional 30 min. The mixture was then cooled to 0 °C, and a solution of 2-hydroxy-4-methylacetophenone (750 mg, 5 mmol) in diethyl ether (20 mL) was added dropwise over 15 min. The reaction mixture was subsequently refluxed for 30 min. After cooling to 0 °C, saturated aqueous NH_4_Cl solution was added dropwise to quench the reaction. The mixture was extracted with diethyl ether (3 × 20 mL), and the combined organic layers were dried with anhydrous MgSO_4_. After filtration, the solvent was removed under reduced pressure to afford the 700 mg (84%) of 8-hydroxythymol as white crystals.

*8-Hydroxythymol (syn. 2-(2-hydroxypropan-2-yl)-5-methylphenol):* white crystals; RI = 1410 (DB-5MS); MS (EI), *m*/*z* (%) 148 (78), 133 (70), 105 (100), 91 (24), 79 (19), 78 (18), 77 (43), 63 (16), 51 (19), 43 (32); analyzed C 72.22, H 8.52%, calculated for C_10_H_14_O_2_, C 72.26, H 8.49, O 19.25%; ^1^H NMR (CDCl_3_) δ 1.63 (s, 6 H, CH_3_-9 and CH_3_-10), 2.26 (ddd, ^4^*J*_2,7_ = −0.7, ^4^*J*_6,7_ = −0.7, ^5^*J*_5,7_ = 0.3, 3 H, CH_3_-7), 2.77 (brs, 1 H, alcoholic OH), 6.63 (ddq, ^3^*J*_5,6_ = 7.9, ^4^*J*_2,6_ = 1.8, ^4^*J*_6,7_ = −0.7, 1 H, CH-6), 6.66 (dqd, ^4^*J*_2,6_ = 1.8, ^4^*J*_2,7_ = −0.7, ^5^*J*_2,5_ = 0.3, 1 H, CH-2), 6.94 (ddq, ^3^*J*_5,6_ = 7,9, ^5^*J*_2,5_ = 0.3, ^5^*J*_5,7_ = 0.3, 1 H, CH-5), 8.96 (brs, 1 H, phenolic OH); ^13^C NMR (CDCl_3_) δ 21.0 (C-7), 30.4 (C-9 and C-10), 76.0 (C-8), 118.1 (C-2), 120.5 (C-6), 125.3 (C-5), 128.3 (C-4), 139.1 (C-1), 155.4 (C-3).

#### 3.7.2. Acylation of 8-Hydroxythymol

Diesters of 8-hydroxythymol with isobutyric, 2-methylbutyric, and isovaleric acids were synthesized by acylation of 8-hydroxythymol using the corresponding acid chlorides in the presence of sodium hydride.

*8-Isobutyryloxythymyl isobutyrate:* It was prepared by suspending NaH (1.0 g of a 60% dispersion in mineral oil) in dry THF (20 mL) under cooling at 0 °C. A solution of 8-hydroxythymol (200 mg, 1.2 mmol) in dry THF (5 mL) was added dropwise, and the reaction mixture was stirred magnetically at room temperature for 30 min. The reaction mixture was cooled again to 0 °C, and a solution of isobutyryl chloride (645 mg, 6 mmol) in THF (5 mL) was added dropwise. Stirring was continued for an additional 30 min at room temperature. The reaction was quenched by addition of saturated aqueous NaCl solution, and the mixture was extracted with diethyl ether (3 × 20 mL). The combined organic layers were washed with saturated aqueous NaCl solution, dried with anhydrous Na_2_SO_4_, filtered, and concentrated under reduced pressure to afford a crude residue (240 mg). Purification by column chromatography on silica gel using n-hexane/diethyl ether (9:1, *v*/*v*) as the eluent afforded 8-isobutyryloxythymyl isobutyrate (20 mg, 5%).

*8-Isobutyryloxythymyl isobutyrate (syn. 2-(2-isobutyryloxy-4-methylphenyl)propan-2-yl isobutyrate, **8-iPr-3-iPr**):* colorless liquid; RI = 1894 (DB-5MS); MS (EI), *m*/*z* (%) 218 (6), 149 (16), 148 (100), 133 (16), 105 (10), 91 (7), 77 (7), 71 (15), 43 (62), 41 (18); HRMS, *m*/*z* 306.1822 [M]^+^, calculated for C_18_H_26_O_4_^•+^, 306.1826; analyzed C 70.59, H 8.53%, calculated for C_18_H_26_O_4_, C 70.56, H 8.55, O 20.89%; ^1^H NMR (CDCl_3_) δ 1.12 (d, ^3^ *J*_2′,3′_ = 7.0, 6 H, CH_3_-3′), 1.34 (d, ^3^ *J*_2″,3″_ = 7.0, 6 H, CH_3_-3″), 1.76 (s, 6 H, CH_3_-9 and CH_3_-10), 2.30 (ddd, ^4^*J*_6,7_ = −0.7, ^4^*J*_2,7_ = −0.7, ^5^*J*_5,7_ = 0.3, 3 H, CH_3_-7), 2.43 (sept, ^3^ *J*_2′,3′_ = 7.0, 1 H, CH-2′), 2.80 (sept, ^3^ *J*_2″,3″_ = 7.0, 1 H, CH-2″), 6.74 (dqd, ^4^*J*_2,6_ = 1.8, ^4^*J*_2,7_ = −0.7, ^5^*J*_2,5_ = 0.3, 1 H, CH-2), 7.00 (ddq, ^3^*J*_5,6_ = 8.1, ^4^*J*_2,6_ = 1.8, ^4^*J*_6,7_ = −0.7, 1 H, CH-6), 7.31 (ddq, ^3^*J*_5,6_ = 8.1, ^5^*J*_2,5_ = 0.3, ^5^*J*_5,7_ = 0.3, 1 H, CH-5); ^13^C NMR (CDCl_3_) δ 19.02^a^ (C-3″), 19.06^a^ (C-3′), 20.9 (C-7), 27.8 (C-9 and C-10), 34.5 (C-2″), 34.6 (C-2′), 79.9 (C-8), 124.6 (C-2), 126.7 (C-6), 126.8 (C-5), 133.4 (C-4), 138.6 (C-1), 147.8 (C-3), 175.5 (C-1″), 175.8 (C-1′). ^a^ The signals of C-3′ and C-3″ may be interchanged.

*8-(2-Methylbutyryloxy)thymyl 2-methylbutyrate (syn. 2-(2-(2-methylbutyryloxy)-4-methylphenyl)propan-2-yl 2-methylbutyrate, **8-sBu-3-sBu**):* It was prepared analogously to the procedure described above for 8-isobutyryloxythymyl isobutyrate, using 8-hydroxythymol (50 mg, 0.3 mmol), 2-methylbutyryl chloride (182 mg, 1.5 mmol), and NaH (250 mg of a 60% dispersion in mineral oil) in dry THF. The reaction afforded 60 mg of a crude product mixture, which was analyzed directly by GC–MS without further purification; RI = 2079 (DB-5MS column); MS (EI), *m*/*z* (%) 149 (29), 148 (100), 133 (20), 121 (7), 105 (17), 91 (8), 85 (11), 77 (6), 57 (41), 41 (18).

*8-Isovaleroxythymyl isovalerate (syn. 2-(2-isovaleroxy-4-methylphenyl)propan-2-yl isovalerate, **8-iBu-3-iBu**):* It was prepared analogously to the procedure described above for 8-isobutyryloxythymyl isobutyrate, using 8-hydroxythymol (50 mg, 0.3 mmol), isovaleryl chloride (182 mg, 1.5 mmol), and NaH (250 mg of a 60% dispersion in mineral oil) in dry THF. The reaction afforded 65 mg of a crude product mixture, which was analyzed directly by GC–MS without further purification; RI = 2068 (DB-5MS column); MS (EI), *m*/*z* (%) 149 (24), 148 (100), 133 (19), 121 (6), 105 (14), 91 (6), 85 (11), 57 (20), 43 (7), 41 (14).

*8-(2-Methylbutyryloxy)thymyl isobutyrate and 8-isobutyryloxythymyl 2-methylbutyrate:* These were prepared analogously to the procedure described above for 8-isobutyryloxythymyl isobutyrate, using 8-hydroxythymol (50 mg, 0.3 mmol), a mixture of isobutyryl chloride (0.75 mmol) and 2-methylbutyryl chloride (0.75 mmol), and NaH (250 mg of a 60% dispersion in mineral oil) in dry THF. The reaction afforded 50 mg of a crude product mixture, which was analyzed directly by GC–MS without further purification.

*8-(2-Methylbutyryloxy)thymyl isobutyrate (syn. 2-(2-isobutyryloxy-4-methylphenyl)propan-2-yl 2-methylbutyrate, **8-sBu-3-iPr**):* RI = 1987 (DB-5MS column); MS (EI), *m*/*z* (%) 218 (5), 149 (16), 148 (100), 133 (20), 105 (10), 78 (6), 77 (8), 71 (15), 43 (47), 41 (17).

*8-Isobutyryloxythymyl 2-methylbutyrate (syn. 2-(2-isobutyryloxypropan-2-yl)-5-methylphenyl 2-methylbutyrate, **8-iPr-3-sBu**):* RI = 1987 (DB-5MS column); MS (EI), *m*/*z* (%) 149 (14), 148 (100), 133 (14), 105 (10), 91 (8), 85 (8), 77 (8), 57 (71), 43 (8), 41 (21).

*8-Isovaleroxythymyl isobutyrate and 8-isobutyryloxythymyl isovalerate:* These were prepared analogously to the procedure described above for 8-isobutyryloxythymyl isobutyrate, using 8-hydroxythymol (50 mg, 0.3 mmol), mixture of isobutyryl chloride (0.75 mmol) and isovaleryl chloride (0.75 mmol), and NaH (250 mg of a 60% dispersion in mineral oil) in dry THF. The reaction afforded 55 mg of a crude product mixture, which was analyzed directly by GC–MS without further purification.

*8-Isovaleroxythymyl isobutyrate (syn. 2-(2-isobutyryloxy-4-methylphenyl)propan-2-yl isovalerate, **8-iBu-3-iPr**):* RI = 1980 (DB-5MS column); MS (EI), *m*/*z* (%) 149 (15), 148 (100), 133 (20), 115 (6), 105 (11), 91 (10), 77 (9), 71 (20), 43 (61), 41 (17).

*8-Isobutyryloxythymyl isovalerate (syn. 2-(2-isobutyryloxypropan-2-yl)-5-methylphenyl isovalerate, **8-iPr-3-iBu**):* RI = 1974 (DB-5MS column); MS (EI), *m*/*z* (%) 149 (15), 148 (100), 133 (15), 105 (8), 91 (8), 85 (8), 77 (7), 57 (26), 43 (13), 41 (17).

### 3.8. Synthesis of 9-Acyloxythymyl Esters

#### 3.8.1. Synthesis of 4,7-Dimethylcoumarin

A mixture of *m*-cresol (6.0 g, 55.6 mmol) and methyl acetoacetate (10.76 g, 92.8 mmol) was treated with 80% H_2_SO_4_ (54 mL), and the resulting mixture was stirred at room temperature for 30 min [25]. The reaction mixture was then poured onto crushed ice, resulting in the formation of a white precipitate. The solid product was collected by filtration, washed with cold water, and dried to afford 4,7-dimethylcoumarin (10.4 g, quantitative yield).

*4,7-dimethylcoumarin (syn. 4,7-dimethyl-2H-chromen-2-one):* white solid; RI = 1725 (DB-5MS); MS (EI), *m*/*z* (%) 174 (77), 147 (10), 146 (97), 145 (100), 131 (24), 117 (22), 115 (27), 91 (16), 77 (13), 51 (11); The obtained NMR data were in full agreement with the literature values reported by Francisco et al. [25].

#### 3.8.2. Synthesis of 8,9-Dehydrothymol

8,9-Dehydrothymol was synthesized according to the procedure described by Divakar et al. [26], with minor modifications. A mixture of 4,7-dimethylcoumarin (10.0 g, 57.5 mmol), NaOH (9.2 g, 0.23 mol), and ethylene glycol (50 mL) was heated under reflux under a nitrogen atmosphere for 2 h. After cooling to room temperature, the reaction mixture was diluted with water and acidified with dilute HCl to pH 2. The product was extracted with *n*-pentane, and the combined organic layers were washed with water, dried over anhydrous Na_2_SO_4_, filtered, and concentrated under reduced pressure. The crude product was purified by dry-flash chromatography on silica gel using *n*-hexane/diethyl ether (98:2, *v*/*v*) as the eluent to afford pure 8,9-dehydrothymol (2.4 g, 28%).

*8,9-Dehydrothymol (syn. 5-methyl-2-(prop-1-en-2-yl)phenol):* colorless liquid; RI = 1221 (DB-5MS); MS (EI), *m*/*z* (%) 148 (85), 133 (55), 115 (13), 105 (100), 103 (15), 91 (20), 79 (15), 78 (12), 77 (28), 51 (12); The obtained NMR data were in full agreement with the literature values reported by Divakar et al. [26].

#### 3.8.3. Synthesis of 9-Hydroxythymol

A dry three-necked flask equipped with a magnetic stirrer, internal thermometer, dropping funnel, and argon inlet was charged with dry THF (20 mL) and finely powdered NaBH_4_ (700 mg, 18.4 mmol) [3]. The mixture was stirred until a homogeneous suspension was obtained and cooled to −5 °C using an ice–salt bath. A solution of iodine (1.7 g, 6.7 mmol) in dry THF (20 mL) was added dropwise under continuous stirring. Subsequently, a solution of 8,9-dehydrothymol (2.0 g, 13.5 mmol) in dry THF (20 mL) was added dropwise over 10 min while maintaining the reaction temperature below 30 °C. The reaction mixture was stirred at 25 °C for 2 h and then cooled to 0 °C. Water (20 mL), THF (20 mL), and 3 M aqueous NaOH solution (50 mL) were added successively, followed by dropwise addition of 30% (*w*/*w*) H_2_O_2_ (918 mg, 27 mmol, 3.06 mL). Stirring was continued for an additional 30 min after complete addition of hydrogen peroxide. The reaction mixture was transferred to a separatory funnel, and the reaction vessel was rinsed with diethyl ether (30 mL). The aqueous phase was saturated with NaCl and extracted with diethyl ether (3 × 30 mL). The combined organic layers were washed with saturated aqueous NaCl solution (2 × 30 mL), dried with anhydrous MgSO_4_, filtered, and concentrated under reduced pressure. The crude product was purified by column chromatography on silica gel using *n*-hexane/diethyl ether (1:1, *v*/*v*) as the eluent to afford 9-hydroxythymol (300 mg, 13%).

*9-Hydroxythymol (syn. 2-(1-hydroxypropan-2-yl)-5-methylphenol):* colorless viscous oil; RI = 1562 (DB-5MS); FTIR (neat; cm^−1^) 3341, 2962, 1619, 1575, 1505, 1419, 1379, 1288, 1248, 1159, 1127, 1013, 946, 860, 807, 737; MS (EI), *m*/*z* (%) 166 (21), 148 (22), 136 (10), 135 (100), 133 (22), 115 (21), 105 (18), 91 (29), 77 (15), 39 (10); analyzed C 72.24, H 8.50%, calculated for C_10_H_14_O_2_, C 72.26, H 8.49, O 19.25%; ^1^H NMR (CDCl_3_) δ 1.28 (d, ^3^*J*_8,10_ = 7.3, 3 H, CH_3_-10), 2.27 (ddd, ^4^*J*_2,7_ = −0.7, ^4^*J*_6,7_ = −0.7, ^5^*J*_5,7_ = 0.3, 3 H, CH_3_-7), 2.91 (brs, 1 H, alcoholic OH), 3.19 (dqdddd, ^3^*J*_8,9B_ = 7.8, ^3^*J*_8,10_ = 7.3, ^3^*J*_8,9A_ = 3.8, ^4^*J*_5,8_ = −0.5, ^5^*J*_2,8_ = 0.3, ^5^*J*_6,8_ = 0.3, 1 H, CH-8), 3.68 (dd, ^2^ *J*_9A,9B_ = −9.8, ^3^ *J*_8,9B_ = 7.8, 1 H, CH-9B), 3.88 (dd, ^2^ *J*_9A,9B_ = −9.8, ^3^ *J*_8,9A_ = 3.8, 1 H, CH-9A), 6.70 (dqd, ^4^*J*_2,6_ = 1.8, ^4^*J*_2,7_ = −0.7, ^5^*J*_2,8_ = 0.3, 1 H, CH-2), 6.71 (ddqd, ^3^*J*_5,6_ = 7.8, ^4^*J*_2,6_ = 1.8, ^4^*J*_6,7_ = −0.7, ^5^*J*_6,8_ = 0.3, 1 H, CH-6), 6.99 (ddq, ^3^*J*_5,6_ = 7.8, ^4^*J*_5,8_ = −0.5, ^5^*J*_5,7_ = 0.3, 1 H, CH-5), 7.78 (brs, 1 H, phenolic OH); ^13^C NMR (CDCl_3_) δ 15.9 (C-10), 21.0 (C-7), 36.7 (C-8), 69.5 (C-9), 117.8 (C-2), 121.5 (C-6), 127.7 (C-4), 127.8 (C-5), 137.9 (C-1), 154.7 (C-3).

#### 3.8.4. Esterification of 9-Hydroxythymol with Corresponding Carboxylic Acids

Mono- and diesters of 9-hydroxythymol with isobutyric, 2-methylbutyric, and isovaleric acids were synthesized following a standard Steglich esterification protocol as described above in Section 3.3.3.

*9-Isobutyryloxythymol and 9-isobutyryloxythymyl isobutyrate:* These were prepared from 9-hydroxythymol (50 mg, 0.3 mmol), isobutyric acid (40 mg, 0.45 mmol), DCC (93 mg, 0.45 mmol), and DMAP (5 mg, 0.04 mmol) in 30 mL of CH_2_Cl_2_. Column chromatographic purification with *n*-hexane/diethyl ether (19:1, and then 4:1, *v*/*v*) yielded 9-isobutyryloxythymyl isobutyrate 25 mg (27%) and 9-isobutyryloxythymol 32 mg (47%).

*9-Isobutyryloxythymol (syn. 2-(2-hydroxy-4-methylphenyl)propyl isobutyrate):* yellowish liquid; RI = 1768 (DB-5MS); FTIR (neat; cm^−1^) 3407, 2974, 2927, 1707, 1621, 1579, 1519, 1460, 1420, 1366, 1291, 1204, 1161, 1080, 1034, 979, 945, 858, 806, 735; MS (EI), *m*/*z* (%) 149 (13), 148 (100), 135 (87), 133 (70), 115 (14), 105 (18), 91 (21), 71 (14), 43 (46), 41 (15); HRMS, *m*/*z* 236.1386 [M]^+^, calculated for C_14_H_20_O_3_^•+^, 236.1407; analyzed C 71.19, H 8.51%, calculated for C_14_H_20_O_3_, C 71.16, H 8.53, O 20.31%; ^1^H NMR (CDCl_3_) δ 1.16 (d, ^3^*J*_2′,3′_ = 7.0, 6 H, CH_3_-3′), 1.34 (d, ^3^*J*_8,10_ = 7.0, 3 H, CH_3_-10), 2.28 (ddd, ^4^*J*_2,7_ = −0.7, ^4^*J*_6,7_ = −0.7, ^5^*J*_5,7_ = 0.4, 3 H, CH_3_-7), 2.57 (sept, ^3^*J*_2′,3′_ = 7.0, 1 H, CH-2′), 3.32 (dqdddd, ^3^*J*_8,9B_ = 8.0, ^3^*J*_8,10_ = 7.0, ^3^*J*_8,9A_ = 5.1, ^4^*J*_5,8_ = −0.7, ^5^*J*_2,8_ = 0.3, ^5^*J*_6,8_ = 0.3, 1 H, CH-8), 3.92 (dd, ^2^ *J*_9A,9B_ = −10.9, ^3^*J*_8,9B_ = 8.0, 1 H, CH-9B), 4.29 (dd, ^2^ *J*_9A,9B_ = −10.9, ^3^ *J*_8,9A_ = 5.1, 1 H, CH-9A), 5.97 (brs, 1 H, OH), 6.67 (dqd, ^4^*J*_2,6_ = 1.8, ^4^*J*_2,7_ = −0.7, ^5^*J*_2,8_ = 0.3, 1 H, CH-2), 6.72 (ddqd, ^3^*J*_5,6_ = 7.8, ^4^*J*_2,6_ = 1.8, ^4^*J*_6,7_ = −0.7, ^5^*J*_6,8_ = 0.3, 1 H, CH-6), 7.03 (ddq, ^3^*J*_5,6_ = 7.8, ^4^*J*_5,8_ = −0.7, ^5^*J*_5,7_ = 0.4, 1 H, CH-5); ^13^C NMR (CDCl_3_) δ 16.5 (C-10), 19.10/19.02 (C-3′), 21.1 (C-7), 32.4 (C-8), 34.2 (C-2′), 69.7 (C-9), 116.9 (C-2), 121.5 (C-6), 125.1 (C-4), 127.1 (C-5), 138.0 (C-1), 154.0 (C-3), 178.2 (C-1′).

*9-Isobutyryloxythymyl isobutyrate (syn. 2-(2-(isobutyryloxy)-4-methylphenyl)propyl isobutyrate, **9-iPr-3-iPr**):* colorless liquid; RI = 1921 (DB-5MS column); UV (CH_3_CN) λ_max_(log ε) 264 (2.52), 212 (3.79), 193 (4.54) nm; FTIR (neat; cm^−1^) 2974, 2937, 2878, 1756, 1733, 1622, 1577, 1508, 1470, 1414, 1388, 1371, 1344, 1229, 1188, 1142, 1127, 1095, 1047, 989, 946, 930, 909, 879, 841, 816, 753; MS (EI), *m*/*z* (%) 218 (9), 149 (14), 148 (100), 135 (26), 133 (24), 105 (5), 91 (8), 71 (26), 43 (49), 41 (9); HRMS, *m*/*z* 306.1830 [M]^+^, calculated for C_18_H_26_O_4_^•+^, 306.1826; analyzed C 70.51, H 8.58%, calculated for C_18_H_26_O_4_, C 70.56, H 8.55, O 20.89%; ^1^H NMR (CDCl_3_) δ 1.1134/1.1027 (d, ^3^*J*_2′,3′_ = 7.0, 6 H, CH_3_-3′), 1.23 (d, ^3^*J*_8,10_ = 7.0, 3 H, CH_3_-10), 1.3368/1.3333 (d, ^3^*J*_2″,3″_ = 7.0, 6 H, CH_3_-3″), 2.32 (ddd, ^4^*J*_2,7_ = −0.7, ^4^*J*_6,7_ = −0.7, ^5^*J*_5,7_ = 0.3, 3 H, CH_3_-7), 2.50 (sept, ^3^*J*_2′,3′_ = 7.0, 1 H, CH-2′), 2.84 (sept, ^3^*J*_2″,3″_ = 7.0, 1 H, CH-2″), 3.20 (dqdddd, ^3^*J*_8,9B_ = 7.4, ^3^*J*_8,10_ = 7.0, ^3^*J*_8,9A_ = 6.6, ^4^*J*_5,8_ = −0.5, ^5^*J*_6,8_ = 0.4, ^5^*J*_2,8_ = 0.3, 1 H, CH-8), 4.08 (dd, ^2^ *J*_9A,9B_ = −10.7, ^3^ *J*_8,9B_ = 7.4, 1 H, CH-9B), 4.15 (dd, ^2^ *J*_9A,9B_ = −10.7, ^3^ *J*_8,9A_ = 6.6, 1 H, CH-9A), 6.82 (dqd, ^4^*J*_2,6_ = 1.8, ^4^*J*_2,7_ = −0.7, ^5^*J*_2,8_ = 0.3, 1 H, CH-2), 7.02 (ddqd, ^3^*J*_5,6_ = 7.9, ^4^*J*_2,6_ = 1.8, ^4^*J*_6,7_ = −0.7, ^5^*J*_6,8_ = 0.4, 1 H, CH-6), 7.18 (ddq, ^3^*J*_5,6_ = 7.9, ^4^*J*_5,8_ = −0.5, ^5^*J*_5,7_ = 0.3, 1 H, CH-5); ^13^C NMR (CDCl_3_) δ 17.4 (C-10), 19.06/19.05 (C-3′), 19.15/19.10 (C-3″), 21.0 (C-7), 32.0 (C-8), 34.1 (C-2′), 34.4 (C-2″), 68.3 (C-9), 123.0 (C-2), 127.1 (C-6), 127.3 (C-5), 131.8 (C-4), 137.6 (C-1), 148.7 (C-3), 175.8 (C-1″), 177.1 (C-1′).

*9-(2-Methylbutyryloxy)thymol and 9-(2-methylbutyryoxy)thymyl 2-methylbutyrate:* These were prepared from 9-hydroxythymol (50 mg, 0.3 mmol), 2-methylbutyric acid (46 mg, 0.45 mmol), DCC (93 mg, 0.45 mmol), and DMAP (5 mg, 0.04 mmol) in 30 mL of CH_2_Cl_2_. Column chromatographic purification with *n*-hexane/diethyl ether (19:1, and then 4:1, *v*/*v*) yielded 9-(2-methylbutyryoxy)thymyl 2-methylbutyrate 22 mg (22%) and 9-(2-methylbutyryloxy)thymol 33 mg (44%).

*9-(2-Methylbutyryloxy)thymol (syn. 2-(2-hydroxy-4-methylphenyl)propyl 2-methylbutyrate):* yellowish liquid; RI = 1863 (DB-5MS column); FTIR (neat; cm^−1^) 3421, 2968, 2936, 2878, 1732, 1705, 1620, 1587, 1520, 1461, 1421, 1376, 1291, 1263, 1191, 1155, 1088, 1030, 1013, 977, 947, 859, 806, 735; MS (EI), *m*/*z* (%) 148 (100), 135 (85), 133 (68), 121 (21), 115 (21), 105 (20), 91 (37), 77 (16), 41 (21); HRMS, *m*/*z* 250.1551 [M]^+^, calculated for C_15_H_22_O_3_^•+^, 250.1563; analyzed C 71.94, H 8.88%, calculated for C_15_H_22_O_3_, C 71.97, H 8.86, O 19.17%; ^1^H NMR (CDCl_3_) 0.8868/0.8816 (ddd, ^3^*J*_3′A,4′_ = 7.5, ^3^*J*_3′B,4′_ = 7.5, ^4^*J*_2′,4′_ = 0.3, 3 H, CH_3_-4′), 1.1382 (d, ^3^*J*_2′,5′_ = 7.0, 3 H, CH_3_-5′), 1.3400 (d, ^3^*J*_8,10_ = 7.0, 3 H, CH_3_-10), 1.4770/1.4697 (dqd, ^2^*J*_3′A,3′B_ = −13.7, ^3^*J*_3′B,4′_ = 7.5, ^3^*J*_2′,3′B_ = 6.4, 1 H, CH-3′B), 1.6725/1.6689 (dqd, ^2^*J*_3′A,3′B_ = −13.7, ^3^*J*_3′A,4′_ = 7.5, ^3^*J*_2′,3′A_ = 7.4, 1 H, CH-3′A), 2.28 (ddd, ^4^*J*_2,7_ = −0.7, ^4^*J*_6,7_ = −0.7, ^5^*J*_5,7_ = 0.3, 3 H, CH_3_-7), 2.3960/2.3925 (dqdq, ^3^*J*_2′,3′A_ = 7.4, ^3^*J*_2′,5′_ = 7.0, ^3^*J*_2′,3′B_ = 6.4, ^4^*J*_2′,4′_ = 0.3, 1 H, CH-2′), 3.32 (dqdddd, ^3^*J*_8,9B_ = 8.0, ^3^*J*_8,10_ = 7.0, ^3^*J*_8,9A_ = 5.0, ^4^*J*_5,8_ = −0.7, ^5^*J*_2,8_ = 0.3, ^5^*J*_6,8_ = 0.3, 1 H, CH-8), 3.9182/3.9075 (dd, ^2^ *J*_9A,9B_ = −10.9, ^3^ *J*_8,9B_ = 8.0, 1 H, CH-9B), 4.3120/4.3018 (dd, ^2^ *J*_9A,9B_ = −10.9, ^3^ *J*_8,9A_ = 5.0, 1 H, CH-9A), 6.0828/6.0590 (brs, 1 H, OH), 6.67 (dqd, ^4^*J*_2,6_ = 1.8, ^4^*J*_2,7_ = −0.7, ^5^*J*_2,8_ = 0.3, 1 H, CH-2), 6.71 (ddqd, ^3^*J*_5,6_ = 7.8, ^4^*J*_2,6_ = 1.8, ^4^*J*_6,7_ = −0.7, ^5^*J*_6,8_ = 0.3, 1 H, CH-6), 7.03 (ddq, ^3^*J*_5,6_ = 7.8, ^4^*J*_5,8_ = −0.7, ^5^*J*_5,7_ = 0.3, 1 H, CH-5); ^13^C NMR (CDCl_3_) δ 11.74/11.68 (C-4′), 16.53/16.50 (C-10), 16.76/16.64 (C-5′), 21.1 (C-7), 26.93/26.83 (C-3′), 32.42/32.40 (C-8), 41.32/41.24 (C-2′), 69.67/69.61 (C-9), 116.93/116.91 (C-2), 121.48/121.46 (C-6), 125.12/125.08 (C-4), 127.04/127.02 (C-5), 138.0 (C-1), 154.09/154.07 (C-3), 177.91/177.83 (C-1′).

*9-(2-Methylbutyryoxy)thymyl 2-methylbutyrate (syn. 2-(4-methyl-2-(2-methylbutyryloxy)phenyl)propyl 2-methylbutyrate, **9-sBu-3-sBu**):* colorless liquid; RI = 2095 (DB-5MS column); FTIR (neat; cm^−1^) 2969, 2936, 2878, 1755, 1733, 1622, 1507, 1461, 1381, 1242, 1217, 1179, 1143, 1122, 1089, 1012, 982, 900, 815, 750; MS (EI), *m*/*z* (%) 232 (7), 149 (15), 148 (100), 136 (3), 135 (20), 133 (17), 121 (4), 85 (16), 58 (3), 57 (47); HRMS, *m*/*z* 334.2143 [M]^+^, calculated for C_20_H_30_O_4_^•+^, 334.2139; analyzed C 71.80, H 9.07%, calculated for C_20_H_30_O_4_, C 71.82, H 9.04, O 19.13%; ^1^H NMR (CDCl_3_) 0.8483/0.8315 (ddd, ^3^*J*_3′A,4′_ = 7.5, ^3^*J*_3′B,4′_ = 7.5, ^4^*J*_2′,4′_ = 0.3, 3 H, CH_3_-4′), 1.03 (ddd, ^3^*J*_3″A,4″_ = 7.5, ^3^*J*_3″B,4″_ = 7.5, ^4^*J*_2″,4″_ = 0.3, 3 H, CH_3_-4″), 1.0890/1.0793 (d, ^3^*J*_2′,5′_ = 7.0, 3 H, CH_3_-5′), 1.24 (d, ^3^*J*_8,10_ = 7.0, 3 H, CH_3_-10), 1.3163/1.3147/1.3127 (d, ^3^*J*_2″,5″_ = 7.0, 3 H, CH_3_-5″), 1.4205/1.4200/1.4154/1.4152 (dqd, ^2^*J*_3′A,3′B_ = −13.7, ^3^*J*_3′B,4′_ = 7.5, ^3^*J*_2′,3′B_ = 6.4, 1 H, CH-3′B), 1.6192/1.6105 (dqd, ^2^*J*_3′A,3′B_ = −13.7, ^3^*J*_3′A,4′_ = 7.5, ^3^*J*_2′,3′A_ = 7.4, 1 H, CH-3′A), 1.6275/1.6265/1.6260/1.6240 (dqd, ^2^*J*_3″A,3″B_ = −13.7, ^3^*J*_3″B,4″_ = 7.5, ^3^*J*_2″,3″B_ = 6.4, 1 H, CH-3″B), 1.8635/1.8627/1.8617/1.8609 (dqd, ^2^*J*_3″A,3″B_ = −13.7, ^3^*J*_3″A,4″_ = 7.5, ^3^*J*_2″,3″A_ = 7.4, 1 H, CH-3″A), 2.32 (ddd, ^4^*J*_2,7_ = −0.7, ^4^*J*_6,7_ = −0.7, ^5^*J*_5,7_ = 0.3, 3 H, CH_3_-7), 2.3270/2.3250 (dqdq, ^3^*J*_2′,3′A_ = 7.4, ^3^*J*_2′,5′_ = 7.0, ^3^*J*_2′,3′B_ = 6.4, ^4^*J*_2′,4′_ = 0.3, 1 H, CH-2′), 2.6596/2.6584 (dqdq, ^3^*J*_2″,3″A_ = 7.4, ^3^*J*_2″,5″_ = 7.0, ^3^*J*_2″,3″B_ = 6.4, ^4^*J*_2″,4″_ = 0.3, 1 H, CH-2″), 3.2098/3.2080/3.2056/3.2040 (dqdddd, ^3^*J*_8,9B_ = 7.6, ^3^*J*_8,10_ = 7.0, ^3^*J*_8,9A_ = 6.4, ^4^*J*_5,8_ = −0.5, ^5^*J*_6,8_ = 0.4, ^5^*J*_2,8_ = 0.3, 1 H, CH-8), 4.0828/4.0756/4.0754/4.0685 (dd, ^2^ *J*_9A,9B_ = −10.7, ^3^ *J*_8,9B_ = 7.6, 1 H, CH-9B), 4.1760/4.1734/4.1692/4.1658 (dd, ^2^ *J*_9A,9B_ = −10.7, ^3^ *J*_8,9A_ = 6.4, 1 H, CH-9A), 6.8111/6.8095 (dqd, ^4^*J*_2,6_ = 1.8, ^4^*J*_2,7_ = −0.7, ^5^*J*_2,8_ = 0.3, 1 H, CH-2), 7.0216/7.0215/7.0215 (ddqd, ^3^*J*_5,6_ = 7.9, ^4^*J*_2,6_ = 1.8, ^4^*J*_6,7_ = −0.7, ^5^*J*_6,8_ = 0.4, 1 H, CH-6), 7.18 (ddq, ^3^*J*_5,6_ = 7.9, ^4^*J*_5,8_ = −0.5, ^5^*J*_5,7_ = 0.3, 1 H, CH-5); ^13^C NMR (CDCl_3_) δ 11.72/11.64 (C-4′), 11.9 (C-4″), 16.71/16.69 (C-5′), 16.84/16.79 (C-5″), 17.50/17.48 (C-10), 21.1 (C-7), 26.82^a^ (C-3′), 26.85^a^ (C-3″), 31.98/31.96 (C-8), 41.24/41.16 (C-2′), 41.4 (C-2″), 68.2 (C-9), 123.0 (C-2), 127.1 (C-6), 127.2 (C-5), 131.8 (C-4), 137.6 (C-1), 148.7 (C-3), 175.4 (C-1″), 176.70/176.68 (C-1′). ^a^ The signals of C-3′ and C-3″ may be interchanged.

*9-Isovaleroxythymol and 9-isovaleroxythymyl isovalerate:* These were prepared from 9-hydroxythymol (50 mg, 0.3 mmol), isovaleric acid (46 mg, 0.45 mmol), DCC (93 mg, 0.45 mmol), and DMAP (5 mg, 0.04 mmol) in 30 mL of CH_2_Cl_2_. Column chromatographic purification with *n*-hexane/diethyl ether (19:1, and then 4:1, *v*/*v*) yielded 9-isovaleroxythymyl isovalerate 25 mg (25%) and 9-isovaleroxythymol 35 mg (47%).

*9-Isovaleroxythymol (syn. 2-(2-hydroxy-4-methylphenyl)propyl isovalerate):* colorless liquid; RI = 1863 (DB-5MS column); MS (EI), *m*/*z* (%) 149 (20), 148 (100), 135 (99), 133 (61), 121 (15), 115 (13), 105 (15), 91 (19), 57 (24), 41 (14).

*9-Isovaleroxythymyl isovalerate (syn. 2-(4-methyl-2-isovaleroxyphenyl)propyl isovalerate, **9-iBu-3-iBu**):* colorless liquid; RI = 2098 (DB-5MS column); MS (EI), *m*/*z* (%) 232 (5), 149 (16), 148 (100), 135 (24), 133 (18), 91 (5), 85 (16), 57 (26), 43 (5), 41 (9).

Mixed diesters of 9-hydroxythymol with isobutyric, 2-methylbutyric, and isovaleric acids were synthesized following a standard Steglich esterification protocol as described above in Section 3.3.3.

*9-Isobutyryloxythymyl 2-methylbutyrate (syn. 2-(1-isobutyryloxypropan-2-yl)-5-methylphenyl 2-methylbutyrate, **9-iPr-3-sBu**):* It was prepared from 9-isobutyryloxythymol (20 mg, 0.08 mmol), 2-methylbutyric acid (9 mg, 0.09 mmol), DCC (18.5 mg, 0.09 mmol), and DMAP (1.3 mg, 0.01 mmol) in 10 mL of CH_2_Cl_2_. Column chromatographic purification with *n*-hexane/diethyl ether (19:1, *v*/*v*) yielded 9-isobutyryloxythymyl 2-methylbutyrate 25 mg (92%); colorless liquid; RI = 2010 (DB-5MS column); FTIR (neat; cm^−1^) 2971, 2937, 2878, 1755, 1734, 1622, 1576, 1508, 1461, 1414, 1383, 1343, 1244, 1216, 1189, 1152, 1143, 1122, 1091, 1024, 1009, 990, 968, 941, 900, 816, 751; MS (EI), *m*/*z* (%) 232 (7), 149 (15), 148 (100), 135 (24), 133 (19), 91 (7), 85 (16), 57 (55), 43 (16), 41 (10); HRMS, *m*/*z* 320.1986 [M]^+^, calculated for C_19_H_28_O_4_^•+^, 320.1982; analyzed C 71.25, H 8.79%, calculated for C_19_H_28_O_4_, C 71.22, H 8.81, O 19.97%; ^1^H NMR (CDCl_3_) δ 1.03 (ddd, ^3^*J*_3″A,4″_ = 7.5, ^3^*J*_3″B,4″_ = 7.5, ^4^*J*_2″,4″_ = 0.3, 3 H, CH_3_-4″), 1.1128/1.1019 (d, ^3^*J*_2′,3′_ = 7.0, 6 H, CH_3_-3′), 1.23 (d, ^3^*J*_8,10_ = 7.0, 3 H, CH_3_-10), 1.3156/1.3132 (d, ^3^*J*_2″,5″_ = 7.0, 3 H, CH_3_-5″), 1.6277/1.6250 (dqd, ^2^*J*_3″A,3″B_ = −13.7, ^3^*J*_3″B,4″_ = 7.5, ^3^*J*_2″,3″B_ = 6.5, 1 H, CH-3″B), 1.8625/1.8608 (ddq, ^2^*J*_3″A,3″B_ = −13.7, ^3^*J*_2″,3″A_ = 7.5, ^3^*J*_3″A,4″_ = 7.5, 1 H, CH-3″A), 2.32 (ddd, ^4^*J*_2,7_ = −0.7, ^4^*J*_6,7_ = −0.7, ^5^*J*_5,7_ = 0.3, 3 H, CH_3_-7), 2.50 (sept, ^3^*J*_2′,3′_ = 7.0, 1 H, CH-2′), 2.6595/2.6570 (dqdq, ^3^*J*_2″,3″A_ = 7.5, ^3^*J*_2″,5″_ = 7.0, ^3^*J*_2″,3″B_ = 6.5, ^4^*J*_2″,4″_ = 0.3, 1 H, CH-2″), 3.2084/3.2047 (dqdddd, ^3^*J*_8,9B_ = 7.5, ^3^*J*_8,10_ = 7.0, ^3^*J*_8,9A_ = 6.6, ^4^*J*_5,8_ = −0.5, ^5^*J*_6,8_ = 0.4, ^5^*J*_2,8_ = 0.3, 1 H, CH-8), 4.0924/4.0862 (dd, ^2^ *J*_9A,9B_ = −10.7, ^3^ *J*_8,9B_ = 7.5, 1 H, CH-9B), 4.1456/4.1425 (dd, ^2^ *J*_9A,9B_ = −10.7, ^3^ *J*_8,9A_ = 6.6, 1 H, CH-9A), 6.8097/6.8079 (dqd, ^4^*J*_2,6_ = 1.8, ^4^*J*_2,7_ = −0.7, ^5^*J*_2,8_ = 0.3, 1 H, CH-2), 7.0246/7.0243 (ddqd, ^3^*J*_5,6_ = 7.9, ^4^*J*_2,6_ = 1.8, ^4^*J*_6,7_ = −0.7, ^5^*J*_6,8_ = 0.4, 1 H, CH-6), 7.18 (ddq, ^3^*J*_5,6_ = 7.9, ^4^*J*_5,8_ = −0.5, ^5^*J*_5,7_ = 0.3, 1 H, CH-5); ^13^C NMR (CDCl_3_) δ 11.86/11.84 (C-4″), 16.84/16.79 (C-5″), 17.5 (C-10), 19.08/19.06 (C-3′), 21.1 (C-7), 26.9 (C-3″), 32.0 (C-8), 34.1 (C-2′), 41.4 (C-2″), 68.3 (C-9), 123.0 (C-2), 127.1 (C-6), 127.2 (C-5), 131.84/131.82 (C-4), 137.6 (C-1), 148.7 (C-3), 175.4 (C-1″), 177.1 (C-1′).

*9-(2-Methylbutyryloxy)thymyl isobutyrate (syn. 2-(2-isobutyryloxy-4-methylphenyl)propyl 2-methylbutyrate, **9-sBu-3-iPr**):* It was prepared from 9-(2-methylbutyryloxy)thymol (20 mg, 0.08 mmol), isobutyric acid (8 mg, 0.09 mmol), DCC (18.5 mg, 0.09 mmol), and DMAP (1.3 mg, 0.01 mmol) in 10 mL of CH_2_Cl_2_. Column chromatographic purification with *n*-hexane/diethyl ether (19:1, *v*/*v*) yielded 9-(2-methylbutyryloxy)thymyl isobutyrate 24 mg (94%); colorless liquid; RI = 2007 (DB-5MS column); FTIR (neat; cm^−1^) 2971, 2937, 2878, 1757, 1733, 1622, 1576, 1508, 1461, 1414, 1387, 1351, 1231, 1181, 1142, 1094, 1046, 1031, 1013, 982, 964, 947, 909, 879, 841, 816, 752; MS (EI), *m*/*z* (%) 218 (10), 149 (15), 148 (100), 135 (27), 133 (22), 91 (7), 71 (16), 57 (15), 43 (23), 41 (7); HRMS, *m*/*z* 320.1983 [M]^+^, calculated for C_19_H_28_O_4_^•+^, 320.1982; analyzed C 71.21, H 8.83%, calculated for C_19_H_28_O_4_, C 71.22, H 8.81, O 19.97%; ^1^H NMR (CDCl_3_) δ 0.8489/0.8326 (ddd, ^3^*J*_3′A,4′_ = 7.5, ^3^*J*_3′B,4′_ = 7.5, ^4^*J*_2′,4′_ = 0.3, 3 H, CH_3_-4′), 1.0898/1.0801 (d, ^3^*J*_2′,5′_ = 7.0, 3 H, CH_3_-5′), 1.24 (d, ^3^*J*_8,10_ = 7.0, 3 H, CH_3_-10), 1.3377/1.3364/1.3339/1.3326 (d, ^3^*J*_2″,3″_ = 7.0, 6 H, CH_3_-3″), 1.4206/1.4158 (dqd, ^2^*J*_3′A,3′B_ = −13.7, ^3^*J*_3′B,4′_ = 7.5, ^3^*J*_2′,3′B_ = 6.5, 1 H, CH-3′B), 1.6203/1.6115 (dqd, ^2^*J*_3′A,3′B_ = −13.7, ^3^*J*_3′A,4′_ = 7.5, ^3^*J*_2′,3′A_ = 7.4, 1 H, CH-3′A), 2.32 (ddd, ^4^*J*_2,7_ = −0.7, ^4^*J*_6,7_ = −0.7, ^5^*J*_5,7_ = 0.3, 3 H, CH_3_-7), 2.33 (dqdq, ^3^*J*_2′,3′A_ = 7.4, ^3^*J*_2′,5′_ = 7.0, ^3^*J*_2′,3′B_ = 6.5, ^4^*J*_2′,4′_ = 0.3, 1 H, CH-2′), 2.8446/2.8433 (sept, ^3^*J*_2″,3″_ = 7.0, 1 H, CH-2″), 3.20 (dqdddd, ^3^*J*_8,9B_ = 7.6, ^3^*J*_8,10_ = 7.0, ^3^*J*_8,9A_ = 6.4, ^4^*J*_5,8_ = −0.5, ^5^*J*_6,8_ = 0.4, ^5^*J*_2,8_ = 0.3, 1 H, CH-8), 4.0713/4.0637 (dd, ^2^ *J*_9A,9B_ = −10.8, ^3^ *J*_8,9B_ = 7.6, 1 H, CH-9B), 4.1770/4.1693 (dd, ^2^ *J*_9A,9B_ = −10.8, ^3^ *J*_8,9A_ = 6.4, 1 H, CH-9A), 6.82 (dqd, ^4^*J*_2,6_ = 1.8, ^4^*J*_2,7_ = −0.7, ^5^*J*_2,8_ = 0.3, 1 H, CH-2), 7.02 (ddqd, ^3^*J*_5,6_ = 7.9, ^4^*J*_2,6_ = 1.8, ^4^*J*_6,7_ = −0.7, ^5^*J*_6,8_ = 0.4, 1 H, CH-6), 7.17 (ddq, ^3^*J*_5,6_ = 7.9, ^4^*J*_5,8_ = −0.5, ^5^*J*_5,7_ = 0.3, 1 H, CH-5); ^13^C NMR (CDCl_3_) δ 11.72/11.64 (C-4′), 16.71/16.69 (C-5′), 17.45/17.42 (C-10), 19.16/19.11 (C-3″), 21.0 (C-7), 26.8 (C-3′), 32.03/32.01 (C-8), 34.4 (C-2″), 41.23/41.15 (C-2′), 68.2 (C-9), 123.0 (C-2), 127.1 (C-6), 127.26/127.25 (C-5), 131.8 (C-4), 137.6 (C-1), 148.7 (C-3), 175.8 (C-1″), 176.71/176.69 (C-1′).

*9-Isobutyryloxythymyl isovalerate (syn. 2-(1-isobutyryloxypropan-2-yl)-5-methylphenyl isovalerate, **9-iPr-3-iBu**):* It was prepared only for GC–MS analysis from 9-isobutyryloxythymol (2.4 mg, 0.01 mmol), isovaleric acid (1 mg, 0.01 mmol), DCC (2.1 mg, 0.01 mmol), and DMAP (1.3 mg, 0.01 mmol) in 1 mL of dry CH_2_Cl_2_; RI = 2008 (DB-5MS column); MS (EI), *m*/*z* (%) 149 (14), 148 (100), 135 (23), 133 (18), 91 (6), 85 (13), 71 (7), 57 (24), 43 (22), 41 (12).

*9-Isovaleroxythymyl isobutyrate (syn. 2-(2-isobutyryloxy-4-methylphenyl)propyl isovalerate, **9-iBu-3-iPr**):* It was prepared only for GC–MS analysis from 9-isovaleroxythymol (2.5 mg, 0.01 mmol), isobutyric acid (0.9 mg, 0.01 mmol), DCC (2.1 mg, 0.01 mmol), and DMAP (1.3 mg, 0.01 mmol) in 1 mL of dry CH_2_Cl_2_; RI = 2011 (DB-5MS column); MS (EI), *m*/*z* (%) 149 (19), 148 (100), 135 (28), 133 (24), 91 (12), 85 (15), 71 (30), 57 (23), 43 (88), 41 (32).

*9-(2-Methylbutyryloxy)thymyl isovalerate (syn. 2-(4-methyl-2-isovaleroxyphenyl)propyl 2-methylbutyrate, **9-sBu-3-iBu**):* It was prepared only for GC–MS analysis from 9-(2-methylbutyryloxy)thymol (2.5 mg, 0.01 mmol), isovaleric acid (1 mg, 0.01 mmol), DCC (2.1 mg, 0.01 mmol), and DMAP (1.3 mg, 0.01 mmol) in 1 mL of dry CH_2_Cl_2_; RI = 2096 (DB-5MS column); MS (EI), *m*/*z* (%) 232 (5), 149 (15), 148 (100), 135 (21), 133 (16), 121 (4), 91 (5), 85 (14), 57 (30), 41 (8).

*9-Isovaleroxythymyl 2-methylbutyrate (syn. 2-(4-methyl-2-(2-methylbutyryloxy)phenyl)propyl isovalerate, **9-iBu-3-sBu**):* It was prepared only for GC–MS analysis from 9-isovaleroxythymol (2.5 mg, 0.01 mmol), 2-methylbutyric acid (1 mg, 0.01 mmol), DCC (2.1 mg, 0.01 mmol), and DMAP (1.3 mg, 0.01 mmol) in 1 mL of dry CH_2_Cl_2_; RI = 2097 (DB-5MS column); MS (EI), *m*/*z* (%) 149 (16), 148 (100), 135 (25), 133 (30), 85 (24), 57 (94), 56 (11), 43 (14), 42 (10), 41 (54).

### 3.9. Synthesis of Esters for RI Standards

#### 3.9.1. Thymyl Esters

Esters of thymol with isobutyric, 2-methylbutyric, and isovaleric acids were synthesized following a standard Steglich esterification protocol as described above in Section 3.3.3.

*Thymyl isobutyrate (syn. 2-isopropyl-5-methylphenyl isobutyrate, **Thy-iPr**):* It was prepared only for GC–MS analysis from thymol (1.5 mg, 0.01 mmol), isobutyric acid (0.9 mg, 0.01 mmol), DCC (2.1 mg, 0.01 mmol), and DMAP (1.3 mg, 0.01 mmol) in 1 mL of dry CH_2_Cl_2_; RI = 1487 (DB-5MS column); MS (EI), *m*/*z* (%) 220 (8), 150 (52), 136 (9), 135 (100), 105 (9), 91 (18), 77 (9), 71 (14), 43 (35), 41 (10).

*Thymyl 2-methylbutyrate (syn. 2-isopropyl-5-methylphenyl 2-methylbutyrate, **Thy-sBu**):* It was prepared only for GC–MS analysis from thymol (1.5 mg, 0.01 mmol), 2-methylbutyric acid (1 mg, 0.01 mmol), DCC (2.1 mg, 0.01 mmol), and DMAP (1.3 mg, 0.01 mmol) in 1 mL of dry CH_2_Cl_2_; RI = 1578 (DB-5MS column); MS (EI), *m*/*z* (%) 234 (11), 150 (67), 135 (100), 121 (7), 115 (10), 105 (9), 91 (16), 77 (7), 57 (48), 41 (13).

*Thymyl isovalerate (syn. 2-isopropyl-5-methylphenyl isovalerate, **Thy-iBu**):* It was prepared only for GC–MS analysis from thymol (1.5 mg, 0.01 mmol), isovaleric acid (1 mg, 0.01 mmol), DCC (2.1 mg, 0.01 mmol), and DMAP (1.3 mg, 0.01 mmol) in 1 mL of dry CH_2_Cl_2_; RI = 1581 (DB-5MS column); MS (EI), *m*/*z* (%) 151 (7), 150 (60), 136 (9), 135 (100), 115 (7), 105 (7), 91 (11), 85 (10), 57 (32), 41 (13).

#### 3.9.2. Carvacryl Esters

Esters of carvacrol with isobutyric, 2-methylbutyric, and isovaleric acids were synthesized following a standard Steglich esterification protocol as described above in Section 3.3.3. These esters were synthesized only for GC–MS analysis.

*Carvacryl isobutyrate (syn. 5-isopropyl-2-methylphenyl isobutyrate, **Carv-iPr**):* It was prepared only for GC–MS analysis from carvacrol (1.5 mg, 0.01 mmol), isobutyric acid (0.9 mg, 0.01 mmol), DCC (2.1 mg, 0.01 mmol), and DMAP (1.3 mg, 0.01 mmol) in 1 mL of dry CH_2_Cl_2_; RI = 1515 (DB-5MS column); MS (EI), *m*/*z* (%) 220 (11), 151 (11), 150 (100), 135 (100), 105 (10), 91 (23), 77 (12), 71 (17), 43 (45), 41 (13).

*Carvacryl 2-methylbutyrate (syn. 5-isopropyl-2-methylphenyl 2-methylbutyrate, **Carv-sBu**):* It was prepared only for GC–MS analysis from carvacrol (1.5 mg, 0.01 mmol), 2-methylbutyric acid (1 mg, 0.01 mmol), DCC (2.1 mg, 0.01 mmol), and DMAP (1.3 mg, 0.01 mmol) in 1 mL of dry CH_2_Cl_2_; RI = 1607 (DB-5MS column); MS (EI), *m*/*z* (%) 234 (11), 150 (100), 135 (86), 133 (6), 107 (5), 105 (8), 91 (23), 85 (12), 77 (11), 57 (66).

*Carvacryl isovalerate (syn. 5-isopropyl-2-methylphenyl isovalerate, **Carv-iBu**):* It was prepared only for GC–MS analysis from carvacrol (1.5 mg, 0.01 mmol), isovaleric acid (1 mg, 0.01 mmol), DCC (2.1 mg, 0.01 mmol), and DMAP (1.3 mg, 0.01 mmol) in 1 mL of dry CH_2_Cl_2_; RI = 1608 (DB-5MS column); MS (EI), *m*/*z* (%) 234 (6), 151 (11), 150 (100), 136 (7), 135 (78), 107 (4), 105 (3), 91 (5), 57 (4), 41 (3).

#### 3.9.3. *p*-Cymene-7-ol (Cuminyl) Esters

Esters of cuminol with isobutyric, 2-methylbutyric, and isovaleric acids were synthesized following a standard Steglich esterification protocol as described above in Section 3.3.3. These esters were synthesized only for GC–MS analysis.

*Cuminyl isobutyrate (syn. 4-isopropylbenzyl isobutyrate, **Cum-iPr**):* It was prepared only for GC–MS analysis from cuminol (1.5 mg, 0.01 mmol), isobutyric acid (0.9 mg, 0.01 mmol), DCC (2.1 mg, 0.01 mmol), and DMAP (1.3 mg, 0.01 mmol) in 1 mL of dry CH_2_Cl_2_; RI = 1554 (DB-5MS column); MS (EI), *m*/*z* (%) 220 (18), 150 (13), 134 (11), 133 (100), 118 (12), 117 (29), 107 (19), 105 (19), 91 (16), 71 (11).

*Cuminyl 2-methylbutyrate (syn. 4-isopropylbenzyl 2-methylbutyrate, **Cum-sBu**):* It was prepared only for GC–MS analysis from cuminol (1.5 mg, 0.01 mmol), 2-methylbutyric acid (1 mg, 0.01 mmol), DCC (2.1 mg, 0.01 mmol), and DMAP (1.3 mg, 0.01 mmol) in 1 mL of dry CH_2_Cl_2_; RI = 1641 (DB-5MS column); MS (EI), *m*/*z* (%) 234 (14), 150 (14), 134 (12), 133 (100), 118 (10), 117 (23), 107 (16), 105 (15), 91 (13), 57 (15).

*Cuminyl isovalerate (syn. 4-isopropylbenzyl isovalerate, **Cum-iBu**):* It was prepared only for GC–MS analysis from cuminol (1.5 mg, 0.01 mmol), isovaleric acid (1 mg, 0.01 mmol), DCC (2.1 mg, 0.01 mmol), and DMAP (1.3 mg, 0.01 mmol) in 1 mL of dry CH_2_Cl_2_; RI = 1652 (DB-5MS column); MS (EI), *m*/*z* (%) 234 (25), 150 (46), 133 (100), 118 (17), 117 (50), 107 (69), 105 (23), 91 (24), 85 (22), 57 (17).

#### 3.9.4. *p*-Cymene-8-ol Esters

Esters of *p*-cymene-8-ol with isobutyric, 2-methylbutyric, and isovaleric acids were synthesized following a standard Steglich esterification protocol as described above in Section 3.3.3.

*p-Cymene-8-yl isobutyrate (syn. 2-(4-methylphenyl)propan-2-yl isobutyrate, **Cym8-iPr**):* It was prepared only for GC–MS analysis from *p*-cymene-8-ol (1.5 mg, 0.01 mmol), isobutyric acid (0.9 mg, 0.01 mmol), DCC (2.1 mg, 0.01 mmol), and DMAP (1.3 mg, 0.01 mmol) in 1 mL of dry CH_2_Cl_2_; RI = 1466 (DB-5MS column); MS (EI), *m*/*z* (%) 133 (36), 132 (100), 131 (18), 117 (97), 116 (18), 115 (63), 92 (22), 91 (52), 65 (20), 43 (13).

*p-Cymene-8-yl 2-methylbutyrate (syn. 2-(4-methylphenyl)propan-2-yl 2-methylbutyrate, **Cym8-sBu**):* It was prepared only for GC–MS analysis from *p*-cymene-8-ol (1.5 mg, 0.01 mmol), 2-methylbutyric acid (1 mg, 0.01 mmol), DCC (2.1 mg, 0.01 mmol), and DMAP (1.3 mg, 0.01 mmol) in 1 mL of dry CH_2_Cl_2_; RI = 1558 (DB-5MS column); MS (EI), *m*/*z* (%) 133 (46), 132 (100), 131 (19), 117 (99), 116 (17), 115 (63), 92 (21), 91 (51), 65 (19), 51 (12).

*p-Cymene-8-yl isovalerate (syn. 2-(4-methylphenyl)propan-2-yl isovalerate, **Cym8-iBu**):* It was prepared only for GC–MS analysis from *p*-cymene-8-ol (1.5 mg, 0.01 mmol), isovaleric acid (1 mg, 0.01 mmol), DCC (2.1 mg, 0.01 mmol), and DMAP (1.3 mg, 0.01 mmol) in 1 mL of dry CH_2_Cl_2_; RI = 1567 (DB-5MS column); MS (EI), *m*/*z* (%) 133 (37), 132 (100), 117 (94), 116 (15), 115 (65), 92 (24), 91 (38), 65 (22), 60 (14), 51 (14).

#### 3.9.5. Synthesis of *p*-Cymene-9-ol and Its Esters

##### Dehydration of *p*-Cymene-8-ol (Synthesis of *p*-Cymenene)

*p*-Cymenene was obtained by acid-catalyzed dehydration of *p*-cymene-8-ol. A solution of *p*-cymene-8-ol (1.5 g, 10 mmol) in CH_2_Cl_2_ (50 mL) was treated with *p*-toluenesulfonic acid (0.172 g, 1 mmol) and an excess of anhydrous MgSO_4_. The reaction mixture was stirred magnetically at room temperature for 1 h. After completion of the reaction, the solvent was removed under reduced pressure and the crude product was purified by dry-flash chromatography on silica gel using *n*-hexane as the eluent to afford *p*-cymenene (1.22 g, 92%).

*p-Cymenene (syn. 1-methyl-4-(prop-1-en-2-yl)benzene):* colorless liquid; RI = 1092 (DB-5MS column); MS (EI), *m*/*z* (%) 132 (82), 117 (100), 116 (20), 115 (75), 92 (19), 91 (56), 89 (18), 65 (23), 63 (26), 51 (19); ^1^H NMR (CDCl_3_) δ 2.14 (dd, ^4^*J*_9B,10_ = −1.5, ^4^*J*_9A,10_ = −0.8, 3 H, CH_3_-10), 2.34 (dddd, ^4^*J*_2,7_ = −0.7, ^4^*J*_6,7_ = −0.7, ^5^*J*_3,7_ = 0.3, ^5^*J*_5,7_ = 0.3, 3 H, CH_3_-7), 5.03 (dq, ^2^*J*_9A,9B_ = 1.6, ^4^*J*_9B,10_ = −1.5, 1 H, CH-9B), 5.33 (dq, ^2^*J*_9A,9B_ = 1.6, ^4^*J*_9A,10_ = −0.8, 1 H, CH-9A), 7.14 (ddqd, ^3^*J*_2,3_ = 8.0, ^4^*J*_2,6_ = 1.9, ^4^*J*_2,7_ = −0.7, ^5^*J*_2,5_ = 0.4, 1 H, CH-2), 7.14 (ddqd, ^3^*J*_5,6_ = 8.0, ^4^*J*_2,6_ = 1.9, ^4^*J*_6,7_ = −0.7, ^5^*J*_3,6_ = 0.4, 1 H, CH-6), 7.37 (dddq, ^3^*J*_2,3_ = 8.0, ^4^*J*_3,5_ = 2.3, ^5^*J*_3,6_ = 0.4, ^5^*J*_3,7_ = 0.3, 1 H, CH-3), 7.37 (dddq, ^3^*J*_5,6_ = 8.0, ^4^*J*_3,5_ = 2.3, ^5^*J*_2,5_ = 0.4, ^5^*J*_5,7_ = 0.3, 1 H, CH-5); ^13^C NMR (CDCl_3_) δ 21.2 (C-7), 22.0 (C-10), 111.7 (C-9), 125.5 (C-3 and C-5), 129.0 (C-2 and C-6), 137.3 (C-1), 138.5 (C-4), 143.2 (C-8).

##### Hydroboration–Oxidation of *p*-Cymenene (Synthesis of *p*-Cymene-9-ol)

*p*-Cymene-9-ol was synthesized by hydroboration–oxidation of *p*-cymenene. A dry three-necked flask equipped with a magnetic stirrer, internal thermometer, dropping funnel, and argon inlet was charged with dry THF (20 mL) and finely powdered NaBH_4_ (0.76 g, 19 mmol) [3]. The mixture was stirred until a homogeneous suspension was obtained and cooled to −5 °C using an ice–salt bath. A solution of iodine (1.93 g, 7.6 mmol) in dry THF (20 mL) was added dropwise under continuous stirring. Subsequently, a solution of *p*-cymenene (1.0 g, 7.6 mmol) in dry THF (10 mL) was added dropwise over 10 min while maintaining the reaction temperature below 30 °C. The reaction mixture was stirred at 25 °C for 2 h and then cooled to 0 °C. Water (8 mL), THF (10 mL), and 3 M aqueous NaOH solution (20 mL) were added successively, followed by dropwise addition of 30% H_2_O_2_ (517 mg, 15.2 mmol, 1.72 mL). Stirring was continued for an additional 20 min after complete addition of hydrogen peroxide. The reaction mixture was transferred to a separatory funnel, and the reaction vessel was rinsed with diethyl ether (30 mL). The aqueous phase was saturated with NaCl and extracted with diethyl ether (3 × 30 mL). The combined organic layers were washed with saturated aqueous NaCl solution (2 × 30 mL), dried with anhydrous MgSO_4_, filtered, and concentrated under reduced pressure. The crude product was purified by column chromatography on silica gel using *n*-hexane/Et_2_O (4:1, *v*/*v*) as the eluent to afford *p*-cymene-9-ol (716 mg, 63%).

*p-Cymene-9-ol (syn. 2-(p-tolyl)propan-1-ol):* brown viscous oil; RI = 1277 (DB-5MS column); MS (EI), *m*/*z* (%) 150 (17), 120 (11), 119 (100), 118 (10), 117 (36), 115 (20), 103 (12), 91 (48), 77 (16), 65 (12); ^1^H NMR (CDCl_3_) δ 1.25 (d, ^3^*J*_8,10_ = 7.0, 3 H, CH_3_-10), 1.58 (brs, 1 H, OH), 2.33 (dddd, ^4^*J*_2,7_ = −0.5, ^4^*J*_6,7_ = −0.5, ^5^*J*_3,7_ = 0.3, ^5^*J*_5,7_ = 0.3, 3 H, CH_3_-7), 2.91 (qtdddd, ^3^*J*_8,10_ = 7.0, ^3^*J*_8,9_ = 6.9, ^5^*J*_2,8_ = 0.3, ^4^*J*_3,8_ = −0.3, ^4^*J*_5,8_ = −0.3, ^5^*J*_6,8_ = 0.3, 1 H, CH-8), 3.67 (d, ^3^*J*_8,9_ = 6.9, 2 H, CH_2_-9), 7.1326 (dq, ^4^*J*_3,8_ = −0.3, ^5^*J*_3,7_ = 0.3, 1 H, CH-3), 7.1326 (dq, ^4^*J*_5,8_ = −0.3, ^5^*J*_5,7_ = 0.3, 1 H, CH-5), 7.1352 (qd, ^4^*J*_2,7_ = −0.5, ^5^*J*_2,8_ = 0.3, 1 H, CH-2), 7.1352 (qd, ^4^*J*_6,7_ = −0.5, ^5^*J*_6,8_ = 0.3, 1 H, CH-6); ^13^C NMR (CDCl_3_) δ 17.8 (C-10), 21.1 (C-7), 42.1 (C-8), 68.9 (C-9), 127.5 (C-3 and C-5), 129.5 (C-2 and C-6), 136.3 (C-1), 140.7 (C-4).

##### *p*-Cymene-9-ol Esters

Esters of *p*-cymene-9-ol with isobutyric, 2-methylbutyric, and isovaleric acids were synthesized following a standard Steglich esterification protocol as described above in Section 3.3.3.

*p-Cymene-9-yl isobutyrate (syn. 2-(p-tolyl)propyl isobutyrate, **Cym9-iPr**):* It was prepared only for GC–MS analysis from *p*-cymene-9-ol (1.5 mg, 0.01 mmol), isobutyric acid (0.9 mg, 0.01 mmol), DCC (2.1 mg, 0.01 mmol), and DMAP (1.3 mg, 0.01 mmol) in 1 mL of dry CH_2_Cl_2_; RI = 1540 (DB-5MS column); MS (EI), *m*/*z* (%) 133 (12), 132 (100), 119 (40), 118 (6), 117 (28), 115 (10), 105 (12), 91 (13), 71 (11), 43 (12).

*p-Cymene-9-yl 2-methylbutyrate (syn. 2-(p-tolyl)propyl 2-methylbutyrate, **Cym9-sBu**):* It was prepared only for GC–MS analysis from *p*-cymene-9-ol (1.5 mg, 0.01 mmol), 2-methylbutyric acid (1 mg, 0.01 mmol), DCC (2.1 mg, 0.01 mmol), and DMAP (1.3 mg, 0.01 mmol) in 1 mL of dry CH_2_Cl_2_; RI = 1629 (DB-5MS column); MS (EI), *m*/*z* (%) 133 (12), 132 (100), 119 (28), 118 (5), 117 (26), 115 (10), 105 (12), 91 (11), 85 (7), 57 (19).

*p-Cymene-9-yl isovalerate (syn. 2-(p-tolyl)propyl isovalerate, **Cym9-iBu**):* It was prepared from *p*-cymene-9-ol (15 mg, 0.1 mmol), isovaleric acid (10 mg, 0.1 mmol), DCC (21 mg, 0.1 mmol), and DMAP (1.3 mg, 0.01 mmol) in 10 mL of dry CH_2_Cl_2_; column chromatographic purification with *n*-hexane/diethyl ether (19:1, *v*/*v*) yielded *p*-cymene-9-yl isovalerate 22 mg (95%); colorless liquid; RI = 1633 (DB-5MS column); MS (EI), *m*/*z* (%) 133 (16), 132 (100), 131 (6), 119 (29), 117 (36), 115 (14), 105 (14), 91 (14), 85 (12), 57 (12); ^1^H NMR (CDCl_3_) δ 0.90 (d, ^3^*J*_3′,4′_ = 6.7, 6 H, CH_3_-4′), 1.28 (d, ^3^*J*_8,10_ = 7.0, 3 H, CH_3_-10), 2.04 (tsept, ^3^*J*_2′,3′_ = 7.2, ^3^*J*_3′,4′_ = 6.7, 1 H, CH-3′), 2.15 (d, ^3^*J*_2′,3′_ = 7.2, 2 H, CH_2_-2′), 2.32 (s, 3 H, CH_3_-7), 3.05 (dqd,^3^*J*_8,9B_ = 7.4, ^3^*J*_8,10_ = 7.0, ^3^*J*_8,9A_ = 6.8, 1 H, CH-8), 4.10 (dd, ^2^*J*_9A,9B_ = −10.8, ^3^*J*_8,9B_ = 7.4, 1 H, CH-9B), 4.19 (dd, ^2^*J*_9A,9B_ = −10.8, ^3^*J*_8,9A_ = 6.8, 1 H, CH-9A), 7.11 (s, 4 H, CH-2, CH-3, CH-5, and CH-6); ^13^C NMR (CDCl_3_) δ 18.3 (C-10), 21.1 (C-7), 22.5 (C-4′), 25.8 (C-3′), 38.7 (C-8), 43.6 (C-2′), 69.3 (C-9), 127.3 (C-3 and C-5), 129.3 (C-2 and C-6), 136.3 (C-1), 140.3 (C-4), 173.2 (C-1′).

## 4. Conclusions

In this study, a comprehensive library of diacylated hydroxythymol derivatives was developed in order to facilitate the GC–MS identification of closely related regioisomeric and acyl-homologous compounds in complex natural matrices. The synthesized set comprised 49 hydroxythymol diesters differing in the position of the second acyloxy substituent on the thymol scaffold and in the nature of the branched-chain acyl residues. Among these compounds, 44 have not previously been described in the literature.

The systematic determination of retention indices on both DB-5MS and HP-Innowax columns provided a unified dual-column RI dataset for this structurally coherent group of compounds. The bis(isobutyryl) derivatives followed the elution order 8 < 2 < 9 < 5 < 6 < 7 on both stationary phases. A broadly similar pattern was observed for other acyl combinations, although several reversals and near-coincidences occurred. These exceptions show that substituent position and acyl-residue identity jointly influence retention. Correlation analysis and the model ester subtraction approach further showed that retention behavior can be rationalized within defined structural subgroups, particularly for the 5-, 6-, 7-, and 9-substituted derivatives. In contrast, the 2- and 8-substituted series displayed systematic deviations, consistent with their more compact and sterically constrained molecular arrangements.

Comparison with literature data revealed noticeable differences in reported RI values and, in certain cases, in EI mass spectral patterns for previously assigned hydroxythymol diesters. These discrepancies underline the limitations of tentative identification based solely on literature RI values or library mass spectra. The present results therefore emphasize the importance of synthetic reference compounds, dual-column RI data, EI-MS comparison, and co-injection for reliable differentiation of closely related hydroxythymol esters.

Application of the synthesized reference library to the more polar fraction of *Doronicum columnae* root essential oil enabled the identification of 16 diacylated hydroxythymol derivatives, 12 of which are reported here for the first time as natural constituents. These findings considerably expand the known chemical diversity of *D. columnae* essential oil and demonstrate that this species contains a structurally rich series of regioisomeric hydroxythymol diesters in addition to the previously reported methoxy-*p*-cymene derivatives.

The botanical application was restricted to one enriched root-oil fraction, and the results do not establish population-wide occurrence or the quantitative composition of the unfractionated oil. The C-6 regioisomers 6-iPr-3-sBu and 6-sBu-3-iPr were assigned using individual standard additions within a partially overlapping chromatographic region, without baseline separation. The library also includes components assigned in synthetic mixtures without individual NMR characterization; their evidence level is identified explicitly. The retention relationships describe the present dataset and require independent testing before predictive use. Assignments concern constitutional structures; the absolute configurations and stereoisomeric composition of the natural constituents were not established.

The GC–MS/RI dataset provides reference information for distinguishing positional and acyl variants of hydroxythymol diesters in complex botanical samples. Its immediate contribution is improved constituent identification through combined structural, mass-spectral and chromatographic evidence. The data may also support comparative phytochemical studies and the development of chemical fingerprints. Their use in botanical authentication or quality control will require validation of marker specificity, reproducibility and variation across independently collected samples.

## Figures and Tables

**Table 1:** Retention indices of hydroxythymol diesters bearing a second acyloxy substituent at positions C-2, C-5, and C-6.

	RIa ^a^	RIp ^b^		RIa	RIp		RIa	RIp
2-iPr-3-iPr	1902	2475	5-iPr-3-iPr	1933	2501	6-iPr-3-iPr	1952	2518
2-sBu-3-iPr	1997	2559	5-sBu-3-iPr	2014	2587	6-sBu-3-iPr	2045	2604
2-iPr-3-sBu	2004	2568	5-iPr-3-sBu			6-iPr-3-sBu	2044	2604
2-iBu-3-iPr	1975	2538	5-iBu-3-iPr	2028	2597	6-iBu-3-iPr	2050	2619
2-iPr-3-iBu	1980	2544	5-iPr-3-iBu			6-iPr-3-iBu	2047	2615
2-sBu-3-sBu	2089	2645	5-sBu-3-sBu	2121	2672	6-sBu-3-sBu	2142	2692
2-sBu-3-iBu	2070	2624	5-sBu-3-iBu	2123	2681	6-sBu-3-iBu	2143	2702
2-iBu-3-sBu	2070	2624	5-iBu-3-sBu			6-iBu-3-sBu	2144	2702
2-iBu-3-iBu	2055	2609	5-iBu-3-iBu	2126	2689	6-iBu-3-iBu	2145	2713

^a^ Experimental retention indices determined relative to a homologous series of *n*-alkanes (C_8_–C_30_) on a non-polar DB-5MS column; ^b^ experimental retention indices determined relative to a homologous series of *n*-alkanes (C_8_–C_30_) on a polar HP-Innowax GC column.

**Table 2:** Retention indices of hydroxythymol diesters bearing a second acyloxy substituent at positions C-7, C-8, and C-9.

	RIa ^a^	RIp ^b^		RIa	RIp		RIa	RIp
7-iPr-3-iPr	1961	2568	8-iPr-3-iPr	1894	2467	9-iPr-3-iPr	1921	2497
7-sBu-3-iPr	2045	2643	8-sBu-3-iPr	1987	2552	9-sBu-3-iPr	2007	2578
7-iPr-3-sBu	2051	2649	8-iPr-3-sBu	1987	2545	9-iPr-3-sBu	2010	2575
7-iBu-3-iPr	2061	2678	8-iBu-3-iPr	1980	2566	9-iBu-3-iPr	2011	2583
7-iPr-3-iBu	2057	2669	8-iPr-3-iBu	1974	2538	9-iPr-3-iBu	2008	2594
7-sBu-3-sBu	2135	2726	8-sBu-3-sBu	2079	2628	9-sBu-3-sBu	2095	2653
7-sBu-3-iBu	2142	2743	8-sBu-3-iBu	/	/	9-sBu-3-iBu	2096	2662
7-iBu-3-sBu	2147	2756	8-iBu-3-sBu	/	/	9-iBu-3-sBu	2097	2670
7-iBu-3-iBu	2150	2771	8-iBu-3-iBu	2068	2632	9-iBu-3-iBu	2098	2680

^a^ Experimental retention indices determined relative to a homologous series of *n*-alkanes (C_8_–C_30_) on a non-polar DB-5MS column; ^b^ experimental retention indices determined relative to a homologous series of *n*-alkanes (C_8_–C_30_) on a polar HP-Innowax GC column; / not obtained.

**Table 3:** Mean residual retention-index increments calculated using the model ester subtraction approach. Individual residual increments were obtained by subtracting the RI value of the corresponding model ester from the RI value of the related hydroxythymol diester. Mean residual increments were calculated separately for each acyloxy position, acyl residue, and chromatographic column. The value of *n* denotes the number of individual residual-increment values included in the calculation of each mean. For complete series, *n* = 3; for the C-8 increments involving sBu or iBu, for which *n* = 2.

Diester	Residual Increment	R = iPr		R = sBu		R = iBu	
		$\bar{\Delta \mathrm{RIa}}$ ^a^	$\bar{\Delta \mathrm{RIp}}$ ^b^	$\bar{\Delta \mathrm{RIa}}$	$\bar{\Delta \mathrm{RIp}}$	$\bar{\Delta \mathrm{RIa}}$	$\bar{\Delta \mathrm{RIp}}$
2R–3R	2R	413	562	503	643	485	624
	3R	381	521	478	610	458	590
5R–3R	5R = 3R ^c^	443	595	537	680	544	689
6R–3R	6R	466	612	561	699	564	711
	3R	439	578	533	663	535	674
7R–3R	7R	474	662	559	737	571	768
	3R	407	549	495	630	501	647
8R–3R	8R	403	550	501	642	490	645
	3R	423	583	521	664	505	650
9R–3R	9R	431	589	518	668	520	678
	3R	379	502	467	582	467	594

^a^ Mean residual retention-index increments calculated for DB-5MS; ^b^ mean residual retention-index increments calculated for HP-Innowax; ^c^ for the 5-substituted series, positions C-3 and C-5 are symmetry-related on the *p*-cymene skeleton; therefore, the corresponding residual increments are equivalent.

**Table 4:** Identification of hydroxythymol diesters in the more polar fraction of *D. columnae* root essential oil.

Code Name	Compound	RIa ^a^	RIp ^b^	ID ^c^	Status
9-iPr-3-iPr	9-Isobutyryloxythymyl isobutyrate	1921	2497	MS, CoI	KNOWN [7,8,9,10]
6-iPr-3-iPr	6-Isobutyryloxythymyl isobutyrate	1952	2518	MS, CoI	NEW
7-iPr-3-iPr	7-Isobutyryloxythymyl isobutyrate	1961	2568	MS, CoI	KNOWN [7,8,9,10]
9-sBu-3-iPr	9-(2-Methylbutyryloxy)thymyl isobutyrate	2007	2578	MS, CoI	KNOWN [7,8,9,10]
9-iPr-3-sBu	9-Isobutyryloxythymyl 2-methylbutyrate	2010	2575	MS, CoI	NEW
6-iPr-3-sBu	6-Isobutyryloxythymyl 2-methylbutyrate	2044 ^d^	2604	MS, CoI	NEW
6-sBu-3-iPr	6-(2-Methylbutyryloxy)thymyl isobutyrate	2045 ^d^	2604	MS, CoI	NEW
7-sBu-3-iPr	7-(2-Methylbutyryloxy)thymyl isobutyrate	2045	2643	MS, CoI	KNOWN [11]
7-iPr-3-sBu	7-Isobutyryloxythymyl 2-methylbutyrate	2051	2649	MS, CoI	NEW
7-iPr-3-iBu	7-Isobutyryloxythymyl isovalerate	2057	2669	MS, CoI	NEW
7-iBu-3-iPr	7-Isovaleroxythymyl isobutyrate	2061	2678	MS, CoI	NEW
9-sBu-3-sBu	9-(2-Methylbutyryloxy)thymyl 2-methylbutyrate	2095	2653	MS, CoI	NEW
7-sBu-3-sBu	7-(2-Methylbutyryloxy)thymyl 2-methylbutyrate	2135	2726	MS, CoI	NEW
7-sBu-3-iBu	7-(2-Methylbutyryloxy)thymyl isovalerate	2142	2743	MS, CoI	NEW
7-iBu-3-sBu	7-Isovaleroxythymyl 2-methylbutyrate	2147	2756	MS, CoI	NEW
7-iBu-3-iBu	7-Isovaleroxythymyl isovalerate	2150	2771	MS, CoI	NEW

^a^ Experimental retention indices determined relative to a homologous series of *n*-alkanes (C_8_–C_30_) on a non-polar DB-5MS column. ^b^ Experimental retention indices determined relative to a homologous series of *n*-alkanes (C_8_–C_30_) on a polar HP-Innowax GC column. ^c^ ID = identification method. ^d^ For 6-iPr-3-sBu and 6-sBu-3-iPr, the DB-5MS co-injection evidence was evaluated from the enhancement of separate pre-existing shoulders in the unspiked fraction (Figure S171). The identical rounded HP-Innowax RI values do not provide independent discrimination between these regioisomers. MS = constituent identified by mass spectra comparison with those listed in Wiley 11, NIST17 [18], MassFinder 2.3, and mass spectra of herein synthesized standards; CoI = constituent identity confirmed by GC co-injection with a synthetic reference compound. The RI values measured for the constituents of the natural fraction and the corresponding synthetic references were identical at the reported precision on both stationary phases; therefore, ΔRI = RI(natural fraction) − RI(synthetic reference) = 0 for all listed constituents on both DB-5MS and HP-Innowax. Co-injection experiments were performed on both the non-polar DB-5MS and polar HP-Innowax columns; NEW = compound reported here for the first time as a natural constituent.

## Data Availability

The original contributions presented in this study are included in the article and Supplementary Materials. Further inquiries can be directed to the corresponding author.

## Supplementary Materials

The following supporting information can be downloaded at https://www.mdpi.com/article/10.3390/plants15213430/s1. Schemes S1–S6, Figures S1–S171 and Tables S1–S29.
