# Supplementary material for: Synthesis-Supported GC–MS/Retention-Index Library of Hydroxythymol Diesters for Analysis of Botanical Matrices
Source: Plants (Basel). 2026 Nov 4;15(21):3430. doi: 10.3390/plants15213430 (PMC13765781; doi:10.3390/plants15213430)
Supplement: Supplementary file 1 [file plants-15-03430-s001.zip › plants-4608423-supplementary.pdf]

Supplementary material

for

Synthesis-supported GC–MS/retention-index library of hydroxythymol diesters for analysis of botanical matrices

Milan Ž. Dimitrijević, Niko S. Radulović*,[1]

[1] Department of Chemistry, Faculty of Sciences and Mathematics, University of Niš, Višegradska 33, 18000, Niš, Serbia

* Correspondence: nikoradulovic@yahoo.com; Tel.: +381-18-533-015; Fax: +381-18-533-014

Content:

Scheme S1. Synthetic pathway to 2-hydroxythymol-derived monoesters and diesters (R,R′ – isopropyl, *sec*-butyl, or isobutyl).

Figure S1. Mass spectrum (EI, 70 eV) of 2-isobutyryloxythymyl isobutyrate.

Figure S2. IR spectrum of 2-isobutyryloxythymyl isobutyrate.

Figure S3. ^{1}H NMR (400 MHz, $CDCl_3$) spectrum of 2-isobutyryloxythymyl isobutyrate and the corresponding expansions with signal assignment.

Figure S4. Simulated (manual iterative full spin, MestreNova 11.0.3) and experimental ^{1}H NMR spectrum of 2-isobutyryloxythymyl isobutyrate.

Figure S5. ^{13}C NMR (100.6 MHz, $CDCl_3$) spectrum of 2-isobutyryloxythymyl isobutyrate.

Table S1. ^{1}H (400 MHz) and ^{13}C (100.6 MHz) NMR data of 2-isobutyryloxythymyl isobutyrate (chloroform-*d*); NMR parameters are derived from manual iterative full spin analysis, along with the observed gHMBC and NOESY correlations.

Figure S6. NOESY (blue arrows) and HMBC (red arrows) interactions of 2-isobutyryloxythymyl isobutyrate.

Figure S7. Mass spectrum (EI, 70 eV) of 2-(2-methylbutyryloxy)thymyl isobutyrate.

Figure S8. Mass spectrum (EI, 70 eV) of 2-isobutyryloxythymyl 2-methylbutyrate.

Figure S9. IR spectrum of the mixture of 2-(2-methylbutyryloxy)thymyl isobutyrate and 2-isobutyryloxythymyl 2-methylbutyrate.

Figure S10. ^{1}H NMR (400 MHz, $CDCl_3$) spectrum of the mixture of 2-(2-methylbutyryloxy)thymyl isobutyrate (y) and 2-isobutyryloxythymyl 2-methylbutyrate (x) and the corresponding expansions with signal assignment.

Figure S11. Simulated (manual iterative full spin, MestreNova 11.0.3) and experimental ^{1}H NMR spectrum of the mixture of 2-(2-methylbutyryloxy)thymyl isobutyrate (y) and 2-isobutyryloxythymyl 2-methylbutyrate (x).

Figure S12. ^{13}C NMR (100.6 MHz, $CDCl_3$) spectrum of the mixture of 2-(2-methylbutyryloxy)thymyl isobutyrate (y) and 2-isobutyryloxythymyl 2-methylbutyrate (x).

Table S2. ^{1}H (400 MHz) and ^{13}C (100.6 MHz) NMR data of 2-(2-methylbutyryloxy)thymyl isobutyrate (chloroform-*d*); NMR parameters are derived from manual iterative full spin analysis, along with the observed gHMBC and NOESY correlations.

Figure S13. NOESY (blue arrows) and HMBC (red arrows) interactions of 2-(2-methylbutyryloxy)thymyl isobutyrate.

Table S3. ^{1}H (400 MHz) and ^{13}C (100.6 MHz) NMR data of 2-isobutyryloxythymyl 2-methylbutyrate (chloroform-*d*); NMR parameters are derived from manual iterative full spin analysis, along with the observed gHMBC and NOESY correlations.

Figure S14. NOESY (blue arrows) and HMBC (red arrows) interactions of 2-isobutyryloxythymyl 2-methylbutyrate.

Figure S15. Mass spectrum (EI, 70 eV) of 2-(2-methylbutyryloxy)thymyl 2-methylbutyrate.

Figure S16. Mass spectrum (EI, 70 eV) of 2-isovaleroxythymyl isobutyrate.

Figure S17. Mass spectrum (EI, 70 eV) of 2-isobutyryloxythymyl isovalerate.

Figure S18. IR spectrum of the mixture of 2-isovaleroxythymyl isobutyrate and 2-isobutyryloxythymyl isovalerate.

Figure S19. ^{1}H NMR (400 MHz, $CDCl_3$) spectrum of the mixture of 2-isovaleroxythymyl isobutyrate (y) and 2-isobutyryloxythymyl isovalerate (x) and the corresponding expansions with signal assignment.

Figure S20. Simulated (manual iterative full spin, MestreNova 11.0.3) and experimental ^{1}H NMR spectrum of the mixture of 2-isovaleroxythymyl isobutyrate (y) and 2-isobutyryloxythymyl isovalerate (x).

Figure S21. ^{13}C NMR (100.6 MHz, $CDCl_3$) spectrum of the mixture of 2-isovaleroxythymyl isobutyrate (y) and 2-isobutyryloxythymyl isovalerate (x).

Table S4. ^{1}H (400 MHz) and ^{13}C (100.6 MHz) NMR data of 2-isovaleroxythymyl isobutyrate (chloroform-*d*); NMR parameters are derived from manual iterative full spin analysis, along with the observed gHMBC and NOESY correlations.

Figure S22. NOESY (blue arrows) and HMBC (red arrows) interactions of 2-isovaleroxythymyl isobutyrate.

Table S5. ^{1}H (400 MHz) and ^{13}C (100.6 MHz) NMR data of 2-isobutyryloxythymyl isovalerate (chloroform-*d*); NMR parameters are derived from manual iterative full spin analysis, along with the observed gHMBC and NOESY correlations.

Figure S23. NOESY (blue arrows) and HMBC (red arrows) interactions of 2-isobutyryloxythymyl isovalerate.

Figure S24. Mass spectrum (EI, 70 eV) of the mixture of 2-isovaleroxythymyl 2-methylbutyrate and 2-(2-methylbutyryloxy)thymyl isovalerate.

Figure S25. IR spectrum of the mixture of 2-isovaleroxythymyl 2-methylbutyrate and 2-(2-methylbutyryloxy)thymyl isovalerate.

Figure S26. ^{1}H NMR (400 MHz, $CDCl_3$) spectrum of the mixture of 2-isovaleroxythymyl 2-methylbutyrate (x) and 2-(2-methylbutyryloxy)thymyl isovalerate (y) and the corresponding expansions with signal assignment.

Figure S27. Simulated (manual iterative full spin, MestreNova 11.0.3) and experimental ^{1}H NMR spectrum of the mixture of 2-isovaleroxythymyl 2-methylbutyrate (x) and 2-(2-methylbutyryloxy)thymyl isovalerate (y).

Figure S28. ^{13}C NMR (100.6 MHz, $CDCl_3$) spectrum of the mixture of 2-isovaleroxythymyl 2-methylbutyrate (x) and 2-(2-methylbutyryloxy)thymyl isovalerate (y).

Table S6. ^{1}H (400 MHz) and ^{13}C (100.6 MHz) NMR data of 2-isovaleroxythymyl 2-methylbutyrate (chloroform-*d*); NMR parameters are derived from manual iterative full spin analysis, along with the observed gHMBC and NOESY correlations.

Figure S29. NOESY (blue arrows) and HMBC (red arrows) interactions of 2-isovaleroxythymyl 2-methylbutyrate.

Table S7. ^{1}H (400 MHz) and ^{13}C (100.6 MHz) NMR data of 2-(2-methylbutyryloxy)thymyl isovalerate (chloroform-*d*); NMR parameters are derived from manual iterative full spin analysis, along with the observed gHMBC and NOESY correlations.

Figure S30. NOESY (blue arrows) and HMBC (red arrows) interactions of 2-(2-methylbutyryloxy)thymyl isovalerate.

Figure S31. Mass spectrum (EI, 70 eV) of 2-isovaleroxythymyl isovalerate.

Scheme S2. Synthetic pathway to 5-hydroxythymol-derived monoesters and diesters (R,R′ – isopropyl, *sec*-butyl, or isobutyl).

Figure S32. Mass spectrum (EI, 70 eV) of 5-isobutyryloxythymyl isobutyrate.

Figure S33. Mass spectrum (EI, 70 eV) of 5-isobutyryloxythymyl 2-methylbutyrate.

Figure S34. Mass spectrum (EI, 70 eV) of 5-isobutyryloxythymyl isovalerate.

Figure S35. Mass spectrum (EI, 70 eV) of 5-(2-methylbutyryloxy)thymyl 2-methylbutyrate.

Figure S36. Mass spectrum (EI, 70 eV) of 5-(2-methylbutyryloxy)thymyl isovalerate.

Figure S37. Mass spectrum (EI, 70 eV) of 5-isovaleroxythymyl isovalerate.

Figure S38. IR spectrum of 5-isovaleroxythymyl isovalerate.

Figure S39. ^{1}H NMR (400 MHz, $CDCl_3$) spectrum of 5-isovaleroxythymyl isovalerate and the corresponding expansions with signal assignment.

Figure S40. Simulated (manual iterative full spin, MestreNova 11.0.3) and experimental ^{1}H NMR spectrum of 5-isovaleroxythymyl isovalerate.

Figure S41. ^{13}C NMR (100.6 MHz, $CDCl_3$) spectrum of 5-isovaleroxythymyl isovalerate.

Table S8. ^{1}H (400 MHz) and ^{13}C (100.6 MHz) NMR data of 5-isovaleroxythymyl isovalerate (chloroform-*d*); NMR parameters are derived from manual iterative full spin analysis, along with the observed gHMBC and NOESY correlations.

Figure S42. NOESY (blue arrows) and HMBC (red arrows) interactions of 5-isovaleroxythymyl isovalerate.

Scheme S3. Synthetic pathway to 6-hydroxythymol-derived monoesters and diesters (R,R′ – isopropyl, *sec*-butyl, or isobutyl).

Figure S43. Mass spectrum (EI, 70 eV) of 6-isobutyryloxythymyl isobutyrate.

Figure S44. IR spectrum of 6-isobutyryloxythymyl isobutyrate.

Figure S45. ^{1}H NMR (400 MHz, $CDCl_3$) spectrum of 6-isobutyryloxythymyl isobutyrate and the corresponding expansions with signal assignment.

Figure S46. Simulated (manual iterative full spin, MestreNova 11.0.3) and experimental ^{1}H NMR spectrum of 6-isobutyryloxythymyl isobutyrate.

Figure S47. ^{13}C NMR (100.6 MHz, $CDCl_3$) spectrum of 6-isobutyryloxythymyl isobutyrate.

Table S9. ^{1}H (400 MHz) and ^{13}C (100.6 MHz) NMR data of 6-isobutyryloxythymyl isobutyrate (chloroform-*d*); NMR parameters are derived from manual iterative full spin analysis, along with the observed gHMBC and NOESY correlations.

Figure S48. NOESY (blue arrows) and HMBC (red arrows) interactions of 6-isobutyryloxythymyl isobutyrate.

Figure S49. Mass spectrum (EI, 70 eV) of 6-(2-methylbutyryloxy)thymyl isobutyrate.

Figure S50. IR spectrum of 6-(2-methylbutyryloxy)thymyl isobutyrate.

Figure S51. ^{1}H NMR (400 MHz, $CDCl_3$) spectrum of 6-(2-methylbutyryloxy)thymyl isobutyrate and the corresponding expansions with signal assignment.

Figure S52. Simulated (manual iterative full spin, MestreNova 11.0.3) and experimental ^{1}H NMR spectrum of 6-(2-methylbutyryloxy)thymyl isobutyrate.

Figure S53. ^{13}C NMR (100.6 MHz, $CDCl_3$) spectrum of 6-(2-methylbutyryloxy)thymyl isobutyrate.

Table S10. ^{1}H (400 MHz) and ^{13}C (100.6 MHz) NMR data of 6-(2-methylbutyryloxy)thymyl isobutyrate (chloroform-*d*); NMR parameters are derived from manual iterative full spin analysis, along with the observed gHMBC and NOESY correlations.

Figure S54. NOESY (blue arrows) and HMBC (red arrows) interactions of 6-(2-methylbutyryloxy)thymyl isobutyrate.

Figure S55. Mass spectrum (EI, 70 eV) of 6-isobutyryloxythymyl 2-methylbutyrate.

Figure S56. IR spectrum of 6-isobutyryloxythymyl 2-methylbutyrate.

Figure S57. ^{1}H NMR (400 MHz, $CDCl_3$) spectrum of 6-isobutyryloxythymyl 2-methylbutyrate and the corresponding expansions with signal assignment.

Figure S58. Simulated (manual iterative full spin, MestreNova 11.0.3) and experimental ^{1}H NMR spectrum of 6-isobutyryloxythymyl 2-methylbutyrate.

Figure S59. ^{13}C NMR (100.6 MHz, $CDCl_3$) spectrum of 6-isobutyryloxythymyl 2-methylbutyrate.

Table S11. ^{1}H (400 MHz) and ^{13}C (100.6 MHz) NMR data of 6-isobutyryloxythymyl 2-methylbutyrate (chloroform-*d*); NMR parameters are derived from manual iterative full spin analysis, along with the observed gHMBC and NOESY correlations.

Figure S60. NOESY (blue arrows) and HMBC (red arrows) interactions of 6-isobutyryloxythymyl 2-methylbutyrate.

Figure S61. Mass spectrum (EI, 70 eV) of 6-isovaleroxythymyl isobutyrate.

Figure S62. Mass spectrum (EI, 70 eV) of 6-isobutyryloxythymyl isovalerate.

Figure S63. Mass spectrum (EI, 70 eV) of 6-(2-methylbutyryloxy)thymyl 2-methylbutyrate.

Figure S64. IR spectrum of 6-(2-methylbutyryloxy)thymyl 2-methylbutyrate.

Figure S65. ^{1}H NMR (400 MHz, $CDCl_3$) spectrum of 6-(2-methylbutyryloxy)thymyl 2-methylbutyrate and the corresponding expansions with signal assignment.

Figure S66. Simulated (manual iterative full spin, MestreNova 11.0.3) and experimental ^{1}H NMR spectrum of 6-(2-methylbutyryloxy)thymyl 2-methylbutyrate.

Figure S67. ^{13}C NMR (100.6 MHz, $CDCl_3$) spectrum of 6-(2-methylbutyryloxy)thymyl 2-methylbutyrate.

Table S12. ^{1}H (400 MHz) and ^{13}C (100.6 MHz) NMR data of 6-(2-methylbutyryloxy)thymyl 2-methylbutyrate (chloroform-*d*); NMR parameters are derived from manual iterative full spin analysis, along with the observed gHMBC and NOESY correlations.

Figure S68. NOESY (blue arrows) and HMBC (red arrows) interactions of 6-(2-methylbutyryloxy)thymyl 2-methylbutyrate.

Figure S69. Mass spectrum (EI, 70 eV) of 6-(2-methylbutyryloxy)thymyl isovalerate.

Figure S70. Mass spectrum (EI, 70 eV) of 6-isovaleroxythymyl 2-methylbutyrate.

Figure S71. Mass spectrum (EI, 70 eV) of 6-isovaleroxythymyl isovalerate.

Scheme S4. Synthetic pathway to 7-hydroxythymol-derived monoesters and diesters (R,R′ – isopropyl, *sec*-butyl, or isobutyl).

Figure S72. Mass spectrum (EI, 70 eV) of 7-isobutyryloxythymyl isobutyrate.

Figure S73. IR spectrum of 7-isobutyryloxythymyl isobutyrate.

Figure S74. ^{1}H NMR (400 MHz, $CDCl_3$) spectrum of 7-isobutyryloxythymyl isobutyrate and the corresponding expansions with signal assignment.

Figure S75. Simulated (manual iterative full spin, MestreNova 11.0.3) and experimental ^{1}H NMR spectrum of 7-isobutyryloxythymyl isobutyrate.

Figure S76. ^{13}C NMR (100.6 MHz, $CDCl_3$) spectrum of 7-isobutyryloxythymyl isobutyrate.

Table S13. ^{1}H (400 MHz) and ^{13}C (100.6 MHz) NMR data of 7-isobutyryloxythymyl isobutyrate (chloroform-*d*); NMR parameters are derived from manual iterative full spin analysis, along with the observed gHMBC and NOESY correlations.

Figure S77. NOESY (blue arrows) and HMBC (red arrows) interactions of 7-isobutyryloxythymyl isobutyrate.

Figure S78. Mass spectrum (EI, 70 eV) of 7-(2-methylbutyryloxy)thymyl isobutyrate.

Figure S79. IR spectrum of 7-(2-methylbutyryloxy)thymyl isobutyrate.

Figure S80. ^{1}H NMR (400 MHz, $CDCl_3$) spectrum of 7-(2-methylbutyryloxy)thymyl isobutyrate and the corresponding expansions with signal assignment.

Figure S81. Simulated (manual iterative full spin, MestreNova 11.0.3) and experimental ^{1}H NMR spectrum of 7-(2-methylbutyryloxy)thymyl isobutyrate.

Figure S82. ^{13}C NMR (100.6 MHz, $CDCl_3$) spectrum of 7-(2-methylbutyryloxy)thymyl isobutyrate.

Table S14. ^{1}H (400 MHz) and ^{13}C (100.6 MHz) NMR data of 7-(2-methylbutyryloxy)thymyl isobutyrate (chloroform-*d*); NMR parameters are derived from manual iterative full spin analysis, along with the observed gHMBC and NOESY correlations.

Figure S83. NOESY (blue arrows) and HMBC (red arrows) interactions of 7-(2-methylbutyryloxy)thymyl isobutyrate.

Figure S84. Mass spectrum (EI, 70 eV) of 7-isobutyryloxythymyl 2-methylbutyrate.

Figure S85. IR spectrum of 7-isobutyryloxythymyl 2-methylbutyrate.

Figure S86. ^{1}H NMR (400 MHz, $CDCl_3$) spectrum of 7-isobutyryloxythymyl 2-methylbutyrate and the corresponding expansions with signal assignment.

Figure S87. Simulated (manual iterative full spin, MestreNova 11.0.3) and experimental ^{1}H NMR spectrum of 7-isobutyryloxythymyl 2-methylbutyrate.

Figure S88. ^{13}C NMR (100.6 MHz, $CDCl_3$) spectrum of 7-isobutyryloxythymyl 2-methylbutyrate.

Table S15. ^{1}H (400 MHz) and ^{13}C (100.6 MHz) NMR data of 7-isobutyryloxythymyl 2-methylbutyrate (chloroform-*d*); NMR parameters are derived from manual iterative full spin analysis, along with the observed gHMBC and NOESY correlations.

Figure S89. NOESY (blue arrows) and HMBC (red arrows) interactions of 7-isobutyryloxythymyl 2-methylbutyrate.

Figure S90. Mass spectrum (EI, 70 eV) of 7-(2-methylbutyryloxy)thymyl 2-methylbutyrate.

Figure S91. IR spectrum of 7-(2-methylbutyryloxy)thymyl 2-methylbutyrate.

Figure S92. ^{1}H NMR (400 MHz, $CDCl_3$) spectrum of 7-(2-methylbutyryloxy)thymyl 2-methylbutyrate and the corresponding expansions with signal assignment.

Figure S93. Simulated (manual iterative full spin, MestreNova 11.0.3) and experimental ^{1}H NMR spectrum of 7-(2-methylbutyryloxy)thymyl 2-methylbutyrate.

Figure S94. ^{13}C NMR (100.6 MHz, $CDCl_3$) spectrum of 7-(2-methylbutyryloxy)thymyl 2-methylbutyrate.

Table S16. ^{1}H (400 MHz) and ^{13}C (100.6 MHz) NMR data of 7-(2-methylbutyryloxy)thymyl 2-methylbutyrate (chloroform-*d*); NMR parameters are derived from manual iterative full spin analysis, along with the observed gHMBC and NOESY correlations.

Figure S95. NOESY (blue arrows) and HMBC (red arrows) interactions of 7-(2-methylbutyryloxy)thymyl 2-methylbutyrate.

Figure S96. Mass spectrum (EI, 70 eV) of 7-isovaleroxythymyl isobutyrate.

Figure S97. IR spectrum of 7-isovaleroxythymyl isobutyrate.

Figure S98. ^{1}H NMR (400 MHz, $CDCl_3$) spectrum of 7-isovaleroxythymyl isobutyrate and the corresponding expansions with signal assignment.

Figure S99. Simulated (manual iterative full spin, MestreNova 11.0.3) and experimental ^{1}H NMR spectrum of 7-isovaleroxythymyl isobutyrate.

Figure S100. ^{13}C NMR (100.6 MHz, $CDCl_3$) spectrum of 7-isovaleroxythymyl isobutyrate.

Table S17. ^{1}H (400 MHz) and ^{13}C (100.6 MHz) NMR data of 7-isovaleroxythymyl isobutyrate (chloroform-*d*); NMR parameters are derived from manual iterative full spin analysis, along with the observed gHMBC and NOESY correlations.

Figure S101. NOESY (blue arrows) and HMBC (red arrows) interactions of 7-isovaleroxythymyl isobutyrate.

Figure S102. Mass spectrum (EI, 70 eV) of 7-isobutyryloxythymyl isovalerate.

Figure S103. IR spectrum of 7-isobutyryloxythymyl isovalerate.

Figure S104. ^{1}H NMR (400 MHz, $CDCl_3$) spectrum of 7-isobutyryloxythymyl isovalerate and the corresponding expansions with signal assignment.

Figure S105. Simulated (manual iterative full spin, MestreNova 11.0.3) and experimental ^{1}H NMR spectrum of 7-isobutyryloxythymyl isovalerate.

Figure S106. ^{13}C NMR (100.6 MHz, $CDCl_3$) spectrum of 7-isobutyryloxythymyl isovalerate.

Table S18. ^{1}H (400 MHz) and ^{13}C (100.6 MHz) NMR data of 7-isobutyryloxythymyl isovalerate (chloroform-*d*); NMR parameters are derived from manual iterative full spin analysis, along with the observed gHMBC and NOESY correlations.

Figure S107. NOESY (blue arrows) and HMBC (red arrows) interactions of 7-isobutyryloxythymyl isovalerate.

Figure S108. Mass spectrum (EI, 70 eV) of 7-(2-methylbutyryloxy)thymyl isovalerate.

Figure S109. IR spectrum of 7-(2-methylbutyryloxy)thymyl isovalerate.

Figure S110. ^{1}H NMR (400 MHz, $CDCl_3$) spectrum of 7-(2-methylbutyryloxy)thymyl isovalerate and the corresponding expansions with signal assignment.

Figure S111. Simulated (manual iterative full spin, MestreNova 11.0.3) and experimental ^{1}H NMR spectrum of 7-(2-methylbutyryloxy)thymyl isovalerate.

Figure S112. ^{13}C NMR (100.6 MHz, $CDCl_3$) spectrum of 7-(2-methylbutyryloxy)thymyl isovalerate.

Table S19. ^{1}H (400 MHz) and ^{13}C (100.6 MHz) NMR data of 7-(2-methylbutyryloxy)thymyl isovalerate (chloroform-*d*); NMR parameters are derived from manual iterative full spin analysis, along with the observed gHMBC and NOESY correlations.

Figure S113. NOESY (blue arrows) and HMBC (red arrows) interactions of 7-(2-methylbutyryloxy)thymyl isovalerate.

Figure S114. Mass spectrum (EI, 70 eV) of 7-isovaleroxythymyl 2-methylbutyrate.

Figure S115. IR spectrum of 7-isovaleroxythymyl 2-methylbutyrate.

Figure S116. ^{1}H NMR (400 MHz, $CDCl_3$) spectrum of 7-isovaleroxythymyl 2-methylbutyrate and the corresponding expansions with signal assignment.

Figure S117. Simulated (manual iterative full spin, MestreNova 11.0.3) and experimental ^{1}H NMR spectrum of 7-isovaleroxythymyl 2-methylbutyrate.

Figure S118. ^{13}C NMR (100.6 MHz, $CDCl_3$) spectrum of 7-isovaleroxythymyl 2-methylbutyrate.

Table S20. ^{1}H (400 MHz) and ^{13}C (100.6 MHz) NMR data of 7-isovaleroxythymyl 2-methylbutyrate (chloroform-*d*); NMR parameters are derived from manual iterative full spin analysis, along with the observed gHMBC and NOESY correlations.

Figure S119. NOESY (blue arrows) and HMBC (red arrows) interactions of 7-isovaleroxythymyl 2-methylbutyrate.

Figure S120. Mass spectrum (EI, 70 eV) of 7-isovaleroxythymyl isovalerate.

Figure S121. IR spectrum of 7-isovaleroxythymyl isovalerate.

Figure S122. ^{1}H NMR (400 MHz, $CDCl_3$) spectrum of 7-isovaleroxythymyl isovalerate and the corresponding expansions with signal assignment.

Figure S123. Simulated (manual iterative full spin, MestreNova 11.0.3) and experimental ^{1}H NMR spectrum of 7-isovaleroxythymyl isovalerate.

Figure S124. ^{13}C NMR (100.6 MHz, $CDCl_3$) spectrum of 7-isovaleroxythymyl isovalerate.

Table S21. ^{1}H (400 MHz) and ^{13}C (100.6 MHz) NMR data of 7-isovaleroxythymyl isovalerate (chloroform-*d*); NMR parameters are derived from manual iterative full spin analysis, along with the observed gHMBC and NOESY correlations.

Figure S125. NOESY (blue arrows) and HMBC (red arrows) interactions of 7-isovaleroxythymyl isovalerate.

Scheme S5. Synthetic pathway to 8-hydroxythymol-derived monoesters and diesters (R,R′ – isopropyl, *sec*-butyl, or isobutyl).

Figure S126. Mass spectrum (EI, 70 eV) of 8-isobutyryloxythymyl isobutyrate.

Figure S127. ^{1}H NMR (400 MHz, $CDCl_3$) spectrum of 8-isobutyryloxythymyl isobutyrate and the corresponding expansions with signal assignment.

Figure S128. Simulated (manual iterative full spin, MestreNova 11.0.3) and experimental ^{1}H NMR spectrum of 8-isobutyryloxythymyl isobutyrate.

Figure S129. ^{13}C NMR (100.6 MHz, $CDCl_3$) spectrum of 8-isobutyryloxythymyl isobutyrate.

Table S22. ^{1}H (400 MHz) and ^{13}C (100.6 MHz) NMR data of 8-isobutyryloxythymyl isobutyrate (chloroform-*d*); NMR parameters are derived from manual iterative full spin analysis, along with the observed gHMBC and NOESY correlations.

Figure S130. NOESY (blue arrows) and HMBC (red arrows) interactions of 8-isobutyryloxythymyl isobutyrate.

Figure S131. Mass spectrum (EI, 70 eV) of 8-(2-methylbutyryloxy)thymyl isobutyrate.

Figure S132. Mass spectrum (EI, 70 eV) of 8-isobutyryloxythymyl 2-methylbutyrate.

Figure S133. Mass spectrum (EI, 70 eV) of 8-(2-methylbutyryloxy)thymyl 2-methylbutyrate.

Figure S134. Mass spectrum (EI, 70 eV) of 8-isobutyryloxythymyl isovalerate.

Figure S135. Mass spectrum (EI, 70 eV) of 8-isovaleroxythymyl isobutyrate.

Figure S136. Mass spectrum (EI, 70 eV) of 8-isovaleroxythymyl isovalerate.

Scheme S6. Synthetic pathway to 9-hydroxythymol-derived monoesters and diesters (R,R′ – isopropyl, *sec*-butyl, or isobutyl).

Figure S137. Mass spectrum (EI, 70 eV) of 9-isobutyryloxythymyl isobutyrate.

Figure S138. IR spectrum of 9-isobutyryloxythymyl isobutyrate.

Figure S139. ^{1}H NMR (400 MHz, $CDCl_3$) spectrum of 9-isobutyryloxythymyl isobutyrate and the corresponding expansions with signal assignment.

Figure S140. Simulated (manual iterative full spin, MestreNova 11.0.3) and experimental ^{1}H NMR spectrum of 9-isobutyryloxythymyl isobutyrate.

Figure S141. ^{13}C NMR (100.6 MHz, $CDCl_3$) spectrum of 9-isobutyryloxythymyl isobutyrate.

Table S23. ^{1}H (400 MHz) and ^{13}C (100.6 MHz) NMR data of 9-isobutyryloxythymyl isobutyrate (chloroform-*d*); NMR parameters are derived from manual iterative full spin analysis, along with the observed gHMBC and NOESY correlations.

Figure S142. NOESY (blue arrows) and HMBC (red arrows) interactions of 9-isobutyryloxythymyl isobutyrate.

Figure S143. Mass spectrum (EI, 70 eV) of 9-(2-methylbutyryloxy)thymyl isobutyrate.

Figure S144. IR spectrum of 9-(2-methylbutyryloxy)thymyl isobutyrate.

Figure S145. ^{1}H NMR (400 MHz, $CDCl_3$) spectrum of 9-(2-methylbutyryloxy)thymyl isobutyrate and the corresponding expansions with signal assignment.

Figure S146. Simulated (manual iterative full spin, MestreNova 11.0.3) and experimental ^{1}H NMR spectrum of 9-(2-methylbutyryloxy)thymyl isobutyrate.

Figure S147. ^{13}C NMR (100.6 MHz, $CDCl_3$) spectrum of 9-(2-methylbutyryloxy)thymyl isobutyrate.

Table S24. ^{1}H (400 MHz) and ^{13}C (100.6 MHz) NMR data of diastereomeric mixture of 9-(2-methylbutyryloxy)thymyl isobutyrate (chloroform-*d*); NMR parameters are derived from manual iterative full spin analysis, along with the observed gHMBC and NOESY correlations.

Figure S148. NOESY (blue arrows) and HMBC (red arrows) interactions of 9-(2-methylbutyryloxy)thymyl isobutyrate.

Figure S149. Mass spectrum (EI, 70 eV) of 9-isobutyryloxythymyl 2-methylbutyrate.

Figure S150. IR spectrum of 9-isobutyryloxythymyl 2-methylbutyrate.

Figure S151. ^{1}H NMR (400 MHz, $CDCl_3$) spectrum of 9-isobutyryloxythymyl 2-methylbutyrate and the corresponding expansions with signal assignment.

Figure S152. Simulated (manual iterative full spin, MestreNova 11.0.3) and experimental ^{1}H NMR spectrum of 9-isobutyryloxythymyl 2-methylbutyrate.

Figure S153. ^{13}C NMR (100.6 MHz, $CDCl_3$) spectrum of 9-isobutyryloxythymyl 2-methylbutyrate.

Table S25. ^{1}H (400 MHz) and ^{13}C (100.6 MHz) NMR data of diastereomeric mixture of 9-isobutyryloxythymyl 2-methylbutyrate (chloroform-*d*); NMR parameters are derived from manual iterative full spin analysis, along with the observed gHMBC and NOESY correlations.

Figure S154. NOESY (blue arrows) and HMBC (red arrows) interactions of 9-isobutyryloxythymyl 2-methylbutyrate.

Figure S155. Mass spectrum (EI, 70 eV) of 9-(2-methylbutyryloxy)thymyl 2-methylbutyrate.

Figure S156. IR spectrum of 9-(2-methylbutyryloxy)thymyl 2-methylbutyrate.

Figure S157. ^{1}H NMR (400 MHz, $CDCl_3$) spectrum of 9-(2-methylbutyryloxy)thymyl 2-methylbutyrate and the corresponding expansions with signal assignment.

Figure S158. Simulated (manual iterative full spin, MestreNova 11.0.3) and experimental ^{1}H NMR spectrum of 9-(2-methylbutyryloxy)thymyl 2-methylbutyrate.

Figure S159. ^{13}C NMR (100.6 MHz, $CDCl_3$) spectrum of 9-(2-methylbutyryloxy)thymyl 2-methylbutyrate.

Table S26. ^{1}H (400 MHz) and ^{13}C (100.6 MHz) NMR data of diastereomeric mixture of 9-(2-methylbutyryloxy)thymyl 2-methylbutyrate (chloroform-*d*); NMR parameters are derived from manual iterative full spin analysis, along with the observed gHMBC and NOESY correlations.

Figure S160. NOESY (blue arrows) and HMBC (red arrows) interactions of 9-(2-methylbutyryloxy)thymyl 2-methylbutyrate.

Figure S161. Mass spectrum (EI, 70 eV) of 9-isovaleroxythymyl isobutyrate.

Figure S162. Mass spectrum (EI, 70 eV) of 9-isobutyryloxythymyl isovalerate.

Figure S163. Mass spectrum (EI, 70 eV) of 9-(2-methylbutyryloxy)thymyl isovalerate.

Figure S164. Mass spectrum (EI, 70 eV) of 9-isovaleroxythymyl 2-methylbutyrate.

Figure S165. Mass spectrum (EI, 70 eV) of 9-isovaleroxythymyl isovalerate.

Table S27. Retention indices of model esters.

Table S28. Residual retention-index increments obtained by subtracting model ester RI values from the corresponding thymol diester RI values.

Table S29. Deviations of calculated residual retention-index increments from the mean residual increments for each regioisomeric series. $\delta\Delta RI = \Delta RIindividual - \overline{\Delta RIa}$. Positive values indicate residual increments higher than the corresponding mean value, whereas negative values indicate lower-than-average values.

Figure S166. Correlation between retention indices determined on the non-polar DB-5MS column (RIa) and the polar HP-Innowax column (RIp) for the synthesized diacylated oxygenated thymol derivatives. Panels show the corresponding regioisomeric series: (**A**) 2-acyloxythymyl esters, (**B**) 5-acyloxythymyl esters, (**C**) 6-acyloxythymyl esters, (**D**) 7-acyloxythymyl esters, (**E**) 8-acyloxythymyl esters, and (**F**) 9-acyloxythymyl esters. Linear regression equations and R^2 values are shown in each panel. The number of data points was n = 9 for the 2-, 6-, 7-, and 9-substituted series, n = 7 for the 8-substituted series, and n = 6 for the 5-substituted series when symmetry-equivalent compounds were counted once.

Figure S167. Pairwise correlations of retention indices determined on the non-polar DB-5MS column (RIa) for homologous 5-, 6-, 7-, and 9-substituted diacylated thymol derivatives. Panels show correlations between the corresponding regioisomeric series: (A) 5- vs 6-substituted derivatives, (B) 5- vs 7-substituted derivatives, (C) 6- vs 7-substituted derivatives, (D) 5- vs 9-substituted derivatives, (E) 6- vs 9-substituted derivatives, and (F) 7- vs 9-substituted derivatives. Only compounds with corresponding acyl-residue combinations in both compared series were included. Because the 5-substituted series contains symmetry-equivalent C-3/C-5 combinations, these were treated as equivalent rather than as independent duplicate

observations; therefore, each pairwise regression was based on n = 6 data points. Linear regression equations and coefficients of determination (R^2) are shown in each panel.

Figure S168. Pairwise correlations of retention indices determined on the polar HP-Innowax column (RIp) for homologous 5-, 6-, 7-, and 9-substituted diacylated thymol derivatives. Panels show correlations between the corresponding regioisomeric series: (**A**) 5- vs 6-substituted derivatives, (**B**) 5- vs 7-substituted derivatives, (**C**) 6- vs 7-substituted derivatives, (**D**) 5- vs 9-substituted derivatives, (**E**) 6- vs 9-substituted derivatives, and (**F**) 7- vs 9-substituted derivatives. Only compounds with corresponding acyl-residue combinations in both compared series were included. Because the 5-substituted series contains symmetry-equivalent C-3/C-5 combinations, these were treated as equivalent rather than as independent duplicate observations; therefore, each pairwise regression was based on n = 6 data points. Linear regression equations and coefficients of determination (R^2) are shown in each panel.

Figure S169. Representative co-injection chromatograms of selected diacylated oxygenated thymol derivatives in the more polar fraction of *Doronicum columnae* root essential oil on the non-polar DB-5MS column. The black trace represents an enlarged total ion chromatogram of the original fraction in the 24–31 min retention-time region, whereas the red and blue traces represent stacked chromatograms obtained after co-injection of the indicated synthetic reference compounds or reference GC–MS components. Peak labels correspond to the compound codes used throughout the manuscript. Co-injection was considered confirmatory when spiking increased only the corresponding chromatographic peak, without formation of a shoulder or secondary peak.

Figure S170. Representative co-injection chromatograms of selected diacylated oxygenated thymol derivatives in the more polar fraction of *Doronicum columnae* root essential oil on the polar HP-Innowax column. The black trace represents an enlarged total ion chromatogram of the original fraction in the 33–39 min retention-time region, whereas the red and blue traces represent stacked chromatograms obtained after co-injection of the indicated synthetic reference compounds or reference GC–MS components. Peak labels correspond to the compound codes used throughout the manuscript. Co-injection was considered confirmatory when spiking increased only the corresponding chromatographic peak, without formation of a shoulder or secondary peak.

Figure S171. Expanded experimental DB-5MS chromatograms illustrating the identification of 6-iPr-3-sBu and 6-sBu-3-iPr in the more polar fraction of *D. columnae* root essential oil. The black trace represents the unspiked fraction, whereas the blue and red traces represent separate co-injection experiments with 6-iPr-

3-sBu and 6-sBu-3-iPr, respectively. The individual additions enhance the corresponding shoulders already present before the larger peak assigned to 7-sBu-3-iPr. The components partially overlap, and baseline separation was not achieved. Normalized traces are vertically offset for clarity. Compound codes correspond to those used throughout the manuscript.

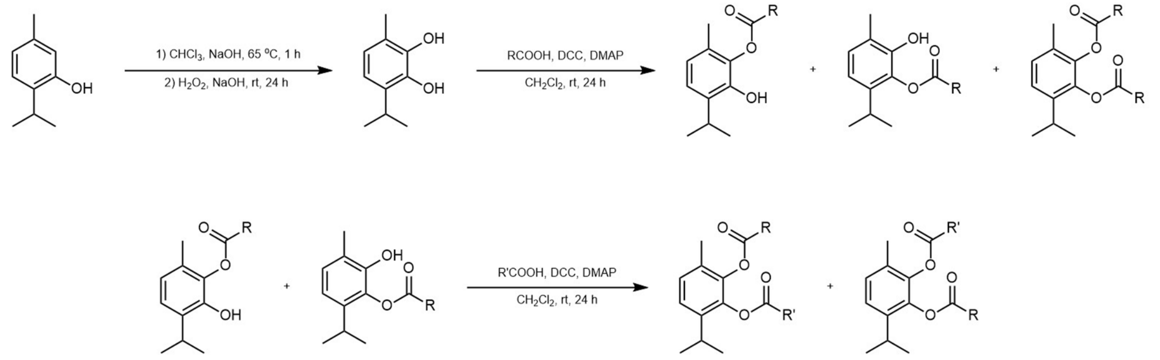

Scheme S1. Synthetic pathway to 2-hydroxythymol-derived monoesters and diesters (R,R' – isopropyl, *sec*-butyl, or isobutyl).

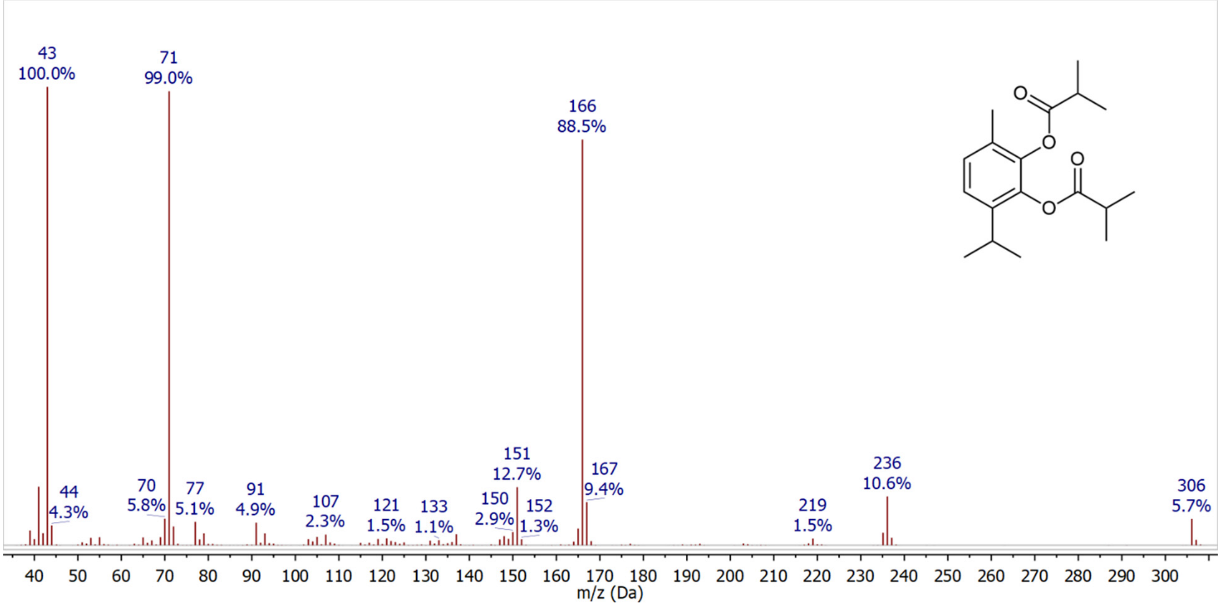

Figure S1. Mass spectrum (EI, 70 eV) of 2-isobutyryloxythymyl isobutyrate.

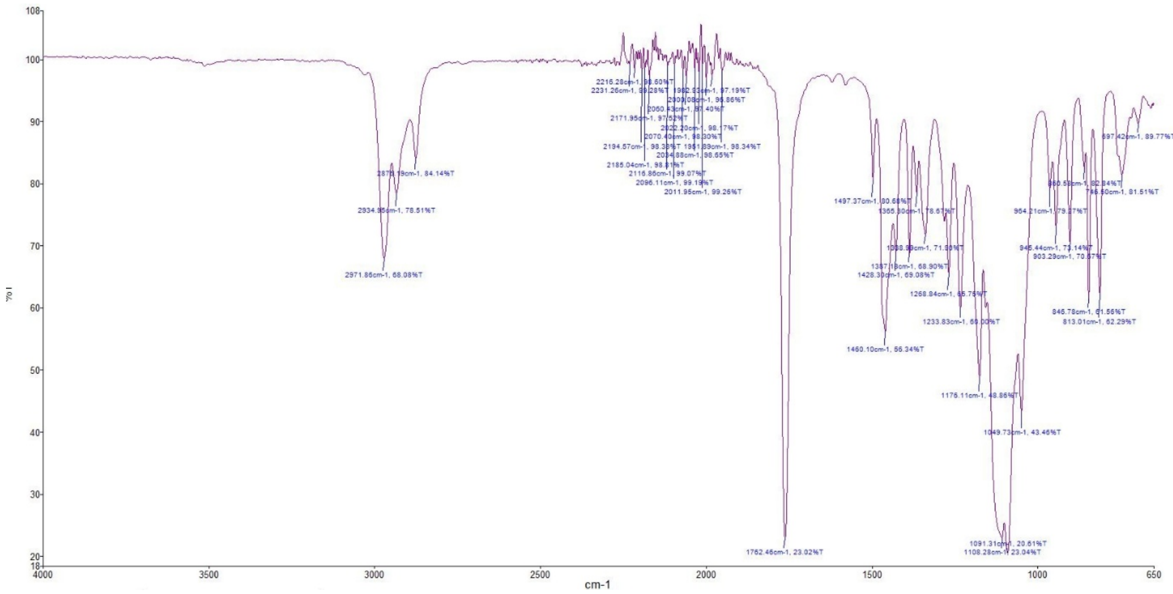

Figure S2. IR spectrum of 2-isobutyryloxythymyl isobutyrate.

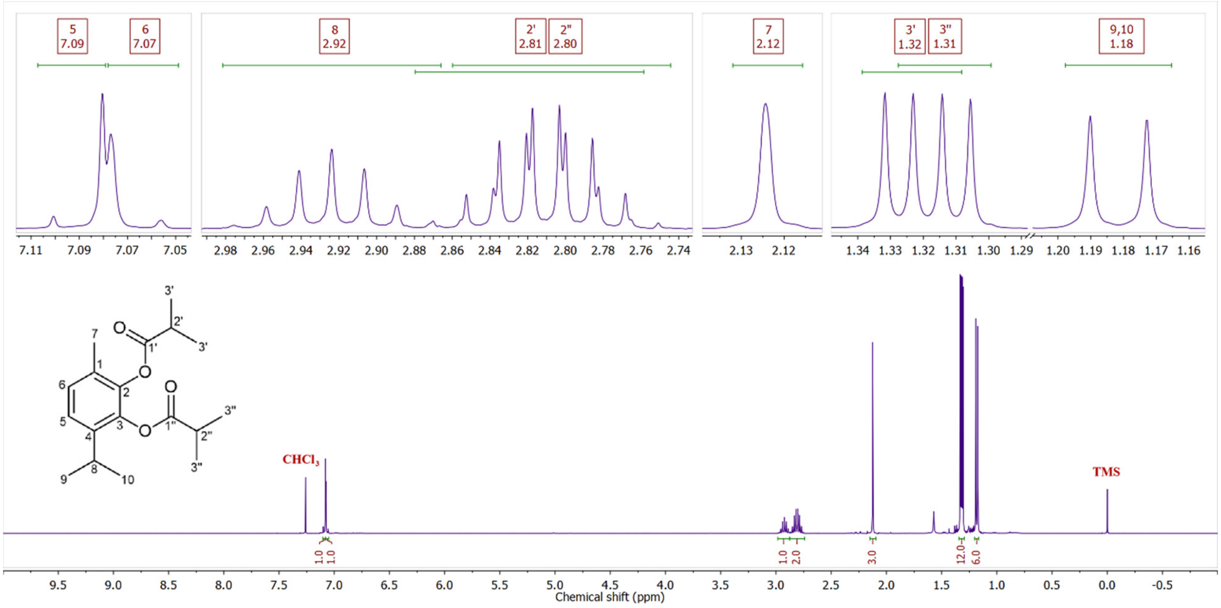

Figure S3. ^{1}H NMR (400 MHz, $CDCl_3$) spectrum of 2-isobutyryloxythymyl isobutyrate and the corresponding expansions with signal assignment.

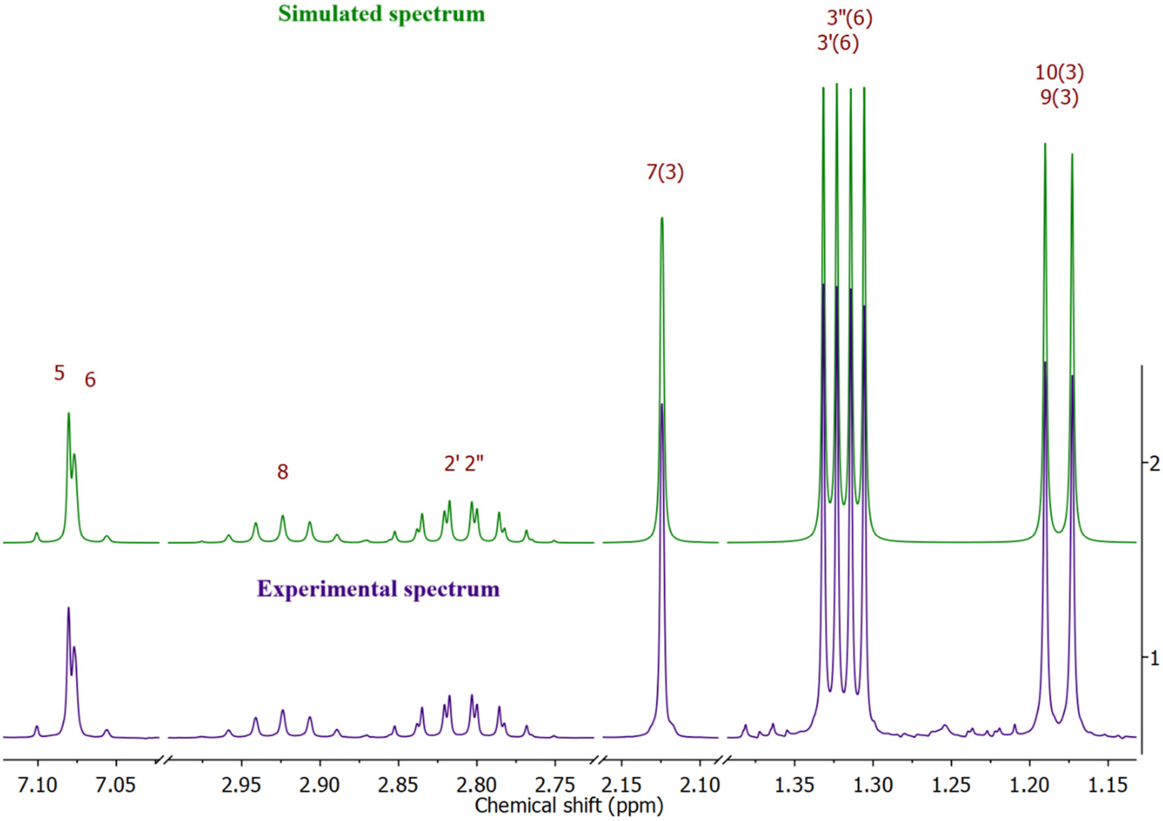

Figure S4. Simulated (manual iterative full spin, MestreNova 11.0.3) and experimental ¹H NMR spectrum of 2-isobutyryloxythymyl isobutyrate.

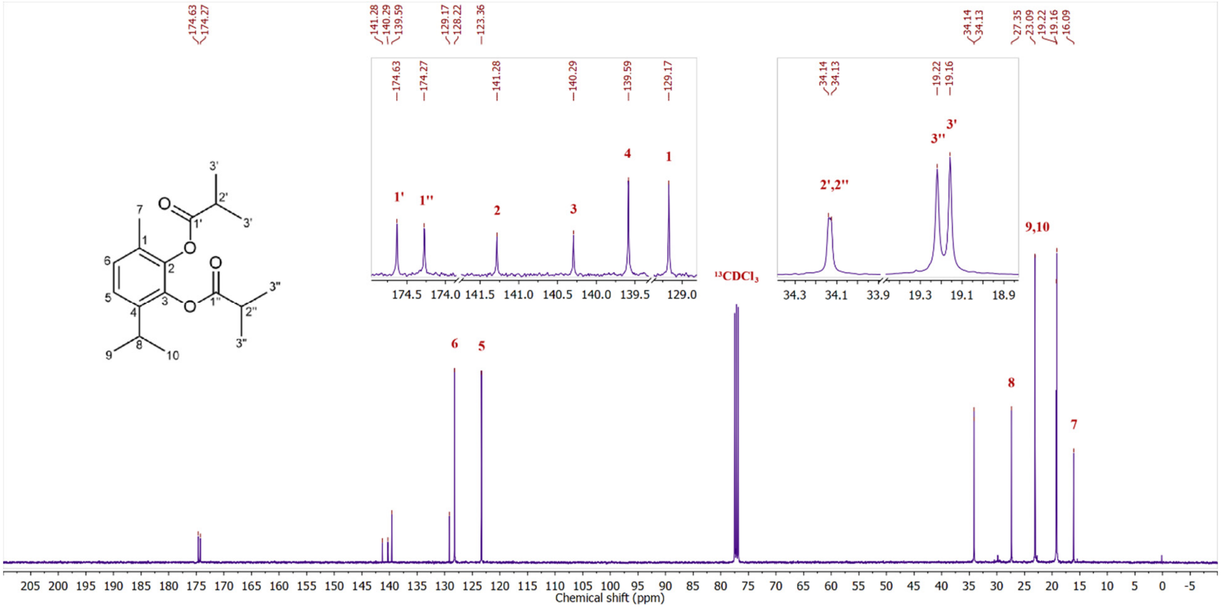

Figure S5. ¹³C NMR (100.6 MHz, $CDCl_3$) spectrum of 2-isobutyryloxythymyl isobutyrate.

Table S1. ^{1}H (400 MHz) and ^{13}C (100.6 MHz) NMR data of 2-isobutyryloxythymyl isobutyrate (chloroform-*d*); NMR parameters are derived from manual iterative full spin analysis, along with the observed gHMBC and NOESY correlations.

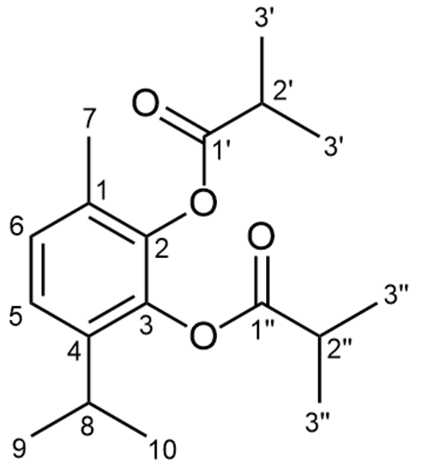

Position	δ_H (m, *J* (Hz), Integration)	δ_C	HMBC[a]	NOESY[b]
1	/	129.17	/	/
2	/	141.28	/	/
3	/	140.29	/	/
4	/	139.59	/	/
5	7.0849 (ddq, $^{3}J_{5,6}$ = 8.1, $^{4}J_{5,8}$ = -0.5, $^{5}J_{5,7}$ = 0.3, 1 H)	123.36	1,3,8	6,9,10
6	7.0720 (dqd, $^{3}J_{5,6}$ = 8.1, $^{4}J_{6,7}$ = -0.7, $^{5}J_{6,8}$ = 0.3, 1 H)	128.22	2,4,7	5,7
7	2.1243 (dd, $^{4}J_{6,7}$ = -0.7, $^{5}J_{5,7}$ = 0.3, 3 H)	16.09	1,2,6	6
8	2.9236 (septdd, $^{3}J_{8,9/10}$ = 6.9, $^{4}J_{5,8}$ = -0.5, $^{5}J_{6,8}$ = 0.3, 1 H)	27.35	3,4,5,9,10	9,10
9 and 10	1.1816 (d, $^{3}J_{8,9/10}$ = 6.9, 6 H)	23.09	4,8,9,10	5,8
1′	/	174.63	/	/
2′	2.8171 (sept, $^{3}J_{2',3'}$ = 7.0, 1 H)	34.14[c]	1′,3′	3′
3′	1.3230 (d, $^{3}J_{2',3'}$ = 7.0, 6 H)	19.16	2′,3′	2′
1′′	/	174.27	/	/
2′′	2.8030 (sept, $^{3}J_{2'',3''}$ = 7.0, 1 H)	34.13[c]	1′′,3′′	3′′
3′′	1.3144 (d, $^{3}J_{2'',3''}$ = 7.0, 6 H)	19.22	2′′,3′′	2′′

[a] gHMBC correlations observed between the hydrogen in this row and the carbon in the listed position.

[b] Cross-peaks observed in the NOESY spectrum.

[c] The signals of C-2′ and C-2′′ may be interchanged.

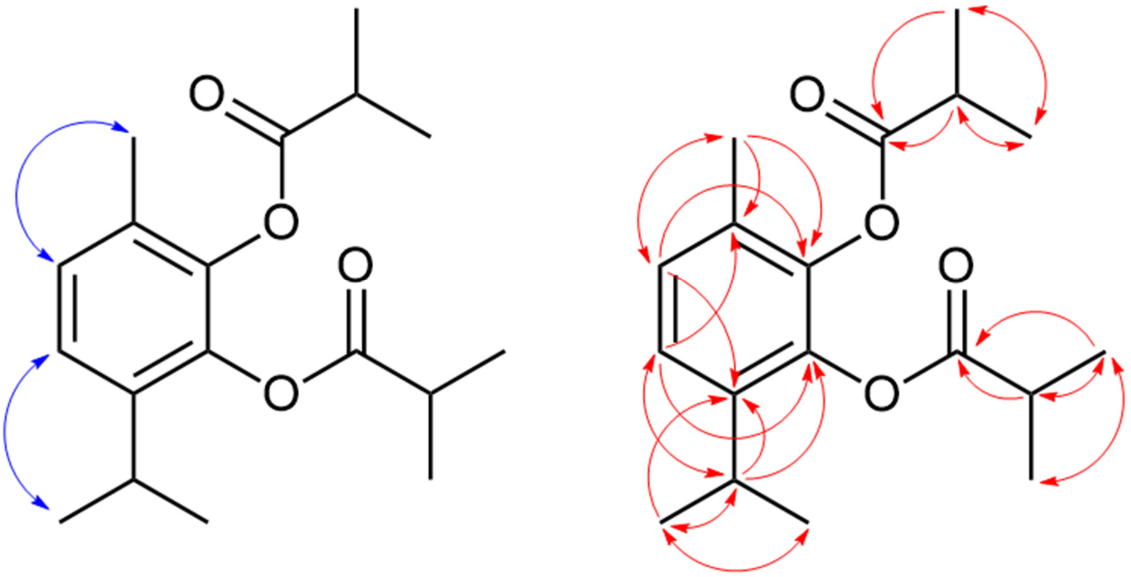

Figure S6. NOESY (blue arrows) and HMBC (red arrows) interactions of 2-isobutyryloxythymyl isobutyrate.

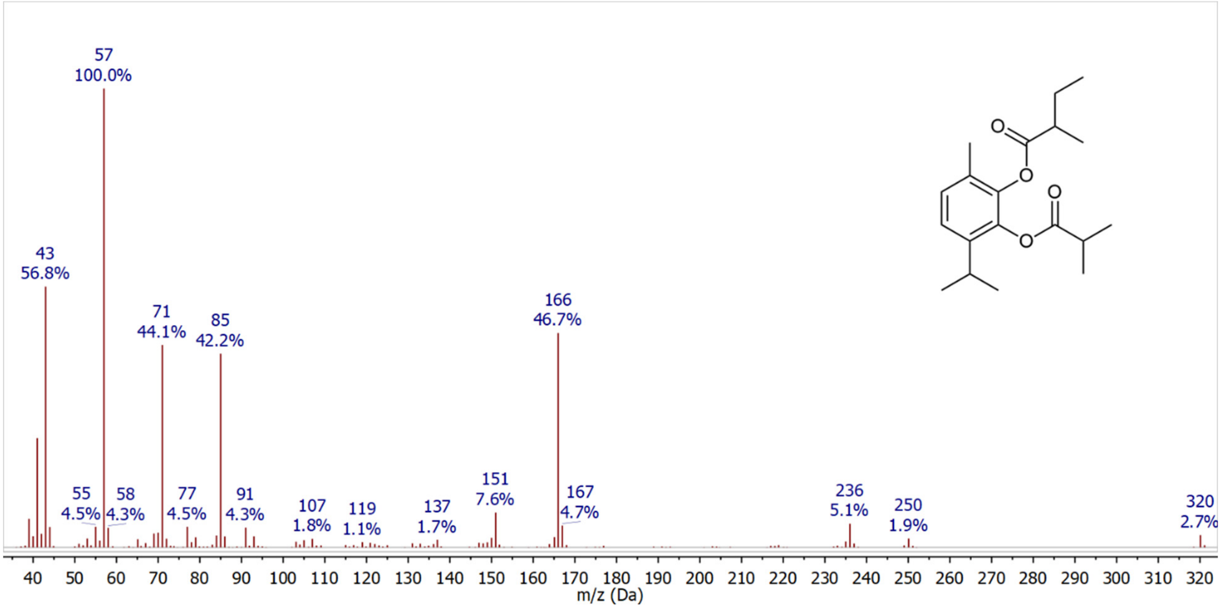

Figure S7. Mass spectrum (EI, 70 eV) of 2-(2-methylbutyryloxy)thymyl isobutyrate.

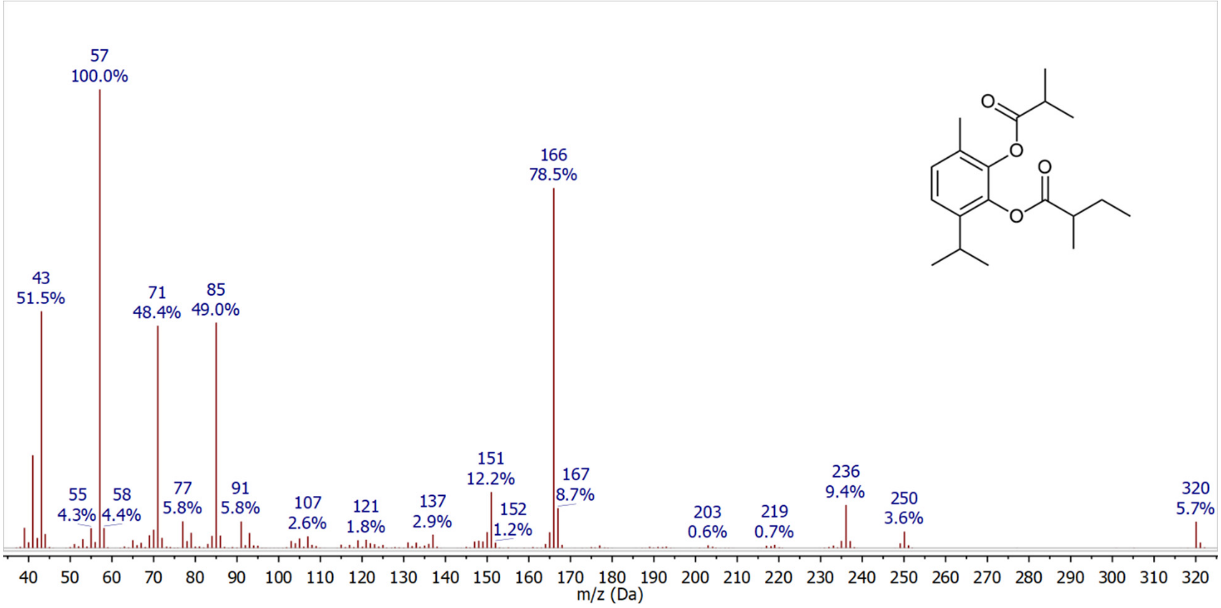

Figure S8. Mass spectrum (EI, 70 eV) of 2-isobutyryloxythymyl 2-methylbutyrate.

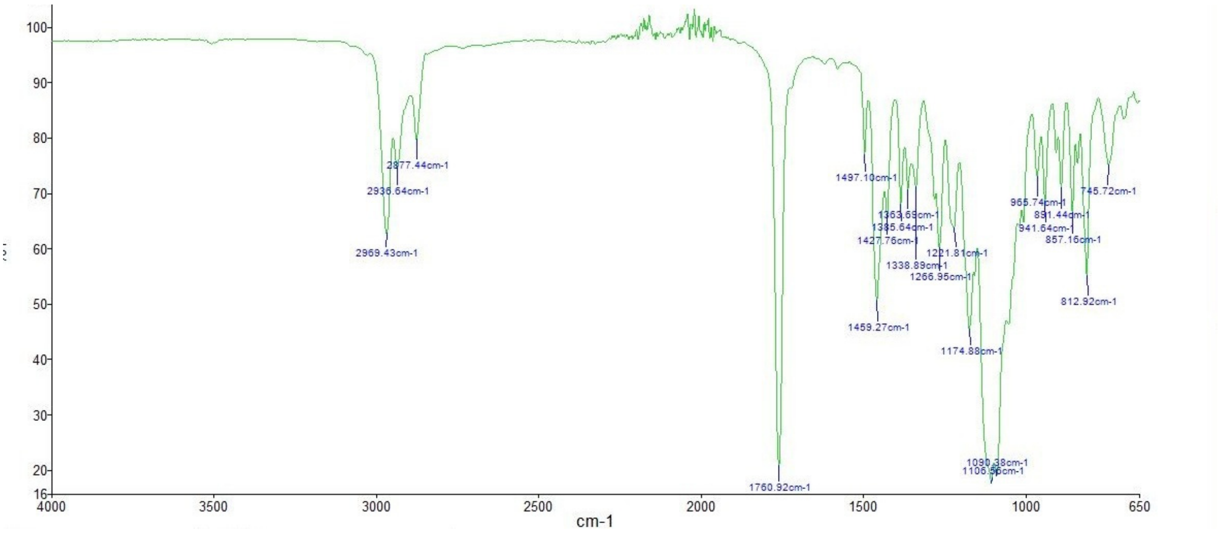

Figure S9. IR spectrum of the mixture of 2-(2-methylbutyryloxy)thymyl isobutyrate and 2-isobutyryloxythymyl 2-methylbutyrate.

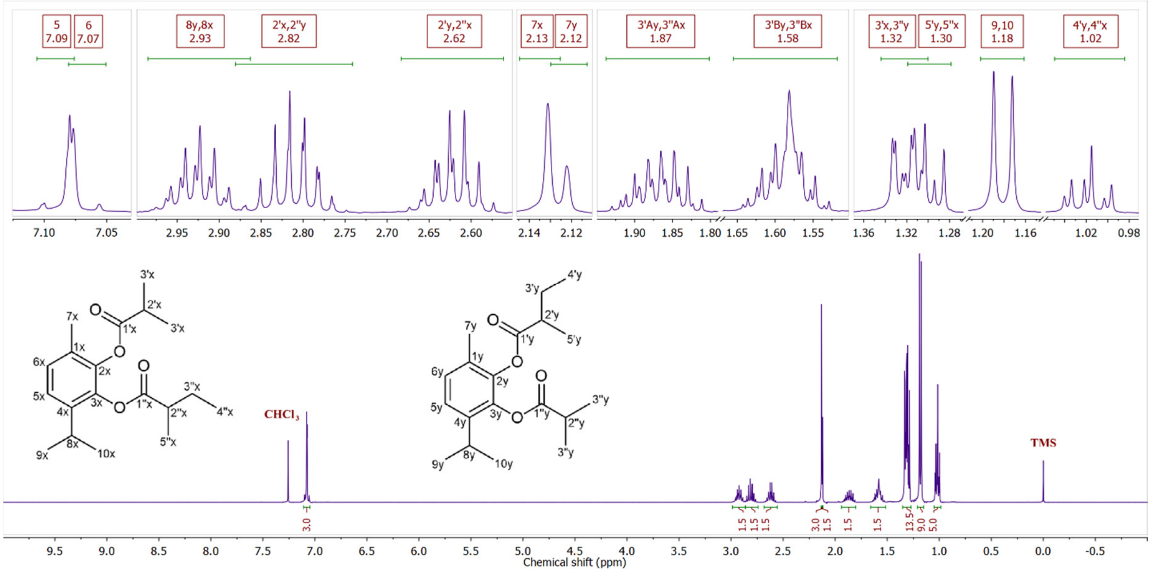

Figure S10. ^{1}H NMR (400 MHz, $CDCl_3$) spectrum of the mixture of 2-(2-methylbutyryloxy)thymyl isobutyrate (y) and 2-isobutyryloxythymyl 2-methylbutyrate (x) and the corresponding expansions with signal assignment.

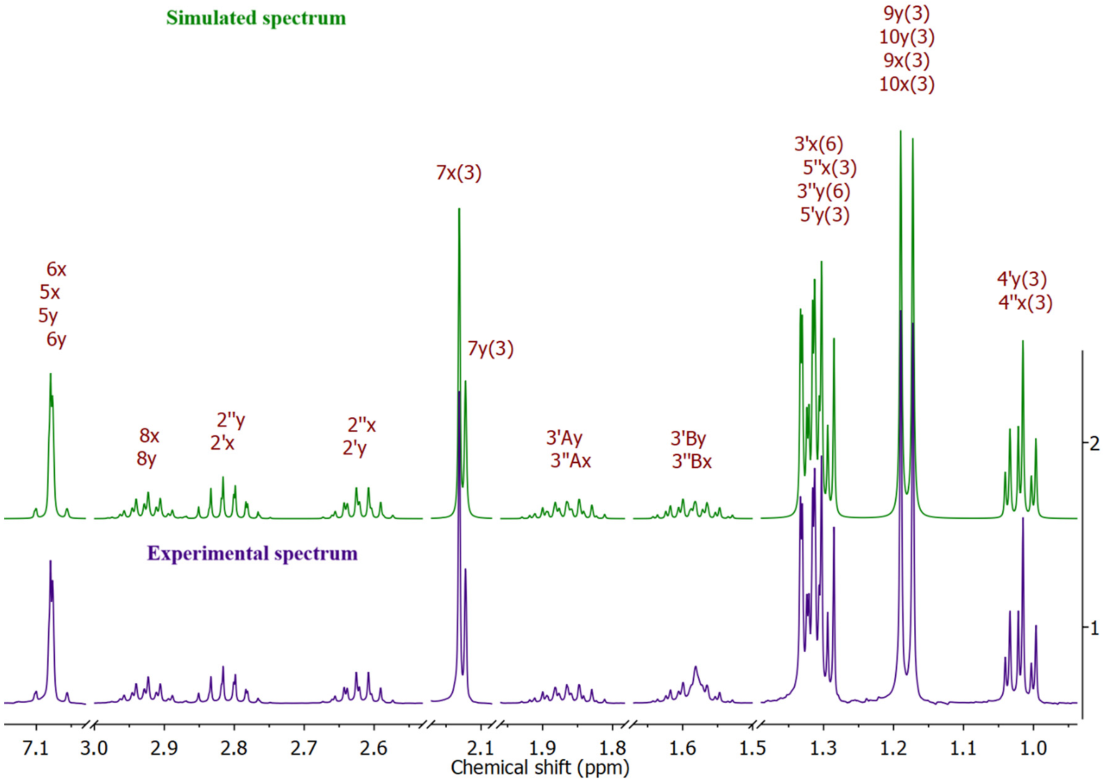

Figure S11. Simulated (manual iterative full spin, MestreNova 11.0.3) and experimental ^{1}H NMR spectrum of the mixture of 2-(2-methylbutyryloxy)thymyl isobutyrate (y) and 2-isobutyryloxythymyl 2-methylbutyrate (x).

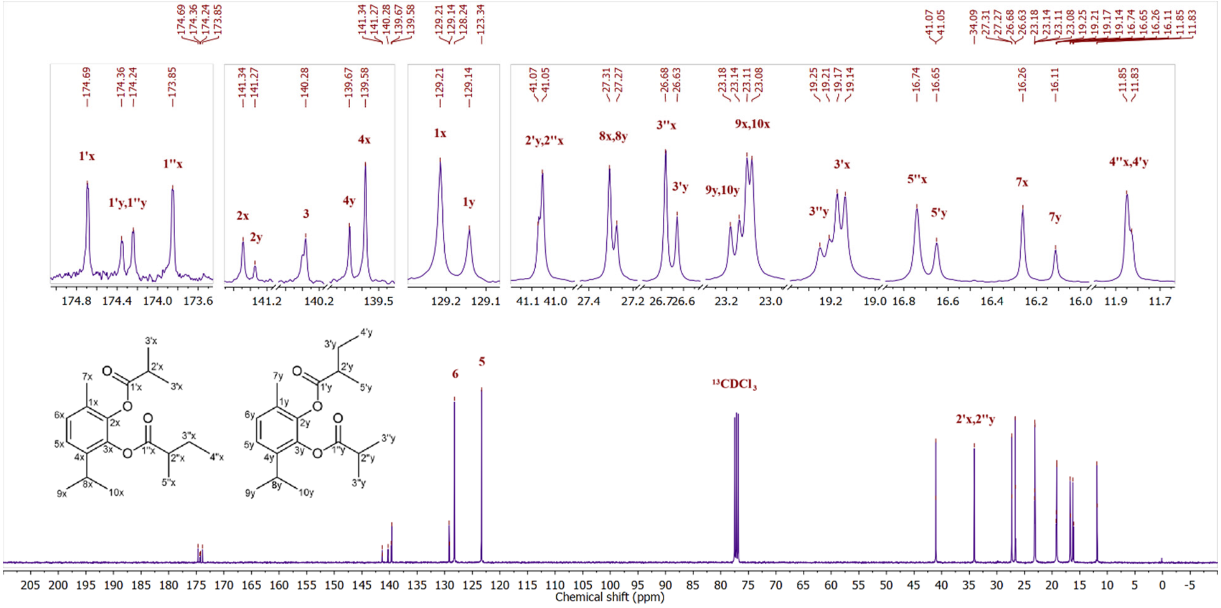

Figure S12. ^{13}C NMR (100.6 MHz, $CDCl_3$) spectrum of the mixture of 2-(2-methylbutyryloxy)thymyl isobutyrate (y) and 2-isobutyryloxythymyl 2-methylbutyrate (x).

Table S2. ^{1}H (400 MHz) and ^{13}C (100.6 MHz) NMR data of 2-(2-methylbutyryloxy)thymyl isobutyrate (chloroform-*d*); NMR parameters are derived from manual iterative full spin analysis, along with the observed gHMBC and NOESY correlations.

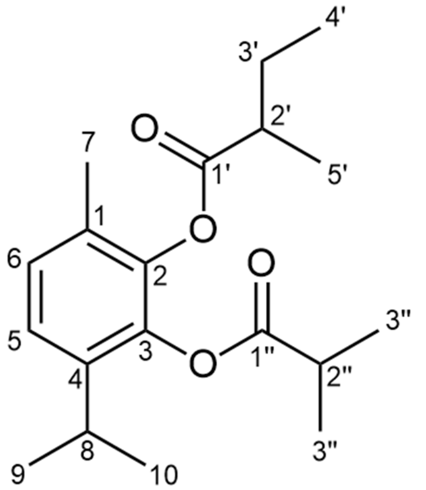

Position	δH (m, *J* (Hz), Integration)	δC	HMBC[a]	NOESY[b]
1	/	129.14	/	/
2	/	141.27	/	/
3	/	140.28	/	/
4	/	139.67	/	/
5	7.0870 (ddq, $^{3}J_{5,6}$ = 8.1, $^{4}J_{5,8}$ = -0.5, $^{5}J_{5,7}$ = 0.3, 1 H)	123.34	1,3,8	6,9,10

6	7.0720 (dqd, $^3J_{5,6}$ = 8.1, $^4J_{6,7}$ = -0.7, $^5J_{6,8}$ = 0.3, 1 H)	128.24	2,4,7	5,7
7	2.1222 (dd, $^4J_{6,7}$ = -0.7, $^5J_{5,7}$ = 0.3, 3 H)	16.11	1,2,6	6
8	2.9285 (septdd, $^3J_{8,9/10}$ = 6.9, $^4J_{5,8}$ = -0.5, $^5J_{6,8}$ = 0.3, 1 H)	27.27	3,4,5,9,10	9,10
9 and 10	1.1811 (d, $^3J_{8,9/10}$ = 6.9, 6 H)	23.14/23.18	4,8,9,10	5,8
1′	/	174.36	/	/
2′	2.6294 (dqdq, $^3J_{2',3'B}$ = 7.0, $^3J_{2',5'}$ = 7.0, $^3J_{2',3'A}$ = 6.6, $^4J_{2',4'}$ = 0.3, 1 H)	41.07	1′,3′,4′,5′	3′,4′,5′
3′A	1.8750 (dqd, $^2J_{3'A,3'B}$ = -13.7, $^3J_{3'A,4'}$ = 7.5, $^3J_{2',3'A}$ = 6.6, 1 H)	26.63	1′,2′,4′,5′	2′,4′,5′
3′B	1.5893 (dqd, $^2J_{3'A,3'B}$ = -13.7, $^3J_{3'B,4'}$ = 7.5, $^3J_{2',3'B}$ = 7.0, 1 H)	26.63	1′,2′,4′,5′	2′,4′,5′
4′	1.0220 (ddd, $^3J_{3'A,4'}$ = 7.5, $^3J_{3'B,4'}$ = 7.5, $^4J_{2',4'}$ = 0.3, 3 H)	11.83	2′,3′	2′,3′
5′	1.3029 (d, $^3J_{2',5'}$ = 7.0, 3 H)	16.65	1′,2′,3′	2′,3′
1′′	/	174.24	/	/
2′′	2.8007 (sept, $^3J_{2'',3''}$ = 7.0, 1 H)	34.09	2′′,3′′	3′′
3′′	1.3123/1.3153 (d, $^3J_{2'',3''}$ = 7.0, 6 H)	19.21/19.25	1′′,2′′,3′′	2′′

[a] gHMBC correlations observed between the hydrogen in this row and the carbon in the listed position.

[b] Cross-peaks observed in the NOESY spectrum.

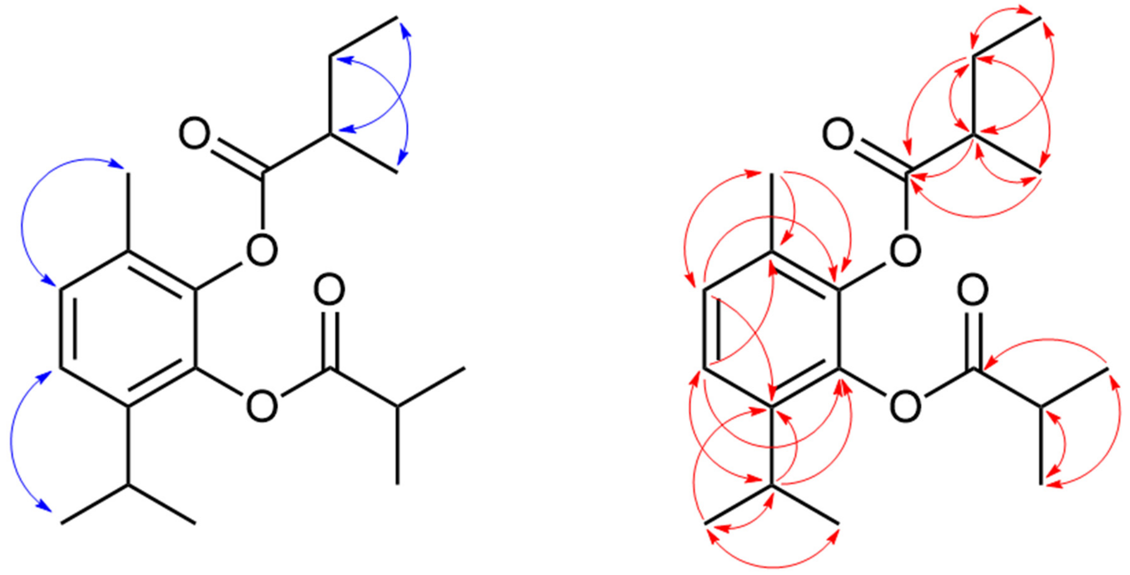

Figure S13. NOESY (blue arrows) and HMBC (red arrows) interactions of 2-(2-methylbutyryloxy)thymyl isobutyrate.

Table S3. ^{1}H (400 MHz) and ^{13}C (100.6 MHz) NMR data of 2-isobutyryloxythymyl 2-methylbutyrate (chloroform-*d*); NMR parameters are derived from manual iterative full spin analysis, along with the observed gHMBC and NOESY correlations.

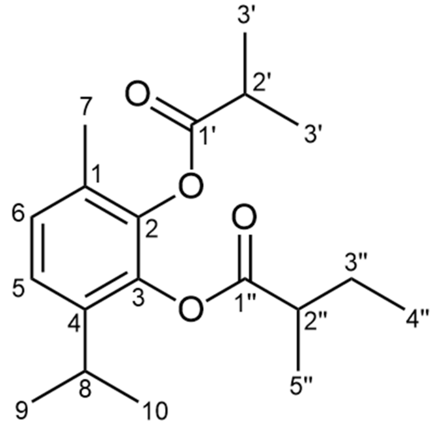

Position	δH (m, *J* (Hz), Integration)	δC	HMBC[a]	NOESY[b]
1	/	129.21	/	/
2	/	141.34	/	/
3	/	140.28	/	/
4	/	139.58	/	/
5	7.0841 (ddq, $^{3}J_{5,6}$ = 8.1, $^{4}J_{5,8}$ = -0.5, $^{5}J_{5,7}$ = 0.3, 1 H)	123.34	1,3,8	6,9,10
6	7.0720 (dqd, $^{3}J_{5,6}$ = 8.1, $^{4}J_{6,7}$ = -0.7, $^{5}J_{6,8}$ = 0.3, 1 H)	128.24	2,4,7	5,7
7	2.1312 (dd, $^{4}J_{6,7}$ = -0.7, $^{5}J_{5,7}$ = 0.3, 3 H)	16.26	1,2,6	6
8	2.9227 (septdd, $^{3}J_{8,9/10}$ = 6.9, $^{4}J_{5,8}$ = -0.5, $^{5}J_{6,8}$ = 0.3, 1 H)	27.31	3,4,5,9,10	9,10
9 and 10	1.1811 (d, $^{3}J_{8,9/10}$ = 6.9, 6 H)	23.08/23.11	4,8,9,10	5,8
1′	/	174.69	/	/
2′	2.8156 (d, $^{3}J_{2',3'}$ = 7.0, 1 H)	34.09	2′,3′	3′
3′	1.3218/1.3245 (d, $^{3}J_{2',3'}$ = 7.0, 6 H)	19.14/19.17	1′,2′,3′	2′
1′′	/	173.85	/	/
2′′	2.6164 (qddq, $^{3}J_{2'',5''}$ = 7.0, $^{3}J_{2'',3''A}$ = 6.9, $^{3}J_{2'',3''B}$ = 6.9, $^{4}J_{2'',4''}$ = 0.3, 1 H)	41.05	1′′,3′′,4′′,5′′	3′′,4′′,5′′
3′′A	1.8636 (dqd, $^{2}J_{3''A,3''B}$ = -13.7, $^{3}J_{3''A,4''}$ = 7.5, $^{3}J_{2'',3''A}$ = 6.9, 1 H)	26.68	1′′,2′′,4′′,5′′	2′′,4′′,5′′
3′′B	1.5830 (dqd, $^{2}J_{3''A,3''B}$ = -13.7, $^{3}J_{3''B,4''}$ = 7.5, $^{3}J_{2'',3''B}$ = 6.9, 1 H)	26.68	1′′,2′′,4′′,5′′	2′′,4′′,5′′
4′′	1.0153 (ddd, $^{3}J_{3''A,4''}$ = 7.5, $^{3}J_{3''B,4''}$ = 7.5, $^{4}J_{2'',4''}$ = 0.3, 3 H)	11.85	2′′,3′′	2′′,3′′
5′′	1.2940 (d, $^{3}J_{2'',5''}$ = 7.0, 3 H)	16.74	1′′,2′′,3′′	2′′,3′′

[a] gHMBC correlations observed between the hydrogen in this row and the carbon in the listed position.

[b] Cross-peaks observed in the NOESY spectrum.

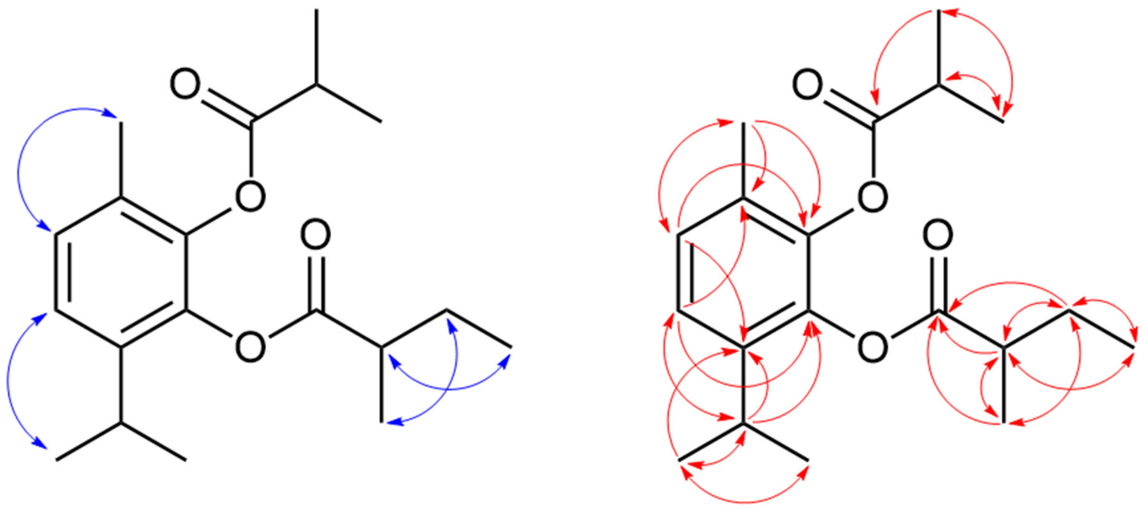

Figure S14. NOESY (blue arrows) and HMBC (red arrows) interactions of 2-isobutyryloxythymyl 2-methylbutyrate.

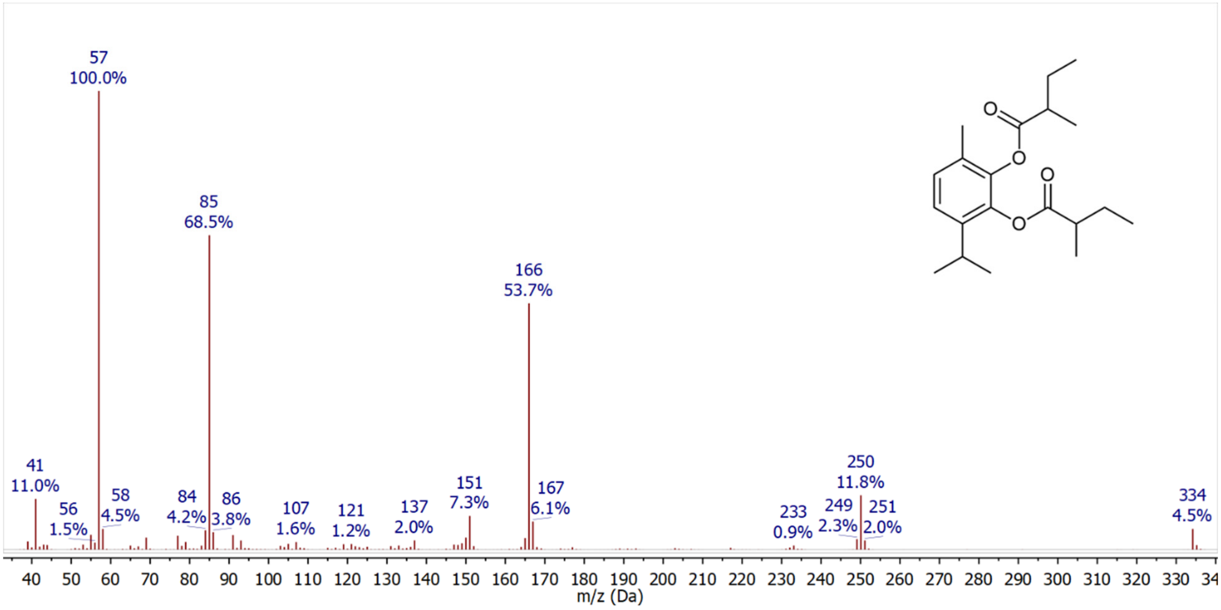

Figure S15. Mass spectrum (EI, 70 eV) of 2-(2-methylbutyryloxy)thymyl 2-methylbutyrate.

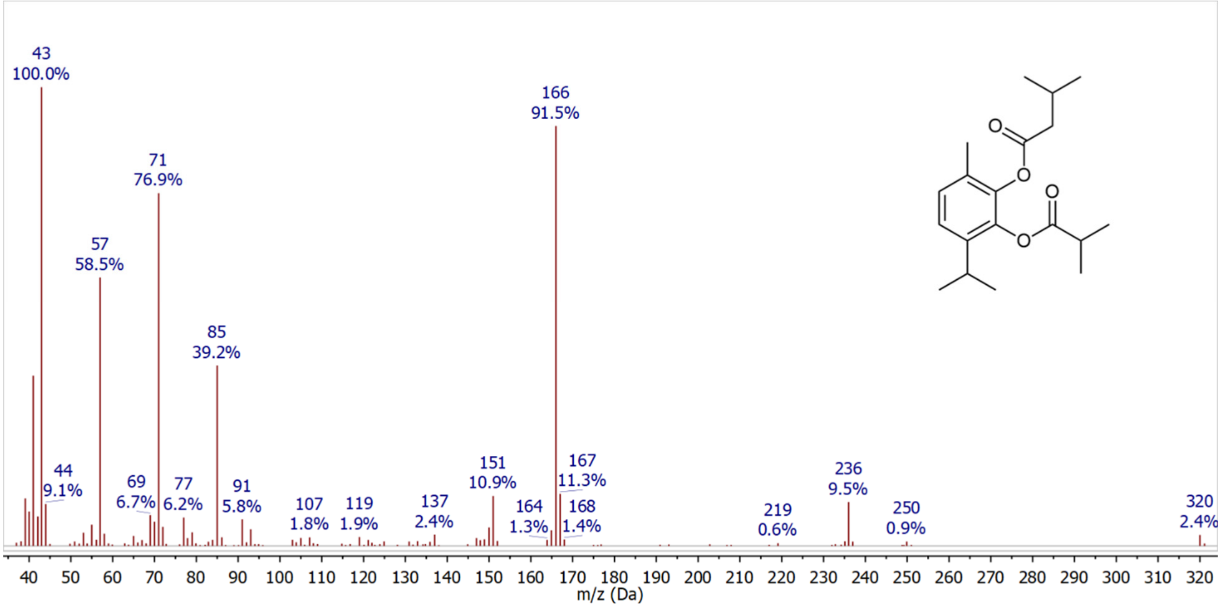

Figure S16. Mass spectrum (EI, 70 eV) of 2-isovaleroxythymyl isobutyrate.

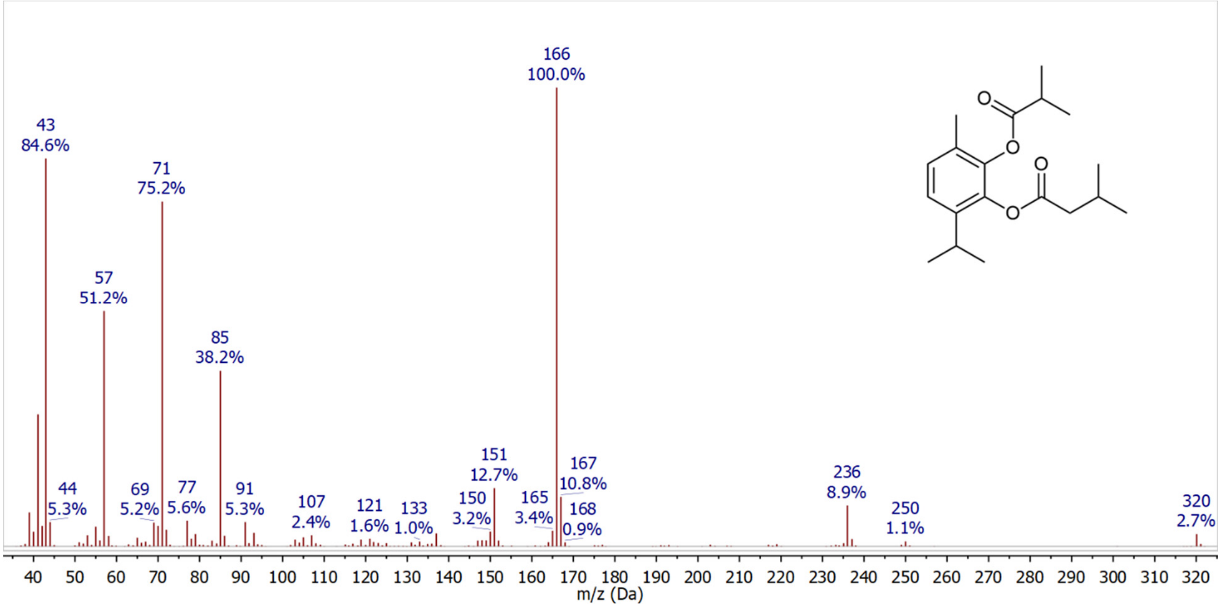

Figure S17. Mass spectrum (EI, 70 eV) of 2-isobutyryloxythymyl isovalerate.

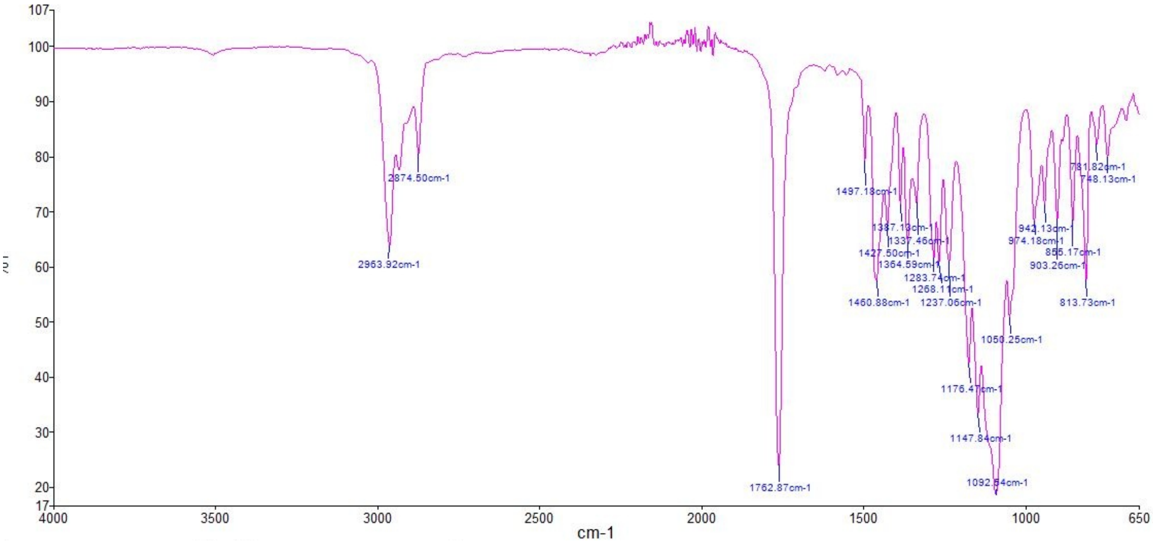

Figure S18. IR spectrum of the mixture of 2-isovaleroxythymyl isobutyrate and 2-isobutyryloxythymyl isovalerate.

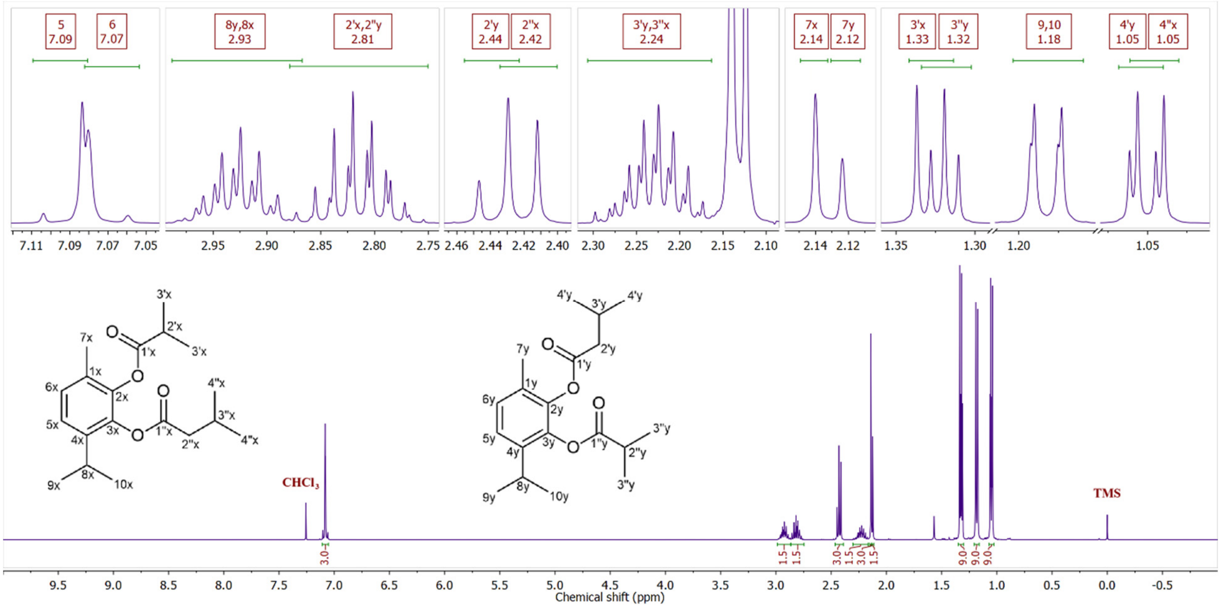

Figure S19. ^{1}H NMR (400 MHz, $CDCl_3$) spectrum of the mixture of 2-isovaleroxythymyl isobutyrate (y) and 2-isobutyryloxythymyl isovalerate (x) and the corresponding expansions with signal assignment.

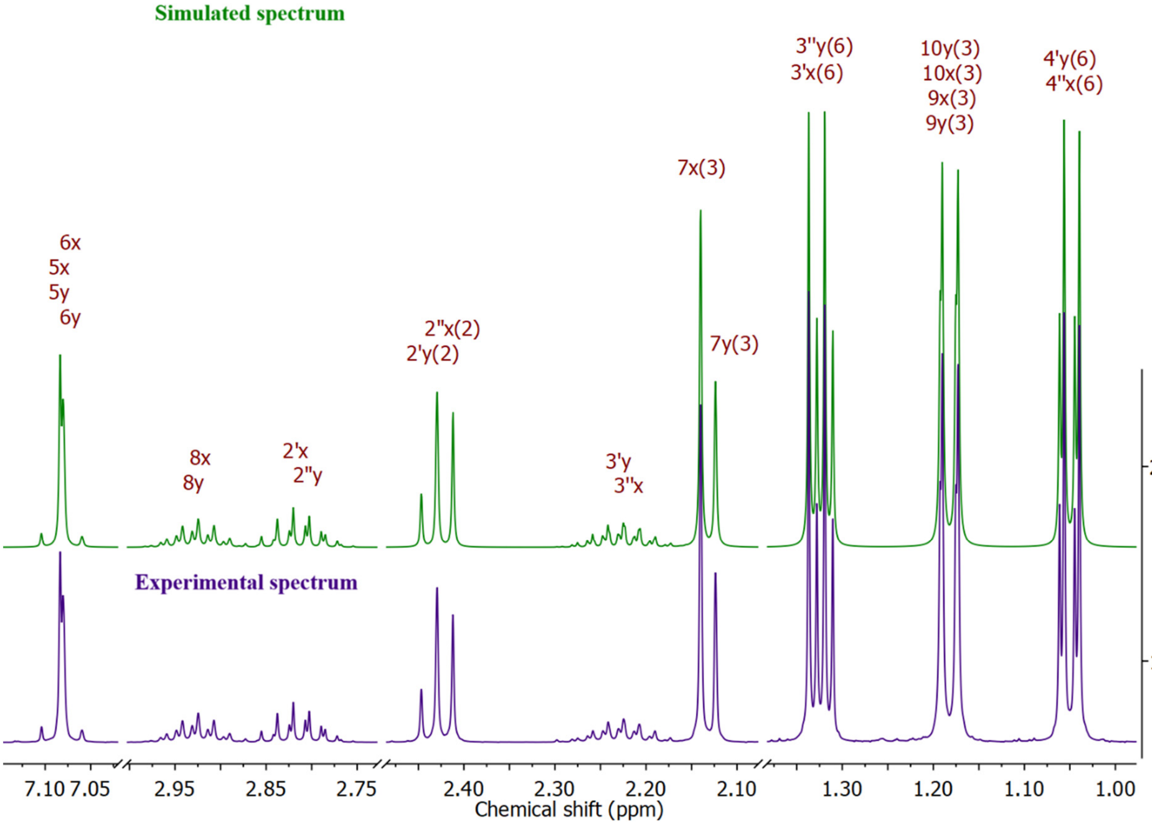

Figure S20. Simulated (manual iterative full spin, MestreNova 11.0.3) and experimental ^{1}H NMR spectrum of the mixture of 2-isovaleroxythymyl isobutyrate (y) and 2-isobutyryloxythymyl isovalerate (x).

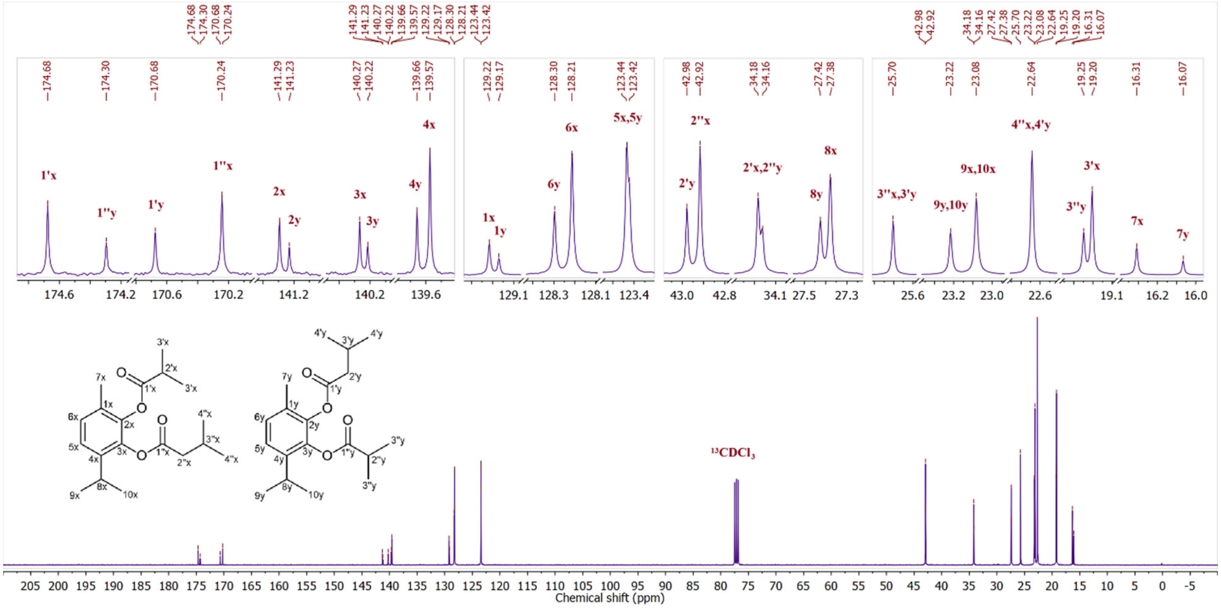

Figure S21. ^{13}C NMR (100.6 MHz, $CDCl_3$) spectrum of the mixture of 2-isovaleroxythymyl isobutyrate (y) and 2-isobutyryloxythymyl isovalerate (x).

Table S4. ^{1}H (400 MHz) and ^{13}C (100.6 MHz) NMR data of 2-isovaleroxythymyl isobutyrate (chloroform-*d*); NMR parameters are derived from manual iterative full spin analysis, along with the observed gHMBC and NOESY correlations.

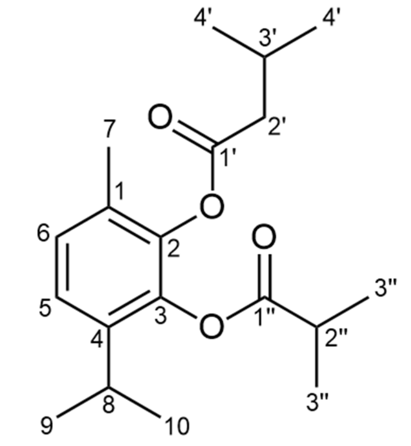

Position	δ_H (m, J (Hz), Integration)	δ_C	HMBC[a]	NOESY[b]
1	/	129.17	/	/
2	/	141.23	/	/
3	/	140.22	/	/
4	/	139.66	/	/
5	7.0880 (ddq, $^3J_{5,6}$ = 8.2, $^4J_{5,8}$ = -0.5, $^5J_{5,7}$ = 0.3, 1 H)	123.42	1,3,8	6,9,10
6	7.0756 (dqd, $^3J_{5,6}$ = 8.2, $^4J_{6,7}$ = -0.6, $^5J_{6,8}$ = 0.3, 1 H)	128.30	2,4,7	5,7
7	2.1237 (dd, $^4J_{6,7}$ = -0.6, $^5J_{5,7}$ = 0.3, 3 H)	16.07	1,2,6	6
8	2.9310 (septdd, $^3J_{8,9/10}$ = 6.9, $^4J_{5,8}$ = -0.5, $^5J_{6,8}$ = 0.3, 1 H)	27.42	3,4,5,9,10	9,10
9 and 10	1.1841 (d, $^3J_{8,9/10}$ = 6.9, 6 H)	23.22	4,8,9,10	5,8
1′	/	170.68	/	/
2′	2.4374 (d, $^3J_{2',3'}$ = 7.2, 2 H)	42.98	1′,3′,4′	3′,4′
3′	2.2305 (tsept, $^3J_{2',3'}$ = 7.2, $^3J_{3',4'}$ = 6.7, 1 H)	25.70	1′,2′,4′	2′,4′
4′	1.0531 (d, $^3J_{3',4'}$ = 6.7, 6 H)	22.64	2′,3′,4′	2′,3′
1′′	/	174.30	/	/
2′′	2.8066 (sept, $^3J_{2'',3''}$ = 7.0, 1 H)	34.16	1′′, 3′′	3′′
3′′	1.3192 (d, $^3J_{2'',3''}$ = 7.0, 6 H)	19.25	1′′,2′′,3′′	2′′

[a] gHMBC correlations observed between the hydrogen in this row and the carbon in the listed position.

[b] Cross-peaks observed in the NOESY spectrum.

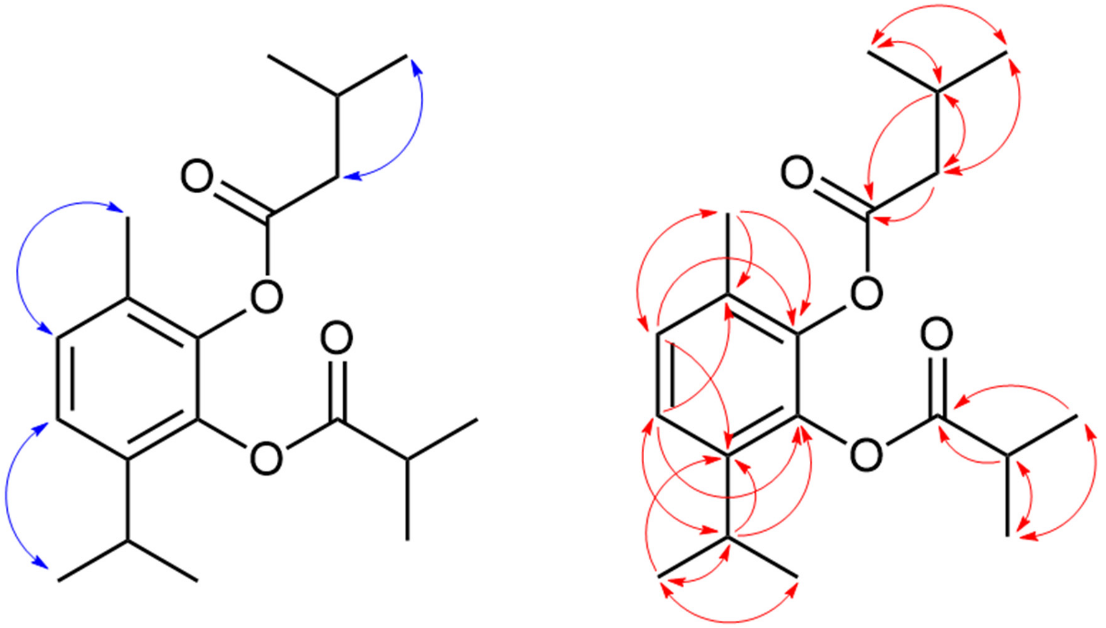

Figure S22. NOESY (blue arrows) and HMBC (red arrows) interactions of 2-isovaleroxythymyl isobutyrate.

Table S5. ^{1}H (400 MHz) and ^{13}C (100.6 MHz) NMR data of 2-isobutyryloxythymyl isovalerate (chloroform-*d*); NMR parameters are derived from manual iterative full spin analysis, along with the observed gHMBC and NOESY correlations.

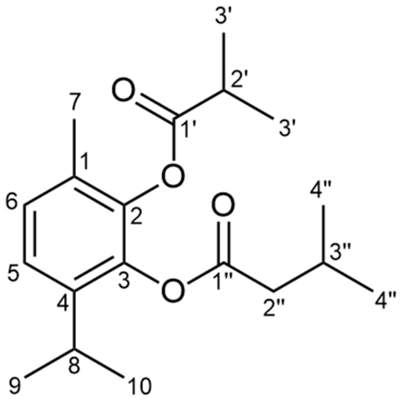

Position	δ_H (m, J (Hz), Integration)	δ_C	HMBC[a]	NOESY[b]
1	/	129.22	/	/
2	/	141.29	/	/
3	/	140.27	/	/
4	/	139.57	/	/
5	7.0880 (ddq, $^{3}J_{5,6}$ = 8.2, $^{4}J_{5,8}$ = -0.5, $^{5}J_{5,7}$ = 0.3, 1 H)	123.44	1,3,8	6,9,10
6	7.0756 (dqd, $^{3}J_{5,6}$ = 8.2, $^{4}J_{6,7}$ = -0.6, $^{5}J_{6,8}$ = 0.3, 1 H)	128.21	2,4,7	5,7
7	2.1400 (dd, $^{4}J_{6,7}$ = -0.6, $^{5}J_{5,7}$ = 0.3, 3 H)	16.31	1,2,6	6

8	2.9242 (septdd, $^3J_{8,9/10}$ = 6.9, $^4J_{5,8}$ = -0.5, $^5J_{6,8}$ = 0.3, 1 H)	27.38	3,4,5,9,10	9,10
9 and 10	1.1815 (d, $^3J_{8,9/10}$ = 6.9, 6 H)	23.08	4,8,9,10	5,8
1′	/	174.68	/	/
2′	2.8198 (sept, $^3J_{2',3'}$ = 7.0, 1 H)	34.18	1′,3′	3′
3′	1.3281 (d, $^3J_{2',3'}$ = 7.0, 6 H)	19.20	2′,3′	12
1′′	/	170.24	/	/
2′′	2.4205 (d, $^3J_{2'',3''}$ = 7.2, 2 H)	42.92	1′′,3′′,4′′	3′′,4′′
3′′	2.2246 (tsept, $^3J_{2'',3''}$ = 7.2, $^3J_{3'',4''}$ = 6.7, 1 H)	25.70	1′′,2′′,4′′	2′′,4′′
4′′	1.0480 (d, $^3J_{3'',4''}$ = 6.7, 6 H)	22.64	2′′,3′′,4′′	2′′,3′′

[a] gHMBC correlations observed between the hydrogen in this row and the carbon in the listed position.

[b] Cross-peaks observed in the NOESY spectrum.

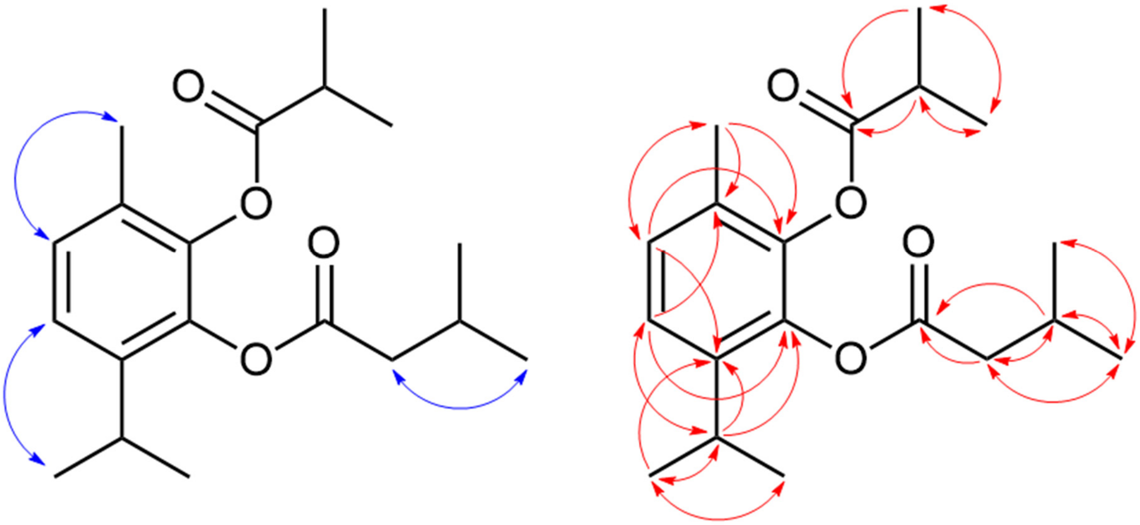

Figure S23. NOESY (blue arrows) and HMBC (red arrows) interactions of 2-isobutyryloxythymyl isovalerate.

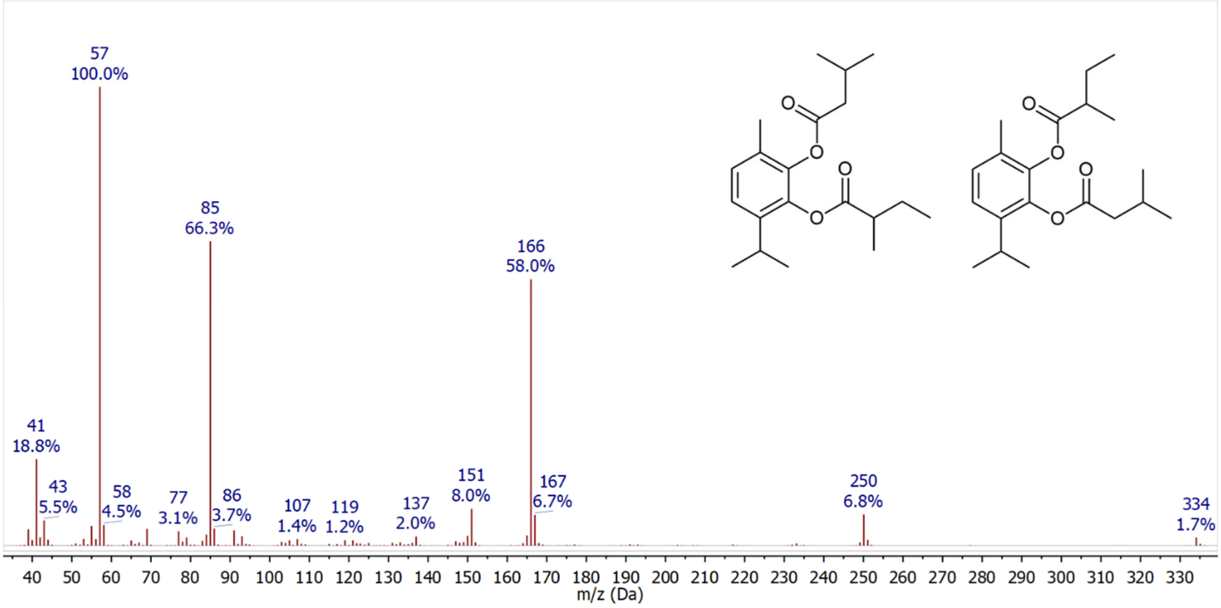

Figure S24. Mass spectrum (EI, 70 eV) of the mixture of 2-isovaleroxythymyl 2-methylbutyrate and 2-(2-methylbutyryloxy)thymyl isovalerate.

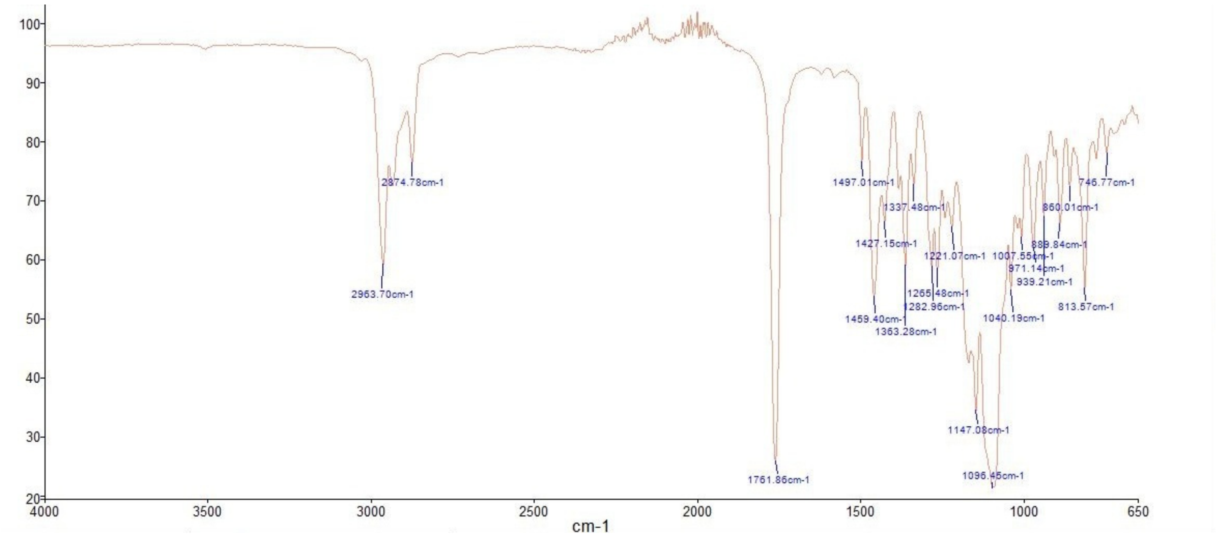

Figure S25. IR spectrum of the mixture of 2-isovaleroxythymyl 2-methylbutyrate and 2-(2-methylbutyryloxy)thymyl isovalerate.

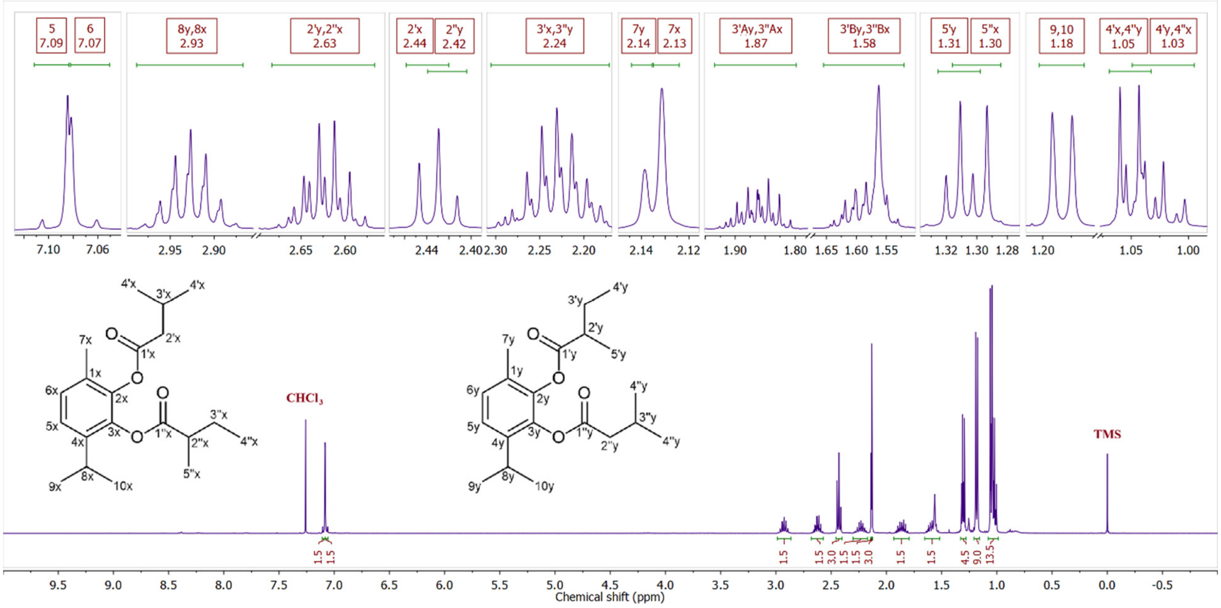

Figure S26. ^{1}H NMR (400 MHz, $CDCl_3$) spectrum of the mixture of 2-isovaleroxythymyl 2-methylbutyrate (x) and 2-(2-methylbutyryloxy)thymyl isovalerate (y) and the corresponding expansions with signal assignment.

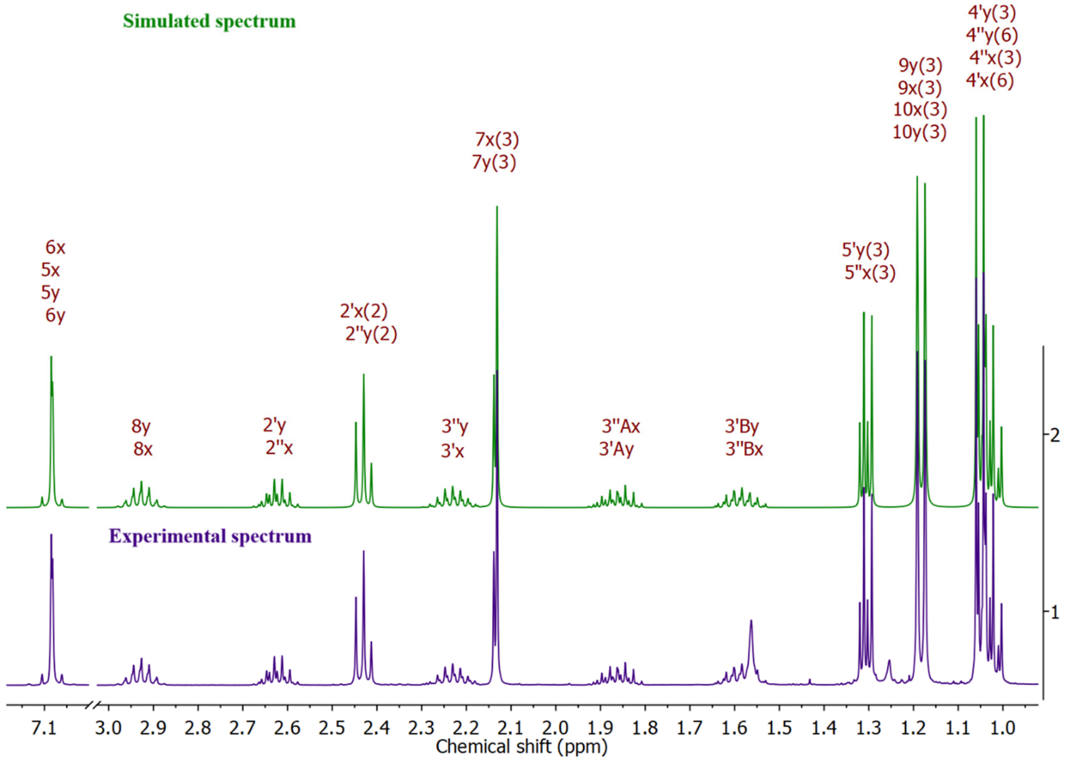

Figure S27. Simulated (manual iterative full spin, MestreNova 11.0.3) and experimental ^{1}H NMR spectrum of the mixture of 2-isovaleroxythymyl 2-methylbutyrate (x) and 2-(2-methylbutyryloxy)thymyl isovalerate (y).

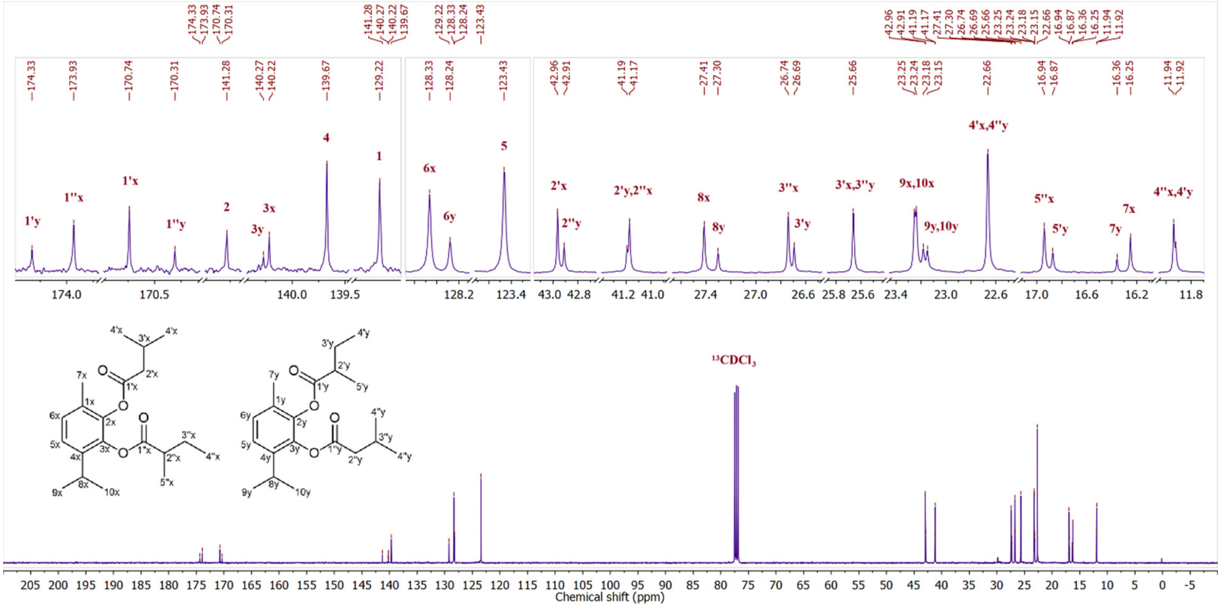

Figure S28. ^{13}C NMR (100.6 MHz, $CDCl_3$) spectrum of the mixture of 2-isovaleroxythymyl 2-methylbutyrate (x) and 2-(2-methylbutyryloxy)thymyl isovalerate (y).

Table S6. ^{1}H (400 MHz) and ^{13}C (100.6 MHz) NMR data of 2-isovaleroxythymyl 2-methylbutyrate (chloroform-*d*); NMR parameters are derived from manual iterative full spin analysis, along with the observed gHMBC and NOESY correlations.

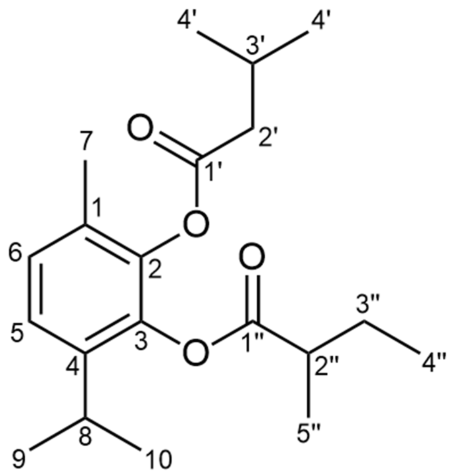

Position	δ_H (m, *J* (Hz), Integration)	δ_C	HMBC[a]	NOESY[b]
1	/	129.22	/	/
2	/	141.28	/	/
3	/	140.22	/	/
4	/	139.67	/	/
5	7.0891 (ddq, $^3J_{5,6}$ = 8.1, $^4J_{5,8}$ = -0.5, $^5J_{5,7}$ = 0.3, 1 H)	123.43	1,3,8	6,9,10
6	7.0770 (dqd, $^3J_{5,6}$ = 8.1, $^4J_{6,7}$ = -0.7, $^5J_{6,8}$ = 0.3, 1 H)	128.33	2,4,7	5,7

7	2.1315 (dd, $^4J_{6,7}$ = -0.7, $^5J_{5,7}$ = 0.3, 3 H)	16.25	1,2,6	6
8	2.9265 (septdd, $^3J_{8,9/10}$ = 6.9, $^4J_{5,8}$ = -0.5, $^5J_{6,8}$ = 0.3, 1 H)	27.41	3,4,5,9,10	9,10
9 and 10	1.1832 (d, $^3J_{8,9/10}$ = 6.9, 6 H)	23.24/23.25	4,8,9,10	5,8
1′	/	170.74	/	/
2′	2.4377 (d, $^3J_{2',3'}$ = 7.1, 2 H)	42.96	1′,3′,4′	3′,4′
3′	2.2309 (tsept, $^3J_{2',3'}$ = 7.1, $^3J_{3',4'}$ = 6.7, 1 H)	25.66	1′,2′,4′	2′,4′
4′	1.0515 (d, $^3J_{3',4'}$ = 6.7, 6 H)	22.66	2′,3′,4′	2′,3′
1′′	/	173.93	/	/
2′′	2.6205 (dqdq, $^3J_{2'',3''A}$ = 7.2, $^3J_{2'',5''}$ = 7.0, $^3J_{2'',3''B}$ = 6.6, $^4J_{2'',4''}$ = 0.3, 1 H)	41.17	1′′,3′′,4′′,5′′	3′′A,3′′B,4′′,5′′
3′′A	1.8604 (dqd, $^2J_{3''A,3''B}$ = -13.7, $^3J_{3''A,4''}$ = 7.4, $^3J_{2'',3''A}$ = 7.2, 1 H)	26.74	1′′,2′′,4′′,5′′	2′′,4′′,5′′
3′′B	1.5845 (dqd, $^2J_{3''A,3''B}$ = -13.7, $^3J_{3''B,4''}$ = 7.4, $^3J_{2'',3''B}$ = 6.6, 1 H)	26.74	1′′,2′′,4′′,5′′	2′′,4′′,5′′
4′′	1.0219 (ddd, $^3J_{3''A,4''}$ = 7.4, $^3J_{3''B,4''}$ = 7.4, $^4J_{2'',4''}$ = 0.3, 3 H)	11.94	2′′,3′′	2′′,3′′A,3′′B
5′′	1.3021 (d, $^3J_{2'',5''}$ = 7.0, 3 H)	16.94	1′′,2′′,3′′	2′′,3′′A,3′′B

[a] gHMBC correlations observed between the hydrogen in this row and the carbon in the listed position.

[b] Cross-peaks observed in the NOESY spectrum.

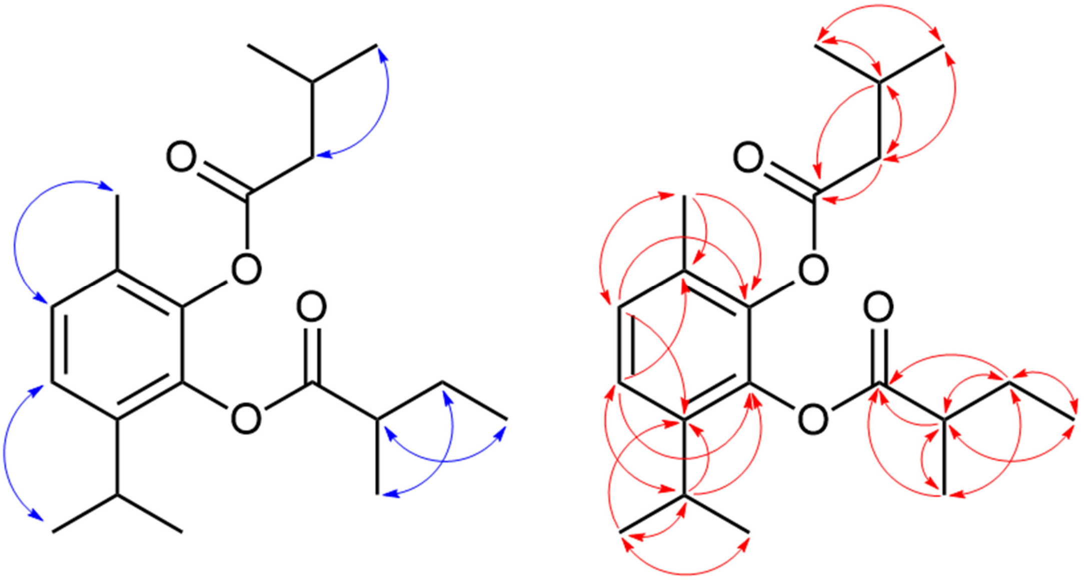

Figure S29. NOESY (blue arrows) and HMBC (red arrows) interactions of 2-isovaleroxythymyl 2-methylbutyrate.

Table S7. ^{1}H (400 MHz) and ^{13}C (100.6 MHz) NMR data of 2-(2-methylbutyryloxy)thymyl isovalerate (chloroform-*d*); NMR parameters are derived from manual iterative full spin analysis, along with the observed gHMBC and NOESY correlations.

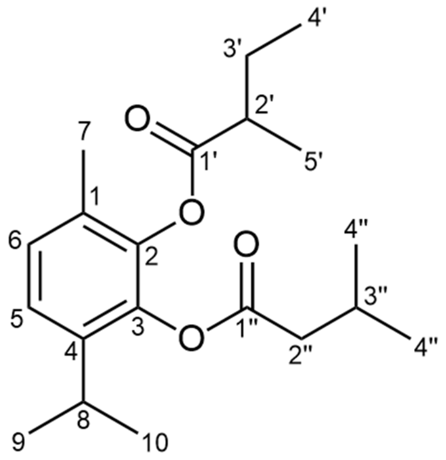

Position	δH (m, *J* (Hz), Integration)	δC	HMBC[a]	NOESY[b]
1	/	129.22	/	/
2	/	141.28	/	/
3	/	140.27	/	/
4	/	139.67	/	/
5	7.0891 (ddq, $^{3}J_{5,6}$ = 8.1, $^{4}J_{5,8}$ = -0.5, $^{5}J_{5,7}$ = 0.3, 1 H)	123.43	1,3,8	6,9,10
6	7.0770 (dqd, $^{3}J_{5,6}$ = 8.1, $^{4}J_{6,7}$ = -0.7, $^{5}J_{6,8}$ = 0.3, 1 H)	128.24	2,4,7	5,7
7	2.1386 (dd, $^{4}J_{6,7}$ = -0.7, $^{5}J_{5,7}$ = 0.3, 3 H)	16.36	1,2,6	6
8	2.9303 (septdd, $^{3}J_{8,9/10}$ = 6.9, $^{4}J_{5,8}$ = -0.5, $^{5}J_{6,8}$ = 0.3, 1 H)	27.30	3,4,5,9,10	9,10
9 and 10	1.1814 (d, $^{3}J_{8,9/10}$ = 6.9, 6 H)	23.15/23.18	4,8,9,10	5,8
1′	/	174.33	/	/
2′	2.6316 (dqdq, $^{3}J_{2',3'A}$ = 7.2, $^{3}J_{2',5'}$ = 7.0, $^{3}J_{2',3'B}$ = 6.6, $^{4}J_{2',4'}$ = 0.3, 1 H)	41.19	1′,3′,4′,5′	3′A,3′B,4′,5′
3′A	1.8709 (dqd, $^{2}J_{3'A,3'B}$ = -13.7, $^{3}J_{3'A,4'}$ = 7.4, $^{3}J_{2',3'A}$ = 7.2, 1 H)	26.69	1′,2′,4′,5′	2′,4′,5′
3′B	1.5904 (dqd, $^{2}J_{3'A,3'B}$ = -13.7, $^{3}J_{3'B,4'}$ = 7.4, $^{3}J_{2',3'B}$ = 6.6, 1 H)	26.69	1′,2′,4′,5′	2′,4′,5′
4′	1.0291 (ddd, $^{3}J_{3'A,4'}$ = 7.4, $^{3}J_{3'B,4'}$ = 7.4, $^{4}J_{2',4'}$ = 0.3, 3 H)	11.92	2′,3′	2′,3′A,3′B
5′	1.3114 (d, $^{3}J_{2',5'}$ = 7.0, 3 H)	16.87	1′,2′,3′	2′,3′A,3′B
1′′	/	170.31	/	/
2′′	2.4210 (d, $^{3}J_{2'',3''}$ = 7.1, 2 H)	42.91	1′′,3′′,4′′	3′′,4′′
3′′	2.2256 (tsept, $^{3}J_{2'',3''}$ = 7.1, $^{3}J_{3'',4''}$ = 6.7, 1 H)	25.66	1′′,2′′,4′′	2′′,4′′
4′′	1.0462 (d, $^{3}J_{3'',4''}$ = 6.7, 6 H)	22.66	2′′,3′′,4′′	2′′,3′′

[a] gHMBC correlations observed between the hydrogen in this row and the carbon in the listed position.

[b] Cross-peaks observed in the NOESY spectrum.

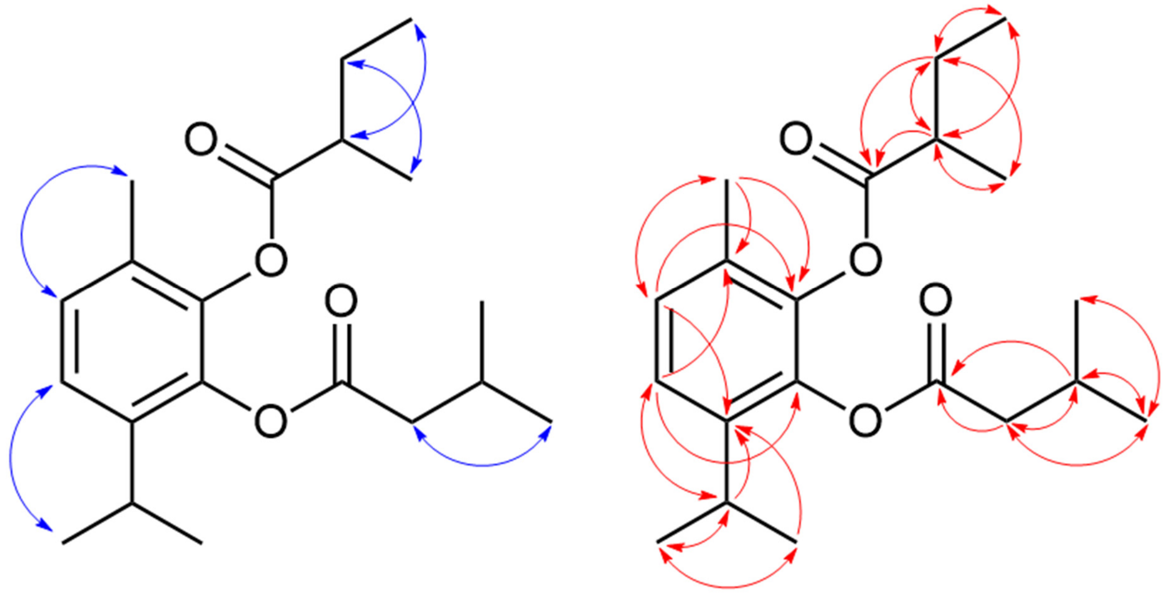

Figure S30. NOESY (blue arrows) and HMBC (red arrows) interactions of 2-(2-methylbutyryloxy)thymyl isovalerate.

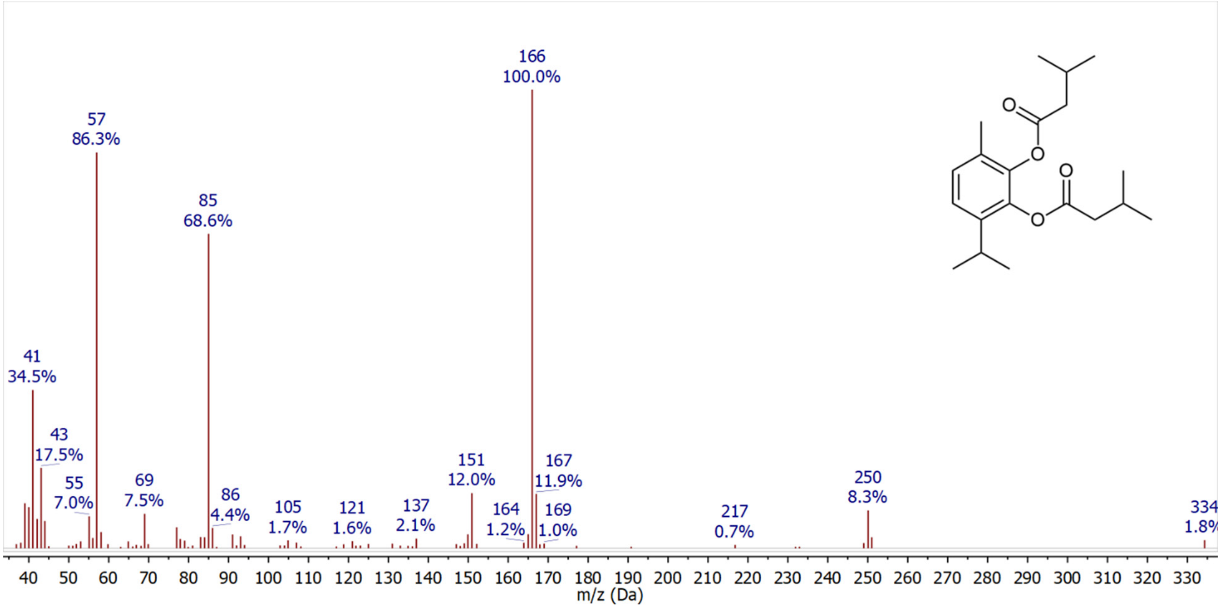

Figure S31. Mass spectrum (EI, 70 eV) of 2-isovaleroxythymyl isovalerate.

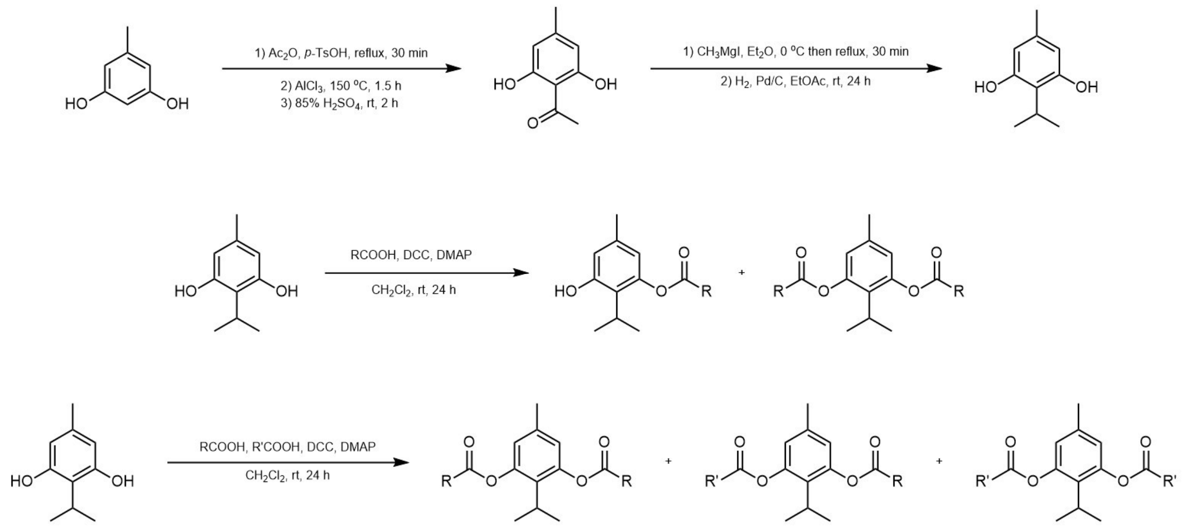

Scheme S2. Synthetic pathway to 5-hydroxythymol-derived monoesters and diesters (R,R′ – isopropyl, *sec*-butyl, or isobutyl).

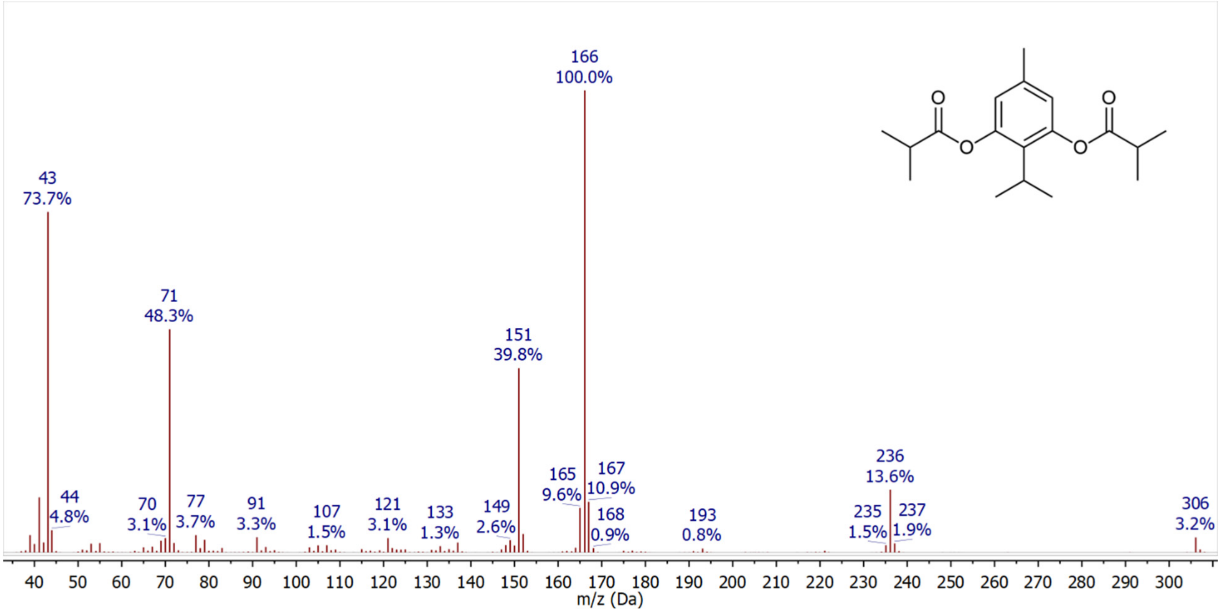

Figure S32. Mass spectrum (EI, 70 eV) of 5-isobutyryloxythymyl isobutyrate.

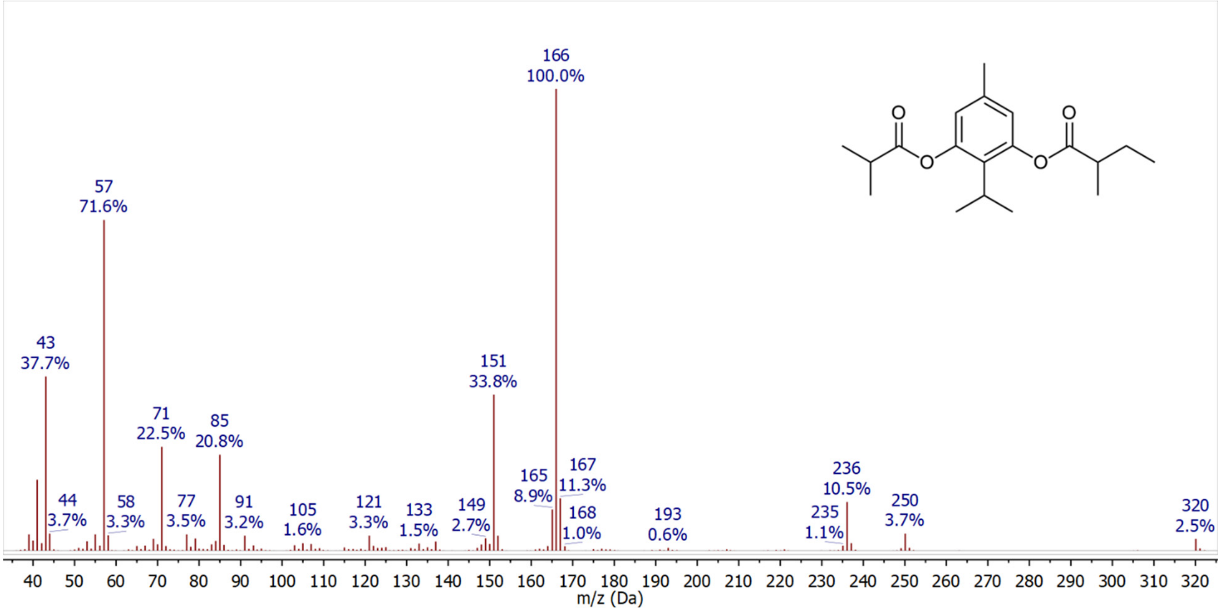

Figure S33. Mass spectrum (EI, 70 eV) of 5-isobutyryloxythymyl 2-methylbutyrate.

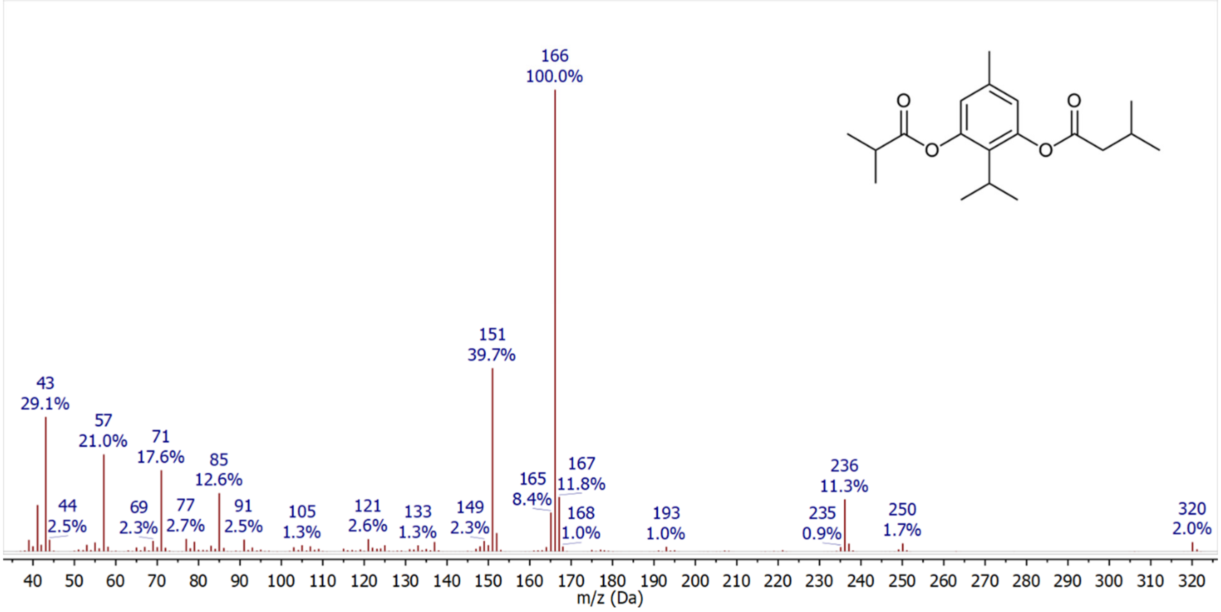

Figure S34. Mass spectrum (EI, 70 eV) of 5-isobutyryloxythymyl isovalerate.

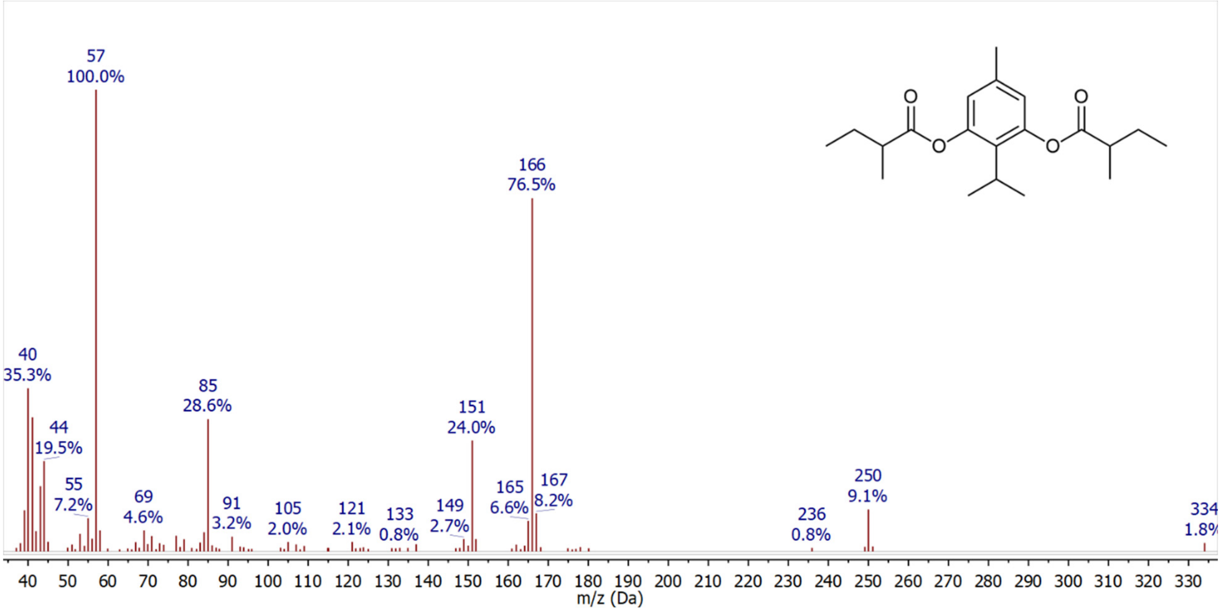

Figure S35. Mass spectrum (EI, 70 eV) of 5-(2-methylbutyryloxy)thymyl 2-methylbutyrate.

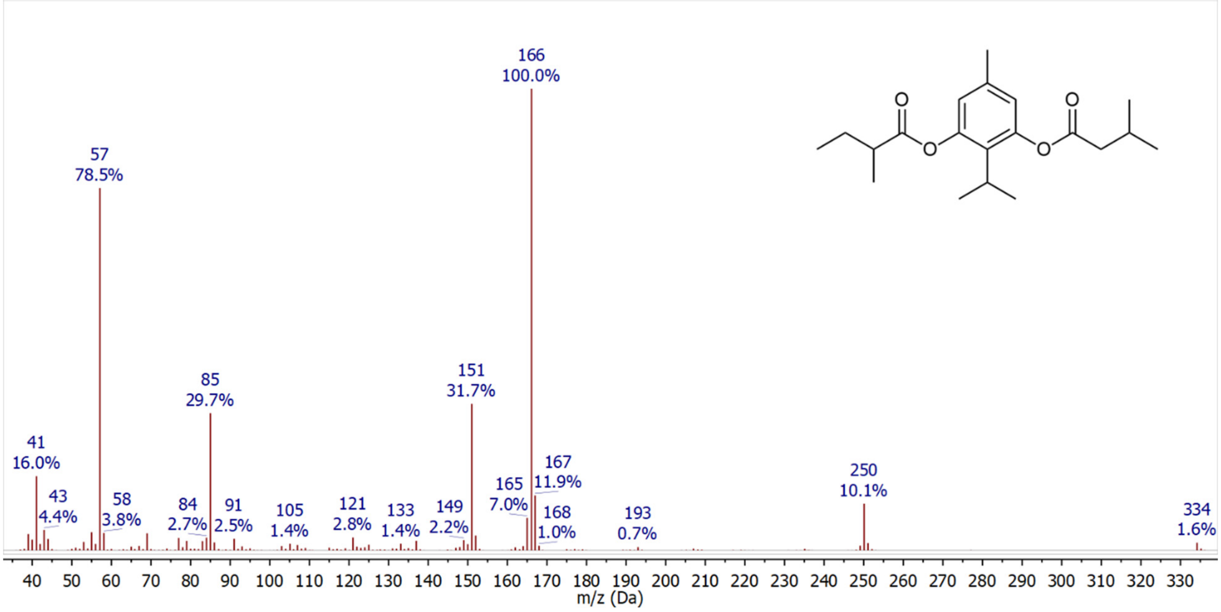

Figure S36. Mass spectrum (EI, 70 eV) of 5-(2-methylbutyryloxy)thymyl isovalerate.

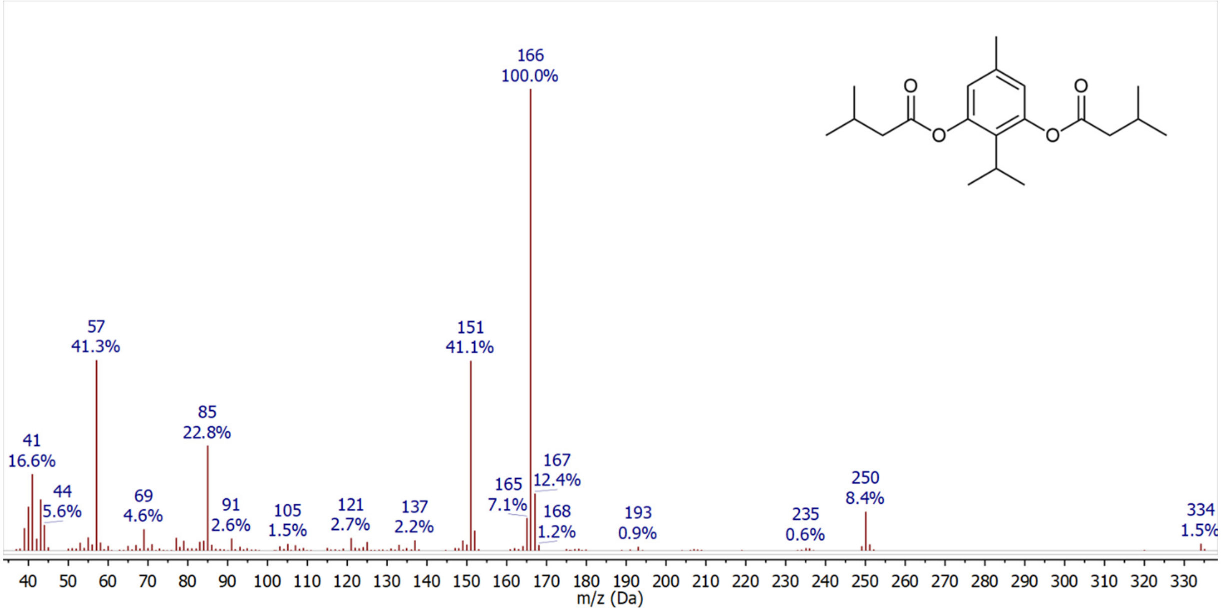

Figure S37. Mass spectrum (EI, 70 eV) of 5-isovaleroxythymyl isovalerate.

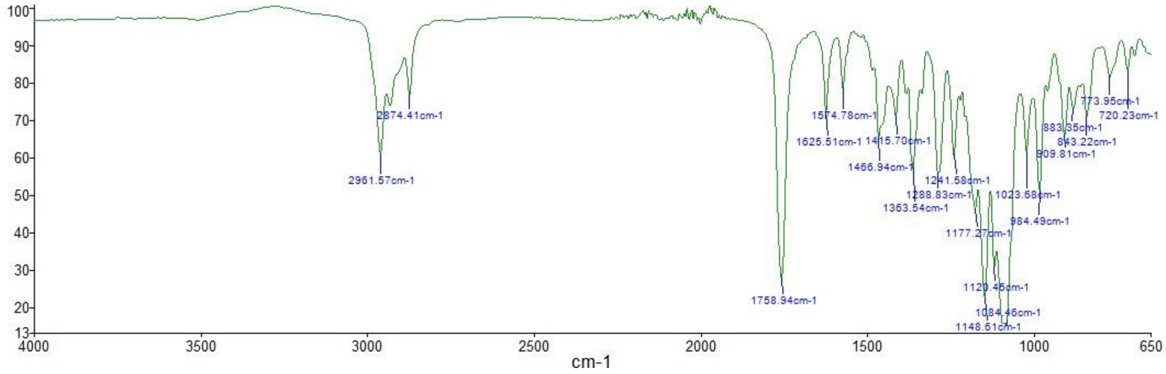

Figure S38. IR spectrum of 5-isovaleroxythymyl isovalerate.

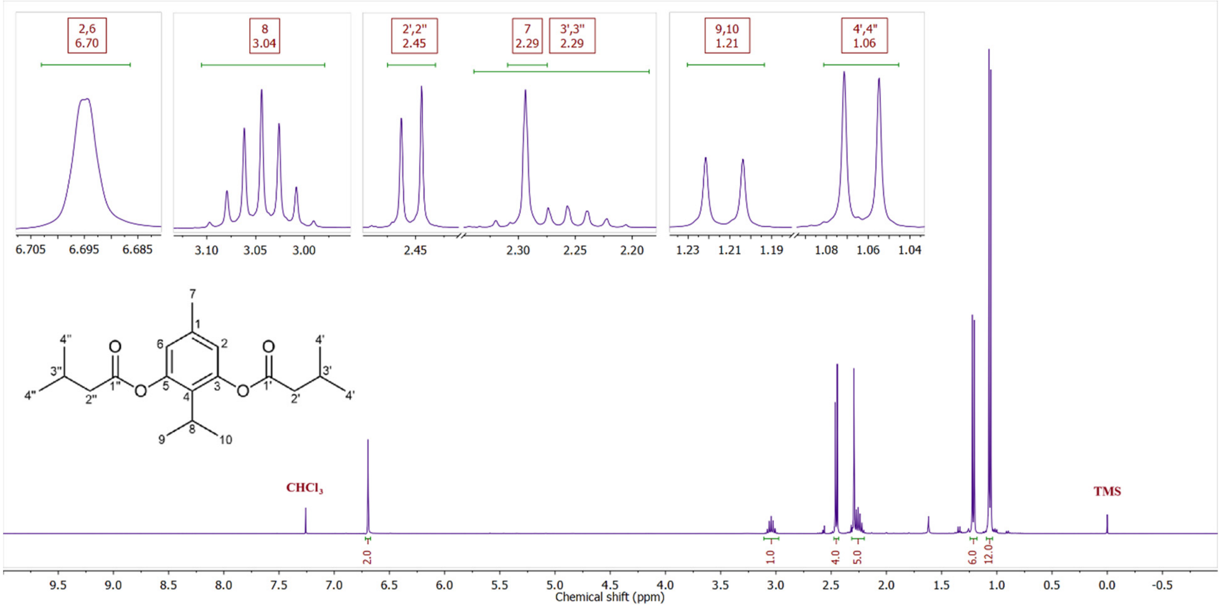

Figure S39. ^{1}H NMR (400 MHz, $CDCl_3$) spectrum of 5-isovaleroxythymyl isovalerate and the corresponding expansions with signal assignment.

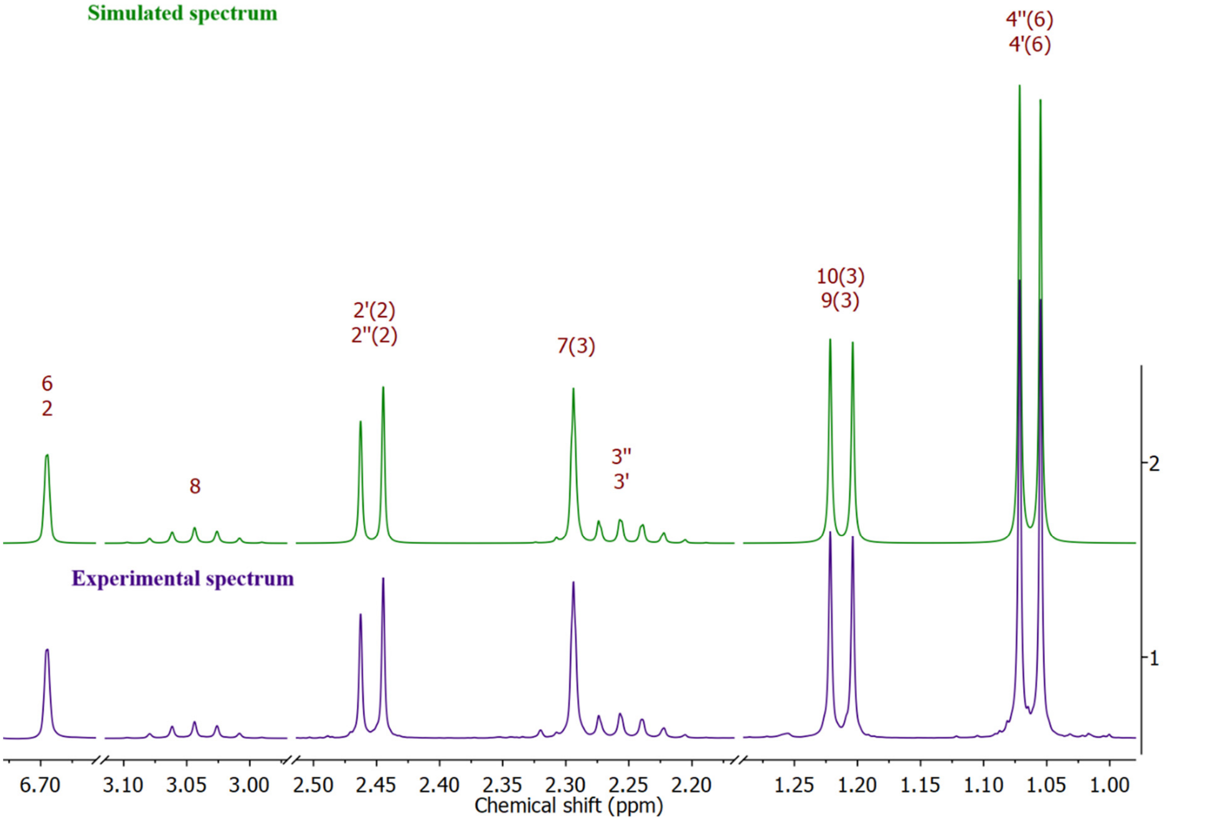

Figure S40. Simulated (manual iterative full spin, MestreNova 11.0.3) and experimental ^{1}H NMR spectrum of 5-isovaleroxythymyl isovalerate.

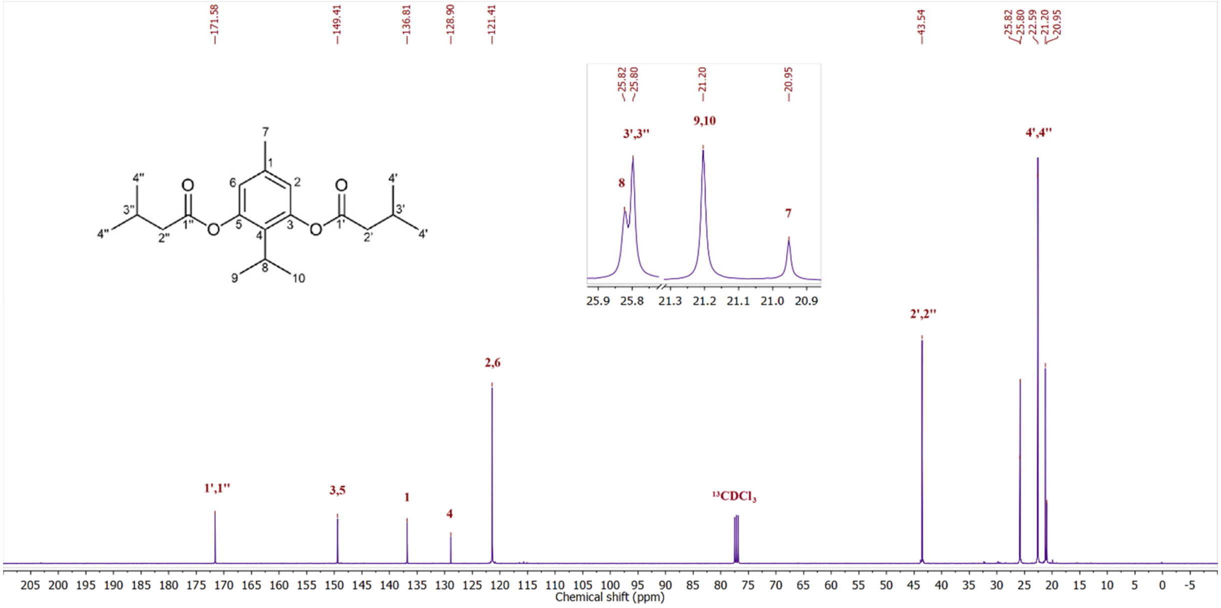

Figure S41. ^{13}C NMR (100.6 MHz, $CDCl_3$) spectrum of 5-isovaleroxythymyl isovalerate.

Table S8. ^{1}H (400 MHz) and ^{13}C (100.6 MHz) NMR data of 5-isovaleroxythymyl isovalerate (chloroform-*d*); NMR parameters are derived from manual iterative full spin analysis, along with the observed gHMBC and NOESY correlations.

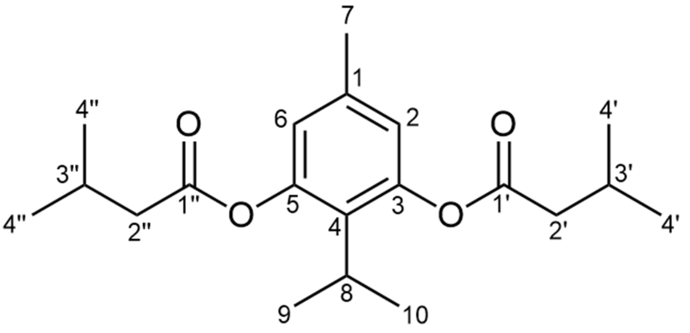

Position	δH (m, *J* (Hz), Integration)	δC	HMBC[a]	NOESY[b]
1	/	136.81	/	/
2 and 6	6.6950 (qd, $^{4}J_{2/6,7}$ = -0.7, $^{5}J_{2/6,8}$ = 0.3, 2 H)	121.41	2,3,4,5,6,7	7
3 and 5	/	149.41	/	/
4	/	128.90	/	/
7	2.2940 (t, $^{4}J_{2/6,7}$ = -0.7, 3 H)	20.95	1,2,6	2,6
8	3.0435 (septt, $^{3}J_{8,9/10}$ = 7.1, $^{5}J_{2/6,8}$ = 0.3, 1 H)	25.82	3,4,5,9,10	9,10
9 and 10	1.2127 (d, $^{3}J_{8,9/10}$ = 7.1, 6 H)	21.20	4,8,9,10	8
1′ and 1′′	/	171.58	/	/
2′ and 2′′	2.4533 (d, $^{3}J_{2'/2'',3'/3''}$ = 7.2, 4 H)	43.54	1′,1′′,3′,3′′,4′,4′′	3′,3′′,4′,4′′
3′ and 3′′	2.2569 (tsept, $^{3}J_{2'/2'',3'/3''}$ = 7.2, $^{3}J_{3'/3'',4'/4''}$ = 6.7, 2 H)	25.80	1′,1′′,2′,2′′,4′,4′′	2′,2′′.4′,4′′
4′ and 4′′	1.0632 (d, $^{3}J_{3'/3'',4'/4''}$ = 6.7, 12 H)	22.59	2′,2′′,3′,3′′,4′,4′′	2′,2′′,3′,3′′

[a] gHMBC correlations observed between the hydrogen in this row and the carbon in the listed position.

[b] Cross-peaks observed in the NOESY spectrum.

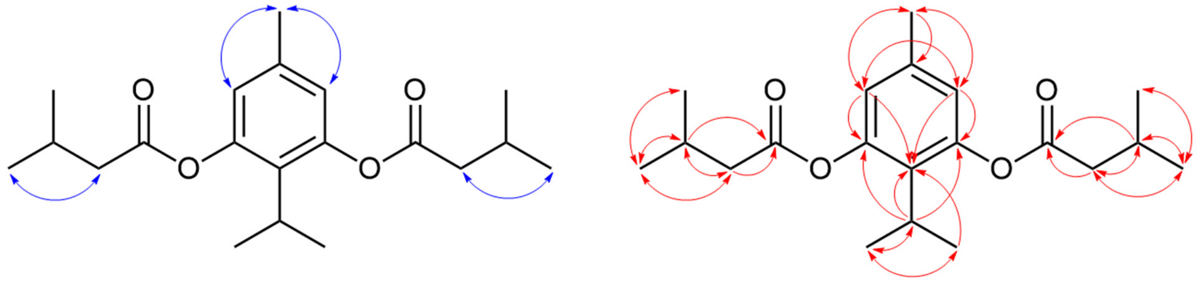

Figure S42. NOESY (blue arrows) and HMBC (red arrows) interactions of 5-isovaleroxythymyl isovalerate.

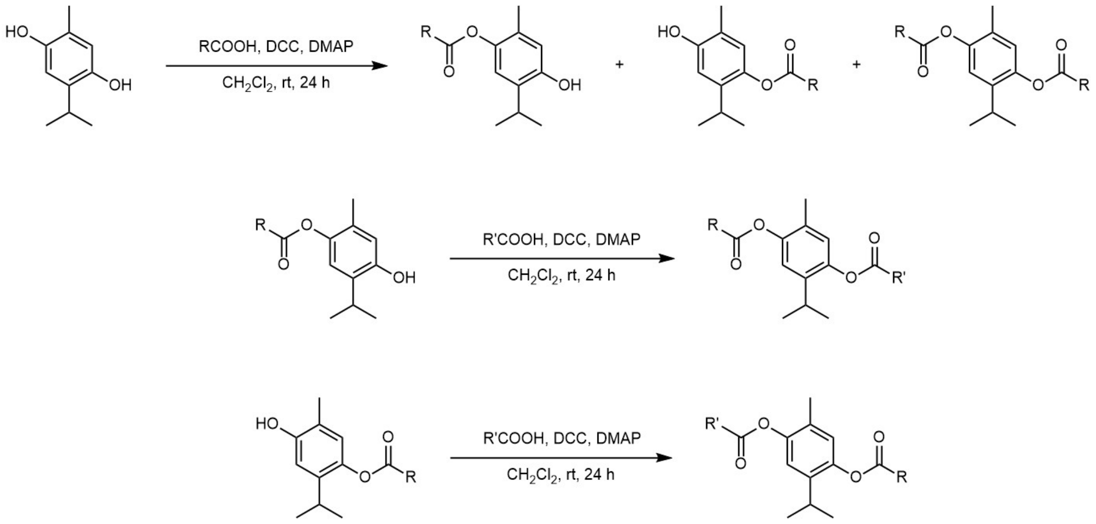

Scheme S3. Synthetic pathway to 6-hydroxythymol-derived monoesters and diesters (R,R' – isopropyl, *sec*-butyl, or isobutyl).

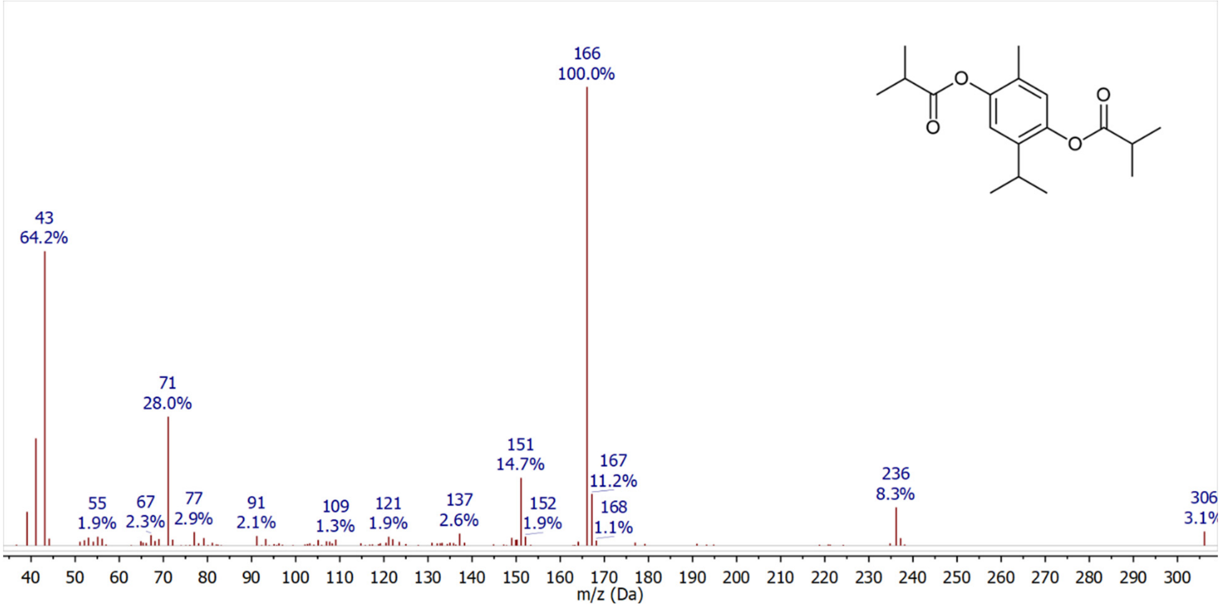

Figure S43. Mass spectrum (EI, 70 eV) of 6-isobutyryloxythymyl isobutyrate.

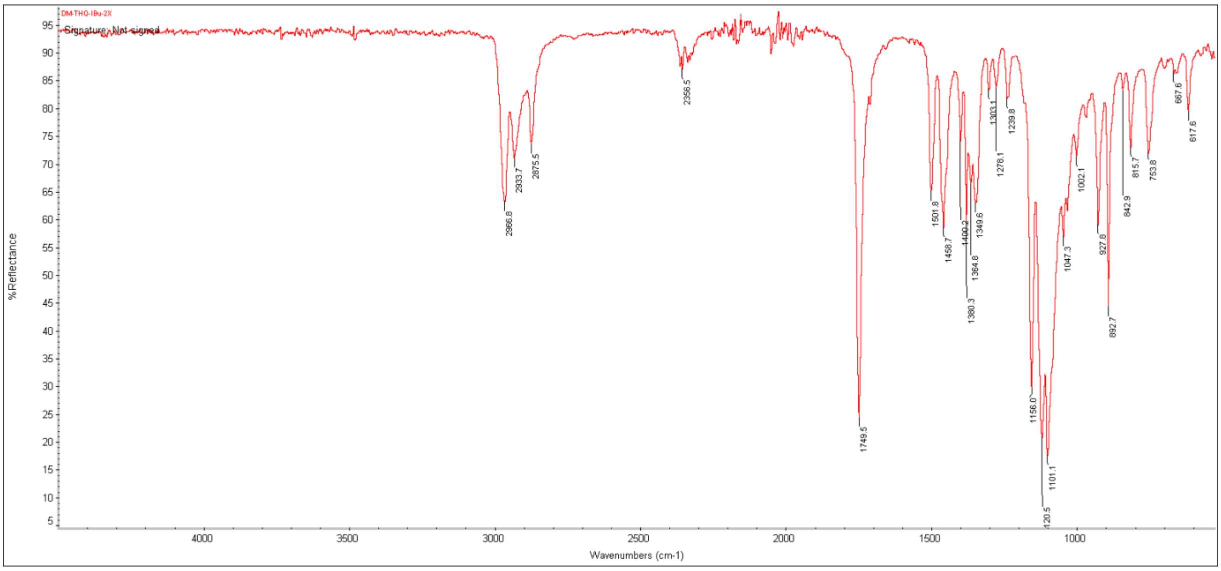

Figure S44. IR spectrum of 6-isobutyryloxythymyl isobutyrate.

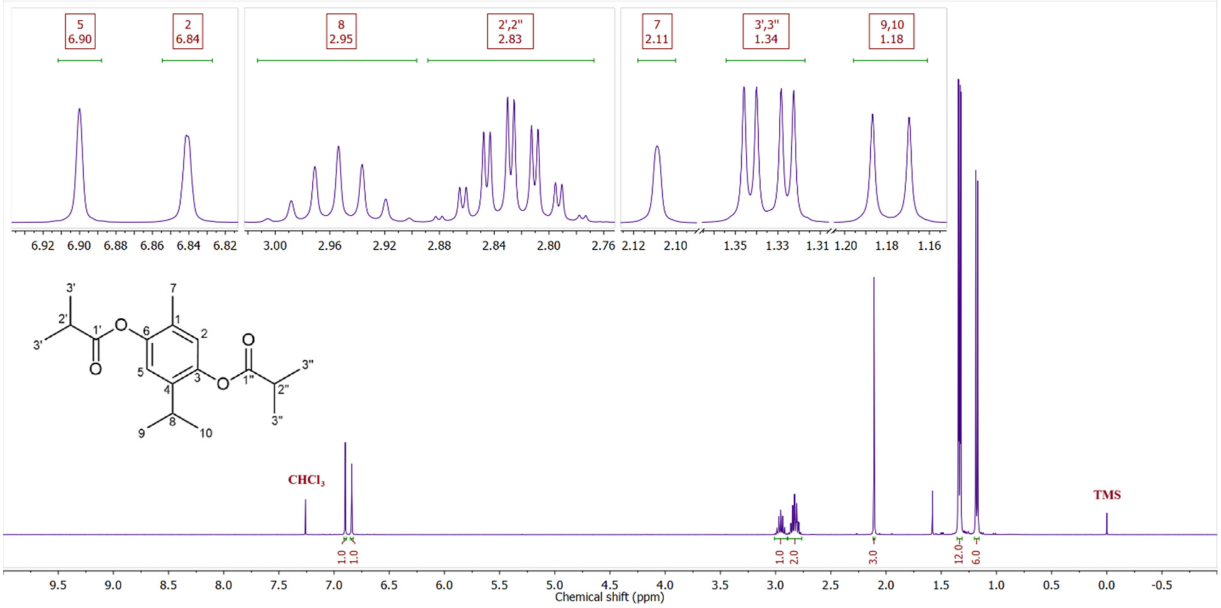

Figure S45. ^1H NMR (400 MHz, $CDCl_3$) spectrum of 6-isobutyryloxythymyl isobutyrate and the corresponding expansions with signal assignment.

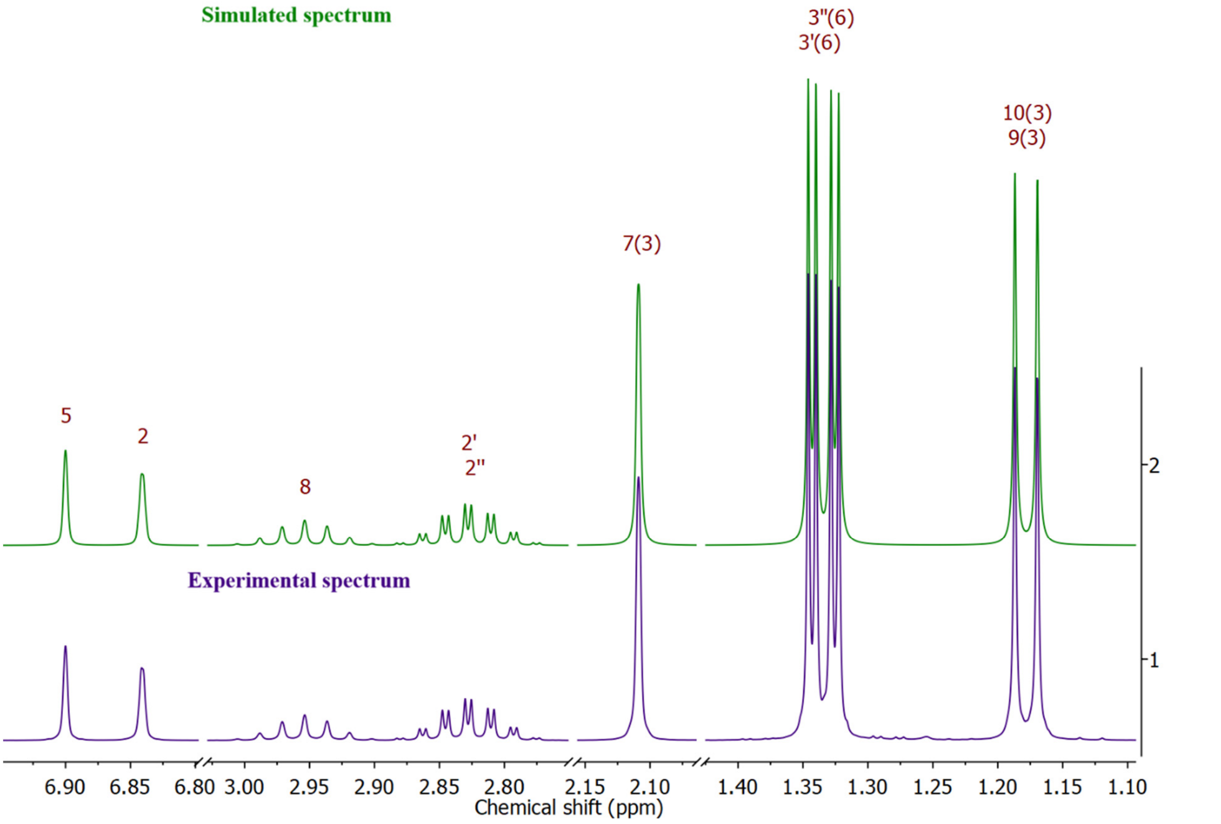

Figure S46. Simulated (manual iterative full spin, MestreNova 11.0.3) and experimental ^1H NMR spectrum of 6-isobutyryloxythymyl isobutyrate.

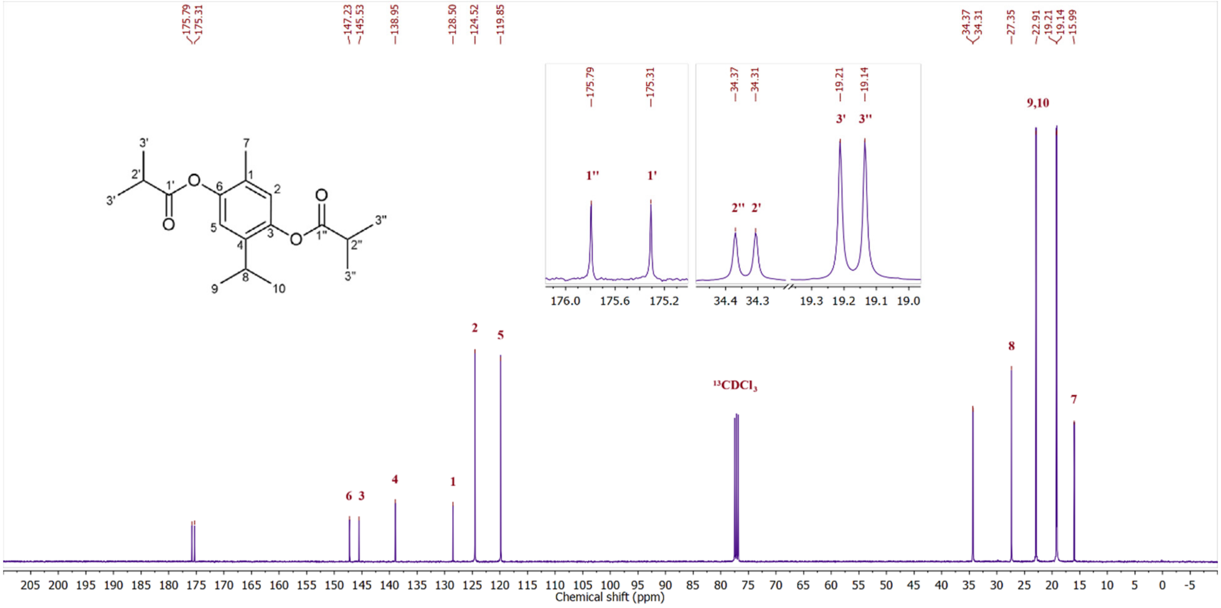

Figure S47. ^{13}C NMR (100.6 MHz, $CDCl_3$) spectrum of 6-isobutyryloxythymyl isobutyrate.

Table S9. ^{1}H (400 MHz) and ^{13}C (100.6 MHz) NMR data of 6-isobutyryloxythymyl isobutyrate (chloroform-*d*); NMR parameters are derived from manual iterative full spin analysis, along with the observed gHMBC and NOESY correlations.

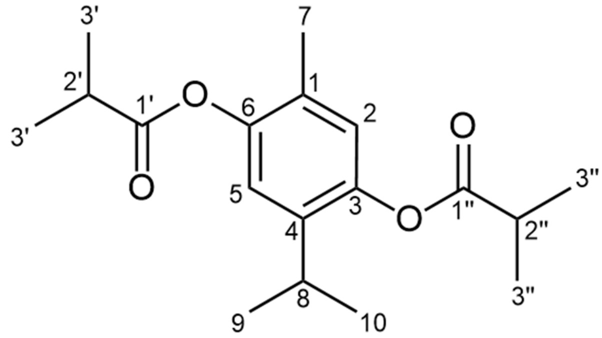

Position	δ_H (m, *J* (Hz), Integration)	δ_C	HMBC[a]	NOESY[b]
1	/	128.50	/	/
2	6.8409 (qdd, $^{4}J_{2,7}$ = -0.7, $^{5}J_{2,5}$ = 0.3, $^{5}J_{2,8}$ = 0.3, 1 H)	124.52	3,4,6,7	7
3	/	145.53	/	/
4	/	138.95	/	/
5	6.9000 (ddq, $^{4}J_{5,8}$ = -0.6, $^{5}J_{2,5}$ = 0.3, $^{5}J_{5,7}$ = 0.3, 1 H)	119.85	1,3,6,8	9,10
6	/	147.23	/	/
7	2.1089 (dd, $^{4}J_{2,7}$ = -0.7, $^{5}J_{5,7}$ = 0.3, 3 H)	15.99	1,2,6	2
8	2.9535 (septdd, $^{3}J_{8,9/10}$ = 6.9, $^{4}J_{5,8}$ = -0.6, $^{5}J_{2,8}$ = 0.3, 1 H)	27.35	3,4,5,9,10	9,10
9 and 10	1.1782 (d, $^{3}J_{8,9/10}$ = 6.9, 6 H)	22.91	4,8,9,10	5,8,9,10
1′	/	175.31	/	/
2′	2.8301 (sept, $^{3}J_{2',3'}$ = 7.0, 1 H)	34.31	1′,3′	3′

3′	1.3372 (d, $^3J_{2',3'}$ = 7.0, 6 H)	19.21	1′,2′,3′	2′,3′
1′′	/	175.79	/	/
2′′	2.8253 (sept, $^3J_{2'',3''}$ = 7.0, 1 H)	34.37	1′′,3′′	3′′
3′′	1.3313 (d, $^3J_{2'',3''}$ = 7.0, 6 H)	19.14	1′′,2′′,3′′	2′′,3′′

[a] gHMBC correlations observed between the hydrogen in this row and the carbon in the listed position.

[b] Cross-peaks observed in the NOESY spectrum.

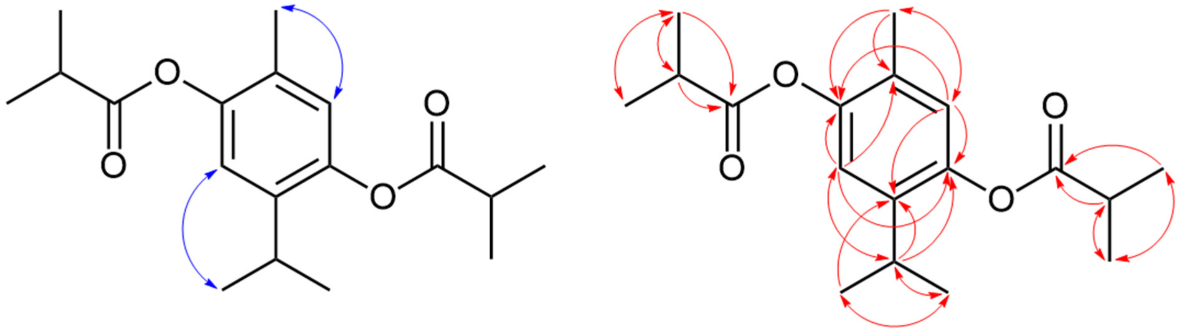

Figure S48. NOESY (blue arrows) and HMBC (red arrows) interactions of 6-isobutyryloxythymyl isobutyrate.

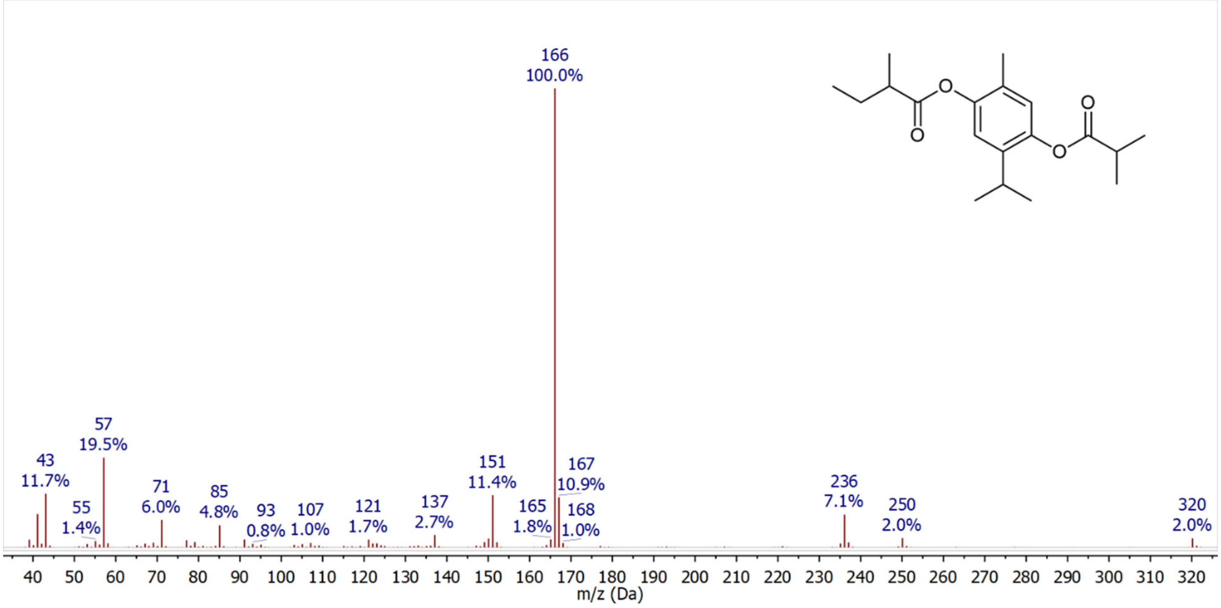

Figure S49. Mass spectrum (EI, 70 eV) of 6-(2-methylbutyryloxy)thymyl isobutyrate.

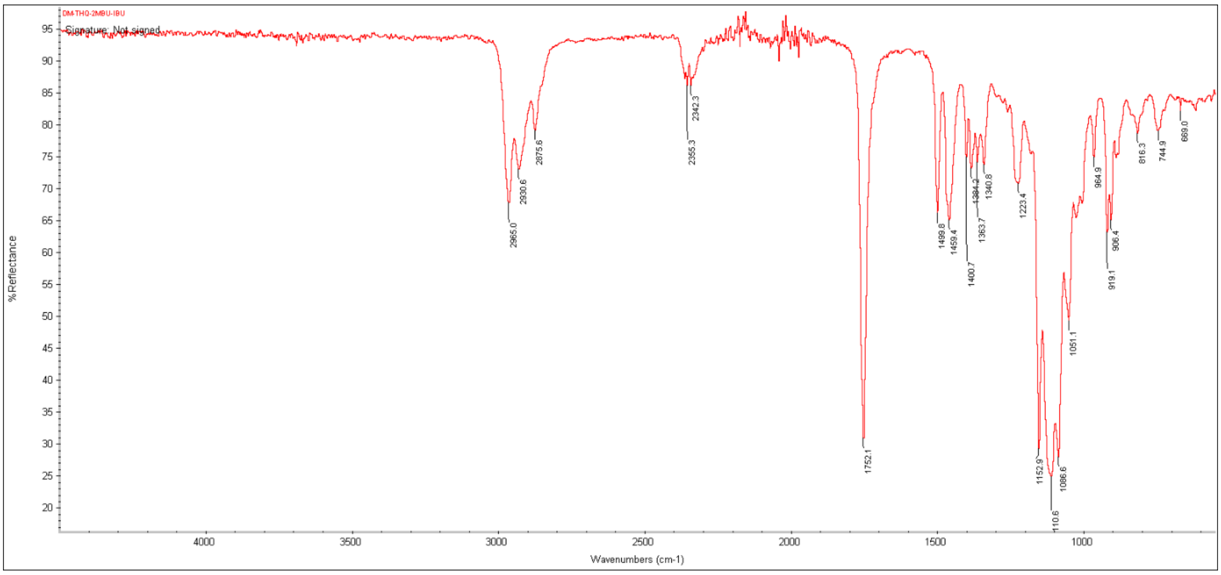

Figure S50. IR spectrum of 6-(2-methylbutyryloxy)thymyl isobutyrate.

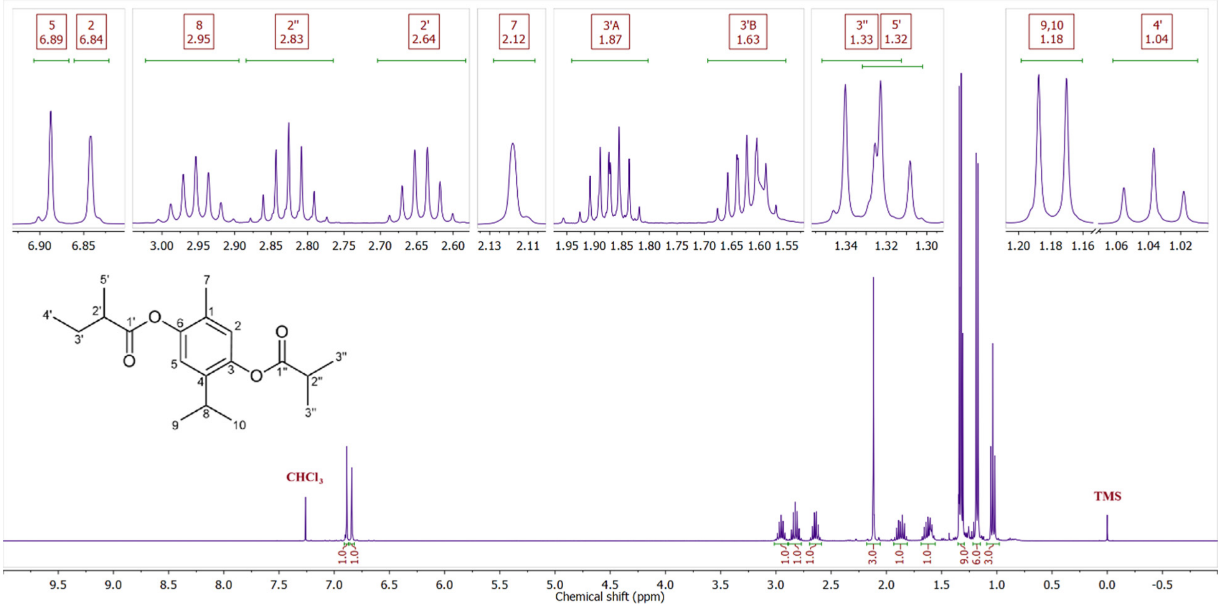

Figure S51. ^{1}H NMR (400 MHz, $CDCl_3$) spectrum of 6-(2-methylbutyryloxy)thymyl isobutyrate and the corresponding expansions with signal assignment.

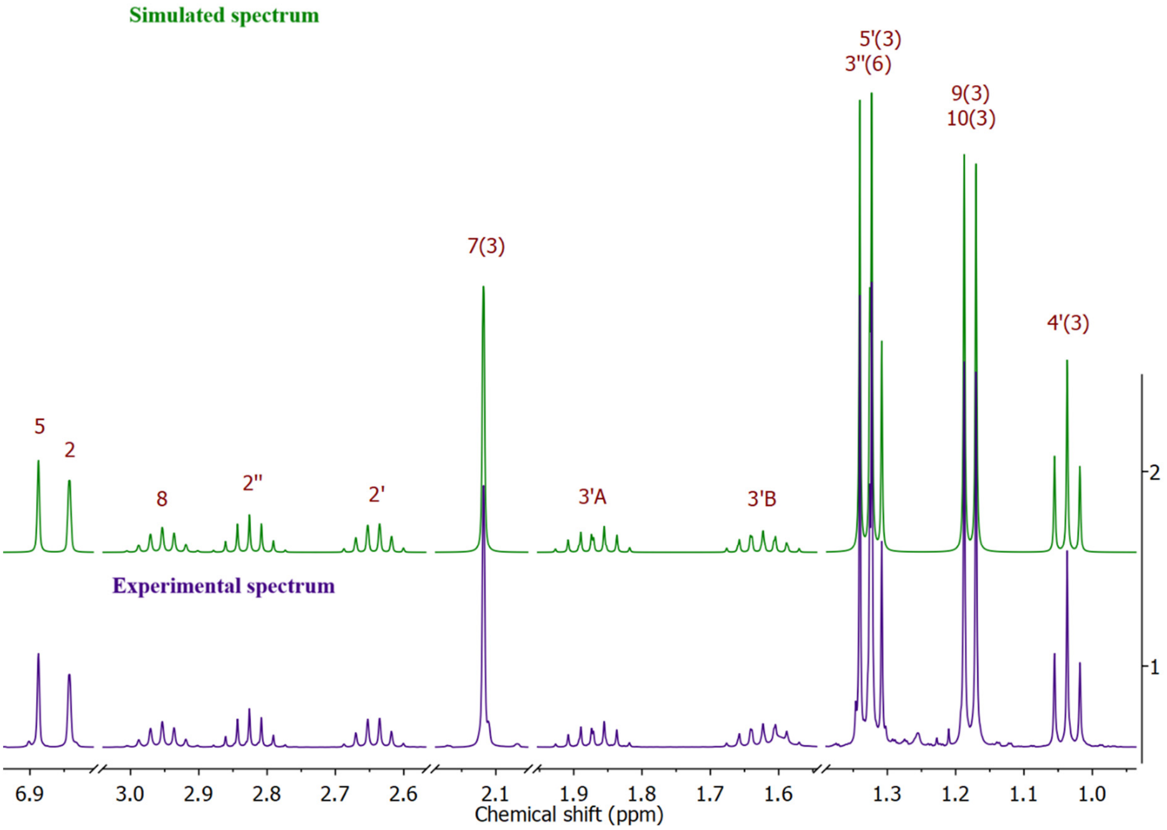

Figure S52. Simulated (manual iterative full spin, MestreNova 11.0.3) and experimental ^{1}H NMR spectrum of 6-(2-methylbutyryloxy)thymyl isobutyrate.

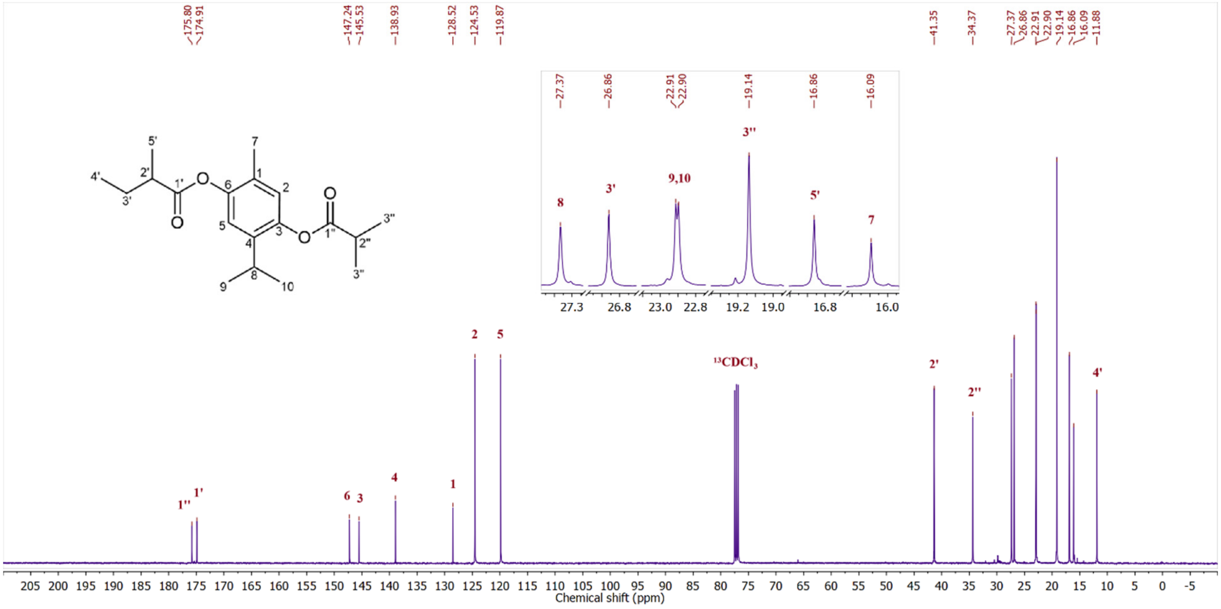

Figure S53. ^{13}C NMR (100.6 MHz, $CDCl_3$) spectrum of 6-(2-methylbutyryloxy)thymyl isobutyrate.

Table S10. ^{1}H (400 MHz) and ^{13}C (100.6 MHz) NMR data of 6-(2-methylbutyryloxy)thymyl isobutyrate (chloroform-*d*); NMR parameters are derived from manual iterative full spin analysis, along with the observed gHMBC and NOESY correlations.

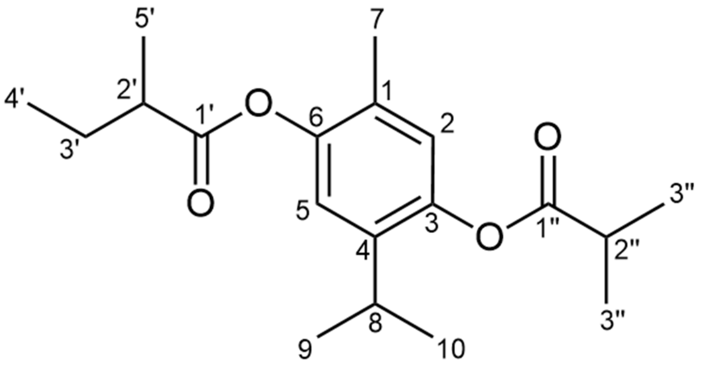

Position	δ_H (m, J (Hz), Integration)	δ_C	HMBC[a]	NOESY[b]
1	/	128.52	/	/
2	6.8422 (qd, $^{4}J_{6,7}$ = -0.7, $^{5}J_{3,6}$ = 0.3, 1 H)	124.53	3,4,6,7	7
3	/	145.53	/	/
4	/	138.93	/	/
5	6.8876 (ddq, $^{4}J_{5,8}$ = -0.6, $^{5}J_{2,5}$ = 0.3, $^{5}J_{5,7}$ = 0.3, 1 H)	119.87	1,3,6,8	9,10
6	/	147.24	/	
7	2.1182 (dd, $^{4}J_{2,7}$ = -0.7, $^{5}J_{2,7}$ = 0.3, 3 H)	16.09	1,2,6	2
8	2.9532 (septd, $^{3}J_{8,9/10}$ = 6.9, $^{4}J_{5,8}$ = -0.6, 1 H)	27.37	3,4,5,9,10	9,10
9 and 10	1.1789 (d, $^{3}J_{8,9/10}$ = 6.9, 6 H)	22.90/22.91	4,8,9,10	5,8,9,10
1′	/	174.91	/	/
2′	2.6437 (dqd, $^{3}J_{2',3'A}$ = 7.5, $^{3}J_{2',5'}$ = 7.0, $^{3}J_{2',3'B}$ = 6.5, $^{4}J_{2',4'}$ = 0.3, 1 H)	41.35	1′,3′,4′,5′	3′,4′,5′
3′A	1.8711 (ddq, $^{2}J_{3'A,3'B}$ = -13.7, $^{3}J_{2',3'A}$ = 7.5, $^{3}J_{3'A,4'}$ = 7.5, 1 H)	26.86	1′,2′,4′,5′	2′,4′,5′
3′B	1.6238 (dqd, $^{2}J_{3'A,3'B}$ = -13.7, $^{3}J_{3'B,4'}$ = 7.5, $^{3}J_{2',3'B}$ = 6.5, 1 H)	26.86	1′,2′,4′,5′	2′,4′,5′
4′	1.0370 (ddd, $^{3}J_{3'A,4'}$ = 7.5, $^{3}J_{3'B,4'}$ = 7.5, $^{4}J_{2',4'}$ = 0.3, 3 H)	11.88	2′,3′	2′,3′
5′	1.3170 (d, $^{3}J_{2',5'}$ = 7.0, 3 H)	16.86	1′,2′,3′	2′,3′
1′′	/	175.80	/	/
2′′	2.8256 (sept, $^{3}J_{2'',3''}$ = 7.0, 1 H)	34.37	1′′,3′′	3′′
3′′	1.3317 (d, $^{3}J_{2'',3''}$ = 7.0, 6 H)	19.14	1′′,2′′,3′′	2′′,3′′

[a] gHMBC correlations observed between the hydrogen in this row and the carbon in the listed position.

[b] Cross-peaks observed in the NOESY spectrum.

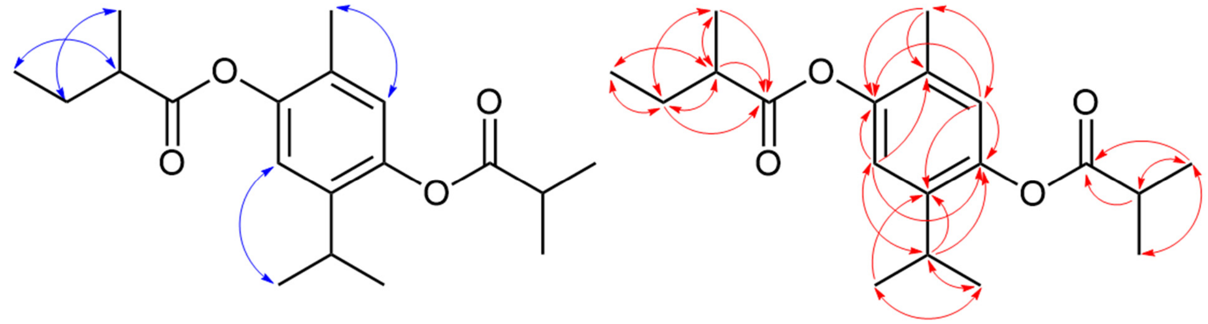

Figure S54. NOESY (blue arrows) and HMBC (red arrows) interactions of 6-(2-methylbutyryloxy)thymyl isobutyrate.

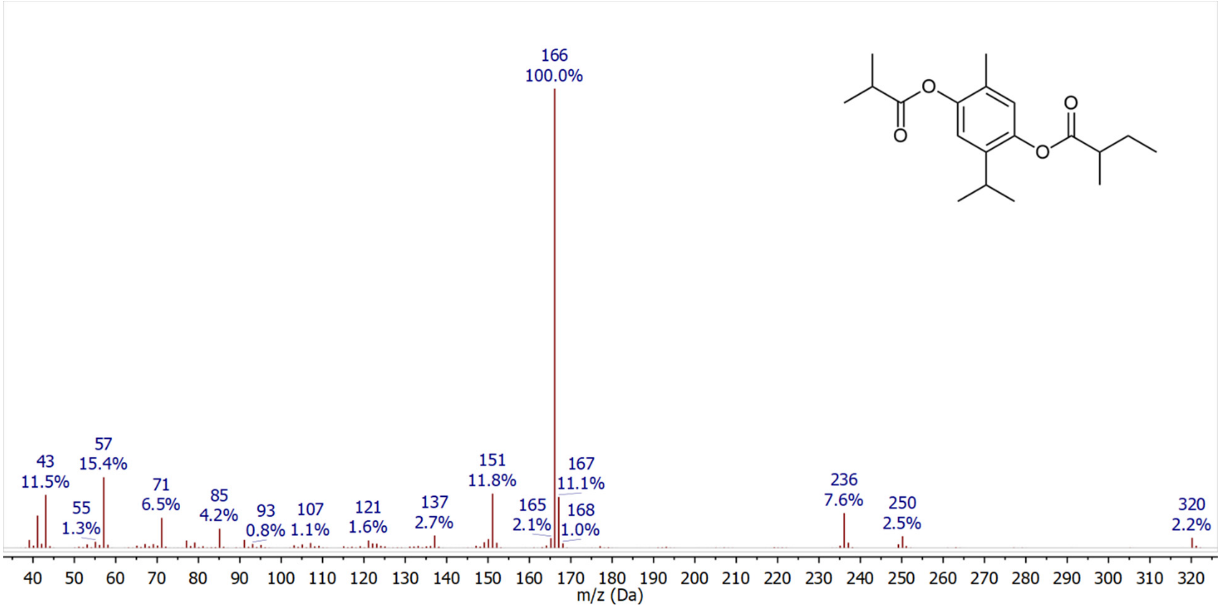

Figure S55. Mass spectrum (EI, 70 eV) of 6-isobutyryloxythymyl 2-methylbutyrate.

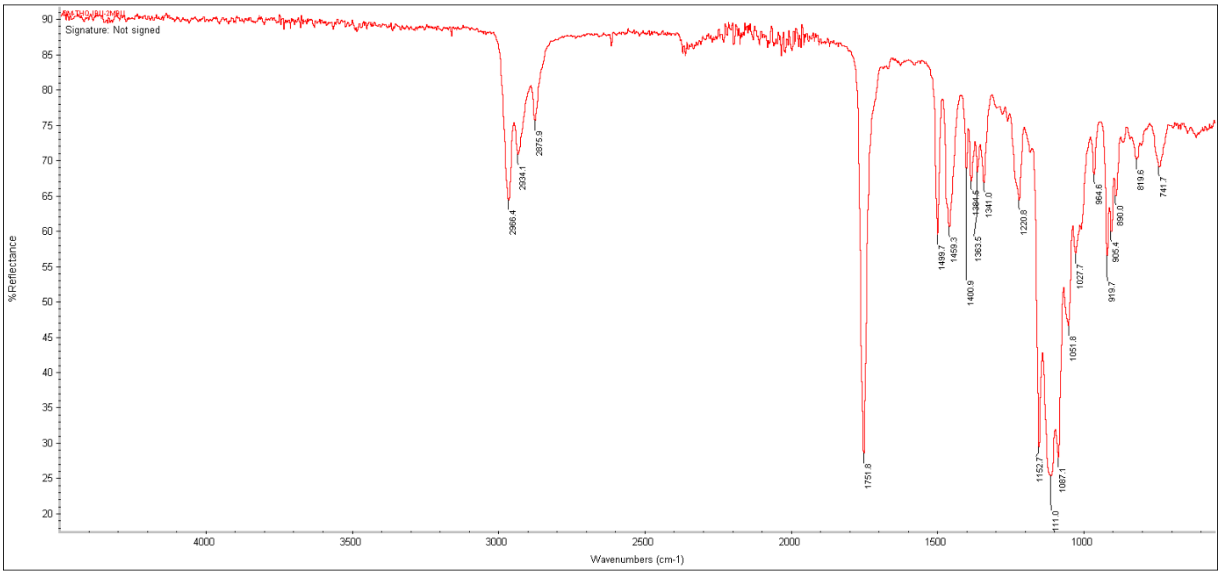

Figure S56. IR spectrum of 6-isobutyryloxythymyl 2-methylbutyrate.

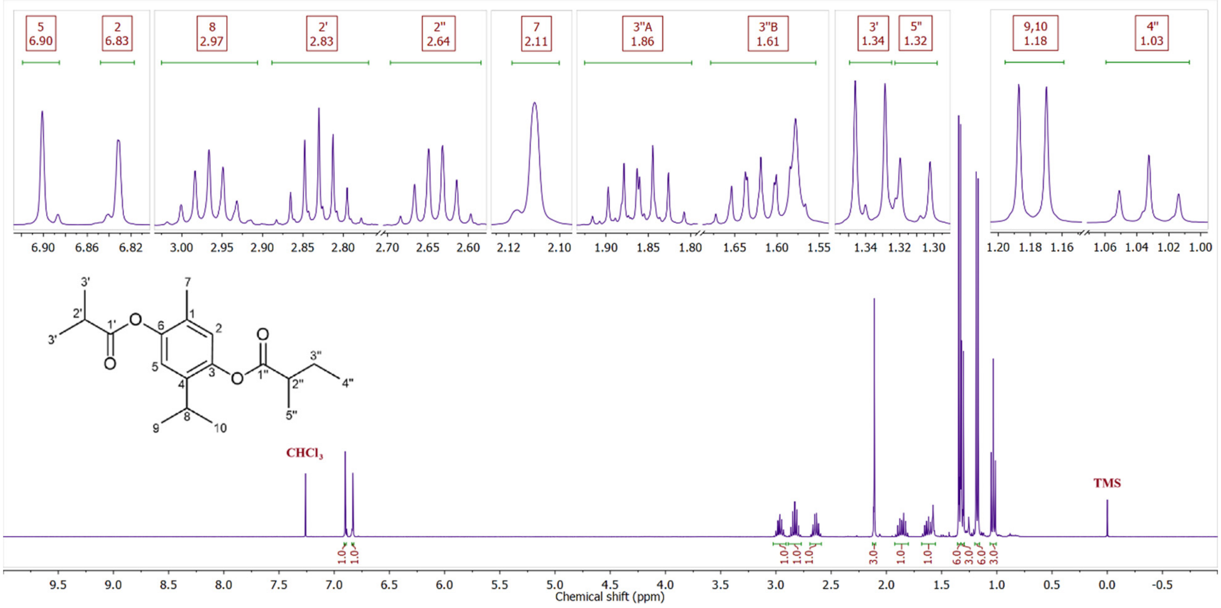

Figure S57. ^{1}H NMR (400 MHz, $CDCl_3$) spectrum of 6-isobutyryloxythymyl 2-methylbutyrate and the corresponding expansions with signal assignment.

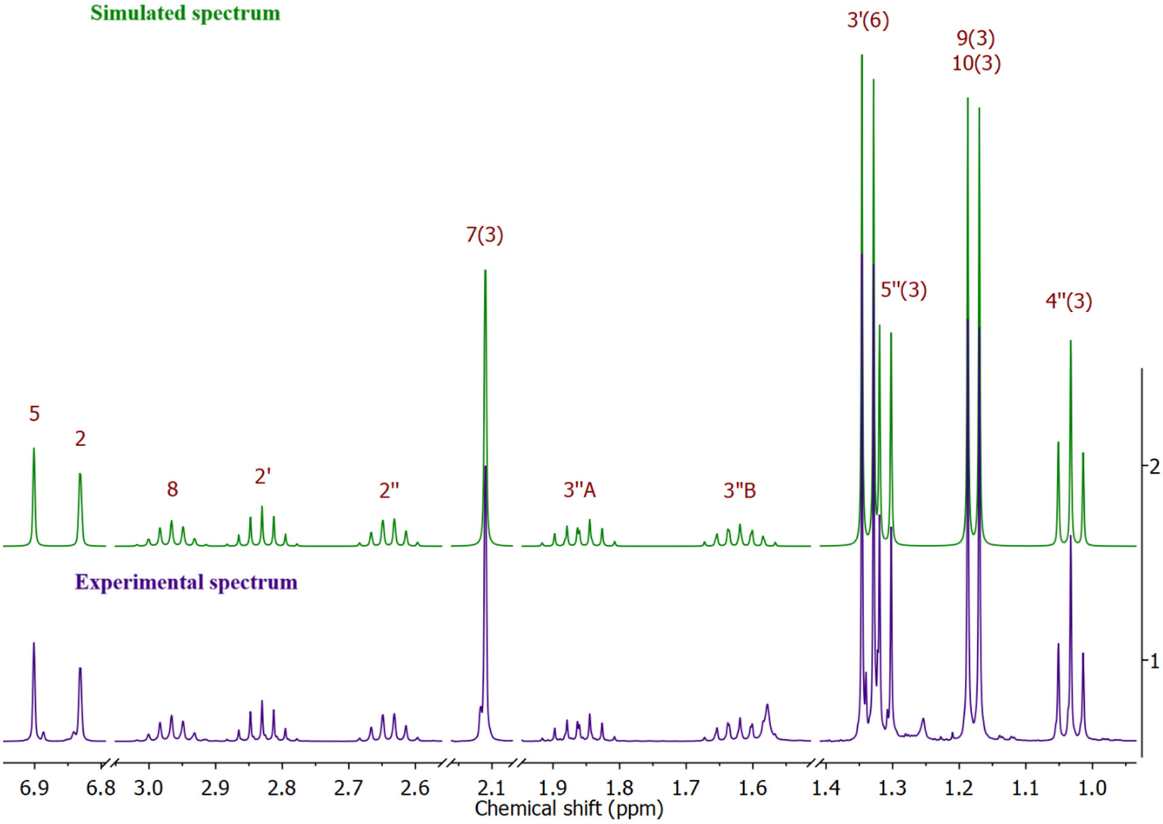

Figure S58. Simulated (manual iterative full spin, MestreNova 11.0.3) and experimental ^{1}H NMR spectrum of 6-isobutyryloxythymyl 2-methylbutyrate.

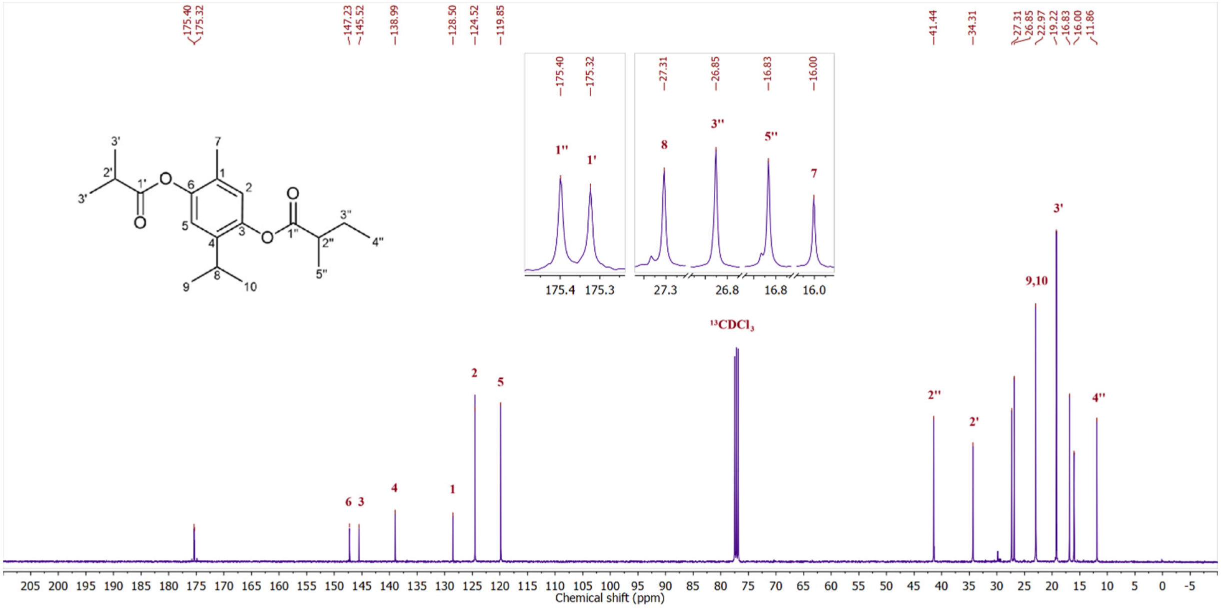

Figure S59. ^{13}C NMR (100.6 MHz, $CDCl_3$) spectrum of 6-isobutyryloxythymyl 2-methylbutyrate.

Table S11. ^{1}H (400 MHz) and ^{13}C (100.6 MHz) NMR data of 6-isobutyryloxythymyl 2-methylbutyrate (chloroform-*d*); NMR parameters are derived from manual iterative full spin analysis, along with the observed gHMBC and NOESY correlations.

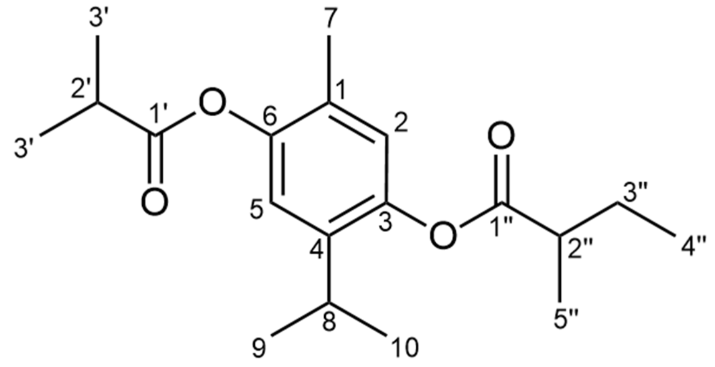

Position	δ_H (m, *J* (Hz), Integral)	δ_C	HMBC[a]	NOESY[b]
1	/	128.50	/	/
2	6.8315 (qd, $^{4}J_{2,7}$ = -0.7, $^{5}J_{2,5}$ = 0.3, 1 H)	124.52	3,4,6,7	7
3	/	145.52	/	/
4	/	138.99	/	/
5	6.9010 (ddq, $^{4}J_{5,8}$ = -0.6, $^{5}J_{2,5}$ = 0.3, $^{5}J_{5,7}$ = 0.3, 1 H)	119.85	1,3,6,8	9,10
6	/	147.23	/	/
7	2.1099 (dd, $^{4}J_{2,7}$ = -0.7, $^{5}J_{2,7}$ = 0.3, 3 H)	16.00	1,2,6	2
8	2.9660 (septd, $^{3}J_{8,9/10}$ = 6.9, $^{4}J_{5,8}$ = -0.6, 1 H)	27.31	3,4,5,9,10	9,10
9 and 10	1.1785 (d, $^{3}J_{8,9/10}$ = 6.9, 6 H)	22.97	4,8,9,10	5,8,9,10
1′	/	175.32	/	/
2′	2.8300 (sept, $^{3}J_{2',3'}$ = 7.0, 1 H)	34.31	1′,3′	3′
3′	1.3373 (d, $^{3}J_{2',3'}$ = 7.0, 6 H)	19.22	1′,2′,3′	2′,3′
1′′	/	175.40	/	/
2′′	2.6400 (dqd, $^{3}J_{2'',3''A}$ = 7.5, $^{3}J_{2'',5''}$ = 7.0, $^{3}J_{2'',3''B}$ = 6.5, $^{4}J_{2'',4''}$ = 0.3, 1 H)	41.44	1′′,3′′,4′′,5′′	3′′,4′′,5′′
3′′A	1.8604 (ddq, $^{2}J_{3''A,3''B}$ = -13.7, $^{3}J_{2'',3''A}$ = 7.5, $^{3}J_{3''A,4''}$ = 7.5, 1 H)	26.85	1′′,2′′,4′′,5′′	2′′,4′′,5′′
3′′B	1.6201 (dqd, $^{2}J_{3''A,3''B}$ = -13.7, $^{3}J_{3''B,4''}$ = 7.5, $^{3}J_{2'',3''B}$ = 6.5, 1 H)	26.85	1′′,2′′,4′′,5′′	2′′,4′′,5′′
4′′	1.0327 (ddd, $^{3}J_{3''A,4''}$ = 7.5, $^{3}J_{3''B,4''}$ = 7.5, $^{4}J_{2'',4''}$ = 0.3, 3 H)	11.86	2′′,3′′	2′′,3′′
5′′	1.3110 (d, $^{3}J_{2'',5''}$ = 7.0, 3 H)	16.83	1′′,2′′,3′′	2′′,3′′

[a] gHMBC correlations observed between the hydrogen in this row and the carbon in the listed position.

[b] Cross-peaks observed in the NOESY spectrum.

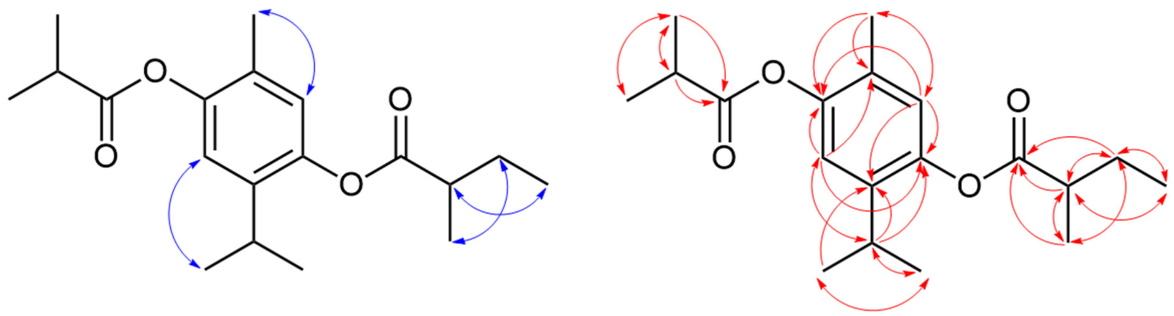

Figure S60. NOESY (blue arrows) and HMBC (red arrows) interactions of 6-isobutyryloxythymyl 2-methylbutyrate.

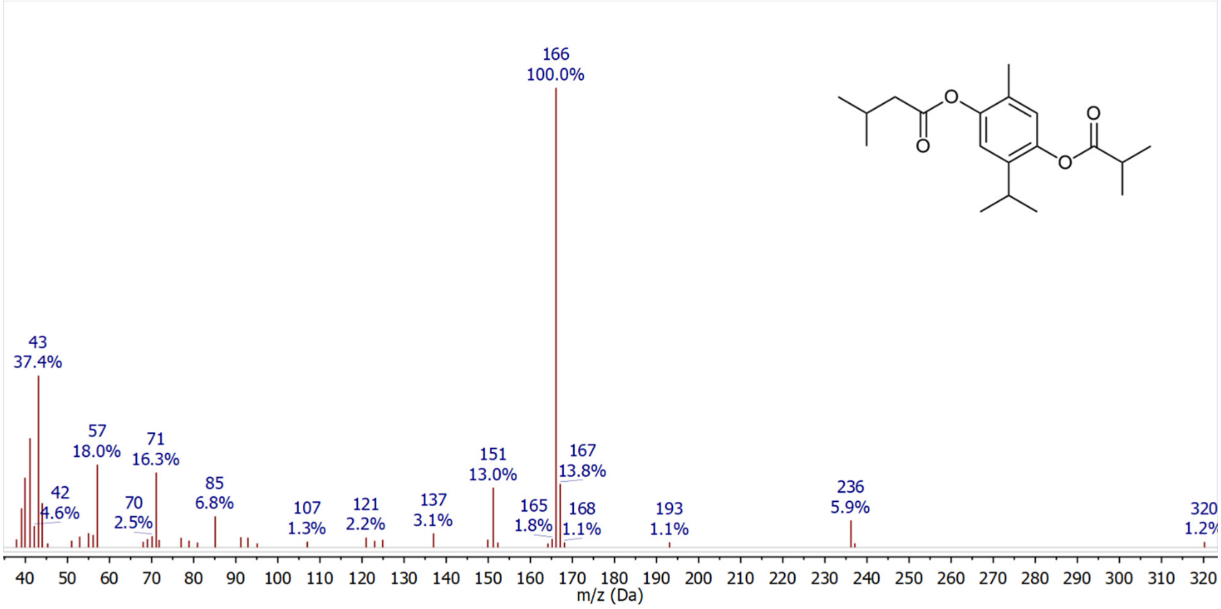

Figure S61. Mass spectrum (EI, 70 eV) of 6-isovaleroxythymyl isobutyrate.

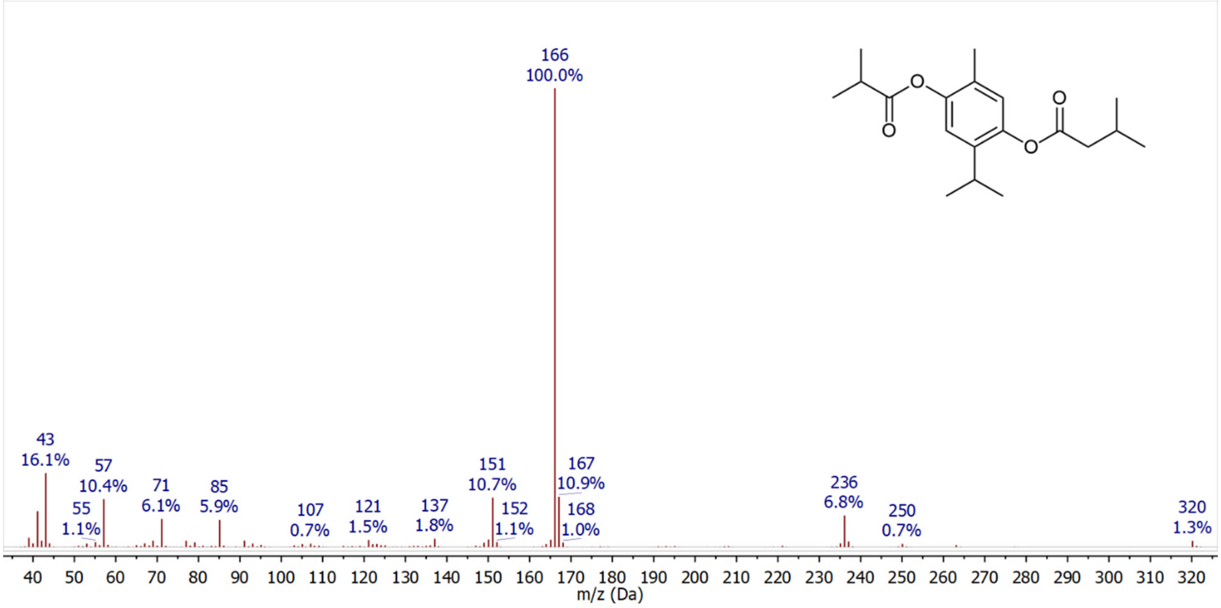

Figure S62. Mass spectrum (EI, 70 eV) of 6-isobutyryloxythymyl isovalerate.

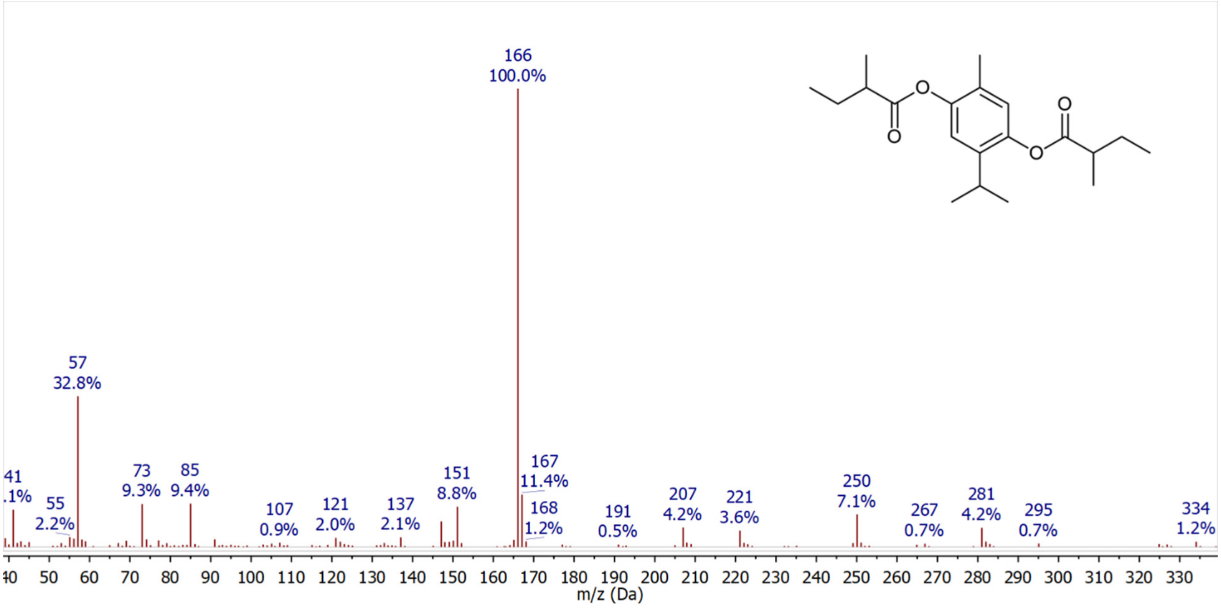

Figure S63. Mass spectrum (EI, 70 eV) of 6-(2-methylbutyryloxy)thymyl 2-methylbutyrate.

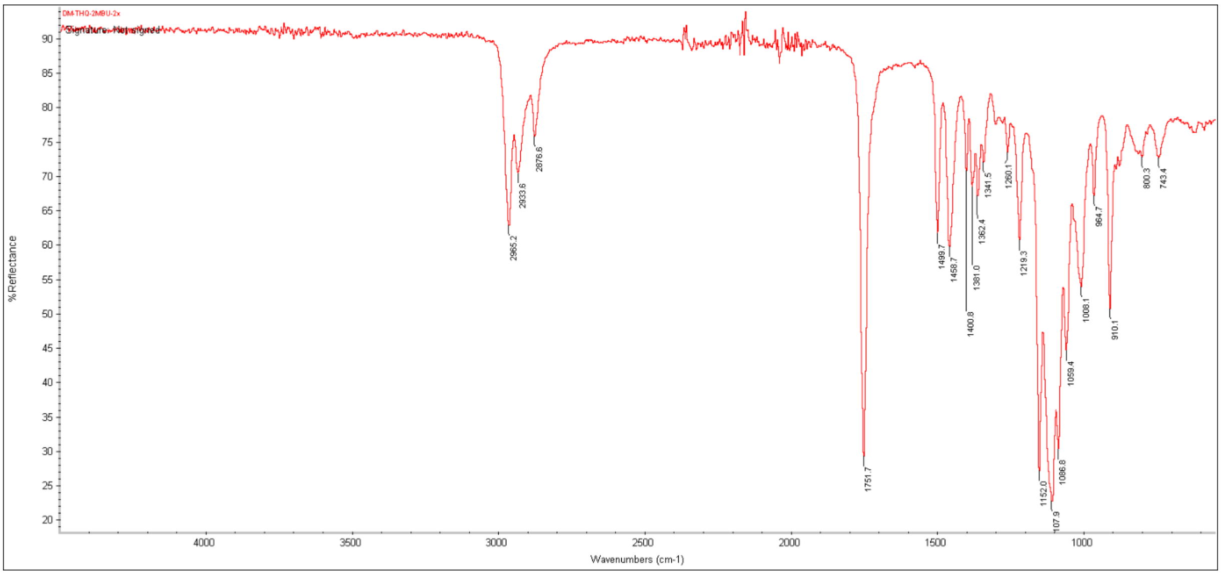

Figure S64. IR spectrum of 6-(2-methylbutyryloxy)thymyl 2-methylbutyrate.

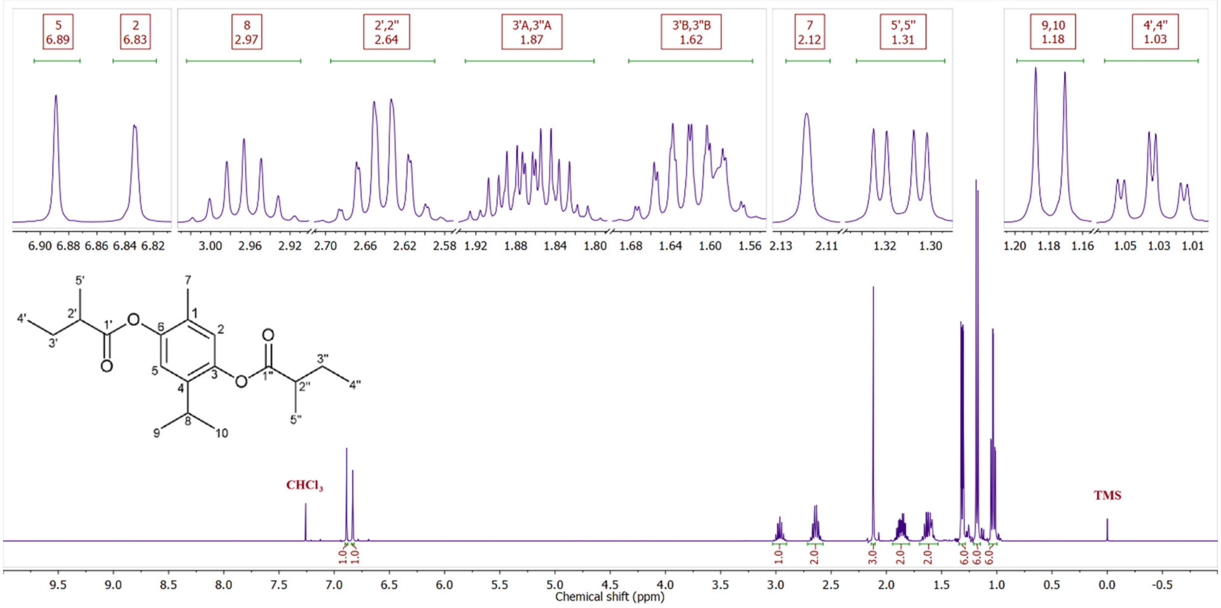

Figure S65. ^{1}H NMR (400 MHz, $CDCl_3$) spectrum of 6-(2-methylbutyryloxy)thymyl 2-methylbutyrate and the corresponding expansions with signal assignment.

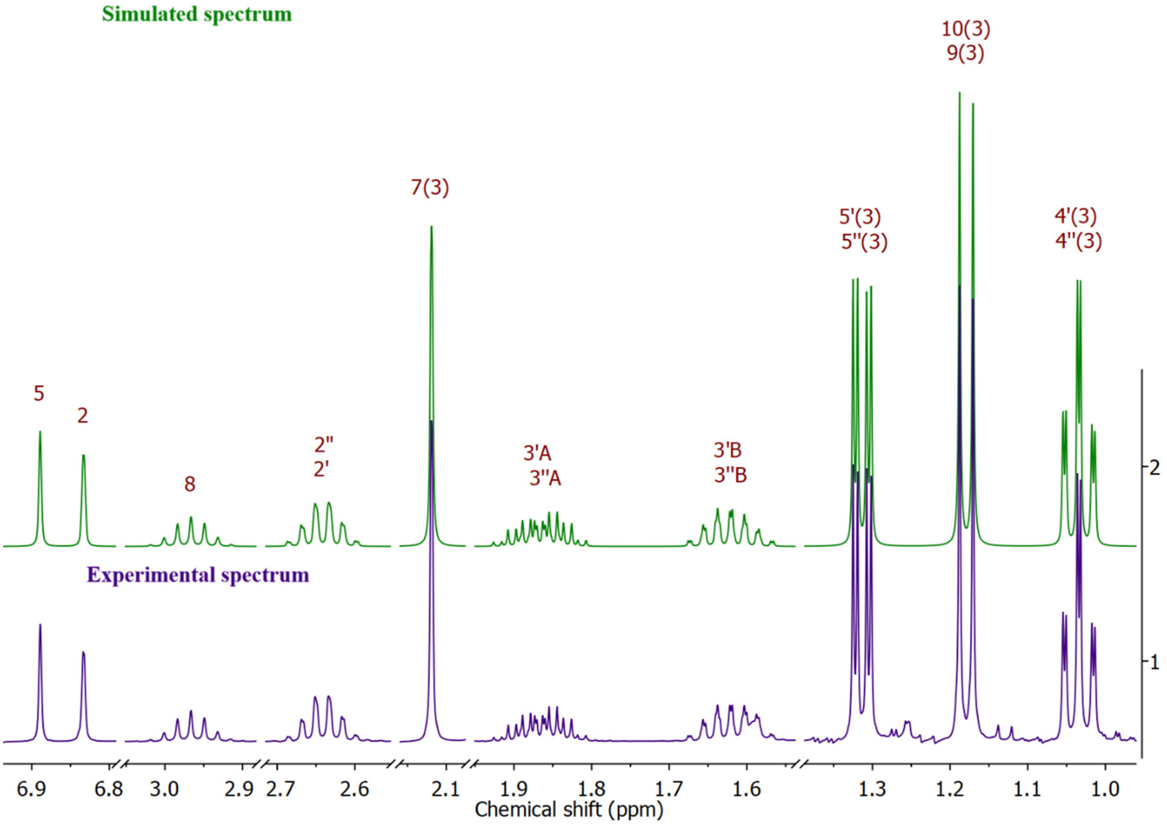

Figure S66. Simulated (manual iterative full spin, MestreNova 11.0.3) and experimental ^{1}H NMR spectrum of 6-(2-methylbutyryloxy)thymyl 2-methylbutyrate.

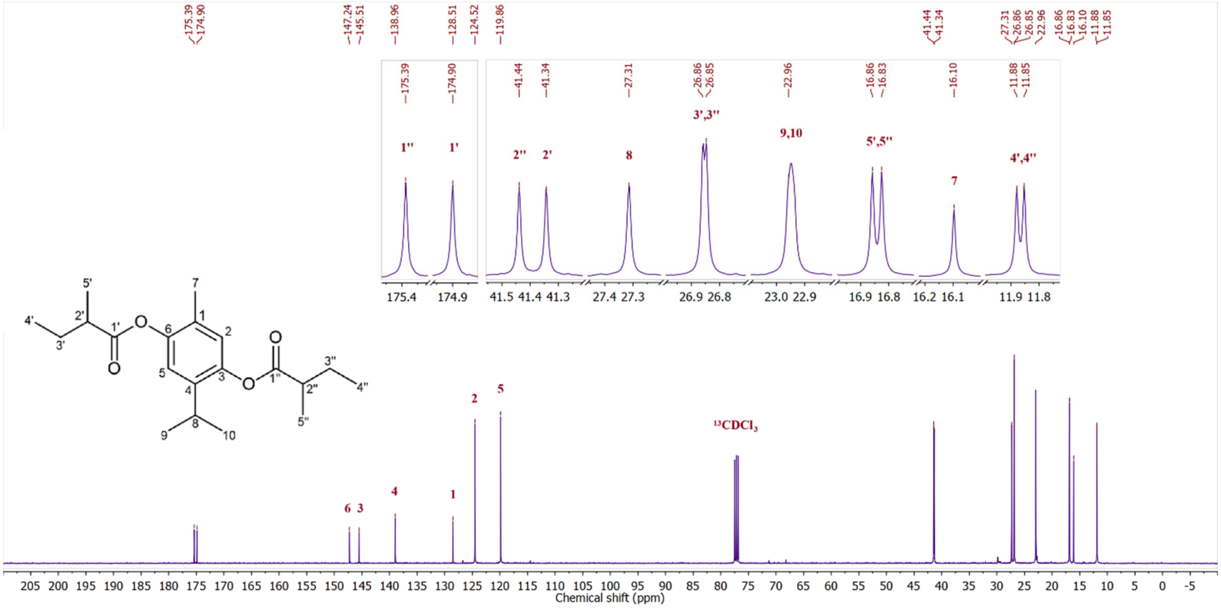

Figure S67. ^{13}C NMR (100.6 MHz, $CDCl_3$) spectrum of 6-(2-methylbutyryloxy)thymyl 2-methylbutyrate.

Table S12. ^{1}H (400 MHz) and ^{13}C (100.6 MHz) NMR data of 6-(2-methylbutyryloxy)thymyl 2-methylbutyrate (chloroform-*d*); NMR parameters are derived from manual iterative full spin analysis, along with the observed gHMBC and NOESY correlations.

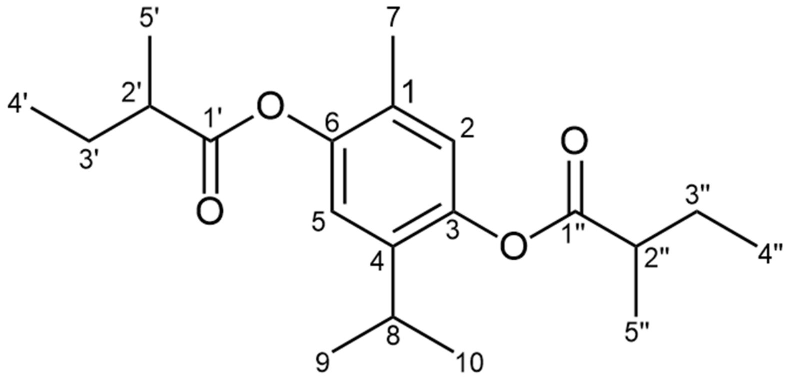

Position	δ_H (m, J (Hz), Integration)	δ_C	HMBC[a]	NOESY[b]
1	/	128.51	/	/
2	6.8330 (qd, $^4J_{2,7}$ = -0.7, $^5J_{2,5}$ = 0.3 H)	124.52	3,4,6,7	7
3	/	145.51	/	/
4	/	138.96	/	/
5	6.8890 (ddq, $^4J_{5,8}$ = -0.6, $^5J_{2,5}$ = 0.3, $^5J_{5,7}$ = 0.3, 1 H)	119.86	1,3,6,8	9,10
6	/	147.24	/	/
7	2.1189 (dd, $^4J_{2,7}$ = -0.7, $^5J_{5,7}$ = 0.3, 3 H)	16.10	1,2,6	2
8	2.9661 (septd, $^3J_{8,9/10}$ = 6.9, $^4J_{5,8}$ = -0.6, 1 H)	27.31	3,4,5,9,10	9,10
9 and 10	1.1792 (d, $^3J_{8,9/10}$ = 6.9, 6 H)	22.96	4,8,9,10	5,8,9,10
1′	/	174.90	/	/
2′	2.6429 (dqd, $^3J_{2',3'A}$ = 7.5, $^3J_{2',5'}$ = 7.0, $^3J_{2',3'B}$ = 6.5, $^4J_{2',4'}$ = 0.3, 1 H)	41.34	1′,3′,4′,5′	3′,4′,5′
3′A	1.8705 (ddq, $^2J_{3'A,3'B}$ = -13.7, $^3J_{2',3'A}$ = 7.5, $^3J_{3'A,4'}$ = 7.5, 1 H)	26.86[c]	1′,2′,4′,5′	2′,4′,5′
3′B	1.6230 (dqd, $^2J_{3'A,3'B}$ = -13.7, $^3J_{3'B,4'}$ = 7.5, $^3J_{2',3'B}$ = 6.5, 1 H)	26.86[c]	1′,2′,4′,5′	2′,4′,5′
4′	1.0363 (ddd, $^3J_{3'A,4'}$ = 7.5, $^3J_{3'B,4'}$ = 7.5, $^4J_{2',4'}$ = 0.3, 3 H)	11.88[d]	2′,3′	2′,3′
5′	1.3163 (d, $^3J_{2',5'}$ = 7.0, 3 H)	16.86[e]	1′,2′,3′	2′,3′
1″	/	175.39	/	/
2″	2.6394 (dqd, $^3J_{2'',3''A}$ = 7.5, $^3J_{2'',5''}$ = 7.0, $^3J_{2'',3''B}$ = 6.5, $^4J_{2'',4''}$ = 0.3, 1 H)	41.44	1″,3″,4″,5″	3″,4″,5″
3″A	1.8600 (ddq, $^2J_{3''A,3''B}$ = -13.7, $^3J_{2'',3''A}$ = 7.5, $^3J_{3''A,4''}$ = 7.5, 1 H)	26.85[c]	1″,2″,4″,5″	2″,4″,5″
3″B	1.6196 (dqd, $^2J_{3''A,3''B}$ = -13.7, $^3J_{3''B,4''}$ = 7.5, $^3J_{2'',3''B}$ = 6.5, 1 H)	26.85[c]	1″,2″,4″,5″	2″,4″,5″
4″	1.0323 (ddd, $^3J_{3''A,4''}$ = 7.5, $^3J_{3''B,4''}$ = 7.5, $^4J_{2'',4''}$ = 0.3, 3 H)	11.85[d]	2″,3″	2″,3″
5″	1.3105 (d, $^3J_{2'',5''}$ = 7.0, 3 H)	16.83[e]	1″,2″,3″	2″,3″

[a] gHMBC correlations observed between the hydrogen in this row and the carbon in the listed position.

[b] Cross-peaks observed in the NOESY spectrum.

[c] The signals of C-3′ and C-3″ may be interchanged.

[d] The signals of C-4′ and C-4″ may be interchanged.

[e] The signals of C-5′ and C-5′′ may be interchanged.

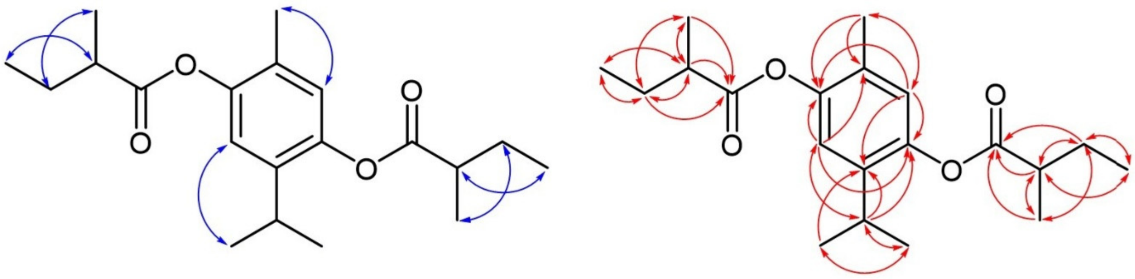

Figure S68. NOESY (blue arrows) and HMBC (red arrows) interactions of 6-(2-methylbutyryloxy)thymyl 2-methylbutyrate.

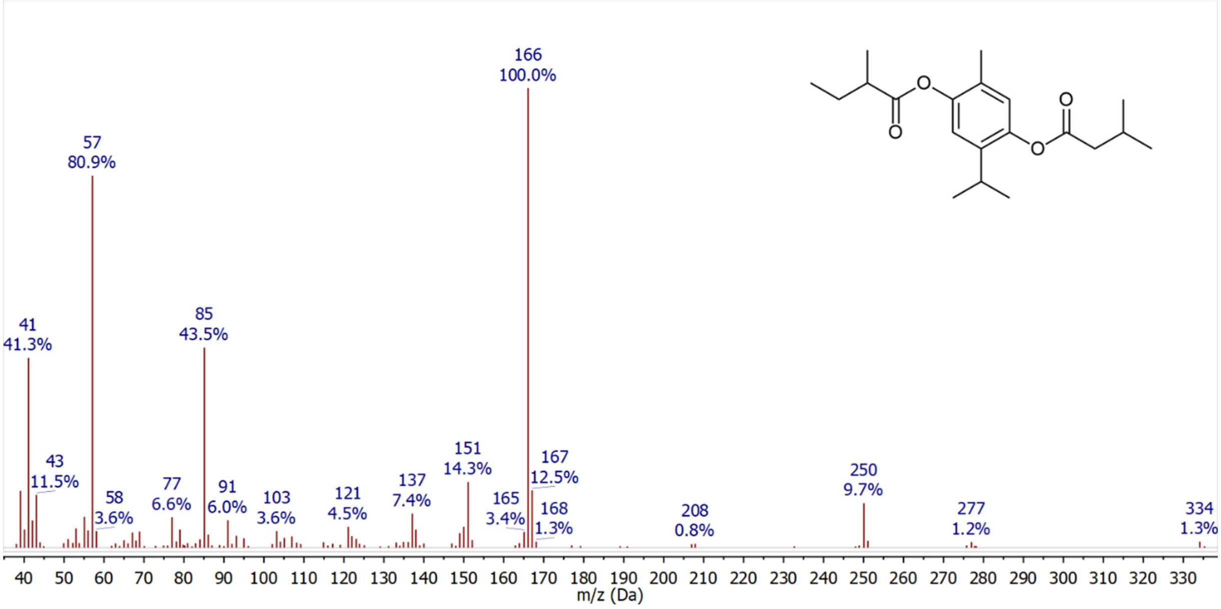

Figure S69. Mass spectrum (EI, 70 eV) of 6-(2-methylbutyryloxy)thymyl isovalerate.

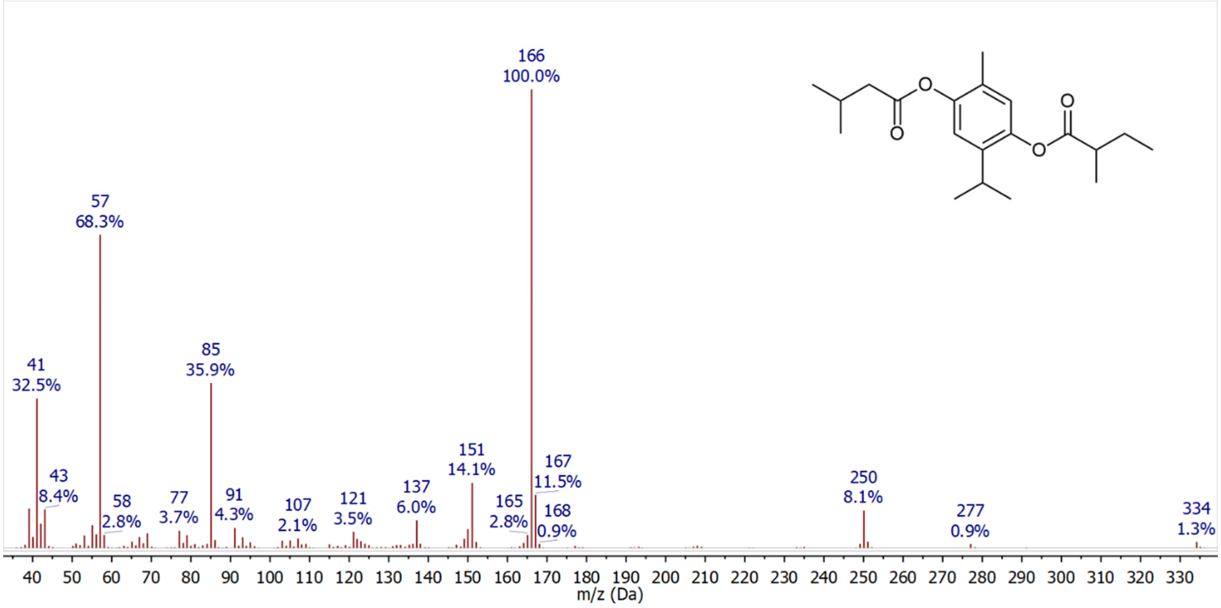

Figure S70. Mass spectrum (EI, 70 eV) of 6-isovaleroxythymyl 2-methylbutyrate.

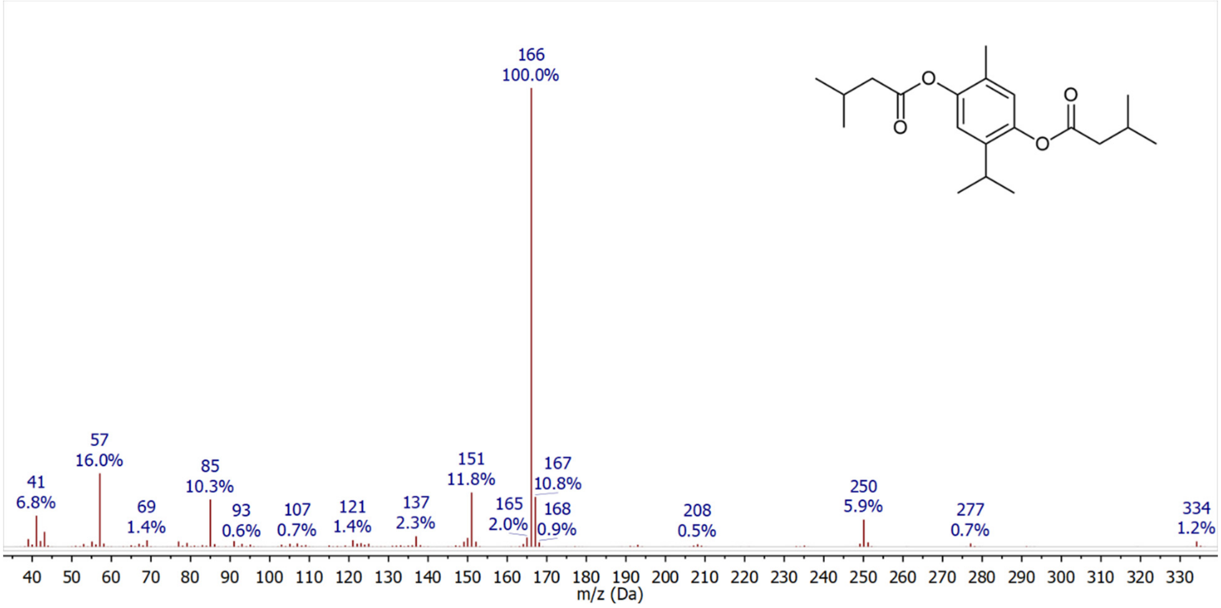

Figure S71. Mass spectrum (EI, 70 eV) of 6-isovaleroxythymyl isovalerate.

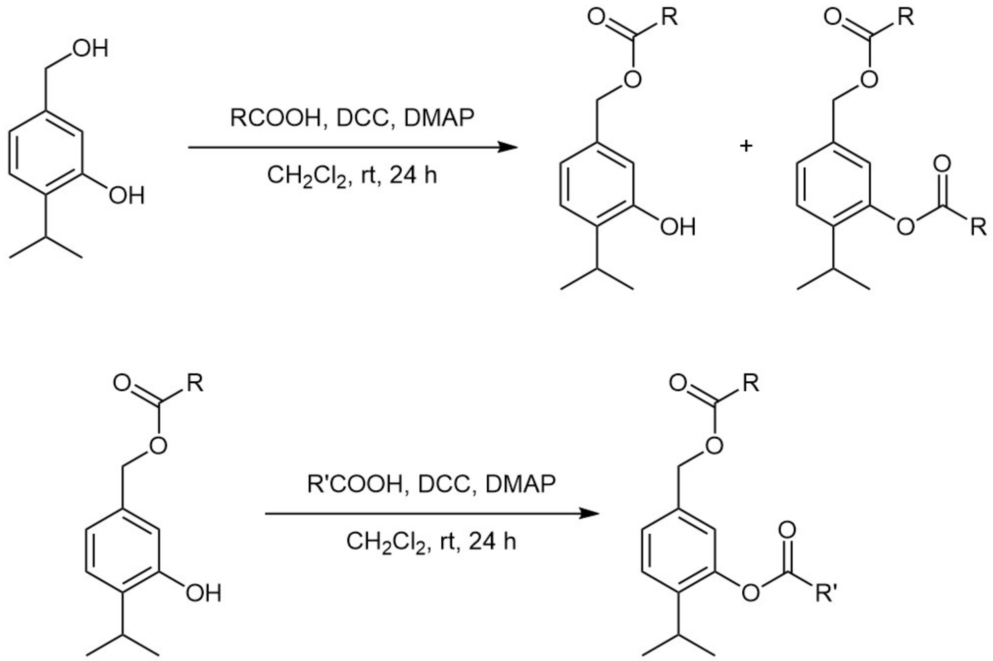

Scheme S4. Synthetic pathway to 7-hydroxythymol-derived monoesters and diesters (R,R‘ – isopropyl, *sec*-butyl, or isobutyl).

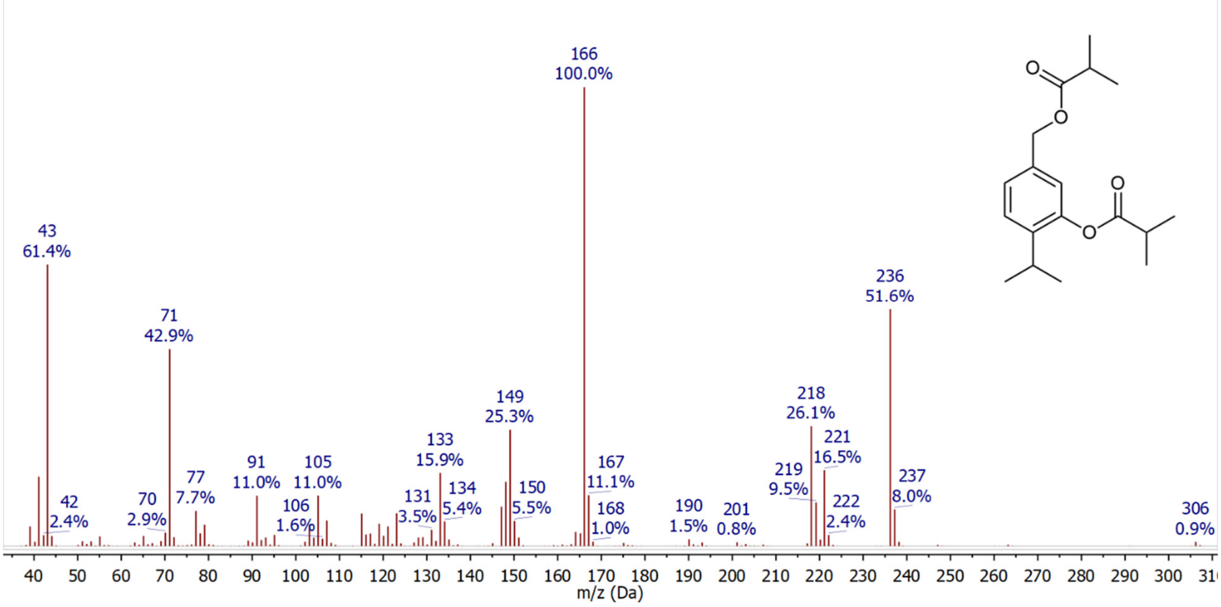

Figure S72. Mass spectrum (EI, 70 eV) of 7-isobutyryloxythymyl isobutyrate.

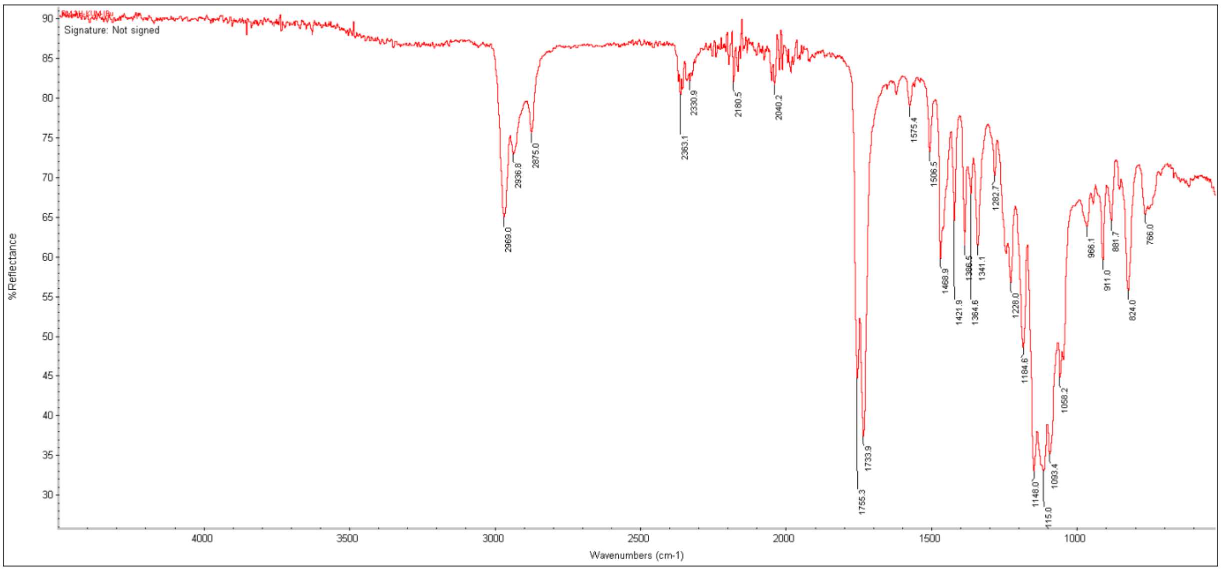

Figure S73. IR spectrum of 7-isobutyryloxythymyl isobutyrate.

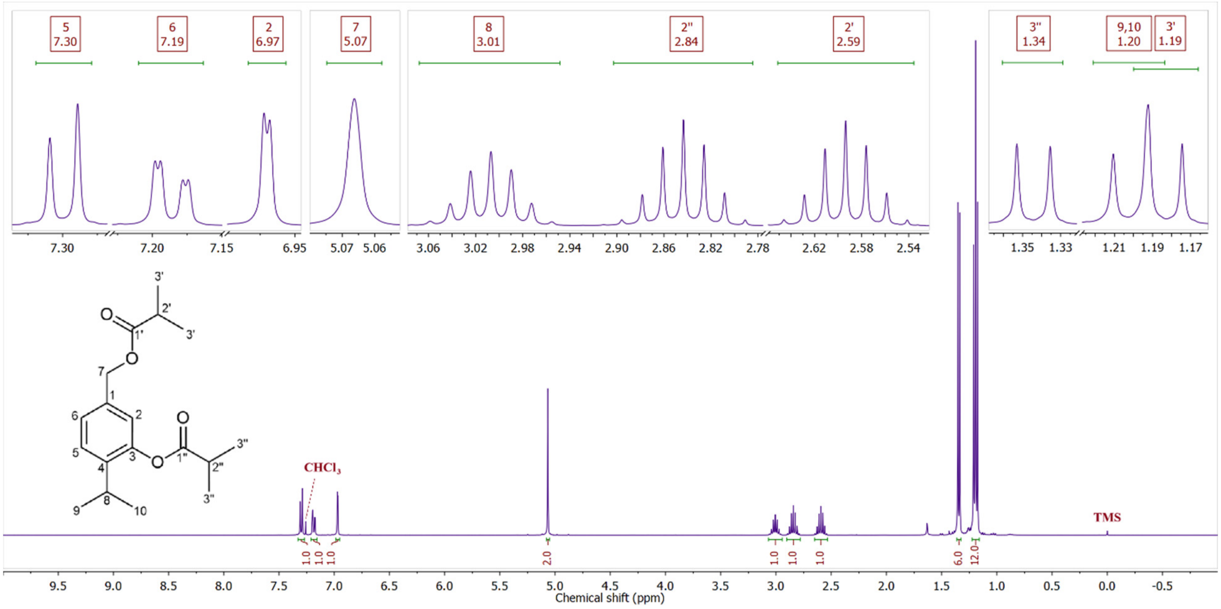

Figure S74. ^{1}H NMR (400 MHz, $CDCl_3$) spectrum of 7-isobutyryloxythymyl isobutyrate and the corresponding expansions with signal assignment.

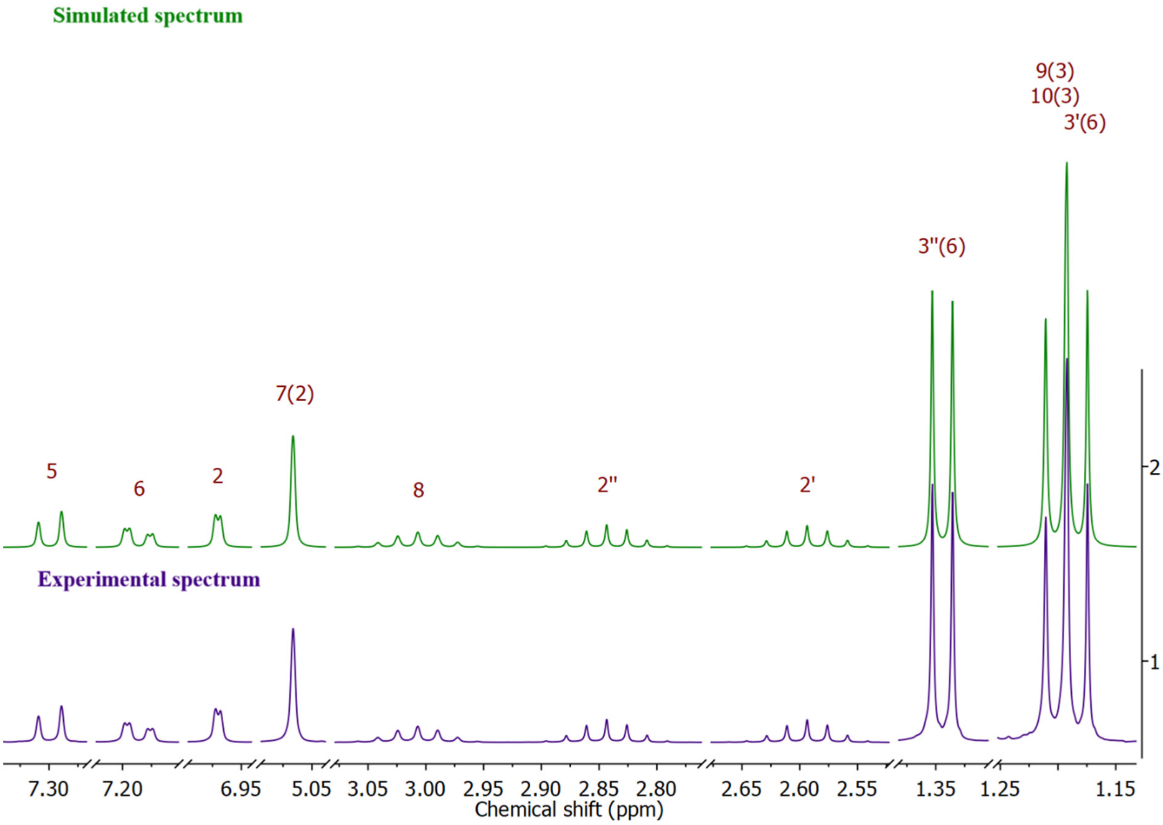

Figure S75. Simulated (manual iterative full spin, MestreNova 11.0.3) and experimental ^{1}H NMR spectrum of 7-isobutyryloxythymyl isobutyrate.

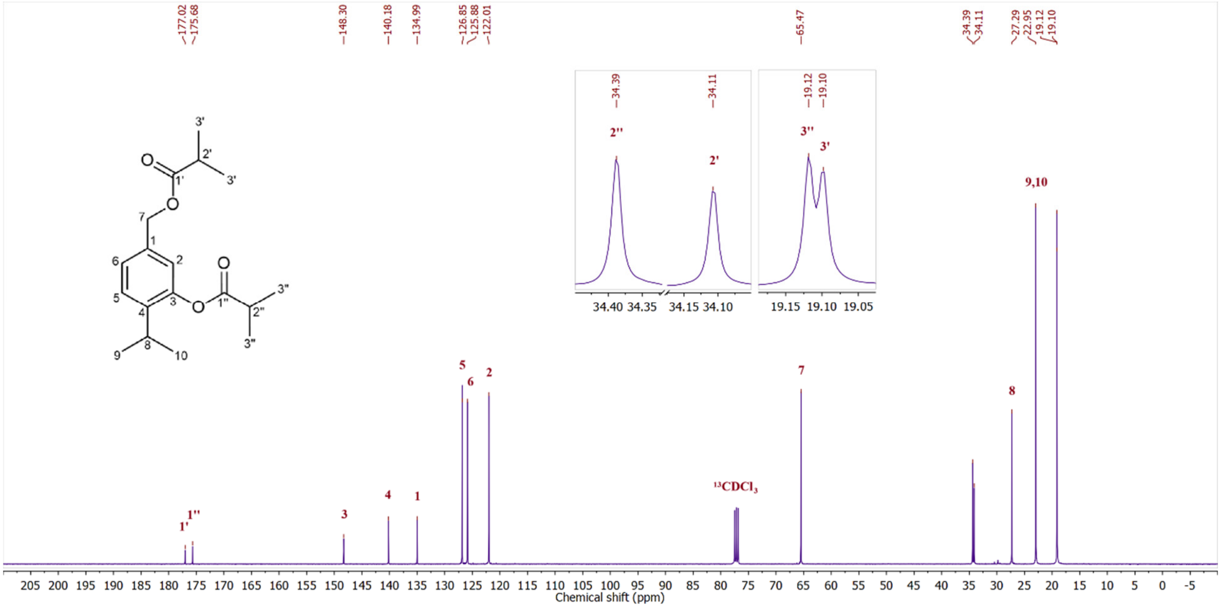

Figure S76. ^{13}C NMR (100.6 MHz, $CDCl_3$) spectrum of 7-isobutyryloxythymyl isobutyrate.

Table S13. ^{1}H (400 MHz) and ^{13}C (100.6 MHz) NMR data of 7-isobutyryloxythymyl isobutyrate (chloroform-*d*); NMR parameters are derived from manual iterative full spin analysis, along with the observed gHMBC and NOESY correlations.

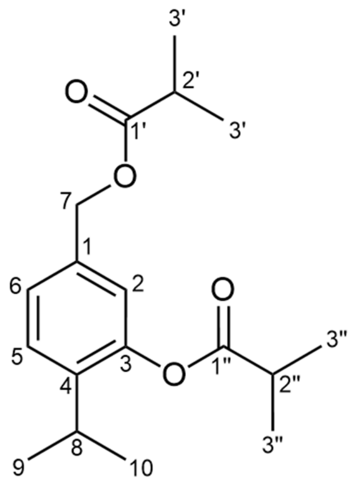

Position	δ_H (m, J (Hz), Integration)	δ_C	HMBC[a]	NOESY[b]
1	/	134.99	/	/
2	6.9703 (dtd, $^{4}J_{2,6}$ = 1.8, $^{4}J_{2,7}$ = -0.5, $^{5}J_{2,8}$ = 0.3, 1 H)	122.01	3,4,6,7	7
3	/	148.30	/	/
4	/	140.18	/	/
5	7.2983 (ddt, $^{3}J_{5,6}$ = 8.0, $^{4}J_{5,8}$ = -0.6, $^{5}J_{5,7}$ = 0.3, 1 H)	126.85	1,3,8	6,8,9,10
6	7.1870 (ddtd, $^{3}J_{5,6}$ = 8.0, $^{4}J_{2,6}$ = 1.8, $^{4}J_{6,7}$ = -0.6, $^{5}J_{6,8}$ = 0.4, 1 H)	125.88	2,4,7	5,7
7	5.0663 (ddd, $^{4}J_{6,7}$ = -0.6, $^{4}J_{2,7}$ = -0.5, $^{5}J_{5,7}$ = 0.3, 2 H)	65.47	1,2,6,1′	2,6
8	3.0067 (septddd, $^{3}J_{8,9/10}$ = 6.9, $^{4}J_{5,8}$ = -0.6, $^{5}J_{6,8}$ = 0.4, $^{5}J_{2,8}$ = 0.3, 1 H)	27.29	3,4,5,9,10	5,9,10
9 and 10	1.2021 (d, $^{3}J_{8,9/10}$ = 6.9, 6 H)	22.95	4,8,9,10	5,8,9,10
1′	/	177.02	/	/
2′	2.5934 (sept, $^{3}J_{2',3'}$ = 7.0, 1 H)	34.11	1′,3′	3′
3′	1.1833 (d, $^{3}J_{2',3'}$ = 7.0, 6 H)	19.10[c]	1′,2′,3′	2′
1′′	/	175.68	/	/
2′′	2.8432 (sept, $^{3}J_{2'',3''}$ = 7.0, 1 H)	34.39	1′′,3′′	3′′
3′′	1.3443 (d, $^{3}J_{2'',3''}$ = 7.0, 6 H)	19.12[c]	1′′,2′′,3′′	2′′

[a] gHMBC correlations observed between the hydrogen in this row and the carbon in the listed position.

[b] Cross-peaks observed in the NOESY spectrum.

[c] The signals of C-3′ and C-3′′ may be interchanged.

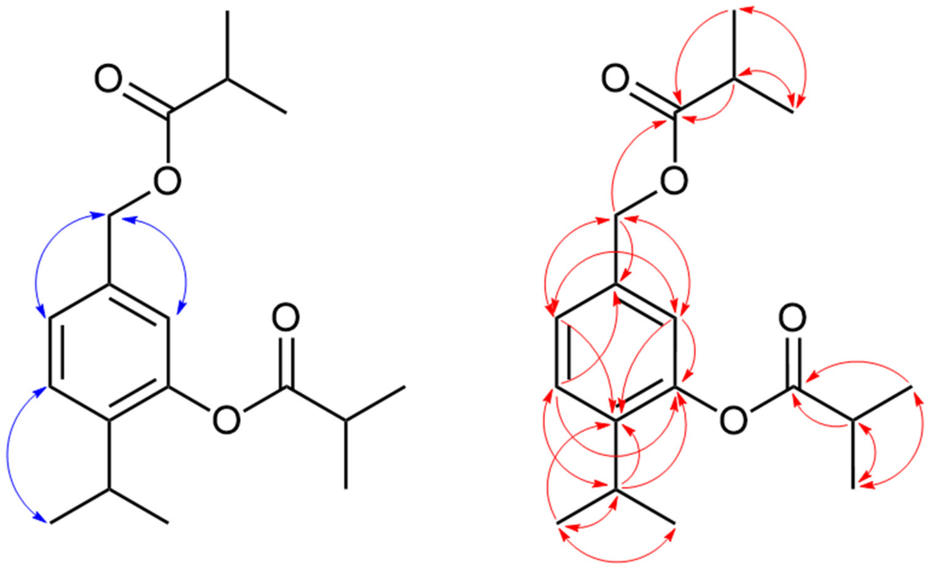

Figure S77. NOESY (blue arrows) and HMBC (red arrows) interactions of 7-isobutyryloxythymyl isobutyrate.

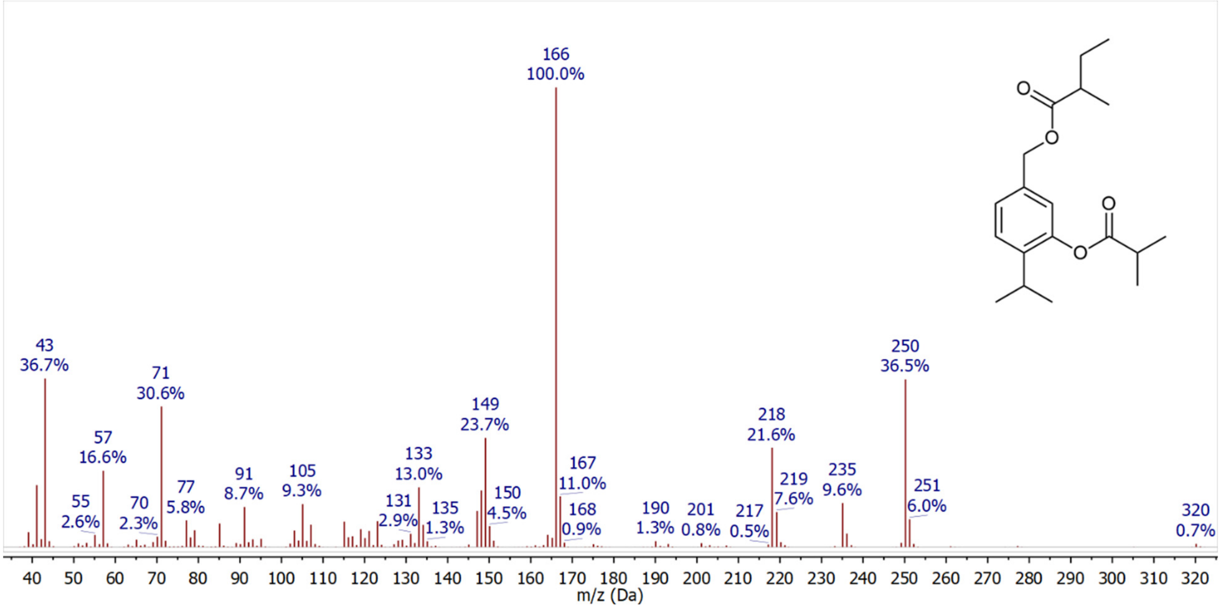

Figure S78. Mass spectrum (EI, 70 eV) of 7-(2-methylbutyryloxy)thymyl isobutyrate.

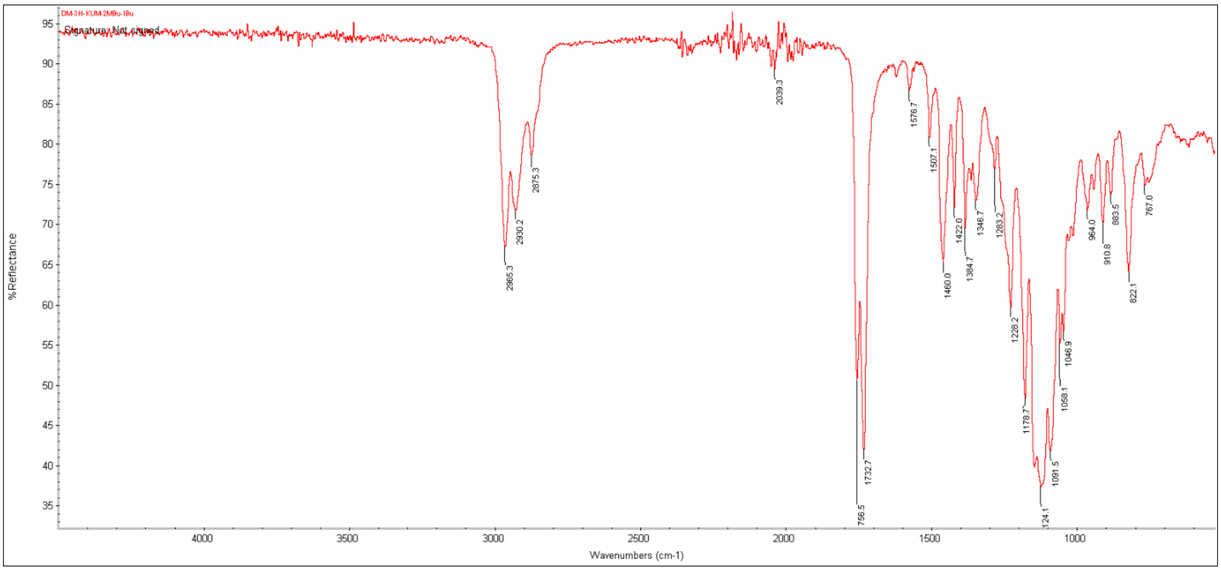

Figure S79. IR spectrum of 7-(2-methylbutyryloxy)thymyl isobutyrate.

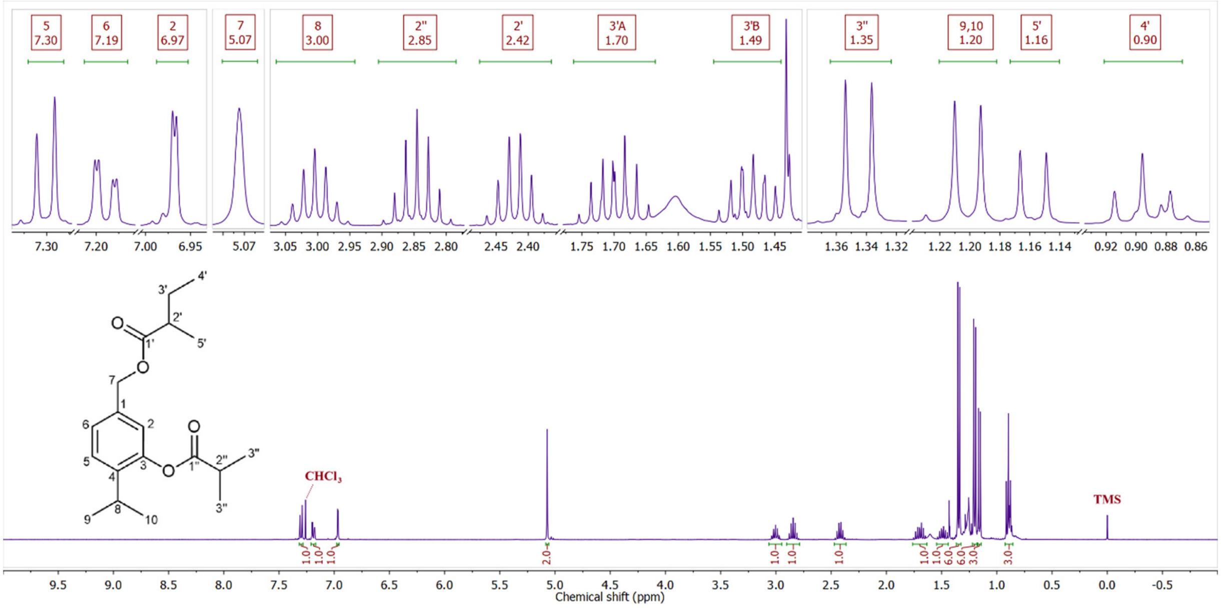

Figure S80. ^{1}H NMR (400 MHz, $CDCl_3$) spectrum of 7-(2-methylbutyryloxy)thymyl isobutyrate and the corresponding expansions with signal assignment.

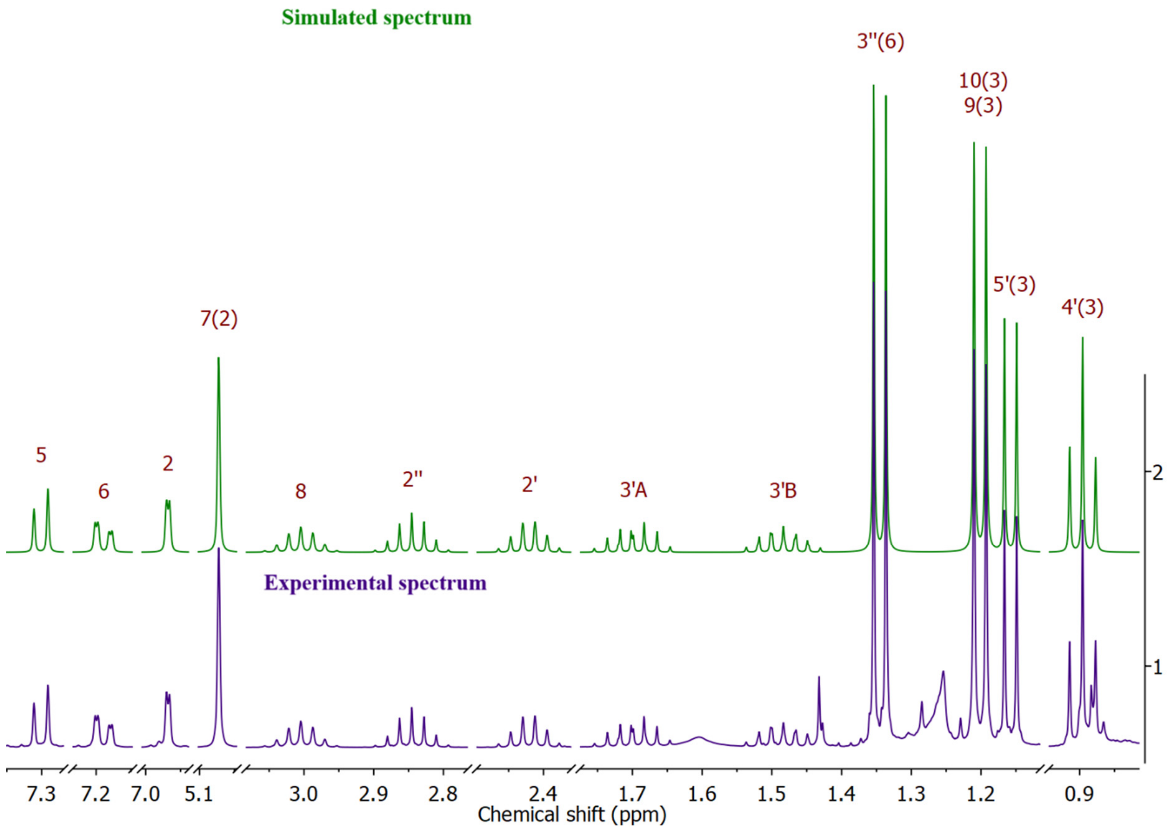

Figure S81. Simulated (manual iterative full spin, MestreNova 11.0.3) and experimental ^{1}H NMR spectrum of 7-(2-methylbutyryloxy)thymyl isobutyrate.

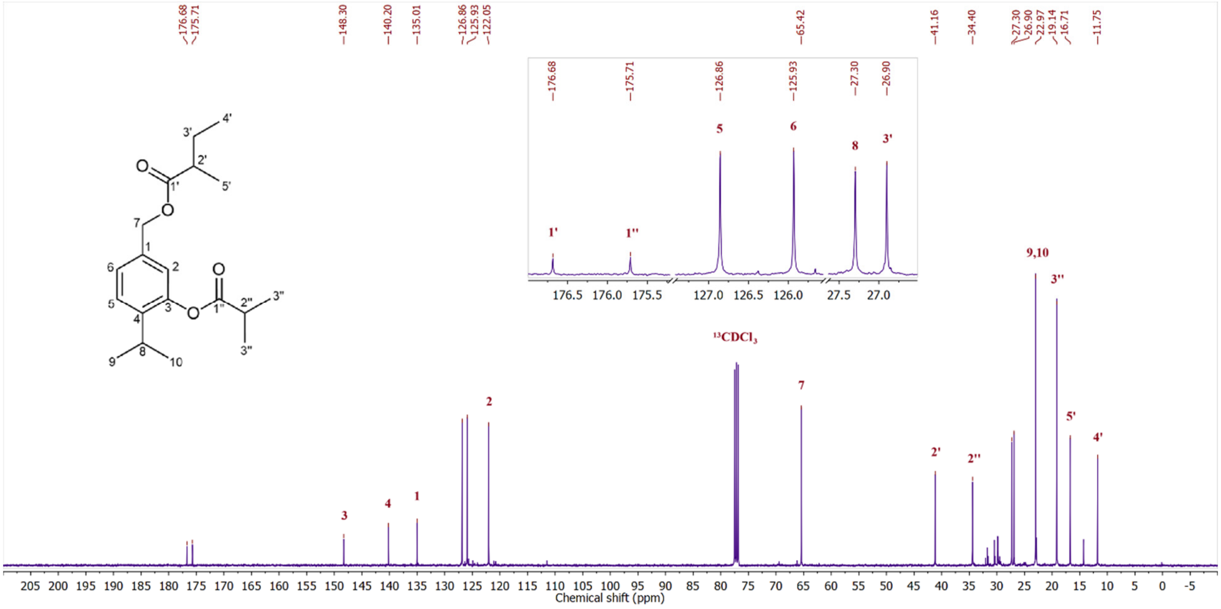

Figure S82. ^{13}C NMR (100.6 MHz, CDCl$_3$) spectrum of 7-(2-methylbutyryloxy)thymyl isobutyrate.

Table S14. ^{1}H (400 MHz) and ^{13}C (100.6 MHz) NMR data of 7-(2-methylbutyryloxy)thymyl isobutyrate (chloroform-*d*); NMR parameters are derived from manual iterative full spin analysis, along with the observed gHMBC and NOESY correlations.

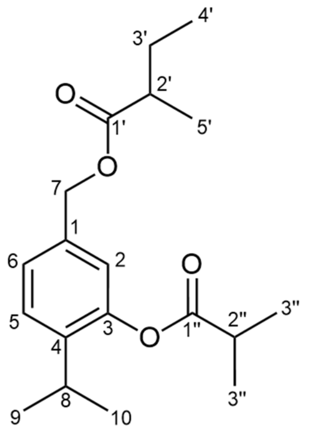

Position	δ_H (m, J (Hz), Integration)	δ_C	HMBC[a]	NOESY[b]
1	/	135.01	/	/
2	6.9679 (dtd, $^{4}J_{2,6}$ = 1.8, $^{4}J_{2,7}$ = -0.6, $^{5}J_{2,8}$ = 0.3, 1 H)	122.05	3,4,6,7	7
3	/	148.30	/	/
4	/	140.20	/	/
5	7.3001 (ddt, $^{3}J_{5,6}$ = 8.0, $^{4}J_{5,8}$ = -0.6, $^{5}J_{5,7}$ = 0.3, 1 H)	126.86	1,3,8	6,9,10
6	7.1903 (ddtd, $^{3}J_{5,6}$ = 8.0, $^{4}J_{2,6}$ = 1.8, $^{4}J_{6,7}$ = -0.6, $^{5}J_{6,8}$ = 0.4, 1 H)	125.93	2,4,7	5,7
7	5.0725 (ddd, $^{4}J_{2,7}$ = -0.6, $^{4}J_{6,7}$ = -0.6, $^{5}J_{5,7}$ = 0.3, 2 H)	65.42	1,2,6,1′	2,6
8	3.0043 (septddd, $^{3}J_{8,9/10}$ = 6.9, $^{4}J_{5,8}$ = -0.6, $^{5}J_{6,8}$ = 0.4, $^{5}J_{2,8}$ = 0.3, 1 H)	27.30	3,4,5,9,10	9,10
9 and 10	1.2014 (d, $^{3}J_{8,9/10}$ = 6.9, 6 H)	22.97	4,8,9,10	5,8,9,10
1′	/	176.68	/	/
2′	2.4205 (dqdq, $^{3}J_{2',3'A}$ = 7.5, $^{3}J_{2',5'}$ = 7.0, $^{3}J_{2',3'B}$ = 6.5, $^{4}J_{2',4'}$ = 0.3, 1 H)	41.16	1′,3′,4′,5′	3′,4′,5′
3′A	1.6987 (ddq, $^{2}J_{3'A,3'B}$ = -13.7, $^{3}J_{2',3'A}$ = 7.5, $^{3}J_{3'A,4'}$ = 7.5, 1 H)	26.90	1′,2′,4′,5′	2′,4′,5′
3′B	1.4846 (dqd, $^{2}J_{3'A,3'B}$ = -13.7, $^{3}J_{3'B,4'}$ = 7.5, $^{3}J_{2',3'B}$ = 6.5, 1 H)	26.90	1′,2′,4′,5′	2′,4′,5′
4′	0.8960 (ddd, $^{3}J_{3'A,4'}$ = 7.5, $^{3}J_{3'B,4'}$ = 7.5, $^{4}J_{2',4'}$ = 0.3, 3 H)	11.75	2′,3′	2′,3′
5′	1.1577 (d, $^{3}J_{2',5'}$ = 7.0, 3 H)	16.71	1′,2′,3′	2′,3′
1′′	/	175.71	/	/
2′′	2.8451 (sept, $^{3}J_{2'',3''}$ = 7.0, 1 H)	34.40	1′′,3′′	3′′
3′′	1.3452 (d, $^{3}J_{2'',3''}$ = 7.0, 6 H)	19.14	1′′,2′′,3′′	2′′

[a] gHMBC correlations observed between the hydrogen in this row and the carbon in the listed position.

[b] Cross-peaks observed in the NOESY spectrum.

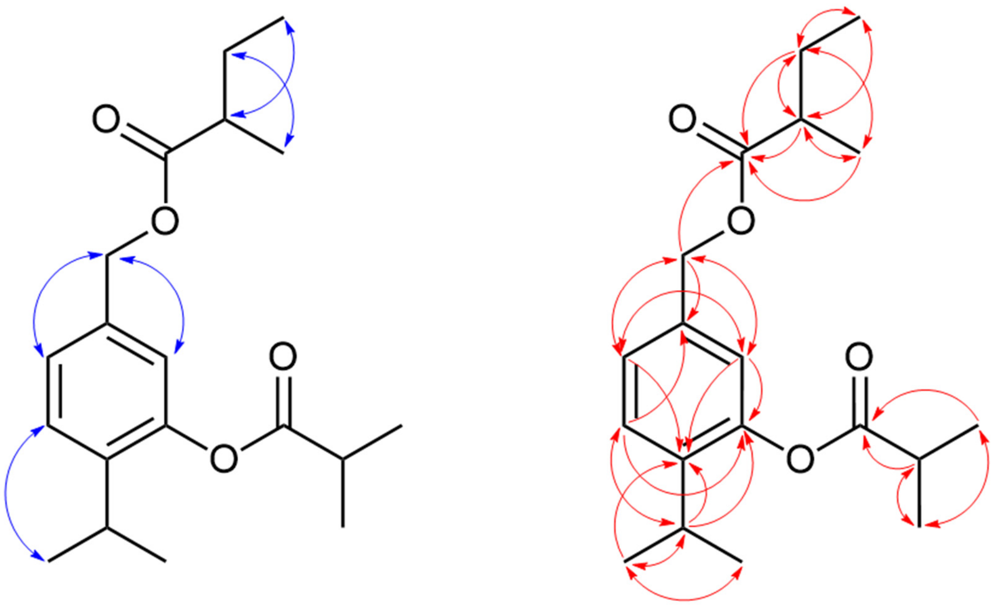

Figure S83. NOESY (blue arrows) and HMBC (red arrows) interactions of 7-(2-methylbutyryloxy)thymyl isobutyrate.

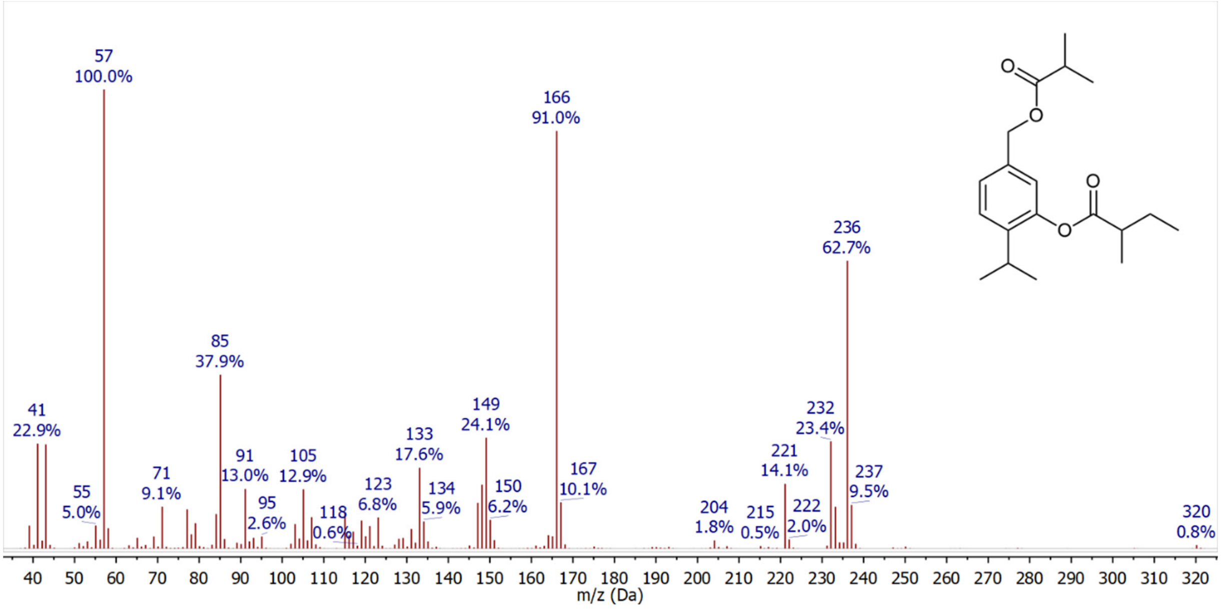

Figure S84. Mass spectrum (EI, 70 eV) of 7-isobutyryloxythymyl 2-methylbutyrate.

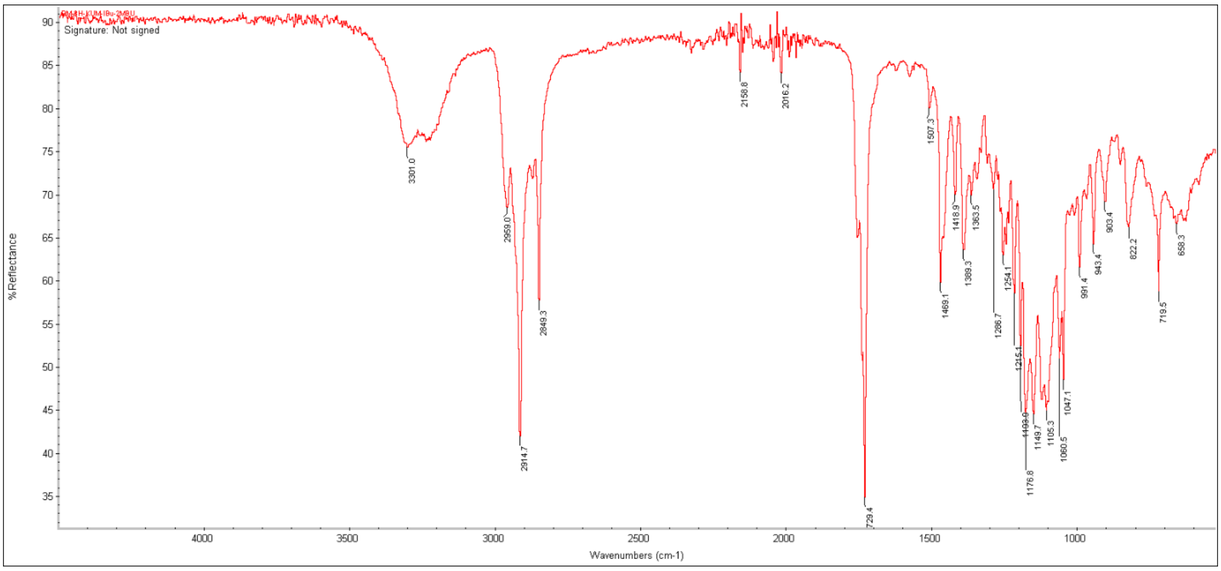

Figure S85. IR spectrum of 7-isobutyryloxythymyl 2-methylbutyrate.

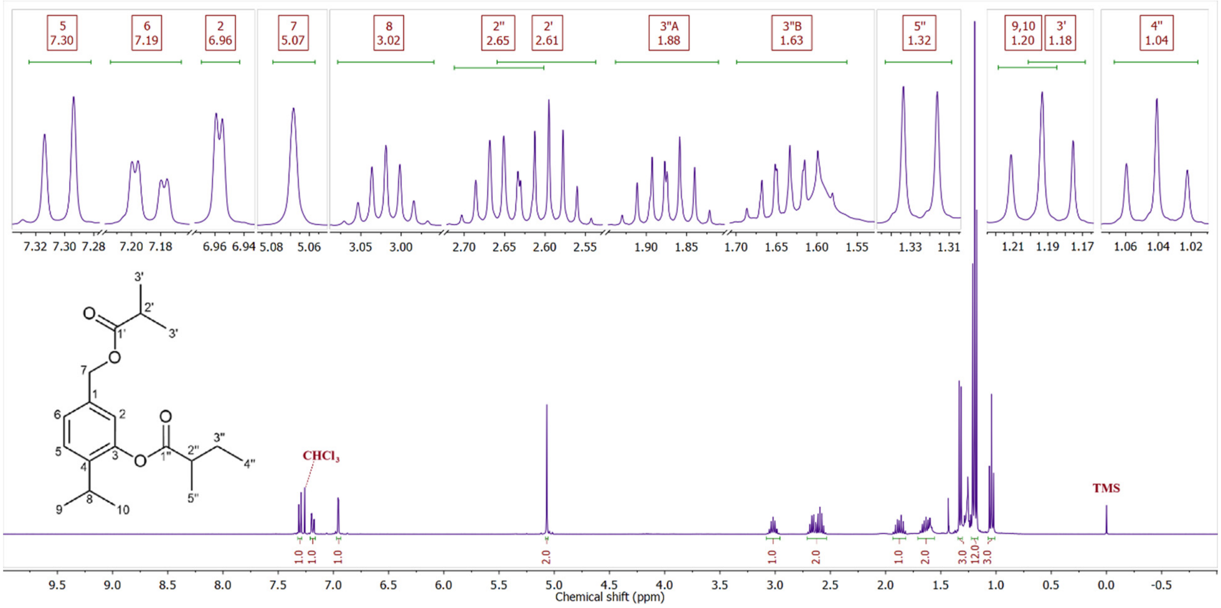

Figure S86. ^{1}H NMR (400 MHz, $CDCl_3$) spectrum of 7-isobutyryloxythymyl 2-methylbutyrate and the corresponding expansions with signal assignment.

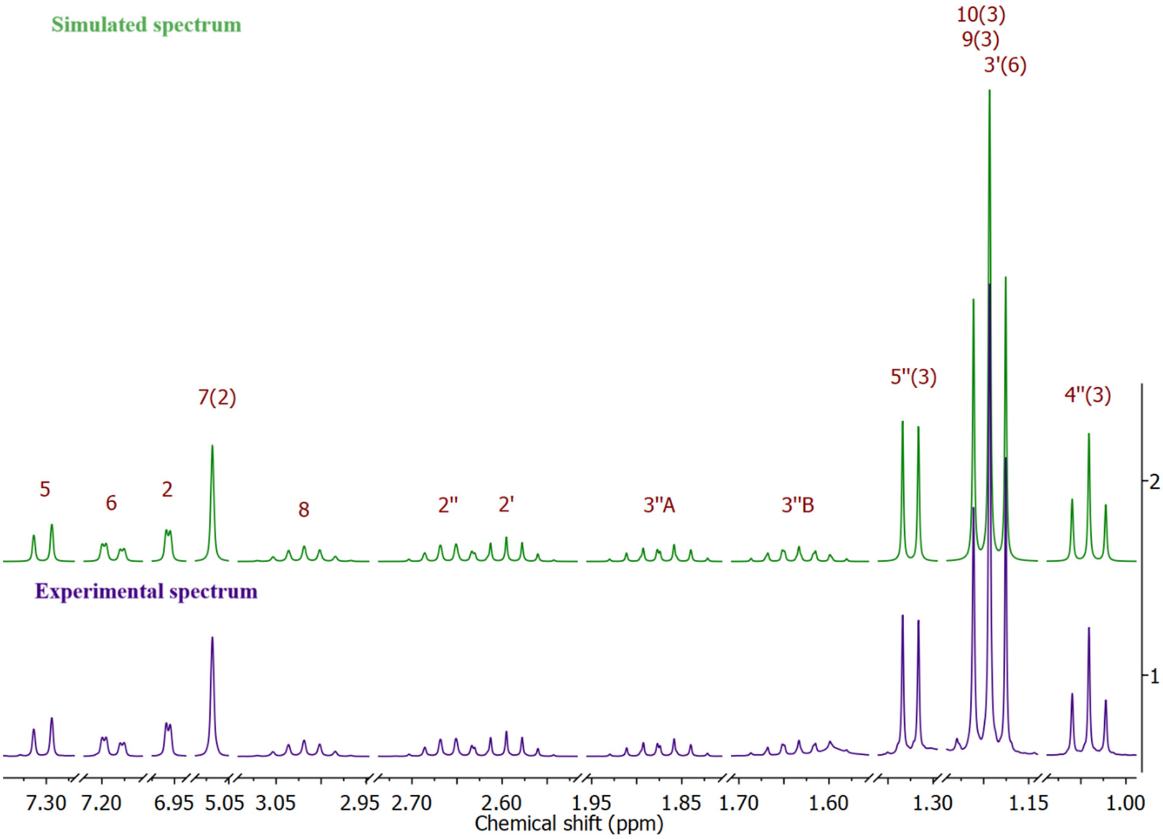

Figure S87. Simulated (manual iterative full spin, MestreNova 11.0.3) and experimental ^{1}H NMR spectrum of 7-isobutyryloxythymyl 2-methylbutyrate.

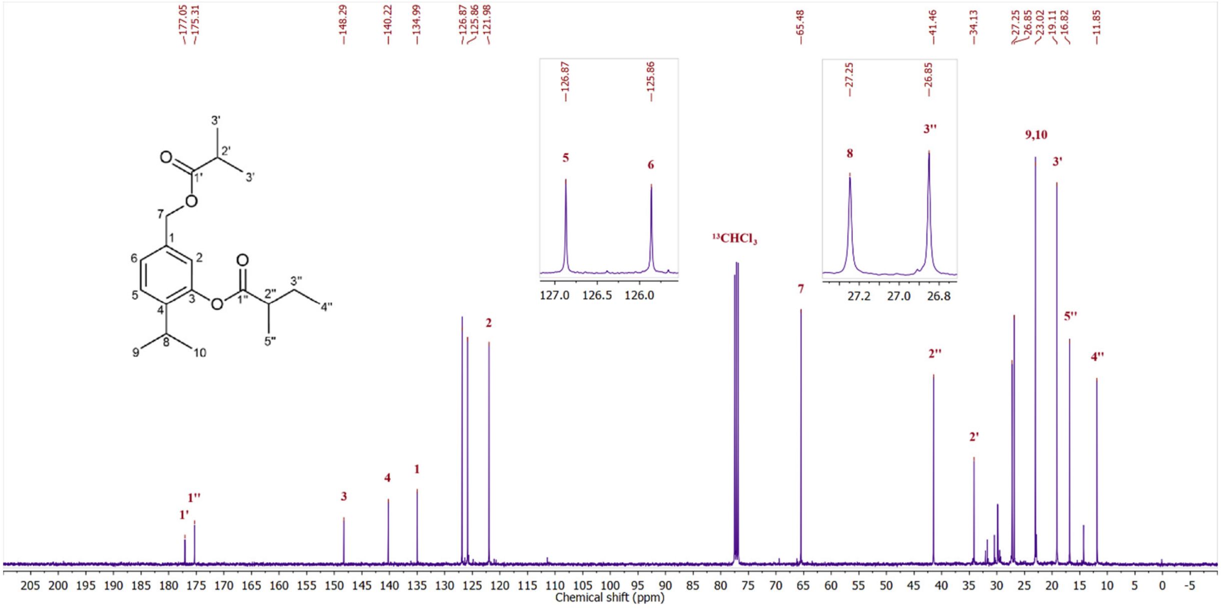

Figure S88. ^{13}C NMR (100.6 MHz, $CDCl_3$) spectrum of 7-isobutyryloxythymyl 2-methylbutyrate.

Table S15. ^{1}H (400 MHz) and ^{13}C (100.6 MHz) NMR data of 7-isobutyryloxythymyl 2-methylbutyrate (chloroform-*d*); NMR parameters are derived from manual iterative full spin analysis, along with the observed gHMBC and NOESY correlations.

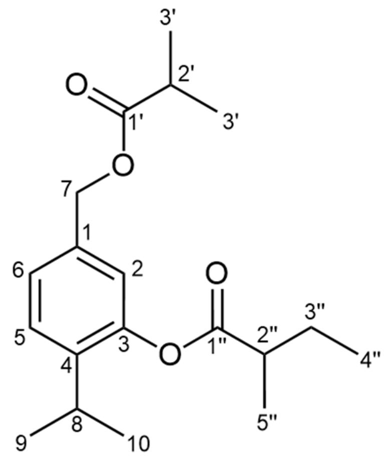

Position	δ_H (m, J (Hz), Integration)	δ_C	HMBC[a]	NOESY[b]
1	/	134.99	/	/
2	6.9570 (dtd, $^{4}J_{2,6}$ = 1.8, $^{4}J_{2,7}$ = -0.6, $^{5}J_{2,8}$ = 0.3, 1 H)	121.98	3,4,6,7	7
3	/	148.29	/	/
4	/	140.22	/	/
5	7.3030 (ddt, $^{3}J_{5,6}$ = 8.0, $^{4}J_{5,8}$ = -0.6, $^{5}J_{5,7}$ = 0.3, 1 H)	126.87	1,3,8	6,9,10
6	7.1884 (ddtd, $^{3}J_{5,6}$ = 8.0, $^{4}J_{2,6}$ = 1.8, $^{4}J_{6,7}$ = -0.6, $^{5}J_{6,8}$ = 0.4, 1 H)	125.86	2,4,7	5,7
7	5.0683 (ddd, $^{4}J_{2,7}$ = -0.6, $^{4}J_{6,7}$ = -0.6, $^{5}J_{5,7}$ = 0.3, 2 H)	65.48	1,2,6,1′	2,6
8	3.0186 (septddd, $^{3}J_{8,9/10}$ = 6.9, $^{4}J_{5,8}$ = -0.5, $^{5}J_{6,8}$ = 0.4, $^{5}J_{2,8}$ = 0.3, 1 H)	27.25	3,4,5,9,10	9,10
9 and 10	1.2026 (d, $^{3}J_{8,9/10}$ = 6.9, 6 H)	23.02	4,8,9,10	5,8,9,10
1′	/	177.05	/	/
2′	2.5949 (sept, $^{3}J_{2′,3′}$ = 7.0, 1 H)	34.13	1′,3′	3′
3′	1.1843 (d, $^{3}J_{2′,3′}$ = 7.0, 6 H)	19.11	1′,2′,3′	2′
1′	/	175.31	/	/
1′′	2.6594 (dqdq, $^{3}J_{2′′,3′′A}$ = 7.5, $^{3}J_{2′′,5′′}$ = 7.0, $^{3}J_{2′′,3′′B}$ = 6.5, $^{4}J_{2′′,4′′}$ = 0.3, 1 H)	41.46	1′′,3′′,4′′,5′′	3′′,4′′,5′′
2′′	1.8743 (ddq, $^{2}J_{3′′A,3′′B}$ = -13.7, $^{3}J_{2′′,3′′A}$ = 7.5, $^{3}J_{3′′A,4′′}$ = 7.5, 1 H)	26.85	1′′,2′′,4′′,5′′	2′′,4′′,5′′
3′′A	1.6343 (dqd, $^{2}J_{3′′A,3′′B}$ = -13.7, $^{3}J_{3′′B,4′′}$ = 7.5, $^{3}J_{2′′,3′′B}$ = 6.5, 1 H)	26.85	1′′,2′′,4′′,5′′	2′′,4′′,5′′
3′′B	1.0412 (ddd, $^{3}J_{3′′A,4′′}$ = 7.5, $^{3}J_{3′′B,4′′}$ = 7.5, $^{4}J_{2′′,4′′}$ = 0.3, 3 H)	11.85	2′′,3′′	2′′.3′′
4′′	1.3250 (d, $^{3}J_{2′′,5′′}$ = 7.0, 3 H)	16.82	1′′,2′′,3′′	2′′,3′′

[a] gHMBC correlations observed between the hydrogen in this row and the carbon in the listed position.

[b] Cross-peaks observed in the NOESY spectrum.

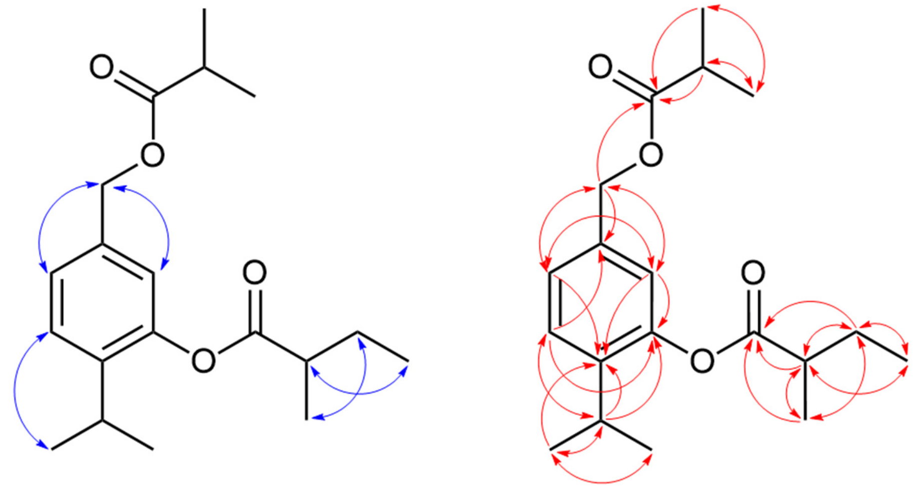

Figure S89. NOESY (blue arrows) and HMBC (red arrows) interactions of 7-isobutyryloxythymyl 2-methylbutyrate.

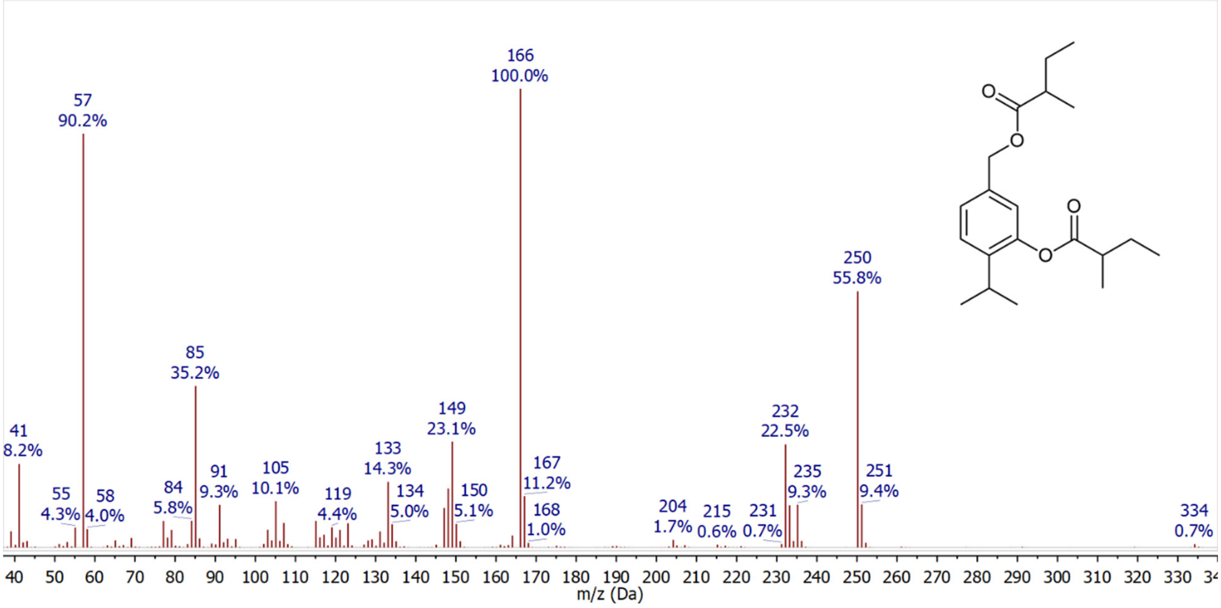

Figure S90. Mass spectrum (EI, 70 eV) of 7-(2-methylbutyryloxy)thymyl 2-methylbutyrate.

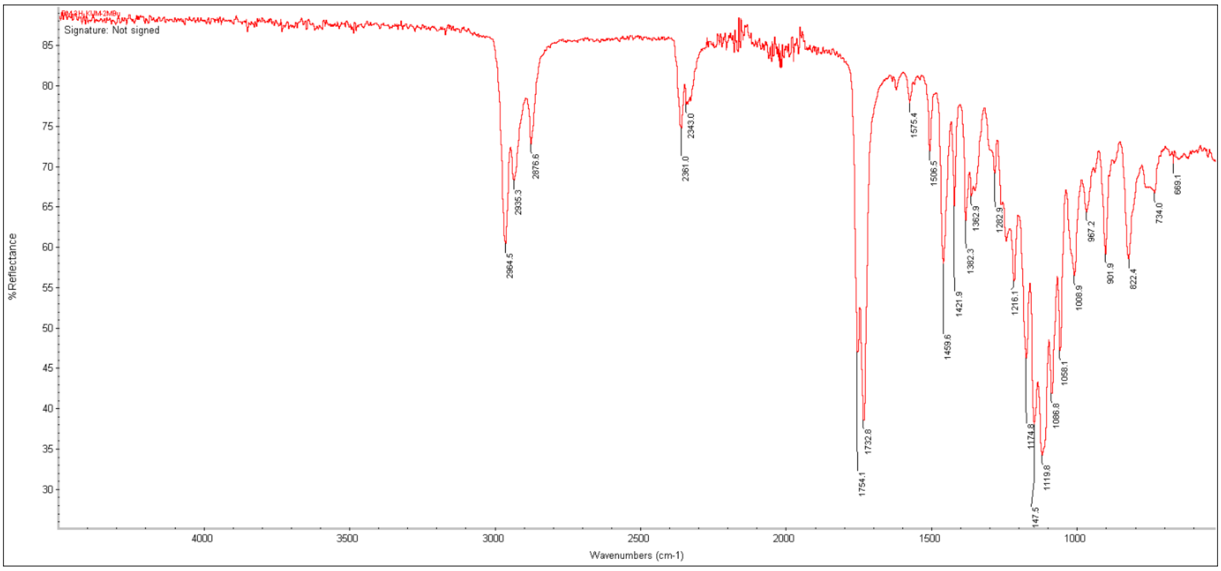

Figure S91. IR spectrum of 7-(2-methylbutyryloxy)thymyl 2-methylbutyrate.

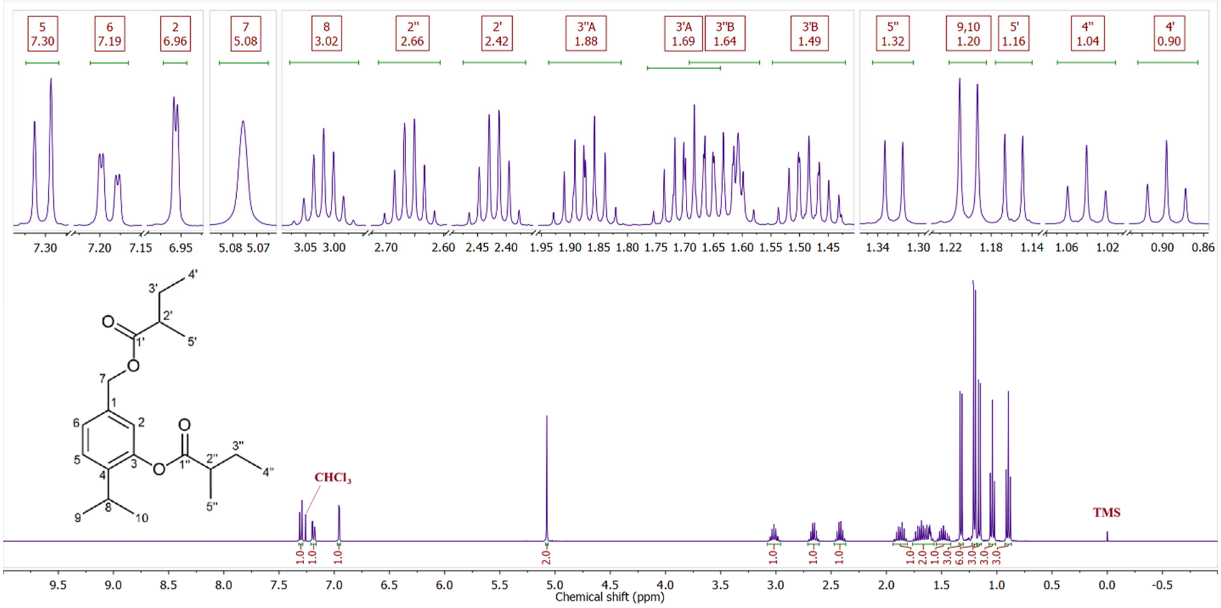

Figure S92. ^{1}H NMR (400 MHz, $CDCl_3$) spectrum of 7-(2-methylbutyryloxy)thymyl 2-methylbutyrate and the corresponding expansions with signal assignment.

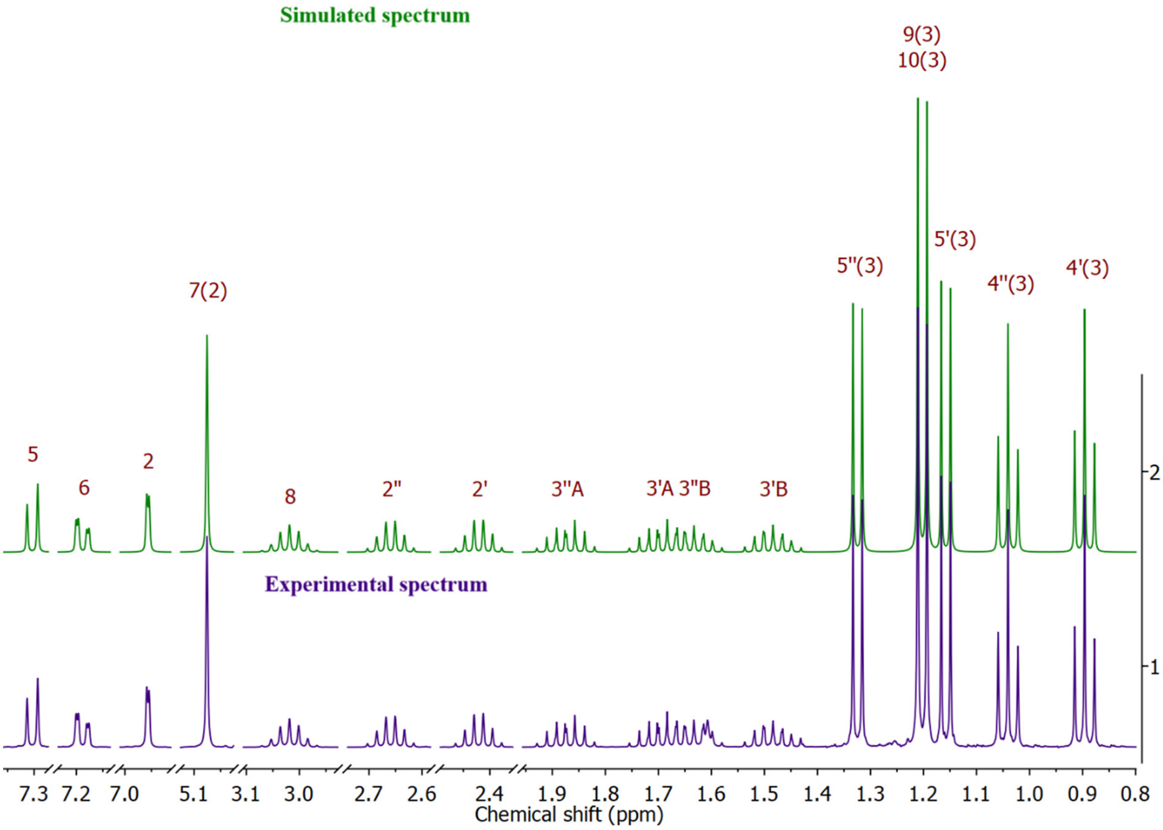

Figure S93. Simulated (manual iterative full spin, MestreNova 11.0.3) and experimental ^{1}H NMR spectrum of 7-(2-methylbutyryloxy)thymyl 2-methylbutyrate.

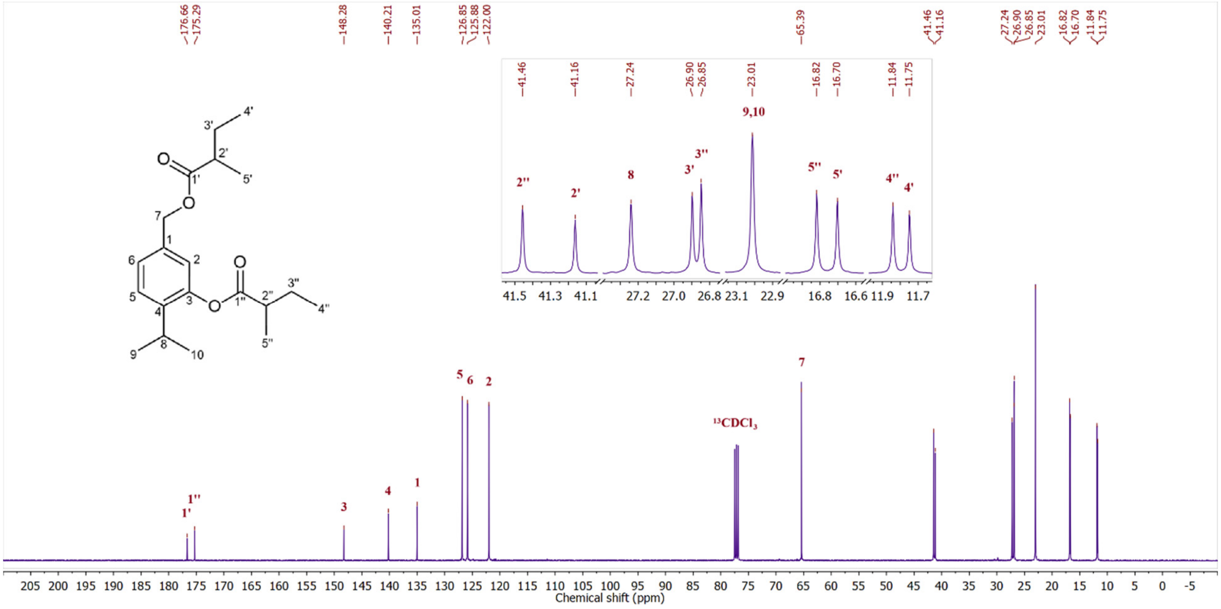

Figure S94. ^{13}C NMR (100.6 MHz, $CDCl_3$) spectrum of 7-(2-methylbutyryloxy)thymyl 2-methylbutyrate.

Table S16. ^{1}H (400 MHz) and ^{13}C (100.6 MHz) NMR data of 7-(2-methylbutyryloxy)thymyl 2-methylbutyrate (chloroform-*d*); NMR parameters are derived from manual iterative full spin analysis, along with the observed gHMBC and NOESY correlations.

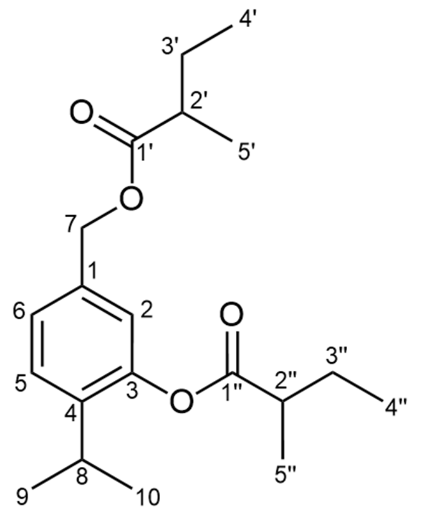

Position	δ_H (m, *J* (Hz), Integration)	δ_C	HMBC[a]	NOESY[b]
1	/	135.01	/	/
2	6.9566 (dtd, ${}^{4}J_{2,6} = 1.8$, ${}^{4}J_{2,7} = -0.6$, ${}^{5}J_{2,8} = 0.3$, 1 H)	122.00	3,4,6,7	7
3	/	148.28	/	/
4	/	140.21	/	/
5	7.3024 (ddt, ${}^{3}J_{5,6} = 8.0$, ${}^{4}J_{5,8} = -0.6$, ${}^{5}J_{5,7} = 0.3$, 1 H)	126.85	1,3,8	6,8,9,10
6	7.1891 (ddtd, ${}^{3}J_{5,6} = 8.0$, ${}^{4}J_{2,6} = 1.8$, ${}^{4}J_{6,7} = -0.6$, ${}^{5}J_{6,8} = 0.4$, 1 H)	125.88	2,4,7	5,7
7	5.0756 (ddd, ${}^{4}J_{2,7} = -0.6$, ${}^{4}J_{6,7} = -0.6$, ${}^{5}J_{5,7} = 0.3$, 2 H)	65.39	1,2,6,1′	2,6
8	3.0184 (septddd, ${}^{3}J_{8,9/10} = 6.9$, ${}^{4}J_{5,8} = -0.6$, ${}^{5}J_{6,8} = 0.4$, ${}^{5}J_{2,8} = 0.3$, 1 H)	27.24	3,4,5,9,10	5,9,10
9 and 10	1.2021 (d, ${}^{3}J_{8,9/10} = 6.9$, 6 H)	23.01	4,8,9,10	5,8,9,10
1′	/	176.66	/	/
2′	2.4206 (dqdq, ${}^{3}J_{2',3'A} = 7.5$, ${}^{3}J_{2',5'} = 7.0$, ${}^{3}J_{2',3'B} = 6.5$, ${}^{4}J_{2',4'} = 0.3$, 1 H)	41.16	1′,3′,4′,5′	3′,4′,5′
3′A	1.6987 (ddq, ${}^{2}J_{3'A,3'B} = -13.7$, ${}^{3}J_{2',3'A} = 7.5$, ${}^{3}J_{3'A,4'} = 7.5$, 1 H)	26.90	1′,2′,4′,5′	2′,4′,5′
3′B	1.4849 (dqd, ${}^{2}J_{3'A,3'B} = -13.7$, ${}^{3}J_{3'B,4'} = 7.5$, ${}^{3}J_{2',3'B} = 6.5$, 1 H)	26.90	1′,2′,4′,5′	2′,4′,5′
4′	0.8965 (ddd, ${}^{3}J_{3'A,4'} = 7.5$, ${}^{3}J_{3'B,4'} = 7.5$, ${}^{4}J_{2',4'} = 0.3$, 3 H)	11.75	2′,3′	2′.3′
5′	1.1579 (d, ${}^{3}J_{2',5'} = 7.0$, 3 H)	16.70	1′,2′,3′	2′,3′
1′′	/	175.29	/	/
2′′	2.6590 (dqdq, ${}^{3}J_{2'',3''A} = 7.5$, ${}^{3}J_{2'',5''} = 7.0$, ${}^{3}J_{2'',3''B} = 6.5$, ${}^{4}J_{2'',4''} = 0.3$, 1 H)	41.46	1′′,3′′,4′′,5′′	3′′,4′′,5′′
3′′A	1.8732 (ddq, ${}^{2}J_{3''A,3''B} = -13.7$, ${}^{3}J_{2'',3''A} = 7.5$, ${}^{3}J_{3''A,4''} = 7.5$, 1 H)	26.85	1′′,2′′,4′′,5′′	2′′,4′′,5′′
3′′B	1.6338 (dqd, ${}^{2}J_{3''A,3''B} = -13.7$, ${}^{3}J_{3''B,4''} = 7.5$, ${}^{3}J_{2'',3''B} = 6.5$, 1 H)	26.85	1′′,2′′,4′′,5′′	2′′,4′′,5′′
4′′	1.0408 (ddd, ${}^{3}J_{3''A,4''} = 7.5$, ${}^{3}J_{3''B,4''} = 7.5$, ${}^{4}J_{2'',4''} = 0.3$, 3 H)	11.84	2′′,3′′	2′′.3′′
5′′	1.3244 (d, ${}^{3}J_{2'',5''} = 7.0$, 3 H)	16.82	1′′,2′′,3′′	2′′,3′′

[a] gHMBC correlations observed between the hydrogen in this row and the carbon in the listed position.

[b] Cross-peaks observed in the NOESY spectrum.

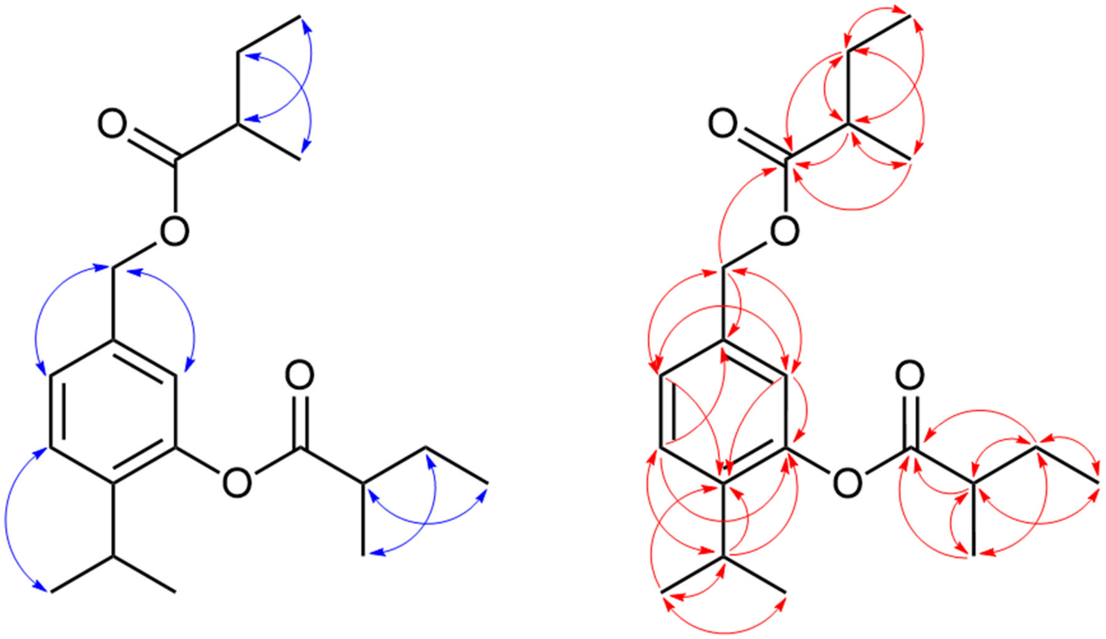

Figure S95. NOESY (blue arrows) and HMBC (red arrows) interactions of 7-(2-methylbutyryloxy)thymyl 2-methylbutyrate.

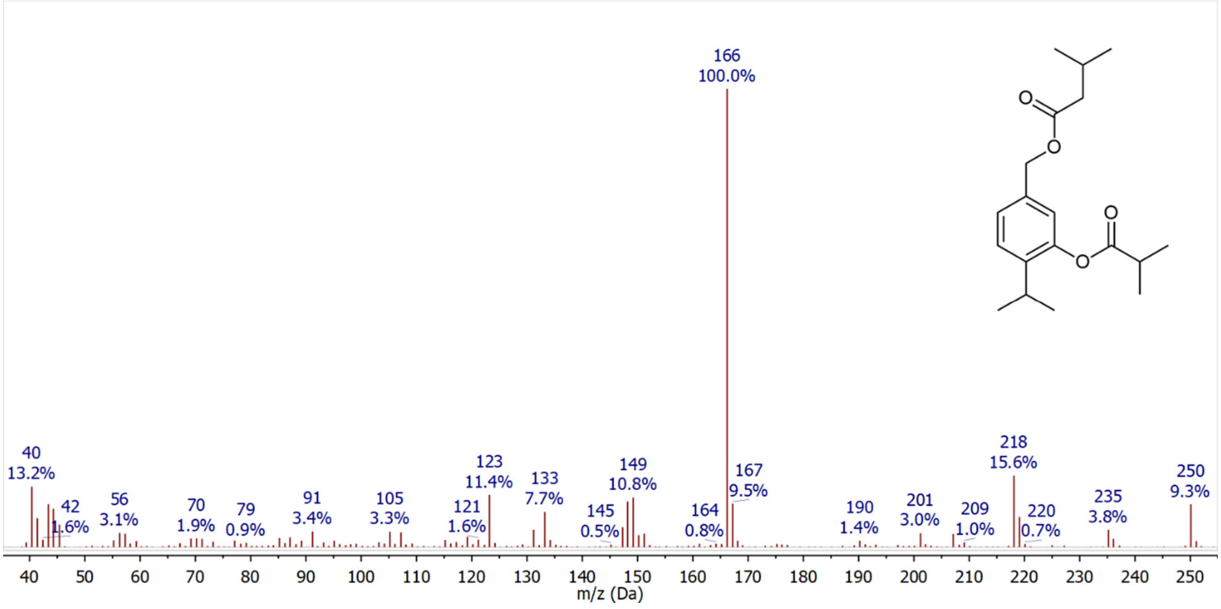

Figure S96. Mass spectrum (EI, 70 eV) of 7-isovaleroxythymyl isobutyrate.

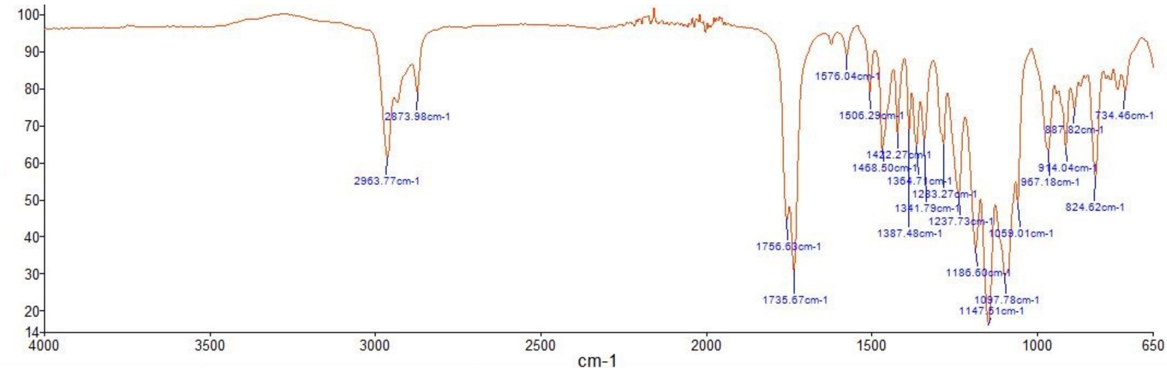

Figure S97. IR spectrum of 7-isovaleroxythymyl isobutyrate.

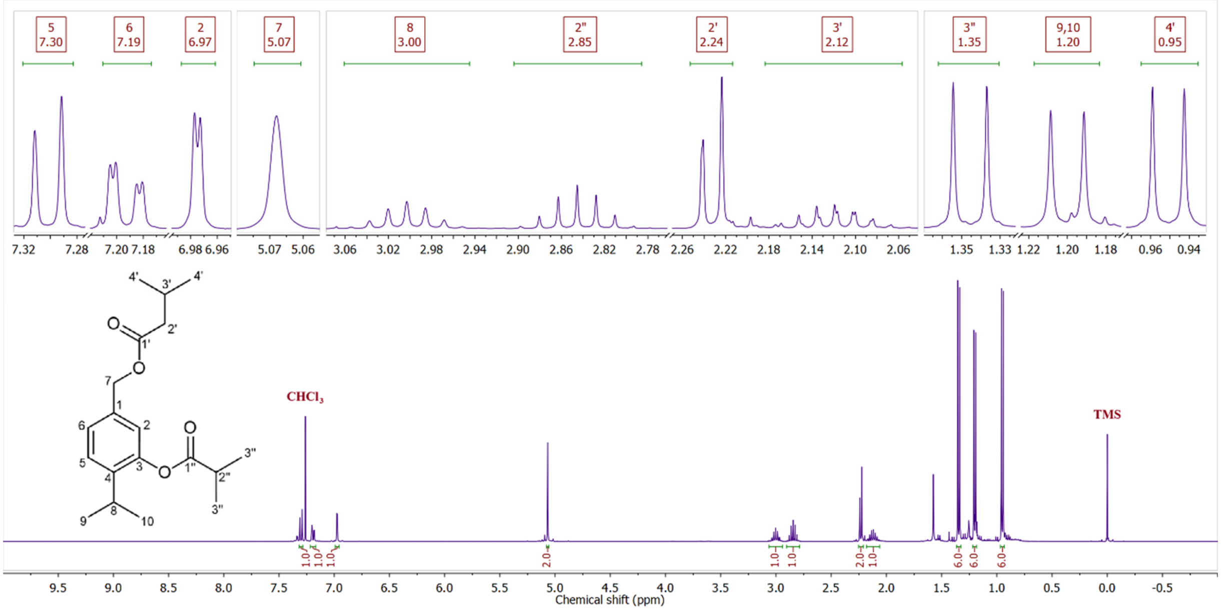

Figure S98. ^{1}H NMR (400 MHz, $CDCl_3$) spectrum of 7-isovaleroxythymyl isobutyrate and the corresponding expansions with signal assignment.

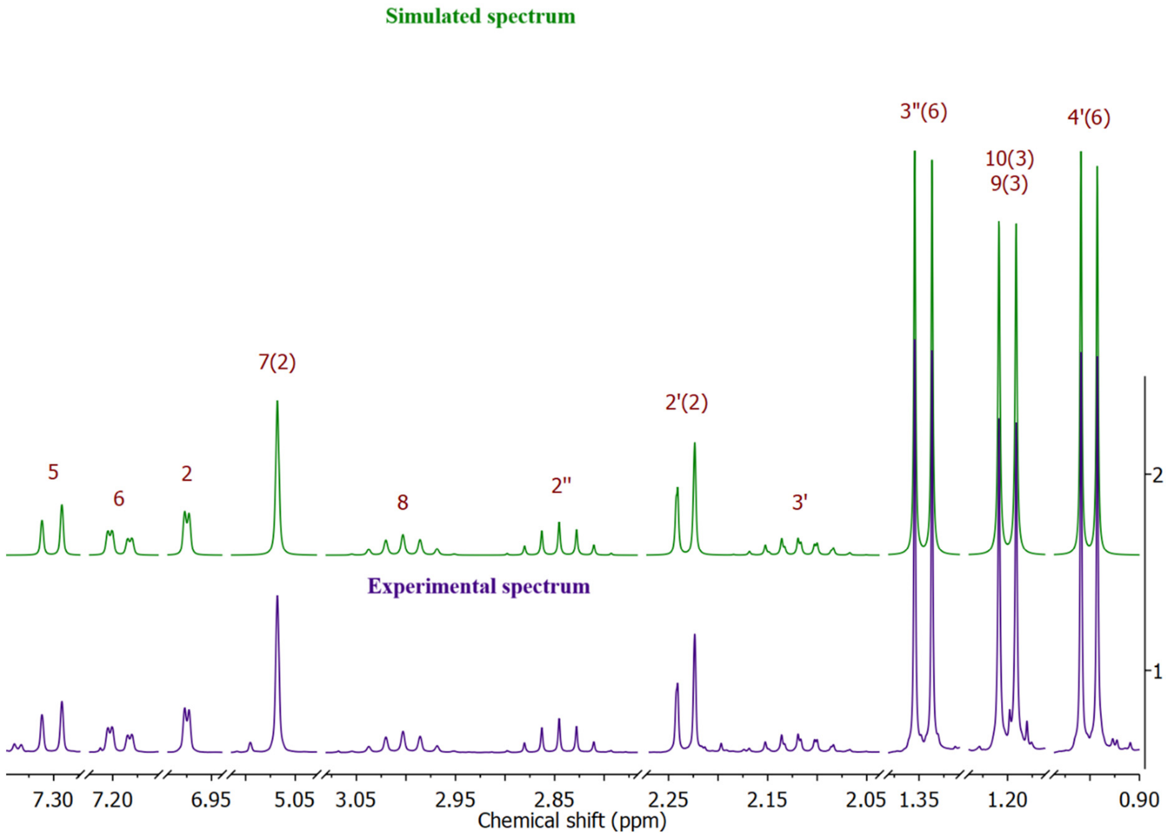

Figure S99. Simulated (manual iterative full spin, MestreNova 11.0.3) and experimental ^{1}H NMR spectrum of 7-isovaleroxythymyl isobutyrate.

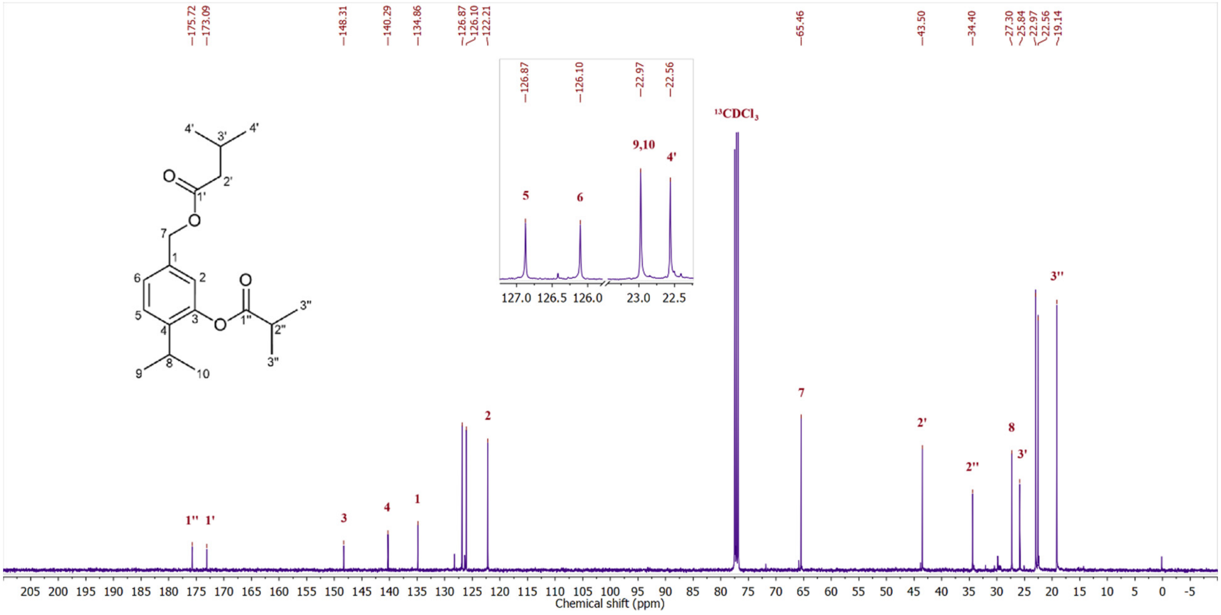

Figure S100. ^{13}C NMR (100.6 MHz, $CDCl_3$) spectrum of 7-isovaleroxythymyl isobutyrate.

Table S17. ^{1}H (400 MHz) and ^{13}C (100.6 MHz) NMR data of 7-isovaleroxythymyl isobutyrate (chloroform-*d*); NMR parameters are derived from manual iterative full spin analysis, along with the observed gHMBC and NOESY correlations.

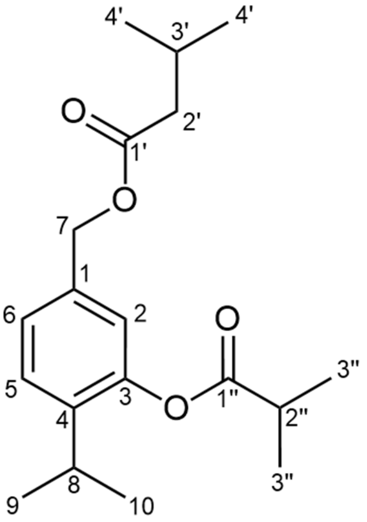

Position	δ$_H$ (m, *J* (Hz), Integration)	δ$_C$	HMBC[a]	NOESY[b]
1	/	134.86	/	/
2	6.9749 (dtd, $^{4}J_{2,6}$ = 1.8, $^{4}J_{2,7}$ = -0.5, $^{5}J_{2,8}$ = 0.3, 1 H)	122.21	3,4,6,7	7
3	/	148.31	/	/
4	/	140.29	/	/
5	7.3008 (ddt, $^{3}J_{5,6}$ = 8.0, $^{4}J_{5,8}$ = -0.6, $^{5}J_{5,7}$ = 0.3, 1 H)	126.87	1,3,8	6,8,9,10
6	7.1937 (ddtd, $^{3}J_{5,6}$ = 8.0, $^{4}J_{2,6}$ = 1.8, $^{4}J_{6,7}$ = -0.6, $^{5}J_{6,8}$ = 0.4, 1 H)	126.10	2,4,7	5,7
7	5.0680 (ddd, $^{4}J_{6,7}$ = -0.6, $^{4}J_{2,7}$ = -0.5, $^{5}J_{5,7}$ = 0.3, 2 H)	65.46	1,2,6,1′	2,6
8	3.0028 (septddd, $^{3}J_{8,9/10}$ = 6.9, $^{4}J_{5,8}$ = -0.6, $^{5}J_{6,8}$ = 0.4, $^{5}J_{2,8}$ = 0.3, 1 H)	27.30	3,4,5,9,10	5,9,10
9 and 10	1.2001 (d, $^{3}J_{8,9/10}$ = 6.9, 6 H)	22.97	4,8,9,10	5,8,9,10
1′	/	173.09	/	/
2′	2.2318 (d, $^{3}J_{2',3'}$ = 7.2, 2 H)	43.50	1′,3′,4′	3′,4′
3′	2.1190 (tsept, $^{3}J_{2',3'}$ = 7.2, $^{3}J_{3',4'}$ = 6.7, 1 H)	25.84	1′,2′,4′	2′,4′
4′	0.9509 (d, $^{3}J_{3',4'}$ = 6.7, 6 H)	22.56	2′,3′,4′	2′,3′
1″	/	175.72	/	/
2″	2.8451 (sept, $^{3}J_{2'',3''}$ = 7.0, 1 H)	34.40	1″,3″	3″
3″	1.3457 (d, $^{3}J_{2'',3''}$ = 7.0, 6 H)	19.14	1″,2″,3″	2″

[a] gHMBC correlations observed between the hydrogen in this row and the carbon in the listed position.

[b] Cross-peaks observed in the NOESY spectrum.

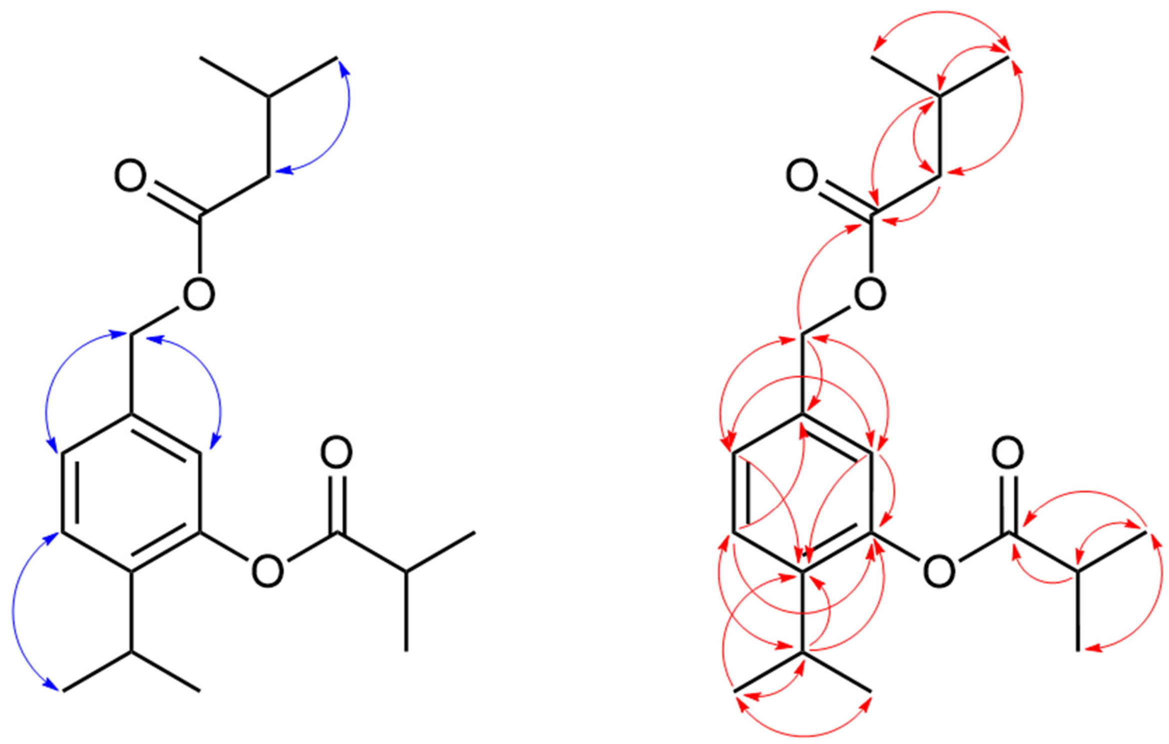

Figure S101. NOESY (blue arrows) and HMBC (red arrows) interactions of 7-isovaleroxythymyl isobutyrate.

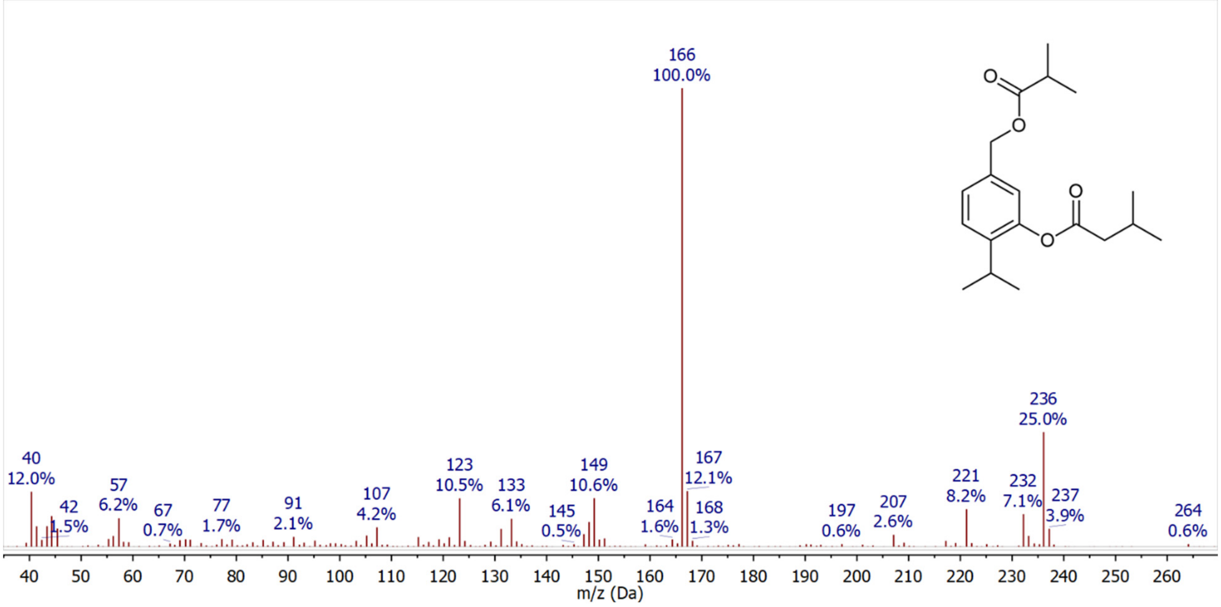

Figure S102. Mass spectrum (EI, 70 eV) of 7-isobutyryloxythymyl isovalerate.

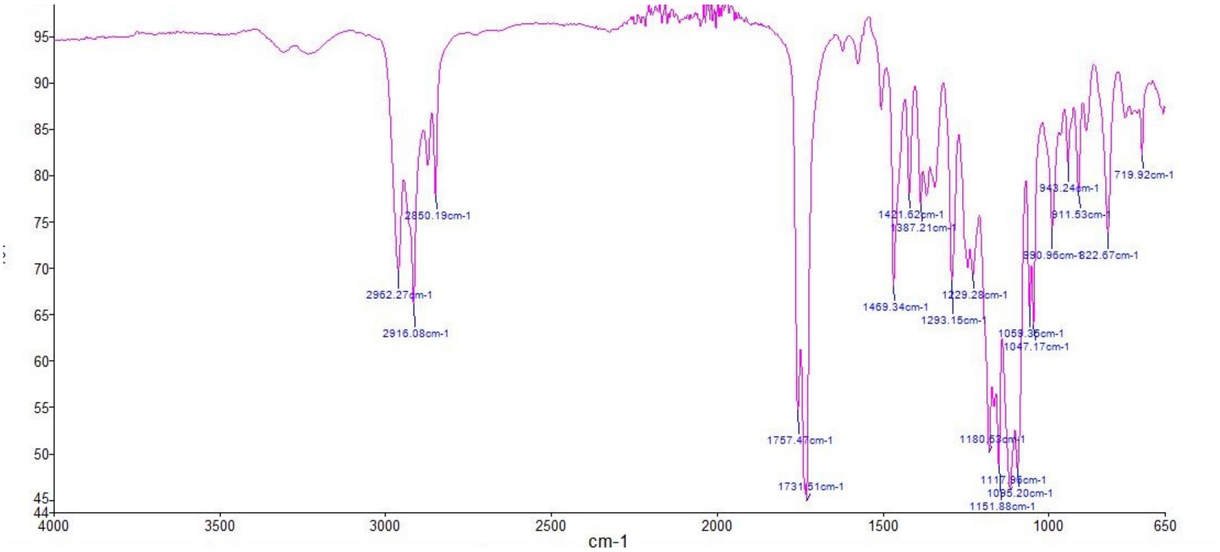

Figure S103. IR spectrum of 7-isobutyryloxythymyl isovalerate.

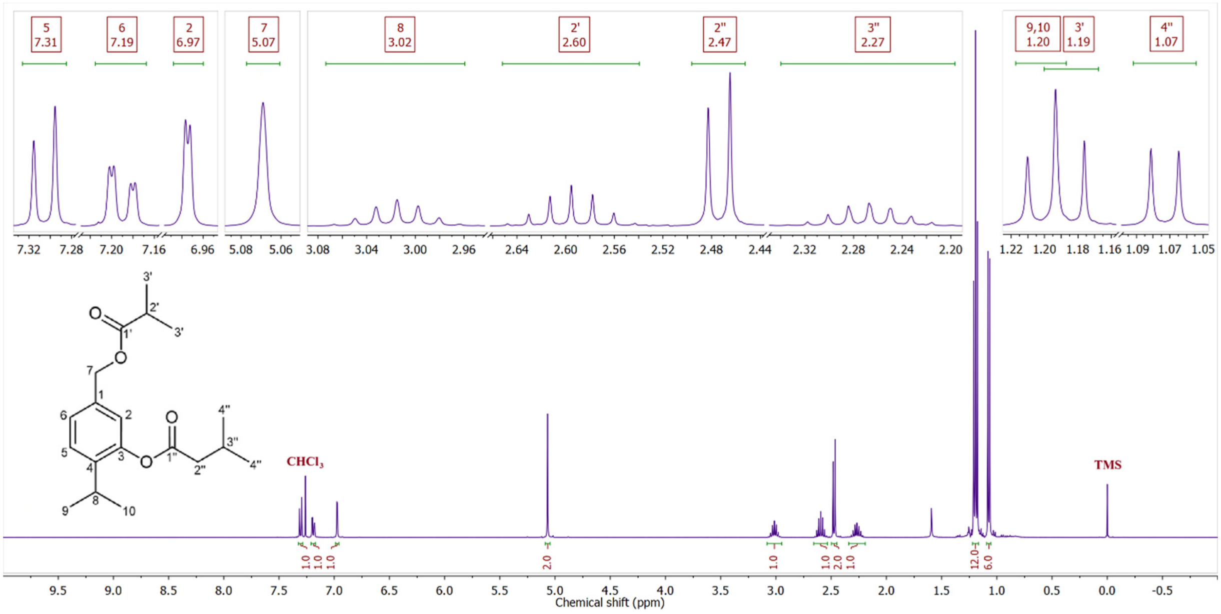

Figure S104. ^{1}H NMR (400 MHz, $CDCl_3$) spectrum of 7-isobutyryloxythymyl isovalerate and the corresponding expansions with signal assignment.

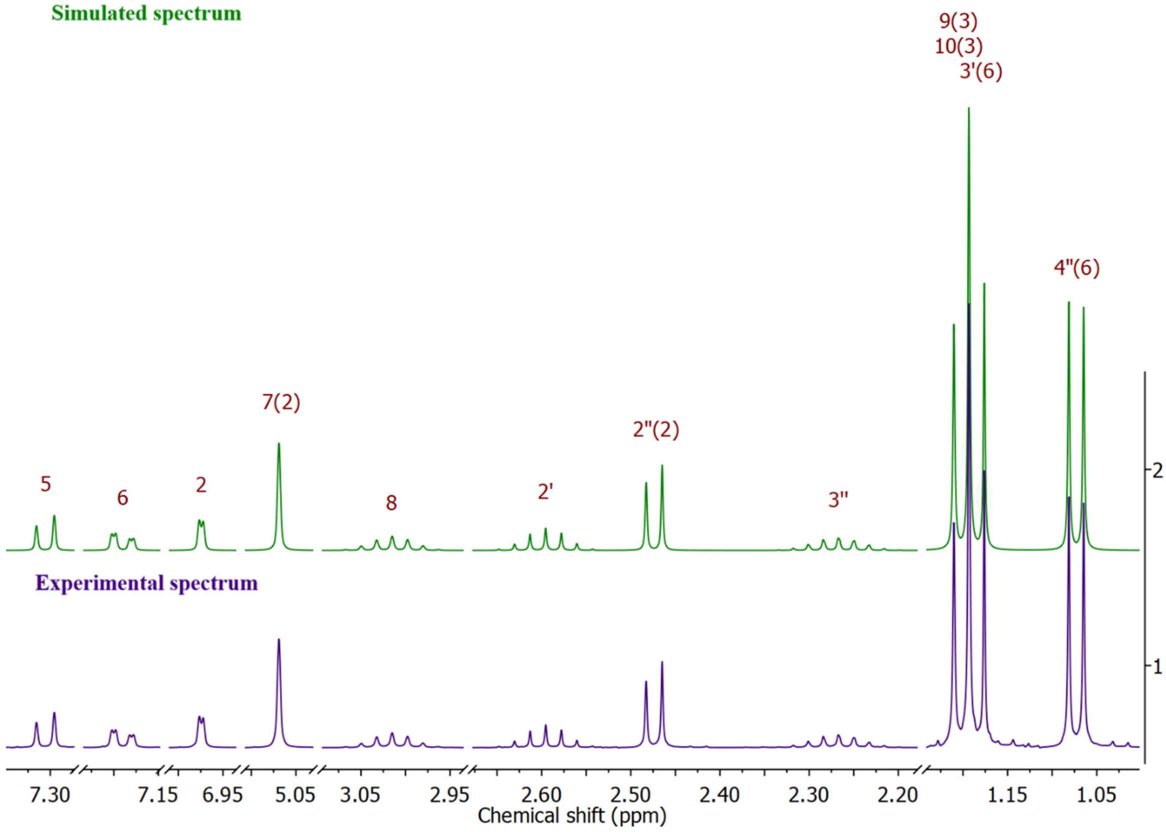

Figure S105. Simulated (manual iterative full spin, MestreNova 11.0.3) and experimental ^{1}H NMR spectrum of 7-isobutyryloxythymyl isovalerate.

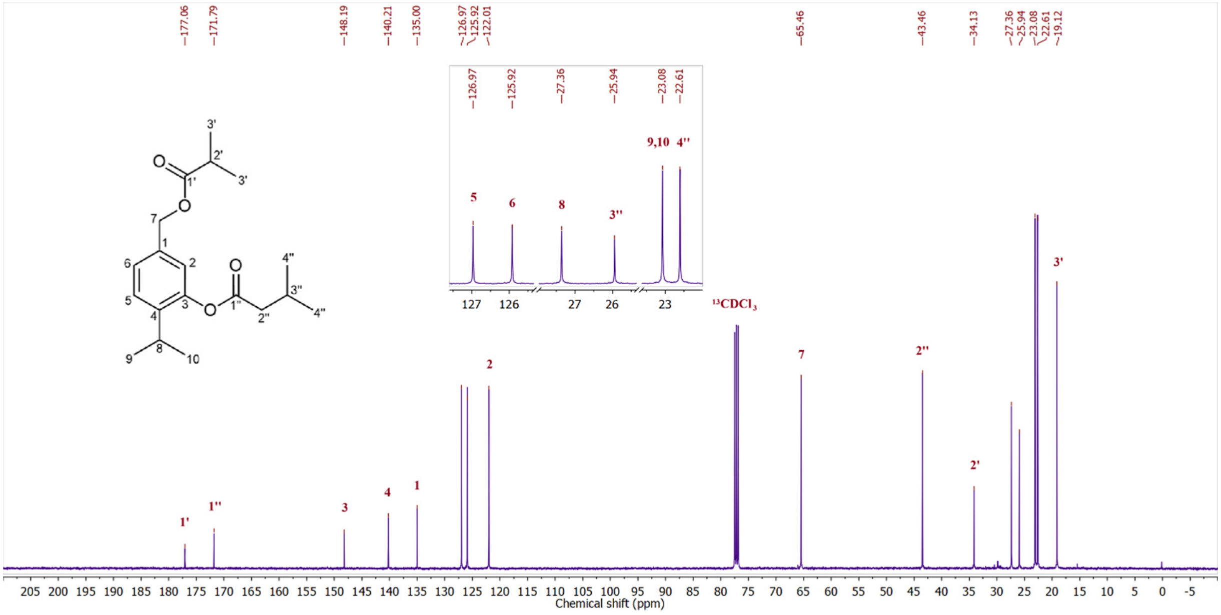

Figure S106. ^{13}C NMR (100.6 MHz, CDCl$_3$) spectrum of 7-isobutyryloxythymyl isovalerate.

Table S18. ^{1}H (400 MHz) and ^{13}C (100.6 MHz) NMR data of 7-isobutyryloxythymyl isovalerate (chloroform-*d*); NMR parameters are derived from manual iterative full spin analysis, along with the observed gHMBC and NOESY correlations.

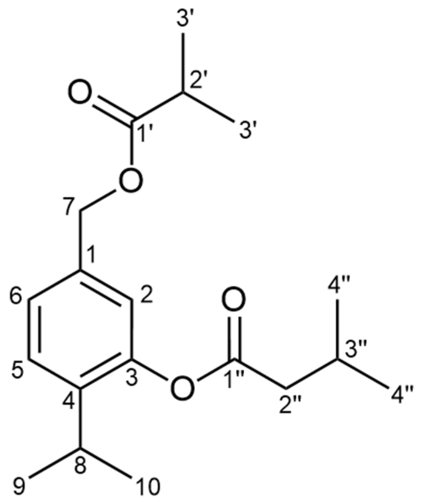

Position	δH (m, *J* (Hz), Integration)	δC	HMBC[a]	NOESY[b]
1	/	135.00	/	/
2	6.9744 (dtd, $^{4}J_{2,6}$ = 1.8, $^{4}J_{2,7}$ = -0.6, $^{5}J_{2,8}$ = 0.3, 1 H)	122.01	3,4,6,7	7
3	/	148.19	/	/
4	/	140.21	/	/
5	7.3046 (ddt, $^{3}J_{5,6}$ = 8.0, $^{4}J_{5,8}$ = -0.6, $^{5}J_{5,7}$ = 0.3, 1 H)	126.97	1,3,8	6,8,9,10
6	7.1909 (ddtd, $^{3}J_{5,6}$ = 8.0, $^{4}J_{2,6}$ = 1.8, $^{4}J_{6,7}$ = -0.6, $^{5}J_{6,8}$ = 0.4, 1 H)	125.92	2,4,7	5,7
7	5.0690 (ddd, $^{4}J_{2,7}$ = -0.6, $^{4}J_{6,7}$ = -0.6, $^{5}J_{5,7}$ = 0.3, 2 H)	65.46	1,2,6,1′	2,6
8	3.0147 (septddd, $^{3}J_{8,9/10}$ = 6.9, $^{4}J_{5,8}$ = -0.6, $^{5}J_{6,8}$ = 0.4, $^{5}J_{2,8}$ = 0.3, 1 H)	27.36	3,4,5,9,10	5,9,10
9 and 10	1.2016 (d, $^{3}J_{8,9/10}$ = 6.9, 6 H)	23.08	4,8,9,10	5,8,9,10
1′	/	177.06	/	/
2′	2.5951 (sept, $^{3}J_{2',3'}$ = 7.0, 1 H)	34.13	1′,3′	3′
3′	1.1850 (d, $^{3}J_{2',3'}$ = 7.0, 6 H)	19.12	1′,2′,3′	2′
1″	/	171.79	/	/
2″	2.4735 (d, $^{3}J_{2'',3''}$ = 7.2, 2 H)	43.46	1″,3″,4″	3″,4″
3″A	2.2672 (tsept, $^{3}J_{2'',3''}$ = 7.2, $^{3}J_{3'',4''}$ = 6.7, 1 H)	25.94	1″,2″,4″	2″,4″
4″	1.0731 (d, $^{3}J_{3'',4''}$ = 6.7, 6 H)	22.61	2″,3″,4″	2″,3″

[a] gHMBC correlations observed between the hydrogen in this row and the carbon in the listed position.

[b] Cross-peaks observed in the NOESY spectrum.

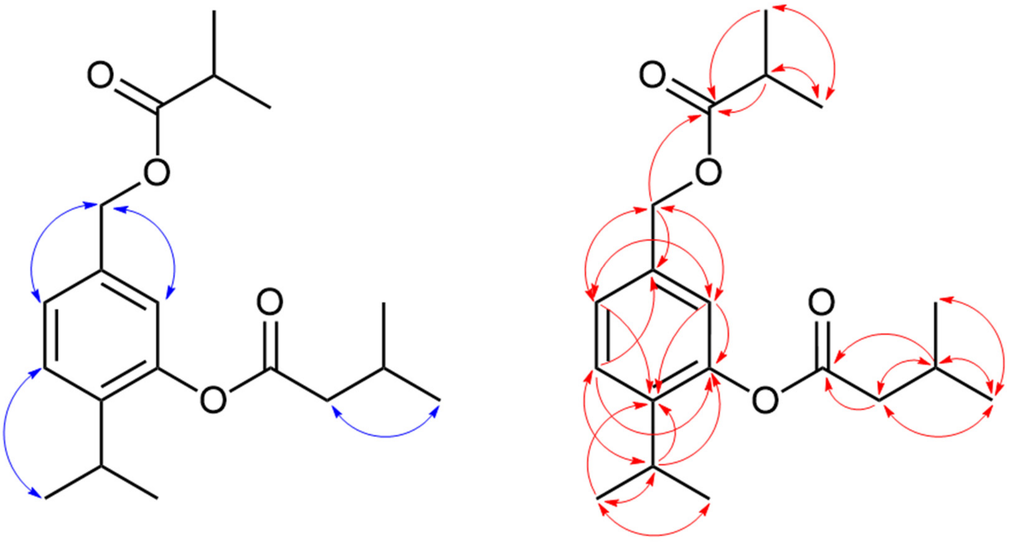

Figure S107. NOESY (blue arrows) and HMBC (red arrows) interactions of 7-isobutyryloxythymyl isovalerate.

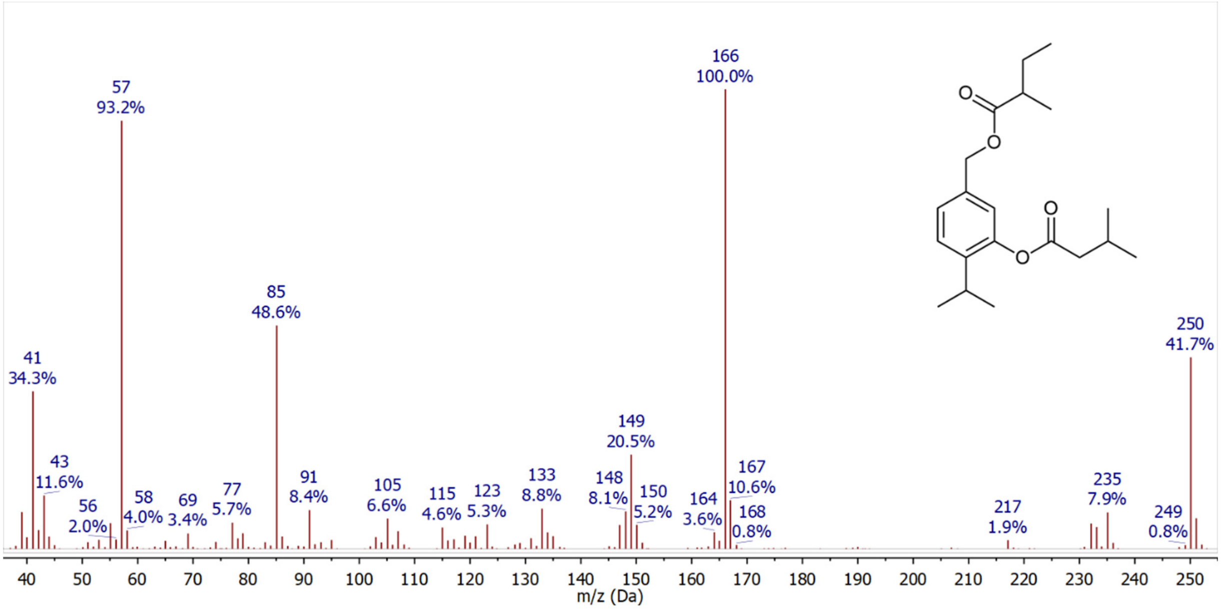

Figure S108. Mass spectrum (EI, 70 eV) of 7-(2-methylbutyryloxy)thymyl isovalerate.

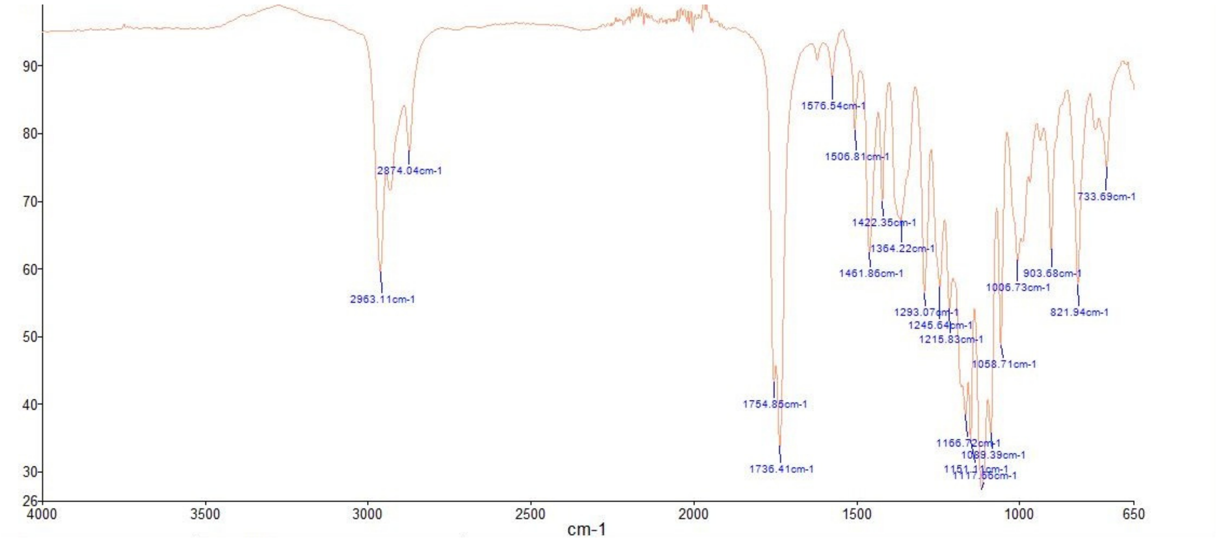

Figure S109. IR spectrum of 7-(2-methylbutyryloxy)thymyl isovalerate.

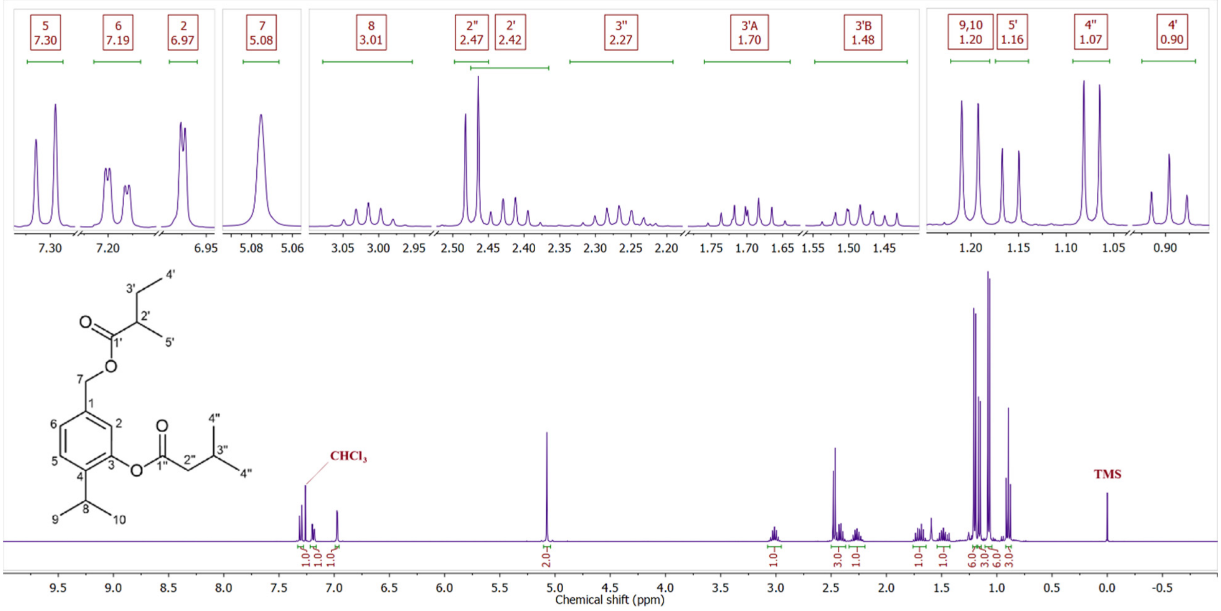

Figure S110. ^1H NMR (400 MHz, $CDCl_3$) spectrum of 7-(2-methylbutyryloxy)thymyl isovalerate and the corresponding expansions with signal assignment.

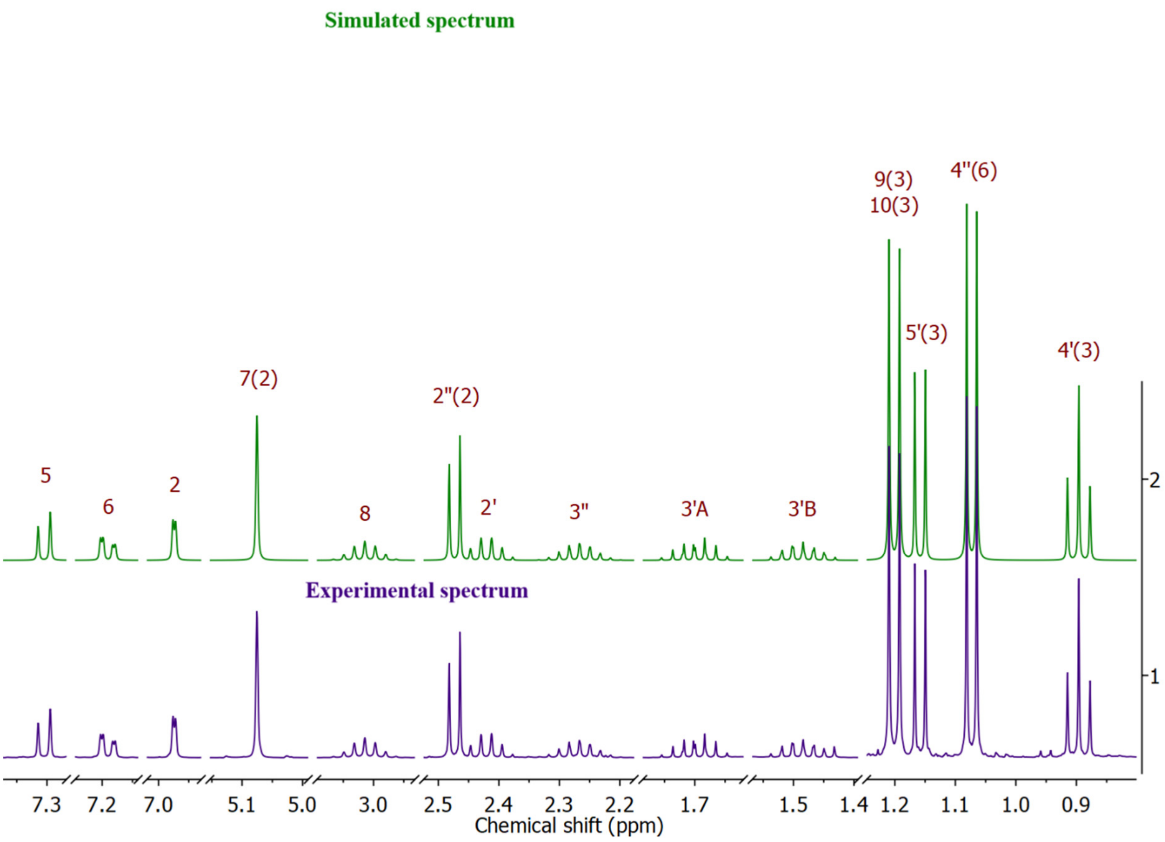

Figure S111. Simulated (manual iterative full spin, MestreNova 11.0.3) and experimental ^{1}H NMR spectrum of 7-(2-methylbutyryloxy)thymyl isovalerate.

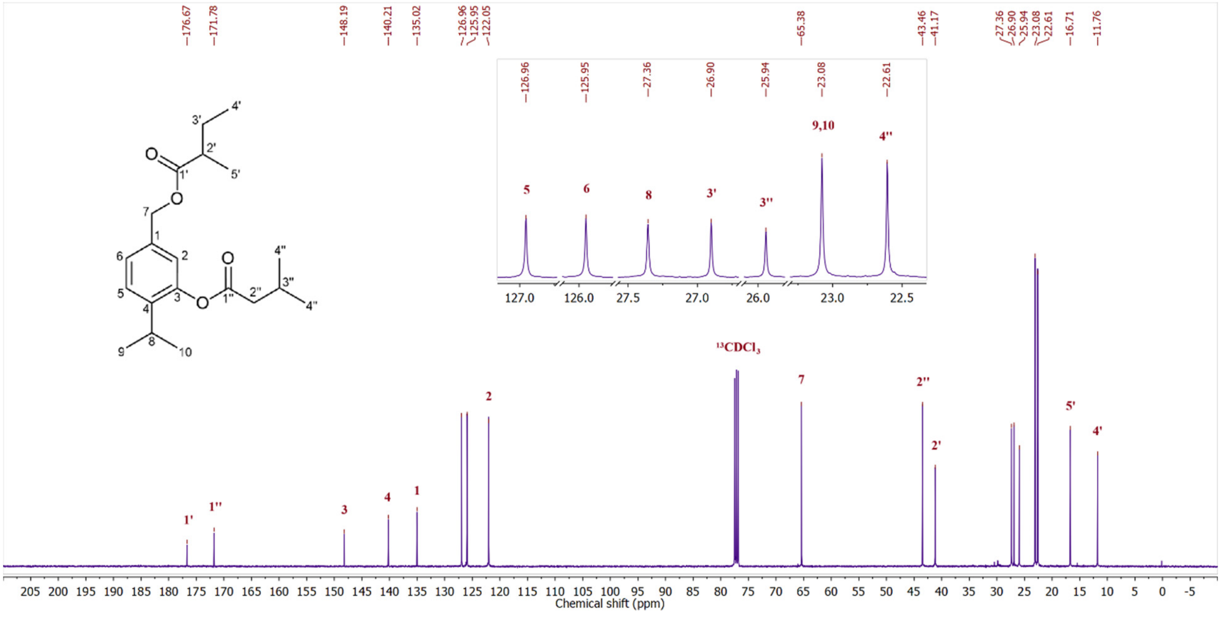

Figure S112. ^{13}C NMR (100.6 MHz, $CDCl_3$) spectrum of 7-(2-methylbutyryloxy)thymyl isovalerate.

Table S19. ^{1}H (400 MHz) and ^{13}C (100.6 MHz) NMR data of 7-(2-methylbutyryloxy)thymyl isovalerate (chloroform-*d*); NMR parameters are derived from manual iterative full spin analysis, along with the observed gHMBC and NOESY correlations.

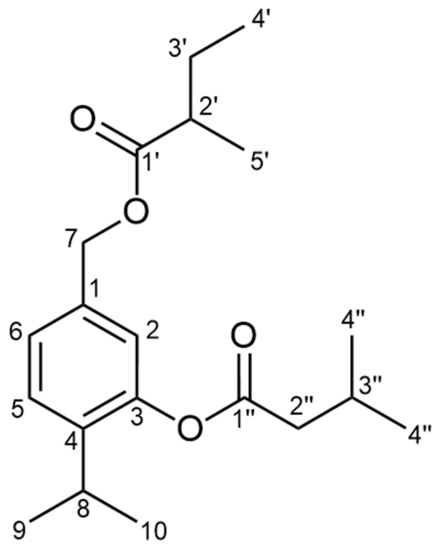

Position	δ_H (m, *J* (Hz), Integration)	δ_C	HMBC[a]	NOESY[b]
1	/	135.02	/	/
2	6.9740 (dtd, $^{4}J_{2,6}$ = 1.8, $^{4}J_{2,7}$ = -0.5, $^{5}J_{2,8}$ = 0.4, 1 H)	122.05	3,4,6,7	7
3	/	148.19	/	/
4	/	140.21	/	/
5	7.3038 (ddt, $^{3}J_{5,6}$ = 8.0, $^{4}J_{5,8}$ = -0.6, $^{5}J_{5,7}$ = 0.3, 1 H)	126.96	1,3,8	6,8,9,10
6	7.1915 (ddtd, $^{3}J_{5,6}$ = 8.0, $^{4}J_{2,6}$ = 1.8, $^{4}J_{6,7}$ = -0.5, $^{5}J_{6,8}$ = 0.4, 1 H)	125.95	2,4,7	5,7
7	5.0754 (ddd, $^{4}J_{2,7}$ = -0.5, $^{4}J_{6,7}$ = -0.5, $^{5}J_{5,7}$ = 0.3, 2 H)	65.38	1,2,6,1′	2,6
8	3.0142 (septddd, $^{3}J_{8,9/10}$ = 6.9, $^{4}J_{5,8}$ = -0.6, $^{5}J_{2,8}$ = 0.4, $^{5}J_{6,8}$ = 0.4, 1 H)	27.36	3,4,5,9,10	5,9,10
9 and 10	1.2011 (d, $^{3}J_{8,9/10}$ = 6.9, 6 H)	23.08	4,8,9,10	5,8,9,10
1′	/	176.67	/	/
2′	2.4203 (dqdq, $^{3}J_{2',3'A}$ = 7.5, $^{3}J_{2',5'}$ = 7.0, $^{3}J_{2',3'B}$ = 6.5, $^{4}J_{2',4'}$ = 0.3, 1 H)	41.17	1′,3′,4′,5′	3′,4′,5′
3′A	1.6992 (ddq, $^{2}J_{3'A,3'B}$ = -13.7, $^{3}J_{2',3'A}$ = 7.5, $^{3}J_{3'A,4'}$ = 7.5, 1 H)	26.90	1′,2′,4′,5′	2′,4′,5′
3′B	1.4851 (dqd, $^{2}J_{3'A,3'B}$ = -13.7, $^{3}J_{3'B,4'}$ = 7.5, $^{3}J_{2',3'B}$ = 6.5, 1 H)	26.90	1′,2′,4′,5′	2′,4′,5′
4′	0.8963 (ddd, $^{3}J_{3'A,4'}$ = 7.5, $^{3}J_{3'B,4'}$ = 7.5, $^{4}J_{2',4'}$ = 0.3, 3 H)	11.76	2′,3′	2′.3′
5′	1.1584 (d, $^{3}J_{2',5'}$ = 7.0, 3 H)	16.71	1′,2′,3′	2′,3′
1′′	/	171.78	/	/
2′′	2.4727 (d, $^{3}J_{2'',3''}$ = 7.2, 2 H)	43.46	1′′,3′′,4′′	3′′,4′′
3′′	2.2668 (tsept, $^{3}J_{2'',3''}$ = 7.2, $^{3}J_{3'',4''}$ = 6.7, 1 H)	25.94	1′′,2′′,4′′	2′′,4′′
4′′	1.0731 (d, $^{3}J_{3'',4''}$ = 6.7, 6 H)	22.61	2′′,3′′,4′′	2′′,3′′

[a] gHMBC correlations observed between the hydrogen in this row and the carbon in the listed position.

[b] Cross-peaks observed in the NOESY spectrum.

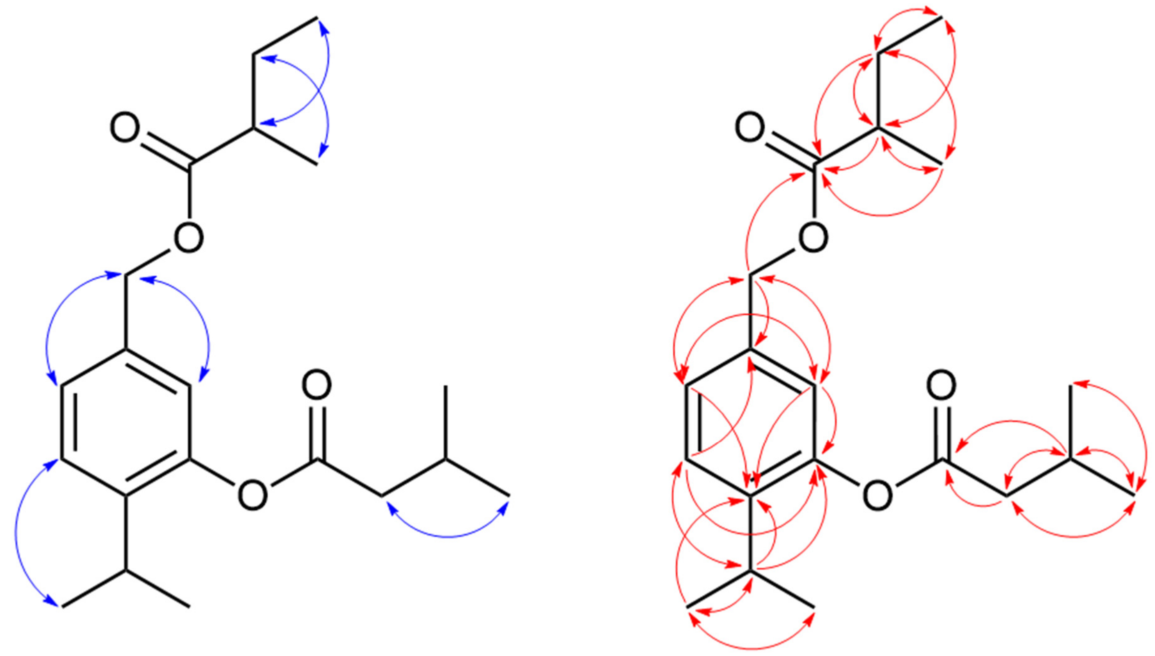

Figure S113. NOESY (blue arrows) and HMBC (red arrows) interactions of 7-(2-methylbutyryloxy)thymyl isovalerate.

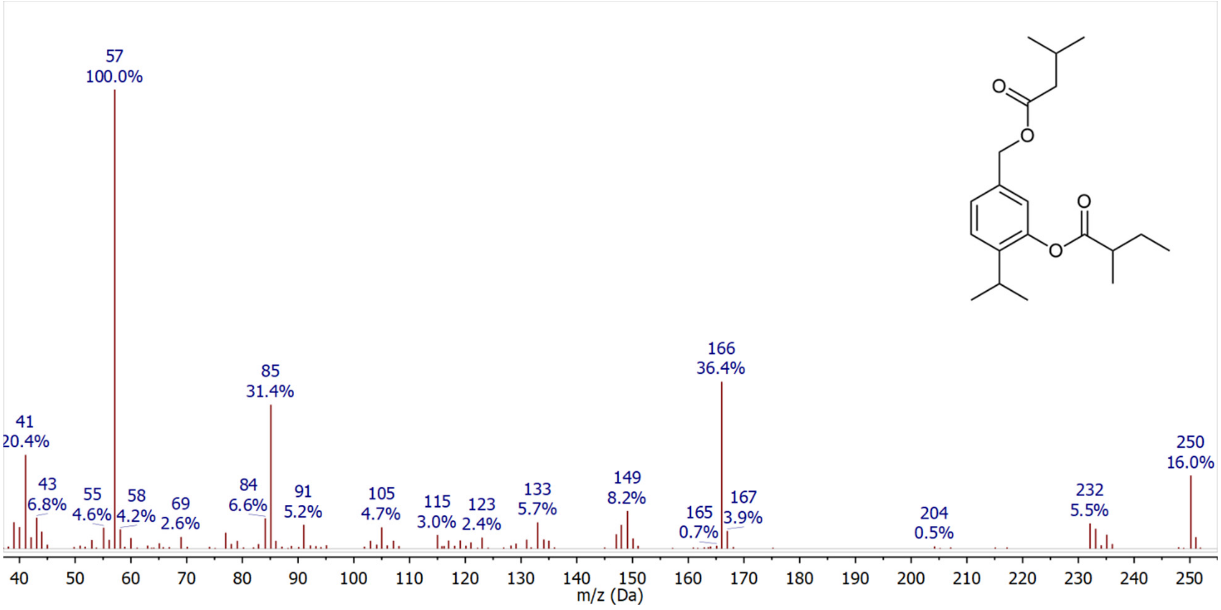

Figure S114. Mass spectrum (EI, 70 eV) of 7-isovaleroxythymyl 2-methylbutyrate.

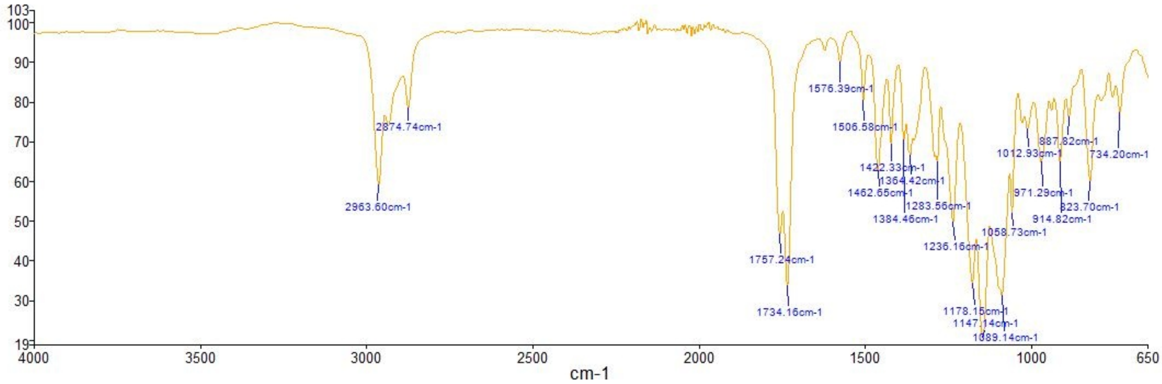

Figure S115. IR spectrum of 7-isovaleroxythymyl 2-methylbutyrate.

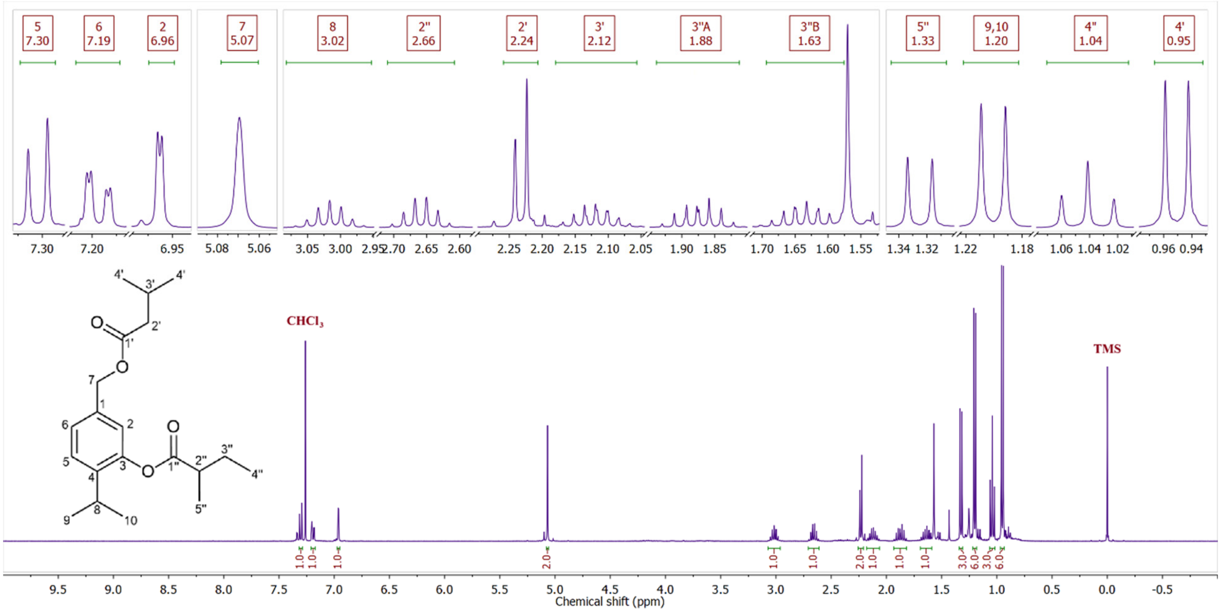

Figure S116. ^{1}H NMR (400 MHz, $CDCl_3$) spectrum of 7-isovaleroxythymyl 2-methylbutyrate and the corresponding expansions with signal assignment.

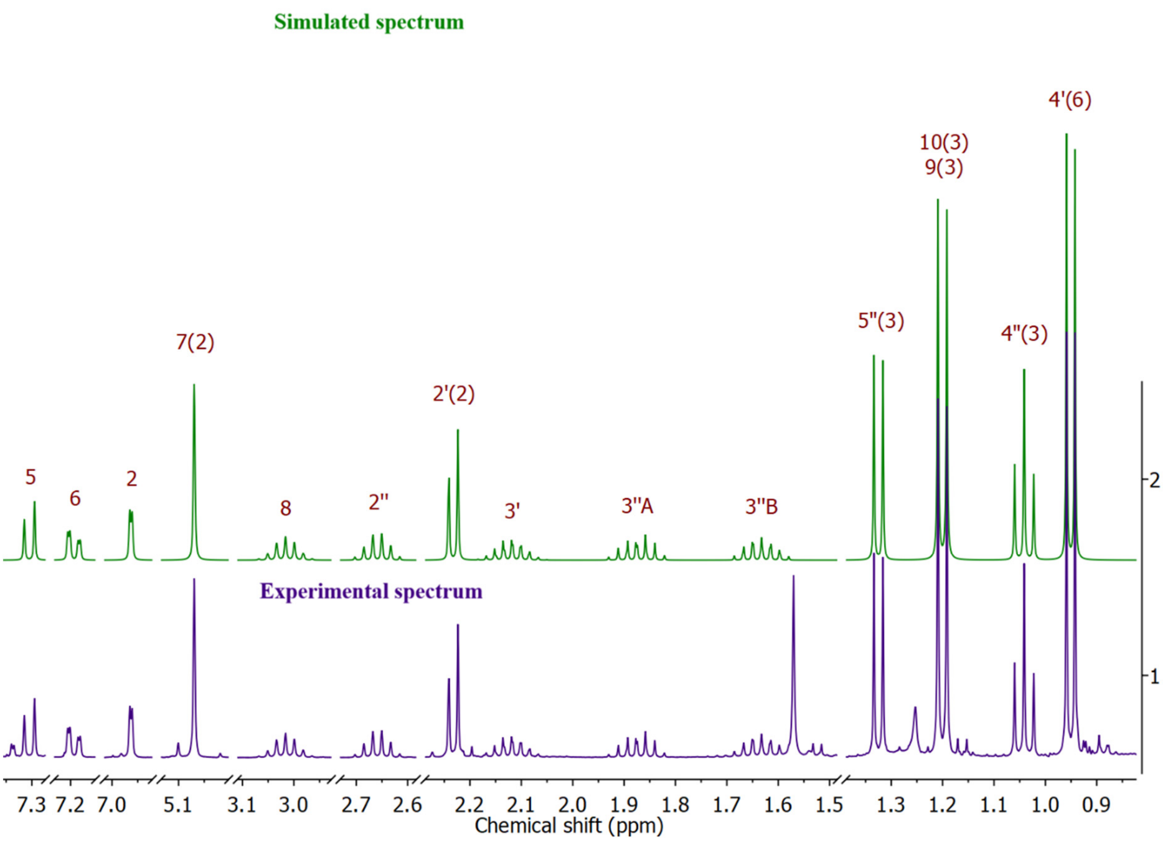

Figure S117. Simulated (manual iterative full spin, MestreNova 11.0.3) and experimental ^{1}H NMR spectrum of 7-isovaleroxythymyl 2-methylbutyrate.

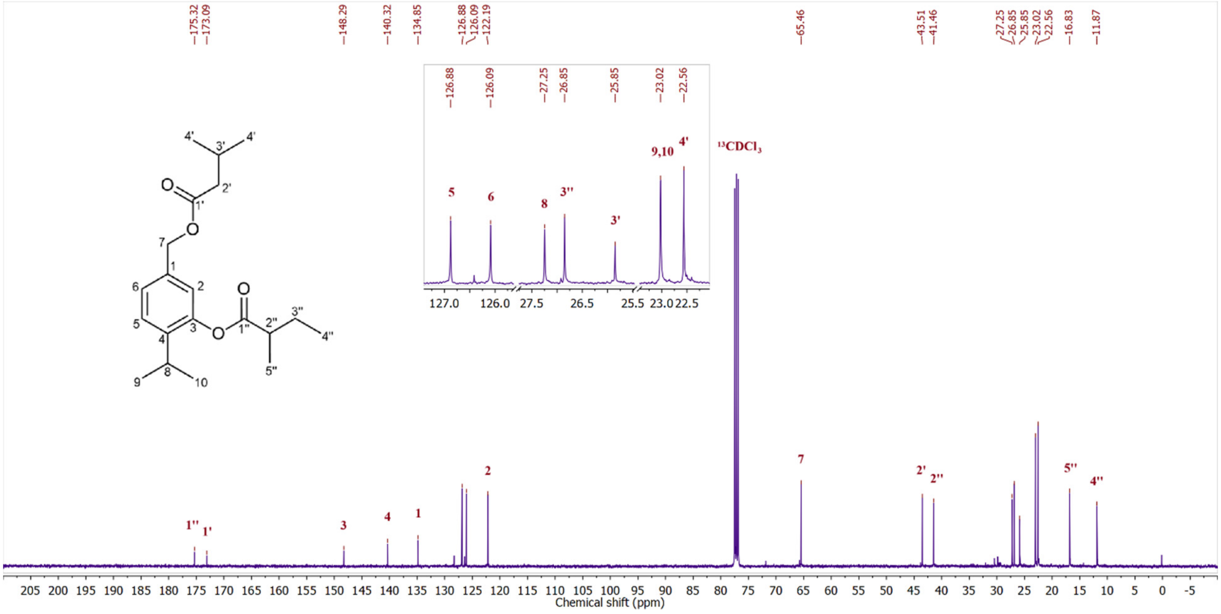

Figure S118. ^{13}C NMR (100.6 MHz, $CDCl_3$) spectrum of 7-isovaleroxythymyl 2-methylbutyrate.

Table S20. ^{1}H (400 MHz) and ^{13}C (100.6 MHz) NMR data of 7-isovaleroxythymyl 2-methylbutyrate (chloroform-*d*); NMR parameters are derived from manual iterative full spin analysis, along with the observed gHMBC and NOESY correlations.

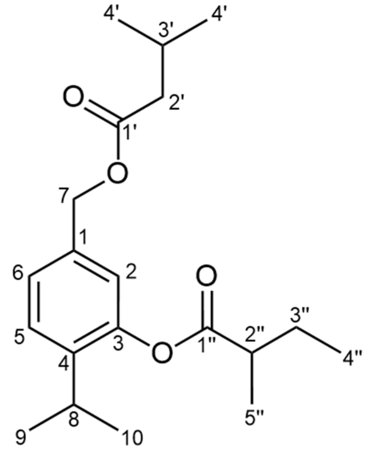

Position	δ_H (m, *J* (Hz), Integration)	δ_C	HMBC[a]	NOESY[b]
1	/	134.85	/	/
2	6.9631 (dtd, $^{4}J_{2,6}$ = 1.8, $^{4}J_{2,7}$ = -0.5, $^{5}J_{2,8}$ = 0.3, 1 H)	122.19	3,4,6,7	7
3	/	148.29	/	/
4	/	140.32	/	/
5	7.3037 (ddt, $^{3}J_{5,6}$ = 8.0, $^{4}J_{5,8}$ = -0.6, $^{5}J_{5,7}$ = 0.3, 1 H)	126.88	1,3,8	6,8,9,10
6	7.1942 (ddtd, $^{3}J_{5,6}$ = 8.0, $^{4}J_{2,6}$ = 1.8, $^{4}J_{6,7}$ = -0.6, $^{5}J_{6,8}$ = 0.4, 1 H)	126.09	2,4,7	5,7
7	5.0694 (ddd, $^{4}J_{6,7}$ = -0.6, $^{4}J_{2,7}$ = -0.5, $^{5}J_{5,7}$ = 0.3, 2 H)	65.46	1,2,6,1′	2,6
8	3.0161 (septddd, $^{3}J_{8,9/10}$ = 6.9, $^{4}J_{5,8}$ = -0.6, $^{5}J_{6,8}$ = 0.4, $^{5}J_{2,8}$ = 0.3, 1 H)	27.25	3,4,5,9,10	5,9,10
9 and 10	1.2005 (d, $^{3}J_{8,9/10}$ = 6.9, 6 H)	23.02	4,8,9,10	5,8,9,10
1′	/	173.09	/	/
2′	2.2317 (d, $^{3}J_{2',3'}$ = 7.2, 2 H)	43.51	1′,3′,4′	3′,4′
3′	2.1189 (tsept, $^{3}J_{2',3'}$ = 7.2, $^{3}J_{3',4'}$ = 6.7, 1 H)	25.85	1′,2′,4′	2′,4′
4′	0.9507 (d, $^{3}J_{3',4'}$ = 6.7, 6 H)	22.56	2′,3′,4′	2′,3′
1′′	/	175.32	/	/
2′′	2.6587 (dqdq, $^{3}J_{2'',3''A}$ = 7.5, $^{3}J_{2'',5''}$ = 7.0, $^{3}J_{2'',3''B}$ = 6.5, $^{4}J_{2'',4''}$ = 0.3, 1 H)	41.46	1′′,3′′,4′′,5′′	3′′,4′′,5′′
3′′A	1.8743 (ddq, $^{2}J_{3''A,3''B}$ = -13.7, $^{3}J_{2'',3''A}$ = 7.5, $^{3}J_{3''A,4''}$ = 7.5, 1 H)	26.85	1′′,2′′,4′′,5′′	2′′,4′′,5′′
3′′B	1.6333 (dqd, $^{2}J_{3''A,3''B}$ = -13.7, $^{3}J_{3''B,4''}$ = 7.5, $^{3}J_{2'',3''B}$ = 6.5, 1 H)	26.85	1′′,2′′,4′′,5′′	2′′,4′′,5′′
4′′	1.0415 (ddd, $^{3}J_{3''A,4''}$ = 7.5, $^{3}J_{3''B,4''}$ = 7.5, $^{4}J_{2'',4''}$ = 0.3, 3 H)	11.87	2′′,3′′	2′′.3′′
5′′	1.3251 (d, $^{3}J_{2'',5''}$ = 7.0, 3 H)	16.83	1′′,2′′,3′′	2′′,3′′

[a] gHMBC correlations observed between the hydrogen in this row and the carbon in the listed position.

[b] Cross-peaks observed in the NOESY spectrum.

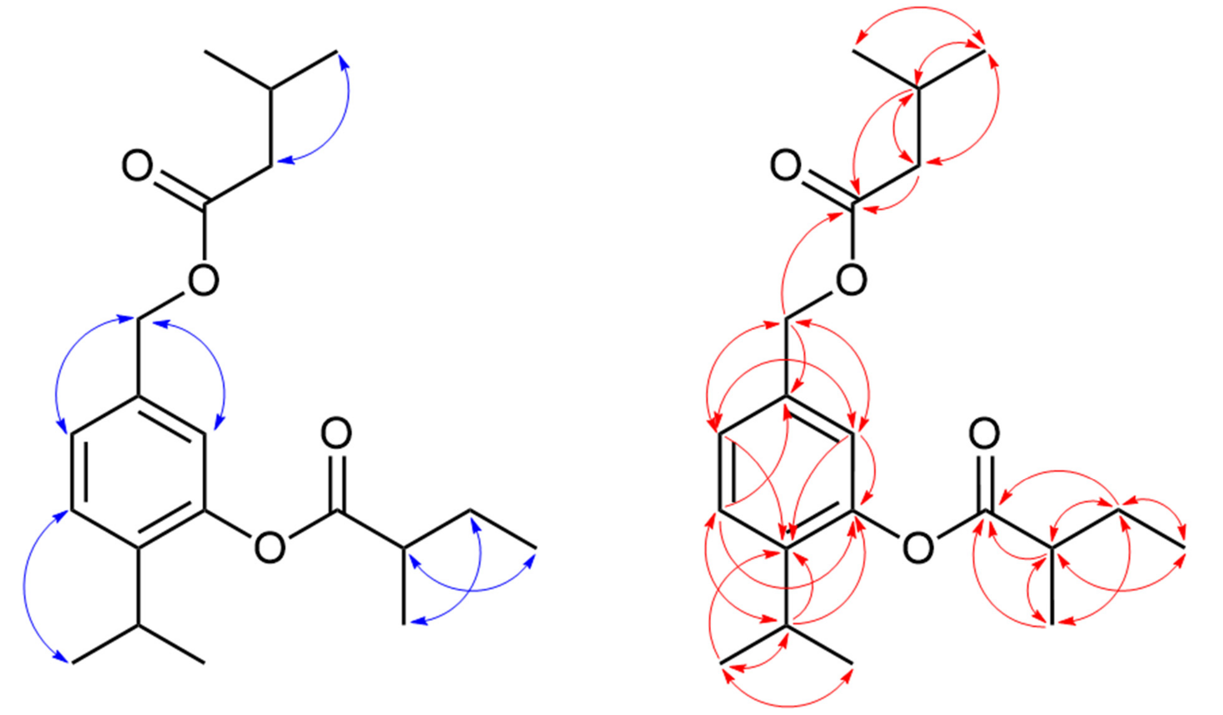

Figure S119. NOESY (blue arrows) and HMBC (red arrows) interactions of 7-isovaleroxythymyl 2-methylbutyrate.

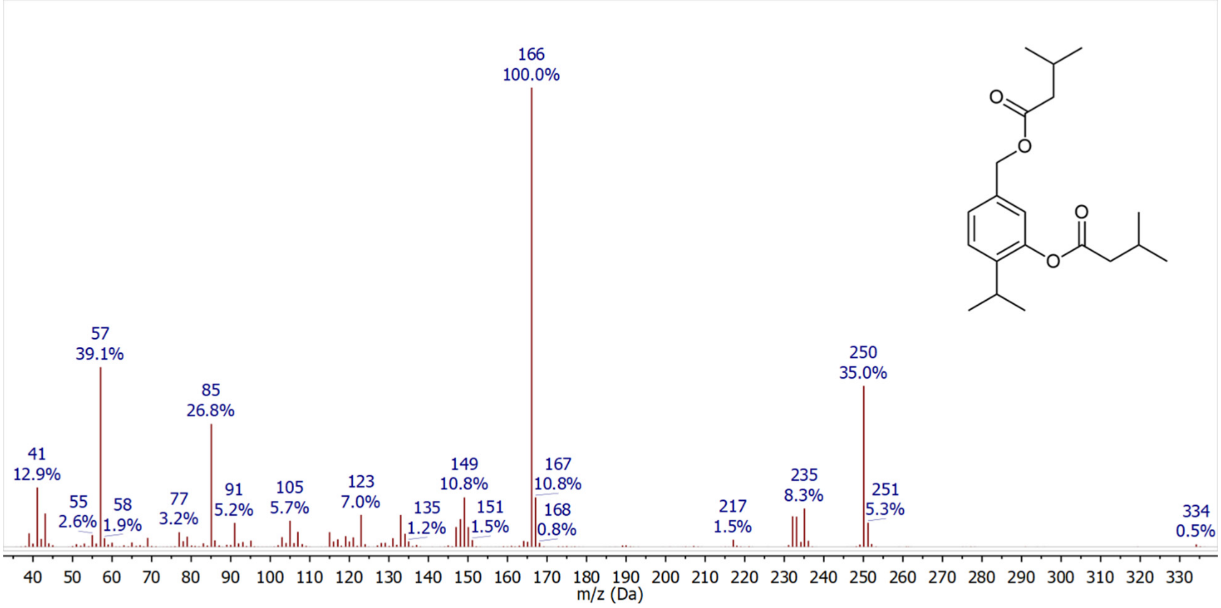

Figure S120. Mass spectrum (EI, 70 eV) of 7-isovaleroxythymyl isovalerate.

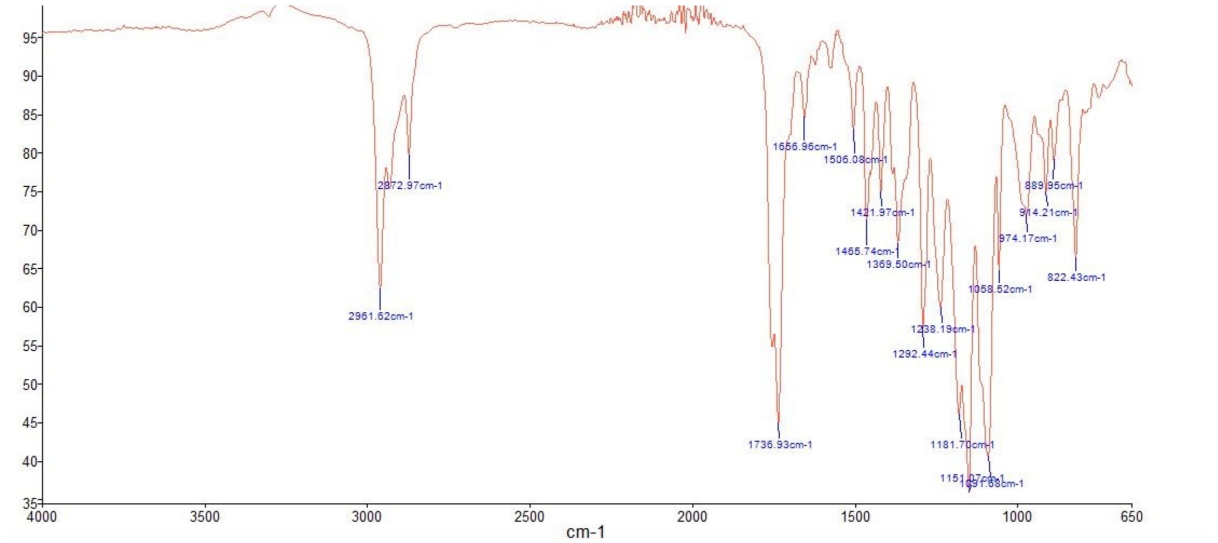

Figure S121. IR spectrum of 7-isovaleroxythymyl isovalerate.

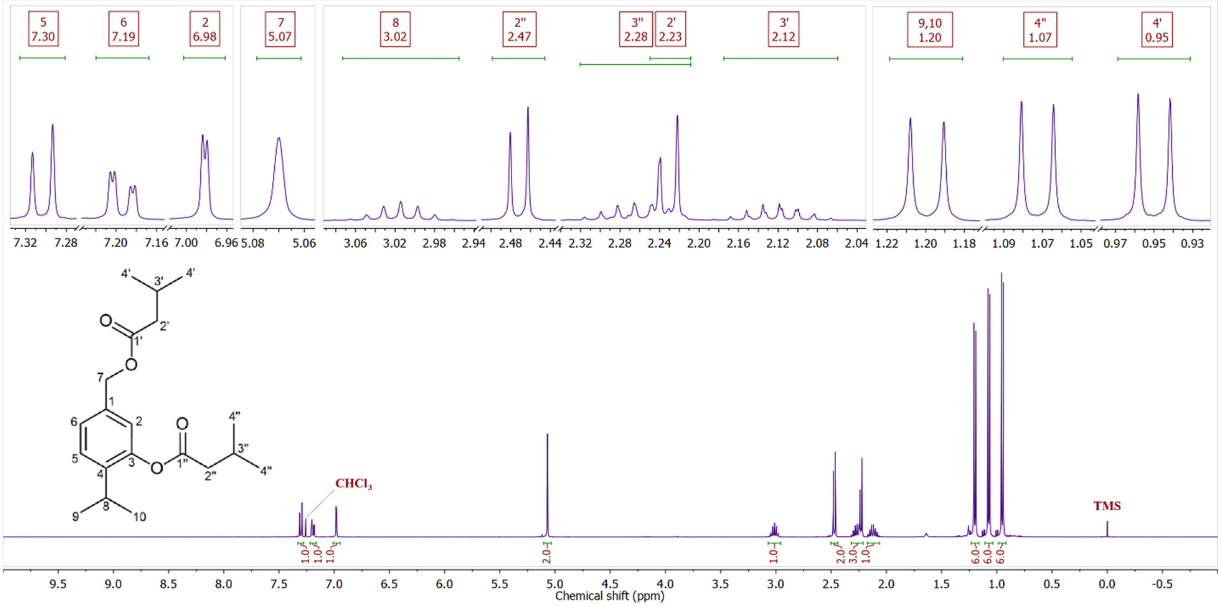

Figure S122. ^{1}H NMR (400 MHz, $CDCl_3$) spectrum of 7-isovaleroxythymyl isovalerate and the corresponding expansions with signal assignment.

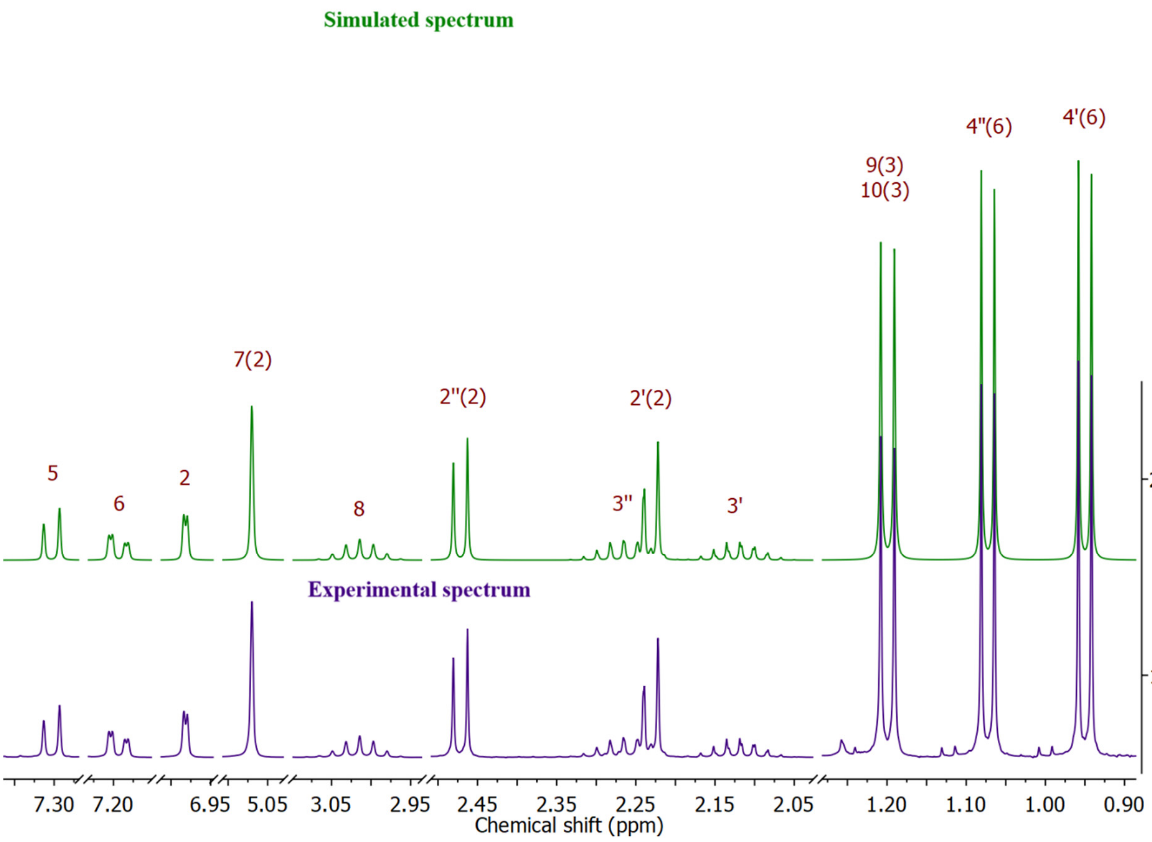

Figure S123. Simulated (manual iterative full spin, MestreNova 11.0.3) and experimental ^{1}H NMR spectrum of 7-isovaleroxythymyl isovalerate.

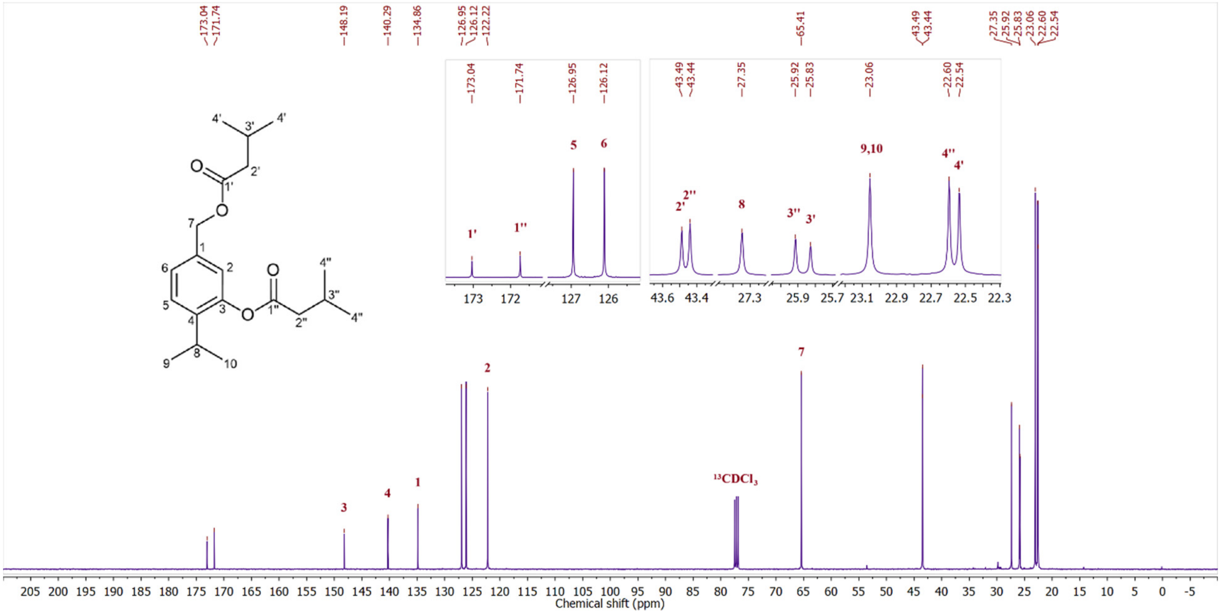

Figure S124. ^{13}C NMR (100.6 MHz, $CDCl_3$) spectrum of 7-isovaleroxythymyl isovalerate.

Table S21. ^{1}H (400 MHz) and ^{13}C (100.6 MHz) NMR data of 7-isovaleroxythymyl isovalerate (chloroform-*d*); NMR parameters are derived from manual iterative full spin analysis, along with the observed gHMBC and NOESY correlations.

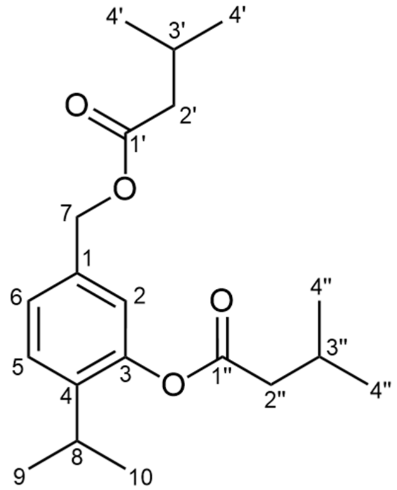

Position	δ_H (m, J (Hz), Integration)	δ_C	HMBC[a]	NOESY[b]
1	/	134.86	/	/
2	6.9817 (dtd, $^{4}J_{2,6}$ = 1.8, $^{4}J_{2,7}$ = -0.6, $^{5}J_{2,8}$ = 0.3, 1 H)	122.22	3,4,6,7	7
3	/	148.19	/	/
4	/	140.29	/	/
5	7.3023 (ddt, $^{3}J_{5,6}$ = 8.0, $^{4}J_{5,8}$ = -0.6, $^{5}J_{5,7}$ = 0.3, 1 H)	126.95	1,3,8	6,8,9,10
6	7.1944 (ddtd, $^{3}J_{5,6}$ = 8.0, $^{4}J_{2,6}$ = 1.8, $^{4}J_{6,7}$ = -0.6, $^{5}J_{6,8}$ = 0.4, 1 H)	126.12	2,4,7	5,7
7	5.0699 (ddd, $^{4}J_{2,7}$ = -0.6, $^{4}J_{6,7}$ = -0.6, $^{5}J_{5,7}$ = 0.3, 2 H)	65.41	1,2,6,1′	2,6
8	3.0141 (septddd, $^{3}J_{8,9/10}$ = 6.9, $^{4}J_{5,8}$ = -0.6, $^{5}J_{6,8}$ = 0.4, $^{5}J_{2,8}$ = 0.3, 1 H)	27.35	3,4,5,9,10	5,9,10
9 and 10	1.1993 (d, $^{3}J_{8,9/10}$ = 6.9, 6 H)	23.06	4,8,9,10	5,8,9,10
1′	/	173.04	/	/
2′	2.2303 (d, $^{3}J_{2',3'}$ = 7.2, 2 H)	43.49	1′,3′,4′	3′,4′
3′	2.1185 (tsept, $^{3}J_{2',3'}$ = 7.2, $^{3}J_{3',4'}$ = 6.7, 1 H)	25.83	1′,2′,4′	2′,4′
4′	0.9500 (d, $^{3}J_{3',4'}$ = 6.7, 6 H)	22.54	2′,3′,4′	2′,3′
1″	/	171.74	/	/
2″	2.4712 (d, $^{3}J_{2'',3''}$ = 7.2, 2 H)	43.44	1″,3″,4″	3″,4″
3″	2.2654 (tsept, $^{3}J_{2'',3''}$ = 7.2, $^{3}J_{3'',4''}$ = 6.7, 1 H)	25.92	1″,2″,4″	2″,4″
4″	1.0726 (d, $^{3}J_{3'',4''}$ = 6.7, 6 H)	22.60	2″,3″,4″	2″,3″

[a] gHMBC correlations observed between the hydrogen in this row and the carbon in the listed position.

[b] Cross-peaks observed in the NOESY spectrum.

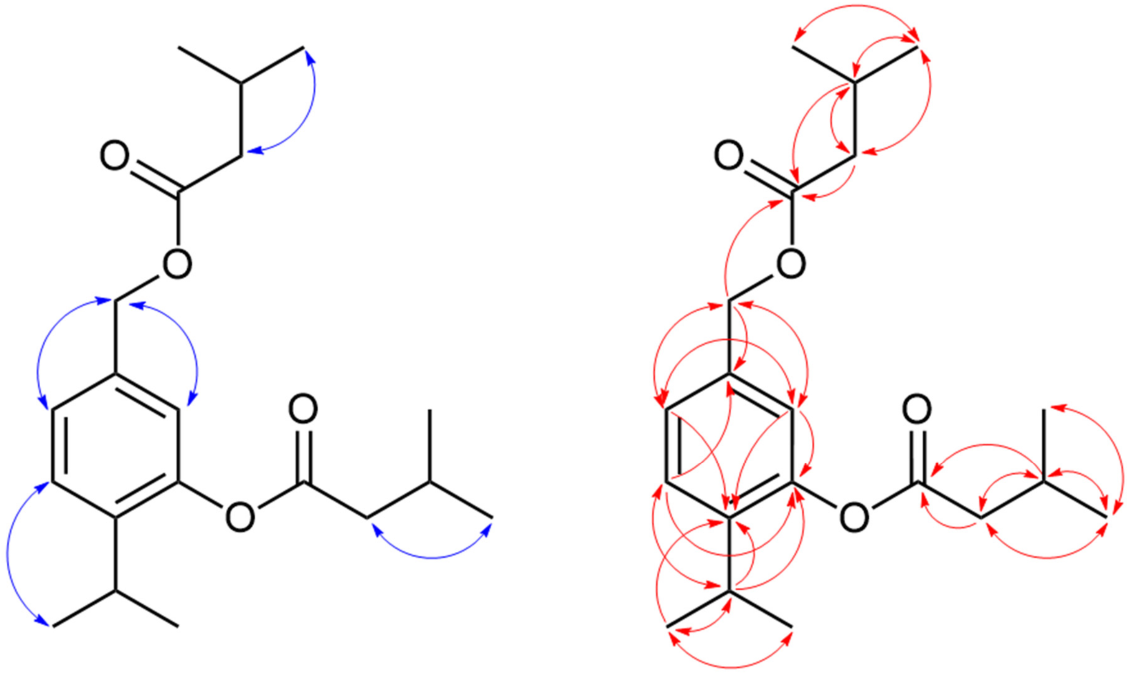

Figure S125. NOESY (blue arrows) and HMBC (red arrows) interactions of 7-isovaleroxythymyl isovalerate.

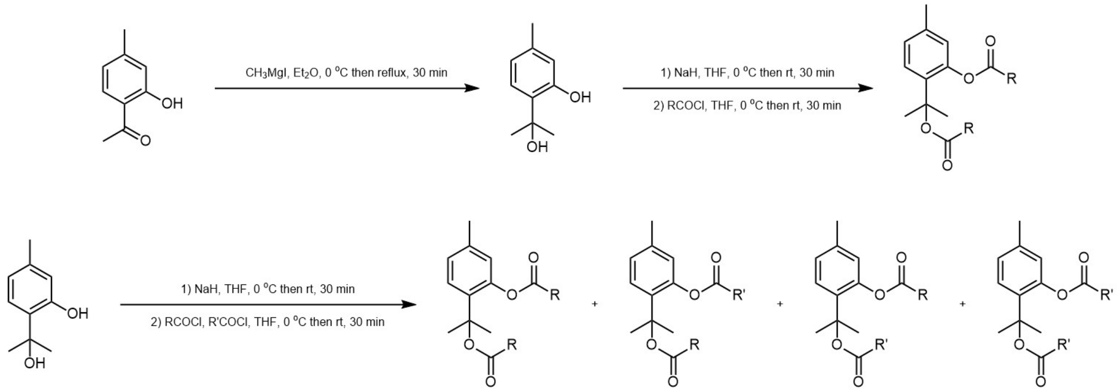

Scheme S5. Synthetic pathway to 8-hydroxythymol-derived monoesters and diesters (R,R′ – isopropyl, *sec*-butyl, or isobutyl).

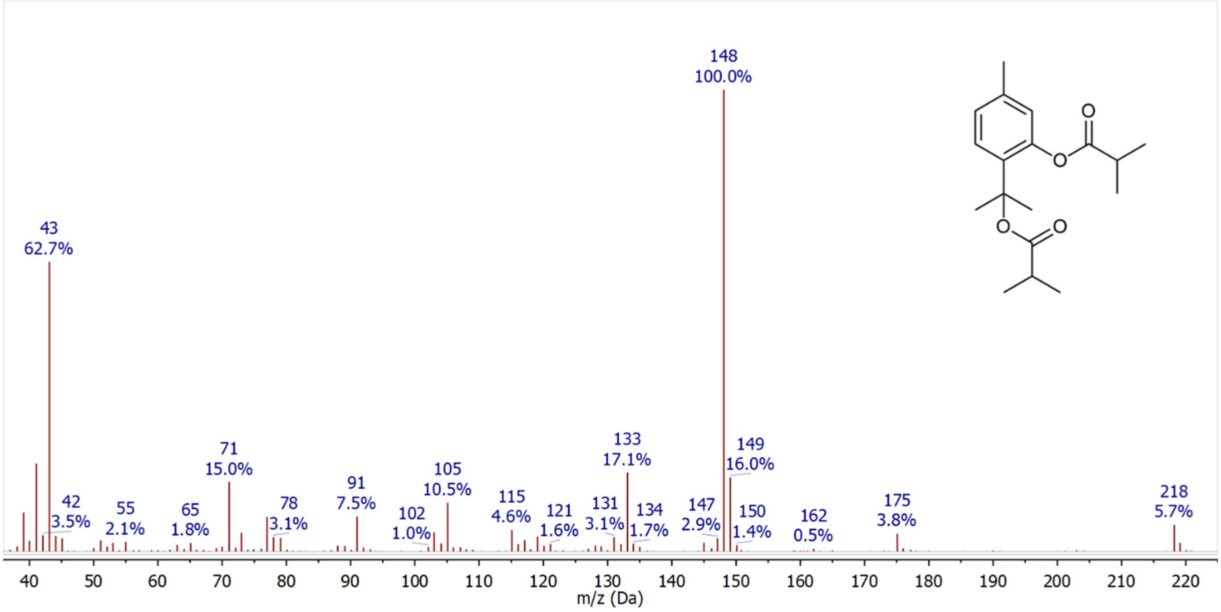

Figure S126. Mass spectrum (EI, 70 eV) of 8-isobutyryloxythymyl isobutyrate.

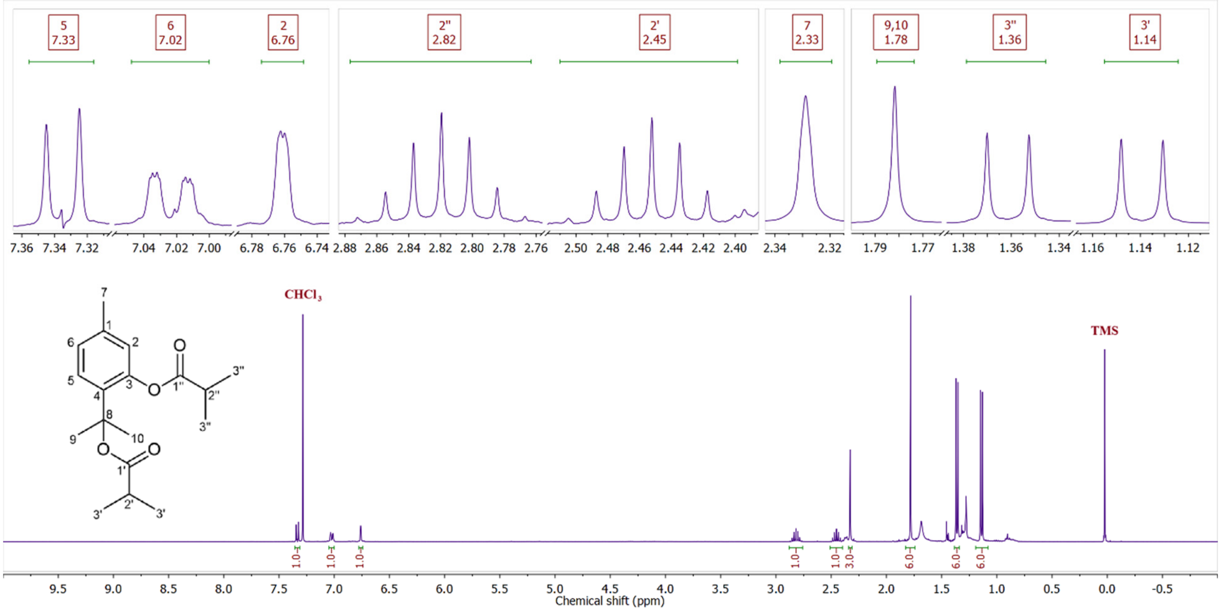

Figure S127. ^{1}H NMR (400 MHz, $CDCl_3$) spectrum of 8-isobutyryloxythymyl isobutyrate and the corresponding expansions with signal assignment.

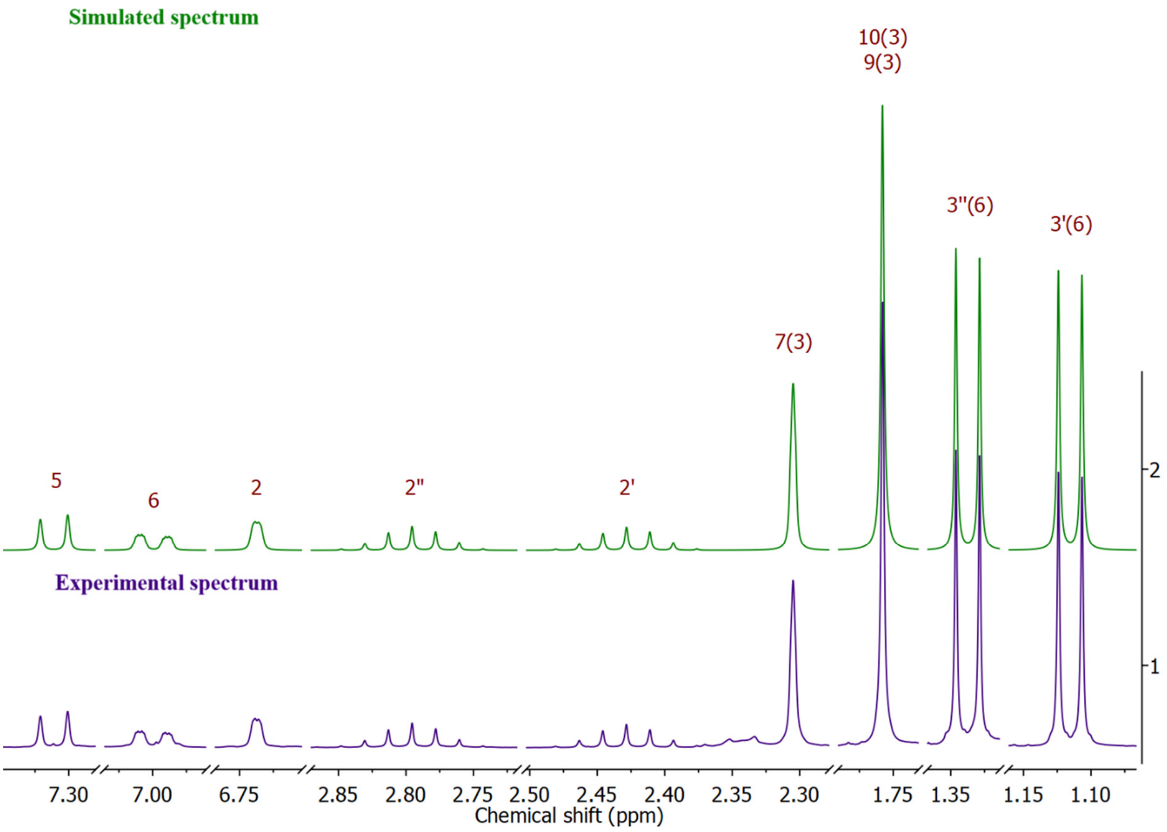

Figure S128. Simulated (manual iterative full spin, MestreNova 11.0.3) and experimental ^{1}H NMR spectrum of 8-isobutyryloxythymyl isobutyrate.

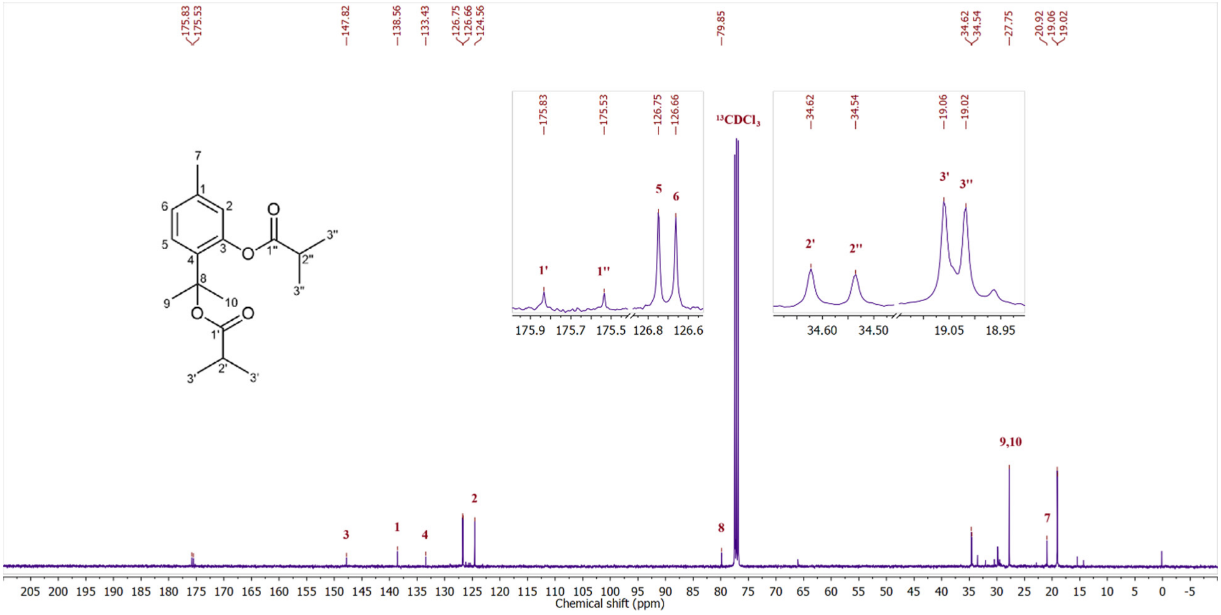

Figure S129. ^{13}C NMR (100.6 MHz, $CDCl_3$) spectrum of 8-isobutyryloxythymyl isobutyrate.

Table S22. ^{1}H (400 MHz) and ^{13}C (100.6 MHz) NMR data of 8-isobutyryloxythymyl isobutyrate (chloroform-*d*); NMR parameters are derived from manual iterative full spin analysis, along with the observed gHMBC and NOESY correlations.

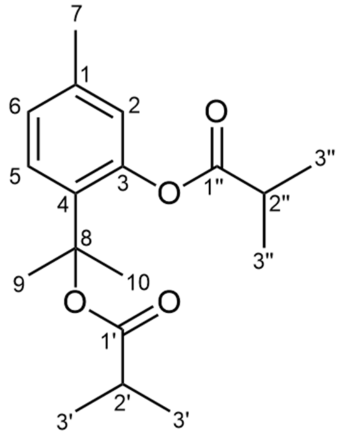

Position	δ_H (m, J (Hz), Integration)	δ_C	HMBC[a]	NOESY[b]
1	/	138.56	/	/
2	6.7372 (dqd, $^4J_{2,6}$ = 1.8, $^4J_{2,7}$ = -0.7, $^5J_{2,5}$ = 0.3, 1 H)	124.56	3,4,6,7	7
3	/	147.82	/	/
4	/	133.43	/	/
5	7.3106 (ddq, $^3J_{5,6}$ = 8.1, $^5J_{2,5}$ = 0.3, $^5J_{5,7}$ = 0.3, 1 H)	126.75	1.3,8	6,9,10
6	6.9996 (ddq, $^3J_{5,6}$ = 8.1, $^4J_{2,6}$ = 1.8, $^4J_{6,7}$ = -0.7, 1 H)	126.66	2,4,7	5,7
7	2.3049 (ddd, $^4J_{6,7}$ = -0.7, $^4J_{2,7}$ = -0.7, $^5J_{5,7}$ = 0.3, 3 H)	20.92	1,2,6	2,6
8	/	79.85	/	/
9 and 10	1.7579 (s, 6 H)	27.75	4,8,9,10	5
1′	/	175.83	/	/
2′	2.4281 (sept, $^3J_{2',3'}$ = 7.0, 1 H)	34.62	1′,3′	3′
3′	1.1155 (d, $^3J_{2',3'}$ = 7.0, 6 H)	19.06[c]	1′,2′,3′	2′
1′′	/	175.53	/	/
2′′	2.7952 (sept, $^3J_{2'',3''}$ = 7.0, 1 H)	34.54	1′′,3′′	3′′
3′′	1.3374 (d, $^3J_{2'',3''}$ = 7.0, 6 H)	19.02[c]	1′′,2′′,3′′	2′′

[a] gHMBC correlations observed between the hydrogen in this row and the carbon in the listed position.

[b] Cross-peaks observed in the NOESY spectrum.

[c] The signals of C-3′ and C-3′′ may be interchanged.

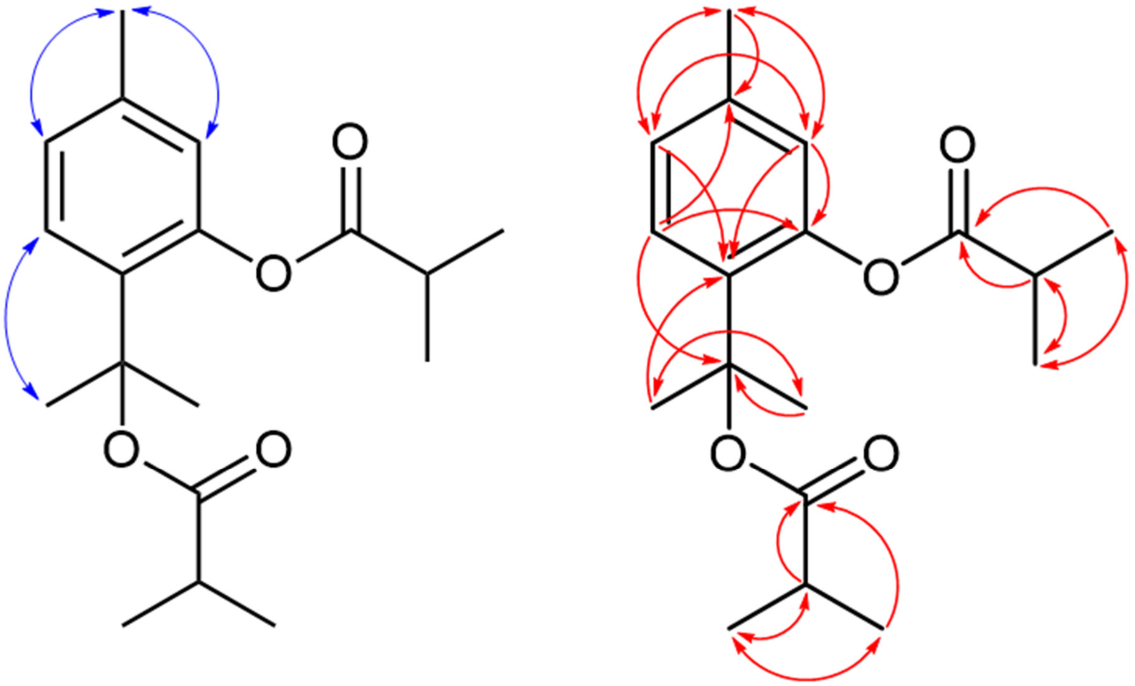

Figure S130. NOESY (blue arrows) and HMBC (red arrows) interactions of 8-isobutyryloxythymyl isobutyrate.

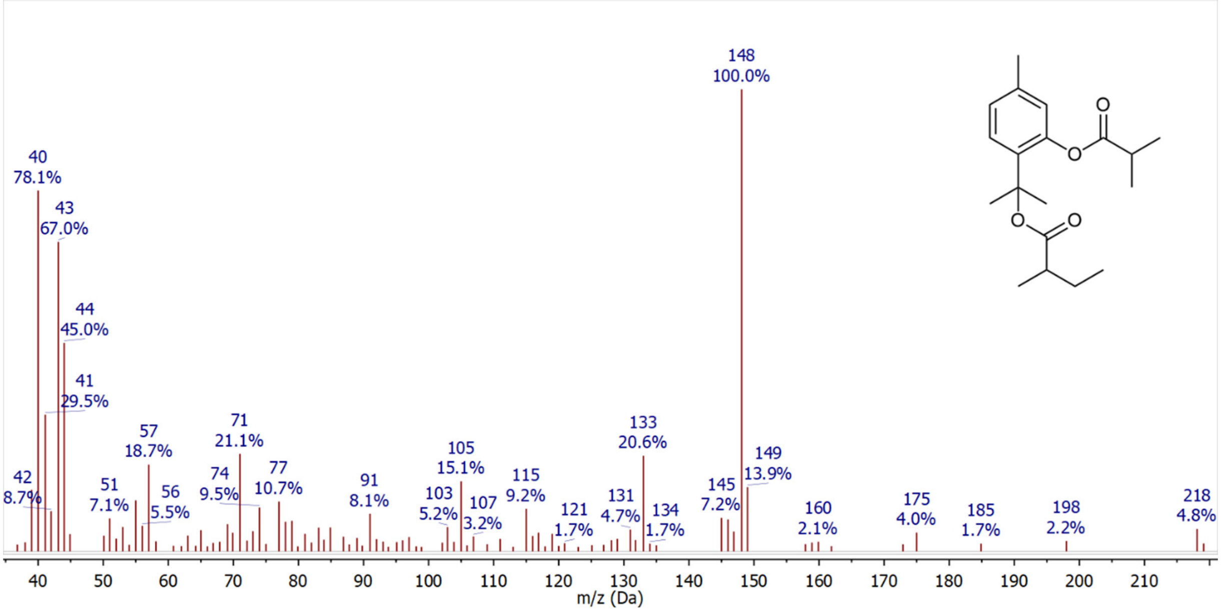

Figure S131. Mass spectrum (EI, 70 eV) of 8-(2-methylbutyryloxy)thymyl isobutyrate.

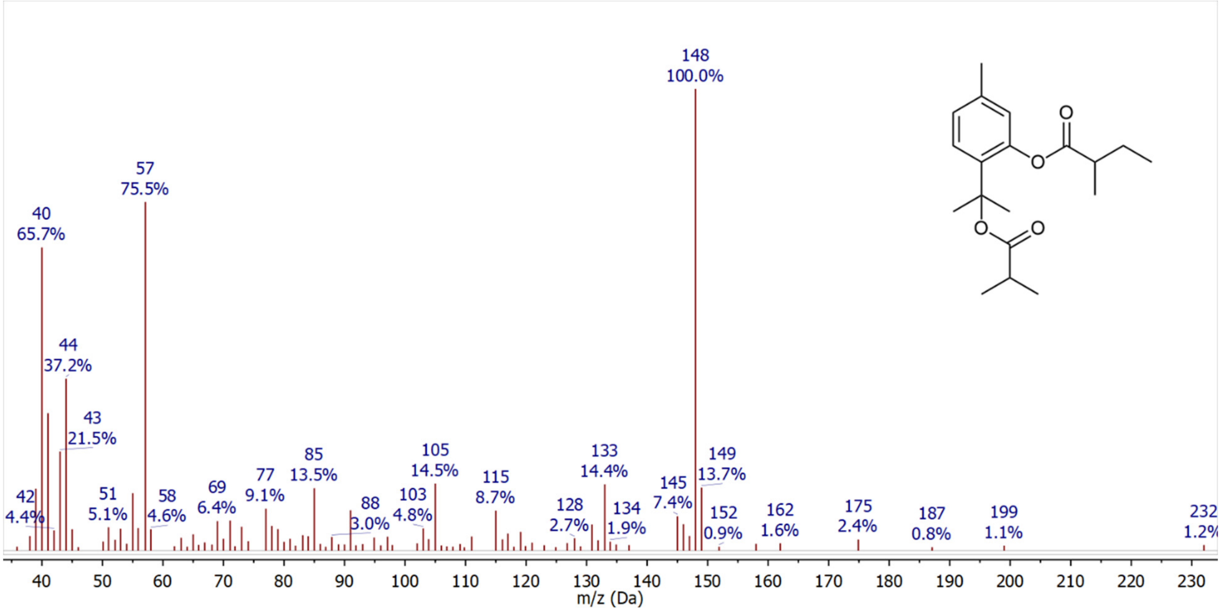

Figure S132. Mass spectrum (EI, 70 eV) of 8-isobutyryloxythymyl 2-methylbutyrate.

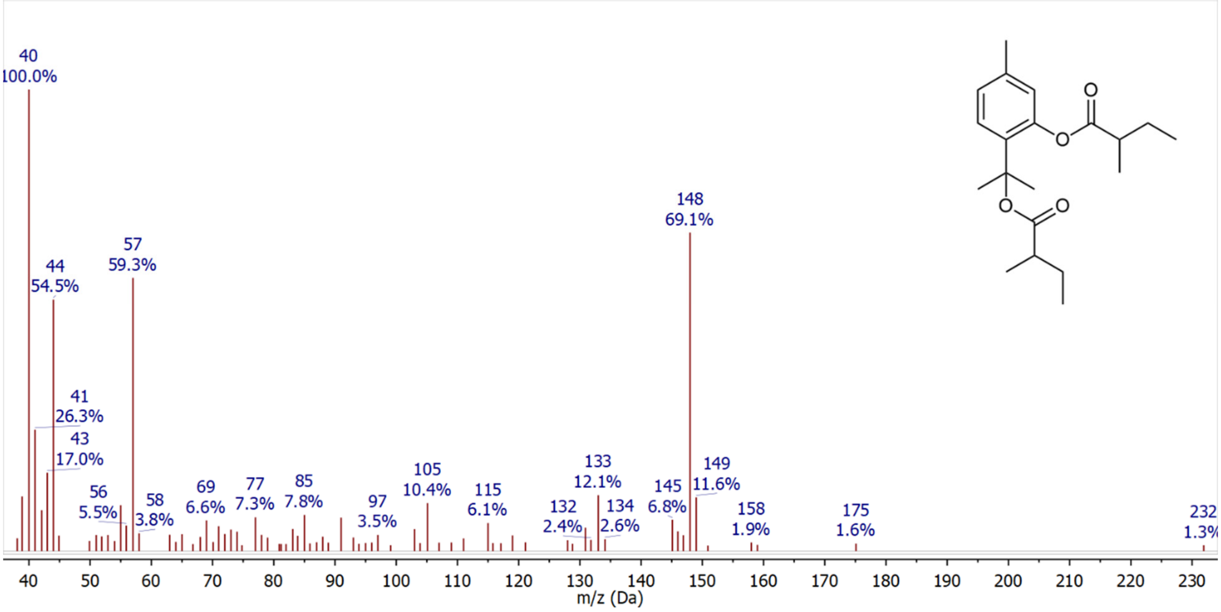

Figure S133. Mass spectrum (EI, 70 eV) of 8-(2-methylbutyryloxy)thymyl 2-methylbutyrate.

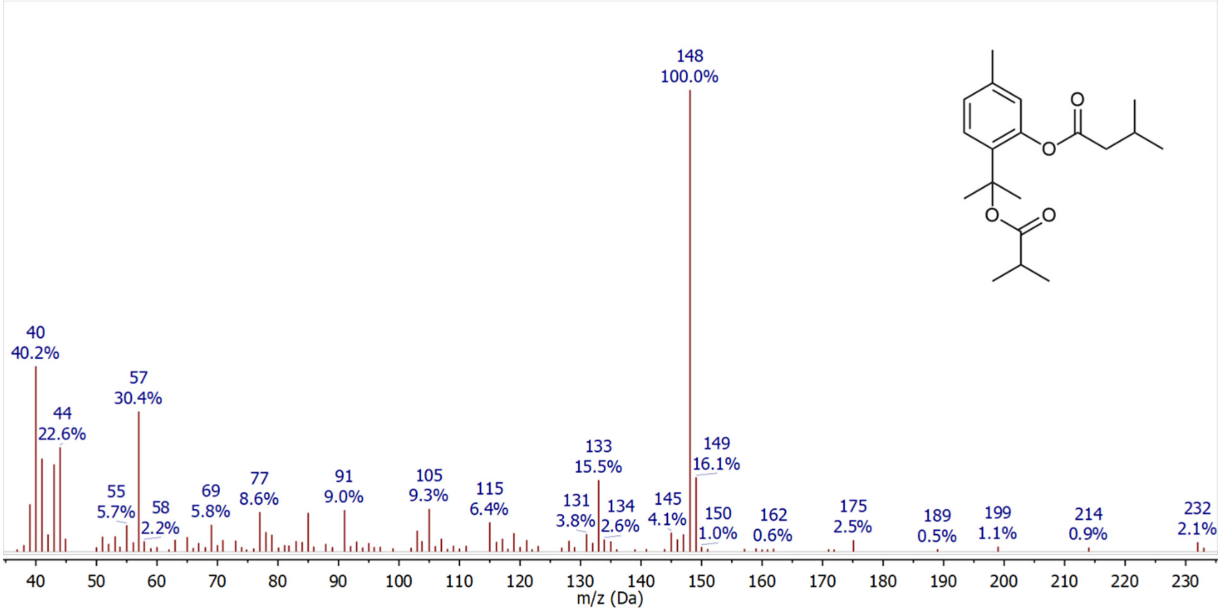

Figure S134. Mass spectrum (EI, 70 eV) of 8-isobutyryloxythymyl isovalerate.

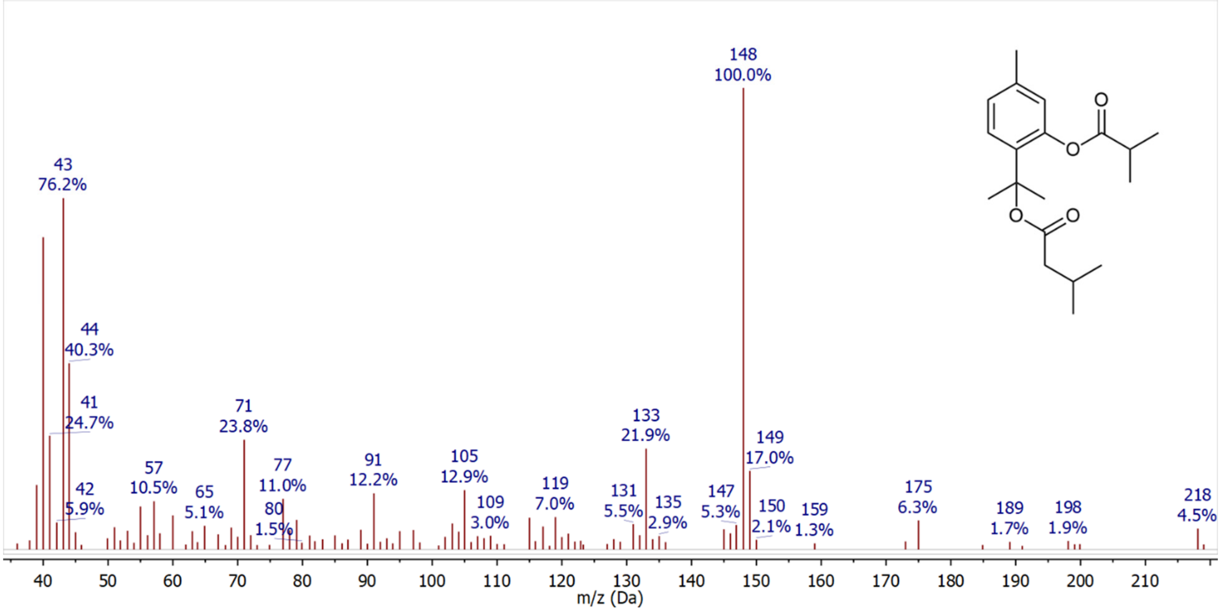

Figure S135. Mass spectrum (EI, 70 eV) of 8-isovaleroxythymyl isobutyrate.

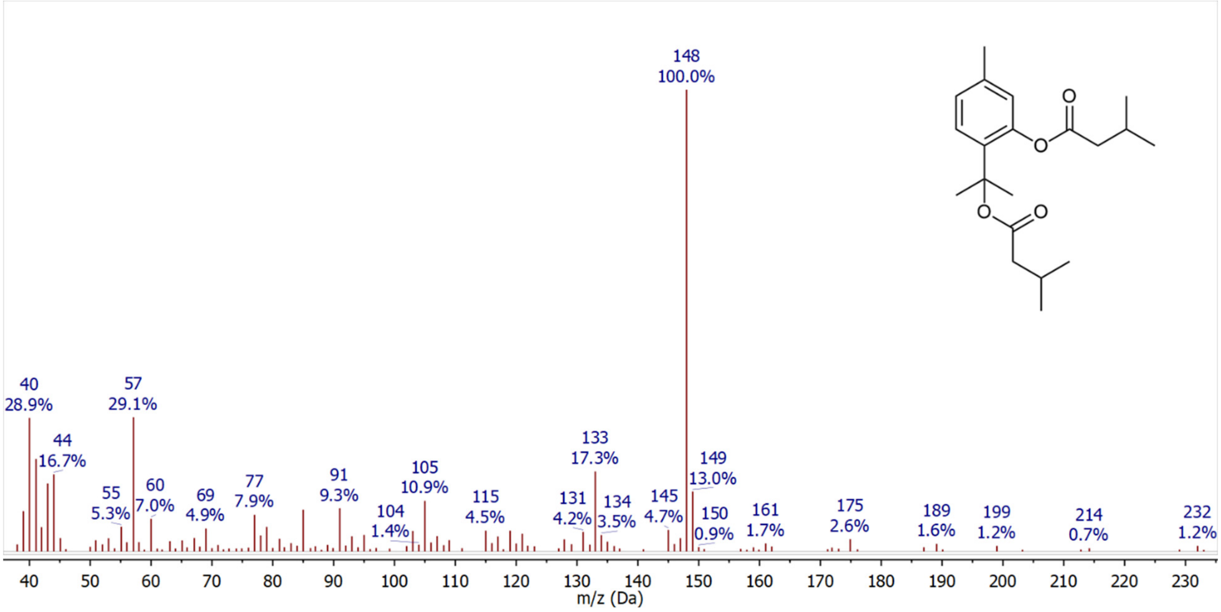

Figure S136. Mass spectrum (EI, 70 eV) of 8-isovaleroxythymyl isovalerate.

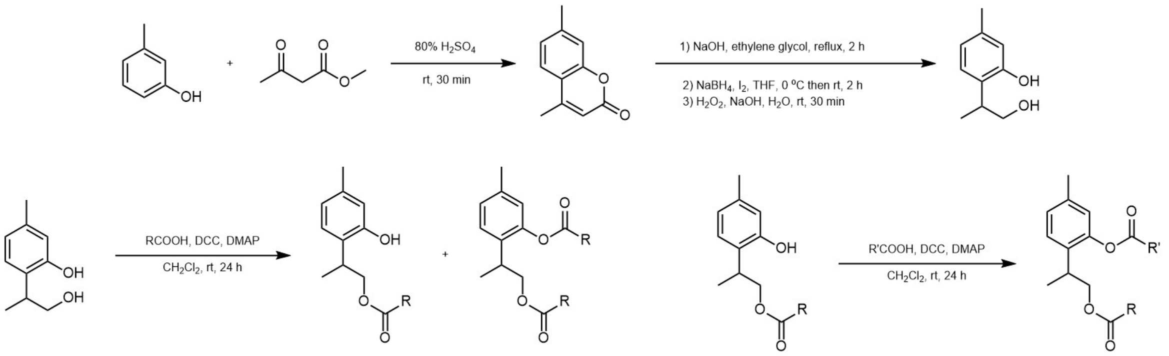

Scheme S6. Synthetic pathway to 9-hydroxythymol-derived monoesters and diesters (R,R' – isopropyl, *sec*-butyl, or isobutyl).

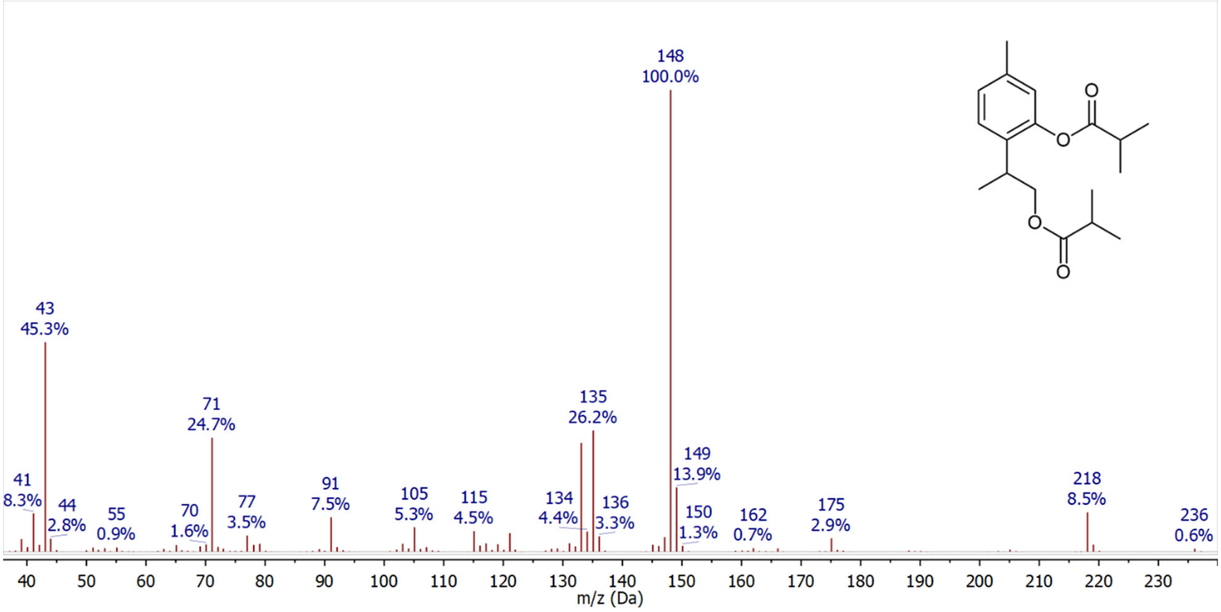

Figure S137. Mass spectrum (EI, 70 eV) of 9-isobutyryloxythymyl isobutyrate.

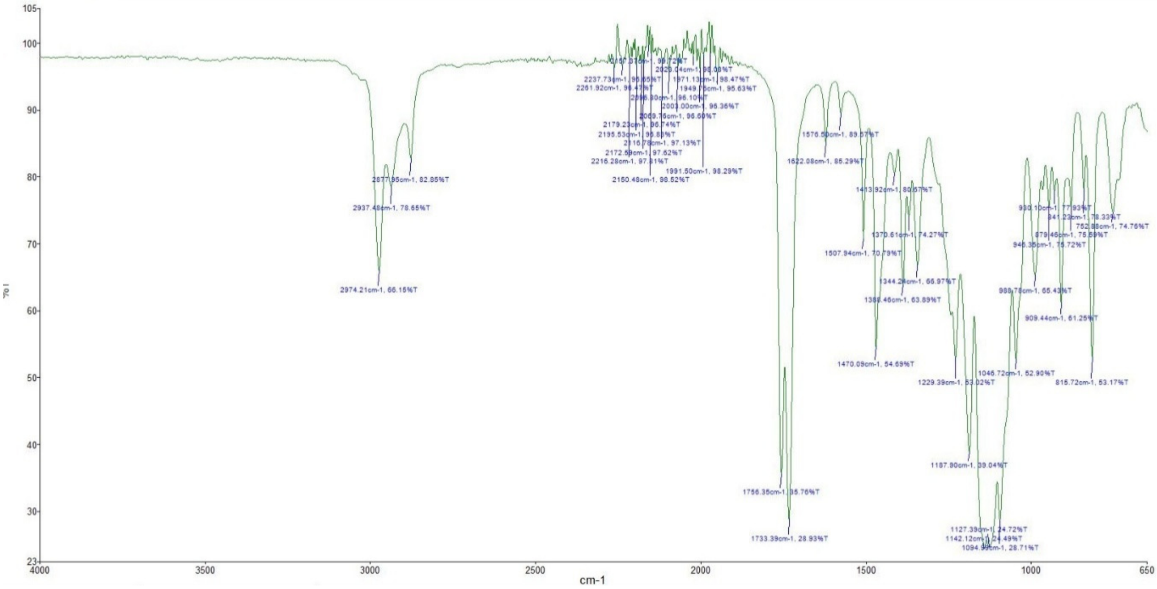

Figure S138. IR spectrum of 9-isobutyryloxythymyl isobutyrate.

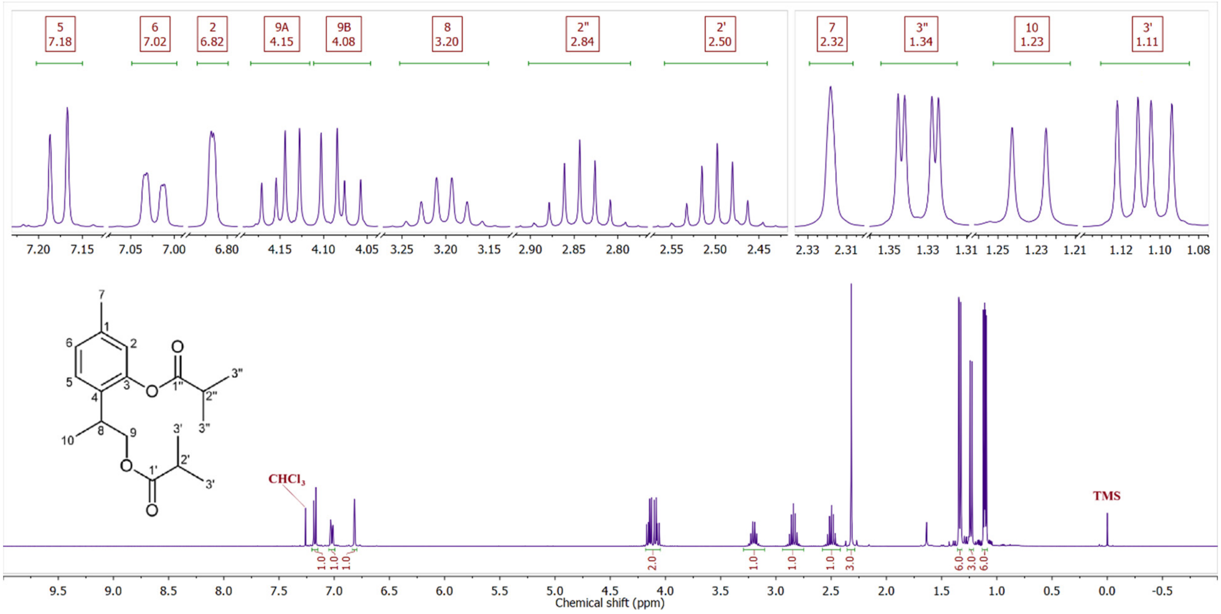

Figure S139. ^{1}H NMR (400 MHz, $CDCl_3$) spectrum of 9-isobutyryloxythymyl isobutyrate and the corresponding expansions with signal assignment.

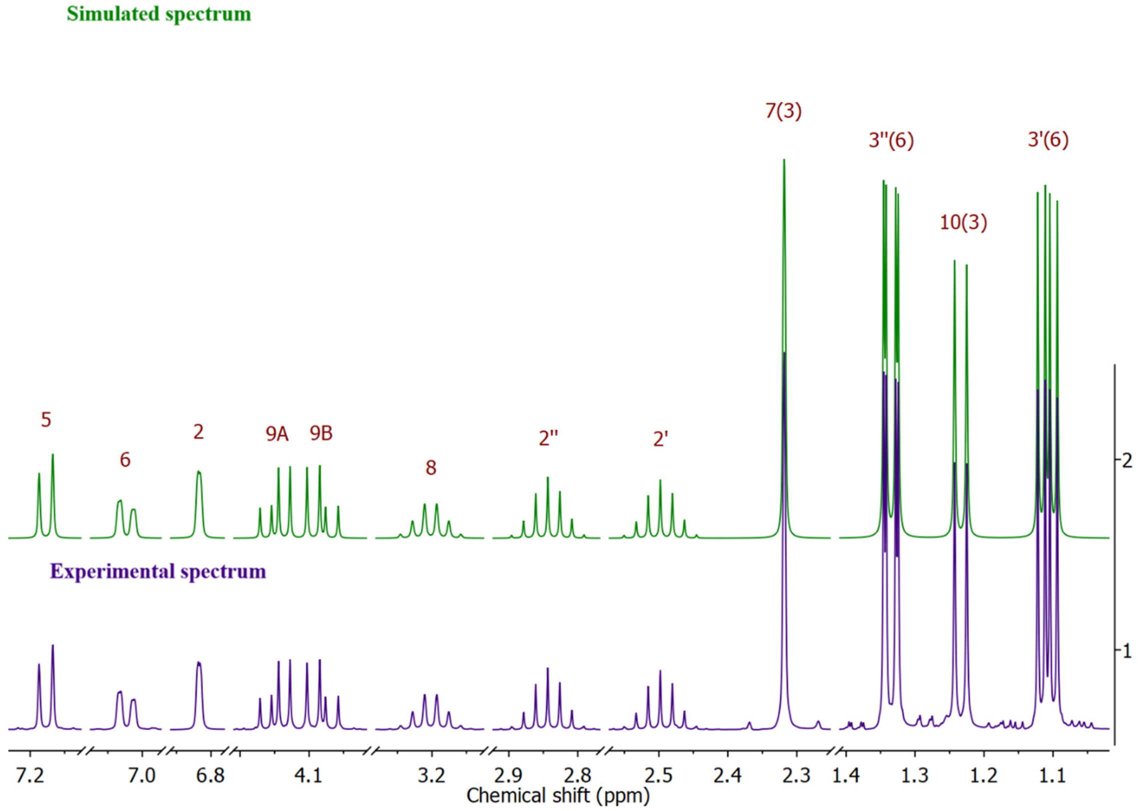

Figure S140. Simulated (manual iterative full spin, MestreNova 11.0.3) and experimental ^{1}H NMR spectrum of 9-isobutyryloxythymyl isobutyrate.

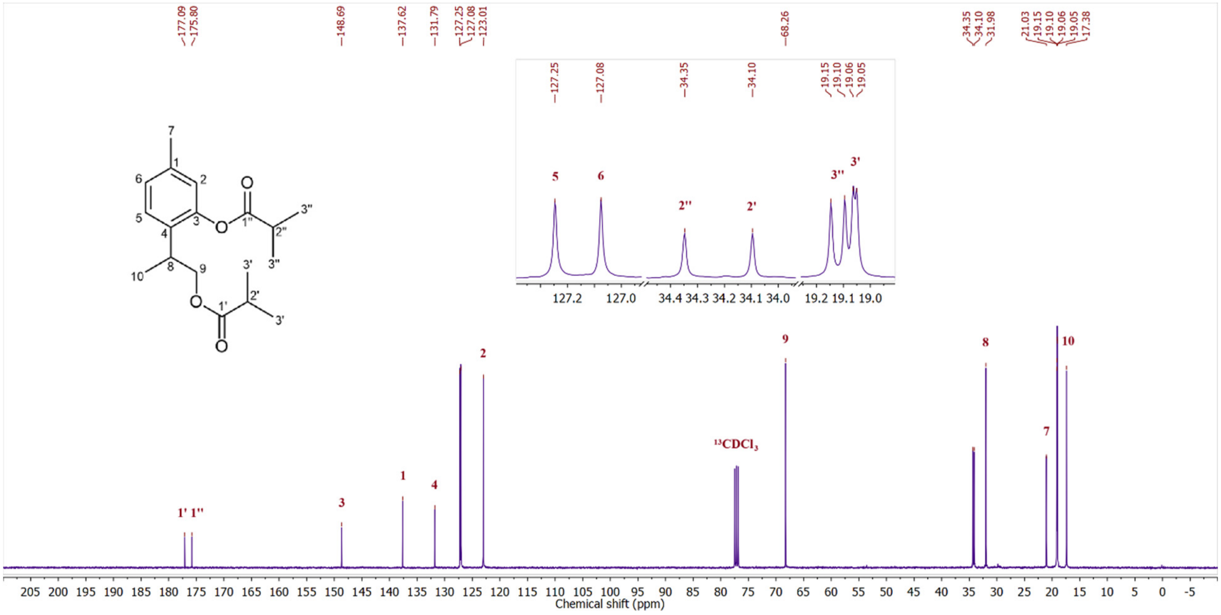

Figure S141. ^{13}C NMR (100.6 MHz, $CDCl_3$) spectrum of 9-isobutyryloxythymyl isobutyrate.

Table S23. ^{1}H (400 MHz) and ^{13}C (100.6 MHz) NMR data of 9-isobutyryloxythymyl isobutyrate (chloroform-*d*); NMR parameters are derived from manual iterative full spin analysis, along with the observed gHMBC and NOESY correlations.

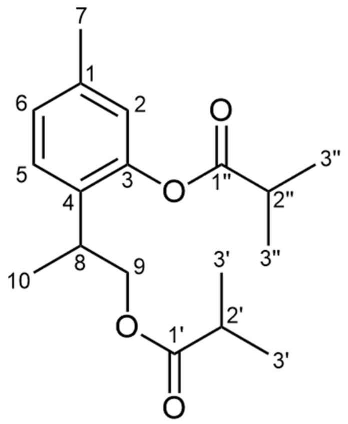

Position	δ_H (m, *J* (Hz), Integration)	δ_C	HMBC[a]	NOESY[b]
1	/	137.62	/	/
2	6.8171 (dqd, $^4J_{2,6}$ = 1.8, $^4J_{2,7}$ = -0.7, $^5J_{2,8}$ = 0.3, 1 H)	123.01	3,4,6,7	7
3	/	148.69	/	/
4	/	131.79	/	/
5	7.1765 (ddq, $^3J_{5,6}$ = 7.9, $^4J_{5,8}$ = -0.5, $^5J_{5,7}$ = 0.3, 1 H)	127.25	1.3,8	6,8,9,10
6	7.0236 (ddqd, $^3J_{5,6}$ = 7.9, $^4J_{2,6}$ = 1.8, $^4J_{6,7}$ = -0.7, $^5J_{6,8}$ = 0.4, 1 H)	127.08	2,4,7	5,7
7	2.3182 (ddd, $^4J_{2,7}$ = -0.7, $^4J_{6,7}$ = -0.7, $^5J_{5,7}$ = 0.3, 3 H)	21.03	1,2,6	2,6
8	3.2019 (dqdddd, $^3J_{8,9B}$ = 7.4, $^3J_{8,10}$ = 7.0, $^3J_{8,9A}$ = 6.6, $^4J_{5,8}$ = -0.5, $^5J_{6,8}$ = 0.4, $^5J_{2,8}$ = 0.3, 1 H)	31.98	3,4,5,9,10	5,9,10
9A	4.1465 (dd, $^2J_{9A,9B}$ = -10.7, $^3J_{8,9A}$ = 6.6, 1 H)	68.26	4,8,10,1’	5,8,10

9B	4.0830 (dd, $^{2}J_{9A,9B}$ = -10.7, $^{3}J_{8,9B}$ = 7.4, 1 H)	68.26	4,8,10,1′	5,8,10
10	1.2339 (d, $^{3}J_{8,10}$ = 7.0, 3 H)	17.38	4,8,9	5,8,9A,9B
1′	/	177.09	/	/
2′	2.4976 (sept, $^{3}J_{2′,3′}$ = 7.0, 1 H)	34.10	1′,3′	3′
3′	1.1134/1.1027 (d, $^{3}J_{2′,3′}$ = 7.0, 6 H)	19.06/19.05	1′,2′,3′	2′
1′′	/	175.80	/	/
2′′	2.8433 (sept, $^{3}J_{2′′,3′′}$ = 7.0, 1 H)	34.35	1′′,3′′	3′′
3′′	1.3368/1.3333 (d, $^{3}J_{2′′,3′′}$ = 7.0, 6 H)	19.15/19.10	1′′,2′′,3′′	2′′

[a] gHMBC correlations observed between the hydrogen in this row and the carbon in the listed position.

[b] Cross-peaks observed in the NOESY spectrum.

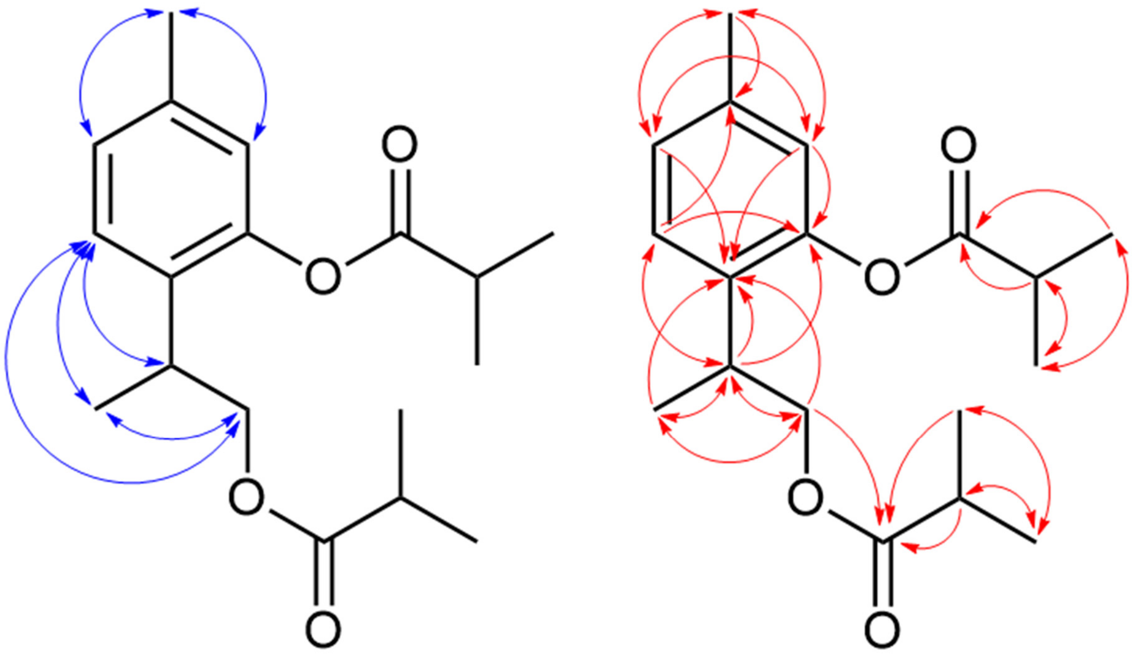

Figure S142. NOESY (blue arrows) and HMBC (red arrows) interactions of 9-isobutyryloxythymyl isobutyrate.

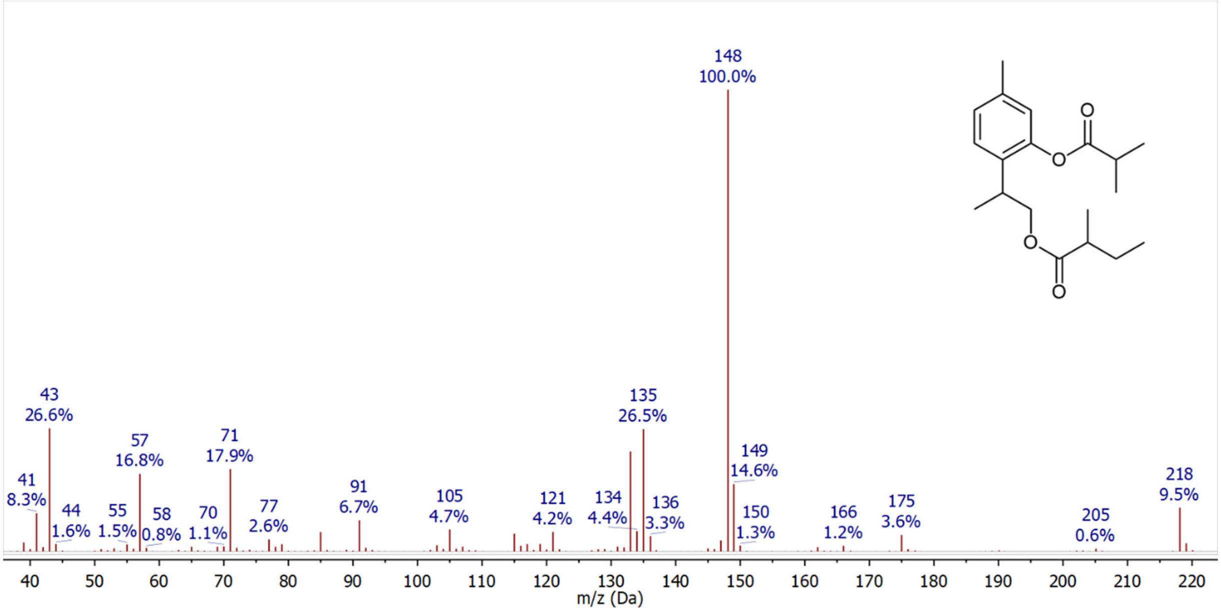

Figure S143. Mass spectrum (EI, 70 eV) of 9-(2-methylbutyryloxy)thymyl isobutyrate.

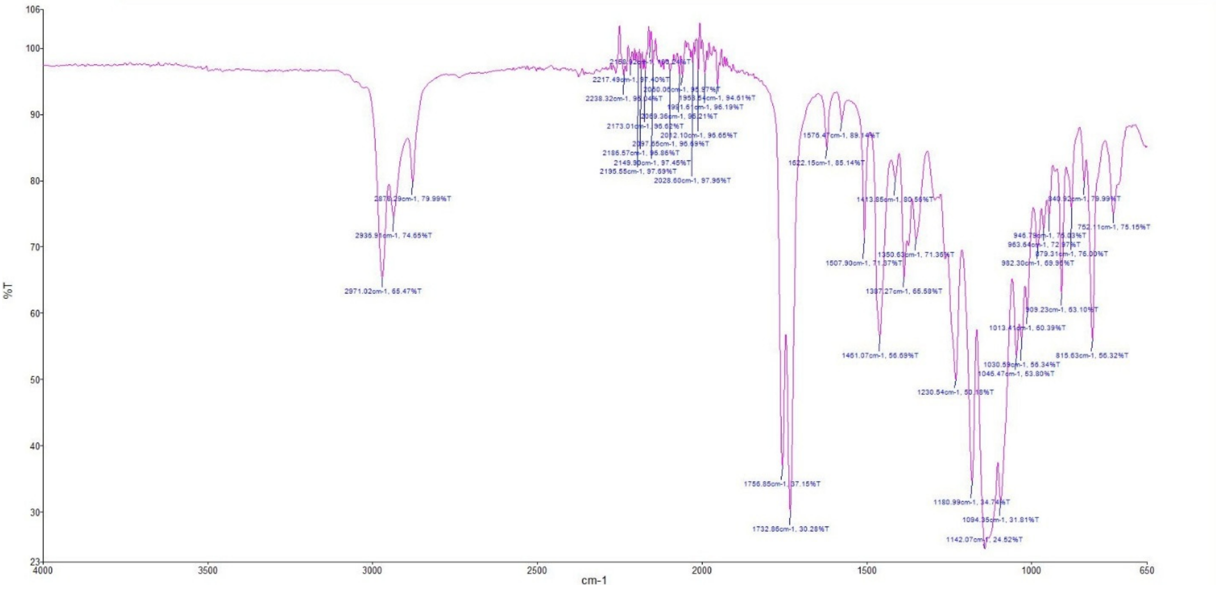

Figure S144. IR spectrum of 9-(2-methylbutyryloxy)thymyl isobutyrate.

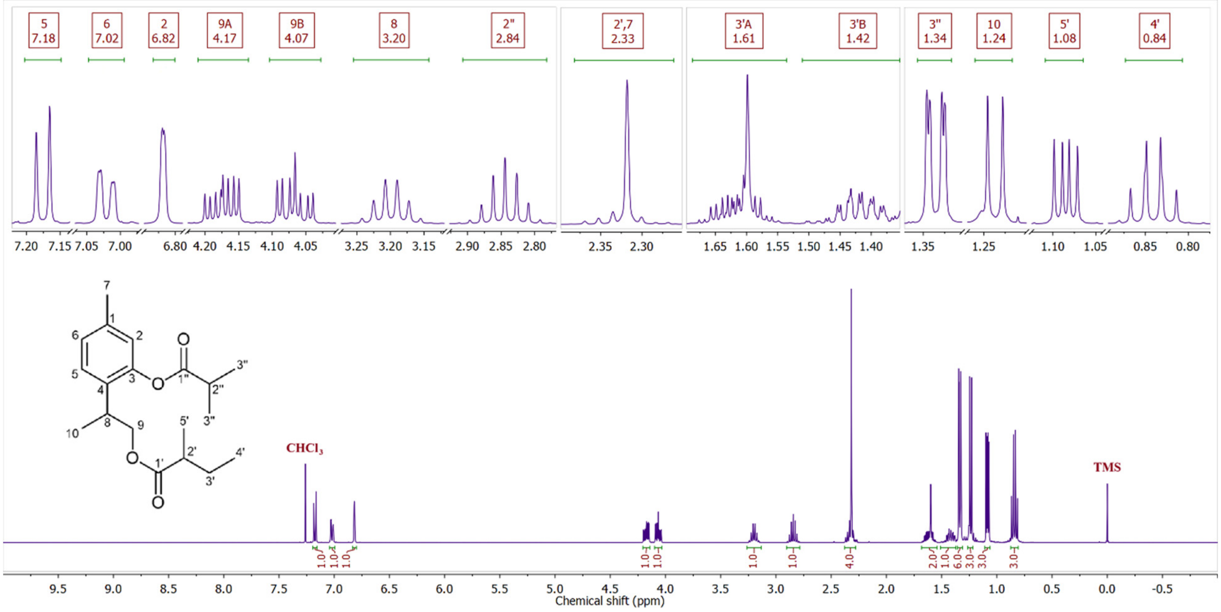

Figure S145. ^{1}H NMR (400 MHz, $CDCl_3$) spectrum of 9-(2-methylbutyryloxy)thymyl isobutyrate and the corresponding expansions with signal assignment.

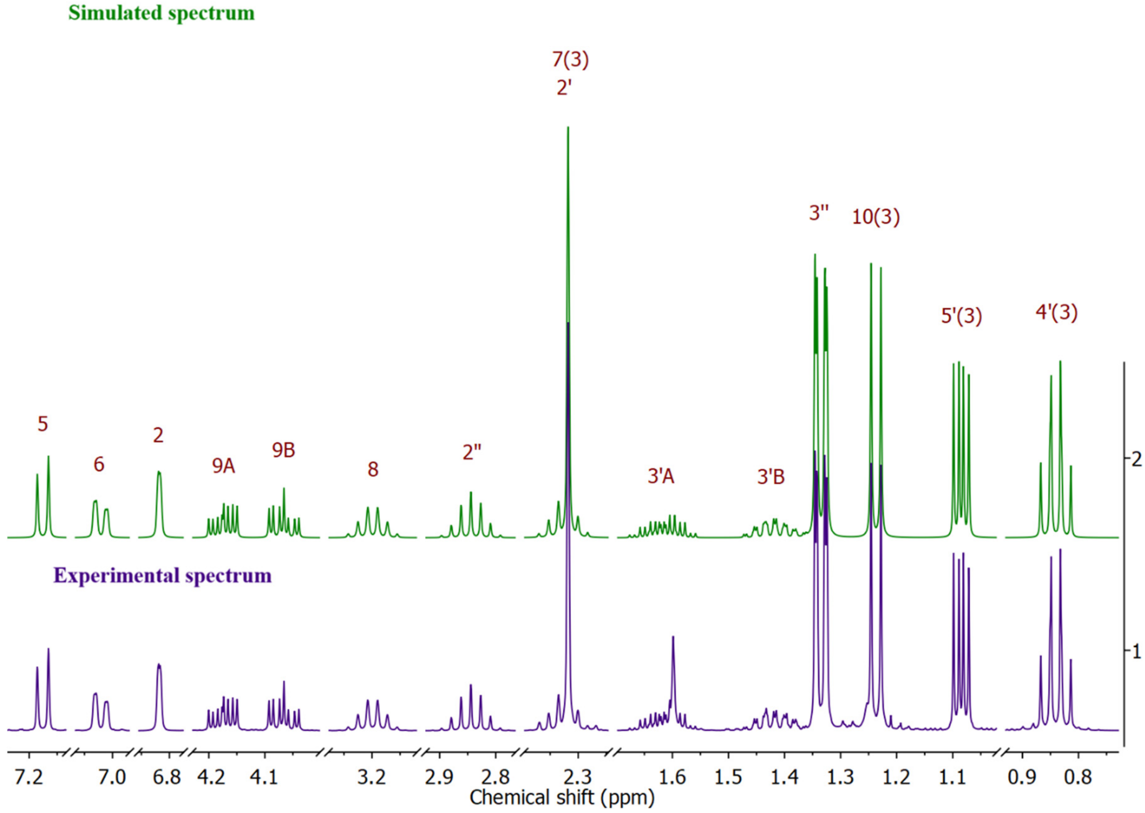

Figure S146. Simulated (manual iterative full spin, MestreNova 11.0.3) and experimental ^{1}H NMR spectrum of 9-(2-methylbutyryloxy)thymyl isobutyrate.

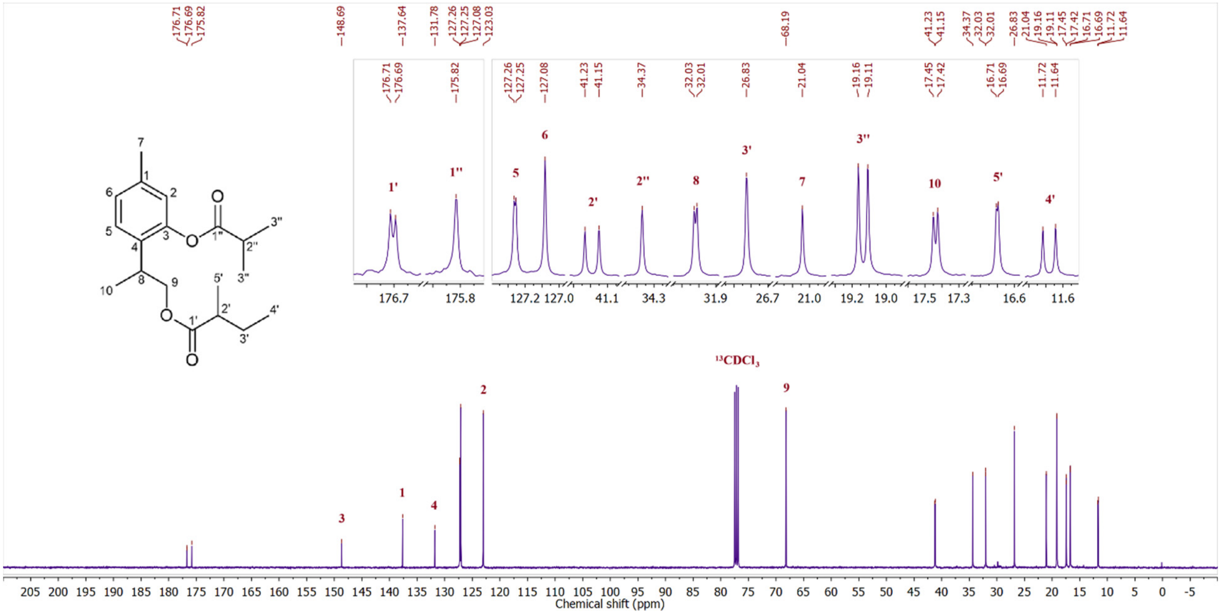

Figure S147. ^{13}C NMR (100.6 MHz, $CDCl_3$) spectrum of 9-(2-methylbutyryloxy)thymyl isobutyrate.

Table S24. ^{1}H (400 MHz) and ^{13}C (100.6 MHz) NMR data of diastereomeric mixture of 9-(2-methylbutyryloxy)thymyl isobutyrate (chloroform-*d*); NMR parameters are derived from manual iterative full spin analysis, along with the observed gHMBC and NOESY correlations.

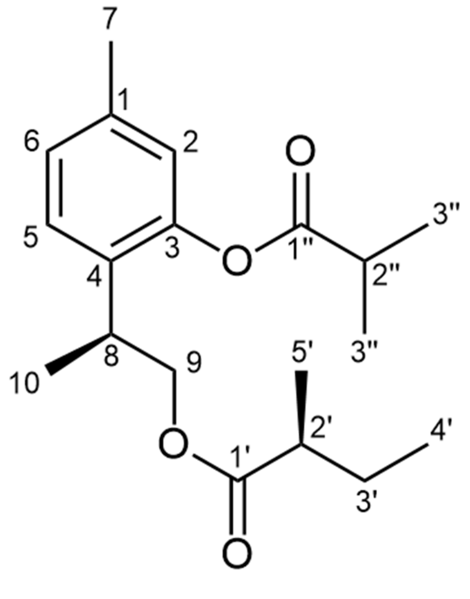

(±)

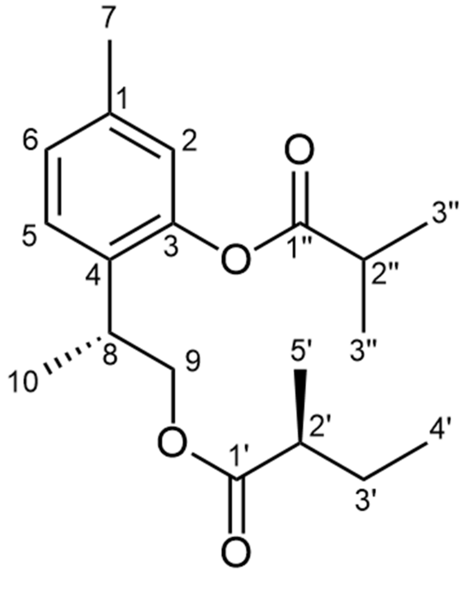

(±)

Position	δ_H (m, J (Hz), Integration)	δ_C	HMBC[a]	NOESY[b]
1	/	137.64	/	/
2	6.8181 (dqd, $^{4}J_{2,6}$ = 1.8, $^{4}J_{2,7}$ = -0.7, $^{5}J_{2,8}$ = 0.3, 1 H)	123.03	3,4,6,7	7

3	/	148.69	/	/
4	/	131.78	/	/
5	7.1748 (ddq, $^{3}J_{5,6}$ = 7.9, $^{4}J_{5,8}$ = -0.5, $^{5}J_{5,7}$ = 0.3, 1 H)	127.26/127.25	1.3,8	6,9,10
6	7.0215 (ddqd, $^{3}J_{5,6}$ = 7.9, $^{4}J_{2,6}$ = 1.8, $^{4}J_{6,7}$ = -0.7, $^{5}J_{6,8}$ = 0.4, 1 H)	127.08	2,4,7	5,7
7	2.3180 (ddd, $^{4}J_{2,7}$ = -0.7, $^{4}J_{6,7}$ = -0.7, $^{5}J_{5,7}$ = 0.3, 3 H)	21.04	1,2,6	2,6
8	3.1988 (dqdddd, $^{3}J_{8,9B}$ = 7.6, $^{3}J_{8,10}$ = 7.0, $^{3}J_{8,9A}$ = 6.4, $^{4}J_{5,8}$ = -0.5, $^{5}J_{6,8}$ = 0.4, $^{5}J_{2,8}$ = 0.3, 1 H)	32.03/32.01	3,4,5,9,10	9,10
9A	4.1770/4.1693 (dd, $^{2}J_{9A,9B}$ = -10.8, $^{3}J_{8,9A}$ = 6.4, 1 H)	68.19	4,8,10,11	5,8,10
9B	4.0713/4.0637 (dd, $^{2}J_{9A,9B}$ = -10.8, $^{3}J_{8,9B}$ = 7.6, 1 H)	68.19	4,8,10,11	5,8,10
10	1.2369 (d, $^{3}J_{8,10}$ = 7.0, 3 H)	17.45/17.42	4,8,9	5,8,9
1′	/	176.71/176.69	/	/
2′	2.3262 (dqdq, $^{3}J_{2',3'A}$ = 7.4, $^{3}J_{2',5'}$ = 7.0, $^{3}J_{2',3'B}$ = 6.5, $^{4}J_{2',4'}$ = 0.3, 1 H)	41.23/41.15	1′,3′,4′,5′	3′,4′,5′
3′A	1.6203/1.6115 (dqd, $^{2}J_{3'A,3'B}$ = -13.7, $^{3}J_{3'A,4'}$ = 7.5, $^{3}J_{2',3'A}$ = 7.4, 1 H)	26.83	1′,2′,3′,4′	2′,4′,5′
3′B	1.4206/1.4158 (dqd, $^{2}J_{3'A,3'B}$ = -13.7, $^{3}J_{3'B,4'}$ = 7.5, $^{3}J_{2',3'B}$ = 6.5, 1 H)	26.83	1′,2′,3′,4′	2′,4′,5′
4′	0.8489/0.8326 (ddd, $^{3}J_{3'A,4'}$ = 7.5, , $^{3}J_{3'B,4'}$ = 7.5, $^{4}J_{2',4'}$ = 0.3, 3 H)	11.72/11.64	2′,3′	2′,3′
5′	1.0898/1.0801 (d, $^{3}J_{2',5'}$ = 7.0, 3 H)	16.71/16.69	1′,2′,3′	2′,3′
1′′	/	175.82	/	/
2′′	2.8446/2.8433 (sept, $^{3}J_{2'',3''}$ = 7.0, 1 H)	34.37	1′′,3′′	3′′
3′′	1.3377/1.3364/1.3339/1.3326 (d, $^{3}J_{2'',3''}$ = 7.0, 6 H)	19.16/19.11	1′′,2′′,3′′	2′′

[a] gHMBC correlations observed between the hydrogen in this row and the carbon in the listed position.

[b] Cross-peaks observed in the NOESY spectrum.

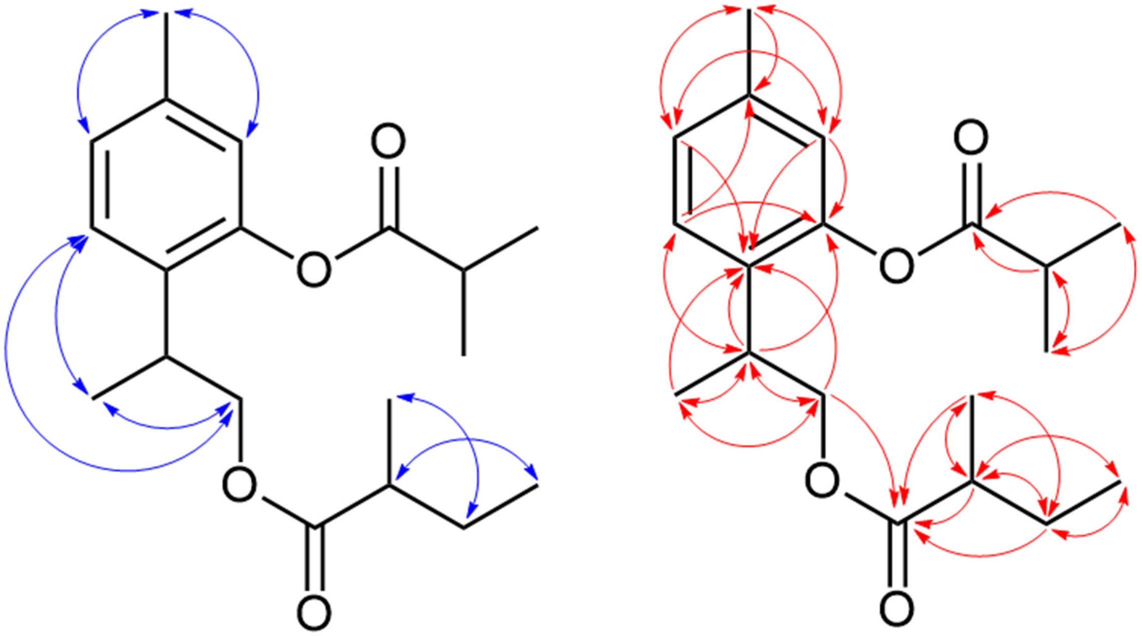

Figure S148. NOESY (blue arrows) and HMBC (red arrows) interactions of 9-(2-methylbutyryloxy)thymyl isobutyrate.

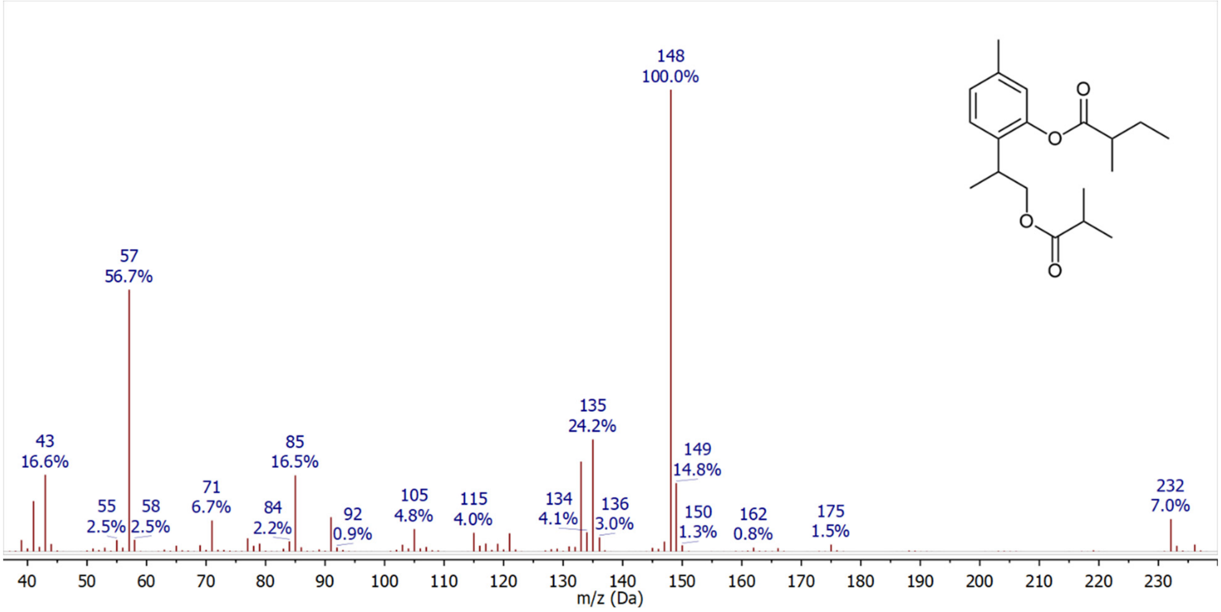

Figure S149. Mass spectrum (EI, 70 eV) of 9-isobutyryloxythymyl 2-methylbutyrate.

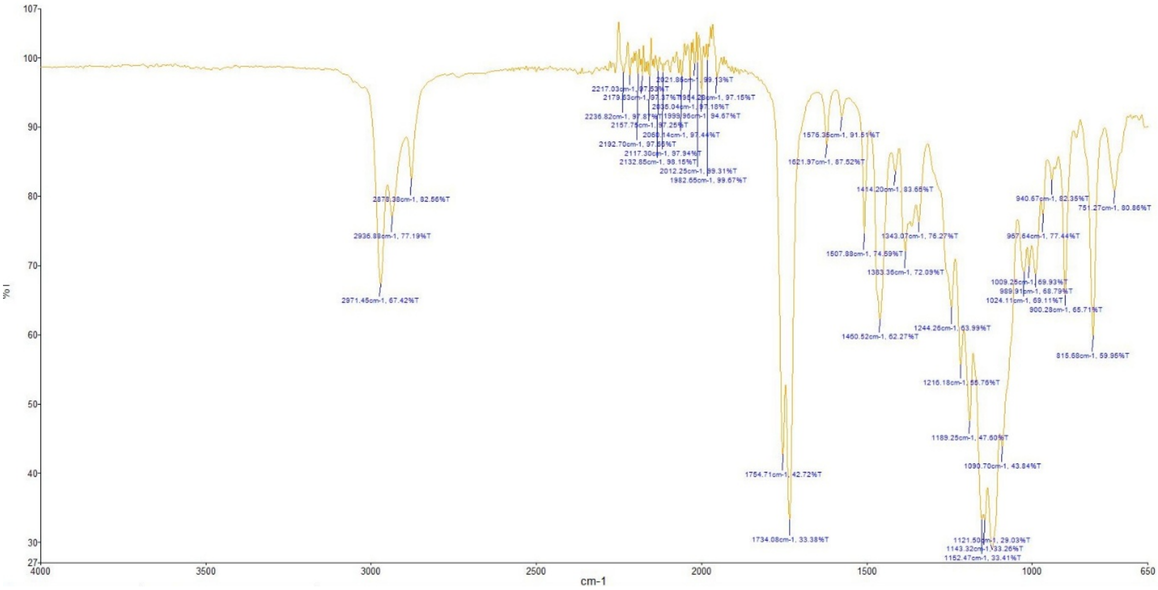

Figure S150. IR spectrum of 9-isobutyryloxythymyl 2-methylbutyrate.

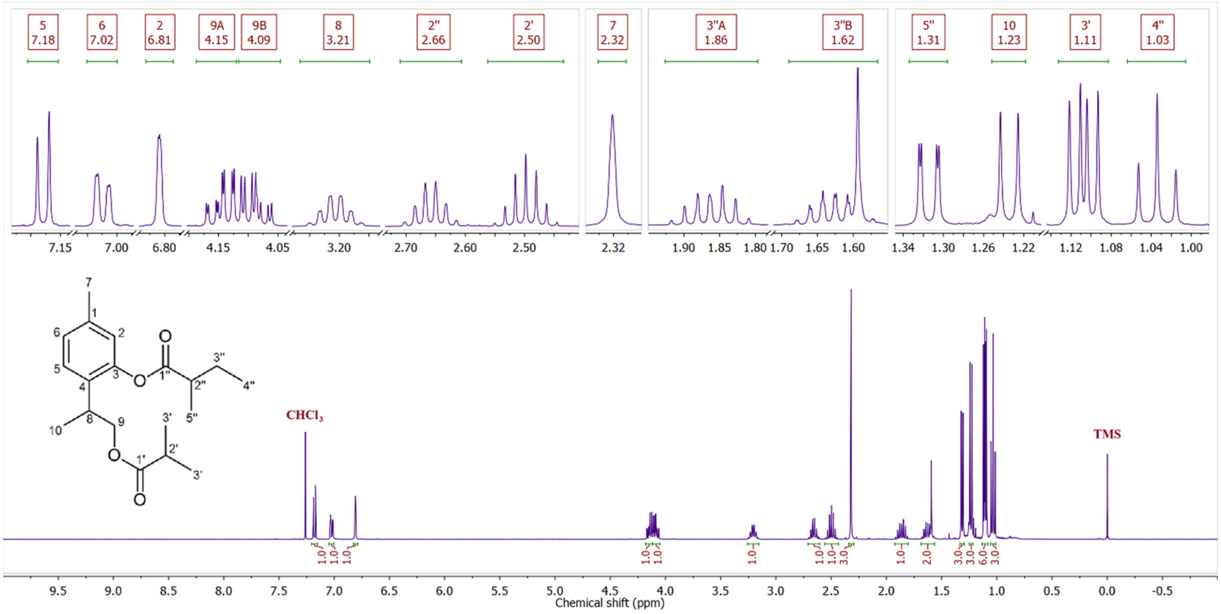

Figure S151. ^{1}H NMR (400 MHz, $CDCl_3$) spectrum of 9-isobutyryloxythymyl 2-methylbutyrate and the corresponding expansions with signal assignment.

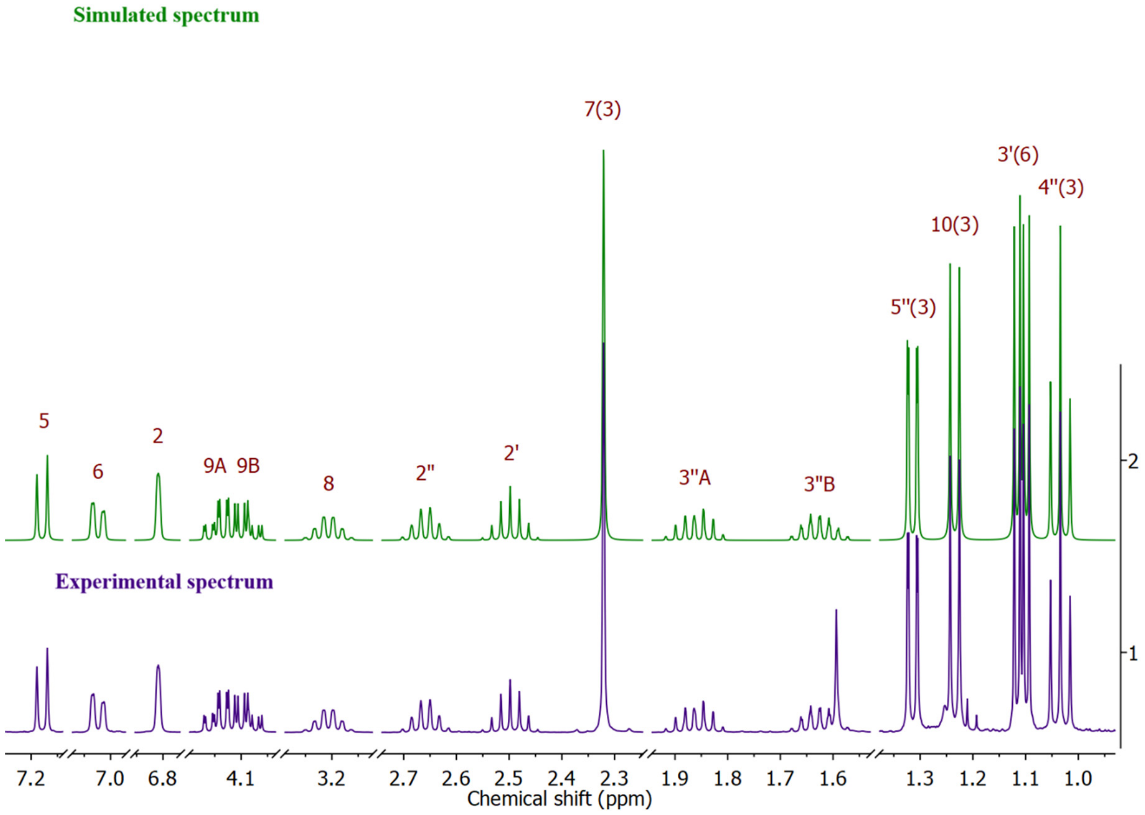

Figure S152. Simulated (manual iterative full spin, MestreNova 11.0.3) and experimental ^{1}H NMR spectrum of 9-isobutyryloxythymyl 2-methylbutyrate.

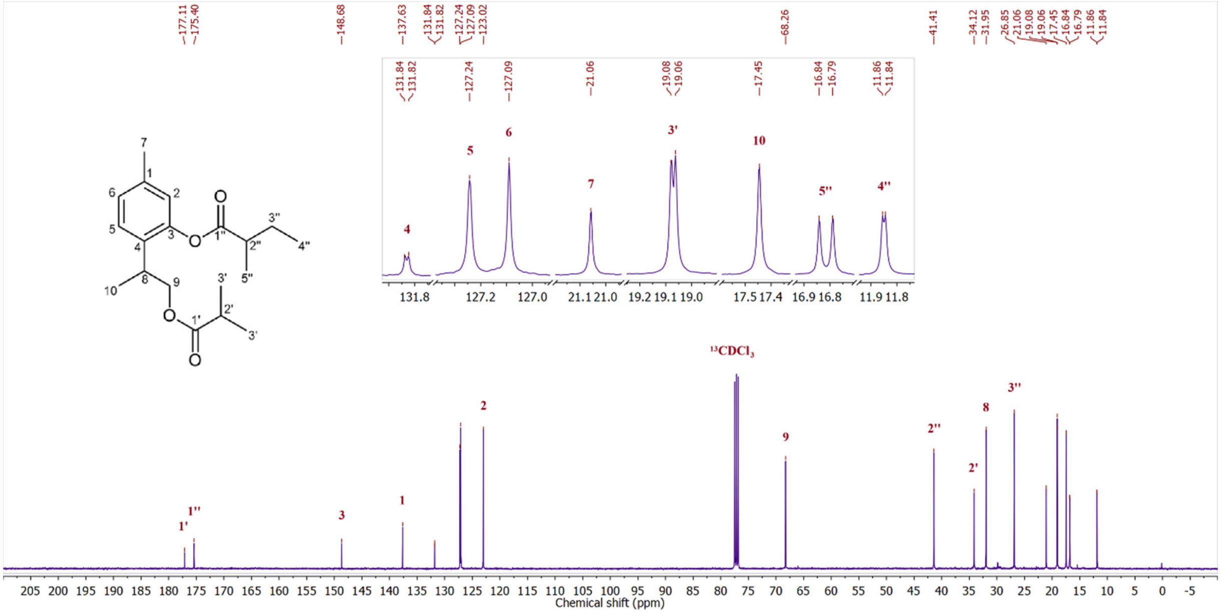

Figure S153. ^{13}C NMR (100.6 MHz, $CDCl_3$) spectrum of 9-isobutyryloxythymyl 2-methylbutyrate.

Table S25. ^{1}H (400 MHz) and ^{13}C (100.6 MHz) NMR data of diastereomeric mixture of 9-isobutyryloxythymyl 2-methylbutyrate (chloroform-*d*); NMR parameters are derived from manual iterative full spin analysis, along with the observed gHMBC and NOESY correlations.

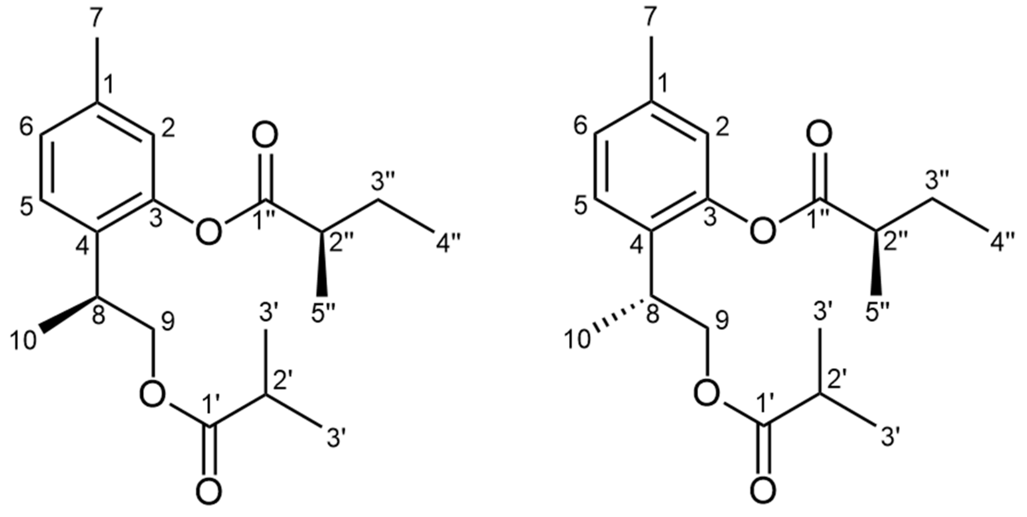

(±) (±)

Position	δ_H (m, J (Hz), Integration)	δ_C	HMBC[a]	NOESY[b]
1	/	137.63	/	/
2	6.8097/6.8079 (dqd, $^{4}J_{2,6}$ = 1.8, $^{4}J_{2,7}$ = -0.7, $^{5}J_{2,8}$ = 0.3, 1 H)	123.02	3,4,6,7	7
3	/	148.68	/	/
4	/	131.84/131.82	/	/
5	7.1785 (ddq, $^{3}J_{5,6}$ = 7.9, $^{4}J_{5,8}$ = -0.5, $^{5}J_{5,7}$ = 0.3, 1 H)	127.24	1.3,8	6,9,10

6	7.0246/7.0243 (ddqd, $^3J_{5,6}$ = 7.9, $^4J_{2,6}$ = 1.8, $^4J_{6,7}$ = -0.7, $^5J_{6,8}$ = 0.4, 1 H)	127.09	2,4,7	5,7
7	2.3207 (ddd, $^4J_{2,7}$ = -0.7, $^4J_{6,7}$ = -0.7, $^5J_{5,7}$ = 0.3, 3 H)	21.06	1,2,6	2,6
8	3.2084/3.2047 (dqdddd, $^3J_{8,9B}$ = 7.5, $^3J_{8,10}$ = 7.0, $^3J_{8,9A}$ = 6.6, $^4J_{5,8}$ = -0.5, $^5J_{6,8}$ = 0.4, $^5J_{2,8}$ = 0.3, 1 H)	31.95	3,4,5,9,10	9,10
9A	4.1456/4.1425 (dd, $^2J_{9A,9B}$ = -10.7, $^3J_{8,9A}$ = 6.6, 1 H)	68.26	4,8,10,1′	5,8,10
9B	4.0924/4.0862 (dd, $^2J_{9A,9B}$ = -10.7, $^3J_{8,9B}$ = 7.5, 1 H)	68.26	4,8,10,1′	5,8,10
10	1.2344 (d, $^3J_{8,10}$ = 7.0, 3 H)	17.45	4,8,9	5,8,9A,9B
1′	/	177.11	/	/
2′	2.4976 (sept, $^3J_{2',3'}$ = 7.0, 1 H)	34.12	1′,3′	3′
3′	1.1128/1.1019 (d, $^3J_{2',3'}$ = 7.0, 6 H)	19.08/19.06	1′,2′,3′	2′
1′′	/	175.40	/	/
2′′	2.6595/2.6570 (dqdq, $^3J_{2'',3''A}$ = 7.5, $^3J_{2'',5''}$ = 7.0, $^3J_{2'',3''B}$ = 6.5, $^4J_{2'',4''}$ = 0.3, 1 H)	41.41	1′′,3′′,4′′,5′′	3′′,4′′,5′′
3′′A	1.8625/1.8608 (ddq, $^2J_{3''A,3''B}$ = -13.7, $^3J_{2'',3''A}$ = 7.5, $^3J_{3''A,4''}$ = 7.5, 1 H)	26.85	1′′,2′′,3′′,4′′	2′′,4′′,5′′
3′′B	1.6277/1.6250 (dqd, $^2J_{3''A,3''B}$ = -13.7, $^3J_{3''B,4''}$ = 7.5, $^3J_{2'',3''B}$ = 6.5, 1 H)	26.85	1′′,2′′,3′′,4′′	2′′,4′′,5′′
4′′	1.0343 (ddd, $^3J_{3''A,4''}$ = 7.5, , $^3J_{3''B,4''}$ = 7.5, $^4J_{2'',4''}$ = 0.3, 3 H)	11.86/11.84	2′′,3′′	2′′,3′′
5′′	1.3156/1.3132 (d, $^3J_{2'',5''}$ = 7.0, 3 H)	16.84/16.79	1′′,2′′,3′′	2′′,3′′

[a] gHMBC correlations observed between the hydrogen in this row and the carbon in the listed position.

[b] Cross-peaks observed in the NOESY spectrum.

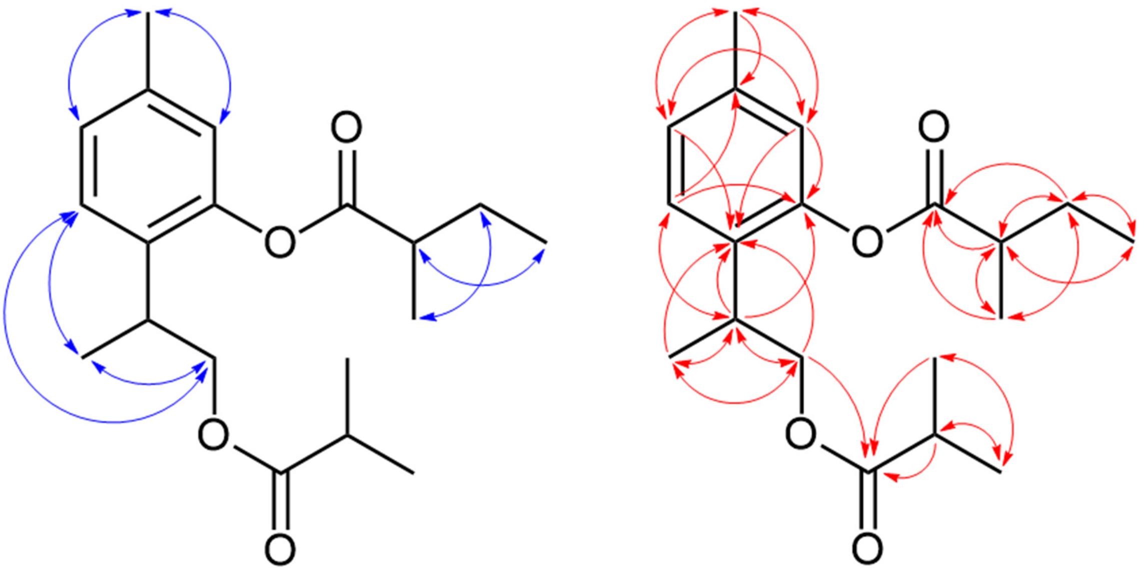

Figure S154. NOESY (blue arrows) and HMBC (red arrows) interactions of 9-isobutyryloxythymyl 2-methylbutyrate.

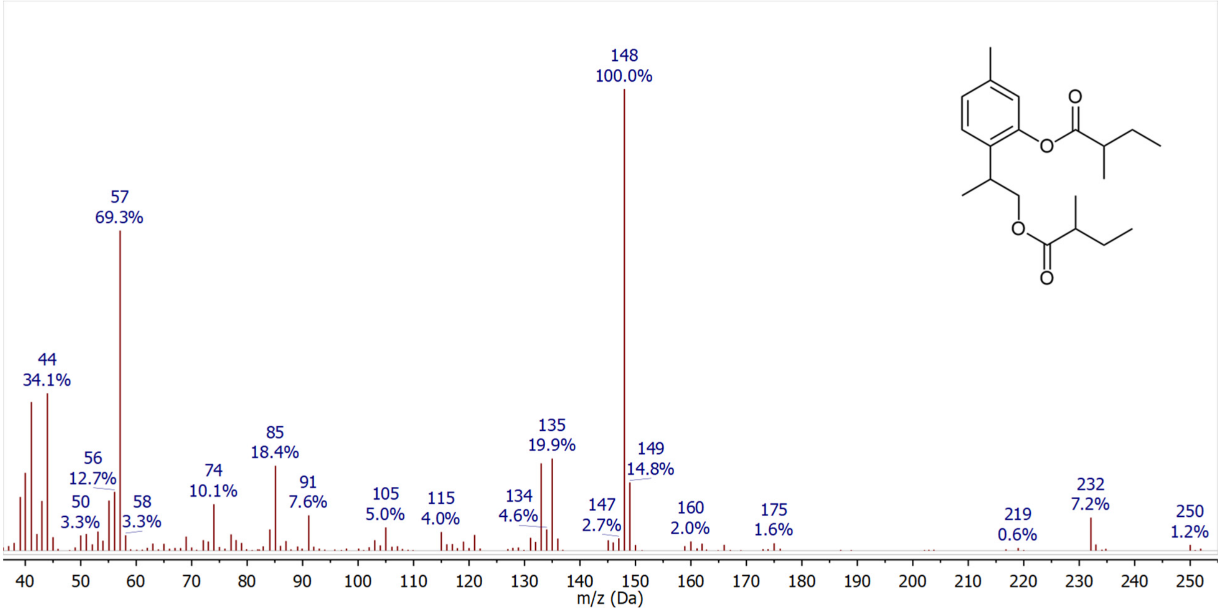

Figure S155. Mass spectrum (EI, 70 eV) of 9-(2-methylbutyryloxy)thymyl 2-methylbutyrate.

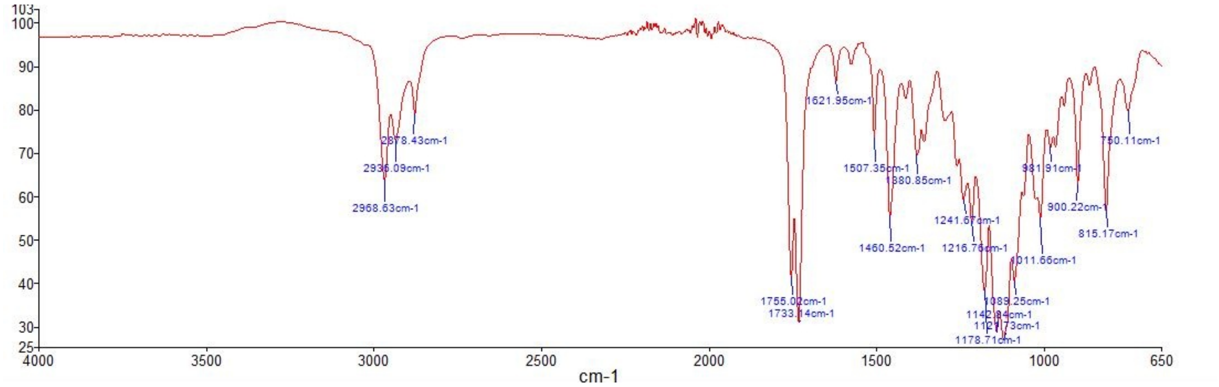

Figure S156. IR spectrum of 9-(2-methylbutyryloxy)thymyl 2-methylbutyrate.

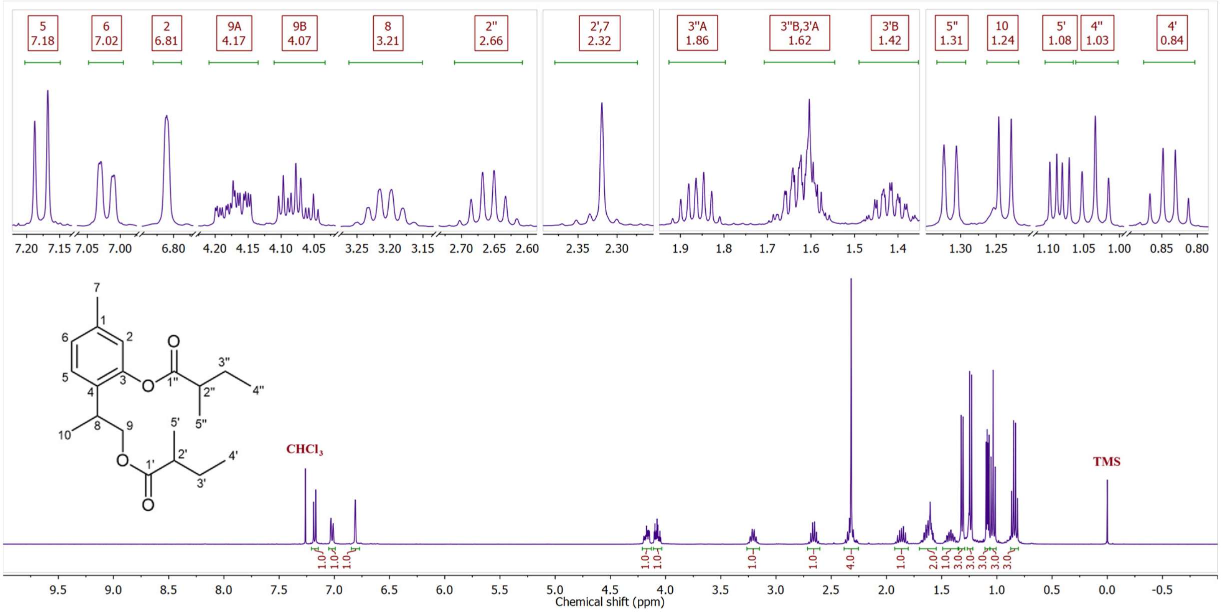

Figure S157. ^{1}H NMR (400 MHz, $CDCl_3$) spectrum of 9-(2-methylbutyryloxy)thymyl 2-methylbutyrate and the corresponding expansions with signal assignment.

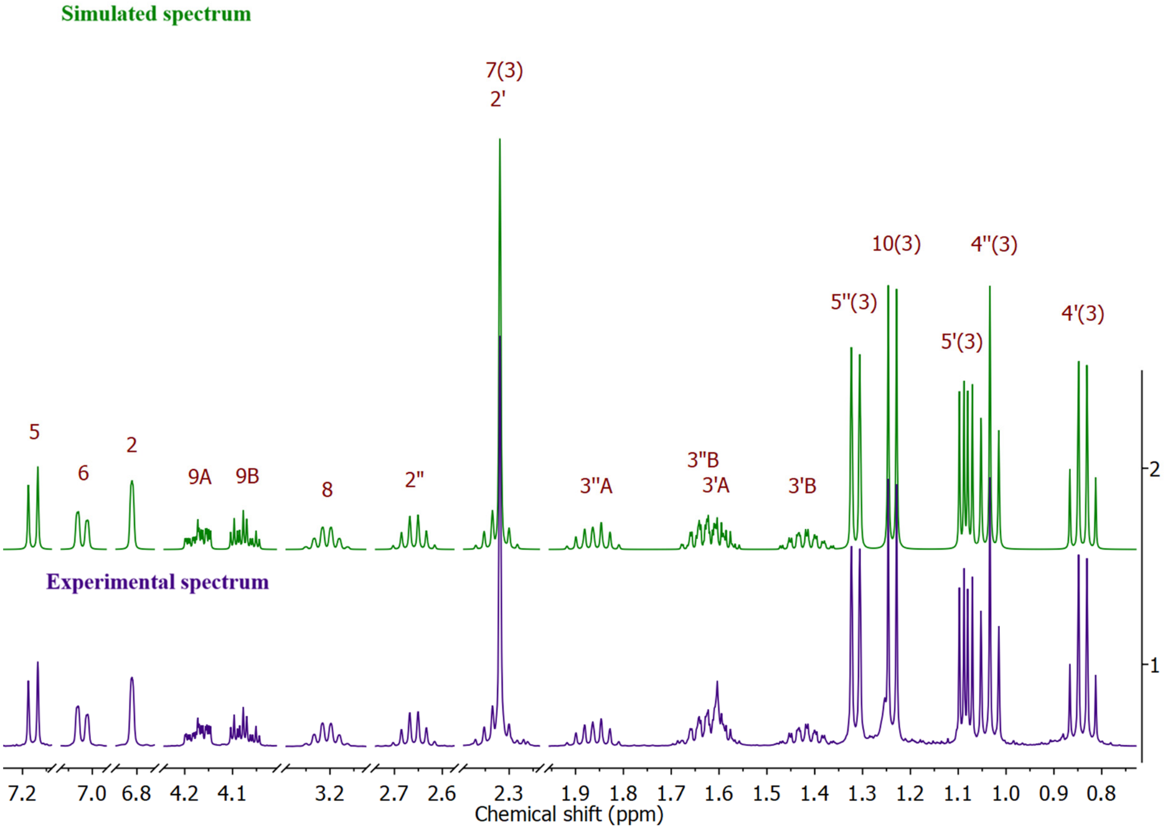

Figure S158. Simulated (manual iterative full spin, MestreNova 11.0.3) and experimental ^{1}H NMR spectrum of 9-(2-methylbutyryloxy)thymyl 2-methylbutyrate.

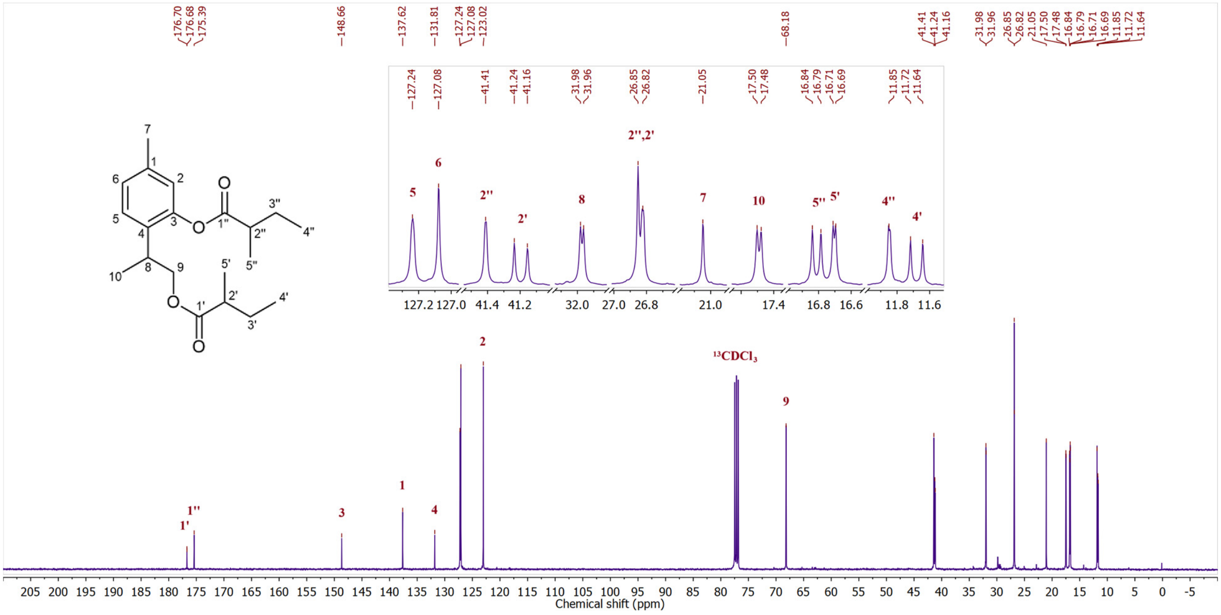

Figure S159. ^{13}C NMR (100.6 MHz, $CDCl_3$) spectrum of 9-(2-methylbutyryloxy)thymyl 2-methylbutyrate.

Table S26. ^{1}H (400 MHz) and ^{13}C (100.6 MHz) NMR data of diastereomeric mixture of 9-(2-methylbutyryloxy)thymyl 2-methylbutyrate (chloroform-d); NMR parameters are derived from manual iterative full spin analysis, along with the observed gHMBC and NOESY correlations.

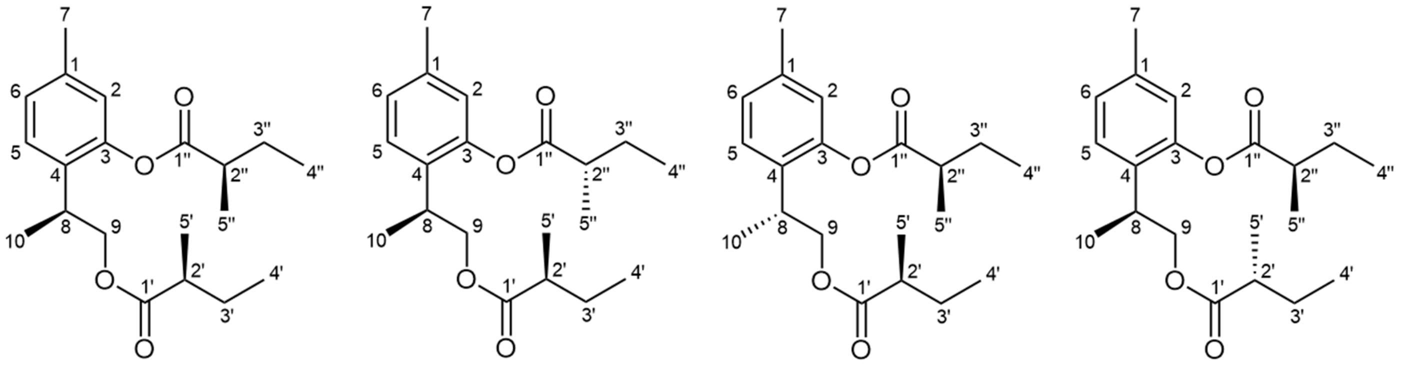

(±) (±) (±) (±)

Position	δ_H (m, J (Hz), Integration)	δ_C	HMBC[a]	NOESY[b]
1	/	137.62	/	/
2	6.8111/6.8095 (dqd, $^4J_{2,6}$ = 1.8, $^4J_{2,7}$ = -0.7, $^5J_{2,8}$ = 0.3, 1 H)	123.02	3,4,6,7	7
3	/	148.66	/	/
4	/	131.81	/	/
5	7.1772 (ddq, $^3J_{5,6}$ = 7.9, $^4J_{5,8}$ = -0.5, $^5J_{5,7}$ = 0.3, 1 H)	127.24	1.3,8	6,8,9,10
6	7.0216/7.0215/7.0215 (ddqd, $^3J_{5,6}$ = 7.9, $^4J_{2,6}$ = 1.8, $^4J_{6,7}$ = -0.7, $^5J_{6,8}$ = 0.4, 1 H)	127.08	2,4,7	5,7
7	2.3195 (ddd, $^4J_{2,7}$ = -0.7, $^4J_{6,7}$ = -0.7, $^5J_{5,7}$ = 0.3, 3 H)	21.05	1,2,6	2,6
8	3.2098/3.2080/3.2056/3.2040 (dqdddd, $^3J_{8,9B}$ = 7.6, $^3J_{8,10}$ = 7.0, $^3J_{8,9A}$ = 6.4, $^4J_{5,8}$ = -0.5, $^5J_{6,8}$ = 0.4, $^5J_{2,8}$ = 0.3, 1 H)	31.98/31.96	3,4,5,9,10	5,9,10

9A	4.1760/4.1734/4.1692/4.1658 (dd, $^{2}J_{9A,9B}$ = -10.7, $^{3}J_{8,9A}$ = 6.4, 1 H)	68.18	4,8,10,1′	5,8,10
9B	4.0828/4.0756/4.0754/4.0685 (dd, $^{2}J_{9A,9B}$ = -10.7, $^{3}J_{8,9B}$ = 7.6, 1 H)	68.18	4,8,10,1′	5,8,10
10	1.2377 (d, $^{3}J_{8,10}$ = 7.0, 3 H)	17.50/17.48	4,8,9	5,8,9
1′	/	176.70/176.68	/	/
2′	2.3270/2.3250 (dqdq, $^{3}J_{2',3'A}$ = 7.4, $^{3}J_{2',5'}$ = 7.0, $^{3}J_{2',3'B}$ = 6.4, $^{4}J_{2',4'}$ = 0.3, 1 H)	41.24/41.16	1′,3′,4′,5′	3′,4′,5′
3′A	1.6192/1.6105 (dqd, $^{2}J_{3'A,3'B}$ = -13.7, $^{3}J_{3'A,4'}$ = 7.5, $^{3}J_{2',3'A}$ = 7.4, 1 H)	26.82[c]	1′,2′,3′,4′	2′,4′,5′
3′B	1.4205/1.4200/1.4154/1.4152 (dqd, $^{2}J_{3'A,3'B}$ = -13.7, $^{3}J_{3'B,4'}$ = 7.5, $^{3}J_{2',3'B}$ = 6.4, 1 H)	26.82[c]	1′,2′,3′,4′	2′,4′,5′
4′	0.8483/0.8315 (ddd, $^{3}J_{3'A,4'}$ = 7.5, $^{3}J_{3'B,4'}$ = 7.5, $^{4}J_{2',4'}$ = 0.3, 3 H)	11.72/11.64	2′,3′	2′,3′
5′	1.0890/1.0793 (d, $^{3}J_{2',5'}$ = 7.0, 3 H)	16.71/16.69	1′,2′,3′	2′,3′
1′′	/	175.39	/	/
2′′	2.6596/2.6584 (dqdq, $^{3}J_{2'',3''A}$ = 7.4, $^{3}J_{2'',5''}$ = 7.0, $^{3}J_{2'',3''B}$ = 6.4, $^{4}J_{2'',4''}$ = 0.3, 1 H)	41.41	1′′,3′′,4′′,5′′	3′′,4′′,5′′
3′′A	1.8635/1.8627/1.8617/1.8609 (dqd, $^{2}J_{3''A,3''B}$ = -13.7, $^{3}J_{3''A,4''}$ = 7.5, $^{3}J_{2'',3''A}$ = 7.4, 1 H)	26.85[c]	1′′,2′′,3′′,4′′	2′′,4′′,5′′
3′′B	1.6275/1.6265/1.6260/1.6240 (dqd, $^{2}J_{3''A,3''B}$ = -13.7, $^{3}J_{3''B,4''}$ = 7.5, $^{3}J_{2'',3''B}$ = 6.4, 1 H)	26.85[c]	1′′,2′′,3′′,4′′	2′′,4′′,5′′
4′′	1.0341 (ddd, $^{3}J_{3''A,4''}$ = 7.5, $^{3}J_{3''B,4''}$ = 7.5, $^{4}J_{2'',4''}$ = 0.3, 3 H)	11.85	2′′,3′′	2′′,3′′
5′′	1.3163/1.3147/1.3127 (d, $^{3}J_{2'',5''}$ = 7.0, 3 H)	16.84/16.79	1′′,2′′,3′′	2′′,3′′

[a] gHMBC correlations observed between the hydrogen in this row and the carbon in the listed position.

[b] Cross-peaks observed in the NOESY spectrum.

[c] The signals of C-3′ and C-3′′ may be interchanged.

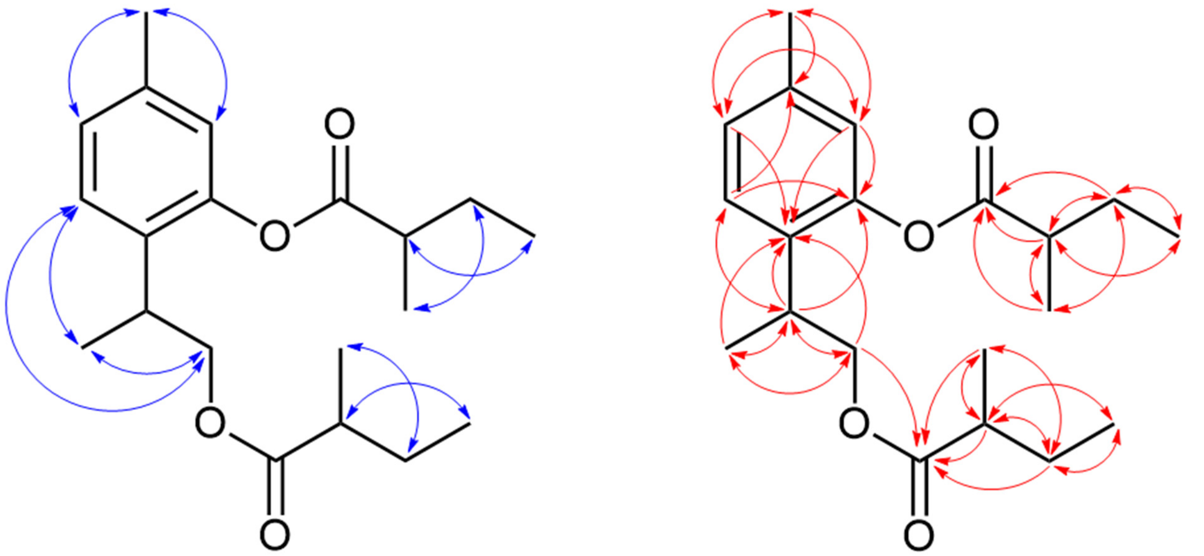

Figure S160. NOESY (blue arrows) and HMBC (red arrows) interactions of 9-(2-methylbutyryloxy)thymyl 2-methylbutyrate.

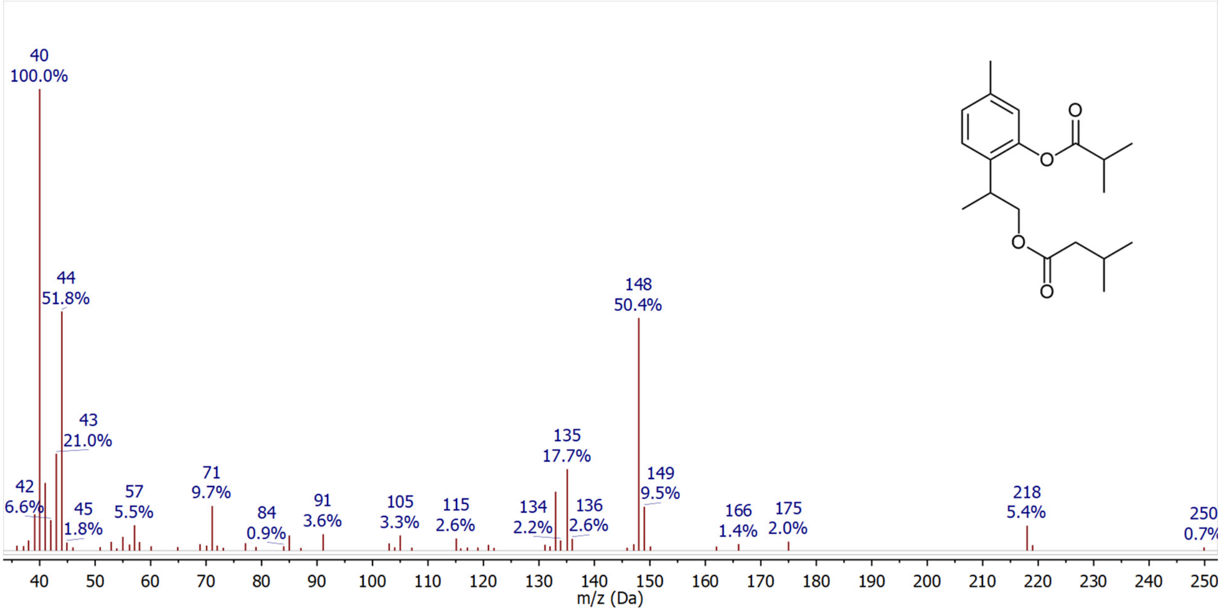

Figure S161. Mass spectrum (EI, 70 eV) of 9-isovaleroxythymyl isobutyrate.

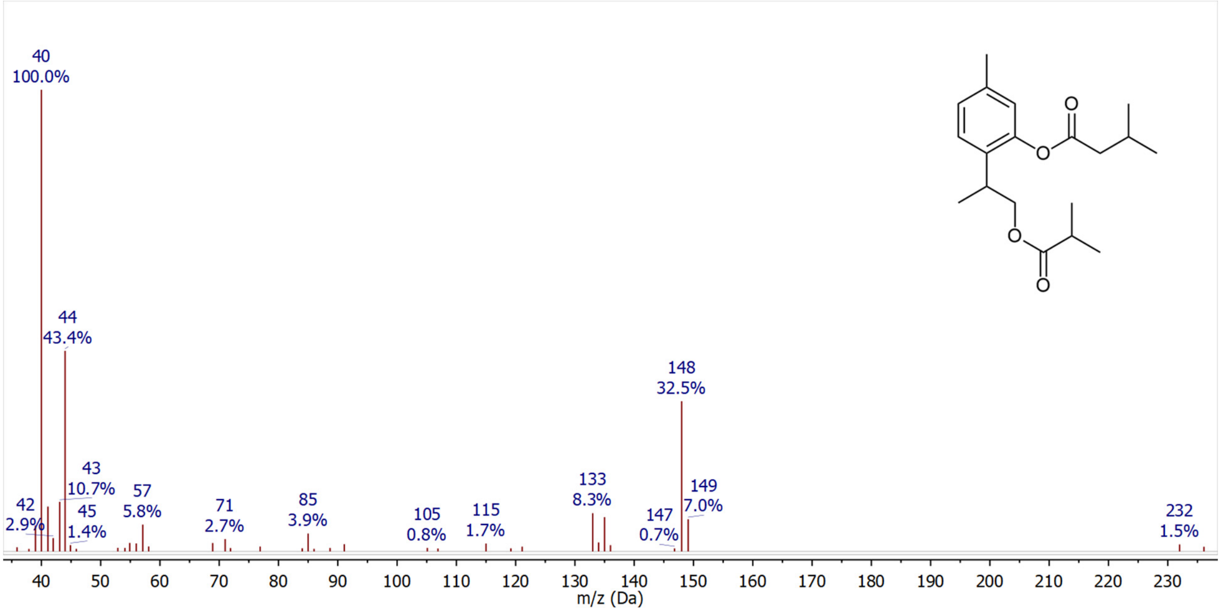

Figure S162. Mass spectrum (EI, 70 eV) of 9-isobutyryloxythymyl isovalerate.

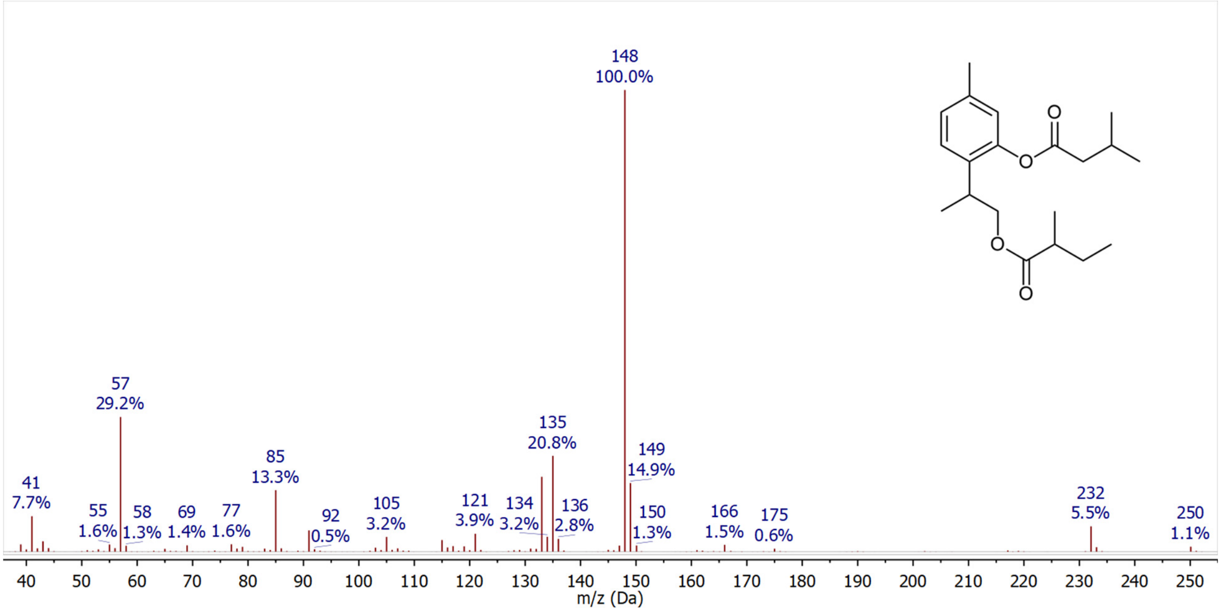

Figure S163. Mass spectrum (EI, 70 eV) of 9-(2-methylbutyryloxy)thymyl isovalerate.

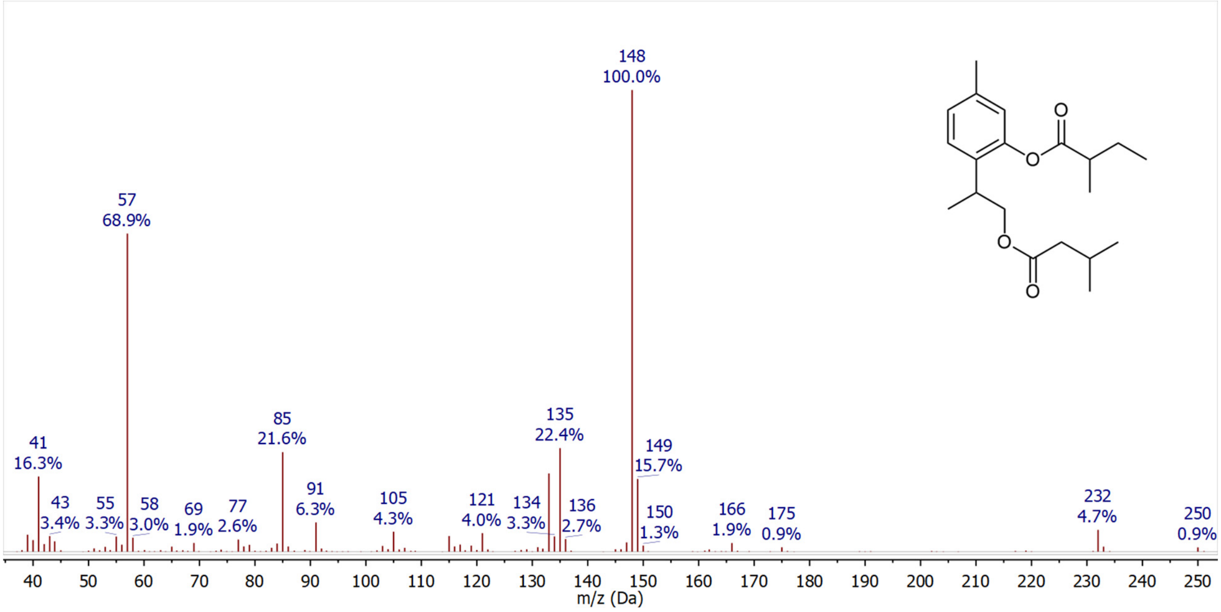

Figure S164. Mass spectrum (EI, 70 eV) of 9-isovaleroxythymyl 2-methylbutyrate.

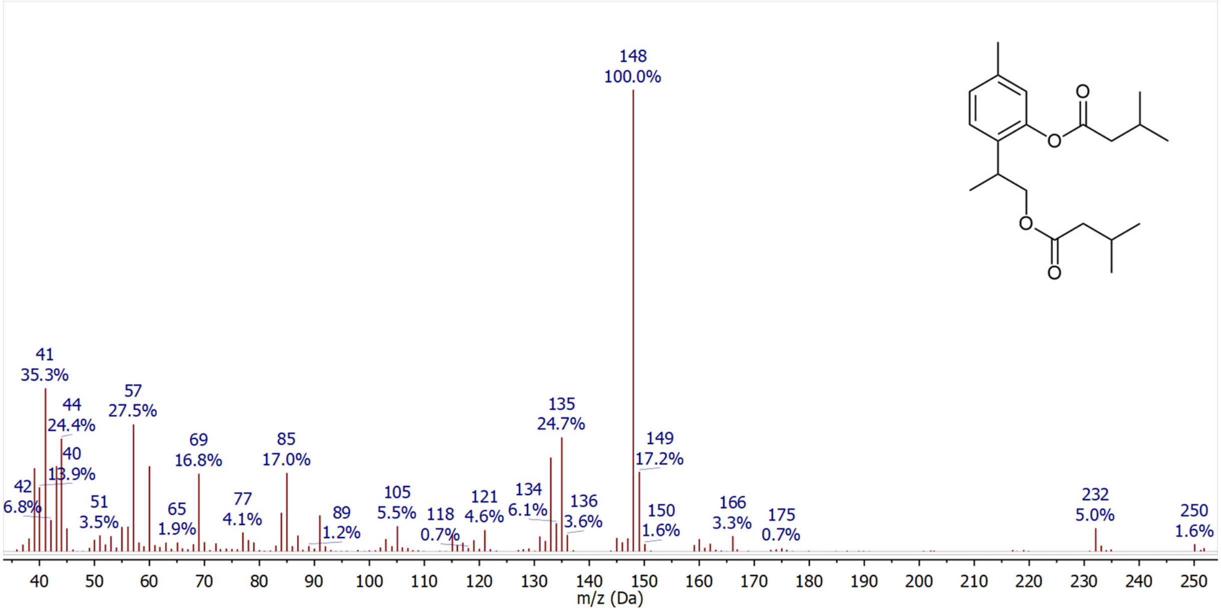

Figure S165. Mass spectrum (EI, 70 eV) of 9-isovaleroxythymyl isovalerate.

Table S27. Retention indices of model esters.

Compound	RIa[a]	RIp[b]
Thymyl isobutyrate (Thy-iPr)	1487	1904
Thymyl 2-methylbutyrate (Thy-sBu)	1578	1992
Thymyl isovalerate (Thy-iBu)	1581	2004
Carvacryl isobutyrate (Carv-iPr)	1515	1940
Carvacryl 2-methylbutyrate (Carv-sBu)	1607	2027
Carvacryl isovalerate (Carv-iBu)	1608	2041
Cuminyl isobutyrate (Cum-iPr)	1554	2019
Cuminyl 2-methylbutyrate (Cum-sBu)	1641	2098
Cuminyl isovalerate (Cum-iBu)	1652	2125
p-Cymene-8-yl isobutyrate (Cym8-iPr)	1466	1878
p-Cymene-8-yl 2-methylbutyrate (Cym8-sBu)	1558	1967
p-Cymene-8-yl isovalerate (Cym8-iBu)	1567	1992
p-Cymene-9-yl isobutyrate (Cym9-iPr)	1540	1987
p-Cymene-9-yl 2-methylbutyrate (Cym9-sBu)	1629	2074
p-Cymene-9-yl isovalerate (Cym9-iBu)	1633	2092

[a] Retention indices determined relative to a homologous series of *n*-alkanes (C_8–C_{30}) on a non-polar DB-5MS column.

[b] Experimental retention indices determined relative to a homologous series of *n*-alkanes (C_8–C_{30}) on a polar HP-Innowax GC column.

Table S28. Residual retention-index increments obtained by subtracting model ester RI values from the corresponding thymol diester RI values.

Diester	Model ester subtracted	Residual increment	R = iPr		R = sBu		R = iBu	
			ΔRIa[a]	ΔRIp[b]	ΔRIa	ΔRIp	ΔRIa	ΔRIp
2-iPr-3R		2-iPr	415	571	426	576	399	540
2-sBu-3R	Thy-R	2-sBu	510	655	511	653	489	620
2-iBu-3R		2-iBu	488	634	492	632	474	605
2R-3-iPr		3-iPr	387	535	390	532	367	497
2R-3-sBu	Carv-R	3-sBu	489	628	482	618	462	583
2R-3-iBu		3-iBu	465	604	463	597	447	568

5-iPr-3R		5-iPr = 3-iPr[c]	446	597	436	595	447	593
5-sBu-3R	Thy-R	5-sBu = 3-sBu[c]	527	683	543	680	542	677
5-iBu-3R		5-iBu = 3-iBu[c]	541	693	545	689	545	685
6-iPr-3R		6-iPr	465	614	466	612	466	611
6-sBu-3R	Thy-R	6-sBu	558	700	564	700	562	698
6-iBu-3R		6-iBu	563	715	566	710	564	709
6R-3-iPr		3-iPr	437	578	438	577	442	578
6R-3-sBu	Carv-R	3-sBu	529	664	535	665	536	661
6R-3-iBu		3-iBu	532	675	536	675	537	672
7-iPr-3R		7-iPr	474	664	473	657	476	665
7-sBu-3R	Thy-R	7-sBu	558	739	557	734	561	739
7-iBu-3R		7-iBu	574	774	569	764	569	767
7R-3-iPr		3-iPr	407	549	404	545	409	553
7R-3-sBu	Cum-R	3-sBu	497	630	494	628	495	631
7R-3-iBu		3-iBu	503	650	501	645	498	646
8-iPr-3R		8-iPr	407	563	409	553	393	534
8-sBu-3R	Thy-R	8-sBu	500	648	501	636	/	/
8-iBu-3R		8-iBu	493	662	/	/	487	628
8R-3-iPr		3-iPr	428	589	429	585	413	574
8R-3-sBu	Cym8-R	3-sBu	521	667	521	661	/	/
8R-3-iBu		3-iBu	508	660	/	/	501	640
9-iPr-3R		9-iPr	434	593	432	583	427	590
9-sBu-3R	Thy-R	9-sBu	520	674	517	661	518	670
9-iBu-3R		9-iBu	524	679	519	678	517	676
9R-3-iPr		3-iPr	381	510	378	504	378	491
9R-3-sBu	Cym9-R	3-sBu	470	588	466	579	464	578
9R-3-iBu		3-iBu	468	607	467	588	465	588

[a] Residual retention-index increments obtained on a non-polar DB-5MS column.

[b] Residual retention-index increments obtained on a polar HP-Innowax GC column.

[c] For the 5-substituted series, positions C-3 and C-5 are symmetry-related on the *p*-cymene skeleton; therefore, the corresponding residual increments are equivalent.

Table S29. Deviations of calculated residual retention-index increments from the mean residual increments for each regioisomeric series. δΔRI = ΔRIindividual − $\overline{\Delta RIa}$. Positive values indicate residual increments higher than the corresponding mean value, whereas negative values indicate lower-than-average values.

Diester	Residual increment	R = iPr		R = sBu		R = iBu	
		δΔRIa[a]	δΔRIp[b]	δΔRIa	δΔRIp	δΔRIa	δΔRIp
2-iPr-3R	2-iPr	2	9	13	14	-14	-22
2-sBu-3R	2-sBu	7	12	8	10	-14	-23
2-iBu-3R	2-iBu	3	10	7	8	-11	-19
2R-3-iPr	3-iPr	6	14	9	11	-14	-24
2R-3-sBu	3-sBu	11	18	4	8	-16	-27
2R-3-iBu	3-iBu	7	14	5	7	-11	-22
5-iPr-3R	5-iPr = 3-iPr[c]	3	2	-7	0	4	-2
5-sBu-3R	5-sBu = 3-sBu[c]	-10	3	6	0	5	-3
5-iBu-3R	5-iBu = 3-iBu[c]	-3	4	1	0	1	-4
6-iPr-3R	6-iPr	-1	2	0	0	0	-1
6-sBu-3R	6-sBu	-3	1	3	1	1	-1
6-iBu-3R	6-iBu	-1	4	2	-1	0	-2
6R-3-iPr	3-iPr	-2	0	-1	-1	3	0
6R-3-sBu	3-sBu	-4	1	2	2	3	-2
6R-3-iBu	3-iBu	-3	1	1	1	2	-2
7-iPr-3R	7-iPr	0	2	-1	-5	2	3
7-sBu-3R	7-sBu	-1	2	-2	-3	2	2
7-iBu-3R	7-iBu	3	6	-2	-4	-2	-1
7R-3-iPr	3-iPr	0	0	-3	-4	2	4
7R-3-sBu	3-sBu	2	0	-1	-2	0	1
7R-3-iBu	3-iBu	2	3	0	-2	-3	-1
8-iPr-3R	8-iPr	4	13	6	3	-10	-16
8-sBu-3R	8-sBu	-1	6	1	-6	/	/
8-iBu-3R	8-iBu	3	17	/	/	-3	-17
8R-3-iPr	3-iPr	5	6	6	2	-10	-9
8R-3-sBu	3-sBu	0	3	0	-3	/	/

8R-3-iBu	3-iBu	4	10	/	/	-4	-10
9-iPr-3R	9-iPr	3	4	1	-6	-4	1
9-sBu-3R	9-sBu	2	6	-1	-7	0	2
9-iBu-3R	9-iBu	4	1	-1	0	-3	-2
9R-3-iPr	3-iPr	2	8	-1	2	-1	-11
9R-3-sBu	3-sBu	3	6	-1	-3	-3	-4
9R-3-iBu	3-iBu	1	13	0	-6	-2	-6

[a] Deviations of individual residual retention-index increments from the corresponding mean values on a non-polar DB-5MS column.

[b] Deviations of calculated residual retention-index increments from the mean residual increments obtained on a polar HP-Innowax GC column.

[c] For the 5-substituted series, positions C-3 and C-5 are symmetry-related on the *p*-cymene skeleton; therefore, the corresponding residual increments are equivalent.

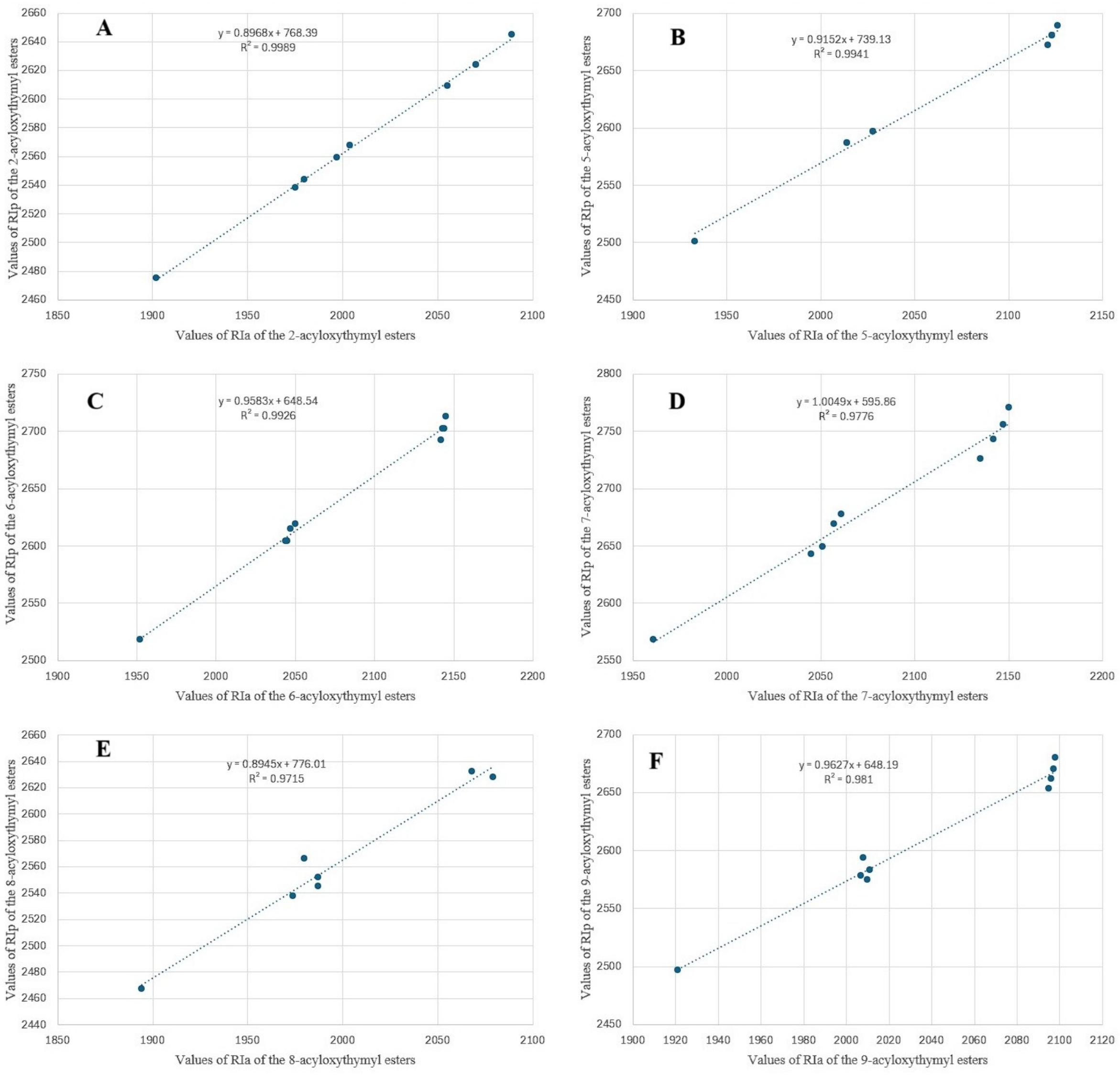

Figure S166. Correlation between retention indices determined on the non-polar DB-5MS column (RIa) and the polar HP-Innowax column (RIp) for the synthesized diacylated oxygenated thymol derivatives. Panels show the corresponding regioisomeric series: (**A**) 2-acyloxythymyl esters, (**B**) 5-acyloxythymyl esters, (**C**) 6-acyloxythymyl esters, (**D**) 7-acyloxythymyl esters, (**E**) 8-acyloxythymyl esters, and (**F**) 9-acyloxythymyl esters. Linear regression equations and R^2 values are shown in each panel. The number of data points was n = 9 for the 2-, 6-, 7-, and 9-substituted series, n = 7 for the 8-substituted series, and n = 6 for the 5-substituted series when symmetry-equivalent compounds were counted once.

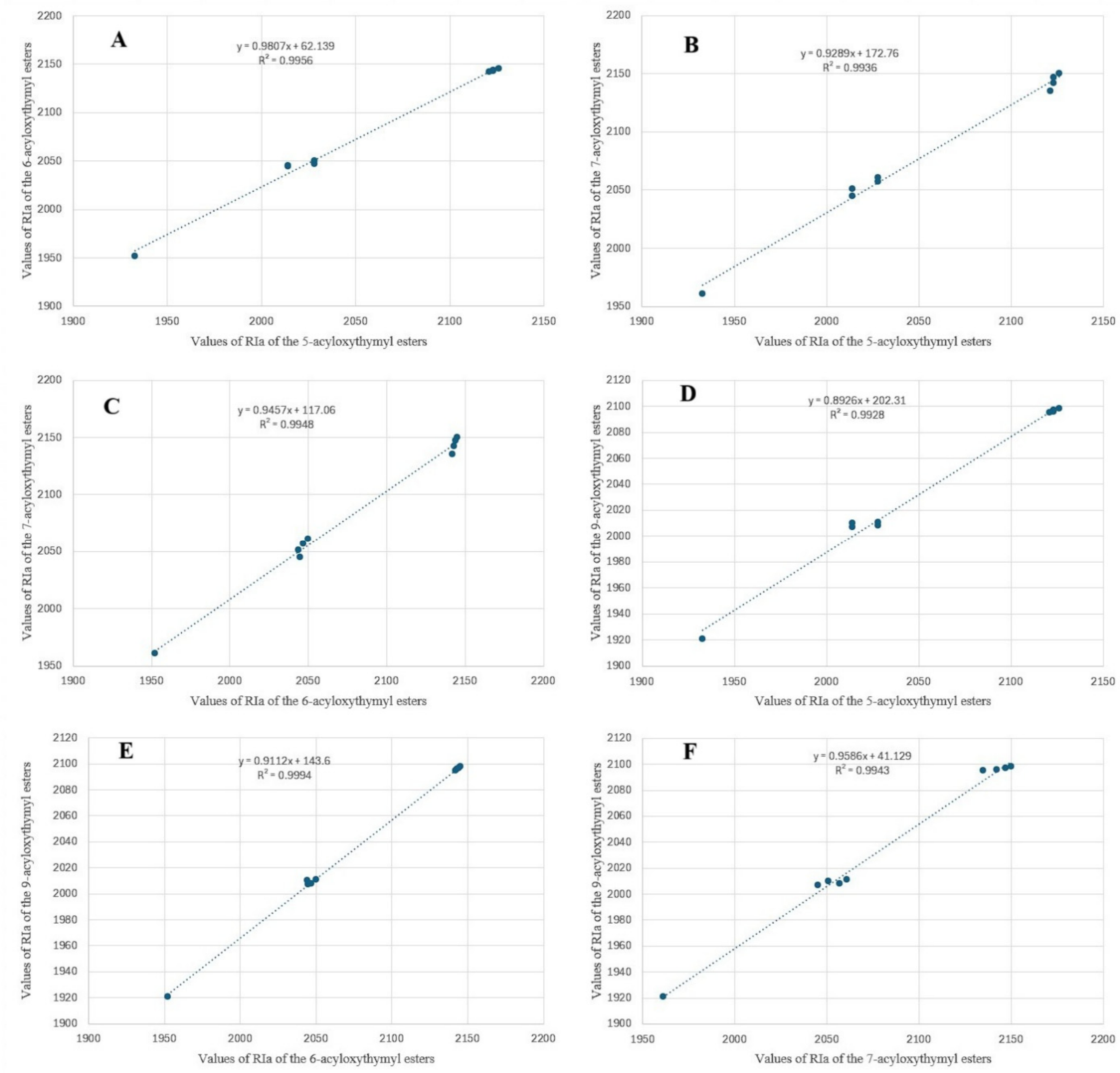

Figure S167. Pairwise correlations of retention indices determined on the non-polar DB-5MS column (RIa) for homologous 5-, 6-, 7-, and 9-substituted diacylated thymol derivatives. Panels show correlations between the corresponding regioisomeric series: (A) 5- vs 6-substituted derivatives, (B) 5- vs 7-substituted derivatives, (C) 6- vs 7-substituted derivatives, (D) 5- vs 9-substituted derivatives, (E) 6- vs 9-substituted derivatives, and (F) 7- vs 9-substituted derivatives. Only compounds with corresponding acyl-residue combinations in both compared series were included. Because the 5-substituted series contains symmetry-equivalent C-3/C-5 combinations, these were treated as equivalent rather than as independent duplicate observations; therefore, each pairwise regression was based on n = 6 data points. Linear regression equations and coefficients of determination (R^2) are shown in each panel.

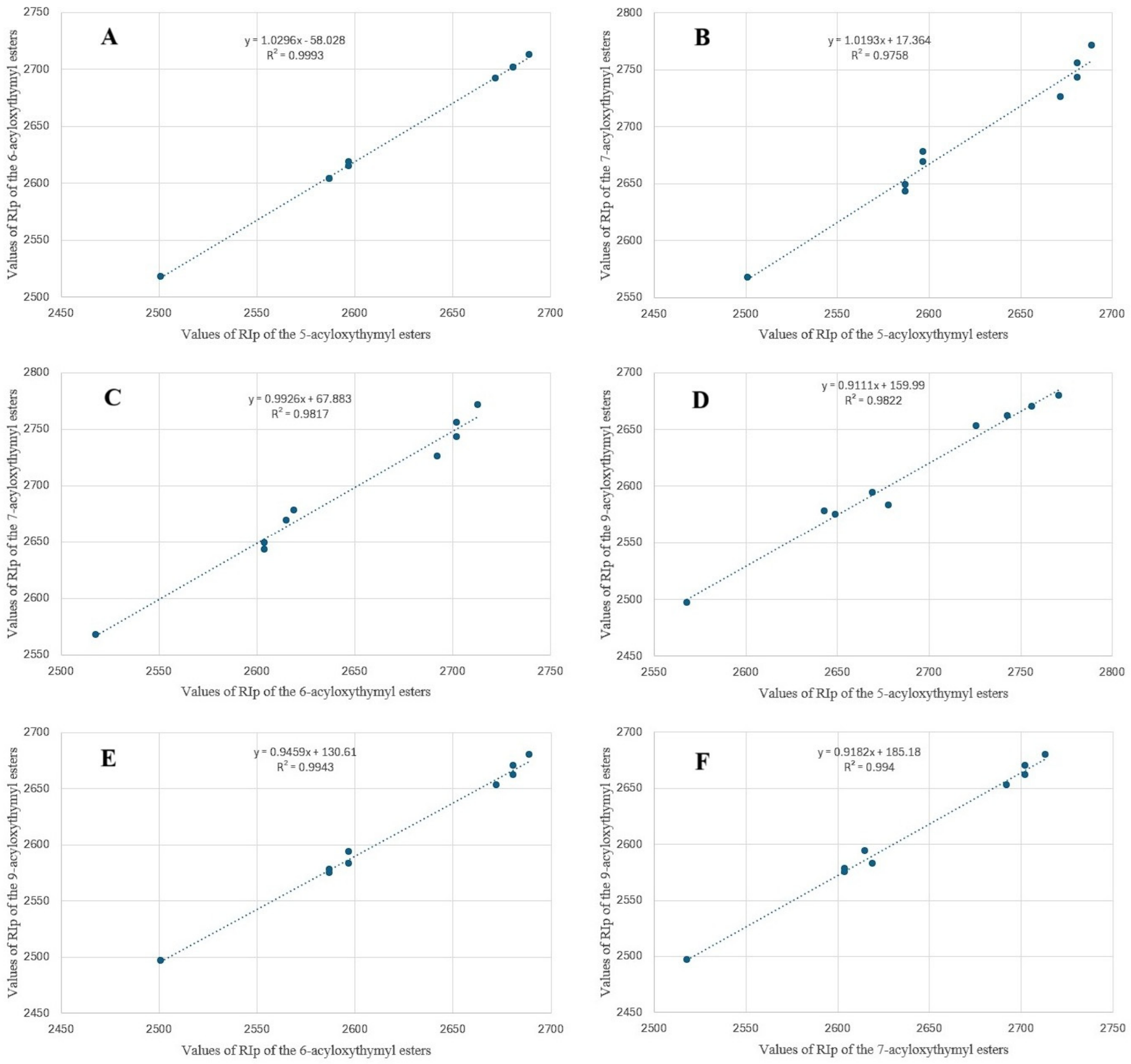

Figure S168. Pairwise correlations of retention indices determined on the polar HP-Innowax column (RIp) for homologous 5-, 6-, 7-, and 9-substituted diacylated thymol derivatives. Panels show correlations between the corresponding regioisomeric series: (**A**) 5- vs 6-substituted derivatives, (**B**) 5- vs 7-substituted derivatives, (**C**) 6- vs 7-substituted derivatives, (**D**) 5- vs 9-substituted derivatives, (**E**) 6- vs 9-substituted derivatives, and (**F**) 7- vs 9-substituted derivatives. Only compounds with corresponding acyl-residue combinations in both compared series were included. Because the 5-substituted series contains symmetry-equivalent C-3/C-5 combinations, these were treated as equivalent rather than as independent duplicate observations; therefore, each pairwise regression was based on n = 6 data points. Linear regression equations and coefficients of determination (R^2) are shown in each panel.

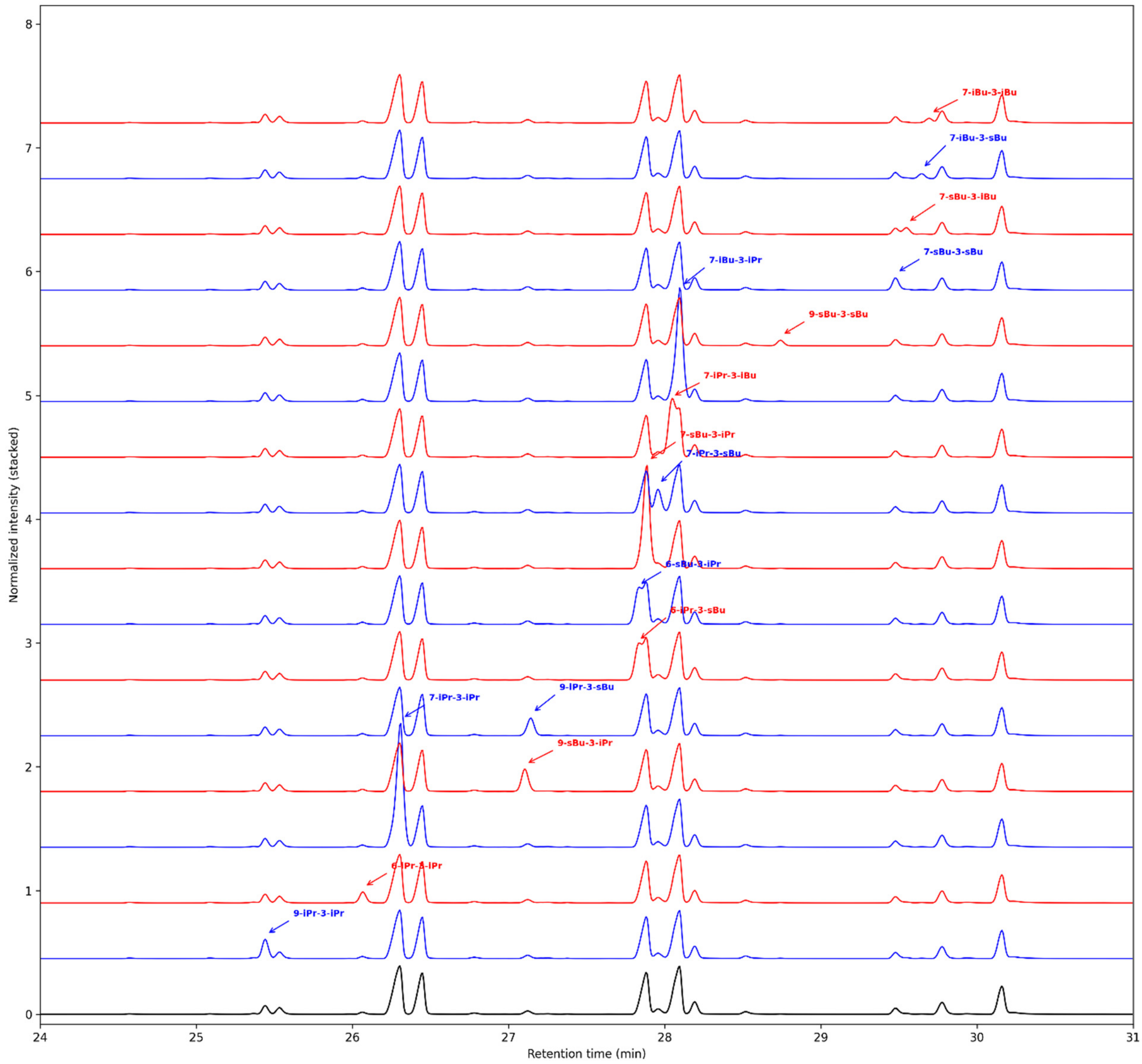

Figure S169. Representative co-injection chromatograms of selected diacylated oxygenated thymol derivatives in the more polar fraction of *D. columnae* root essential oil on the non-polar DB-5MS column. The black trace represents an enlarged total ion chromatogram of the original fraction in the 24–31 min retention-time region, whereas the red and blue traces represent stacked chromatograms obtained after co-injection of the indicated synthetic reference compounds or reference GC–MS components. Peak labels correspond to the compound codes used throughout the manuscript. Co-injection was considered confirmatory when spiking increased only the corresponding chromatographic peak, without formation of a shoulder or secondary peak.

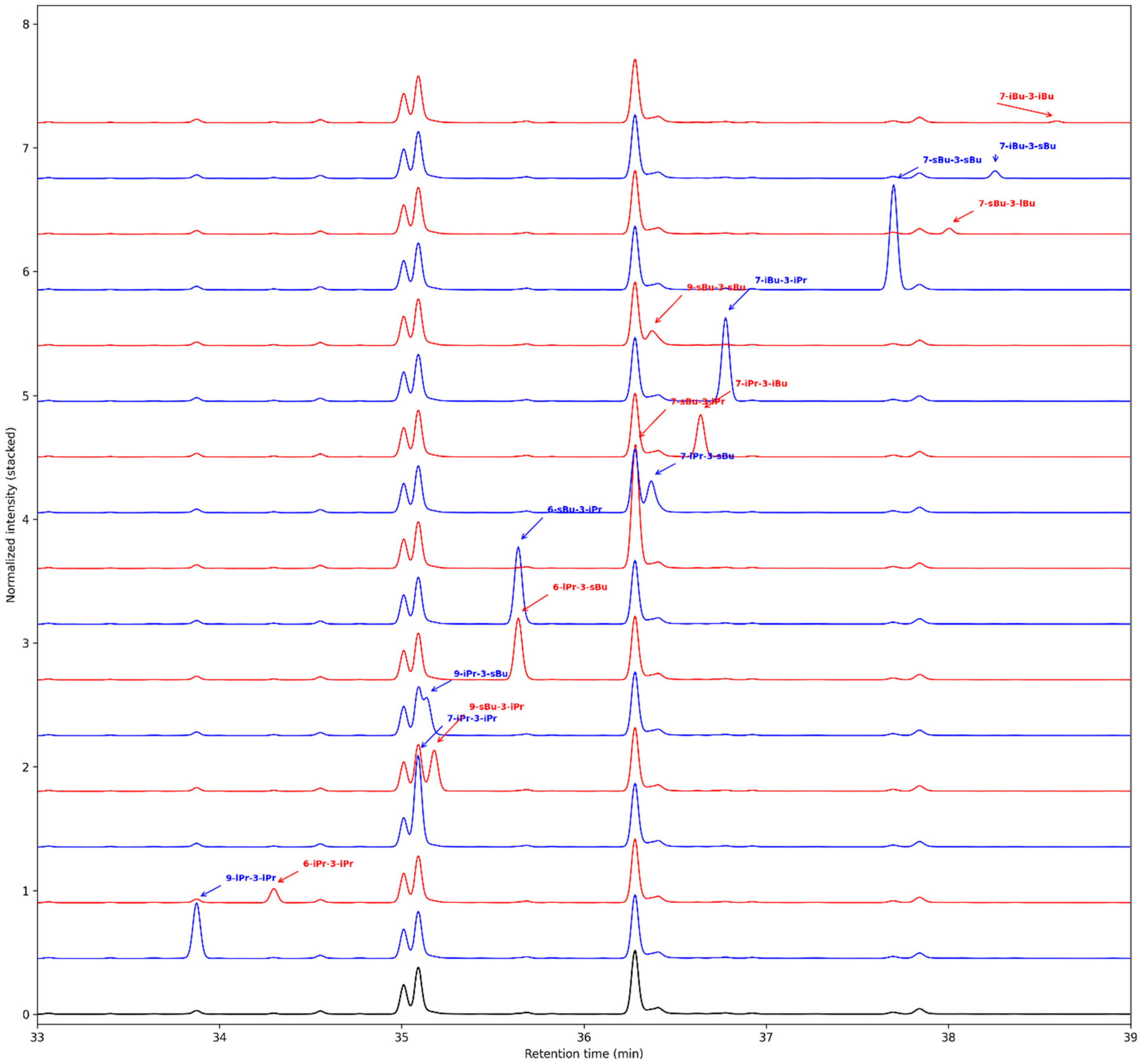

Figure S170. Representative co-injection chromatograms of selected diacylated oxygenated thymol derivatives in the more polar fraction of *D. columnae* root essential oil on the polar HP-Innowax column. The black trace represents an enlarged total ion chromatogram of the original fraction in the 33–39 min retention-time region, whereas the red and blue traces represent stacked chromatograms obtained after co-injection of the indicated synthetic reference compounds or reference GC–MS components. Peak labels correspond to the compound codes used throughout the manuscript. Co-injection was considered confirmatory when spiking increased only the corresponding chromatographic peak, without formation of a shoulder or secondary peak.

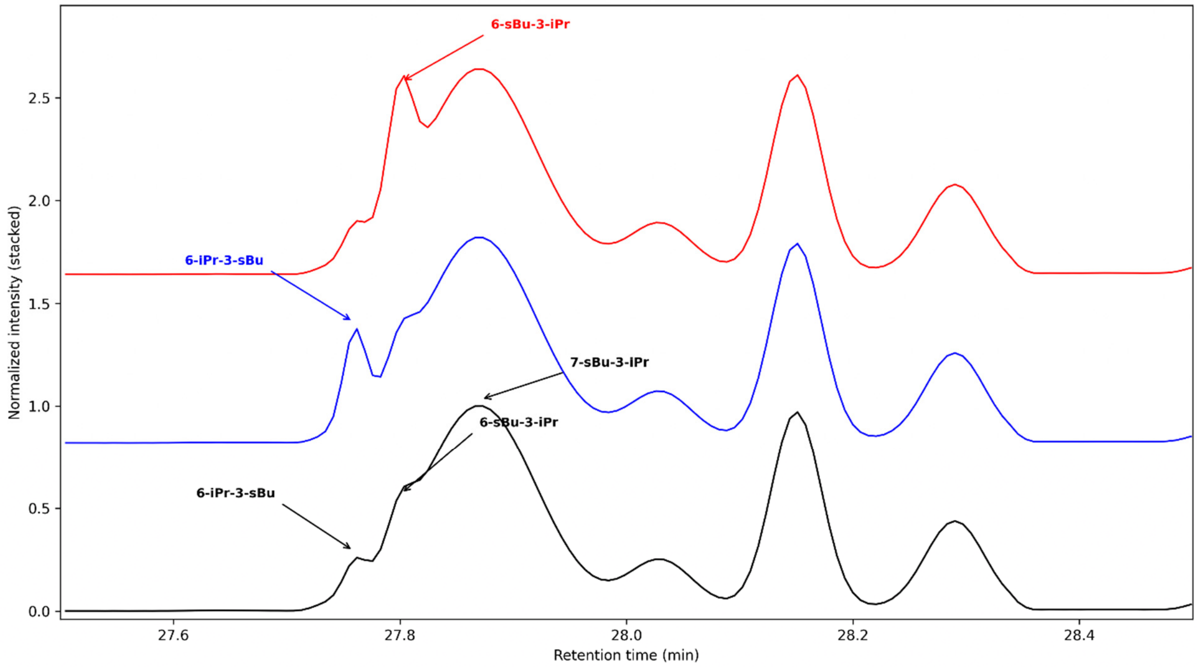

Figure S171. Expanded experimental DB-5MS chromatograms illustrating the identification of 6-iPr-3-sBu and 6-sBu-3-iPr in the more polar fraction of *D. columnae* root essential oil. The black trace represents the unspiked fraction, whereas the blue and red traces represent separate co-injection experiments with 6-iPr-3-sBu and 6-sBu-3-iPr, respectively. The individual additions enhance the corresponding shoulders already present before the larger peak assigned to 7-sBu-3-iPr. The components partially overlap, and baseline separation was not achieved. Normalized traces are vertically offset for clarity. Compound codes correspond to those used throughout the manuscript.
